# Pan-cancer analysis of whole genomes

**DOI:** 10.1038/s41586-020-1969-6

**Published:** 2020-02-05

**Authors:** The ICGC/TCGA Pan-Cancer Analysis of Whole Genomes Consortium, Lauri A. Aaltonen, Federico Abascal, Adam Abeshouse, Hiroyuki Aburatani, David J. Adams, Nishant Agrawal, Keun Soo Ahn, Sung-Min Ahn, Hiroshi Aikata, Rehan Akbani, Kadir C. Akdemir, Hikmat Al-Ahmadie, Sultan T. Al-Sedairy, Fatima Al-Shahrour, Malik Alawi, Monique Albert, Kenneth Aldape, Ludmil B. Alexandrov, Adrian Ally, Kathryn Alsop, Eva G. Alvarez, Fernanda Amary, Samirkumar B. Amin, Brice Aminou, Ole Ammerpohl, Matthew J. Anderson, Yeng Ang, Davide Antonello, Pavana Anur, Samuel Aparicio, Elizabeth L. Appelbaum, Yasuhito Arai, Axel Aretz, Koji Arihiro, Shun-ichi Ariizumi, Joshua Armenia, Laurent Arnould, Sylvia Asa, Yassen Assenov, Gurnit Atwal, Sietse Aukema, J. Todd Auman, Miriam R. R. Aure, Philip Awadalla, Marta Aymerich, Gary D. Bader, Adrian Baez-Ortega, Matthew H. Bailey, Peter J. Bailey, Miruna Balasundaram, Saianand Balu, Pratiti Bandopadhayay, Rosamonde E. Banks, Stefano Barbi, Andrew P. Barbour, Jonathan Barenboim, Jill Barnholtz-Sloan, Hugh Barr, Elisabet Barrera, John Bartlett, Javier Bartolome, Claudio Bassi, Oliver F. Bathe, Daniel Baumhoer, Prashant Bavi, Stephen B. Baylin, Wojciech Bazant, Duncan Beardsmore, Timothy A. Beck, Sam Behjati, Andreas Behren, Beifang Niu, Cindy Bell, Sergi Beltran, Christopher Benz, Andrew Berchuck, Anke K. Bergmann, Erik N. Bergstrom, Benjamin P. Berman, Daniel M. Berney, Stephan H. Bernhart, Rameen Beroukhim, Mario Berrios, Samantha Bersani, Johanna Bertl, Miguel Betancourt, Vinayak Bhandari, Shriram G. Bhosle, Andrew V. Biankin, Matthias Bieg, Darell Bigner, Hans Binder, Ewan Birney, Michael Birrer, Nidhan K. Biswas, Bodil Bjerkehagen, Tom Bodenheimer, Lori Boice, Giada Bonizzato, Johann S. De Bono, Arnoud Boot, Moiz S. Bootwalla, Ake Borg, Arndt Borkhardt, Keith A. Boroevich, Ivan Borozan, Christoph Borst, Marcus Bosenberg, Mattia Bosio, Jacqueline Boultwood, Guillaume Bourque, Paul C. Boutros, G. Steven Bova, David T. Bowen, Reanne Bowlby, David D. L. Bowtell, Sandrine Boyault, Rich Boyce, Jeffrey Boyd, Alvis Brazma, Paul Brennan, Daniel S. Brewer, Arie B. Brinkman, Robert G. Bristow, Russell R. Broaddus, Jane E. Brock, Malcolm Brock, Annegien Broeks, Angela N. Brooks, Denise Brooks, Benedikt Brors, Søren Brunak, Timothy J. C. Bruxner, Alicia L. Bruzos, Alex Buchanan, Ivo Buchhalter, Christiane Buchholz, Susan Bullman, Hazel Burke, Birgit Burkhardt, Kathleen H. Burns, John Busanovich, Carlos D. Bustamante, Adam P. Butler, Atul J. Butte, Niall J. Byrne, Anne-Lise Børresen-Dale, Samantha J. Caesar-Johnson, Andy Cafferkey, Declan Cahill, Claudia Calabrese, Carlos Caldas, Fabien Calvo, Niedzica Camacho, Peter J. Campbell, Elias Campo, Cinzia Cantù, Shaolong Cao, Thomas E. Carey, Joana Carlevaro-Fita, Rebecca Carlsen, Ivana Cataldo, Mario Cazzola, Jonathan Cebon, Robert Cerfolio, Dianne E. Chadwick, Dimple Chakravarty, Don Chalmers, Calvin Wing Yiu Chan, Kin Chan, Michelle Chan-Seng-Yue, Vishal S. Chandan, David K. Chang, Stephen J. Chanock, Lorraine A. Chantrill, Aurélien Chateigner, Nilanjan Chatterjee, Kazuaki Chayama, Hsiao-Wei Chen, Jieming Chen, Ken Chen, Yiwen Chen, Zhaohong Chen, Andrew D. Cherniack, Jeremy Chien, Yoke-Eng Chiew, Suet-Feung Chin, Juok Cho, Sunghoon Cho, Jung Kyoon Choi, Wan Choi, Christine Chomienne, Zechen Chong, Su Pin Choo, Angela Chou, Angelika N. Christ, Elizabeth L. Christie, Eric Chuah, Carrie Cibulskis, Kristian Cibulskis, Sara Cingarlini, Peter Clapham, Alexander Claviez, Sean Cleary, Nicole Cloonan, Marek Cmero, Colin C. Collins, Ashton A. Connor, Susanna L. Cooke, Colin S. Cooper, Leslie Cope, Vincenzo Corbo, Matthew G. Cordes, Stephen M. Cordner, Isidro Cortés-Ciriano, Kyle Covington, Prue A. Cowin, Brian Craft, David Craft, Chad J. Creighton, Yupeng Cun, Erin Curley, Ioana Cutcutache, Karolina Czajka, Bogdan Czerniak, Rebecca A. Dagg, Ludmila Danilova, Maria Vittoria Davi, Natalie R. Davidson, Helen Davies, Ian J. Davis, Brandi N. Davis-Dusenbery, Kevin J. Dawson, Francisco M. De La Vega, Ricardo De Paoli-Iseppi, Timothy Defreitas, Angelo P. Dei Tos, Olivier Delaneau, John A. Demchok, Jonas Demeulemeester, German M. Demidov, Deniz Demircioğlu, Nening M. Dennis, Robert E. Denroche, Stefan C. Dentro, Nikita Desai, Vikram Deshpande, Amit G. Deshwar, Christine Desmedt, Jordi Deu-Pons, Noreen Dhalla, Neesha C. Dhani, Priyanka Dhingra, Rajiv Dhir, Anthony DiBiase, Klev Diamanti, Li Ding, Shuai Ding, Huy Q. Dinh, Luc Dirix, HarshaVardhan Doddapaneni, Nilgun Donmez, Michelle T. Dow, Ronny Drapkin, Oliver Drechsel, Ruben M. Drews, Serge Serge, Tim Dudderidge, Ana Dueso-Barroso, Andrew J. Dunford, Michael Dunn, Lewis Jonathan Dursi, Fraser R. Duthie, Ken Dutton-Regester, Jenna Eagles, Douglas F. Easton, Stuart Edmonds, Paul A. Edwards, Sandra E. Edwards, Rosalind A. Eeles, Anna Ehinger, Juergen Eils, Roland Eils, Adel El-Naggar, Matthew Eldridge, Kyle Ellrott, Serap Erkek, Georgia Escaramis, Shadrielle M. G. Espiritu, Xavier Estivill, Dariush Etemadmoghadam, Jorunn E. Eyfjord, Bishoy M. Faltas, Daiming Fan, Yu Fan, William C. Faquin, Claudiu Farcas, Matteo Fassan, Aquila Fatima, Francesco Favero, Nodirjon Fayzullaev, Ina Felau, Sian Fereday, Martin L. Ferguson, Vincent Ferretti, Lars Feuerbach, Matthew A. Field, J. Lynn Fink, Gaetano Finocchiaro, Cyril Fisher, Matthew W. Fittall, Anna Fitzgerald, Rebecca C. Fitzgerald, Adrienne M. Flanagan, Neil E. Fleshner, Paul Flicek, John A. Foekens, Kwun M. Fong, Nuno A. Fonseca, Christopher S. Foster, Natalie S. Fox, Michael Fraser, Scott Frazer, Milana Frenkel-Morgenstern, William Friedman, Joan Frigola, Catrina C. Fronick, Akihiro Fujimoto, Masashi Fujita, Masashi Fukayama, Lucinda A. Fulton, Robert S. Fulton, Mayuko Furuta, P. Andrew Futreal, Anja Füllgrabe, Stacey B. Gabriel, Steven Gallinger, Carlo Gambacorti-Passerini, Jianjiong Gao, Shengjie Gao, Levi Garraway, Øystein Garred, Erik Garrison, Dale W. Garsed, Nils Gehlenborg, Josep L. L. Gelpi, Joshy George, Daniela S. Gerhard, Clarissa Gerhauser, Jeffrey E. Gershenwald, Mark Gerstein, Moritz Gerstung, Gad Getz, Mohammed Ghori, Ronald Ghossein, Nasra H. Giama, Richard A. Gibbs, Bob Gibson, Anthony J. Gill, Pelvender Gill, Dilip D. Giri, Dominik Glodzik, Vincent J. Gnanapragasam, Maria Elisabeth Goebler, Mary J. Goldman, Carmen Gomez, Santiago Gonzalez, Abel Gonzalez-Perez, Dmitry A. Gordenin, James Gossage, Kunihito Gotoh, Ramaswamy Govindan, Dorthe Grabau, Janet S. Graham, Robert C. Grant, Anthony R. Green, Eric Green, Liliana Greger, Nicola Grehan, Sonia Grimaldi, Sean M. Grimmond, Robert L. Grossman, Adam Grundhoff, Gunes Gundem, Qianyun Guo, Manaswi Gupta, Shailja Gupta, Ivo G. Gut, Marta Gut, Jonathan Göke, Gavin Ha, Andrea Haake, David Haan, Siegfried Haas, Kerstin Haase, James E. Haber, Nina Habermann, Faraz Hach, Syed Haider, Natsuko Hama, Freddie C. Hamdy, Anne Hamilton, Mark P. Hamilton, Leng Han, George B. Hanna, Martin Hansmann, Nicholas J. Haradhvala, Olivier Harismendy, Ivon Harliwong, Arif O. Harmanci, Eoghan Harrington, Takanori Hasegawa, David Haussler, Steve Hawkins, Shinya Hayami, Shuto Hayashi, D. Neil Hayes, Stephen J. Hayes, Nicholas K. Hayward, Steven Hazell, Yao He, Allison P. Heath, Simon C. Heath, David Hedley, Apurva M. Hegde, David I. Heiman, Michael C. Heinold, Zachary Heins, Lawrence E. Heisler, Eva Hellstrom-Lindberg, Mohamed Helmy, Seong Gu Heo, Austin J. Hepperla, José María Heredia-Genestar, Carl Herrmann, Peter Hersey, Julian M. Hess, Holmfridur Hilmarsdottir, Jonathan Hinton, Satoshi Hirano, Nobuyoshi Hiraoka, Katherine A. Hoadley, Asger Hobolth, Ermin Hodzic, Jessica I. Hoell, Steve Hoffmann, Oliver Hofmann, Andrea Holbrook, Aliaksei Z. Holik, Michael A. Hollingsworth, Oliver Holmes, Robert A. Holt, Chen Hong, Eun Pyo Hong, Jongwhi H. Hong, Gerrit K. Hooijer, Henrik Hornshøj, Fumie Hosoda, Yong Hou, Volker Hovestadt, William Howat, Alan P. Hoyle, Ralph H. Hruban, Jianhong Hu, Taobo Hu, Xing Hua, Kuan-lin Huang, Mei Huang, Mi Ni Huang, Vincent Huang, Yi Huang, Wolfgang Huber, Thomas J. Hudson, Michael Hummel, Jillian A. Hung, David Huntsman, Ted R. Hupp, Jason Huse, Matthew R. Huska, Barbara Hutter, Carolyn M. Hutter, Daniel Hübschmann, Christine A. Iacobuzio-Donahue, Charles David Imbusch, Marcin Imielinski, Seiya Imoto, William B. Isaacs, Keren Isaev, Shumpei Ishikawa, Murat Iskar, S. M. Ashiqul Islam, Michael Ittmann, Sinisa Ivkovic, Jose M. G. Izarzugaza, Jocelyne Jacquemier, Valerie Jakrot, Nigel B. Jamieson, Gun Ho Jang, Se Jin Jang, Joy C. Jayaseelan, Reyka Jayasinghe, Stuart R. Jefferys, Karine Jegalian, Jennifer L. Jennings, Seung-Hyup Jeon, Lara Jerman, Yuan Ji, Wei Jiao, Peter A. Johansson, Amber L. Johns, Jeremy Johns, Rory Johnson, Todd A. Johnson, Clemency Jolly, Yann Joly, Jon G. Jonasson, Corbin D. Jones, David R. Jones, David T. W. Jones, Nic Jones, Steven J. M. Jones, Jos Jonkers, Young Seok Ju, Hartmut Juhl, Jongsun Jung, Malene Juul, Randi Istrup Juul, Sissel Juul, Natalie Jäger, Rolf Kabbe, Andre Kahles, Abdullah Kahraman, Vera B. Kaiser, Hojabr Kakavand, Sangeetha Kalimuthu, Christof von Kalle, Koo Jeong Kang, Katalin Karaszi, Beth Karlan, Rosa Karlić, Dennis Karsch, Katayoon Kasaian, Karin S. Kassahn, Hitoshi Katai, Mamoru Kato, Hiroto Katoh, Yoshiiku Kawakami, Jonathan D. Kay, Stephen H. Kazakoff, Marat D. Kazanov, Maria Keays, Electron Kebebew, Richard F. Kefford, Manolis Kellis, James G. Kench, Catherine J. Kennedy, Jules N. A. Kerssemakers, David Khoo, Vincent Khoo, Narong Khuntikeo, Ekta Khurana, Helena Kilpinen, Hark Kyun Kim, Hyung-Lae Kim, Hyung-Yong Kim, Hyunghwan Kim, Jaegil Kim, Jihoon Kim, Jong K. Kim, Youngwook Kim, Tari A. King, Wolfram Klapper, Kortine Kleinheinz, Leszek J. Klimczak, Stian Knappskog, Michael Kneba, Bartha M. Knoppers, Youngil Koh, Jan Komorowski, Daisuke Komura, Mitsuhiro Komura, Gu Kong, Marcel Kool, Jan O. Korbel, Viktoriya Korchina, Andrey Korshunov, Michael Koscher, Roelof Koster, Zsofia Kote-Jarai, Antonios Koures, Milena Kovacevic, Barbara Kremeyer, Helene Kretzmer, Markus Kreuz, Savitri Krishnamurthy, Dieter Kube, Kiran Kumar, Pardeep Kumar, Sushant Kumar, Yogesh Kumar, Ritika Kundra, Kirsten Kübler, Ralf Küppers, Jesper Lagergren, Phillip H. Lai, Peter W. Laird, Sunil R. Lakhani, Christopher M. Lalansingh, Emilie Lalonde, Fabien C. Lamaze, Adam Lambert, Eric Lander, Pablo Landgraf, Luca Landoni, Anita Langerød, Andrés Lanzós, Denis Larsimont, Erik Larsson, Mark Lathrop, Loretta M. S. Lau, Chris Lawerenz, Rita T. Lawlor, Michael S. Lawrence, Alexander J. Lazar, Ana Mijalkovic Lazic, Xuan Le, Darlene Lee, Donghoon Lee, Eunjung Alice Lee, Hee Jin Lee, Jake June-Koo Lee, Jeong-Yeon Lee, Juhee Lee, Ming Ta Michael Lee, Henry Lee-Six, Kjong-Van Lehmann, Hans Lehrach, Dido Lenze, Conrad R. Leonard, Daniel A. Leongamornlert, Ignaty Leshchiner, Louis Letourneau, Ivica Letunic, Douglas A. Levine, Lora Lewis, Tim Ley, Chang Li, Constance H. Li, Haiyan Irene Li, Jun Li, Lin Li, Shantao Li, Siliang Li, Xiaobo Li, Xiaotong Li, Xinyue Li, Yilong Li, Han Liang, Sheng-Ben Liang, Peter Lichter, Pei Lin, Ziao Lin, W. M. Linehan, Ole Christian Lingjærde, Dongbing Liu, Eric Minwei Liu, Fei-Fei Fei Liu, Fenglin Liu, Jia Liu, Xingmin Liu, Julie Livingstone, Dimitri Livitz, Naomi Livni, Lucas Lochovsky, Markus Loeffler, Georgina V. Long, Armando Lopez-Guillermo, Shaoke Lou, David N. Louis, Laurence B. Lovat, Yiling Lu, Yong-Jie Lu, Youyong Lu, Claudio Luchini, Ilinca Lungu, Xuemei Luo, Hayley J. Luxton, Andy G. Lynch, Lisa Lype, Cristina López, Carlos López-Otín, Eric Z. Ma, Yussanne Ma, Gaetan MacGrogan, Shona MacRae, Geoff Macintyre, Tobias Madsen, Kazuhiro Maejima, Andrea Mafficini, Dennis T. Maglinte, Arindam Maitra, Partha P. Majumder, Luca Malcovati, Salem Malikic, Giuseppe Malleo, Graham J. Mann, Luisa Mantovani-Löffler, Kathleen Marchal, Giovanni Marchegiani, Elaine R. Mardis, Adam A. Margolin, Maximillian G. Marin, Florian Markowetz, Julia Markowski, Jeffrey Marks, Tomas Marques-Bonet, Marco A. Marra, Luke Marsden, John W. M. Martens, Sancha Martin, Jose I. Martin-Subero, Iñigo Martincorena, Alexander Martinez-Fundichely, Yosef E. Maruvka, R. Jay Mashl, Charlie E. Massie, Thomas J. Matthew, Lucy Matthews, Erik Mayer, Simon Mayes, Michael Mayo, Faridah Mbabaali, Karen McCune, Ultan McDermott, Patrick D. McGillivray, Michael D. McLellan, John D. McPherson, John R. McPherson, Treasa A. McPherson, Samuel R. Meier, Alice Meng, Shaowu Meng, Andrew Menzies, Neil D. Merrett, Sue Merson, Matthew Meyerson, William Meyerson, Piotr A. Mieczkowski, George L. Mihaiescu, Sanja Mijalkovic, Tom Mikkelsen, Michele Milella, Linda Mileshkin, Christopher A. Miller, David K. Miller, Jessica K. Miller, Gordon B. Mills, Ana Milovanovic, Sarah Minner, Marco Miotto, Gisela Mir Arnau, Lisa Mirabello, Chris Mitchell, Thomas J. Mitchell, Satoru Miyano, Naoki Miyoshi, Shinichi Mizuno, Fruzsina Molnár-Gábor, Malcolm J. Moore, Richard A. Moore, Sandro Morganella, Quaid D. Morris, Carl Morrison, Lisle E. Mose, Catherine D. Moser, Ferran Muiños, Loris Mularoni, Andrew J. Mungall, Karen Mungall, Elizabeth A. Musgrove, Ville Mustonen, David Mutch, Francesc Muyas, Donna M. Muzny, Alfonso Muñoz, Jerome Myers, Ola Myklebost, Peter Möller, Genta Nagae, Adnan M. Nagrial, Hardeep K. Nahal-Bose, Hitoshi Nakagama, Hidewaki Nakagawa, Hiromi Nakamura, Toru Nakamura, Kaoru Nakano, Tannistha Nandi, Jyoti Nangalia, Mia Nastic, Arcadi Navarro, Fabio C. P. Navarro, David E. Neal, Gerd Nettekoven, Felicity Newell, Steven J. Newhouse, Yulia Newton, Alvin Wei Tian Ng, Anthony Ng, Jonathan Nicholson, David Nicol, Yongzhan Nie, G. Petur Nielsen, Morten Muhlig Nielsen, Serena Nik-Zainal, Michael S. Noble, Katia Nones, Paul A. Northcott, Faiyaz Notta, Brian D. O’Connor, Peter O’Donnell, Maria O’Donovan, Sarah O’Meara, Brian Patrick O’Neill, J. Robert O’Neill, David Ocana, Angelica Ochoa, Layla Oesper, Christopher Ogden, Hideki Ohdan, Kazuhiro Ohi, Lucila Ohno-Machado, Karin A. Oien, Akinyemi I. Ojesina, Hidenori Ojima, Takuji Okusaka, Larsson Omberg, Choon Kiat Ong, Stephan Ossowski, German Ott, B. F. Francis Ouellette, Christine P’ng, Marta Paczkowska, Salvatore Paiella, Chawalit Pairojkul, Marina Pajic, Qiang Pan-Hammarström, Elli Papaemmanuil, Irene Papatheodorou, Nagarajan Paramasivam, Ji Wan Park, Joong-Won Park, Keunchil Park, Kiejung Park, Peter J. Park, Joel S. Parker, Simon L. Parsons, Harvey Pass, Danielle Pasternack, Alessandro Pastore, Ann-Marie Patch, Iris Pauporté, Antonio Pea, John V. Pearson, Chandra Sekhar Pedamallu, Jakob Skou Pedersen, Paolo Pederzoli, Martin Peifer, Nathan A. Pennell, Charles M. Perou, Marc D. Perry, Gloria M. Petersen, Myron Peto, Nicholas Petrelli, Robert Petryszak, Stefan M. Pfister, Mark Phillips, Oriol Pich, Hilda A. Pickett, Todd D. Pihl, Nischalan Pillay, Sarah Pinder, Mark Pinese, Andreia V. Pinho, Esa Pitkänen, Xavier Pivot, Elena Piñeiro-Yáñez, Laura Planko, Christoph Plass, Paz Polak, Tirso Pons, Irinel Popescu, Olga Potapova, Aparna Prasad, Shaun R. Preston, Manuel Prinz, Antonia L. Pritchard, Stephenie D. Prokopec, Elena Provenzano, Xose S. Puente, Sonia Puig, Montserrat Puiggròs, Sergio Pulido-Tamayo, Gulietta M. Pupo, Colin A. Purdie, Michael C. Quinn, Raquel Rabionet, Janet S. Rader, Bernhard Radlwimmer, Petar Radovic, Benjamin Raeder, Keiran M. Raine, Manasa Ramakrishna, Kamna Ramakrishnan, Suresh Ramalingam, Benjamin J. Raphael, W. Kimryn Rathmell, Tobias Rausch, Guido Reifenberger, Jüri Reimand, Jorge Reis-Filho, Victor Reuter, Iker Reyes-Salazar, Matthew A. Reyna, Sheila M. Reynolds, Esther Rheinbay, Yasser Riazalhosseini, Andrea L. Richardson, Julia Richter, Matthew Ringel, Markus Ringnér, Yasushi Rino, Karsten Rippe, Jeffrey Roach, Lewis R. Roberts, Nicola D. Roberts, Steven A. Roberts, A. Gordon Robertson, Alan J. Robertson, Javier Bartolomé Rodriguez, Bernardo Rodriguez-Martin, F. Germán Rodríguez-González, Michael H. A. Roehrl, Marius Rohde, Hirofumi Rokutan, Gilles Romieu, Ilse Rooman, Tom Roques, Daniel Rosebrock, Mara Rosenberg, Philip C. Rosenstiel, Andreas Rosenwald, Edward W. Rowe, Romina Royo, Steven G. Rozen, Yulia Rubanova, Mark A. Rubin, Carlota Rubio-Perez, Vasilisa A. Rudneva, Borislav C. Rusev, Andrea Ruzzenente, Gunnar Rätsch, Radhakrishnan Sabarinathan, Veronica Y. Sabelnykova, Sara Sadeghi, S. Cenk Sahinalp, Natalie Saini, Mihoko Saito-Adachi, Gordon Saksena, Adriana Salcedo, Roberto Salgado, Leonidas Salichos, Richard Sallari, Charles Saller, Roberto Salvia, Michelle Sam, Jaswinder S. Samra, Francisco Sanchez-Vega, Chris Sander, Grant Sanders, Rajiv Sarin, Iman Sarrafi, Aya Sasaki-Oku, Torill Sauer, Guido Sauter, Robyn P. M. Saw, Maria Scardoni, Christopher J. Scarlett, Aldo Scarpa, Ghislaine Scelo, Dirk Schadendorf, Jacqueline E. Schein, Markus B. Schilhabel, Matthias Schlesner, Thorsten Schlomm, Heather K. Schmidt, Sarah-Jane Schramm, Stefan Schreiber, Nikolaus Schultz, Steven E. Schumacher, Roland F. Schwarz, Richard A. Scolyer, David Scott, Ralph Scully, Raja Seethala, Ayellet V. Segre, Iris Selander, Colin A. Semple, Yasin Senbabaoglu, Subhajit Sengupta, Elisabetta Sereni, Stefano Serra, Dennis C. Sgroi, Mark Shackleton, Nimish C. Shah, Sagedeh Shahabi, Catherine A. Shang, Ping Shang, Ofer Shapira, Troy Shelton, Ciyue Shen, Hui Shen, Rebecca Shepherd, Ruian Shi, Yan Shi, Yu-Jia Shiah, Tatsuhiro Shibata, Juliann Shih, Eigo Shimizu, Kiyo Shimizu, Seung Jun Shin, Yuichi Shiraishi, Tal Shmaya, Ilya Shmulevich, Solomon I. Shorser, Charles Short, Raunak Shrestha, Suyash S. Shringarpure, Craig Shriver, Shimin Shuai, Nikos Sidiropoulos, Reiner Siebert, Anieta M. Sieuwerts, Lina Sieverling, Sabina Signoretti, Katarzyna O. Sikora, Michele Simbolo, Ronald Simon, Janae V. Simons, Jared T. Simpson, Peter T. Simpson, Samuel Singer, Nasa Sinnott-Armstrong, Payal Sipahimalani, Tara J. Skelly, Marcel Smid, Jaclyn Smith, Karen Smith-McCune, Nicholas D. Socci, Heidi J. Sofia, Matthew G. Soloway, Lei Song, Anil K. Sood, Sharmila Sothi, Christos Sotiriou, Cameron M. Soulette, Paul N. Span, Paul T. Spellman, Nicola Sperandio, Andrew J. Spillane, Oliver Spiro, Jonathan Spring, Johan Staaf, Peter F. Stadler, Peter Staib, Stefan G. Stark, Lucy Stebbings, Ólafur Andri Stefánsson, Oliver Stegle, Lincoln D. Stein, Alasdair Stenhouse, Chip Stewart, Stephan Stilgenbauer, Miranda D. Stobbe, Michael R. Stratton, Jonathan R. Stretch, Adam J. Struck, Joshua M. Stuart, Henk G. Stunnenberg, Hong Su, Xiaoping Su, Ren X. Sun, Stephanie Sungalee, Hana Susak, Akihiro Suzuki, Fred Sweep, Monika Szczepanowski, Holger Sültmann, Takashi Yugawa, Angela Tam, David Tamborero, Benita Kiat Tee Tan, Donghui Tan, Patrick Tan, Hiroko Tanaka, Hirokazu Taniguchi, Tomas J. Tanskanen, Maxime Tarabichi, Roy Tarnuzzer, Patrick Tarpey, Morgan L. Taschuk, Kenji Tatsuno, Simon Tavaré, Darrin F. Taylor, Amaro Taylor-Weiner, Jon W. Teague, Bin Tean Teh, Varsha Tembe, Javier Temes, Kevin Thai, Sarah P. Thayer, Nina Thiessen, Gilles Thomas, Sarah Thomas, Alan Thompson, Alastair M. Thompson, John F. F. Thompson, R. Houston Thompson, Heather Thorne, Leigh B. Thorne, Adrian Thorogood, Grace Tiao, Nebojsa Tijanic, Lee E. Timms, Roberto Tirabosco, Marta Tojo, Stefania Tommasi, Christopher W. Toon, Umut H. Toprak, David Torrents, Giampaolo Tortora, Jörg Tost, Yasushi Totoki, David Townend, Nadia Traficante, Isabelle Treilleux, Jean-Rémi Trotta, Lorenz H. P. Trümper, Ming Tsao, Tatsuhiko Tsunoda, Jose M. C. Tubio, Olga Tucker, Richard Turkington, Daniel J. Turner, Andrew Tutt, Masaki Ueno, Naoto T. Ueno, Christopher Umbricht, Husen M. Umer, Timothy J. Underwood, Lara Urban, Tomoko Urushidate, Tetsuo Ushiku, Liis Uusküla-Reimand, Alfonso Valencia, David J. Van Den Berg, Steven Van Laere, Peter Van Loo, Erwin G. Van Meir, Gert G. Van den Eynden, Theodorus Van der Kwast, Naveen Vasudev, Miguel Vazquez, Ravikiran Vedururu, Umadevi Veluvolu, Shankar Vembu, Lieven P. C. Verbeke, Peter Vermeulen, Clare Verrill, Alain Viari, David Vicente, Caterina Vicentini, K. VijayRaghavan, Juris Viksna, Ricardo E. Vilain, Izar Villasante, Anne Vincent-Salomon, Tapio Visakorpi, Douglas Voet, Paresh Vyas, Ignacio Vázquez-García, Nick M. Waddell, Nicola Waddell, Claes Wadelius, Lina Wadi, Rabea Wagener, Jeremiah A. Wala, Jian Wang, Jiayin Wang, Linghua Wang, Qi Wang, Wenyi Wang, Yumeng Wang, Zhining Wang, Paul M. Waring, Hans-Jörg Warnatz, Jonathan Warrell, Anne Y. Warren, Sebastian M. Waszak, David C. Wedge, Dieter Weichenhan, Paul Weinberger, John N. Weinstein, Joachim Weischenfeldt, Daniel J. Weisenberger, Ian Welch, Michael C. Wendl, Johannes Werner, Justin P. Whalley, David A. Wheeler, Hayley C. Whitaker, Dennis Wigle, Matthew D. Wilkerson, Ashley Williams, James S. Wilmott, Gavin W. Wilson, Julie M. Wilson, Richard K. Wilson, Boris Winterhoff, Jeffrey A. Wintersinger, Maciej Wiznerowicz, Stephan Wolf, Bernice H. Wong, Tina Wong, Winghing Wong, Youngchoon Woo, Scott Wood, Bradly G. Wouters, Adam J. Wright, Derek W. Wright, Mark H. Wright, Chin-Lee Wu, Dai-Ying Wu, Guanming Wu, Jianmin Wu, Kui Wu, Yang Wu, Zhenggang Wu, Liu Xi, Tian Xia, Qian Xiang, Xiao Xiao, Rui Xing, Heng Xiong, Qinying Xu, Yanxun Xu, Hong Xue, Shinichi Yachida, Sergei Yakneen, Rui Yamaguchi, Takafumi N. Yamaguchi, Masakazu Yamamoto, Shogo Yamamoto, Hiroki Yamaue, Fan Yang, Huanming Yang, Jean Y. Yang, Liming Yang, Lixing Yang, Shanlin Yang, Tsun-Po Yang, Yang Yang, Xiaotong Yao, Marie-Laure Yaspo, Lucy Yates, Christina Yau, Chen Ye, Kai Ye, Venkata D. Yellapantula, Christopher J. Yoon, Sung-Soo Yoon, Fouad Yousif, Jun Yu, Kaixian Yu, Willie Yu, Yingyan Yu, Ke Yuan, Yuan Yuan, Denis Yuen, Christina K. Yung, Olga Zaikova, Jorge Zamora, Marc Zapatka, Jean C. Zenklusen, Thorsten Zenz, Nikolajs Zeps, Cheng-Zhong Zhang, Fan Zhang, Hailei Zhang, Hongwei Zhang, Hongxin Zhang, Jiashan Zhang, Jing Zhang, Junjun Zhang, Xiuqing Zhang, Xuanping Zhang, Yan Zhang, Zemin Zhang, Zhongming Zhao, Liangtao Zheng, Xiuqing Zheng, Wanding Zhou, Yong Zhou, Bin Zhu, Hongtu Zhu, Jingchun Zhu, Shida Zhu, Lihua Zou, Xueqing Zou, Anna deFazio, Nicholas van As, Carolien H. M. van Deurzen, Marc J. van de Vijver, L. van’t Veer, Christian von Mering

**Affiliations:** 1 Applied Tumor Genomics Research Program, Research Programs Unit, University of Helsinki, Helsinki, Finland; 2 Wellcome Sanger Institute, Wellcome Genome Campus, Hinxton, UK; 3 Memorial Sloan Kettering Cancer Center, New York, NY USA; 4 Genome Science Division, Research Center for Advanced Science and Technology, University of Tokyo, Tokyo, Japan; 5 Department of Surgery, University of Chicago, Chicago, IL USA; 6 Department of Surgery, Division of Hepatobiliary and Pancreatic Surgery, School of Medicine, Keimyung University Dongsan Medical Center, Daegu, South Korea; 7 Department of Oncology, Gil Medical Center, Gachon University, Incheon, South Korea; 8 Hiroshima University, Hiroshima, Japan; 9 Department of Bioinformatics and Computational Biology, The University of Texas MD Anderson Cancer Center, Houston, TX USA; 10 University of Texas MD Anderson Cancer Center, Houston, TX USA; 11 King Faisal Specialist Hospital and Research Centre, Al Maather, Riyadh, Saudi Arabia; 12 Bioinformatics Unit, Spanish National Cancer Research Centre (CNIO), Madrid, Spain; 13 Bioinformatics Core Facility, University Medical Center Hamburg, Hamburg, Germany; 14 Heinrich Pette Institute, Leibniz Institute for Experimental Virology, Hamburg, Germany; 15 Ontario Tumour Bank, Ontario Institute for Cancer Research, Toronto, ON Canada; 16 Department of Pathology, The University of Texas MD Anderson Cancer Center, Houston, TX USA; 17 Laboratory of Pathology, Center for Cancer Research, National Cancer Institute, Bethesda, MD USA; 18 Department of Cellular and Molecular Medicine and Department of Bioengineering, University of California San Diego, La Jolla, CA USA; 19 UC San Diego Moores Cancer Center, San Diego, CA USA; 20 Canada’s Michael Smith Genome Sciences Centre, BC Cancer, Vancouver, BC Canada; 21 Sir Peter MacCallum Department of Oncology, Peter MacCallum Cancer Centre, University of Melbourne, Melbourne, VIC Australia; 22 Centre for Research in Molecular Medicine and Chronic Diseases (CiMUS), Universidade de Santiago de Compostela, Santiago de Compostela, Spain; 23 Department of Zoology, Genetics and Physical Anthropology, (CiMUS), Universidade de Santiago de Compostela, Santiago de Compostela, Spain; 24 The Biomedical Research Centre (CINBIO), Universidade de Vigo, Vigo, Spain; 25 Royal National Orthopaedic Hospital - Bolsover, London, UK; 26 Department of Genomic Medicine, The University of Texas MD Anderson Cancer Center, Houston, TX USA; 27 Quantitative and Computational Biosciences Graduate Program, Baylor College of Medicine, Houston, TX USA; 28 The Jackson Laboratory for Genomic Medicine, Farmington, CT USA; 29 Genome Informatics Program, Ontario Institute for Cancer Research, Toronto, ON Canada; 30 Institute of Human Genetics, Christian-Albrechts-University, Kiel, Germany; 31 Institute of Human Genetics, Ulm University and Ulm University Medical Center, Ulm, Germany; 32 Queensland Centre for Medical Genomics, Institute for Molecular Bioscience, University of Queensland, St. Lucia, Brisbane, QLD Australia; 33 Salford Royal NHS Foundation Trust, Salford, UK; 34 Department of Surgery, Pancreas Institute, University and Hospital Trust of Verona, Verona, Italy; 35 Molecular and Medical Genetics, OHSU Knight Cancer Institute, Oregon Health and Science University, Portland, OR USA; 36 Department of Molecular Oncology, BC Cancer Research Centre, Vancouver, BC Canada; 37 The McDonnell Genome Institute at Washington University, St. Louis, MO USA; 38 University College London, London, UK; 39 Division of Cancer Genomics, National Cancer Center Research Institute, National Cancer Center, Tokyo, Japan; 40 DLR Project Management Agency, Bonn, Germany; 41 Tokyo Women’s Medical University, Tokyo, Japan; 42 Center for Molecular Oncology, Memorial Sloan Kettering Cancer Center, New York, NY USA; 43 Los Alamos National Laboratory, Los Alamos, NM USA; 44 Department of Pathology, University Health Network, Toronto General Hospital, Toronto, ON Canada; 45 Nottingham University Hospitals NHS Trust, Nottingham, UK; 46 Epigenomics and Cancer Risk Factors, German Cancer Research Center (DKFZ), Heidelberg, Germany; 47 Computational Biology Program, Ontario Institute for Cancer Research, Toronto, ON Canada; 48 Department of Molecular Genetics, University of Toronto, Toronto, ON Canada; 49 Vector Institute, Toronto, ON Canada; 50 Hematopathology Section, Institute of Pathology, Christian-Albrechts-University, Kiel, Germany; 51 Department of Pathology and Laboratory Medicine, School of Medicine, University of North Carolina at Chapel Hill, Chapel Hill, NC USA; 52 Department of Cancer Genetics, Institute for Cancer Research, Oslo University Hospital, The Norwegian Radium Hospital, Oslo, Norway; 53 Pathology, Hospital Clinic, Institut d’Investigacions Biomèdiques August Pi i Sunyer (IDIBAPS), University of Barcelona, Barcelona, Spain; 54 Department of Veterinary Medicine, Transmissible Cancer Group, University of Cambridge, Cambridge, UK; 55 Alvin J. Siteman Cancer Center, Washington University School of Medicine, St. Louis, MO USA; 56 Wolfson Wohl Cancer Research Centre, Institute of Cancer Sciences, University of Glasgow, Glasgow, UK; 57 Lineberger Comprehensive Cancer Center, University of North Carolina at Chapel Hill, Chapel Hill, NC USA; 58 Broad Institute of MIT and Harvard, Cambridge, MA USA; 59 Dana-Farber/Boston Children’s Cancer and Blood Disorders Center, Boston, MA USA; 60 Department of Pediatrics, Harvard Medical School, Boston, MA USA; 61 Leeds Institute of Medical Research @ St. James’s, University of Leeds, St. James’s University Hospital, Leeds, UK; 62 Department of Pathology and Diagnostics, University and Hospital Trust of Verona, Verona, Italy; 63 Department of Surgery, Princess Alexandra Hospital, Brisbane, QLD Australia; 64 Surgical Oncology Group, Diamantina Institute, University of Queensland, Brisbane, QLD Australia; 65 Department of Population and Quantitative Health Sciences, Case Western Reserve University School of Medicine, Cleveland, OH USA; 66 Research Health Analytics and Informatics, University Hospitals Cleveland Medical Center, Cleveland, OH USA; 67 Gloucester Royal Hospital, Gloucester, UK; 68 European Molecular Biology Laboratory, European Bioinformatics Institute (EMBL-EBI), Cambridge, UK; 69 Diagnostic Development, Ontario Institute for Cancer Research, Toronto, ON Canada; 70 Barcelona Supercomputing Center (BSC), Barcelona, Spain; 71 Arnie Charbonneau Cancer Institute, University of Calgary, Calgary, AB Canada; 72 Departments of Surgery and Oncology, University of Calgary, Calgary, AB Canada; 73 Department of Pathology, Oslo University Hospital, The Norwegian Radium Hospital, Oslo, Norway; 74 PanCuRx Translational Research Initiative, Ontario Institute for Cancer Research, Toronto, ON Canada; 75 Department of Oncology, Sidney Kimmel Comprehensive Cancer Center at Johns Hopkins University School of Medicine, Baltimore, MD USA; 76 University Hospital Southampton NHS Foundation Trust, Southampton, UK; 77 Royal Stoke University Hospital, Stoke-on-Trent, UK; 78 Genome Sequence Informatics, Ontario Institute for Cancer Research, Toronto, ON Canada; 79 Human Longevity Inc, San Diego, CA USA; 80 Olivia Newton-John Cancer Research Institute, La Trobe University, Heidelberg, VIC Australia; 81 Computer Network Information Center, Chinese Academy of Sciences, Beijing, China; 82 Genome Canada, Ottawa, ON Canada; 83 CNAG-CRG, Centre for Genomic Regulation (CRG), Barcelona Institute of Science and Technology (BIST), Barcelona, Spain; 84 Universitat Pompeu Fabra (UPF), Barcelona, Spain; 85 Buck Institute for Research on Aging, Novato, CA USA; 86 Duke University Medical Center, Durham, NC USA; 87 Department of Human Genetics, Hannover Medical School, Hannover, Germany; 88 Center for Bioinformatics and Functional Genomics, Cedars-Sinai Medical Center, Los Angeles, CA USA; 89 Department of Biomedical Sciences, Cedars-Sinai Medical Center, Los Angeles, CA USA; 90 The Hebrew University Faculty of Medicine, Jerusalem, Israel; 91 Barts Cancer Institute, Barts and the London School of Medicine and Dentistry, Queen Mary University of London, London, UK; 92 Department of Computer Science, Bioinformatics Group, University of Leipzig, Leipzig, Germany; 93 Interdisciplinary Center for Bioinformatics, University of Leipzig, Leipzig, Germany; 94 Transcriptome Bioinformatics, LIFE Research Center for Civilization Diseases, University of Leipzig, Leipzig, Germany; 95 Department of Medical Oncology, Dana-Farber Cancer Institute, Boston, MA USA; 96 Department of Cancer Biology, Dana-Farber Cancer Institute, Boston, MA USA; 97 Harvard Medical School, Boston, MA USA; 98 USC Norris Comprehensive Cancer Center, University of Southern California, Los Angeles, CA USA; 99 Department of Diagnostics and Public Health, University and Hospital Trust of Verona, Verona, Italy; 100 Department of Mathematics, Aarhus University, Aarhus, Denmark; 101 Department of Molecular Medicine (MOMA), Aarhus University Hospital, Aarhus N, Denmark; 102 Instituto Carlos Slim de la Salud, Mexico City, Mexico; 103 Department of Medical Biophysics, University of Toronto, Toronto, ON Canada; 104 Cancer Division, Garvan Institute of Medical Research, Kinghorn Cancer Centre, University of New South Wales (UNSW Sydney), Sydney, NSW Australia; 105 South Western Sydney Clinical School, Faculty of Medicine, University of New South Wales (UNSW Sydney), Liverpool, NSW Australia; 106 West of Scotland Pancreatic Unit, Glasgow Royal Infirmary, Glasgow, UK; 107 Center for Digital Health, Berlin Institute of Health and Charitè - Universitätsmedizin Berlin, Berlin, Germany; 108 Heidelberg Center for Personalized Oncology (DKFZ-HIPO), German Cancer Research Center (DKFZ), Heidelberg, Germany; 109 The Preston Robert Tisch Brain Tumor Center, Duke University Medical Center, Durham, NC USA; 110 Massachusetts General Hospital, Boston, MA USA; 111 National Institute of Biomedical Genomics, Kalyani, West Bengal India; 112 Institute of Clinical Medicine and Institute of Oral Biology, University of Oslo, Oslo, Norway; 113 University of North Carolina at Chapel Hill, Chapel Hill, NC USA; 114 ARC-Net Centre for Applied Research on Cancer, University and Hospital Trust of Verona, Verona, Italy; 115 The Institute of Cancer Research, London, UK; 116 Centre for Computational Biology, Duke-NUS Medical School, Singapore, Singapore; 117 Programme in Cancer and Stem Cell Biology, Duke-NUS Medical School, Singapore, Singapore; 118 Division of Oncology and Pathology, Department of Clinical Sciences Lund, Lund University, Lund, Sweden; 119 Department of Pediatric Oncology, Hematology and Clinical Immunology, Heinrich-Heine-University, Düsseldorf, Germany; 120 Laboratory for Medical Science Mathematics, RIKEN Center for Integrative Medical Sciences, Yokohama, Japan; 121 RIKEN Center for Integrative Medical Sciences, Yokohama, Japan; 122 Department of Internal Medicine/Hematology, Friedrich-Ebert-Hospital, Neumünster, Germany; 123 Departments of Dermatology and Pathology, Yale University, New Haven, CT USA; 124 Centre for Genomic Regulation (CRG), The Barcelona Institute of Science and Technology, Barcelona, Spain; 125 Radcliffe Department of Medicine, University of Oxford, Oxford, UK; 126 Canadian Center for Computational Genomics, McGill University, Montreal, QC Canada; 127 Department of Human Genetics, McGill University, Montreal, QC Canada; 128 Department of Human Genetics, University of California Los Angeles, Los Angeles, CA USA; 129 Department of Pharmacology, University of Toronto, Toronto, ON Canada; 130 Faculty of Medicine and Health Technology, Tampere University and Tays Cancer Center, Tampere University Hospital, Tampere, Finland; 131 Haematology, Leeds Teaching Hospitals NHS Trust, Leeds, UK; 132 Translational Research and Innovation, Centre Léon Bérard, Lyon, France; 133 Fox Chase Cancer Center, Philadelphia, PA USA; 134 International Agency for Research on Cancer, World Health Organization, Lyon, France; 135 Earlham Institute, Norwich, UK; 136 Norwich Medical School, University of East Anglia, Norwich, UK; 137 Department of Molecular Biology, Faculty of Science, Radboud Institute for Molecular Life Sciences, Radboud University, Nijmegen, HB The Netherlands; 138 CRUK Manchester Institute and Centre, Manchester, UK; 139 Department of Radiation Oncology, University of Toronto, Toronto, ON Canada; 140 Division of Cancer Sciences, Manchester Cancer Research Centre, University of Manchester, Manchester, UK; 141 Radiation Medicine Program, Princess Margaret Cancer Centre, Toronto, ON Canada; 142 Department of Pathology, Brigham and Women’s Hospital, Harvard Medical School, Boston, MA USA; 143 Department of Surgery, Division of Thoracic Surgery, The Johns Hopkins University School of Medicine, Baltimore, MD USA; 144 Division of Molecular Pathology, The Netherlands Cancer Institute, Oncode Institute, Amsterdam, CX The Netherlands; 145 Department of Biomolecular Engineering, University of California Santa Cruz, Santa Cruz, CA USA; 146 UC Santa Cruz Genomics Institute, University of California Santa Cruz, Santa Cruz, CA USA; 147 Division of Applied Bioinformatics, German Cancer Research Center (DKFZ), Heidelberg, Germany; 148 German Cancer Consortium (DKTK), German Cancer Research Center (DKFZ), Heidelberg, Germany; 149 National Center for Tumor Diseases (NCT) Heidelberg, Heidelberg, Germany; 150 Center for Biological Sequence Analysis, Department of Bio and Health Informatics, Technical University of Denmark, Lyngby, Denmark; 151 Novo Nordisk Foundation Center for Protein Research, University of Copenhagen, Copenhagen, Denmark; 152 Institute for Molecular Bioscience, University of Queensland, St. Lucia, Brisbane, QLD Australia; 153 Biomedical Engineering, Oregon Health and Science University, Portland, OR USA; 154 Division of Theoretical Bioinformatics, German Cancer Research Center (DKFZ), Heidelberg, Germany; 155 Institute of Pharmacy and Molecular Biotechnology and BioQuant, Heidelberg University, Heidelberg, Germany; 156 Federal Ministry of Education and Research, Berlin, Germany; 157 Melanoma Institute Australia, University of Sydney, Sydney, NSW Australia; 158 Pediatric Hematology and Oncology, University Hospital Muenster, Muenster, Germany; 159 Department of Pathology, Johns Hopkins University School of Medicine, Baltimore, MD USA; 160 McKusick-Nathans Institute of Genetic Medicine, Sidney Kimmel Comprehensive Cancer Center at Johns Hopkins University School of Medicine, Baltimore, MD USA; 161 Foundation Medicine, Inc, Cambridge, MA USA; 162 Department of Biomedical Data Science, Stanford University School of Medicine, Stanford, CA USA; 163 Department of Genetics, Stanford University School of Medicine, Stanford, CA USA; 164 Bakar Computational Health Sciences Institute and Department of Pediatrics, University of California, San Francisco, CA USA; 165 Institute of Clinical Medicine, Faculty of Medicine, University of Oslo, Oslo, Norway; 166 National Cancer Institute, National Institutes of Health, Bethesda, MD USA; 167 Royal Marsden NHS Foundation Trust, London and Sutton, UK; 168 Genome Biology Unit, European Molecular Biology Laboratory (EMBL), Heidelberg, Germany; 169 Department of Oncology, University of Cambridge, Cambridge, UK; 170 Li Ka Shing Centre, Cancer Research UK Cambridge Institute, University of Cambridge, Cambridge, UK; 171 Institut Gustave Roussy, Villejuif, France; 172 Cambridge University Hospitals NHS Foundation Trust, Cambridge, UK; 173 Department of Haematology, University of Cambridge, Cambridge, UK; 174 Anatomia Patológica, Hospital Clinic, Institut d’Investigacions Biomèdiques August Pi i Sunyer (IDIBAPS), University of Barcelona, Barcelona, Spain; 175 Spanish Ministry of Science and Innovation, Madrid, Spain; 176 University of Michigan Comprehensive Cancer Center, Ann Arbor, MI USA; 177 Department for BioMedical Research, University of Bern, Bern, Switzerland; 178 Department of Medical Oncology, Inselspital, University Hospital and University of Bern, Bern, Switzerland; 179 Graduate School for Cellular and Biomedical Sciences, University of Bern, Bern, Switzerland; 180 University of Pavia, Pavia, Italy; 181 University of Alabama at Birmingham, Birmingham, AL USA; 182 UHN Program in BioSpecimen Sciences, Toronto General Hospital, Toronto, ON Canada; 183 Department of Urology, Icahn School of Medicine at Mount Sinai, New York, NY USA; 184 Centre for Law and Genetics, University of Tasmania, Sandy Bay Campus, Hobart, TAS Australia; 185 Faculty of Biosciences, Heidelberg University, Heidelberg, Germany; 186 Department of Biochemistry, Microbiology and Immunology, Faculty of Medicine, University of Ottawa, Ottawa, ON Canada; 187 Division of Anatomic Pathology, Mayo Clinic, Rochester, MN USA; 188 Division of Cancer Epidemiology and Genetics, National Cancer Institute, National Institutes of Health, Bethesda, MD USA; 189 Illawarra Shoalhaven Local Health District L3 Illawarra Cancer Care Centre, Wollongong Hospital, Wollongong, NSW Australia; 190 BioForA, French National Institute for Agriculture, Food, and Environment (INRAE), ONF, Orléans, France; 191 Department of Biostatistics, Bloomberg School of Public Health, Johns Hopkins University, Baltimore, MD USA; 192 University of California San Diego, San Diego, CA USA; 193 Division of Experimental Pathology, Mayo Clinic, Rochester, MN USA; 194 Centre for Cancer Research, The Westmead Institute for Medical Research, University of Sydney, Sydney, NSW Australia; 195 Department of Gynaecological Oncology, Westmead Hospital, Sydney, NSW Australia; 196 PDXen Biosystems Inc, Seoul, South Korea; 197 Korea Advanced Institute of Science and Technology, Daejeon, South Korea; 198 Electronics and Telecommunications Research Institute, Daejeon, South Korea; 199 Institut National du Cancer (INCA), Boulogne-Billancourt, France; 200 Department of Genetics, Informatics Institute, University of Alabama at Birmingham, Birmingham, AL USA; 201 Division of Medical Oncology, National Cancer Centre, Singapore, Singapore; 202 Medical Oncology, University and Hospital Trust of Verona, Verona, Italy; 203 Department of Pediatrics, University Hospital Schleswig-Holstein, Kiel, Germany; 204 Hepatobiliary/Pancreatic Surgical Oncology Program, University Health Network, Toronto, ON Canada; 205 School of Biological Sciences, University of Auckland, Auckland, New Zealand; 206 Department of Surgery, University of Melbourne, Parkville, VIC Australia; 207 The Murdoch Children’s Research Institute, Royal Children’s Hospital, Parkville, VIC Australia; 208 Walter and Eliza Hall Institute, Parkville, VIC Australia; 209 Vancouver Prostate Centre, Vancouver, Canada; 210 Lunenfeld-Tanenbaum Research Institute, Mount Sinai Hospital, Toronto, ON Canada; 211 University of East Anglia, Norwich, UK; 212 Norfolk and Norwich University Hospital NHS Trust, Norwich, UK; 213 Victorian Institute of Forensic Medicine, Southbank, VIC Australia; 214 Department of Biomedical Informatics, Harvard Medical School, Boston, MA USA; 215 Department of Chemistry, Centre for Molecular Science Informatics, University of Cambridge, Cambridge, UK; 216 Ludwig Center at Harvard Medical School, Boston, MA USA; 217 Human Genome Sequencing Center, Baylor College of Medicine, Houston, TX USA; 218 Peter MacCallum Cancer Centre, University of Melbourne, Melbourne, VIC Australia; 219 Physics Division, Optimization and Systems Biology Lab, Massachusetts General Hospital, Boston, MA USA; 220 Department of Medicine, Baylor College of Medicine, Houston, TX USA; 221 University of Cologne, Cologne, Germany; 222 International Genomics Consortium, Phoenix, AZ USA; 223 Genomics Research Program, Ontario Institute for Cancer Research, Toronto, ON Canada; 224 Barking Havering and Redbridge University Hospitals NHS Trust, Romford, UK; 225 Children’s Hospital at Westmead, University of Sydney, Sydney, NSW Australia; 226 Department of Medicine, Section of Endocrinology, University and Hospital Trust of Verona, Verona, Italy; 227 Computational Biology Center, Memorial Sloan Kettering Cancer Center, New York, NY USA; 228 Department of Biology, ETH Zurich, Zürich, Switzerland; 229 Department of Computer Science, ETH Zurich, Zurich, Switzerland; 230 SIB Swiss Institute of Bioinformatics, Lausanne, Switzerland; 231 Weill Cornell Medical College, New York, NY USA; 232 Academic Department of Medical Genetics, University of Cambridge, Addenbrooke’s Hospital, Cambridge, UK; 233 MRC Cancer Unit, University of Cambridge, Cambridge, UK; 234 Departments of Pediatrics and Genetics, University of North Carolina at Chapel Hill, Chapel Hill, NC USA; 235 Seven Bridges Genomics, Charlestown, MA USA; 236 Annai Systems, Inc, Carlsbad, CA USA; 237 Department of Pathology, General Hospital of Treviso, Department of Medicine, University of Padua, Treviso, Italy; 238 Department of Computational Biology, University of Lausanne, Lausanne, Switzerland; 239 Department of Genetic Medicine and Development, University of Geneva Medical School, Geneva, CH Switzerland; 240 Swiss Institute of Bioinformatics, University of Geneva, Geneva, CH Switzerland; 241 The Francis Crick Institute, London, UK; 242 University of Leuven, Leuven, Belgium; 243 Institute of Medical Genetics and Applied Genomics, University of Tübingen, Tübingen, Germany; 244 Computational and Systems Biology, Genome Institute of Singapore, Singapore, Singapore; 245 School of Computing, National University of Singapore, Singapore, Singapore; 246 Big Data Institute, Li Ka Shing Centre, University of Oxford, Oxford, UK; 247 Biomedical Data Science Laboratory, Francis Crick Institute, London, UK; 248 Bioinformatics Group, Department of Computer Science, University College London, London, UK; 249 The Edward S. Rogers Sr. Department of Electrical and Computer Engineering, University of Toronto, Toronto, ON Canada; 250 Breast Cancer Translational Research Laboratory JC Heuson, Institut Jules Bordet, Brussels, Belgium; 251 Department of Oncology, Laboratory for Translational Breast Cancer Research, KU Leuven, Leuven, Belgium; 252 Institute for Research in Biomedicine (IRB Barcelona), The Barcelona Institute of Science and Technology, Barcelona, Spain; 253 Research Program on Biomedical Informatics, Universitat Pompeu Fabra, Barcelona, Spain; 254 Division of Medical Oncology, Princess Margaret Cancer Centre, Toronto, ON Canada; 255 Department of Physiology and Biophysics, Weill Cornell Medicine, New York, NY USA; 256 Institute for Computational Biomedicine, Weill Cornell Medicine, New York, NY USA; 257 Department of Pathology, UPMC Shadyside, Pittsburgh, PA USA; 258 Independent Consultant, Wellesley, USA; 259 Department of Cell and Molecular Biology, Science for Life Laboratory, Uppsala University, Uppsala, Sweden; 260 Department of Medicine and Department of Genetics, Washington University School of Medicine, St. Louis, St. Louis, MO USA; 261 Hefei University of Technology, Anhui, China; 262 Translational Cancer Research Unit, GZA Hospitals St.-Augustinus, Center for Oncological Research, Faculty of Medicine and Health Sciences, University of Antwerp, Antwerp, Belgium; 263 Simon Fraser University, Burnaby, BC Canada; 264 University of Pennsylvania, Philadelphia, PA USA; 265 Faculty of Science and Technology, University of Vic—Central University of Catalonia (UVic-UCC), Vic, Spain; 266 The Wellcome Trust, London, UK; 267 The Hospital for Sick Children, Toronto, ON Canada; 268 Department of Pathology, Queen Elizabeth University Hospital, Glasgow, UK; 269 Department of Genetics and Computational Biology, QIMR Berghofer Medical Research Institute, Brisbane, QLD Australia; 270 Department of Oncology, Centre for Cancer Genetic Epidemiology, University of Cambridge, Cambridge, UK; 271 Department of Public Health and Primary Care, Centre for Cancer Genetic Epidemiology, University of Cambridge, Cambridge, UK; 272 Prostate Cancer Canada, Toronto, ON Canada; 273 University of Cambridge, Cambridge, UK; 274 Department of Laboratory Medicine, Translational Cancer Research, Lund University Cancer Center at Medicon Village, Lund University, Lund, Sweden; 275 Heidelberg University, Heidelberg, Germany; 276 New BIH Digital Health Center, Berlin Institute of Health (BIH) and Charité - Universitätsmedizin Berlin, Berlin, Germany; 277 CIBER Epidemiología y Salud Pública (CIBERESP), Madrid, Spain; 278 Research Group on Statistics, Econometrics and Health (GRECS), UdG, Barcelona, Spain; 279 Quantitative Genomics Laboratories (qGenomics), Barcelona, Spain; 280 Icelandic Cancer Registry, Icelandic Cancer Society, Reykjavik, Iceland; 281 State Key Laboratory of Cancer Biology, and Xijing Hospital of Digestive Diseases, Fourth Military Medical University, Shaanxi, China; 282 Department of Medicine (DIMED), Surgical Pathology Unit, University of Padua, Padua, Italy; 283 Rigshospitalet, Copenhagen, Denmark; 284 Center for Cancer Genomics, National Cancer Institute, National Institutes of Health, Bethesda, MD USA; 285 Department of Biochemistry and Molecular Medicine, University of Montreal, Montreal, QC Canada; 286 Australian Institute of Tropical Health and Medicine, James Cook University, Douglas, QLD Australia; 287 Department of Neuro-Oncology, Istituto Neurologico Besta, Milano, Italy; 288 Bioplatforms Australia, North Ryde, NSW Australia; 289 Department of Pathology (Research), University College London Cancer Institute, London, UK; 290 Department of Surgical Oncology, Princess Margaret Cancer Centre, Toronto, ON Canada; 291 Department of Medical Oncology, Josephine Nefkens Institute and Cancer Genomics Centre, Erasmus Medical Center, Rotterdam, CN The Netherlands; 292 The University of Queensland Thoracic Research Centre, The Prince Charles Hospital, Brisbane, QLD Australia; 293 CIBIO/InBIO - Research Center in Biodiversity and Genetic Resources, Universidade do Porto, Vairão, Portugal; 294 HCA Laboratories, London, UK; 295 University of Liverpool, Liverpool, UK; 296 The Azrieli Faculty of Medicine, Bar-Ilan University, Safed, Israel; 297 Department of Neurosurgery, University of Florida, Gainesville, FL USA; 298 Department of Pathology, Graduate School of Medicine, University of Tokyo, Tokyo, Japan; 299 University of Milano Bicocca, Monza, Italy; 300 BGI-Shenzhen, Shenzhen, China; 301 Department of Pathology, Oslo University Hospital Ulleval, Oslo, Norway; 302 Center for Biomedical Informatics, Harvard Medical School, Boston, MA USA; 303 Department Biochemistry and Molecular Biomedicine, University of Barcelona, Barcelona, Spain; 304 Office of Cancer Genomics, National Cancer Institute, National Institutes of Health, Bethesda, MD USA; 305 Cancer Epigenomics, German Cancer Research Center (DKFZ), Heidelberg, Germany; 306 Department of Cancer Biology, The University of Texas MD Anderson Cancer Center, Houston, TX USA; 307 Department of Surgical Oncology, The University of Texas MD Anderson Cancer Center, Houston, TX USA; 308 Department of Computer Science, Yale University, New Haven, CT USA; 309 Department of Molecular Biophysics and Biochemistry, Yale University, New Haven, CT USA; 310 Program in Computational Biology and Bioinformatics, Yale University, New Haven, CT USA; 311 Center for Cancer Research, Massachusetts General Hospital, Boston, MA USA; 312 Department of Pathology, Massachusetts General Hospital, Boston, MA USA; 313 Department of Pathology, Memorial Sloan Kettering Cancer Center, New York, NY USA; 314 Division of Gastroenterology and Hepatology, Mayo Clinic, Rochester, MN USA; 315 University of Sydney, Sydney, NSW Australia; 316 University of Oxford, Oxford, UK; 317 Department of Surgery, Academic Urology Group, University of Cambridge, Cambridge, UK; 318 Department of Medicine II, University of Würzburg, Wuerzburg, Germany; 319 Sylvester Comprehensive Cancer Center, University of Miami, Miami, FL USA; 320 Institut Hospital del Mar d’Investigacions Mèdiques (IMIM), Barcelona, Spain; 321 Genome Integrity and Structural Biology Laboratory, National Institute of Environmental Health Sciences (NIEHS), Durham, NC USA; 322 St. Thomas’s Hospital, London, UK; 323 Osaka International Cancer Center, Osaka, Japan; 324 Department of Pathology, Skåne University Hospital, Lund University, Lund, Sweden; 325 Department of Medical Oncology, Beatson West of Scotland Cancer Centre, Glasgow, UK; 326 National Human Genome Research Institute, National Institutes of Health, Bethesda, MD USA; 327 Centre for Cancer Research, Victorian Comprehensive Cancer Centre, University of Melbourne, Melbourne, VIC Australia; 328 Department of Medicine, Section of Hematology/Oncology, University of Chicago, Chicago, IL USA; 329 German Center for Infection Research (DZIF), Partner Site Hamburg-Borstel-Lübeck-Riems, Hamburg, Germany; 330 Bioinformatics Research Centre (BiRC), Aarhus University, Aarhus, Denmark; 331 Department of Biotechnology, Ministry of Science and Technology, Government of India, New Delhi, Delhi India; 332 National Cancer Centre Singapore, Singapore, Singapore; 333 Brandeis University, Waltham, MA USA; 334 Department of Urologic Sciences, University of British Columbia, Vancouver, BC Canada; 335 Department of Internal Medicine, Stanford University, Stanford, CA USA; 336 The University of Texas Health Science Center at Houston, Houston, TX USA; 337 Imperial College NHS Trust, Imperial College, London, INY UK; 338 Senckenberg Institute of Pathology, University of Frankfurt Medical School, Frankfurt, Germany; 339 Department of Medicine, Division of Biomedical Informatics, UC San Diego School of Medicine, San Diego, CA USA; 340 Center for Precision Health, School of Biomedical Informatics, The University of Texas Health Science Center, Houston, TX USA; 341 Oxford Nanopore Technologies, New York, NY USA; 342 Institute of Medical Science, University of Tokyo, Tokyo, Japan; 343 Howard Hughes Medical Institute, University of California Santa Cruz, Santa Cruz, CA USA; 344 Wakayama Medical University, Wakayama, Japan; 345 Department of Internal Medicine, Division of Medical Oncology, Lineberger Comprehensive Cancer Center, University of North Carolina at Chapel Hill, Chapel Hill, NC USA; 346 University of Tennessee Health Science Center for Cancer Research, Memphis, TN USA; 347 Department of Histopathology, Salford Royal NHS Foundation Trust, Salford, UK; 348 Faculty of Biology, Medicine and Health, University of Manchester, Manchester, UK; 349 BIOPIC, ICG and College of Life Sciences, Peking University, Beijing, China; 350 Peking-Tsinghua Center for Life Sciences, Peking University, Beijing, China; 351 Children’s Hospital of Philadelphia, Philadelphia, PA USA; 352 Department of Bioinformatics and Computational Biology and Department of Systems Biology, The University of Texas MD Anderson Cancer Center, Houston, TX USA; 353 Karolinska Institute, Stockholm, Sweden; 354 The Donnelly Centre, University of Toronto, Toronto, ON Canada; 355 Department of Medical Genetics, College of Medicine, Hallym University, Chuncheon, South Korea; 356 Department of Experimental and Health Sciences, Institute of Evolutionary Biology (UPF-CSIC), Universitat Pompeu Fabra, Barcelona, Spain; 357 Health Data Science Unit, University Clinics, Heidelberg, Germany; 358 Massachusetts General Hospital Center for Cancer Research, Charlestown, MA USA; 359 Hokkaido University, Sapporo, Japan; 360 Department of Pathology and Clinical Laboratory, National Cancer Center Hospital, Tokyo, Japan; 361 Department of Genetics, University of North Carolina at Chapel Hill, Chapel Hill, NC USA; 362 Computational Biology, Leibniz Institute on Aging - Fritz Lipmann Institute (FLI), Jena, Germany; 363 University of Melbourne Centre for Cancer Research, Melbourne, VIC Australia; 364 University of Nebraska Medical Center, Omaha, NE USA; 365 Syntekabio Inc, Daejeon, South Korea; 366 Department of Pathology, Academic Medical Center, Amsterdam, AZ The Netherlands; 367 China National GeneBank-Shenzhen, Shenzhen, China; 368 Division of Molecular Genetics, German Cancer Research Center (DKFZ), Heidelberg, Germany; 369 Division of Life Science and Applied Genomics Center, Hong Kong University of Science and Technology, Clear Water Bay, Hong Kong, China; 370 Icahn School of Medicine at Mount Sinai, New York, NY USA; 371 Geneplus-Shenzhen, Shenzhen, China; 372 School of Computer Science and Technology, Xi’an Jiaotong University, Xi’an, China; 373 AbbVie, North Chicago, IL USA; 374 Institute of Pathology, Charité – University Medicine Berlin, Berlin, Germany; 375 Centre for Translational and Applied Genomics, British Columbia Cancer Agency, Vancouver, BC Canada; 376 Edinburgh Royal Infirmary, Edinburgh, UK; 377 Berlin Institute for Medical Systems Biology, Max Delbrück Center for Molecular Medicine, Berlin, Germany; 378 Department of Pediatric Immunology, Hematology and Oncology, University Hospital, Heidelberg, Germany; 379 German Cancer Research Center (DKFZ), Heidelberg, Germany; 380 Heidelberg Institute for Stem Cell Technology and Experimental Medicine (HI-STEM), Heidelberg, Germany; 381 Institute for Computational Biomedicine, Weill Cornell Medical College, New York, NY USA; 382 New York Genome Center, New York, NY USA; 383 Department of Urology, James Buchanan Brady Urological Institute, Johns Hopkins University School of Medicine, Baltimore, MD USA; 384 Department of Preventive Medicine, Graduate School of Medicine, The University of Tokyo, Tokyo, Japan; 385 Department of Molecular and Cellular Biology, Baylor College of Medicine, Houston, TX USA; 386 Department of Pathology and Immunology, Baylor College of Medicine, Houston, TX USA; 387 Michael E. DeBakey Veterans Affairs Medical Center, Houston, TX USA; 388 Technical University of Denmark, Lyngby, Denmark; 389 Department of Pathology, College of Medicine, Hanyang University, Seoul, South Korea; 390 Academic Unit of Surgery, School of Medicine, College of Medical, Veterinary and Life Sciences, University of Glasgow, Glasgow Royal Infirmary, Glasgow, UK; 391 Department of Pathology, Asan Medical Center, College of Medicine, Ulsan University, Songpa-gu, Seoul South Korea; 392 Science Writer, Garrett Park, MD USA; 393 International Cancer Genome Consortium (ICGC)/ICGC Accelerating Research in Genomic Oncology (ARGO) Secretariat, Ontario Institute for Cancer Research, Toronto, ON Canada; 394 University of Ljubljana, Ljubljana, Slovenia; 395 Department of Public Health Sciences, University of Chicago, Chicago, IL USA; 396 Research Institute, NorthShore University HealthSystem, Evanston, IL USA; 397 Department for Biomedical Research, University of Bern, Bern, Switzerland; 398 Centre of Genomics and Policy, McGill University and Génome Québec Innovation Centre, Montreal, QC Canada; 399 Carolina Center for Genome Sciences, University of North Carolina at Chapel Hill, Chapel Hill, NC USA; 400 Hopp Children’s Cancer Center (KiTZ), Heidelberg, Germany; 401 Pediatric Glioma Research Group, German Cancer Research Center (DKFZ), Heidelberg, Germany; 402 Cancer Research UK, London, UK; 403 Indivumed GmbH, Hamburg, Germany; 404 Genome Integration Data Center, Syntekabio, Inc, Daejeon, South Korea; 405 University Hospital Zurich, Zurich, Switzerland; 406 Clinical Bioinformatics, Swiss Institute of Bioinformatics, Geneva, Switzerland; 407 Institute for Pathology and Molecular Pathology, University Hospital Zurich, Zurich, Switzerland; 408 Institute of Molecular Life Sciences, University of Zurich, Zurich, Switzerland; 409 MRC Human Genetics Unit, MRC IGMM, University of Edinburgh, Edinburgh, UK; 410 Women’s Cancer Program at the Samuel Oschin Comprehensive Cancer Institute, Cedars-Sinai Medical Center, Los Angeles, CA USA; 411 Department of Biology, Bioinformatics Group, Division of Molecular Biology, Faculty of Science, University of Zagreb, Zagreb, Croatia; 412 Department for Internal Medicine II, University Hospital Schleswig-Holstein, Kiel, Germany; 413 Genetics and Molecular Pathology, SA Pathology, Adelaide, SA Australia; 414 Department of Gastric Surgery, National Cancer Center Hospital, Tokyo, Japan; 415 Department of Bioinformatics, Division of Cancer Genomics, National Cancer Center Research Institute, Tokyo, Japan; 416 A.A. Kharkevich Institute of Information Transmission Problems, Moscow, Russia; 417 Oncology and Immunology, Dmitry Rogachev National Research Center of Pediatric Hematology, Moscow, Russia; 418 Skolkovo Institute of Science and Technology, Moscow, Russia; 419 Department of Surgery, The George Washington University, School of Medicine and Health Science, Washington, DC USA; 420 Endocrine Oncology Branch, Center for Cancer Research, National Cancer Institute, National Institutes of Health, Bethesda, MD USA; 421 Melanoma Institute Australia, Macquarie University, Sydney, NSW Australia; 422 MIT Computer Science and Artificial Intelligence Laboratory, Massachusetts Institute of Technology, Cambridge, MA USA; 423 Tissue Pathology and Diagnostic Oncology, Royal Prince Alfred Hospital, Sydney, NSW Australia; 424 Cholangiocarcinoma Screening and Care Program and Liver Fluke and Cholangiocarcinoma Research Centre, Faculty of Medicine, Khon Kaen University, Khon Kaen, Thailand; 425 Controlled Department and Institution, New York, NY USA; 426 Englander Institute for Precision Medicine, Weill Cornell Medicine, New York, NY USA; 427 National Cancer Center, Gyeonggi, South Korea; 428 Department of Biochemistry, College of Medicine, Ewha Womans University, Seoul, South Korea; 429 Health Sciences Department of Biomedical Informatics, University of California San Diego, La Jolla, CA USA; 430 Research Core Center, National Cancer Centre Korea, Goyang-si, South Korea; 431 Department of Health Sciences and Technology, Sungkyunkwan University School of Medicine, Seoul, South Korea; 432 Samsung Genome Institute, Seoul, South Korea; 433 Breast Oncology Program, Dana-Farber/Brigham and Women’s Cancer Center, Boston, MA USA; 434 Department of Surgery, Memorial Sloan Kettering Cancer Center, New York, NY USA; 435 Division of Breast Surgery, Brigham and Women’s Hospital, Boston, MA USA; 436 Integrative Bioinformatics Support Group, National Institute of Environmental Health Sciences (NIEHS), Durham, NC USA; 437 Department of Clinical Science, University of Bergen, Bergen, Norway; 438 Center For Medical Innovation, Seoul National University Hospital, Seoul, South Korea; 439 Department of Internal Medicine, Seoul National University Hospital, Seoul, South Korea; 440 Institute of Computer Science, Polish Academy of Sciences, Warsawa, Poland; 441 Functional and Structural Genomics, German Cancer Research Center (DKFZ), Heidelberg, Germany; 442 Laboratory of Translational Genomics, Division of Cancer Epidemiology and Genetics, National Cancer Institute, , National Institutes of Health, Bethesda, MD USA; 443 Institute for Medical Informatics Statistics and Epidemiology, University of Leipzig, Leipzig, Germany; 444 Morgan Welch Inflammatory Breast Cancer Research Program and Clinic, The University of Texas MD Anderson Cancer Center, Houston, TX USA; 445 Department of Hematology and Oncology, Georg-Augusts-University of Göttingen, Göttingen, Germany; 446 Institute of Cell Biology (Cancer Research), University of Duisburg-Essen, Essen, Germany; 447 King’s College London and Guy’s and St. Thomas’ NHS Foundation Trust, London, UK; 448 Center for Epigenetics, Van Andel Research Institute, Grand Rapids, MI USA; 449 The University of Queensland Centre for Clinical Research, Royal Brisbane and Women’s Hospital, Herston, QLD Australia; 450 Department of Pediatric Oncology and Hematology, University of Cologne, Cologne, Germany; 451 University of Düsseldorf, Düsseldorf, Germany; 452 Department of Pathology, Institut Jules Bordet, Brussels, Belgium; 453 Institute of Biomedicine, Sahlgrenska Academy at University of Gothenburg, Gothenburg, Sweden; 454 Children’s Medical Research Institute, Sydney, NSW Australia; 455 ILSbio, LLC Biobank, Chestertown, MD USA; 456 Division of Genetics and Genomics, Boston Children’s Hospital, Harvard Medical School, Boston, MA USA; 457 Institute for Bioengineering and Biopharmaceutical Research (IBBR), Hanyang University, Seoul, South Korea; 458 Department of Statistics, University of California Santa Cruz, Santa Cruz, CA USA; 459 National Genotyping Center, Institute of Biomedical Sciences, Academia Sinica, Taipei, Taiwan; 460 Department of Vertebrate Genomics/Otto Warburg Laboratory Gene Regulation and Systems Biology of Cancer, Max Planck Institute for Molecular Genetics, Berlin, Germany; 461 McGill University and Genome Quebec Innovation Centre, Montreal, QC Canada; 462 biobyte solutions GmbH, Heidelberg, Germany; 463 Gynecologic Oncology, NYU Laura and Isaac Perlmutter Cancer Center, New York University, New York, NY USA; 464 Division of Oncology, Stem Cell Biology Section, Washington University School of Medicine, St. Louis, MO USA; 465 Department of Systems Biology, The University of Texas MD Anderson Cancer Center, Houston, TX USA; 466 Harvard University, Cambridge, MA USA; 467 Urologic Oncology Branch, Center for Cancer Research, National Cancer Institute, National Institutes of Health, Bethesda, MD USA; 468 University of Oslo, Oslo, Norway; 469 University of Toronto, Toronto, ON Canada; 470 Peking University, Beijing, China; 471 School of Life Sciences, Peking University, Beijing, China; 472 Leidos Biomedical Research, Inc, McLean, VA USA; 473 Hematology, Hospital Clinic, Institut d’Investigacions Biomèdiques August Pi i Sunyer (IDIBAPS), University of Barcelona, Barcelona, Spain; 474 Second Military Medical University, Shanghai, China; 475 Chinese Cancer Genome Consortium, Shenzhen, China; 476 Department of Medical Oncology, Beijing Hospital, Beijing, China; 477 Laboratory of Molecular Oncology, Key Laboratory of Carcinogenesis and Translational Research (Ministry of Education), Peking University Cancer Hospital and Institute, Beijing, China; 478 School of Medicine/School of Mathematics and Statistics, University of St. Andrews, St, Andrews, Fife UK; 479 Institute for Systems Biology, Seattle, WA USA; 480 Department of Biochemistry and Molecular Biology, Faculty of Medicine, University Institute of Oncology-IUOPA, Oviedo, Spain; 481 Institut Bergonié, Bordeaux, France; 482 Cancer Unit, MRC University of Cambridge, Cambridge, UK; 483 Department of Pathology and Laboratory Medicine, Center for Personalized Medicine, Children’s Hospital Los Angeles, Los Angeles, CA USA; 484 John Curtin School of Medical Research, Canberra, ACT Australia; 485 MVZ Department of Oncology, PraxisClinic am Johannisplatz, Leipzig, Germany; 486 Department of Information Technology, Ghent University, Ghent, Belgium; 487 Department of Plant Biotechnology and Bioinformatics, Ghent University, Ghent, Belgium; 488 Institute for Genomic Medicine, Nationwide Children’s Hospital, Columbus, OH USA; 489 Computational Biology Program, School of Medicine, Oregon Health and Science University, Portland, OR USA; 490 Department of Surgery, Duke University, Durham, NC USA; 491 Institució Catalana de Recerca i Estudis Avançats (ICREA), Barcelona, Spain; 492 Institut Català de Paleontologia Miquel Crusafont, Universitat Autònoma de Barcelona, Barcelona, Spain; 493 University of Glasgow, Glasgow, UK; 494 Institut d’Investigacions Biomèdiques August Pi i Sunyer (IDIBAPS), Barcelona, Spain; 495 Division of Oncology, Washington University School of Medicine, St. Louis, MO USA; 496 Department of Surgery and Cancer, Imperial College, London, INY UK; 497 Applications Department, Oxford Nanopore Technologies, Oxford, UK; 498 Department of Obstetrics, Gynecology and Reproductive Services, University of California San Francisco, San Francisco, CA USA; 499 Department of Biochemistry and Molecular Medicine, University California at Davis, Sacramento, CA USA; 500 STTARR Innovation Facility, Princess Margaret Cancer Centre, Toronto, ON Canada; 501 Discipline of Surgery, Western Sydney University, Penrith, NSW Australia; 502 Yale School of Medicine, Yale University, New Haven, CT USA; 503 Department of Genetics, Lineberger Comprehensive Cancer Center, University of North Carolina at Chapel Hill, Chapel Hill, NC USA; 504 Departments of Neurology and Neurosurgery, Henry Ford Hospital, Detroit, MI USA; 505 Precision Oncology, OHSU Knight Cancer Institute, Oregon Health and Science University, Portland, OR USA; 506 Institute of Pathology, University Medical Center Hamburg-Eppendorf, Hamburg, Germany; 507 Department of Health Sciences, Faculty of Medical Sciences, Kyushu University, Fukuoka, Japan; 508 Heidelberg Academy of Sciences and Humanities, Heidelberg, Germany; 509 Department of Clinical Pathology, University of Melbourne, Melbourne, VIC, Australia; 510 Department of Pathology, Roswell Park Cancer Institute, Buffalo, NY USA; 511 Department of Computer Science, University of Helsinki, Helsinki, Finland; 512 Institute of Biotechnology, University of Helsinki, Helsinki, Finland; 513 Organismal and Evolutionary Biology Research Programme, University of Helsinki, Helsinki, Finland; 514 Department of Obstetrics and Gynecology, Division of Gynecologic Oncology, Washington University School of Medicine, St. Louis, MO USA; 515 Penrose St. Francis Health Services, Colorado Springs, CO USA; 516 Institute of Pathology, Ulm University and University Hospital of Ulm, Ulm, Germany; 517 National Cancer Center, Tokyo, Japan; 518 Genome Institute of Singapore, Singapore, Singapore; 519 32Program in Computational Biology and Bioinformatics, Yale University, New Haven, CT USA; 520 German Cancer Aid, Bonn, Germany; 521 Programme in Cancer and Stem Cell Biology, Centre for Computational Biology, Duke-NUS Medical School, Singapore, Singapore; 522 The Chinese University of Hong Kong, Shatin, NT, Hong Kong China; 523 Fourth Military Medical University, Shaanxi, China; 524 The University of Cambridge School of Clinical Medicine, Cambridge, UK; 525 St. Jude Children’s Research Hospital, Memphis, TN USA; 526 University Health Network, Princess Margaret Cancer Centre, Toronto, ON Canada; 527 Center for Biomolecular Science and Engineering, University of California Santa Cruz, Santa Cruz, CA USA; 528 Department of Medicine, University of Chicago, Chicago, IL USA; 529 Department of Neurology, Mayo Clinic, Rochester, MN USA; 530 Cambridge Oesophagogastric Centre, Cambridge University Hospitals NHS Foundation Trust, Cambridge, UK; 531 Department of Computer Science, Carleton College, Northfield, MN USA; 532 Institute of Cancer Sciences, College of Medical Veterinary and Life Sciences, University of Glasgow, Glasgow, UK; 533 Department of Epidemiology, University of Alabama at Birmingham, Birmingham, AL USA; 534 HudsonAlpha Institute for Biotechnology, Huntsville, AL USA; 535 O’Neal Comprehensive Cancer Center, University of Alabama at Birmingham, Birmingham, AL USA; 536 Department of Pathology, Keio University School of Medicine, Tokyo, Japan; 537 Department of Hepatobiliary and Pancreatic Oncology, National Cancer Center Hospital, Tokyo, Japan; 538 Sage Bionetworks, Seattle, WA USA; 539 Lymphoma Genomic Translational Research Laboratory, National Cancer Centre, Singapore, Singapore; 540 Department of Clinical Pathology, Robert-Bosch-Hospital, Stuttgart, Germany; 541 Department of Cell and Systems Biology, University of Toronto, Toronto, ON Canada; 542 Department of Biosciences and Nutrition, Karolinska Institutet, Stockholm, Sweden; 543 Center for Liver Cancer, Research Institute and Hospital, National Cancer Center, Gyeonggi, South Korea; 544 Division of Hematology-Oncology, Samsung Medical Center, Sungkyunkwan University School of Medicine, Seoul, South Korea; 545 Samsung Advanced Institute for Health Sciences and Technology, Sungkyunkwan University School of Medicine, Seoul, South Korea; 546 Cheonan Industry-Academic Collaboration Foundation, Sangmyung University, Cheonan, South Korea; 547 NYU Langone Medical Center, New York, NY USA; 548 Department of Hematology and Medical Oncology, Cleveland Clinic, Cleveland, OH USA; 549 Department of Radiation Oncology, University of California San Francisco, San Francisco, CA USA; 550 Department of Health Sciences Research, Mayo Clinic, Rochester, MN USA; 551 Helen F. Graham Cancer Center at Christiana Care Health Systems, Newark, DE USA; 552 Heidelberg University Hospital, Heidelberg, Germany; 553 CSRA Incorporated, Fairfax, VA USA; 554 Research Department of Pathology, University College London Cancer Institute, London, UK; 555 Department of Research Oncology, Guy’s Hospital, King’s Health Partners AHSC, King’s College London School of Medicine, London, UK; 556 Faculty of Medicine and Health Sciences, Macquarie University, Sydney, NSW Australia; 557 University Hospital of Minjoz, INSERM UMR 1098, Besançon, France; 558 Spanish National Cancer Research Centre, Madrid, Spain; 559 Center of Digestive Diseases and Liver Transplantation, Fundeni Clinical Institute, Bucharest, Romania; 560 Cureline, Inc, South San Francisco, CA USA; 561 St. Luke’s Cancer Centre, Royal Surrey County Hospital NHS Foundation Trust, Guildford, UK; 562 Cambridge Breast Unit, Addenbrooke’s Hospital, Cambridge University Hospital NHS Foundation Trust and NIHR Cambridge Biomedical Research Centre, Cambridge, UK; 563 East of Scotland Breast Service, Ninewells Hospital, Aberdeen, UK; 564 Department of Genetics, Microbiology and Statistics, University of Barcelona, IRSJD, IBUB, Barcelona, Spain; 565 Department of Obstetrics and Gynecology, Medical College of Wisconsin, Milwaukee, WI USA; 566 Hematology and Medical Oncology, Winship Cancer Institute of Emory University, Atlanta, GA USA; 567 Department of Computer Science, Princeton University, Princeton, NJ USA; 568 Vanderbilt Ingram Cancer Center, Vanderbilt University, Nashville, TN USA; 569 Ohio State University College of Medicine and Arthur G. James Comprehensive Cancer Center, Columbus, OH USA; 570 Department of Surgery, Yokohama City University Graduate School of Medicine, Kanagawa, Japan; 571 Division of Chromatin Networks, German Cancer Research Center (DKFZ) and BioQuant, Heidelberg, Germany; 572 Research Computing Center, University of North Carolina at Chapel Hill, Chapel Hill, NC USA; 573 School of Molecular Biosciences and Center for Reproductive Biology, Washington State University, Pullman, WA USA; 574 Finsen Laboratory and Biotech Research and Innovation Centre (BRIC), University of Copenhagen, Copenhagen, Denmark; 575 Department of Laboratory Medicine and Pathobiology, University of Toronto, Toronto, ON Canada; 576 Department of Pathology, Human Oncology and Pathogenesis Program, Memorial Sloan Kettering Cancer Center, New York, NY USA; 577 University Hospital Giessen, Pediatric Hematology and Oncology, Giessen, Germany; 578 Oncologie Sénologie, ICM Institut Régional du Cancer, Montpellier, France; 579 Institute of Clinical Molecular Biology, Christian-Albrechts-University, Kiel, Germany; 580 Institute of Pathology, University of Wuerzburg, Wuerzburg, Germany; 581 Department of Urology, North Bristol NHS Trust, Bristol, UK; 582 SingHealth, Duke-NUS Institute of Precision Medicine, National Heart Centre Singapore, Singapore, Singapore; 583 Department of Computer Science, University of Toronto, Toronto, ON Canada; 584 Bern Center for Precision Medicine, University Hospital of Bern, University of Bern, Bern, Switzerland; 585 Englander Institute for Precision Medicine, Weill Cornell Medicine and New York Presbyterian Hospital, New York, NY USA; 586 Meyer Cancer Center, Weill Cornell Medicine, New York, NY USA; 587 Pathology and Laboratory, Weill Cornell Medical College, New York, NY USA; 588 Vall d’Hebron Institute of Oncology: VHIO, Barcelona, Spain; 589 General and Hepatobiliary-Biliary Surgery, Pancreas Institute, University and Hospital Trust of Verona, Verona, Italy; 590 National Centre for Biological Sciences, Tata Institute of Fundamental Research, Bangalore, India; 591 Indiana University, Bloomington, IN USA; 592 Department of Pathology, GZA-ZNA Hospitals, Antwerp, Belgium; 593 Analytical Biological Services, Inc, Wilmington, DE USA; 594 Sydney Medical School, University of Sydney, Sydney, NSW Australia; 595 cBio Center, Dana-Farber Cancer Institute, Harvard Medical School, Boston, MA USA; 596 Department of Cell Biology, Harvard Medical School, Boston, MA USA; 597 Advanced Centre for Treatment Research and Education in Cancer, Tata Memorial Centre, Navi Mumbai, Maharashtra India; 598 School of Environmental and Life Sciences, Faculty of Science, The University of Newcastle, Ourimbah, NSW Australia; 599 Department of Dermatology, University Hospital of Essen, Essen, Germany; 600 Bioinformatics and Omics Data Analytics, German Cancer Research Center (DKFZ), Heidelberg, Germany; 601 Department of Urology, Charité Universitätsmedizin Berlin, Berlin, Germany; 602 Martini-Clinic, Prostate Cancer Center, University Medical Center Hamburg-Eppendorf, Hamburg, Germany; 603 Department of General Internal Medicine, University of Kiel, Kiel, Germany; 604 German Cancer Consortium (DKTK), Partner site Berlin, Berlin, Germany; 605 Cancer Research Institute, Beth Israel Deaconess Medical Center, Boston, MA USA; 606 University of Pittsburgh, Pittsburgh, PA USA; 607 Department of Ophthalmology and Ocular Genomics Institute, Massachusetts Eye and Ear, Harvard Medical School, Boston, MA USA; 608 Center for Psychiatric Genetics, NorthShore University HealthSystem, Evanston, IL USA; 609 Van Andel Research Institute, Grand Rapids, MI USA; 610 Laboratory of Molecular Medicine, Human Genome Center, Institute of Medical Science, University of Tokyo, Tokyo, Japan; 611 Japan Agency for Medical Research and Development, Tokyo, Japan; 612 Korea University, Seoul, South Korea; 613 Murtha Cancer Center, Walter Reed National Military Medical Center, Bethesda, MD USA; 614 Human Genetics, University of Kiel, Kiel, Germany; 615 Department of Oncologic Pathology, Dana-Farber Cancer Institute, Harvard Medical School, Boston, MA USA; 616 Oregon Health and Science University, Portland, OR USA; 617 Center for RNA Interference and Noncoding RNA, The University of Texas MD Anderson Cancer Center, Houston, TX USA; 618 Department of Experimental Therapeutics, The University of Texas MD Anderson Cancer Center, Houston, TX USA; 619 Department of Gynecologic Oncology and Reproductive Medicine, The University of Texas MD Anderson Cancer Center, Houston, TX USA; 620 University Hospitals Coventry and Warwickshire NHS Trust, Coventry, UK; 621 Department of Radiation Oncology, Radboud University Nijmegen Medical Centre, Nijmegen, GA The Netherlands; 622 Institute for Genomics and Systems Biology, University of Chicago, Chicago, IL USA; 623 Clinic for Hematology and Oncology, St.-Antonius-Hospital, Eschweiler, Germany; 624 Computational and Systems Biology Program, Memorial Sloan Kettering Cancer Center, New York, NY USA; 625 University of Iceland, Reykjavik, Iceland; 626 Division of Computational Genomics and Systems Genetics, German Cancer Research Center (DKFZ), Heidelberg, Germany; 627 Dundee Cancer Centre, Ninewells Hospital, Dundee, UK; 628 Department for Internal Medicine III, University of Ulm and University Hospital of Ulm, Ulm, Germany; 629 Institut Curie, INSERM Unit 830, Paris, France; 630 Department of Gastroenterology and Hepatology, Yokohama City University Graduate School of Medicine, Kanagawa, Japan; 631 Department of Laboratory Medicine, Radboud University Nijmegen Medical Centre, Nijmegen, GA The Netherlands; 632 Division of Cancer Genome Research, German Cancer Research Center (DKFZ), Heidelberg, Germany; 633 Department of General Surgery, Singapore General Hospital, Singapore, Singapore; 634 Cancer Science Institute of Singapore, National University of Singapore, Singapore, Singapore; 635 Department of Medical and Clinical Genetics, Genome-Scale Biology Research Program, University of Helsinki, Helsinki, Finland; 636 East Anglian Medical Genetics Service, Cambridge University Hospitals NHS Foundation Trust, Cambridge, UK; 637 Irving Institute for Cancer Dynamics, Columbia University, New York, NY USA; 638 Institute of Molecular and Cell Biology, Singapore, Singapore; 639 Laboratory of Cancer Epigenome, Division of Medical Science, National Cancer Centre Singapore, Singapore, Singapore; 640 Universite Lyon, INCa-Synergie, Centre Léon Bérard, Lyon, France; 641 Department of Urology, Mayo Clinic, Rochester, MN USA; 642 Royal National Orthopaedic Hospital - Stanmore, Stanmore, Middlesex UK; 643 Department of Biochemistry, Genetics and Immunology, University of Vigo, Vigo, Spain; 644 Giovanni Paolo II / I.R.C.C.S. Cancer Institute, Bari, BA Italy; 645 Neuroblastoma Genomics, German Cancer Research Center (DKFZ), Heidelberg, Germany; 646 Fondazione Policlinico Universitario Gemelli IRCCS, Rome, Italy, Rome, Italy; 647 University of Verona, Verona, Italy; 648 Centre National de Génotypage, CEA - Institute de Génomique, Evry, France; 649 CAPHRI Research School, Maastricht University, Maastricht, ER The Netherlands; 650 Department of Biopathology, Centre Léon Bérard, Lyon, France; 651 Université Claude Bernard Lyon 1, Villeurbanne, France; 652 Core Research for Evolutional Science and Technology (CREST), JST, Tokyo, Japan; 653 Department of Biological Sciences, Laboratory for Medical Science Mathematics, Graduate School of Science, University of Tokyo, Yokohama, Japan; 654 Department of Medical Science Mathematics, Medical Research Institute, Tokyo Medical and Dental University (TMDU), Tokyo, Japan; 655 Cancer Ageing and Somatic Mutation Programme, Wellcome Sanger Institute, Hinxton, UK; 656 University Hospitals Birmingham NHS Foundation Trust, Birmingham, UK; 657 Centre for Cancer Research and Cell Biology, Queen’s University, Belfast, UK; 658 Breast Medical Oncology, The University of Texas MD Anderson Cancer Center, Houston, TX USA; 659 Department of Surgery, Johns Hopkins University School of Medicine, Baltimore, MD USA; 660 Department of Oncology-Pathology, Science for Life Laboratory, Karolinska Institute, Stockholm, Sweden; 661 School of Cancer Sciences, Faculty of Medicine, University of Southampton, Southampton, UK; 662 Department of Gene Technology, Tallinn University of Technology, Tallinn, Estonia; 663 Genetics and Genome Biology Program, SickKids Research Institute, The Hospital for Sick Children, Toronto, ON Canada; 664 Departments of Neurosurgery and Hematology and Medical Oncology, Winship Cancer Institute and School of Medicine, Emory University, Atlanta, GA USA; 665 Department of Clinical and Molecular Medicine, Faculty of Medicine and Health Sciences, Norwegian University of Science and Technology, Trondheim, Norway; 666 Argmix Consulting, North Vancouver, BC Canada; 667 Department of Information Technology, Ghent University, Interuniversitair Micro-Electronica Centrum (IMEC), Ghent, Belgium; 668 Nuffield Department of Surgical Sciences, John Radcliffe Hospital, University of Oxford, Oxford, UK; 669 Institute of Mathematics and Computer Science, University of Latvia, Riga, LV Latvia; 670 Discipline of Pathology, Sydney Medical School, University of Sydney, Sydney, NSW Australia; 671 Department of Applied Mathematics and Theoretical Physics, Centre for Mathematical Sciences, University of Cambridge, Cambridge, UK; 672 Department of Epidemiology and Biostatistics, Memorial Sloan Kettering Cancer Center, New York, NY USA; 673 Department of Statistics, Columbia University, New York, NY USA; 674 Department of Immunology, Genetics and Pathology, Science for Life Laboratory, Uppsala University, Uppsala, Sweden; 675 School of Electronic and Information Engineering, Xi’an Jiaotong University, Xi’an, China; 676 Department of Histopathology, Cambridge University Hospitals NHS Foundation Trust, Cambridge, UK; 677 Oxford NIHR Biomedical Research Centre, University of Oxford, Oxford, UK; 678 Georgia Regents University Cancer Center, Augusta, GA USA; 679 Wythenshawe Hospital, Manchester, UK; 680 Department of Genetics, Washington University School of Medicine, St.Louis, MO USA; 681 Department of Biological Oceanography, Leibniz Institute of Baltic Sea Research, Rostock, Germany; 682 Wellcome Centre for Human Genetics, University of Oxford, Oxford, UK; 683 Department of Molecular and Human Genetics, Baylor College of Medicine, Houston, TX USA; 684 Thoracic Oncology Laboratory, Mayo Clinic, Rochester, MN USA; 685 Institute for Genomic Medicine, Nationwide Children’s Hospital, Columbus, OH USA; 686 Department of Obstetrics and Gynecology, Division of Gynecologic Oncology, Mayo Clinic, Rochester, MN USA; 687 International Institute for Molecular Oncology, Poznań, Poland; 688 Poznan University of Medical Sciences, Poznań, Poland; 689 Genomics and Proteomics Core Facility High Throughput Sequencing Unit, German Cancer Research Center (DKFZ), Heidelberg, Germany; 690 NCCS-VARI Translational Research Laboratory, National Cancer Centre Singapore, Singapore, Singapore; 691 Edison Family Center for Genome Sciences and Systems Biology, Washington University, St. Louis, MO USA; 692 MRC-University of Glasgow Centre for Virus Research, Glasgow, UK; 693 Department of Medical Informatics and Clinical Epidemiology, Division of Bioinformatics and Computational Biology, OHSU Knight Cancer Institute, Oregon Health and Science University, Portland, OR USA; 694 School of Electronic Information and Communications, Huazhong University of Science and Technology, Wuhan, China; 695 Department of Applied Mathematics and Statistics, Johns Hopkins University, Baltimore, MD USA; 696 Department of Cancer Genome Informatics, Graduate School of Medicine, Osaka University, Osaka, Japan; 697 Institute of Computer Science, Heidelberg University, Heidelberg, Germany; 698 School of Mathematics and Statistics, University of Sydney, Sydney, NSW Australia; 699 Ben May Department for Cancer Research, University of Chicago, Chicago, IL USA; 700 Department of Human Genetics, University of Chicago, Chicago, IL USA; 701 Tri-Institutional PhD Program in Computational Biology and Medicine, Weill Cornell Medicine, New York, NY USA; 702 The First Affiliated Hospital, Xi’an Jiaotong University, Xi’an, China; 703 Department of Medicine and Therapeutics, The Chinese University of Hong Kong, Shatin, NT, Hong Kong China; 704 Department of Biostatistics, The University of Texas MD Anderson Cancer Center, Houston, TX USA; 705 Duke-NUS Medical School, Singapore, Singapore; 706 Department of Surgery, Ruijin Hospital, Shanghai Jiaotong University School of Medicine, Shanghai, China; 707 School of Computing Science, University of Glasgow, Glasgow, UK; 708 Division of Orthopaedic Surgery, Oslo University Hospital, Oslo, Norway; 709 Eastern Clinical School, Monash University, Melbourne, VIC Australia; 710 Epworth HealthCare, Richmond, VIC Australia; 711 Department of Biostatistics and Computational Biology, Dana-Farber Cancer Institute and Harvard Medical School, Boston, MA USA; 712 Department of Biomedical Informatics, College of Medicine, The Ohio State University, Columbus, OH USA; 713 The Ohio State University Comprehensive Cancer Center (OSUCCC – James), Columbus, OH USA; 714 The University of Texas School of Biomedical Informatics (SBMI) at Houston, Houston, TX USA; 715 Department of Biostatistics, University of North Carolina at Chapel Hill, Chapel Hill, NC USA; 716 Department of Biochemistry and Molecular Genetics, Feinberg School of Medicine, Northwestern University, Chicago, IL USA; 717 Faculty of Medicine and Health, University of Sydney, Sydney, NSW Australia; 718 Department of Pathology, Erasmus Medical Center Rotterdam, Rotterdam, GD The Netherlands; 719 Division of Molecular Carcinogenesis, The Netherlands Cancer Institute, Amsterdam, CX The Netherlands; 720 Institute of Molecular Life Sciences and Swiss Institute of Bioinformatics, University of Zurich, Zurich, Switzerland

**Keywords:** Cancer genomics, Cancer genomics

## Abstract

Cancer is driven by genetic change, and the advent of massively parallel sequencing has enabled systematic documentation of this variation at the whole-genome scale^1–3^. Here we report the integrative analysis of 2,658 whole-cancer genomes and their matching normal tissues across 38 tumour types from the Pan-Cancer Analysis of Whole Genomes (PCAWG) Consortium of the International Cancer Genome Consortium (ICGC) and The Cancer Genome Atlas (TCGA). We describe the generation of the PCAWG resource, facilitated by international data sharing using compute clouds. On average, cancer genomes contained 4–5 driver mutations when combining coding and non-coding genomic elements; however, in around 5% of cases no drivers were identified, suggesting that cancer driver discovery is not yet complete. Chromothripsis, in which many clustered structural variants arise in a single catastrophic event, is frequently an early event in tumour evolution; in acral melanoma, for example, these events precede most somatic point mutations and affect several cancer-associated genes simultaneously. Cancers with abnormal telomere maintenance often originate from tissues with low replicative activity and show several mechanisms of preventing telomere attrition to critical levels. Common and rare germline variants affect patterns of somatic mutation, including point mutations, structural variants and somatic retrotransposition. A collection of papers from the PCAWG Consortium describes non-coding mutations that drive cancer beyond those in the *TERT* promoter^4^; identifies new signatures of mutational processes that cause base substitutions, small insertions and deletions and structural variation^5,6^; analyses timings and patterns of tumour evolution^7^; describes the diverse transcriptional consequences of somatic mutation on splicing, expression levels, fusion genes and promoter activity^8,9^; and evaluates a range of more-specialized features of cancer genomes^8,10–18^.

## Abstract (web summary)

The flagship paper of the ICGC/TCGA Pan-Cancer Analysis of Whole Genomes Consortium describes the generation of the integrative analyses of 2,658 cancer whole genomes and their matching normal tissues across 38 tumour types, the structures for international data sharing and standardized analyses, and the main scientific findings from across the consortium studies.

## Main

Cancer is the second most-frequent cause of death worldwide, killing more than 8 million people every year; the incidence of cancer is expected to increase by more than 50% over the coming decades^19,20^. ‘Cancer’ is a catch-all term used to denote a set of diseases characterized by autonomous expansion and spread of a somatic clone. To achieve this behaviour, the cancer clone must co-opt multiple cellular pathways that enable it to disregard the normal constraints on cell growth, modify the local microenvironment to favour its own proliferation, invade through tissue barriers, spread to other organs and evade immune surveillance^21^. No single cellular program directs these behaviours. Rather, there is a large pool of potential pathogenic abnormalities from which individual cancers draw their own combinations: the commonalities of macroscopic features across tumours belie a vastly heterogeneous landscape of cellular abnormalities.

This heterogeneity arises from the stochastic nature of Darwinian evolution. There are three preconditions for Darwinian evolution: characteristics must vary within a population; this variation must be heritable from parent to offspring; and there must be competition for survival within the population. In the context of somatic cells, heritable variation arises from mutations acquired stochastically throughout life, notwithstanding additional contributions from germline and epigenetic variation. A subset of these mutations alter the cellular phenotype, and a small subset of those variants confer an advantage on clones during the competition to escape the tight physiological controls wired into somatic cells. Mutations that provide a selective advantage to the clone are termed driver mutations, as opposed to selectively neutral passenger mutations.

Initial studies using massively parallel sequencing demonstrated the feasibility of identifying every somatic point mutation, copy-number change and structural variant (SV) in a given cancer^1–3^. In 2008, recognizing the opportunity that this advance in technology provided, the global cancer genomics community established the ICGC with the goal of systematically documenting the somatic mutations that drive common tumour types^22^.

## The pan-cancer analysis of whole genomes

**Extended Data Fig. 1:**
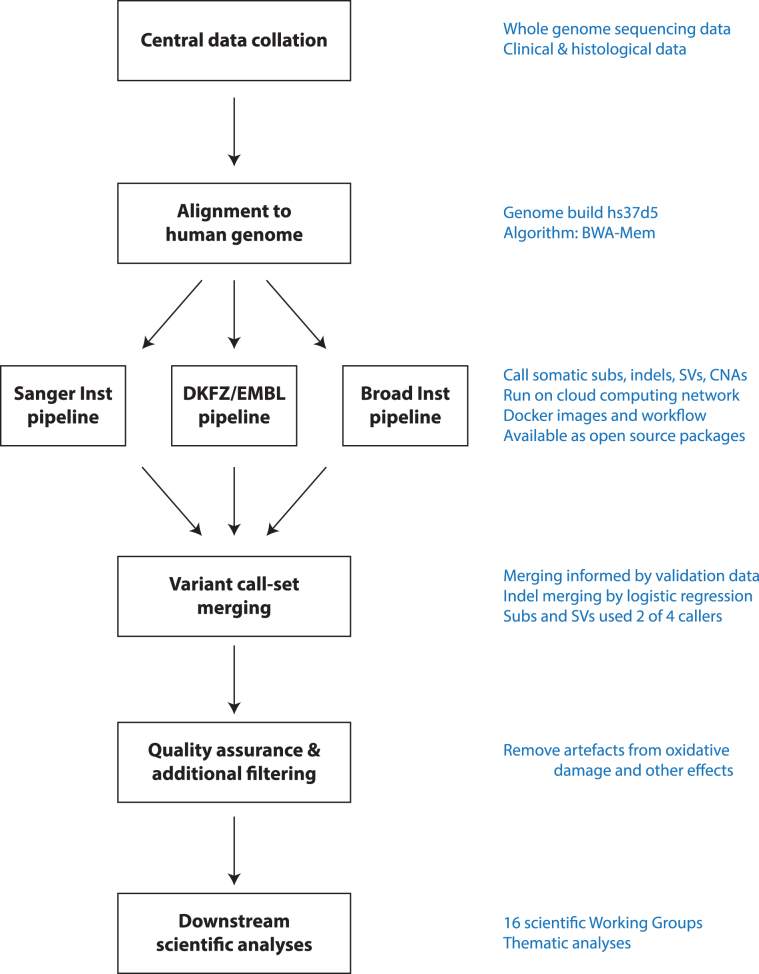
Flow-chart showing key steps in the analysis of PCAWG genomes. After alignment to the genome, somatic mutations were identified by three pipelines, with subsequent merging into a consensus variant set used for downstream scientific analyses. Subs, substitutions; DKFZ/EMBL, the German Cancer Research Centre (DKFZ) and Europen Molecular Biology Laboratory (EMBL).

The expansion of whole-genome sequencing studies from individual ICGC and TCGA working groups presented the opportunity to undertake a meta-analysis of genomic features across tumour types. To achieve this, the PCAWG Consortium was established. A Technical Working Group implemented the informatics analyses by aggregating the raw sequencing data from different working groups that studied individual tumour types, aligning the sequences to the human genome and delivering a set of high-quality somatic mutation calls for downstream analysis (Extended Data Fig. 1). Given the recent meta-analysis of exome data from the TCGA Pan-Cancer Atlas^23–25^, scientific working groups concentrated their efforts on analyses best-informed by whole-genome sequencing data.

We collected genome data from 2,834 donors (Extended Data Table 1), of which 176 were excluded after quality assurance. A further 75 had minor issues that could affect some of the analyses (grey-listed donors) and 2,583 had data of optimal quality (white-listed donors) (Supplementary Table 1). Across the 2,658 white- and grey-listed donors, whole-genome sequencing data were available from 2,605 primary tumours and 173 metastases or local recurrences. Mean read coverage was 39× for normal samples, whereas tumours had a bimodal coverage distribution with modes at 38× and 60× (Supplementary Fig. 1). RNA-sequencing data were available for 1,222 donors. The final cohort comprised 1,469 men (55%) and 1,189 women (45%), with a mean age of 56 years (range, 1–90 years) across 38 tumour types (Extended Data Table 1 and Supplementary Table 1).

To identify somatic mutations, we analysed all 6,835 samples using a uniform set of algorithms for alignment, variant calling and quality control (Extended Data Fig. 1, Supplementary Fig. 2 and Supplementary Methods 2). We used three established pipelines to call somatic single-nucleotide variations (SNVs), small insertions and deletions (indels), copy-number alterations (CNAs) and SVs. Somatic retrotransposition events, mitochondrial DNA mutations and telomere lengths were also called by bespoke algorithms. RNA-sequencing data were uniformly processed to call transcriptomic alterations. Germline variants identified by the three separate pipelines included single-nucleotide polymorphisms, indels, SVs and mobile-element insertions (Supplementary Table 2).

The requirement to uniformly realign and call variants on approximately 5,800 whole genomes presented considerable computational challenges, and raised ethical issues owing to the use of data from different jurisdictions (Extended Data Table 2). We used cloud computing^26,27^ to distribute alignment and variant calling across 13 data centres on 3 continents (Supplementary Table 3). Core pipelines were packaged into Docker containers^28^ as reproducible, stand-alone packages, which we have made available for download. Data repositories for raw and derived datasets, together with portals for data visualization and exploration, have also been created (Box 1 and Supplementary Table 4).

Box 1  Online resources for data access, visualization and analysis The PCAWG landing page (http://docs.icgc.org/pcawg) provides links to several data resources for interactive online browsing, analysis and download of PCAWG data and results (Supplementary Table 4).

**Direct download of PCAWG data**

Aligned PCAWG read data in BAM format are also available at the European Genome Phenome Archive (EGA; https://www.ebi.ac.uk/ega/search/site/pcawg under accession number EGAS00001001692). In addition, all open-tier PCAWG genomics data, as well as reference datasets used for analysis, can be downloaded from the ICGC Data Portal at http://docs.icgc.org/pcawg/data/. Controlled-tier genomic data, including SNVs and indels that originated from TCGA projects (in VCF format) and aligned reads (in BAM format) can be downloaded using the Score (https://www.overture.bio/) software package, which has accelerated and secure file transfer, as well as BAM slicing facilities to selectively download defined regions of genomic alignments.

**PCAWG computational pipelines**

The core alignment, somatic variant-calling, quality-control and variant consensus-generation pipelines used by PCAWG have each been packaged into portable cross-platform images using the Dockstore system^84^ and released under an Open Source licence that enables unrestricted use and redistribution. All PCAWG Dockstore images are available to the public at https://dockstore.org/organizations/PCAWG/collections/PCAWG.

**ICGC Data Portal**

The ICGC Data Portal^85^ (https://dcc.icgc.org) serves as the main entry point for accessing PCAWG datasets with a single uniform web interface and a high-performance data-download client. This uniform interface provides users with easy access to the myriad of PCAWG sequencing data and variant calls that reside in many repositories and compute clouds worldwide. Streaming technology^86^ provides users with high-level visualizations in real time of BAM and VCF files stored remotely on the Cancer Genome Collaboratory.

**UCSC Xena**

UCSC Xena^87^ (https://pcawg.xenahubs.net) visualizes all PCAWG primary results, including copy-number, gene-expression, gene-fusion and promoter-usage alterations, simple somatic mutations, large somatic structural variations, mutational signatures and phenotypic data. These open-access data are available through a public Xena hub, and consensus simple somatic mutations can be loaded to the local computer of a user via a private Xena hub. Kaplan–Meier plots, histograms, box plots, scatter plots and transcript-specific views offer additional visualization options and statistical analyses.

**The Expression Atlas**

The Expression Atlas (https://www.ebi.ac.uk/gxa/home) contains RNA-sequencing and expression microarray data for querying gene expression across tissues, cell types, developmental stages and/or experimental conditions^88^. Two different views of the data are provided: summarized expression levels for each tumour type and gene expression at the level of individual samples, including reference-gene expression datasets for matching normal tissues.

**PCAWG Scout**

PCAWG Scout (http://pcawgscout.bsc.es/) provides a framework for -omics workflow and website templating to generate on-demand, in-depth analyses of the PCAWG data that are openly available to the whole research community. Views of protected data are available that still safeguard sensitive data. Through the PCAWG Scout web interface, users can access an array of reports and visualizations that leverage on-demand bioinformatic computing infrastructure to produce results in real time, allowing users to discover trends as well as form and test hypotheses.

**Chromothripsis Explorer**

Chromothripsis Explorer (http://compbio.med.harvard.edu/chromothripsis/) is a portal that allows structural variation in the PCAWG dataset to be explored on an individual patient basis through the use of circos plots. Patterns of chromothripsis can also be explored in aggregated formats.

## Benchmarking of genetic variant calls

**Fig. 1:**
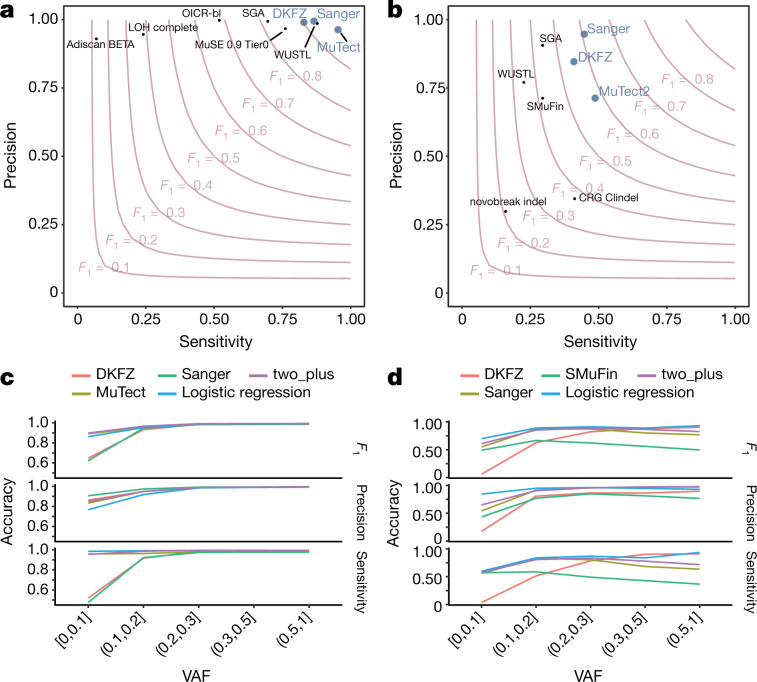
Validation of variant-calling pipelines in PCAWG. **a**, Scatter plot of estimated sensitivity and precision for somatic SNVs across individual algorithms assessed in the validation exercise across *n* = 63 PCAWG samples. Core algorithms included in the final PCAWG call set are shown in blue. **b**, Sensitivity and precision estimates across individual algorithms for somatic indels. **c**, Accuracy (precision, sensitivity and *F*_1_ score, defined as 2 × sensitivity × precision/(sensitivity + precision)) of somatic SNV calls across variant allele fractions (VAFs) for the core algorithms. The accuracy of two methods of combining variant calls (two-plus, which was used in the final dataset, and logistic regression) is also shown. **d**, Accuracy of indel calls across variant allele fractions.

To benchmark mutation calling, we ran the 3 core pipelines, together with 10 additional pipelines, on 63 representative tumour–normal genome pairs (Supplementary Note 1). For 50 of these cases, we performed validation by hybridization of tumour and matched normal DNA to a custom bait set with deep sequencing^29^. The 3 core somatic variant-calling pipelines had individual estimates of sensitivity of 80–90% to detect a true somatic SNV called by any of the 13 pipelines; more than 95% of SNV calls made by each of the core pipelines were genuine somatic variants (Fig. 1a). For indels—a more-challenging class of variants to identify with short-read sequencing—the 3 core algorithms had individual sensitivity estimates in the range of 40–50%, with precision of 70–95% (Fig. 1b). For individual SV algorithms, we estimated precision to be in the range 80–95% for samples in the 63-sample pilot dataset.

**Extended Data Fig. 2:**
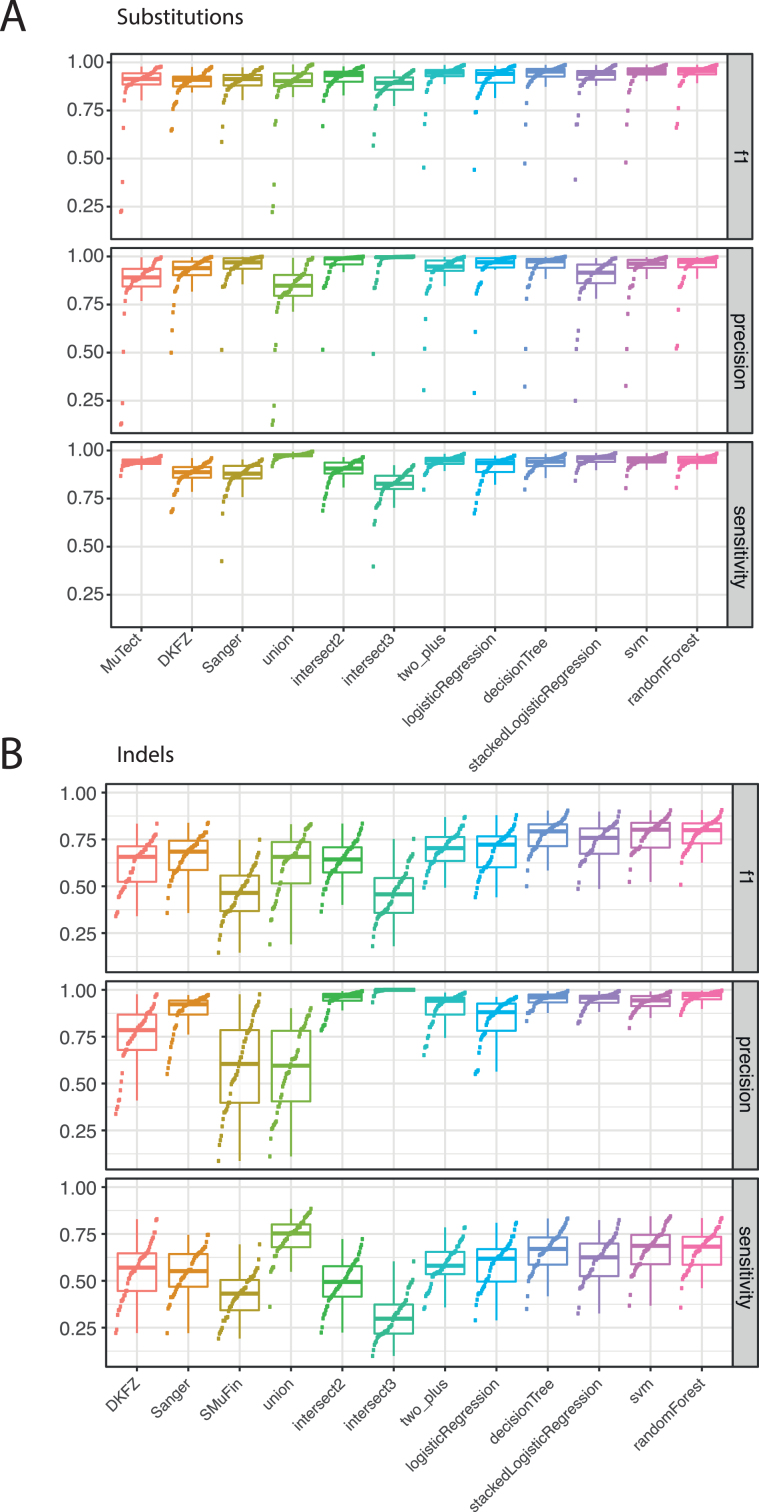
Distribution of accuracy estimates across algorithms and samples from validation data. **a**, *F*_1_ accuracy, precision and sensitivity estimates for somatic SNVs across the core algorithms and different approaches to merging the call-sets. The box plots demarcate the interquartile range and median of estimates across the *n* = 50 samples in the validation dataset. **b**, *F*_1_ accuracy, precision and sensitivity estimates for somatic indels (*n* = 50 samples). SVM, support vector machine; union, calls made by all variant-calling algorithms; intersect2, calls made by any combination of two variant-calling algorithms; intersect3, calls made by any three variant-calling algorithms.

Next, we defined a strategy to merge results from the three pipelines into one final call-set to be used for downstream scientific analyses (Methods and Supplementary Note 2). Sensitivity and precision of consensus somatic variant calls were 95% (90% confidence interval, 88–98%) and 95% (90% confidence interval, 71–99%), respectively, for SNVs (Extended Data Fig. 2). For somatic indels, sensitivity and precision were 60% (34–72%) and 91% (73–96%), respectively (Extended Data Fig. 2). Regarding somatic SVs, we estimate the sensitivity of merged calls to be 90% for true calls generated by any one pipeline; precision was estimated as 97.5%. The improvement in calling accuracy from combining different pipelines was most noticeable in variants with low variant allele fractions, which probably originate from tumour subclones (Fig. 1c, d). Germline variant calls, phased using a haplotype-reference panel, displayed a precision of more than 99% and a sensitivity of 92–98% (Supplementary Note 2).

## Analysis of PCAWG data

The uniformly generated, high-quality set of variant calls across more than 2,500 donors provided the springboard for a series of scientific working groups to explore the biology of cancer. A comprehensive suite of companion papers that describe the analyses and discoveries across these thematic areas is copublished with this paper^4–18^ (Extended Data Table 3).

## Pan-cancer burden of somatic mutations

**Extended Data Fig. 3:**
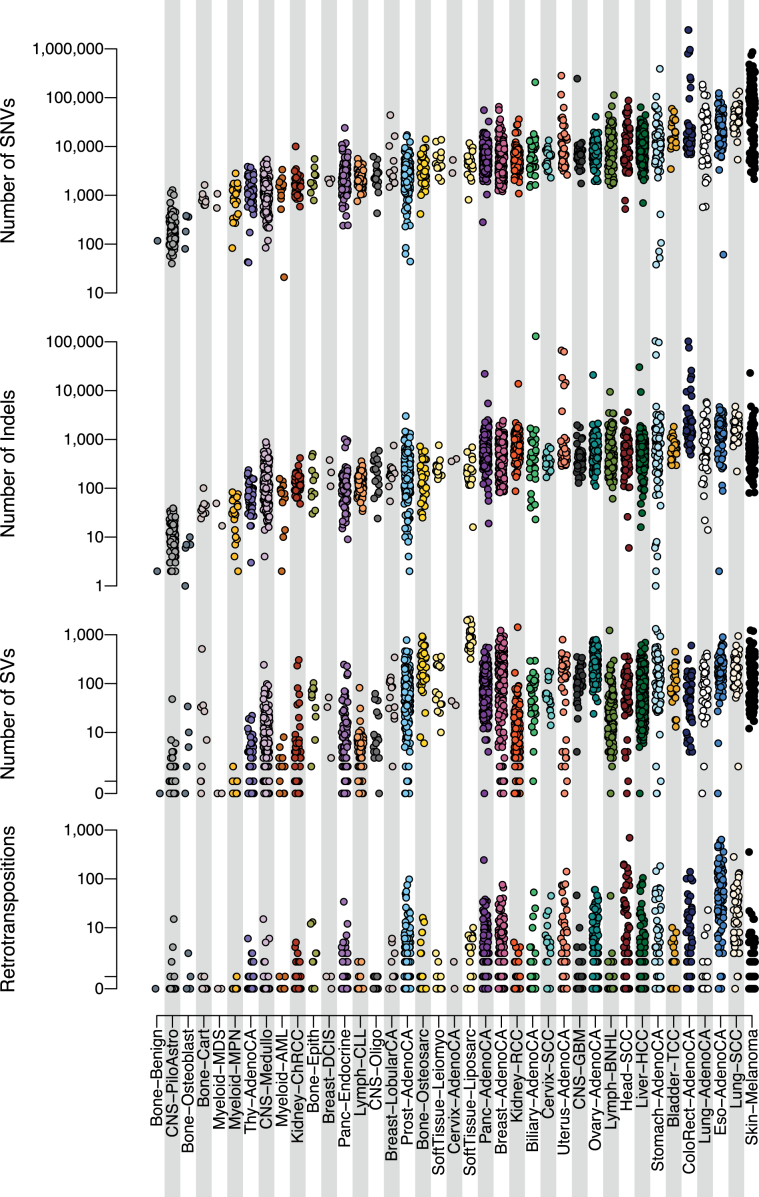
Distribution of numbers of somatic mutations of different classes across tumour types. The *y* axis is on a log scale. The 2,583 donors with the highest quality metrics (white-listed donors) are plotted. SNVs indicate substitutions; indels are taken as insertions or deletions <100 bp in size; retrotranspositions are the combined counts of somatic retrotransposon insertions, transductions and somatic pseudogene insertions.

Across the 2,583 white-listed PCAWG donors, we called 43,778,859 somatic SNVs, 410,123 somatic multinucleotide variants, 2,418,247 somatic indels, 288,416 somatic SVs, 19,166 somatic retrotransposition events and 8,185 de novo mitochondrial DNA mutations (Supplementary Table 1). There was considerable heterogeneity in the burden of somatic mutations across patients and tumour types, with a broad correlation in mutation burden among different classes of somatic variation (Extended Data Fig. 3). Analysed at a per-patient level, this correlation held, even when considering tumours with similar purity and ploidy (Supplementary Fig. 3). Why such correlation should apply on a pan-cancer basis is unclear. It is likely that age has some role, as we observe a correlation between most classes of somatic mutation and age at diagnosis (around 190 SNVs per year, *P* = 0.02; about 22 indels per year, *P* = 5 × 10^−5^; 1.5 SVs per year, *P* < 2 × 10^−16^; linear regression with likelihood ratio tests; Supplementary Fig. 4). Other factors are also likely to contribute to the correlations among classes of somatic mutation, as there is evidence that some DNA-repair defects can cause multiple types of somatic mutation^30^, and a single carcinogen can cause a range of DNA lesions^31^.

## Panorama of driver mutations in cancer

We extracted the subset of somatic mutations in PCAWG tumours that have high confidence to be driver events on the basis of current knowledge. One challenge to pinpointing the specific driver mutations in an individual tumour is that not all point mutations in recurrently mutated cancer-associated genes are drivers^32^. For genomic elements significantly mutated in PCAWG data, we developed a ‘rank-and-cut’ approach to identify the probable drivers (Supplementary Methods 8.1). This approach works by ranking the observed mutations in a given genomic element based on recurrence, estimated functional consequence and expected pattern of drivers in that element. We then estimate the excess burden of somatic mutations in that genomic element above that expected for the background mutation rate, and cut the ranked mutations at this level. Mutations in each element with the highest driver ranking were then assigned as probable drivers; those below the threshold will probably have arisen through chance and were assigned as probable passengers. Improvements to features that are used to rank the mutations and the methods used to measure them will contribute to further development of the rank-and-cut approach.

We also needed to account for the fact that some bona fide cancer genomic elements were not rediscovered in PCAWG data because of low statistical power. We therefore added previously known cancer-associated genes to the discovery set, creating a ‘compendium of mutational driver elements’ (Supplementary Methods 8.2). Then, using stringent rules to nominate driver point mutations that affect these genomic elements on the basis of prior knowledge^33^, we separated probable driver from passenger point mutations. To cover all classes of variant, we also created a compendium of known driver SVs, using analogous rules to identify which somatic CNAs and SVs are most likely to act as drivers in each tumour. For probable pathogenic germline variants, we identified all truncating germline point mutations and SVs that affect high-penetrance germline cancer-associated genes.

**Fig. 2:**
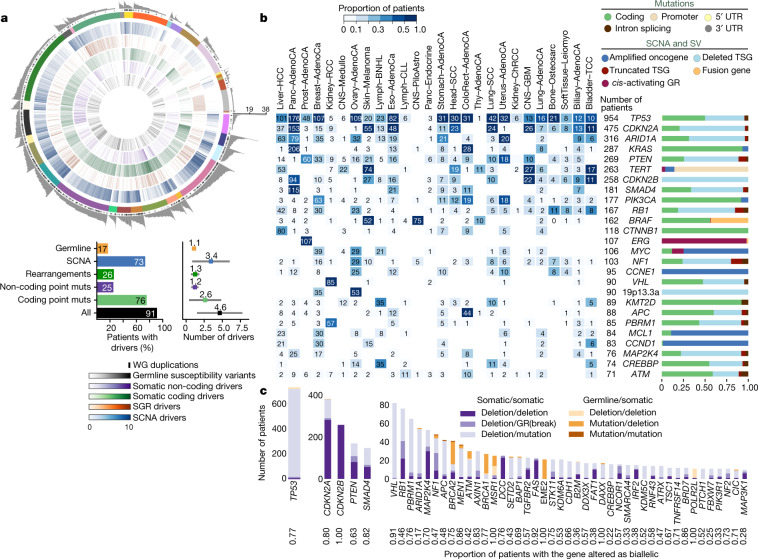
Panorama of driver mutations in PCAWG. **a**, Top, putative driver mutations in PCAWG, represented as a circos plot. Each sector represents a tumour in the cohort. From the periphery to the centre of the plot the concentric rings represent: (1) the total number of driver alterations; (2) the presence of whole-genome (WG) duplication; (3) the tumour type; (4) the number of driver CNAs; (5) the number of driver genomic rearrangements; (6) driver coding point mutations; (7) driver non-coding point mutations; and (8) pathogenic germline variants. Bottom, snapshots of the panorama of driver mutations. The horizontal bar plot (left) represents the proportion of patients with different types of drivers. The dot plot (right) represents the mean number of each type of driver mutation across tumours with at least one event (the square dot) and the standard deviation (grey whiskers), based on *n* = 2,583 patients. **b**, Genomic elements targeted by different types of mutations in the cohort altered in more than 65 tumours. Both germline and somatic variants are included. Left, the heat map shows the recurrence of alterations across cancer types. The colour indicates the proportion of mutated tumours and the number indicates the absolute count of mutated tumours. Right, the proportion of each type of alteration that affects each genomic element. **c**, Tumour-suppressor genes with biallelic inactivation in 10 or more patients. The values included under the gene labels represent the proportions of patients who have biallelic mutations in the gene out of all patients with a somatic mutation in that gene. GR, genomic rearrangement; SCNA, somatic copy-number alteration; SGR, somatic genome rearrangement; TSG, tumour suppressor gene; UTR, untranslated region.

This analysis defined a set of mutations that we could confidently assert, based on current knowledge, drove tumorigenesis in the more than 2,500 tumours of PCAWG. We found that 91% of tumours had at least one identified driver mutation, with an average of 4.6 drivers per tumour identified, showing extensive variation across cancer types (Fig. 2a). For coding point mutations, the average was 2.6 drivers per tumour, similar to numbers estimated in known cancer-associated genes in tumours in the TCGA using analogous approaches^32^.

To address the frequency of non-coding driver point mutations, we combined promoters and enhancers that are known targets of non-coding drivers^34–37^ with those newly discovered in PCAWG data; this is reported in a companion paper^4^. Using this approach, only 13% (785 out of 5,913) of driver point mutations were non-coding in PCAWG. Nonetheless, 25% of PCAWG tumours bear at least one putative non-coding driver point mutation, and one third (237 out of 785) affected the *TERT* promoter (9% of PCAWG tumours). Overall, non-coding driver point mutations are less frequent than coding driver mutations. With the exception of the *TERT* promoter, individual enhancers and promoters are only infrequent targets of driver mutations^4^.

Across tumour types, SVs and point mutations have different relative contributions to tumorigenesis. Driver SVs are more prevalent in breast adenocarcinomas (6.4 ± 3.7 SVs (mean ± s.d.) compared with 2.2 ± 1.3 point mutations; *P* < 1 × 10^−16^, Mann–Whitney *U*-test) and ovary adenocarcinomas (5.8 ± 2.6 SVs compared with 1.9 ± 1.0 point mutations; *P* < 1 × 10^−16^), whereas driver point mutations have a larger contribution in colorectal adenocarcinomas (2.4 ± 1.4 SVs compared with 7.4 ± 7.0 point mutations; *P* = 4 × 10^−10^) and mature B cell lymphomas (2.2 ± 1.3 SVs compared with 6 ± 3.8 point mutations; *P* < 1 × 10^−16^), as previously shown^38^. Across tumour types, there are differences in which classes of mutation affect a given genomic element (Fig. 2b).

We confirmed that many driver mutations that affect tumour-suppressor genes are two-hit inactivation events (Fig. 2c). For example, of the 954 tumours in the cohort with driver mutations in *TP53*, 736 (77%) had both alleles mutated, 96% of which (707 out of 736) combined a somatic point mutation that affected one allele with somatic deletion of the other allele. Overall, 17% of patients had rare germline protein-truncating variants (PTVs) in cancer-predisposition genes^39^, DNA-damage response genes^40^ and somatic driver genes. Biallelic inactivation due to somatic alteration on top of a germline PTV was observed in 4.5% of patients overall, with 81% of these affecting known cancer-predisposition genes (such as *BRCA1*, *BRCA2* and *ATM*).

## PCAWG tumours with no apparent drivers

**Extended Data Fig. 4:**
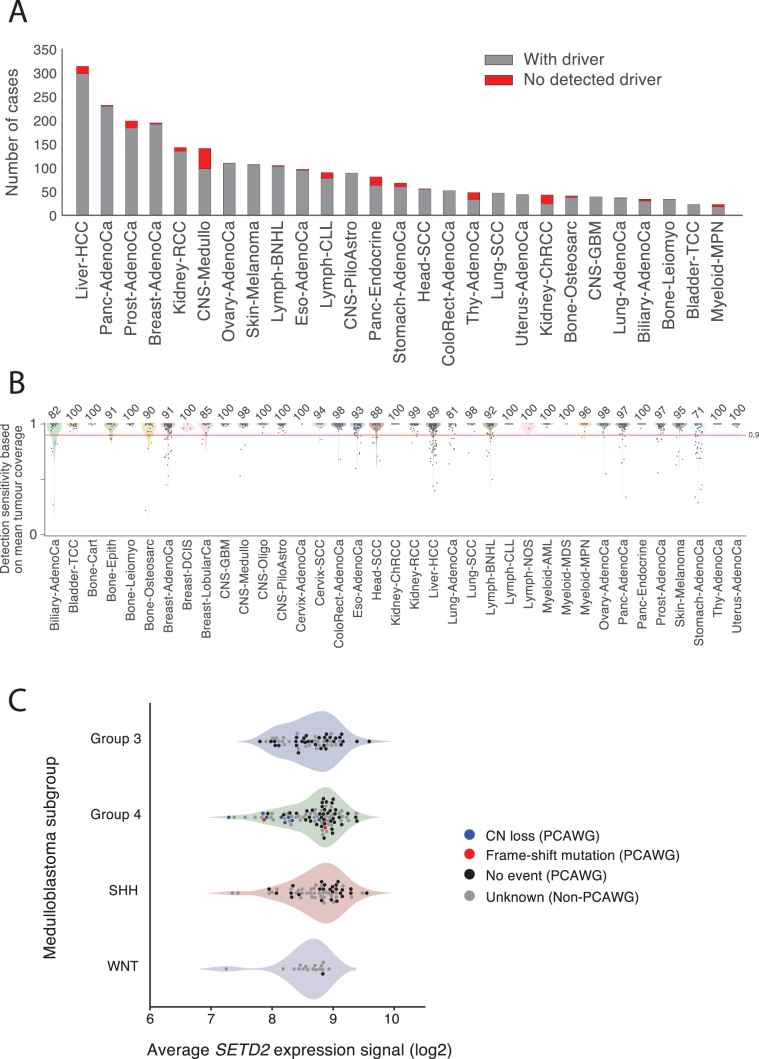
Patients with no detected driver mutations in PCAWG. **a**, Number (red) of patients without detected driver mutations distributed across the different tumour types studied. **b**, Estimated sensitivity for detecting somatic point mutations genome-wide across tumour types (total sample size: *n* = 2,583 patients). Each point represents the estimate for a single patient, layered on violin plots that show the estimated density distribution of sensitivity values for that tumour type (the width proportional is to density). **c**, *SETD2* expression levels across different medulloblastoma subtypes. Points represent individual patients, coloured by whether the gene exhibited focal copy number (CN) loss or a truncating point mutation, or was the wild-type gene. The coloured areas are violin plots showing the estimated density distribution of expression values for that medulloblastoma subtype.

Although more than 90% of PCAWG cases had identified drivers, we found none in 181 tumours (Extended Data Fig. 4a). Reasons for missing drivers have not yet been systematically evaluated in a pan-cancer cohort, and could arise from either technical or biological causes.

**Fig. 3:**
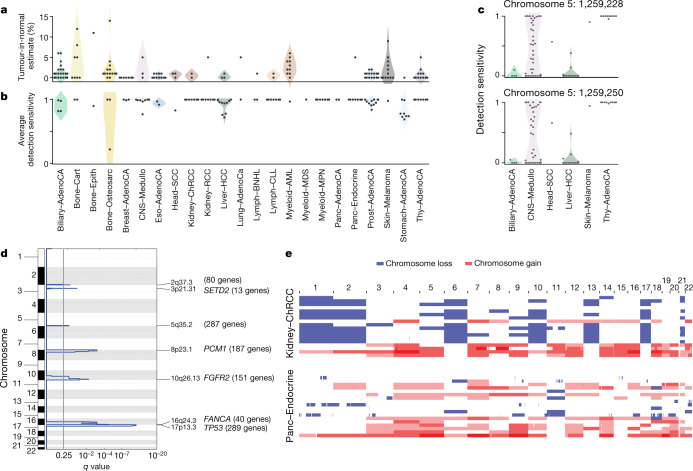
Analysis of patients with no detected driver mutations. **a**, Individual estimates of the percentage of tumour-in-normal contamination across patients with no driver mutations in PCAWG (*n* = 181). No data were available for myelodysplastic syndromes and acute myeloid leukaemia. Points represent estimates for individual patients, and the coloured areas are estimated density distributions (violin plots). Abbreviations of the tumour types are defined in Extended Data Table 1. **b**, Average detection sensitivity by tumour type for tumours without known drivers (*n* = 181). Each dot represents a given sample and is the average sensitivity of detecting clonal substitutions across the genome, taking into account purity and ploidy. Coloured areas are estimated density distributions, shown for cohorts with at least five cases. **c**, Detection sensitivity for *TERT* promoter hotspots in tumour types in which *TERT* is frequently mutated. Coloured areas are estimated density distributions. **d**, Significant copy-number losses identified by two-sided hypothesis testing using GISTIC2.0, corrected for multiple-hypothesis testing. Numbers in parentheses indicate the number of genes in significant regions when analysing medulloblastomas without known drivers (*n* = 42). Significant regions with known cancer-associated genes are labelled with the representative cancer-associated gene. **e**, Aneuploidy in chromophobe renal cell carcinomas and pancreatic neuroendocrine tumours without known drivers. Patients are ordered on the *y* axis by tumour type and then by presence of whole-genome duplication (bottom) or not (top).

Technical explanations could include poor-quality samples, inadequate sequencing or failures in the bioinformatic algorithms used. We assessed the quality of the samples and found that 4 of the 181 cases with no known drivers had more than 5% tumour DNA contamination in their matched normal sample (Fig. 3a). Using an algorithm designed to correct for this contamination^41^, we identified previously missed mutations in genes relevant to the respective cancer types. Similarly, if the fraction of tumour cells in the cancer sample is low through stromal contamination, the detection of driver mutations can be impaired. Most tumours with no known drivers had an average power to detect mutations close to 100%; however, a few had power in the 70–90% range (Fig. 3b and Extended Data Fig. 4b). Even in adequately sequenced genomes, lack of read depth at specific driver loci can impair mutation detection. For example, only around 50% of PCAWG tumours had sufficient coverage to call a mutation (≥90% power) at the two *TERT* promoter hotspots, probably because the high GC content of this region causes biased coverage (Fig. 3c). In fact, 6 hepatocellular carcinomas and 2 biliary cholangiocarcinomas among the 181 cases with no known drivers actually did contain *TERT* mutations, which were discovered after deep targeted sequencing^42^.

Finally, technical reasons for missing driver mutations include failures in the bioinformatic algorithms. This affected 35 myeloproliferative neoplasms in PCAWG, in which the *JAK2*^*V617F*^ driver mutation should have been called. Our somatic variant-calling algorithms rely on ‘panels of normals’, typically from blood samples, to remove recurrent sequencing artefacts. As 2–5% of healthy individuals carry occult haematopoietic clones^43^, recurrent driver mutations in these clones can enter panels of normals.

With regard to biological causes, tumours may be driven by mutations in cancer-associated genes that are not yet described for that tumour type. Using driver discovery algorithms on tumours with no known drivers, no individual genes reached significance for point mutations. However, we identified a recurrent CNA that spanned *SETD2* in medulloblastomas that lacked known drivers (Fig. 3d), indicating that restricting hypothesis testing to missing-driver cases can improve power if undiscovered genes are enriched in such tumours. Inactivation of *SETD2* in medulloblastoma significantly decreased gene expression (*P* = 0.002) (Extended Data Fig. 4c). Notably, *SETD2* mutations occurred exclusively in medulloblastoma group-4 tumours (*P* < 1 × 10^−4^). Group-4 medulloblastomas are known for frequent mutations in other chromatin-modifying genes^44^, and our results suggest that *SETD2* loss of function is an additional driver that affects chromatin regulators in this subgroup.

Two tumour types had a surprisingly high fraction of patients without identified driver mutations: chromophobe renal cell carcinoma (44%; 19 out of 43) and pancreatic neuroendocrine cancers (22%; 18 out of 81) (Extended Data Fig. 4a). A notable feature of the missing-driver cases in both tumour types was a remarkably consistent profile of chromosomal aneuploidy—patterns that have previously been reported^45,46^ (Fig. 3e). The absence of other identified driver mutations in these patients raises the possibility that certain combinations of whole-chromosome gains and losses may be sufficient to initiate a cancer in the absence of more-targeted driver events such as point mutations or fusion genes of focal CNAs.

Even after accounting for technical issues and novel drivers, 5.3% of PCAWG tumours still had no identifiable driver events. In a research setting, in which we are interested in drawing conclusions about populations of patients, the consequences of technical issues that affect occasional samples will be mitigated by sample size. In a clinical setting, in which we are interested in the driver mutations in a specific patient, these issues become substantially more important. Careful and critical appraisal of the whole pipeline—including sample acquisition, genome sequencing, mapping, variant calling and driver annotation, as done here—should be required for laboratories that offer clinical sequencing of cancer genomes.

## Patterns of clustered mutations and SVs

**Fig. 4:**
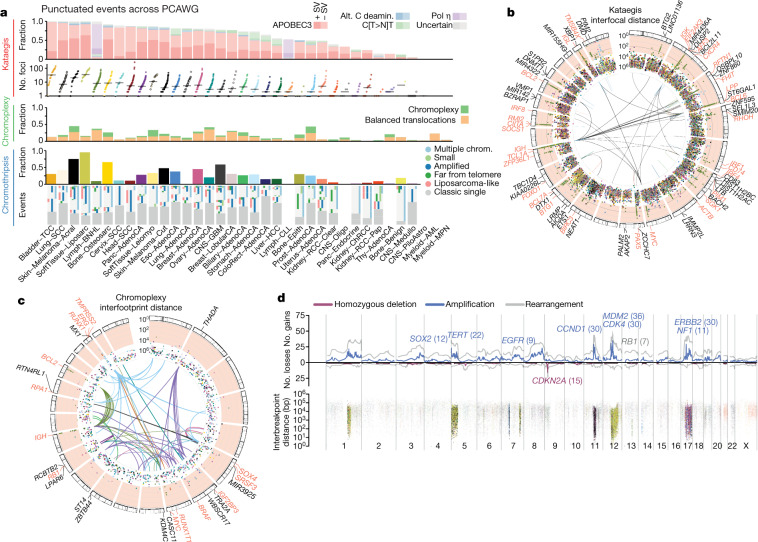
Patterns of clustered mutational processes in PCAWG. **a**, Kataegis. Top, prevalence of different types of kataegis and their association with SVs (≤1 kb from the focus). Bottom, the distribution of the number of foci of kataegis per sample. Chromoplexy. Prevalence of chromoplexy across cancer types, subdivided into balanced translocations and more complex events. Chromothripsis. Top, frequency of chromothripsis across cancer types. Bottom, for each cancer type a column is shown, in which each row is a chromothripsis region represented by five coloured rectangles relating to its categorization. **b**, Circos rainfall plot showing the distances between consecutive kataegis events across PCAWG compared with their genomic position. Lymphoid tumours (khaki, B cell non-Hodgkin’s lymphoma; orange, chronic lymphocytic leukaemia) have hypermutation hot spots (≥3 foci with distance ≤1 kb; pale red zone), many of which are near known cancer-associated genes (red annotations) and have associated SVs (≤10 kb from the focus; shown as arcs in the centre). **c**, Circos rainfall plot as in **b** that shows the distance versus the position of consecutive chromoplexy and reciprocal translocation footprints across PCAWG. Lymphoid, prostate and thyroid cancers exhibit recurrent events (≥2 footprints with distance ≤10 kb; pale red zone) that are likely to be driver SVs and are annotated with nearby genes and associated SVs, which are shown as bold and thin arcs for chromoplexy and reciprocal translocations, respectively (colours as in **a**). **d**, Effect of chromothripsis along the genome and involvement of PCAWG driver genes. Top, number of chromothripsis-induced gains or losses (grey) and amplifications (blue) or deletions (red). Within the identified chromothripsis regions, selected recurrently rearranged (light grey), amplified (blue) and homozygously deleted (magenta) driver genes are indicated. Bottom, interbreakpoint distance between all subsequent breakpoints within chromothripsis regions across cancer types, coloured by cancer type. Regions with an average interbreakpoint distance <10 kb are highlighted. C[T>N]T, kataegis with a pattern of thymine mutations in a Cp TpT context.

**Extended Data Fig. 5:**
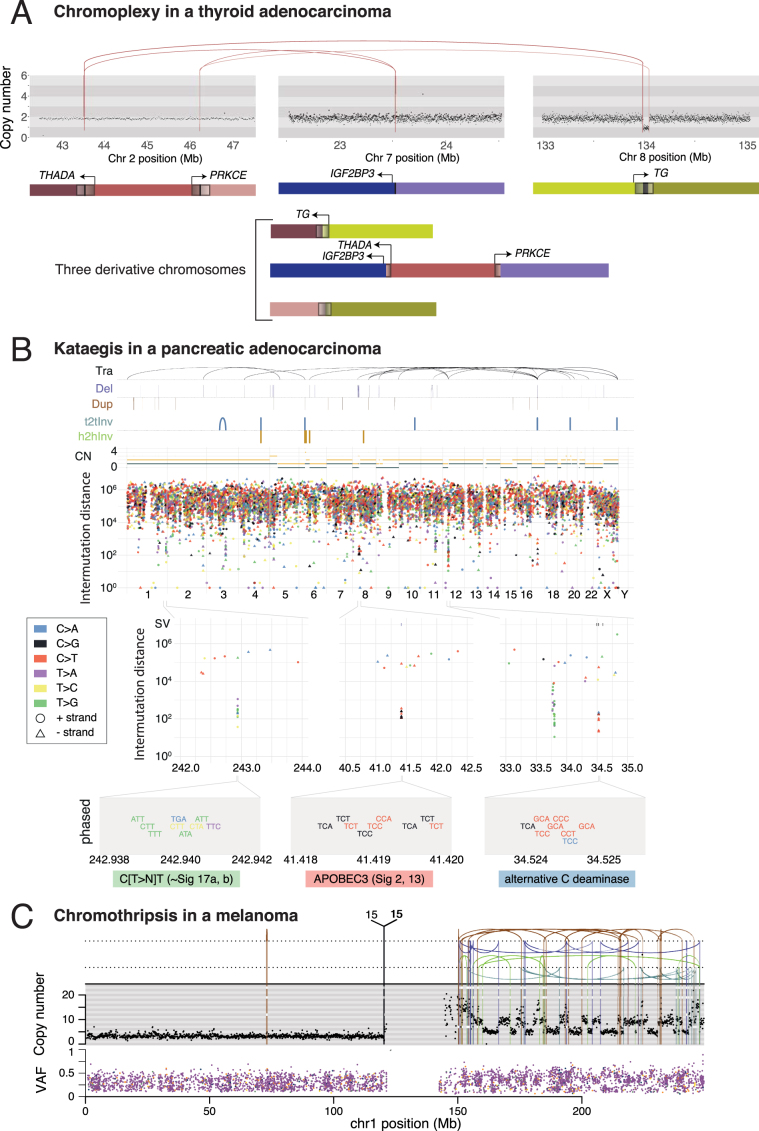
Examples of clustered mutational processes. **a**, Chromoplexy example in a thyroid adenocarcinoma. Genes at the breakpoints are schematically depicted in their normal genomic context and again in the reconstructed derivative chromosomes below. **b**, Distinct kataegis signatures in the genome of a pancreatic adenocarcinoma sample. SVs and their classification are shown above the main rainfall plot, as well as the total and minor allele copy number. Tra, translocation; del, deletion; dup, duplication; t2tInv, tail-to-tail inversion; h2hInv, head-to-head inversion. Magnifications of the three foci on chromosomes 1, 8 and 12, respectively, highlight distinct manifestations of kataegis. Left, a novel process similar to signature 17 with T > N mutations at CT or TT dinucleotides. Middle, the prototypical APOBEC3A/B type with C > T (signature 2) and/or C>G/A (signature 13) substitutions at TpC. Right, an alternative cytidine deaminase(s) with a preference for substitutions at C/GpC. Most of the SNVs in each of these foci can be phased to the same allele and no evidence of anti-phasing is observed. **c**, Example of a chromothripsis event in a melanoma. The black points (top) represent copy-number estimates from individual genomic bins, with SVs shown as coloured arcs (translocation in black, deletion in purple, duplication in brown, tail-to-tail inversion in cyan, head-to-head inversion in green) that mostly demarcate copy-number changes. The mate chromosomes are displayed above translocations. Bottom, the variant allele fractions of somatic mutations distributed along the relevant chromosomal region.

Some somatic mutational processes generate multiple mutations in a single catastrophic event, typically clustered in genomic space, leading to substantial reconfiguration of the genome. Three such processes have previously been described: (1) chromoplexy, in which repair of co-occurring double-stranded DNA breaks—typically on different chromosomes—results in shuffled chains of rearrangements^47,48^ (Extended Data Fig. 5a); (2) kataegis, a focal hypermutation process that leads to locally clustered nucleotide substitutions, biased towards a single DNA strand^49–51^ (Extended Data Fig. 5b); and (3) chromothripsis, in which tens to hundreds of DNA breaks occur simultaneously, clustered on one or a few chromosomes, with near-random stitching together of the resulting fragments^52–55^ (Extended Data Fig. 5c). We characterized the PCAWG genomes for these three processes (Fig. 4).

Chromoplexy events and reciprocal translocations were identified in 467 (17.8%) samples (Fig. 4a, c). Chromoplexy was prominent in prostate adenocarcinoma and lymphoid malignancies, as previously described^47,48^, and—unexpectedly—thyroid adenocarcinoma. Different genomic loci were recurrently rearranged by chromoplexy across the three tumour types, mediated by positive selection for particular fusion genes or enhancer-hijacking events. Of 13 fusion genes or enhancer hijacking events in 48 thyroid adenocarcinomas, at least 4 (31%) were caused by chromoplexy, with a further 4 (31%) part of complexes that contained chromoplexy footprints (Extended Data Fig. 5a). These events generated fusion genes that involved *RET* (two cases) and *NTRK3* (one case)^56^, and the juxtaposition of the oncogene *IGF2BP3* with regulatory elements from highly expressed genes (five cases).

Kataegis events were found in 60.5% of all cancers, with particularly high abundance in lung squamous cell carcinoma, bladder cancer, acral melanoma and sarcomas (Fig. 4a, b). Typically, kataegis comprises C > N mutations in a TpC context, which are probably caused by APOBEC activity^49–51^, although a T > N conversion in a **T**pT or Cp**T** process (the affected T is highlighted in bold) attributed to error-prone polymerases has recently been described^57^. The APOBEC signature accounted for 81.7% of kataegis events and correlated positively with *APOBEC3B* expression levels, somatic SV burden and age at diagnosis (Supplementary Fig. 5). Furthermore, 5.7% of kataegis events involved the T > N error-prone polymerase signature and 2.3% of events, most notably in sarcomas, showed cytidine deamination in an alternative Gp**C** or Cp**C** context.

Kataegis events were frequently associated with somatic SV breakpoints (Fig. 4a and Supplementary Fig. 6a), as previously described^50,51^. Deletions and complex rearrangements were most-strongly associated with kataegis, whereas tandem duplications and other simple SV classes were only infrequently associated (Supplementary Fig. 6b). Kataegis inducing predominantly T > N mutations in Cp**T**pT context was enriched near deletions, specifically those in the 10–25-kilobase (kb) range (Supplementary Fig. 6c).

**Extended Data Fig. 6:**
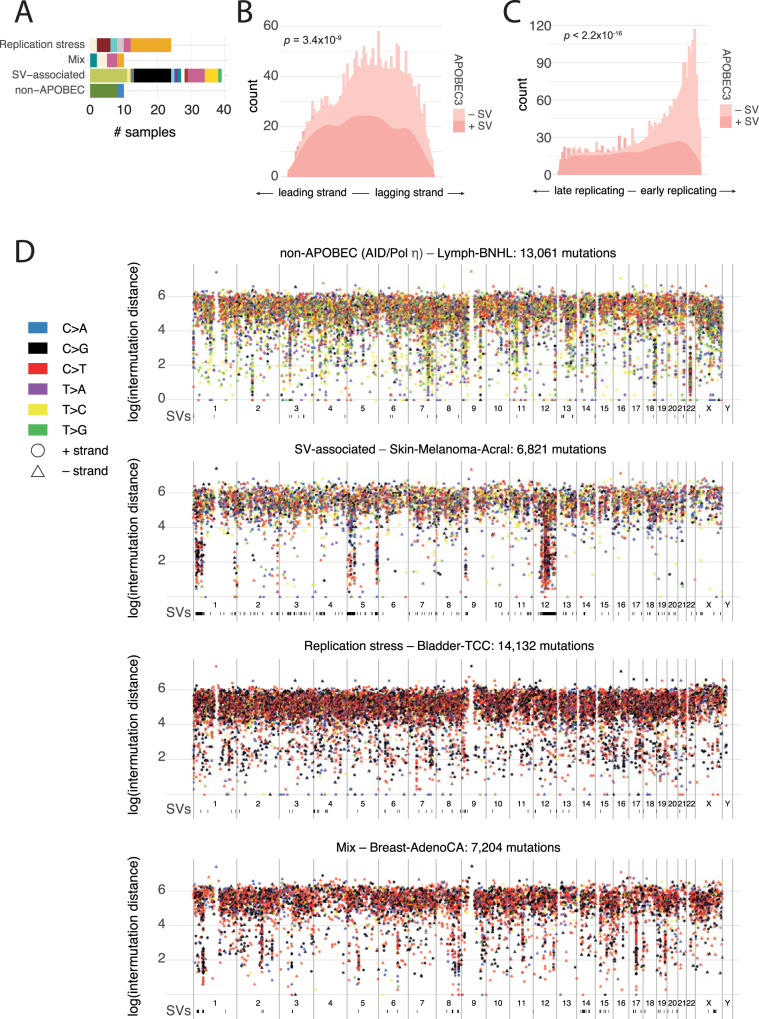
Patterns of intense kataegis. **a**, Distribution of the tumour types (colour-coded as in Extended Data Fig. 3) of the samples in the top 5% of kataegis intensity in each of the four identified genome-wide patterns: non-APOBEC, replication stress, rearrangement-associated and the combination of the last two. **b**, **c**, Distribution of leading/lagging strand (**b**) and replication timing bias (**c**) for rearrangement-(in)dependent APOBEC kataegis, based on *n* = 2,583 tumours. *P* values were derived using a two-sided Mann–Whittney *U*-test. **d**, Example rainfall plots for each of the four identified kataegis patterns.

Samples with extreme kataegis burden (more than 30 foci) comprise four types of focal hypermutation (Extended Data Fig. 6): (1) off-target somatic hypermutation and foci of T > N at Cp**T**pT, found in B cell non-Hodgkin lymphoma and oesophageal adenocarcinomas, respectively; (2) APOBEC kataegis associated with complex rearrangements, notably found in sarcoma and melanoma; (3) rearrangement-independent APOBEC kataegis on the lagging strand and in early-replicating regions, mainly found in bladder and head and neck cancer; and (4) a mix of the last two types. Kataegis only occasionally led to driver mutations (Supplementary Table 5).

**Extended Data Fig. 7:**
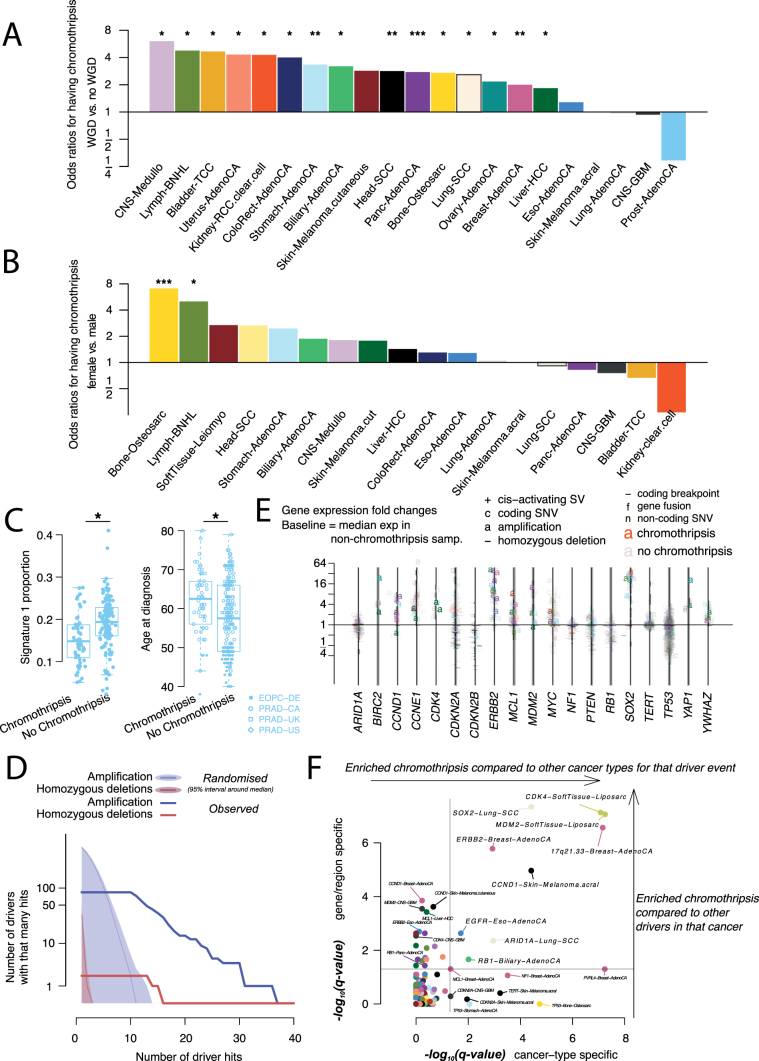
Association of chromothripsis with covariates and driver events. **a**, Odds ratios per cancer type of containing chromothripsis in whole-genome duplicated versus diploid samples (*n* = 2,583 patients). ***q < 0.001; **q < 0.01; *q < 0.05. Two-sided hypothesis testing was performed using Fisher–Boschloo tests, corrected for multiple-hypothesis testing. **b**, Same as **a** for female versus male. **c**, Proportion of mutations explained by single-base substitution signature 1 and age at diagnosis in prostate cancer samples (*n* = 210 patients) with or without chromothripsis (*q* < 0.05). The early-onset prostate cancer project drives the signal and was sequenced at lower depth. For the box-and-whisker plots, the box denotes the interquartile range, with the median marked as a horizontal line. The whiskers extend as far as the range or 1.5× the interquartile range, whichever is less. Two-sided hypothesis testing was performed using Mann–Whitney *U*-tests. **d**, Counts of co-occurrence of chromothripsis with amplification (blue) and homozygous deletions (red) in driver regions: observed (thick line) versus randomized (shaded area and thin line). The cumulative number of drivers that were hit is plotted as a function of the number of times those drivers were hit. **e**, For each sample in which chromothripsis coincided with a driver event in those genes, we show the fold change in gene expression compared to the median expression of the gene in non-chromothripsis samples of the same cancer type, coloured by cancer type and shaped by the type of driver event. We show with added transparency the fold changes calculated the same way for samples with driver mutations hitting the same driver genes, but that had no evidence of chromothripsis. Analysis is based on *n* = 1,222 patients with RNA-sequencing data. **f**, Enrichment of co-occurrence of chromothripsis with driver events. The *x* axis shows the association of chromothripsis with a driver in a given cancer type compared with its rate of association with that driver in all other cancer types. The *y* axis shows the association of chromothripsis with a driver in a given cancer type compared with its rate of association with all other drivers in that type. Exact binomial tests are used and *P* values are corrected for multiple testing according to the Benjamini–Hochberg method.

We identified chromothripsis in 587 samples (22.3%), most frequently among sarcoma, glioblastoma, lung squamous cell carcinoma, melanoma and breast adenocarcinoma^18^. Chromothripsis increased with whole-genome duplications in most cancer types (Extended Data Fig. 7a), as previously shown in medulloblastoma^58^. The most recurrently associated driver was *TP53*^52^ (pan-cancer odds ratio = 3.22; pan-cancer *P* = 8.3 × 10^−35^; *q* < 0.05 in breast lobular (odds ratio = 13), colorectal (odds ratio = 25), prostate (odds ratio = 2.6) and hepatocellular (odds ratio = 3.9) cancers; Fisher–Boschloo tests). In two cancer types (osteosarcoma and B cell lymphoma), women had a higher incidence of chromothripsis than men (Extended Data Fig. 7b). In prostate cancer, we observed a higher incidence of chromothripsis in patients with late-onset than early-onset disease^59^ (Extended Data Fig. 7c).

Chromothripsis regions coincided with 3.6% of all identified drivers in PCAWG and around 7% of copy-number drivers (Fig. 4d). These proportions are considerably enriched compared to expectation if selection were not acting on these events (Extended Data Fig. 7d). The majority of coinciding driver events were amplifications (58%), followed by homozygous deletions (34%) and SVs within genes or promoter regions (8%). We frequently observed a ≥2-fold increase or decrease in expression of amplified or deleted drivers, respectively, when these loci were part of a chromothripsis event, compared with samples without chromothripsis (Extended Data Fig. 7e).

Chromothripsis manifested in diverse patterns and frequencies across tumour types, which we categorized on the basis of five characteristics (Fig. 4a). In liposarcoma, for example, chromothripsis events often involved multiple chromosomes, with universal *MDM2* amplification^60^ and co-amplification of *TERT* in 4 of 19 cases (Fig. 4d). By contrast, in glioblastoma the events tended to affect a smaller region on a single chromosome that was distant from the telomere, resulting in focal amplification of *EGFR* and *MDM2* and loss of *CDKN2A*. Acral melanomas frequently exhibited *CCND1* amplification, and lung squamous cell carcinomas *SOX2* amplifications. In both cases, these drivers were more-frequently altered by chromothripsis compared with other drivers in the same cancer type and to other cancer types for the same driver (Fig. 4d and Extended Data Fig. 7f). Finally, in chromophobe renal cell carcinoma, chromothripsis nearly always affected chromosome 5 (Supplementary Fig. 7): these samples had breakpoints immediately adjacent to *TERT*, increasing *TERT* expression by 80-fold on average compared with samples without rearrangements (*P* = 0.0004; Mann–Whitney *U*-test).

## Timing clustered mutations in evolution

An unanswered question for clustered mutational processes is whether they occur early or late in cancer evolution. To address this, we used molecular clocks to define broad epochs in the life history of each tumour^49,61^. One transition point is between clonal and subclonal mutations: clonal mutations occurred before, and subclonal mutations after, the emergence of the most-recent common ancestor. In regions with copy-number gains, molecular time can be further divided according to whether mutations preceded the copy-number gain (and were themselves duplicated) or occurred after the gain (and therefore present on only one chromosomal copy)^7^.

**Fig. 5:**
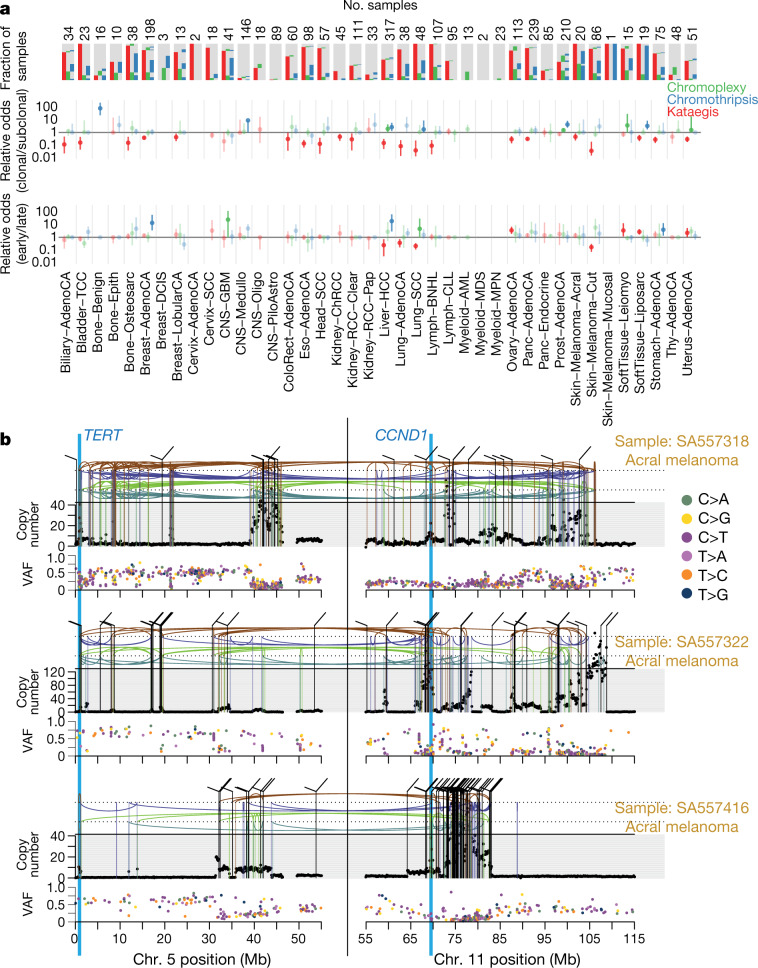
Timing of clustered events in PCAWG. **a**, Extent and timing of chromothripsis, kataegis and chromoplexy across PCAWG. Top, stacked bar charts illustrate co-occurrence of chromothripsis, kataegis and chromoplexy in the samples. Middle, relative odds of clustered events being clonal or subclonal are shown with bootstrapped 95% confidence intervals. Point estimates are highlighted when they do not overlap odds of 1:1. Bottom, relative odds of the events being early or late clonal are shown as above. Sample sizes (number of patients) are shown across the top. **b**, Three representative patients with acral melanoma and chromothripsis-induced amplification that simultaneously affects *TERT* and *CCND1*. The black points (top) represent sequence coverage from individual genomic bins, with SVs shown as coloured arcs (translocation in black, deletion in purple, duplication in brown, tail-to-tail inversion in cyan and head-to-head inversion in green). Bottom, the variant allele fractions of somatic point mutations.

**Extended Data Fig. 8:**
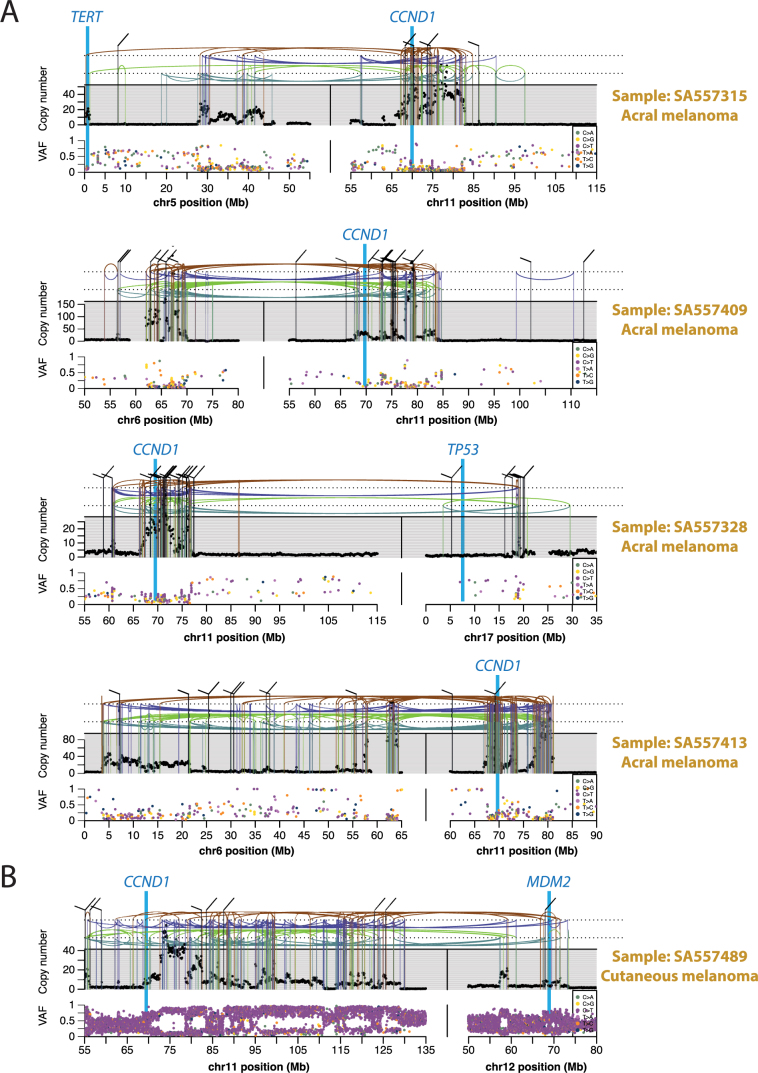
Further examples of chromothripsis-induced amplification targeting multiple cancer-associated genes simultaneously in melanoma. **a**, Examples of amplifications that occurred early in the development of melanoma. The black points (top) represent copy-number estimates from individual genomic bins, with SVs shown as coloured arcs (translocation in black, deletion in purple, duplication in brown, tail-to-tail inversion in cyan and head-to-head inversion in green) that mostly demarcate copy-number changes. Bottom, the variant allele fractions of SNVs distributed along the relevant chromosomal region. The paucity of somatic mutations at high variant allele fractions in the most-heavily amplified regions indicates that these amplifications began very early in tumour evolution, before the lineage had had opportunity to acquire many SNVs. **b**, Example of an amplification that occurred late in melanoma development. The large numbers of somatic mutations at high variant allele fractions in the most-heavily amplified regions indicate that these amplifications began late in tumour evolution, after the lineage had already acquired many SNVs.

Chromothripsis tended to have greater relative odds of being clonal than subclonal, suggesting that it occurs early in cancer evolution, especially in liposarcomas, prostate adenocarcinoma and squamous cell lung cancer (Fig. 5a). As previously reported, chromothripsis was especially common in melanomas^62^. We identified 89 separate chromothripsis events that affected 66 melanomas (61%); 47 out of 89 events affected genes known to be recurrently altered in melanoma^63^ (Supplementary Table 6). Involvement of a region on chromosome 11 that includes the cell-cycle regulator *CCND1* occurred in 21 cases (10 out of 86 cutaneous, and 11 out of 21 acral or mucosal melanomas), typically combining chromothripsis with amplification (19 out of 21 cases) (Extended Data Fig. 8). Co-involvement of other cancer-associated genes in the same chromothripsis event was also frequent, including *TERT* (five cases), *CDKN2A* (three cases), *TP53* (two cases) and *MYC* (two cases) (Fig. 5b). In these co-amplifications, a chromothripsis event involving multiple chromosomes initiated the process, creating a derivative chromosome in which hundreds of fragments were stitched together in a near-random order (Fig. 5b). This derivative then rearranged further, leading to massive co-amplification of the multiple target oncogenes together with regions located nearby on the derivative chromosome.

**Extended Data Fig. 9:**
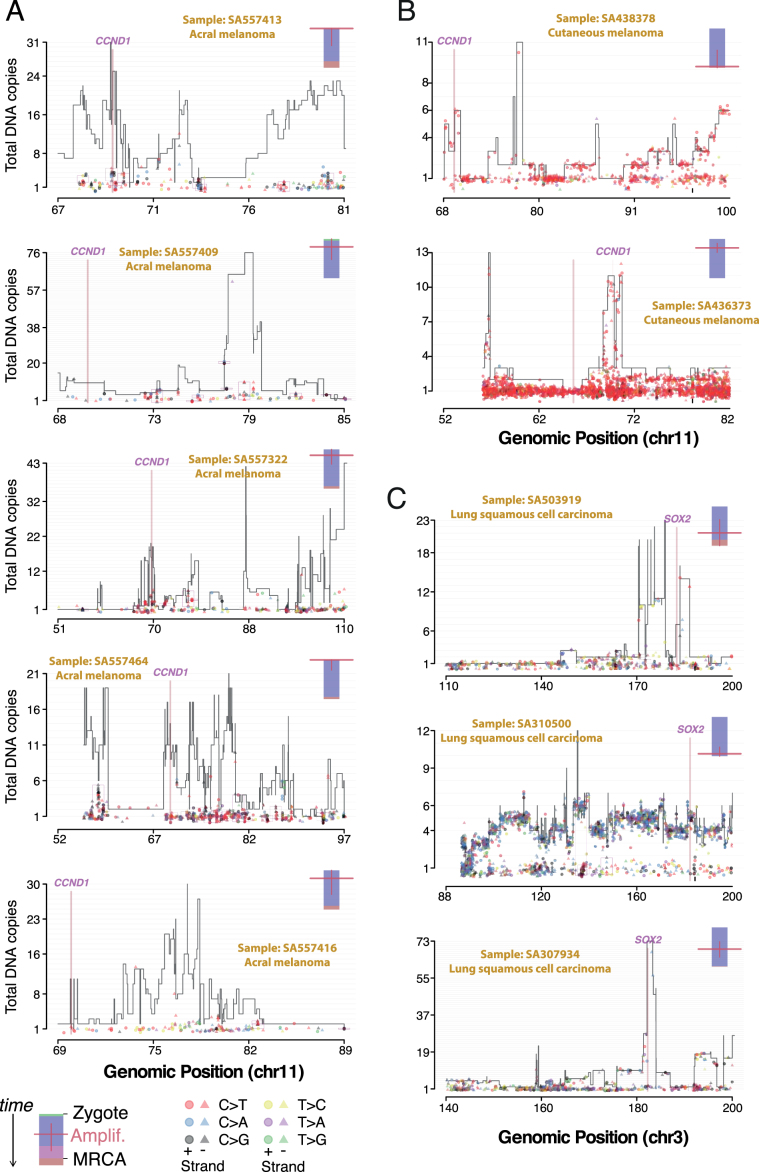
Timing the amplifications after chromothripsis in molecular time for 10 representative cases. **a**, Copy-number plot of chromothriptic regions categorized as ‘liposarc-like’ in five acral melanomas with *CCND1* amplification. Segments indicate the copy number of the major allele. Points represent SNV multiplicities, that is, the estimated number of copies carrying each SNV, coloured by base change and shaped by strand. Small vertical arrows link SNVs to their corresponding copy-number segment. Kataegis foci are shown within black boxes and show typical strand specificities (all triangles or all circles), similar multiplicities and base changes of signatures 2 and 13 (red and black, respectively). A coloured bar (top right) represents the molecular timing of the amplification (red bar; high is early, low is late) and is coloured by the fraction of total SNVs assigned to the following timing categories: clonal [early], clonal mutations that occurred before duplications involving the relevant chromosome (including whole-genome duplications); clonal [late], clonal mutations that occurred after such duplications; and clonal [NA], mutations that occurred when no duplication was observed. **b**, Same as **a** in two cutaneous melanomas, one shows early amplification, the other late amplification. **c**, Same as **a**, **b**, for three lung squamous cell carcinomas and late amplification of *SOX2*.

In these cases of amplified chromothripsis, we can use the inferred number of copies bearing each SNV to time the amplification process. SNVs present on the chromosome before amplification will themselves be amplified and are therefore reported in a high fraction of sequence reads (Fig. 5b and Extended Data Fig. 8). By contrast, late SNVs that occur after the amplification has concluded will be present on only one chromosome copy out of many, and thus have a low variant allele fraction. Regions of *CCND1* amplification had few—sometimes zero—mutations at high variant allele fraction in acral melanomas, in contrast to later *CCND1* amplifications in cutaneous melanomas, in which hundreds to thousands of mutations typically predated amplification (Fig. 5b and Extended Data Fig. 9a, b). Thus, both chromothripsis and the subsequent amplification generally occurred very early during the evolution of acral melanoma. By comparison, in lung squamous cell carcinomas, similar patterns of chromothripsis followed by *SOX2* amplification are characterized by many amplified SNVs, suggesting a later event in the evolution of these cancers (Extended Data Fig. 9c).

Notably, in cancer types in which the mutational load was sufficiently high, we could detect a larger-than-expected number of SNVs on an intermediate number of DNA copies, suggesting that they appeared during the amplification process (Supplementary Fig. 8).

## Germline effects on somatic mutations

**Fig. 6:**
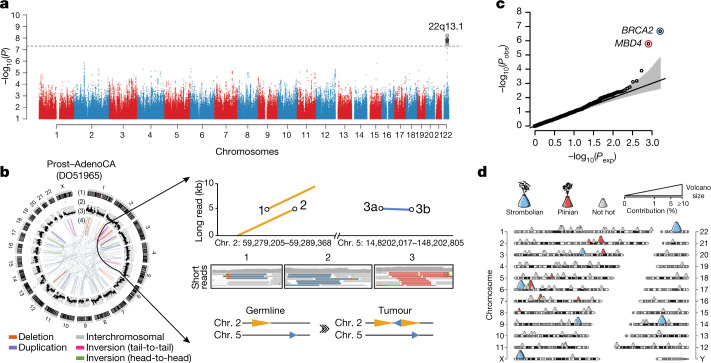
Germline determinants of the somatic mutation landscape. **a**, Association between common (MAF > 5%) germline variants and somatic APOBEC3B-like mutagenesis in individuals of European ancestry (*n* = 1,201). Two-sided hypothesis testing was performed with PLINK v.1.9. To mitigate multiple-hypothesis testing, the significance threshold was set to genome-wide significance (*P* < 5 × 10^−8^). **b**, Templated insertion SVs in a *BRCA1*-associated prostate cancer. Left, chromosome bands (1); SVs ≤ 10 megabases (Mb) (2); 1-kb read depth corrected to copy number 0–6 (3); inter- and intrachromosomal SVs > 10 Mb (4). Right, a complex somatic SV composed of a 2.2-kb tandem duplication on chromosome 2 together with a 232-base-pair (bp) inverted templated insertion SV that is derived from chromosome 5 and inserted inbetween the tandem duplication (bottom). Consensus sequence alignment of locally assembled Oxford Nanopore Technologies long sequencing reads to chromosomes 2 and 5 of the human reference genome (top). Breakpoints are circled and marked as 1 (beginning of tandem duplication), 2 (end of tandem duplication) or 3 (inverted templated insertion). For each breakpoint, the middle panel shows Illumina short reads at SV breakpoints. **c**, Association between rare germline PTVs (MAF < 0.5%) and somatic CpG mutagenesis (approximately with signature 1) in individuals of European ancestry (*n* = 1,201). Genes highlighted in blue or red were associated with lower or higher somatic mutation rates. Two-sided hypothesis testing was performed using linear-regression models with sex, age at diagnosis and cancer project as variables. To mitigate multiple-hypothesis testing, the significance threshold was set to exome-wide significance (*P* < 2.5 × 10^−6^). The black line represents the identity line that would be followed if the observed *P* values followed the null expectation; the shaded area shows the 95% confidence intervals. **d**, Catalogue of polymorphic germline L1 source elements that are active in cancer. The chromosomal map shows germline source L1 elements as volcano symbols. Each volcano is colour-coded according to the type of source L1 activity. The contribution of each source locus (expressed as a percentage) to the total number of transductions identified in PCAWG tumours is represented as a gradient of volcano size, with top contributing elements exhibiting larger sizes.

**Extended Data Fig. 10:**
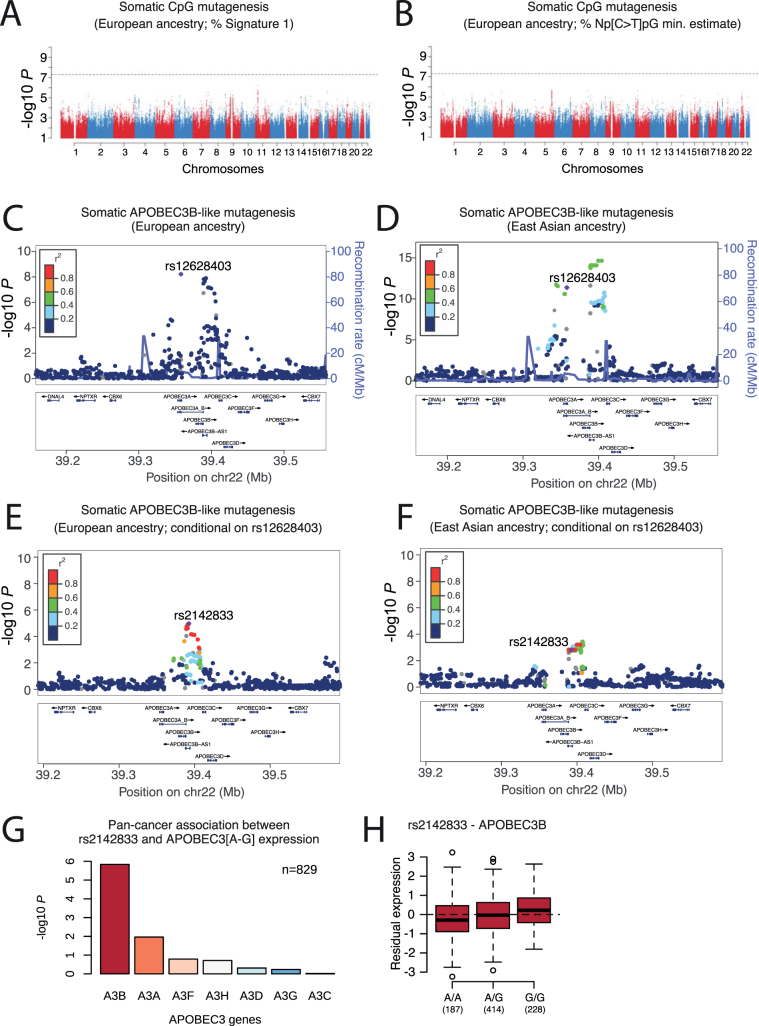
Association between common germline variants and endogenous mutational processes. Genome-wide association of somatic CpG mutagenesis in individuals of European ancestry (*n* = 1,201 patients) based on mutational signature analysis (**a**) and NpCpG motif analysis (**b**). Two-sided hypothesis testing was performed using PLINK v.1.9. To mitigate multiple-hypothesis testing, the significance threshold was set to genome-wide significance (*P* < 5 × 10^−8^). **c**, **d**, Locuszoom plot for somatic APOBEC3B-like mutagenesis association results, linkage disequilibrium and recombination rates around the genome-wide significant 22q13.1 locus in individuals with European (**c**) and East Asian (**d**) ancestry (*n* = 1,201 and 318 patients, respectively). Locuszoom plot for somatic APOBEC3B-like mutagenesis association results around the 22q13.1 locus in individuals with European (**e**) and East Asian (**f**) ancestry after conditioning on rs12628403. **g**, **h**, Association between rs2142833 and expression of *APOBEC3* genes in PCAWG tumour samples (adjusted for sex, age at diagnosis, histology and population structure in linear-regression models with two-sided hypothesis testing not corrected for multiple tests). For the box-and-whisker plot, the box denotes the interquartile range, with the median marked as a horizontal line. The whiskers extend as far as the range or 1.5× the interquartile range, whichever is less. Outliers are shown as points.

We integrated the set of 88 million germline genetic variant calls with somatic mutations in PCAWG, to study germline determinants of somatic mutation rates and patterns. First, we performed a genome-wide association study of somatic mutational processes with common germline variants (minor allele frequency (MAF) > 5%) in individuals with inferred European ancestry. An independent genome-wide association study was performed in East Asian individuals from Asian cancer genome projects. We focused on two prevalent endogenous mutational processes: spontaneous deamination of 5-methylcytosine at CpG dinucleotides^5^ (signature 1) and activity of the APOBEC3 family of cytidine deaminases^64^ (signatures 2 and 13). No locus reached genome-wide significance (*P* < 5 × 10^−8^) for signature 1 (Extended Data Fig. 10a, b). However, a locus at 22q13.1 predicted an APOBEC3B-like mutagenesis at the pan-cancer level^65^ (Fig. 6a). The strongest signal at 22q13.1 was driven by rs12628403, and the minor (non-reference) allele was protective against APOBEC3B-like mutagenesis (*β* = −0.43, *P* = 5.6 × 10^−9^, MAF = 8.2%, *n* = 1,201 donors) (Extended Data Fig. 10c). This variant tags a common, approximately 30-kb germline SV that deletes the *APOBEC3B* coding sequence and fuses the *APOBEC3B* 3′ untranslated region with the coding sequence of *APOBEC3A*. The deletion is known to increase breast cancer risk and APOBEC mutagenesis in breast cancer genomes^66,67^. Here, we found that rs12628403 reduces APOBEC3B-like mutagenesis specifically in cancer types with low levels of APOBEC mutagenesis (*β*_low_ = −0.50, *P*_low_ = 1 × 10^−8^; *β*_high_ = 0.17, *P*_high_ = 0.2), and increases APOBEC3A-like mutagenesis in cancer types with high levels of APOBEC mutagenesis (*β*_high_ = 0.44, *P*_high_ = 8 × 10^−4^; *β*_low_ = −0.21, *P*_low_ = 0.02). Moreover, we identified a second, novel locus at 22q13.1 that was associated with APOBEC3B-like mutagenesis across cancer types (rs2142833, *β* = 0.23, *P* = 1.3 × 10^−8^). We independently validated the association between both loci and APOBEC3B-like mutagenesis using East Asian individuals from Asian cancer genome projects (*β*_rs12628403_ = 0.57, *P*_rs12628403_ = 4.2 × 10^−12^; *β*_rs2142833_ = 0.58, *P*_rs2142833_ = 8 × 10^−15^) (Extended Data Fig. 10d). Notably, in a conditional analysis that accounted for rs12628403, we found that rs2142833 and rs12628403 are inherited independently in Europeans (*r*^*2*^<0.1), and rs2142833 remained significantly associated with APOBEC3B-like mutagenesis in Europeans (*β*_EUR_ = 0.17, *P*_EUR_ = 3 × 10^−5^) and East Asians (*β*_ASN_ = 0.25, *P*_ASN_ = 2 × 10^−3^) (Extended Data Fig. 10e, f). Analysis of donor-matched expression data further suggests that rs2142833 is a *cis*-expression quantitative trait locus (eQTL) for *APOBEC3B* at the pan-cancer level (*β* = 0.19, *P* = 2 × 10^−6^) (Extended Data Fig. 10g, h), consistent with *cis*-eQTL studies in normal cells^68,69^.

**Extended Data Fig. 11:**
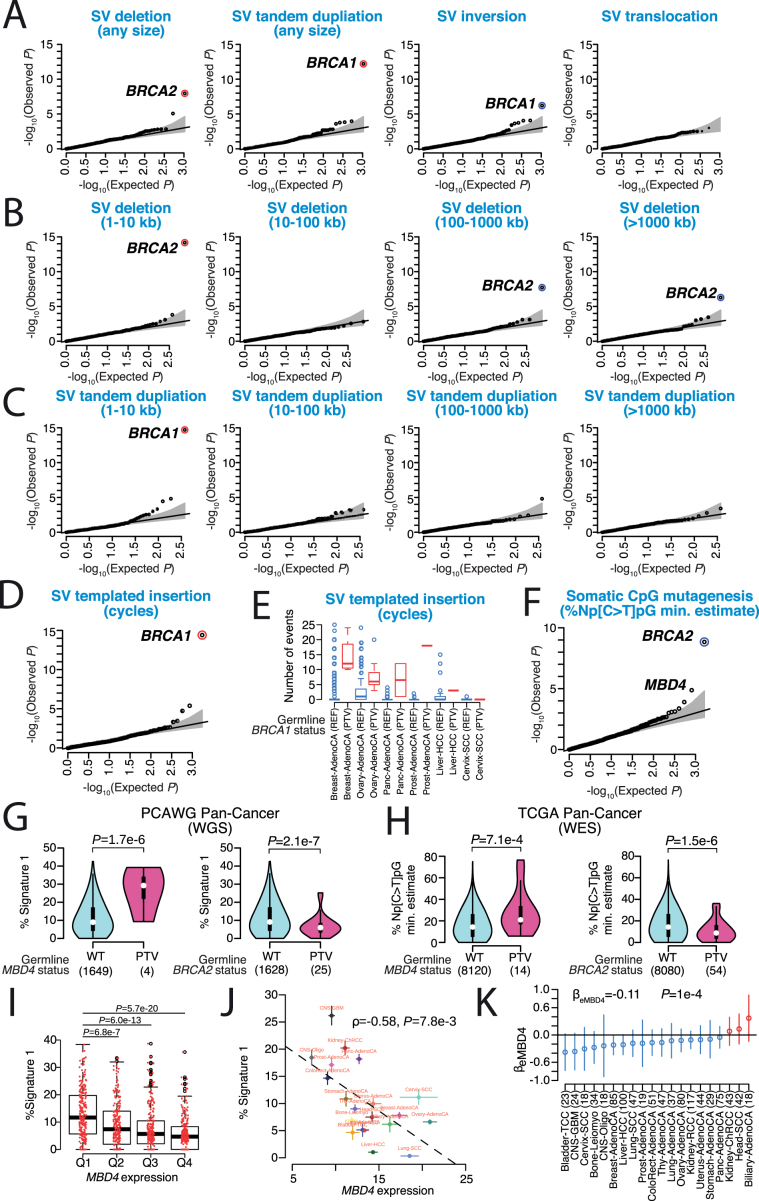
Association between rare germline PTVs in protein-coding genes and somatic mutational phenotypes. **a**–**d**, **f**, Data are based on two-sided rare-variant association testing across *n* = 2,583 patients, with a stringent *P* value threshold of *P* < 2.5 × 10^−6^ used to mitigate multiple-hypothesis testing (significant genes marked with coloured circles). Blue/red circles mark genes that decrease/increase somatic mutation rates. The black line represents the identity line that would be followed if the observed *P* values followed the null expectation, with the shaded area showing the 95% confidence intervals. **a**, *QQ* plots for the proportion of somatic SV deletions, tandem duplications, inversions and translocation in cancer genomes. **b**, *QQ* plots for the proportion of somatic SV deletions in cancer genomes stratified by four size groups (1–10 kb, 10–100 kb, 100–1,000 kb and >1,000 kb). **c**, *QQ* plots for the proportion of somatic SV tandem duplications in cancer genomes stratified by four size groups (1–10 kb, 10–100 kb, 100–1,000 kb and >1,000 kb). **d**, *QQ* plot for the presence or absence of somatic SV templated insertion (cycles) in cancer genomes. **e**, Number of SV-templated insertion cycles in PCAWG tumours with germline *BRCA1* PTVs. Only histological samples with at least one germline *BRCA1* PTV carrier are shown (*n* = 1,095 patients combined). The box denotes the interquartile range, with the median marked as a horizontal line. The whiskers extend as far as the range or 1.5× the interquartile range, whichever is less. Outliers are shown as points. **f**, *QQ* plot for somatic CpG mutagenesis in cancer genomes based on NpCpG motif analysis. **g**, Violin plots show estimated densities of the proportion of somatic CpG mutations in PCAWG donors with germline *MBD4* and *BRCA2* PTVs. The box denotes the interquartile range, with the median marked as a white point. The whiskers extend as far as the range or 1.5× the interquartile range, whichever is less. Two-sided hypothesis testing, not corrected for multiple testing, was performed using linear regression models. **h**, Replication of germline *MBD4* and *BRCA2* PTV associations with somatic CpG mutagenesis in TCGA whole-exome sequencing donors. Violin plots show the estimated density of the proportion of somatic CpG mutations in TCGA exomes with germline *MBD4* and *BRCA2* PTVs. The box denotes the interquartile range, with the median marked as a white point. The whiskers extend as far as the range or 1.5× the interquartile range, whichever is less. Two-sided hypothesis testing, not corrected for multiple testing, was performed using linear-regression models. **i**, Correlation between *MBD4* expression and somatic CpG mutagenesis in primary solid PCAWG tumours. Hypothesis testing was two-sided and not corrected for multiple testing, using linear-regression models. The box denotes the interquartile range, with the median marked as a horizontal line. The whiskers extend as far as the range or 1.5× the interquartile range, whichever is less. **j**, Data are mean ± s.e.m. across *n* = 20 tumour types. The dashed black line shows the fitted line to the data, estimated using linear-regression models. Hypothesis testing was two-sided and not corrected for multiple testing, using Spearman’s rank correlations. **k**, *MBD4* effect sizes (open circles) with 95% confidence intervals (error bars) for individual cancer types were estimated using linear-regression analysis after (if available) accounting for sex, age at diagnosis (young/old) and ICGC project. Hypothesis testing was two-sided and not corrected for multiple testing.

Second, we performed a rare-variant association study (MAF <0.5%) to investigate the relationship between germline PTVs and somatic DNA rearrangements in individuals with European ancestry (Extended Data Fig. 11a–c). Germline *BRCA2* and *BRCA1* PTVs were associated with an increased burden of small (less than 10 kb) somatic SV deletions (*P* = 1 × 10^−8^) and tandem duplications (*P* = 6 × 10^−13^), respectively, corroborating recent studies in breast and ovarian cancer^30,70^. In PCAWG data, this pattern also extends to other tumour types, including adenocarcinomas of the prostate and pancreas^6^, typically in the setting of biallelic inactivation. In addition, tumours with high levels of small SV tandem duplications frequently exhibited a novel and distinct class of SVs termed ‘cycles of templated insertions’^6^. These complex SV events consist of DNA templates that are copied from across the genome, joined into one contiguous sequence and inserted into a single derivative chromosome. We found a significant association between germline *BRCA1* PTVs and templated insertions at the pan-cancer level (*P* = 4 × 10^−15^) (Extended Data Fig. 11d, e). Whole-genome long-read sequencing data generated for a *BRCA1*-deficient PCAWG prostate tumour verified the small tandem-duplication and templated-insertion SV phenotypes (Fig. 6b). Almost all (20 out of 21) of *BRCA1*-associated tumours with a templated-insertion SV phenotype displayed combined germline and somatic hits in the gene. Together, these data suggest that biallelic inactivation of *BRCA1* is a driver of the templated-insertion SV phenotype.

Third, rare-variant association analysis revealed that patients with germline *MBD4* PTVs had increased rates of somatic C > T mutation rates at CpG dinucleotides (*P* < 2.5 × 10^−6^) (Fig. 6c and Extended Data Fig. 11f, g). Analysis of previously published whole-exome sequencing samples from the TCGA (*n* = 8,134) replicated the association between germline *MBD4* PTVs and increased somatic CpG mutagenesis at the pan-cancer level (*P* = 7.1 × 10^−4^) (Extended Data Fig. 11h). Moreover, gene-expression profiling revealed a significant but modest correlation between *MBD4* expression and somatic CpG mutation rates between and within PCAWG tumour types (Extended Data Fig. 11i–k). *MBD4* encodes a DNA-repair gene that removes thymidines from T:G mismatches within methylated CpG sites^71^, a functionality that would be consistent with a CpG mutational signature in cancer.

**Extended Data Fig. 12:**
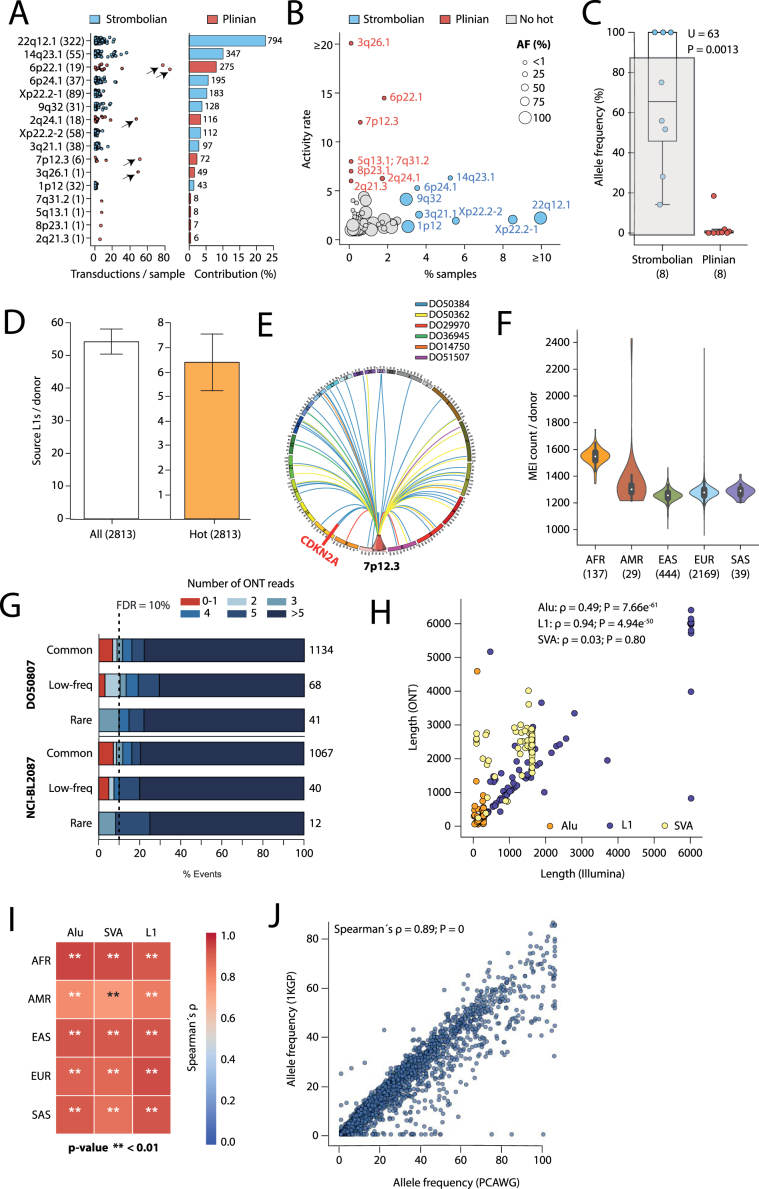
Germline MEI call set. **a**, Left, dots show the number of transductions promoted by each hot element in individual samples. Arrows highlight retrotransposition burst. Right, the contribution of each hot locus is represented. The total number of transductions mediated by each source element is shown on the right. **b**, Source L1 activity rate (that is, measured as the average number of transductions mediated by an element) versus the percentage of samples with retrotransposition activity in which the germline element is active. For visualization purposes, extreme points observed for a source L1 with an activity rate of 49 and for a L1 active in 31% of the samples are shown at ≥20 and ≥10, respectively. **c**, Contrasting allele frequencies for Strombolian and Plinian source loci (sample sizes shown under each axis label). The box denotes the interquartile range, with the median marked as a white point. The whiskers extend as far as the range or 1.5× the interquartile range, whichever is less. Hypothesis testing was performed using two-sided Mann–Whitney *U*-tests without correction for multiple tests. **d**, Numbers of active and hot source L1 elements per donor. Data are mean ± s.d. number of elements per donor. **e**, The novel Plinian source element on 7p12.3 mediates 72 transductions among only 6 cancer samples. This generates a transduction that induces the deletion of the tumour-suppressor gene *CDKN2A*. **f**, Violin plots show the estimated number of distinct germline MEI alleles per PCAWG donor. The box denotes the interquartile range, with the median marked as a white point. The whiskers extend as far as the range or 1.5× the interquartile range, whichever is less. Donors are grouped according to their genetic ancestry: AFR, African; AMR, admixed American; EAS, East Asian; EUR, European; SAS, South Asian. Sample sizes are shown under each axis label. **g**, For each type of MEI (L1, Alu and SVA) identified both in PCAWG and in the 1000 Genomes Project (1KGP), the correlations between allele frequency estimates per ancestry derived from both projects are displayed in a blue (0) to red (1) coloured gradient. *n* = 2,583 PCAWG patients. Two-sided hypothesis testing was performed using Spearman’s rank correlations without correction for multiple tests. **h**, Example correlation between MEI allele frequencies derived from PCAWG and the 1000 Genomes Project for individuals with European ancestry (*n* = 1,201 patients in PCAWG). Two-sided hypothesis testing was performed using Spearman’s rank correlations without correction for multiple tests. **i**, Evaluation of TraFiC-mem false-discovery rate on a liver hepatocellular carcinoma sample (DO50807) and a cell line (NCI-BL2087) sequenced using single-molecule sequencing with MinION (Oxford Nanopore). For each allele frequency bin (common, >5%; low frequency, 1–5%; rare, <1%), the percentage of events supported by *N* long reads is represented (*N* ranges from 0–1 to more than 5). MEIs supported by at least two Nanopore reads were considered to be true positives (blue palette) and were classified as false positives (red) otherwise. The total number of germline MEIs per allele frequency bin is shown on the right. **j**, Correlation between predicted MEI lengths from Illumina and Nanopore data. Two-sided hypothesis testing was performed using Spearman’s rank correlations without correction for multiple testing.

Fourth, we assessed long interspersed nuclear elements (LINE-1; L1 hereafter) that mediate somatic retrotransposition events^72–74^. We identified 114 germline source L1 elements capable of active somatic retrotransposition, including 70 that represent insertions with respect to the human reference genome (Fig. 6d and Supplementary Table 7), and 53 that were tagged by single-nucleotide polymorphisms in strong linkage disequilibrium (Supplementary Table 7). Only 16 germline L1 elements accounted for 67% (2,440 out of 3,669) of all L1-mediated transductions^10^ detected in the PCAWG dataset (Extended Data Fig. 12a). These 16 hot-L1 elements followed two broad patterns of somatic activity (8 of each), which we term Strombolian and Plinian in analogy to patterns of volcanic activity. Strombolian L1s are frequently active in cancer, but mediate only small-to-modest eruptions of somatic L1 activity in cancer samples (Extended Data Fig. 12b). By contrast, Plinian L1s are more rarely seen, but display aggressive somatic activity. Whereas Strombolian elements are typically relatively common (MAF > 2%) and sometimes even fixed in the human population, all Plinian elements were infrequent (MAF ≤ 2%) in PCAWG donors (Extended Data Fig. 12c; *P* = 0.001, Mann–Whitney *U*-test). This dichotomous pattern of activity and allele frequency may reflect differences in age and selective pressures, with Plinian elements potentially inserted into the human germline more recently. PCAWG donors bear on average between 50 and 60 L1 source elements and between 5 and 7 elements with hot activity (Extended Data Fig. 12d), but only 38% (1,075 out of 2,814) of PCAWG donors carried ≥1 Plinian element. Some L1 germline source loci caused somatic loss of tumour-suppressor genes (Extended Data Fig. 12e). Many are restricted to individual continental population ancestries (Extended Data Fig. 12f–j).

## Replicative immortality

One of the hallmarks of cancer is the ability of cancer to evade cellular senescence^21^. Normal somatic cells typically have finite cell division potential; telomere attrition is one mechanism to limit numbers of mitoses^75^. Cancers enlist multiple strategies to achieve replicative immortality. Overexpression of the telomerase gene, *TERT*, which maintains telomere lengths, is especially prevalent. This can be achieved through point mutations in the promoter that lead to de novo transcription factor binding^34,37^; hitching *TERT* to highly active regulatory elements elsewhere in the genome^46,76^; insertions of viral enhancers upstream of the gene^77,78^; and increased dosage through chromosomal amplification, as we have seen in melanoma (Fig. 5b). In addition, there is an ‘alternative lengthening of telomeres’ (ALT) pathway, in which telomeres are lengthened through homologous recombination, mediated by loss-of-function mutations in the *ATRX* and *DAXX* genes^79^.

As reported in a companion paper^13^, 16% of tumours in the PCAWG dataset exhibited somatic mutations in at least one of *ATRX*, *DAXX* and *TERT*. *TERT* alterations were detected in 270 samples, whereas 128 tumours had alterations in *ATRX* or *DAXX*, of which 71 were protein-truncating. In the companion paper, which focused on describing patterns of ALT and *TERT-*mediated telomere maintenance^13^, 12 features of telomeric sequence were measured in the PCAWG cohort. These included counts of nine variants of the core hexameric sequence, the number of ectopic telomere-like insertions within the genome, the number of genomic breakpoints and telomere length as a ratio between tumour and normal. Here we used the 12 features as an overview of telomere integrity across all tumours in the PCAWG dataset.

**Fig. 7:**
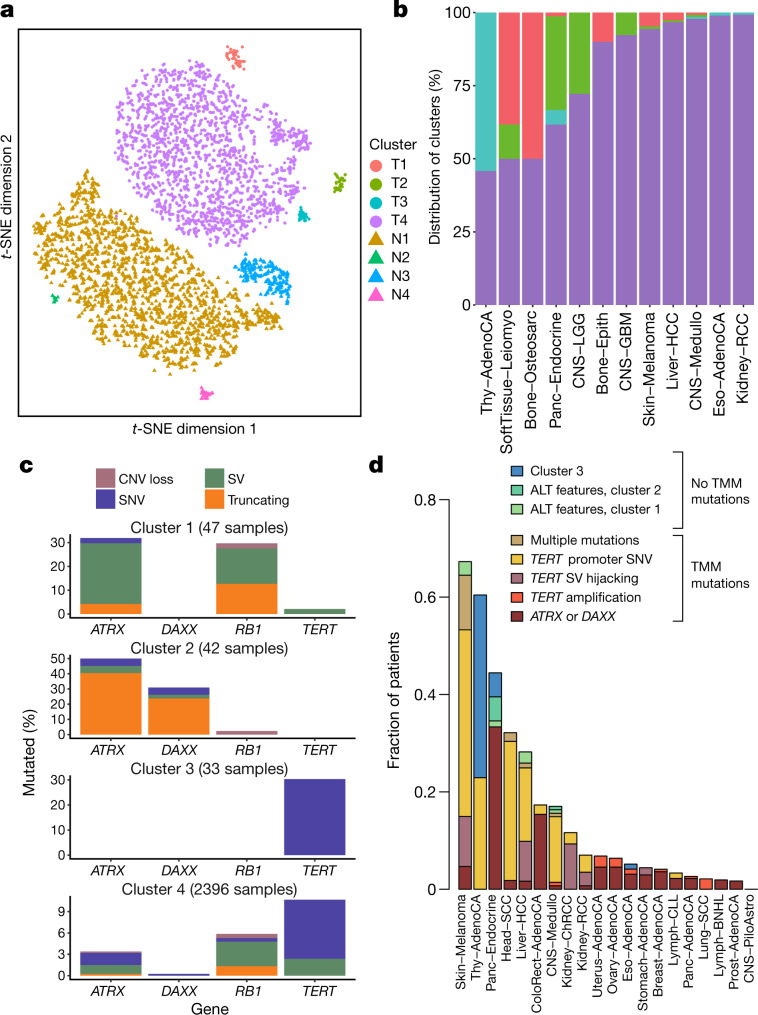
Telomere sequence patterns across PCAWG. **a**, Scatter plot of the clusters of telomere patterns identified across PCAWG using *t*-distributed stochastic neighbour embedding (*t*-SNE), based on *n* = 2,518 tumour samples and their matched normal samples. Axes have arbitrary dimensions such that samples with similar telomere profiles are clustered together and samples with dissimilar telomere profiles are far apart with high probability. **b**, Distribution of the four tumour-specific clusters of telomere patterns in selected tumour types from PCAWG. **c**, Distribution of relevant driver mutations associated with alternative lengthening of telomere and normal telomere maintenance across the four clusters. **d**, Distribution of telomere maintenance abnormalities across tumour types with more than 40 patients in PCAWG. Samples were classified as tumour clusters 1–3 if they fell into a relevant cluster without mutations in *TERT*, *ATRX* or *DAXX* and had no ALT phenotype. TMM, telomere maintenance mechanisms.

**Extended Data Fig. 13:**
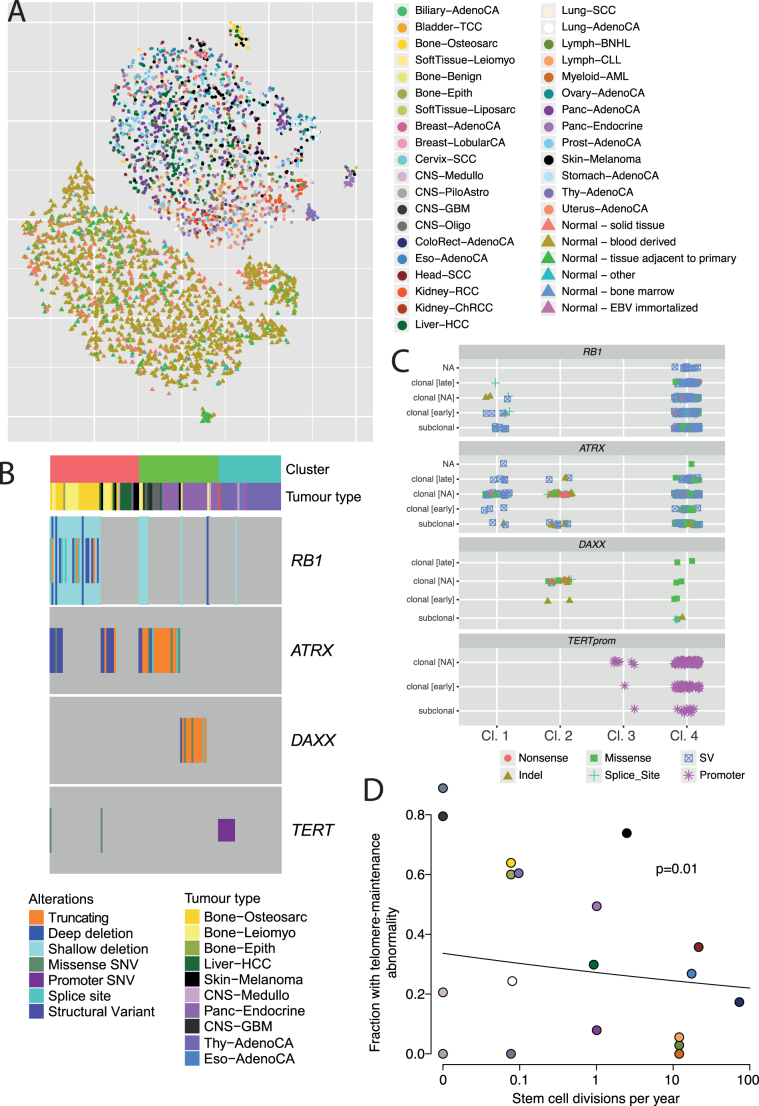
Different mechanisms of telomere lengthening in cancer. **a**, Scatter plot showing the four clusters of tumour-specific telomere patterns identified across PCAWG samples, together with the clusters of matched normal samples, generated by *t*-distributed stochastic neighbour embedding. Circles represent tumour samples and triangles represent matched normal samples. Points are coloured by tissue of origin. Data are based on *n* = 2,518 tumour samples and their matched normal samples. **b**, Patterns of comutation of the relevant driver mutations across individual patients. Columns in plot represent individual patients, coloured by type of abnormality observed. **c**, Distribution of clonality of driver mutations in genes relevant to telomere maintenance across clusters. Clonal [early], clonal mutations that occurred before duplications involving the relevant chromosome (including whole-genome duplications); clonal [late], clonal mutations that occurred after such duplications; and clonal [NA], mutations that occurred when no duplication was observed. **d**, Relationship between the estimated number of stem cell divisions per year and rate of telomere maintenance abnormalities across tumour types. The analysis uses data on estimated rates of stem cell division per year across *n* = 19 tissue types previously collated from the literature^82^. Tumour types are coloured according to the scheme shown in Extended Data Fig. 3. Two-sided hypothesis testing was performed using likelihood ratio tests on Poisson regression models with no correction for multiple tests.

On the basis of these 12 features, tumour samples formed 4 distinct subclusters (Fig. 7a and Extended Data Fig. 13a), suggesting that telomere-maintenance mechanisms are more diverse than the well-established *TERT* and ALT dichotomy. Clusters C1 (47 tumours) and C2 (42 tumours) were enriched for traits of the ALT pathway—having longer telomeres, more genomic breakpoints, more ectopic telomere insertions and variant telomere sequence motifs (Supplementary Fig. 9). C1 and C2 were distinguished from one another by the latter having a considerable increase in the number of TTCGGG and TGAGGG variant motifs among the telomeric hexamers. Thyroid adenocarcinomas were markedly enriched among C3 samples (26 out of 33 C3 samples; *P* < 10^−16^); the C1 cluster (ALT subtype 1) was common among sarcomas; and both pancreatic endocrine neoplasms and low-grade gliomas had a high proportion of samples in the C2 cluster (ALT subtype 2) (Fig. 7b). Notably, some of the thyroid adenocarcinomas and pancreatic neuroendocrine tumours that cluster together (cluster C3) had matched normal samples that also cluster together (normal cluster N3) (Extended Data Fig. 13a) and which share common properties. For example, the GTAGGG repeat was overrepresented among samples in this group (Supplementary Fig. 10).

Somatic driver mutations were also unevenly distributed across the four clusters (Fig. 7c). C1 tumours were enriched for *RB1* mutations or SVs (*P* = 3 × 10^−5^), as well as frequent SVs that affected *ATRX* (*P* = 6 × 10^−14^), but not *DAXX*. *RB1* and *ATRX* mutations were largely mutually exclusive (Extended Data Fig. 13b). By contrast, C2 tumours were enriched for somatic point mutations in *ATRX* and *DAXX* (*P* = 6 × 10^−5^), but not *RB1*. The enrichment of *RB1* mutations in C1 remained significant when only leiomyosarcomas and osteosarcomas were considered, confirming that this enrichment is not merely a consequence of the different distribution of tumour types across clusters. C3 samples had frequent *TERT* promoter mutations (30%; *P* = 2 × 10^−6^).

There was a marked predominance of *RB1* mutations in C1. Nearly a third of the samples in C1 contained an *RB1* alteration, which were evenly distributed across truncating SNVs, SVs and shallow deletions (Extended Data Fig. 13c). Previous research has shown that *RB1* mutations are associated with long telomeres in the absence of *TERT* mutations and *ATRX* inactivation^80^, and studies using mouse models have shown that knockout of Rb-family proteins causes elongated telomeres^81^. The association with the C1 cluster here suggests that *RB1* mutations can represent another route to activating the ALT pathway, which has subtly different properties of telomeric sequence compared with the inactivation of *DAXX*—these fall almost exclusively in cluster C2.

Tumour types with the highest rates of abnormal telomere maintenance mechanisms often originate in tissues that have low endogenous replicative activity (Fig. 7d). In support of this, we found an inverse correlation between previously estimated rates of stem cell division across tissues^82^ and the frequency of telomere maintenance abnormalities (*P* = 0.01, Poisson regression) (Extended Data Fig. 13d). This suggests that restriction of telomere maintenance is an important tumour-suppression mechanism, particularly in tissues with low steady-state cellular proliferation, in which a clone must overcome this constraint to achieve replicative immortality.

## Conclusions and future perspectives

The resource reported in this paper and its companion papers has yielded insights into the nature and timing of the many mutational processes that shape large- and small-scale somatic variation in the cancer genome; the patterns of selection that act on these variations; the widespread effect of somatic variants on transcription; the complementary roles of the coding and non-coding genome for both germline and somatic mutations; the ubiquity of intratumoral heterogeneity; and the distinctive evolutionary trajectory of each cancer type. Many of these insights can be obtained only from an integrated analysis of all classes of somatic mutation on a whole-genome scale, and would not be accessible with, for example, targeted exome sequencing.

The promise of precision medicine is to match patients to targeted therapies using genomics. A major barrier to its evidence-based implementation is the daunting heterogeneity of cancer chronicled in these papers, from tumour type to tumour type, from patient to patient, from clone to clone and from cell to cell. Building meaningful clinical predictors from genomic data can be achieved, but will require knowledge banks comprising tens of thousands of patients with comprehensive clinical characterization^83^. As these sample sizes will be too large for any single funding agency, pharmaceutical company or health system, international collaboration and data sharing will be required. The next phase of ICGC, ICGC-ARGO (https:// www.icgc-argo.org/), will bring the cancer genomics community together with healthcare providers, pharmaceutical companies, data science and clinical trials groups to build comprehensive knowledge banks of clinical outcome and treatment data from patients with a wide variety of cancers, matched with detailed molecular profiling.

Extending the story begun by TCGA, ICGC and other cancer genomics projects, the PCAWG has brought us closer to a comprehensive narrative of the causal biological changes that drive cancer phenotypes. We must now translate this knowledge into sustainable, meaningful clinical treatments.

## Methods

### Samples

We compiled an inventory of matched tumour–normal whole-cancer genomes in the ICGC Data Coordinating Centre. Most samples came from treatment-naive, primary cancers, although a small number of donors had multiple samples of primary, metastatic and/or recurrent tumours. Our inclusion criteria were: (1) matched tumour and normal specimen pair; (2) a minimal set of clinical fields; and (3) characterization of tumour and normal whole genomes using Illumina HiSeq paired-end sequencing reads.

We collected genome data from 2,834 donors, representing all ICGC and TCGA donors that met these criteria at the time of the final data freeze in autumn 2014 (Extended Data Table 1). After quality assurance (Supplementary Methods 2.5), data from 176 donors were excluded as unusable, 75 had minor issues that could affect some analyses (grey-listed donors) and 2,583 had data of optimal quality (white-listed donors) (Supplementary Table 1). Across the 2,658 white- and grey-listed donors, whole-genome sequences were available from 2,605 primary tumours and 173 metastases or local recurrences. Matching normal samples were obtained from blood (2,064 donors), tissue adjacent to the primary tumour (87 donors) or from distant sites (507 donors). Whole-genome sequencing data were available for tumour and normal DNA for the entire cohort. The mean read coverage was 39× for normal samples, whereas tumours had a bimodal coverage distribution with modes at 38× and 60× (Supplementary Fig. 1). The majority of specimens (65.3%) were sequenced using 101-bp paired-end reads. An additional 28% were sequenced with 100-bp paired-end reads. Of the remaining specimens, 4.7% were sequenced with read lengths longer than 101 bp, and 1.9% with read lengths shorter than 100 bp. The distribution of read lengths by tumour cohort is shown in Supplementary Fig. 11. Median read length for whole-genome sequencing paired-end reads was 101 bp (mean = 106.2, s.d. = 16.7; minimum–maximum = 50–151). RNA-sequencing data were collected and re-analysed centrally for 1,222 donors, including 1,178 primary tumours, 67 metastases or local recurrences and 153 matched normal tissue samples adjacent to the primary tumour.

Demographically, the cohort included 1,469 men (55%) and 1,189 women (45%), with a mean age of 56 years (range, 1–90 years) (Supplementary Table 1). Using population ancestry-differentiated single nucleotide polymorphisms, the ancestry distribution was heavily weighted towards donors of European descent (77% of total) followed by East Asians (16%), as expected for large contributions from European, North American and Australian projects (Supplementary Table 1).

We consolidated histopathology descriptions of the tumour samples, using the ICD-0-3 tumour site controlled vocabulary^89^. Overall, the PCAWG dataset comprises 38 distinct tumour types (Extended Data Table 1 and Supplementary Table 1). Although the most common tumour types are included in the dataset, their distribution does not match the relative population incidences, largely owing to differences among contributing ICGC/TCGA groups in the numbers of sequenced samples.

### Uniform processing and somatic variant calling

To generate a consistent set of somatic mutation calls that could be used for cross-tumour analyses, we analysed all 6,835 samples using a uniform set of algorithms for alignment, variant calling and quality control (Extended Data Fig. 1, Supplementary Fig. 2, Supplementary Table 3 and Supplementary Methods 2). We used the BWA-MEM algorithm^90^ to align each tumour and normal sample to human reference build hs37d5 (as used in the 1000 Genomes Project^91^). Somatic mutations were identified in the aligned data using three established pipelines, which were run independently on each tumour–normal pair. Each of the three pipelines—labelled ‘Sanger’^92–95^, ‘EMBL/DKFZ’^96,97^ and ‘Broad’^98–101^ after the computational biology groups that created or assembled them—consisted of multiple software packages for calling somatic SNVs, small indels, CNAs and somatic SVs (with intrachromosomal SVs defined as those >100 bp). Two additional variant algorithms^102,103^ were included to further improve accuracy across a broad range of clonal and subclonal mutations. We tested different merging strategies using validation data, and choses the optimal method for each variant type to generate a final consensus set of mutation calls (Supplementary Methods S2.4).

Somatic retrotransposition events, including Alu and LINE-1 insertions^72^, L1-mediated transductions^73^ and pseudogene formation^104^, were called using a dedicated pipeline^73^. We removed these retrotransposition events from the somatic SV call-set. Mitochondrial DNA mutations were called using a published algorithm^105^. RNA-sequencing data were uniformly processed to quantify normalized gene-level expression, splicing variation and allele-specific expression, and to identify fusion transcripts, alternative promoter usage and sites of RNA editing^8^.

### Integration, phasing and validation of germline variant call-sets

Calls of common (≥1% frequency in PCAWG) and rare (<1%) germline variants including single-nucleotide polymorphisms, indels, SVs and mobile-element insertions (MEIs) were generated using a population-scale genetic polymorphism-detection approach^91,106^. The uniform germline data-processing workflow comprised variant identification using six different variant-calling algorithms^96,107,108^ and was orchestrated using the Butler workflow system^109^.

We performed call-set benchmarking, merging, variant genotyping and statistical haplotype-block phasing^91^ (Supplementary Methods 3.4). Using this strategy, we identified 80.1 million germline single-nucleotide polymorphisms, 5.9 million germline indels, 1.8 million multi-allelic short (<50 bp) germline variants, as well as germline SVs ≥ 50 bp in size including 29,492 biallelic deletions and 27,254 MEIs (Supplementary Table 2). We statistically phased this germline variant set using haplotypes from the 1000 Genomes Project^91^ as a reference panel, yielding an N50-phased block length of 265 kb based on haploid chromosomes from donor-matched tumour genomes. Precision estimates for germline SNVs and indels were >99% for the phased merged call-set, and sensitivity estimates ranged from 92% to 98%.

### Core alignment and variant calling by cloud computing

The requirement to uniformly realign and call variants on nearly 5,800 whole genomes (tumour plus normal) presented considerable computational challenges, and raised ethical issues owing to the use of data from different jurisdictions (Extended Data Table 2). To process the data, we adopted a cloud-computing architecture^26^ in which the alignment and variant calling was spread across 13 data centres on 3 continents, representing a mixture of commercial, infrastructure-as-a-service, academic cloud compute and traditional academic high-performance computer clusters (Supplementary Table 3). Together, the effort used 10 million CPU-core hours.

To generate reproducible variant calling across the 13 data centres, we built the core pipelines into Docker containers^28^, in which the workflow description, required code and all associated dependencies were packaged together in stand-alone packages. These heavily tested, extensively validated workflows are available for download (Box 1).

### Validation, benchmarking and merging of somatic variant calls

To evaluate the performance of each of the mutation-calling pipelines and determine an integration strategy, we performed a large-scale deep-sequencing validation experiment (Supplementary Notes 1). We selected a pilot set of 63 representative tumour–normal pairs, on which we ran the 3 core pipelines, together with a set of 10 additional somatic variant-calling pipelines contributed by members of the PCAWG SNV Calling Methods Working Group. Sufficient DNA remained for 50 of the 63 cases for validation, which was performed by hybridization of tumour and matched normal DNA to a custom RNA bait set, followed by deep sequencing, as previously described^29^. Although performed using the same sequencing chemistry as the original whole-genome sequencing analyses, the considerably greater depth achieved in the validation experiment enabled accurate assessment of sensitivity and precision of variant calls. Variant calls in repeat-masked regions were not tested, owing to the challenge of designing reliable validation probes in these areas.

The 3 core pipelines had individual estimates of sensitivity of 80–90% to detect a true somatic SNV called by any of the 13 pipelines; with >95% of SNV calls made by each of the core pipelines being genuine somatic variants (Fig. 1a). For indels—a more-challenging class of variants to identify in short-read sequencing data—the 3 core algorithms had individual sensitivity estimates in the range of 40–50%, with precision 70–95% (Fig. 1b). Validation of SV calls is inherently more difficult, as methods based on PCR or hybridization to RNA baits often fail to isolate DNA that spans the breakpoint. To assess the accuracy of SV calls, we therefore used the property that an SV must either generate a copy-number change or be balanced, whereas artefactual calls will not respect this property. For individual SV-calling algorithms, we estimated precision to be in the range of 80–95% for samples in the 63-sample pilot dataset.

Next, we examined multiple methods for merging calls made by several algorithms into a single definitive call-set to be used for downstream analysis. The final consensus calls for SNVs were based on a simple approach that required two or more methods to agree on a call. For indels, because methods were less concordant, we used stacked logistic regression^110,111^ to integrate the calls. The merged SV set includes all calls made by two or more of the four primary SV-calling algorithms^96,100,112,113^. Consensus CNA calls were obtained by joining the outputs of six individual CNA-calling algorithms with SV consensus breakpoints to obtain base-pair resolution CNAs (Supplementary Methods 2.4.3). Consensus purity and ploidy were derived, and a multitier system was developed for consensus copy-number calls (Supplementary Methods 2.4.3, and described in detail elsewhere^7^).

Overall, the sensitivity and precision of the consensus somatic variant calls were 95% (90% confidence interval, 88–98%) and 95% (90% confidence interval, 71–99%), respectively, for SNVs (Extended Data Fig. 2). For somatic indels, sensitivity and precision were 60% (90% confidence interval, 34–72%) and 91% (90% confidence interval, 73–96%), respectively. Regarding SVs, we estimate the sensitivity of the merging algorithm to be 90% for true calls generated by any one calling pipeline; precision was estimated to be 97.5%. That is, 97.5% of SVs in the merged SV call-set had an associated copy-number change or balanced partner rearrangement. The improvement in calling accuracy from combining different pipelines was most noticeable in variants that had low variant allele fractions, which are likely to originate from subclonal populations of the tumour (Fig. 1c, d). There remains much work to be done to improve indel calling software; we still lack sensitivity for calling even fully clonal complex indels from short-read sequencing data.

### Reporting summary

Further information on research design is available in the Nature Research Reporting Summary linked to this paper.

## Online content

Any methods, additional references, Nature Research reporting summaries, source data, extended data, supplementary information, acknowledgements, peer review information; details of author contributions and competing interests; and statements of data and code availability are available at 10.1038/s41586-020-1969-6.

## Supplementary information

Supplementary Information This file contains Supplementary Figures 1-19, Supplementary Methods and Supplementary Notes 1-6

Reporting Summary

Supplementary Tables This zipped file contains Supplementary Tables 1-21 and a Supplementary Table Guide

Supplementary Information Supplementary information

## Figures and Tables

**Extended Data Table 1:**
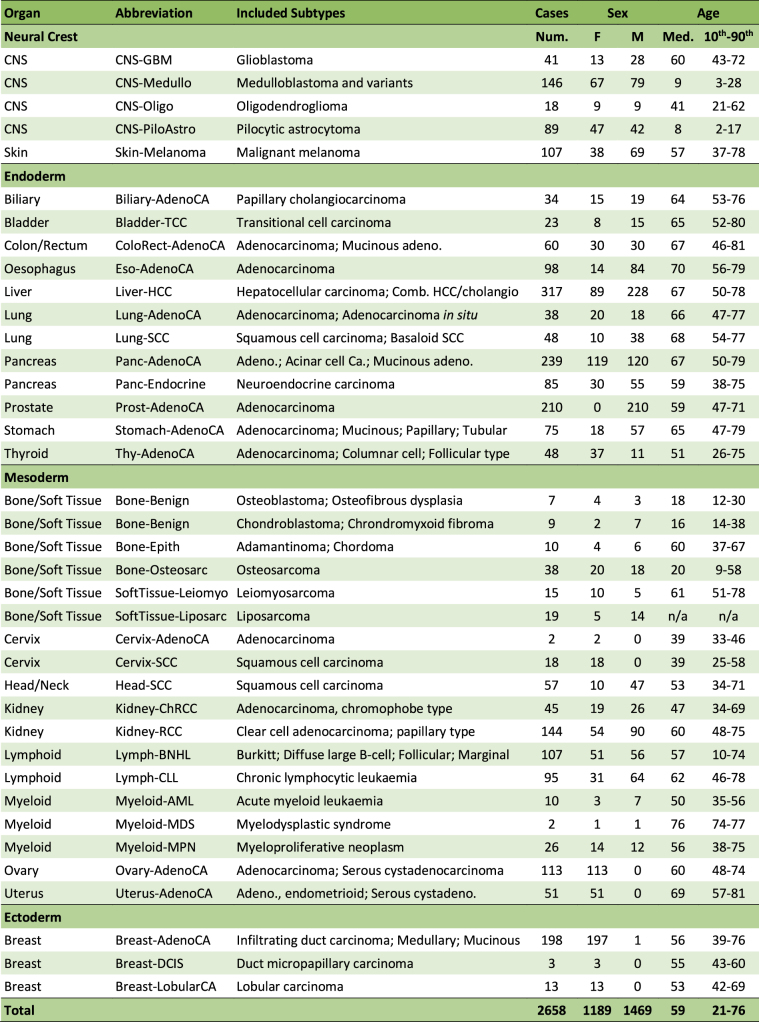
Overview of the tumour types included in PCAWG project

Adeno., adenocarcinoma; Ca., carcinoma; Comb., combined; F, female; HCC, hepatocellular carcinoma; M, male; Med, median; 10–90th, 10–90th centiles; SCC, squamous cell carcinoma.

**Extended Data Table 2:**
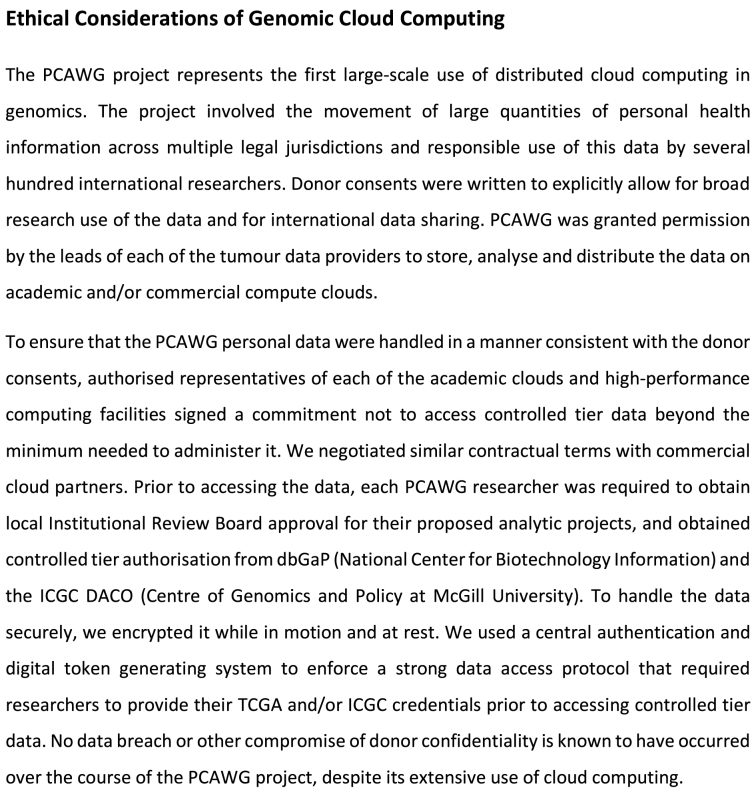
Ethical considerations of genomic cloud computing

**Extended Data Table 3:**
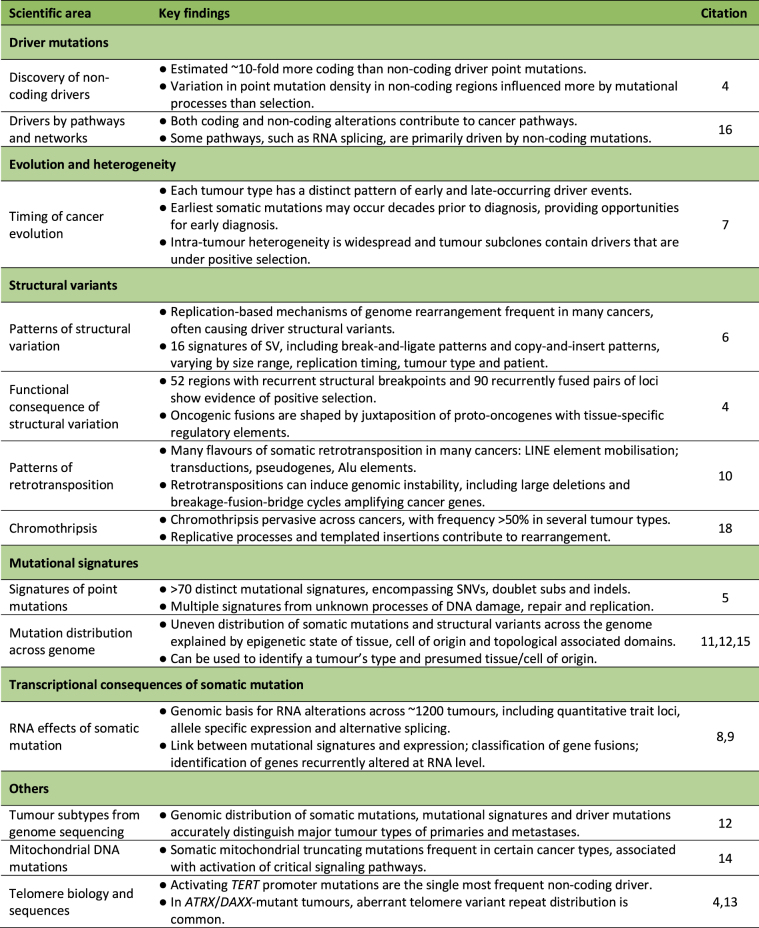
Scientific output using PCAWG data, in bite-size chunks

Key findings are described further in associated papers^4–18^.

## Data Availability

The PCAWG-generated alignments, somatic variant calls, annotations and derived datasets are available for general research use for browsing and download at http://dcc.icgc.org/pcawg/ (Box 1 and Supplementary Table 4). In accordance with the data access policies of the ICGC and TCGA projects, most molecular, clinical and specimen data are in an open tier which does not require access approval. To access potentially identifying information, such as germline alleles and underlying read data, researchers will need to apply to the TCGA Data Access Committee (DAC) via dbGaP (https://dbgap.ncbi.nlm.nih.gov/aa/wga.cgi?page=login) for access to the TCGA portion of the dataset, and to the ICGC Data Access Compliance Office (DACO; http://icgc.org/daco) for the ICGC portion. In addition, to access somatic single nucleotide variants derived from TCGA donors, researchers will also need to obtain dbGaP authorization. Beyond the core sequence data and variant call-sets, the analyses in this paper used a number of datasets that were derived from the variant calls (Supplementary Table 4). The individual datasets are available at Synapse (https://www.synapse.org/), and are denoted with synXXXXX accession numbers; all these datasets are also mirrored at https://dcc.icgc.org, with full links, filenames, accession numbers and descriptions detailed in Supplementary Table 4. The datasets encompass: clinical data from each patient including demographics, tumour stage and vital status (syn10389158); harmonized tumour histopathology annotations using a standardised hierarchical ontology (syn1038916); inferred purity and ploidy values for each tumour sample (syn8272483); driver mutations for each patient from their cancer genome spanning all classes of variant, and coding versus non-coding drivers (syn11639581); mutational signatures inferred from PCAWG donors (syn11804065), including APOBEC mutagenesis (syn7437313); and transcriptional data from RNA sequencing, including gene expression levels (syn5553985, syn5553991, syn8105922) and gene fusions (syn10003873, syn7221157).

## Code availability

Computational pipelines for calling somatic mutations are available to the public at https://dockstore.org/organizations/PCAWG/collections/PCAWG. A range of data-visualization and -exploration tools are also available for the PCAWG data (Box 1).
