# Author Correction: Pan-cancer analysis of whole genomes

**DOI:** 10.1038/s41586-022-05598-w

**Published:** 2023-01-25

**Authors:** The ICGC/TCGA Pan-Cancer Analysis of Whole Genomes Consortium, Lauri A. Aaltonen, Federico Abascal, Adam Abeshouse, Hiroyuki Aburatani, David J. Adams, Nishant Agrawal, Keun Soo Ahn, Sung-Min Ahn, Hiroshi Aikata, Rehan Akbani, Kadir C. Akdemir, Hikmat Al-Ahmadie, Sultan T. Al-Sedairy, Fatima Al-Shahrour, Malik Alawi, Monique Albert, Kenneth Aldape, Ludmil B. Alexandrov, Adrian Ally, Kathryn Alsop, Eva G. Alvarez, Fernanda Amary, Samirkumar B. Amin, Brice Aminou, Ole Ammerpohl, Matthew J. Anderson, Yeng Ang, Davide Antonello, Pavana Anur, Samuel Aparicio, Elizabeth L. Appelbaum, Yasuhito Arai, Axel Aretz, Koji Arihiro, Shun-ichi Ariizumi, Joshua Armenia, Laurent Arnould, Sylvia Asa, Yassen Assenov, Gurnit Atwal, Sietse Aukema, J. Todd Auman, Miriam R. R. Aure, Philip Awadalla, Marta Aymerich, Gary D. Bader, Adrian Baez-Ortega, Matthew H. Bailey, Peter J. Bailey, Miruna Balasundaram, Saianand Balu, Pratiti Bandopadhayay, Rosamonde E. Banks, Stefano Barbi, Andrew P. Barbour, Jonathan Barenboim, Jill Barnholtz-Sloan, Hugh Barr, Elisabet Barrera, John Bartlett, Javier Bartolome, Claudio Bassi, Oliver F. Bathe, Daniel Baumhoer, Prashant Bavi, Stephen B. Baylin, Wojciech Bazant, Duncan Beardsmore, Timothy A. Beck, Sam Behjati, Andreas Behren, Beifang Niu, Cindy Bell, Sergi Beltran, Christopher Benz, Andrew Berchuck, Anke K. Bergmann, Erik N. Bergstrom, Benjamin P. Berman, Daniel M. Berney, Stephan H. Bernhart, Rameen Beroukhim, Mario Berrios, Samantha Bersani, Johanna Bertl, Miguel Betancourt, Vinayak Bhandari, Shriram G. Bhosle, Andrew V. Biankin, Matthias Bieg, Darell Bigner, Hans Binder, Ewan Birney, Michael Birrer, Nidhan K. Biswas, Bodil Bjerkehagen, Tom Bodenheimer, Lori Boice, Giada Bonizzato, Johann S. De Bono, Arnoud Boot, Moiz S. Bootwalla, Ake Borg, Arndt Borkhardt, Keith A. Boroevich, Ivan Borozan, Christoph Borst, Marcus Bosenberg, Mattia Bosio, Jacqueline Boultwood, Guillaume Bourque, Paul C. Boutros, G. Steven Bova, David T. Bowen, Reanne Bowlby, David D. L. Bowtell, Sandrine Boyault, Rich Boyce, Jeffrey Boyd, Alvis Brazma, Paul Brennan, Daniel S. Brewer, Arie B. Brinkman, Robert G. Bristow, Russell R. Broaddus, Jane E. Brock, Malcolm Brock, Annegien Broeks, Angela N. Brooks, Denise Brooks, Benedikt Brors, Søren Brunak, Timothy J. C. Bruxner, Alicia L. Bruzos, Alex Buchanan, Ivo Buchhalter, Christiane Buchholz, Susan Bullman, Hazel Burke, Birgit Burkhardt, Kathleen H. Burns, John Busanovich, Carlos D. Bustamante, Adam P. Butler, Atul J. Butte, Niall J. Byrne, Anne-Lise Børresen-Dale, Samantha J. Caesar-Johnson, Andy Cafferkey, Declan Cahill, Claudia Calabrese, Carlos Caldas, Fabien Calvo, Niedzica Camacho, Peter J. Campbell, Elias Campo, Cinzia Cantù, Shaolong Cao, Thomas E. Carey, Joana Carlevaro-Fita, Rebecca Carlsen, Ivana Cataldo, Mario Cazzola, Jonathan Cebon, Robert Cerfolio, Dianne E. Chadwick, Dimple Chakravarty, Don Chalmers, Calvin Wing Yiu Chan, Kin Chan, Michelle Chan-Seng-Yue, Vishal S. Chandan, David K. Chang, Stephen J. Chanock, Lorraine A. Chantrill, Aurélien Chateigner, Nilanjan Chatterjee, Kazuaki Chayama, Hsiao-Wei Chen, Jieming Chen, Ken Chen, Yiwen Chen, Zhaohong Chen, Andrew D. Cherniack, Jeremy Chien, Yoke-Eng Chiew, Suet-Feung Chin, Juok Cho, Sunghoon Cho, Jung Kyoon Choi, Wan Choi, Christine Chomienne, Zechen Chong, Su Pin Choo, Angela Chou, Angelika N. Christ, Elizabeth L. Christie, Eric Chuah, Carrie Cibulskis, Kristian Cibulskis, Sara Cingarlini, Peter Clapham, Alexander Claviez, Sean Cleary, Nicole Cloonan, Marek Cmero, Colin C. Collins, Ashton A. Connor, Susanna L. Cooke, Colin S. Cooper, Leslie Cope, Vincenzo Corbo, Matthew G. Cordes, Stephen M. Cordner, Isidro Cortés-Ciriano, Kyle Covington, Prue A. Cowin, Brian Craft, David Craft, Chad J. Creighton, Yupeng Cun, Erin Curley, Ioana Cutcutache, Karolina Czajka, Bogdan Czerniak, Rebecca A. Dagg, Ludmila Danilova, Maria Vittoria Davi, Natalie R. Davidson, Helen Davies, Ian J. Davis, Brandi N. Davis-Dusenbery, Kevin J. Dawson, Francisco M. De La Vega, Ricardo De Paoli-Iseppi, Timothy Defreitas, Angelo P. Dei Tos, Olivier Delaneau, John A. Demchok, Jonas Demeulemeester, German M. Demidov, Deniz Demircioğlu, Nening M. Dennis, Robert E. Denroche, Stefan C. Dentro, Nikita Desai, Vikram Deshpande, Amit G. Deshwar, Christine Desmedt, Jordi Deu-Pons, Noreen Dhalla, Neesha C. Dhani, Priyanka Dhingra, Rajiv Dhir, Anthony DiBiase, Klev Diamanti, Li Ding, Shuai Ding, Huy Q. Dinh, Luc Dirix, HarshaVardhan Doddapaneni, Nilgun Donmez, Michelle T. Dow, Ronny Drapkin, Oliver Drechsel, Ruben M. Drews, Serge Serge, Tim Dudderidge, Ana Dueso-Barroso, Andrew J. Dunford, Michael Dunn, Lewis Jonathan Dursi, Fraser R. Duthie, Ken Dutton-Regester, Jenna Eagles, Douglas F. Easton, Stuart Edmonds, Paul A. Edwards, Sandra E. Edwards, Rosalind A. Eeles, Anna Ehinger, Juergen Eils, Roland Eils, Adel El-Naggar, Matthew Eldridge, Kyle Ellrott, Serap Erkek, Georgia Escaramis, Shadrielle M. G. Espiritu, Xavier Estivill, Dariush Etemadmoghadam, Jorunn E. Eyfjord, Bishoy M. Faltas, Daiming Fan, Yu Fan, William C. Faquin, Claudiu Farcas, Matteo Fassan, Aquila Fatima, Francesco Favero, Nodirjon Fayzullaev, Ina Felau, Sian Fereday, Martin L. Ferguson, Vincent Ferretti, Lars Feuerbach, Matthew A. Field, J. Lynn Fink, Gaetano Finocchiaro, Cyril Fisher, Matthew W. Fittall, Anna Fitzgerald, Rebecca C. Fitzgerald, Adrienne M. Flanagan, Neil E. Fleshner, Paul Flicek, John A. Foekens, Kwun M. Fong, Nuno A. Fonseca, Christopher S. Foster, Natalie S. Fox, Michael Fraser, Scott Frazer, Milana Frenkel-Morgenstern, William Friedman, Joan Frigola, Catrina C. Fronick, Akihiro Fujimoto, Masashi Fujita, Masashi Fukayama, Lucinda A. Fulton, Robert S. Fulton, Mayuko Furuta, P. Andrew Futreal, Anja Füllgrabe, Stacey B. Gabriel, Steven Gallinger, Carlo Gambacorti-Passerini, Jianjiong Gao, Shengjie Gao, Levi Garraway, Øystein Garred, Erik Garrison, Dale W. Garsed, Nils Gehlenborg, Josep L. L. Gelpi, Joshy George, Daniela S. Gerhard, Clarissa Gerhauser, Jeffrey E. Gershenwald, Mark Gerstein, Moritz Gerstung, Gad Getz, Mohammed Ghori, Ronald Ghossein, Nasra H. Giama, Richard A. Gibbs, Bob Gibson, Anthony J. Gill, Pelvender Gill, Dilip D. Giri, Dominik Glodzik, Vincent J. Gnanapragasam, Maria Elisabeth Goebler, Mary J. Goldman, Carmen Gomez, Santiago Gonzalez, Abel Gonzalez-Perez, Dmitry A. Gordenin, James Gossage, Kunihito Gotoh, Ramaswamy Govindan, Dorthe Grabau, Janet S. Graham, Robert C. Grant, Anthony R. Green, Eric Green, Liliana Greger, Nicola Grehan, Sonia Grimaldi, Sean M. Grimmond, Robert L. Grossman, Adam Grundhoff, Gunes Gundem, Qianyun Guo, Manaswi Gupta, Shailja Gupta, Ivo G. Gut, Marta Gut, Jonathan Göke, Gavin Ha, Andrea Haake, David Haan, Siegfried Haas, Kerstin Haase, James E. Haber, Nina Habermann, Faraz Hach, Syed Haider, Natsuko Hama, Freddie C. Hamdy, Anne Hamilton, Mark P. Hamilton, Leng Han, George B. Hanna, Martin Hansmann, Nicholas J. Haradhvala, Olivier Harismendy, Ivon Harliwong, Arif O. Harmanci, Eoghan Harrington, Takanori Hasegawa, David Haussler, Steve Hawkins, Shinya Hayami, Shuto Hayashi, D. Neil Hayes, Stephen J. Hayes, Nicholas K. Hayward, Steven Hazell, Yao He, Allison P. Heath, Simon C. Heath, David Hedley, Apurva M. Hegde, David I. Heiman, Michael C. Heinold, Zachary Heins, Lawrence E. Heisler, Eva Hellstrom-Lindberg, Mohamed Helmy, Seong Gu Heo, Austin J. Hepperla, José María Heredia-Genestar, Carl Herrmann, Peter Hersey, Julian M. Hess, Holmfridur Hilmarsdottir, Jonathan Hinton, Satoshi Hirano, Nobuyoshi Hiraoka, Katherine A. Hoadley, Asger Hobolth, Ermin Hodzic, Jessica I. Hoell, Steve Hoffmann, Oliver Hofmann, Andrea Holbrook, Aliaksei Z. Holik, Michael A. Hollingsworth, Oliver Holmes, Robert A. Holt, Chen Hong, Eun Pyo Hong, Jongwhi H. Hong, Gerrit K. Hooijer, Henrik Hornshøj, Fumie Hosoda, Yong Hou, Volker Hovestadt, William Howat, Alan P. Hoyle, Ralph H. Hruban, Jianhong Hu, Taobo Hu, Xing Hua, Kuan-lin Huang, Mei Huang, Mi Ni Huang, Vincent Huang, Yi Huang, Wolfgang Huber, Thomas J. Hudson, Michael Hummel, Jillian A. Hung, David Huntsman, Ted R. Hupp, Jason Huse, Matthew R. Huska, Barbara Hutter, Carolyn M. Hutter, Daniel Hübschmann, Christine A. Iacobuzio-Donahue, Charles David Imbusch, Marcin Imielinski, Seiya Imoto, William B. Isaacs, Keren Isaev, Shumpei Ishikawa, Murat Iskar, S. M. Ashiqul Islam, Michael Ittmann, Sinisa Ivkovic, Jose M. G. Izarzugaza, Jocelyne Jacquemier, Valerie Jakrot, Nigel B. Jamieson, Gun Ho Jang, Se Jin Jang, Joy C. Jayaseelan, Reyka Jayasinghe, Stuart R. Jefferys, Karine Jegalian, Jennifer L. Jennings, Seung-Hyup Jeon, Lara Jerman, Yuan Ji, Wei Jiao, Peter A. Johansson, Amber L. Johns, Jeremy Johns, Rory Johnson, Todd A. Johnson, Clemency Jolly, Yann Joly, Jon G. Jonasson, Corbin D. Jones, David R. Jones, David T. W. Jones, Nic Jones, Steven J. M. Jones, Jos Jonkers, Young Seok Ju, Hartmut Juhl, Jongsun Jung, Malene Juul, Randi Istrup Juul, Sissel Juul, Natalie Jäger, Rolf Kabbe, Andre Kahles, Abdullah Kahraman, Vera B. Kaiser, Hojabr Kakavand, Sangeetha Kalimuthu, Christof von Kalle, Koo Jeong Kang, Katalin Karaszi, Beth Karlan, Rosa Karlić, Dennis Karsch, Katayoon Kasaian, Karin S. Kassahn, Hitoshi Katai, Mamoru Kato, Hiroto Katoh, Yoshiiku Kawakami, Jonathan D. Kay, Stephen H. Kazakoff, Marat D. Kazanov, Maria Keays, Electron Kebebew, Richard F. Kefford, Manolis Kellis, James G. Kench, Catherine J. Kennedy, Jules N. A. Kerssemakers, David Khoo, Vincent Khoo, Narong Khuntikeo, Ekta Khurana, Helena Kilpinen, Hark Kyun Kim, Hyung-Lae Kim, Hyung-Yong Kim, Hyunghwan Kim, Jaegil Kim, Jihoon Kim, Jong K. Kim, Youngwook Kim, Tari A. King, Wolfram Klapper, Kortine Kleinheinz, Leszek J. Klimczak, Stian Knappskog, Michael Kneba, Bartha M. Knoppers, Youngil Koh, Jan Komorowski, Daisuke Komura, Mitsuhiro Komura, Gu Kong, Marcel Kool, Jan O. Korbel, Viktoriya Korchina, Andrey Korshunov, Michael Koscher, Roelof Koster, Zsofia Kote-Jarai, Antonios Koures, Milena Kovacevic, Barbara Kremeyer, Helene Kretzmer, Markus Kreuz, Savitri Krishnamurthy, Dieter Kube, Kiran Kumar, Pardeep Kumar, Sushant Kumar, Yogesh Kumar, Ritika Kundra, Kirsten Kübler, Ralf Küppers, Jesper Lagergren, Phillip H. Lai, Peter W. Laird, Sunil R. Lakhani, Christopher M. Lalansingh, Emilie Lalonde, Fabien C. Lamaze, Adam Lambert, Eric Lander, Pablo Landgraf, Luca Landoni, Anita Langerød, Andrés Lanzós, Denis Larsimont, Erik Larsson, Mark Lathrop, Loretta M. S. Lau, Chris Lawerenz, Rita T. Lawlor, Michael S. Lawrence, Alexander J. Lazar, Ana Mijalkovic Lazic, Xuan Le, Darlene Lee, Donghoon Lee, Eunjung Alice Lee, Hee Jin Lee, Jake June-Koo Lee, Jeong-Yeon Lee, Juhee Lee, Ming Ta Michael Lee, Henry Lee-Six, Kjong-Van Lehmann, Hans Lehrach, Dido Lenze, Conrad R. Leonard, Daniel A. Leongamornlert, Ignaty Leshchiner, Louis Letourneau, Ivica Letunic, Douglas A. Levine, Lora Lewis, Tim Ley, Chang Li, Constance H. Li, Haiyan Irene Li, Jun Li, Lin Li, Shantao Li, Siliang Li, Xiaobo Li, Xiaotong Li, Xinyue Li, Yilong Li, Han Liang, Sheng-Ben Liang, Peter Lichter, Pei Lin, Ziao Lin, W. M. Linehan, Ole Christian Lingjærde, Dongbing Liu, Eric Minwei Liu, Fei-Fei Fei Liu, Fenglin Liu, Jia Liu, Xingmin Liu, Julie Livingstone, Dimitri Livitz, Naomi Livni, Lucas Lochovsky, Markus Loeffler, Georgina V. Long, Armando Lopez-Guillermo, Shaoke Lou, David N. Louis, Laurence B. Lovat, Yiling Lu, Yong-Jie Lu, Youyong Lu, Claudio Luchini, Ilinca Lungu, Xuemei Luo, Hayley J. Luxton, Andy G. Lynch, Lisa Lype, Cristina López, Carlos López-Otín, Eric Z. Ma, Yussanne Ma, Gaetan MacGrogan, Shona MacRae, Geoff Macintyre, Tobias Madsen, Kazuhiro Maejima, Andrea Mafficini, Dennis T. Maglinte, Arindam Maitra, Partha P. Majumder, Luca Malcovati, Salem Malikic, Giuseppe Malleo, Graham J. Mann, Luisa Mantovani-Löffler, Kathleen Marchal, Giovanni Marchegiani, Elaine R. Mardis, Adam A. Margolin, Maximillian G. Marin, Florian Markowetz, Julia Markowski, Jeffrey Marks, Tomas Marques-Bonet, Marco A. Marra, Luke Marsden, John W. M. Martens, Sancha Martin, Jose I. Martin-Subero, Iñigo Martincorena, Alexander Martinez-Fundichely, Yosef E. Maruvka, R. Jay Mashl, Charlie E. Massie, Thomas J. Matthew, Lucy Matthews, Erik Mayer, Simon Mayes, Michael Mayo, Faridah Mbabaali, Karen McCune, Ultan McDermott, Patrick D. McGillivray, Michael D. McLellan, John D. McPherson, John R. McPherson, Treasa A. McPherson, Samuel R. Meier, Alice Meng, Shaowu Meng, Andrew Menzies, Neil D. Merrett, Sue Merson, Matthew Meyerson, William Meyerson, Piotr A. Mieczkowski, George L. Mihaiescu, Sanja Mijalkovic, Tom Mikkelsen, Michele Milella, Linda Mileshkin, Christopher A. Miller, David K. Miller, Jessica K. Miller, Gordon B. Mills, Ana Milovanovic, Sarah Minner, Marco Miotto, Gisela Mir Arnau, Lisa Mirabello, Chris Mitchell, Thomas J. Mitchell, Satoru Miyano, Naoki Miyoshi, Shinichi Mizuno, Fruzsina Molnár-Gábor, Malcolm J. Moore, Richard A. Moore, Sandro Morganella, Quaid D. Morris, Carl Morrison, Lisle E. Mose, Catherine D. Moser, Ferran Muiños, Loris Mularoni, Andrew J. Mungall, Karen Mungall, Elizabeth A. Musgrove, Ville Mustonen, David Mutch, Francesc Muyas, Donna M. Muzny, Alfonso Muñoz, Jerome Myers, Ola Myklebost, Peter Möller, Genta Nagae, Adnan M. Nagrial, Hardeep K. Nahal-Bose, Hitoshi Nakagama, Hidewaki Nakagawa, Hiromi Nakamura, Toru Nakamura, Kaoru Nakano, Tannistha Nandi, Jyoti Nangalia, Mia Nastic, Arcadi Navarro, Fabio C. P. Navarro, David E. Neal, Gerd Nettekoven, Felicity Newell, Steven J. Newhouse, Yulia Newton, Alvin Wei Tian Ng, Anthony Ng, Jonathan Nicholson, David Nicol, Yongzhan Nie, G. Petur Nielsen, Morten Muhlig Nielsen, Serena Nik-Zainal, Michael S. Noble, Katia Nones, Paul A. Northcott, Faiyaz Notta, Brian D. O’Connor, Peter O’Donnell, Maria O’Donovan, Sarah O’Meara, Brian Patrick O’Neill, J. Robert O’Neill, David Ocana, Angelica Ochoa, Layla Oesper, Christopher Ogden, Hideki Ohdan, Kazuhiro Ohi, Lucila Ohno-Machado, Karin A. Oien, Akinyemi I. Ojesina, Hidenori Ojima, Takuji Okusaka, Larsson Omberg, Choon Kiat Ong, Stephan Ossowski, German Ott, B. F. Francis Ouellette, Christine P’ng, Marta Paczkowska, Salvatore Paiella, Chawalit Pairojkul, Marina Pajic, Qiang Pan-Hammarström, Elli Papaemmanuil, Irene Papatheodorou, Nagarajan Paramasivam, Ji Wan Park, Joong-Won Park, Keunchil Park, Kiejung Park, Peter J. Park, Joel S. Parker, Simon L. Parsons, Harvey Pass, Danielle Pasternack, Alessandro Pastore, Ann-Marie Patch, Iris Pauporté, Antonio Pea, John V. Pearson, Chandra Sekhar Pedamallu, Jakob Skou Pedersen, Paolo Pederzoli, Martin Peifer, Nathan A. Pennell, Charles M. Perou, Marc D. Perry, Gloria M. Petersen, Myron Peto, Nicholas Petrelli, Robert Petryszak, Stefan M. Pfister, Mark Phillips, Oriol Pich, Hilda A. Pickett, Todd D. Pihl, Nischalan Pillay, Sarah Pinder, Mark Pinese, Andreia V. Pinho, Esa Pitkänen, Xavier Pivot, Elena Piñeiro-Yáñez, Laura Planko, Christoph Plass, Paz Polak, Tirso Pons, Irinel Popescu, Olga Potapova, Aparna Prasad, Shaun R. Preston, Manuel Prinz, Antonia L. Pritchard, Stephenie D. Prokopec, Elena Provenzano, Xose S. Puente, Sonia Puig, Montserrat Puiggròs, Sergio Pulido-Tamayo, Gulietta M. Pupo, Colin A. Purdie, Michael C. Quinn, Raquel Rabionet, Janet S. Rader, Bernhard Radlwimmer, Petar Radovic, Benjamin Raeder, Keiran M. Raine, Manasa Ramakrishna, Kamna Ramakrishnan, Suresh Ramalingam, Benjamin J. Raphael, W. Kimryn Rathmell, Tobias Rausch, Guido Reifenberger, Jüri Reimand, Jorge Reis-Filho, Victor Reuter, Iker Reyes-Salazar, Matthew A. Reyna, Sheila M. Reynolds, Esther Rheinbay, Yasser Riazalhosseini, Andrea L. Richardson, Julia Richter, Matthew Ringel, Markus Ringnér, Yasushi Rino, Karsten Rippe, Jeffrey Roach, Lewis R. Roberts, Nicola D. Roberts, Steven A. Roberts, A. Gordon Robertson, Alan J. Robertson, Javier Bartolomé Rodriguez, Bernardo Rodriguez-Martin, F. Germán Rodríguez-González, Michael H. A. Roehrl, Marius Rohde, Hirofumi Rokutan, Gilles Romieu, Ilse Rooman, Tom Roques, Daniel Rosebrock, Mara Rosenberg, Philip C. Rosenstiel, Andreas Rosenwald, Edward W. Rowe, Romina Royo, Steven G. Rozen, Yulia Rubanova, Mark A. Rubin, Carlota Rubio-Perez, Vasilisa A. Rudneva, Borislav C. Rusev, Andrea Ruzzenente, Gunnar Rätsch, Radhakrishnan Sabarinathan, Veronica Y. Sabelnykova, Sara Sadeghi, S. Cenk Sahinalp, Natalie Saini, Mihoko Saito-Adachi, Gordon Saksena, Adriana Salcedo, Roberto Salgado, Leonidas Salichos, Richard Sallari, Charles Saller, Roberto Salvia, Michelle Sam, Jaswinder S. Samra, Francisco Sanchez-Vega, Chris Sander, Grant Sanders, Rajiv Sarin, Iman Sarrafi, Aya Sasaki-Oku, Torill Sauer, Guido Sauter, Robyn P. M. Saw, Maria Scardoni, Christopher J. Scarlett, Aldo Scarpa, Ghislaine Scelo, Dirk Schadendorf, Jacqueline E. Schein, Markus B. Schilhabel, Matthias Schlesner, Thorsten Schlomm, Heather K. Schmidt, Sarah-Jane Schramm, Stefan Schreiber, Nikolaus Schultz, Steven E. Schumacher, Roland F. Schwarz, Richard A. Scolyer, David Scott, Ralph Scully, Raja Seethala, Ayellet V. Segre, Iris Selander, Colin A. Semple, Yasin Senbabaoglu, Subhajit Sengupta, Elisabetta Sereni, Stefano Serra, Dennis C. Sgroi, Mark Shackleton, Nimish C. Shah, Sagedeh Shahabi, Catherine A. Shang, Ping Shang, Ofer Shapira, Troy Shelton, Ciyue Shen, Hui Shen, Rebecca Shepherd, Ruian Shi, Yan Shi, Yu-Jia Shiah, Tatsuhiro Shibata, Juliann Shih, Eigo Shimizu, Kiyo Shimizu, Seung Jun Shin, Yuichi Shiraishi, Tal Shmaya, Ilya Shmulevich, Solomon I. Shorser, Charles Short, Raunak Shrestha, Suyash S. Shringarpure, Craig Shriver, Shimin Shuai, Nikos Sidiropoulos, Reiner Siebert, Anieta M. Sieuwerts, Lina Sieverling, Sabina Signoretti, Katarzyna O. Sikora, Michele Simbolo, Ronald Simon, Janae V. Simons, Jared T. Simpson, Peter T. Simpson, Samuel Singer, Nasa Sinnott-Armstrong, Payal Sipahimalani, Tara J. Skelly, Marcel Smid, Jaclyn Smith, Karen Smith-McCune, Nicholas D. Socci, Heidi J. Sofia, Matthew G. Soloway, Lei Song, Anil K. Sood, Sharmila Sothi, Christos Sotiriou, Cameron M. Soulette, Paul N. Span, Paul T. Spellman, Nicola Sperandio, Andrew J. Spillane, Oliver Spiro, Jonathan Spring, Johan Staaf, Peter F. Stadler, Peter Staib, Stefan G. Stark, Lucy Stebbings, Ólafur Andri Stefánsson, Oliver Stegle, Lincoln D. Stein, Alasdair Stenhouse, Chip Stewart, Stephan Stilgenbauer, Miranda D. Stobbe, Michael R. Stratton, Jonathan R. Stretch, Adam J. Struck, Joshua M. Stuart, Henk G. Stunnenberg, Hong Su, Xiaoping Su, Ren X. Sun, Stephanie Sungalee, Hana Susak, Akihiro Suzuki, Fred Sweep, Monika Szczepanowski, Holger Sültmann, Takashi Yugawa, Angela Tam, David Tamborero, Benita Kiat Tee Tan, Donghui Tan, Patrick Tan, Hiroko Tanaka, Hirokazu Taniguchi, Tomas J. Tanskanen, Maxime Tarabichi, Roy Tarnuzzer, Patrick Tarpey, Morgan L. Taschuk, Kenji Tatsuno, Simon Tavaré, Darrin F. Taylor, Amaro Taylor-Weiner, Jon W. Teague, Bin Tean Teh, Varsha Tembe, Javier Temes, Kevin Thai, Sarah P. Thayer, Nina Thiessen, Gilles Thomas, Sarah Thomas, Alan Thompson, Alastair M. Thompson, John F. F. Thompson, R. Houston Thompson, Heather Thorne, Leigh B. Thorne, Adrian Thorogood, Grace Tiao, Nebojsa Tijanic, Lee E. Timms, Roberto Tirabosco, Marta Tojo, Stefania Tommasi, Christopher W. Toon, Umut H. Toprak, David Torrents, Giampaolo Tortora, Jörg Tost, Yasushi Totoki, David Townend, Nadia Traficante, Isabelle Treilleux, Jean-Rémi Trotta, Lorenz H. P. Trümper, Ming Tsao, Tatsuhiko Tsunoda, Jose M. C. Tubio, Olga Tucker, Richard Turkington, Daniel J. Turner, Andrew Tutt, Masaki Ueno, Naoto T. Ueno, Christopher Umbricht, Husen M. Umer, Timothy J. Underwood, Lara Urban, Tomoko Urushidate, Tetsuo Ushiku, Liis Uusküla-Reimand, Alfonso Valencia, David J. Van Den Berg, Steven Van Laere, Peter Van Loo, Erwin G. Van Meir, Gert G. Van den Eynden, Theodorus Van der Kwast, Naveen Vasudev, Miguel Vazquez, Ravikiran Vedururu, Umadevi Veluvolu, Shankar Vembu, Lieven P. C. Verbeke, Peter Vermeulen, Clare Verrill, Alain Viari, David Vicente, Caterina Vicentini, K. VijayRaghavan, Juris Viksna, Ricardo E. Vilain, Izar Villasante, Anne Vincent-Salomon, Tapio Visakorpi, Douglas Voet, Paresh Vyas, Ignacio Vázquez-García, Nick M. Waddell, Nicola Waddell, Claes Wadelius, Lina Wadi, Rabea Wagener, Jeremiah A. Wala, Jian Wang, Jiayin Wang, Linghua Wang, Qi Wang, Wenyi Wang, Yumeng Wang, Zhining Wang, Paul M. Waring, Hans-Jörg Warnatz, Jonathan Warrell, Anne Y. Warren, Sebastian M. Waszak, David C. Wedge, Dieter Weichenhan, Paul Weinberger, John N. Weinstein, Joachim Weischenfeldt, Daniel J. Weisenberger, Ian Welch, Michael C. Wendl, Johannes Werner, Justin P. Whalley, David A. Wheeler, Hayley C. Whitaker, Dennis Wigle, Matthew D. Wilkerson, Ashley Williams, James S. Wilmott, Gavin W. Wilson, Julie M. Wilson, Richard K. Wilson, Boris Winterhoff, Jeffrey A. Wintersinger, Maciej Wiznerowicz, Stephan Wolf, Bernice H. Wong, Tina Wong, Winghing Wong, Youngchoon Woo, Scott Wood, Bradly G. Wouters, Adam J. Wright, Derek W. Wright, Mark H. Wright, Chin-Lee Wu, Dai-Ying Wu, Guanming Wu, Jianmin Wu, Kui Wu, Yang Wu, Zhenggang Wu, Liu Xi, Tian Xia, Qian Xiang, Xiao Xiao, Rui Xing, Heng Xiong, Qinying Xu, Yanxun Xu, Hong Xue, Shinichi Yachida, Sergei Yakneen, Rui Yamaguchi, Takafumi N. Yamaguchi, Masakazu Yamamoto, Shogo Yamamoto, Hiroki Yamaue, Fan Yang, Huanming Yang, Jean Y. Yang, Liming Yang, Lixing Yang, Shanlin Yang, Tsun-Po Yang, Yang Yang, Xiaotong Yao, Marie-Laure Yaspo, Lucy Yates, Christina Yau, Chen Ye, Kai Ye, Venkata D. Yellapantula, Christopher J. Yoon, Sung-Soo Yoon, Fouad Yousif, Jun Yu, Kaixian Yu, Willie Yu, Yingyan Yu, Ke Yuan, Yuan Yuan, Denis Yuen, Christina K. Yung, Olga Zaikova, Jorge Zamora, Marc Zapatka, Jean C. Zenklusen, Thorsten Zenz, Nikolajs Zeps, Cheng-Zhong Zhang, Fan Zhang, Hailei Zhang, Hongwei Zhang, Hongxin Zhang, Jiashan Zhang, Jing Zhang, Junjun Zhang, Xiuqing Zhang, Xuanping Zhang, Yan Zhang, Zemin Zhang, Zhongming Zhao, Liangtao Zheng, Xiuqing Zheng, Wanding Zhou, Yong Zhou, Bin Zhu, Hongtu Zhu, Jingchun Zhu, Shida Zhu, Lihua Zou, Xueqing Zou, Anna deFazio, Nicholas van As, Carolien H. M. van Deurzen, Marc J. van de Vijver, L. van’t Veer, Christian von Mering

**Affiliations:** 1 Applied Tumor Genomics Research Program, Research Programs Unit, University of Helsinki, Helsinki, Finland; 2 Wellcome Sanger Institute, Wellcome Genome Campus, Hinxton, UK; 3 Memorial Sloan Kettering Cancer Center, New York, NY USA; 4 Genome Science Division, Research Center for Advanced Science and Technology, University of Tokyo, Tokyo, Japan; 5 Department of Surgery, University of Chicago, Chicago, IL USA; 6 Department of Surgery, Division of Hepatobiliary and Pancreatic Surgery, School of Medicine, Keimyung University Dongsan Medical Center, Daegu, South Korea; 7 Department of Oncology, Gil Medical Center, Gachon University, Incheon, South Korea; 8 Hiroshima University, Hiroshima, Japan; 9 Department of Bioinformatics and Computational Biology, The University of Texas MD Anderson Cancer Center, Houston, TX USA; 10 University of Texas MD Anderson Cancer Center, Houston, TX USA; 11 King Faisal Specialist Hospital and Research Centre, Al Maather, Riyadh, Saudi Arabia; 12 Bioinformatics Unit, Spanish National Cancer Research Centre (CNIO), Madrid, Spain; 13 Bioinformatics Core Facility, University Medical Center Hamburg, Hamburg, Germany; 14 Heinrich Pette Institute, Leibniz Institute for Experimental Virology, Hamburg, Germany; 15 Ontario Tumour Bank, Ontario Institute for Cancer Research, Toronto, ON Canada; 16 Department of Pathology, The University of Texas MD Anderson Cancer Center, Houston, TX USA; 17 Laboratory of Pathology, Center for Cancer Research, National Cancer Institute, Bethesda, MD USA; 18 Department of Cellular and Molecular Medicine and Department of Bioengineering, University of California San Diego, La Jolla, CA USA; 19 UC San Diego Moores Cancer Center, San Diego, CA USA; 20 Canada’s Michael Smith Genome Sciences Centre, BC Cancer, Vancouver, BC Canada; 21 Sir Peter MacCallum Department of Oncology, Peter MacCallum Cancer Centre, University of Melbourne, Melbourne, VIC Australia; 22 Centre for Research in Molecular Medicine and Chronic Diseases (CiMUS), Universidade de Santiago de Compostela, Santiago de Compostela, Spain; 23 Department of Zoology, Genetics and Physical Anthropology, (CiMUS), Universidade de Santiago de Compostela, Santiago de Compostela, Spain; 24 The Biomedical Research Centre (CINBIO), Universidade de Vigo, Vigo, Spain; 25 Royal National Orthopaedic Hospital - Bolsover, London, UK; 26 Department of Genomic Medicine, The University of Texas MD Anderson Cancer Center, Houston, TX USA; 27 Quantitative and Computational Biosciences Graduate Program, Baylor College of Medicine, Houston, TX USA; 28 The Jackson Laboratory for Genomic Medicine, Farmington, CT USA; 29 Genome Informatics Program, Ontario Institute for Cancer Research, Toronto, ON Canada; 30 Institute of Human Genetics, Christian-Albrechts-University, Kiel, Germany; 31 Institute of Human Genetics, Ulm University and Ulm University Medical Center, Ulm, Germany; 32 Queensland Centre for Medical Genomics, Institute for Molecular Bioscience, University of Queensland, St. Lucia, Brisbane, QLD Australia; 33 Salford Royal NHS Foundation Trust, Salford, UK; 34 Department of Surgery, Pancreas Institute, University and Hospital Trust of Verona, Verona, Italy; 35 Molecular and Medical Genetics, OHSU Knight Cancer Institute, Oregon Health and Science University, Portland, OR USA; 36 Department of Molecular Oncology, BC Cancer Research Centre, Vancouver, BC Canada; 37 The McDonnell Genome Institute at Washington University, St. Louis, MO USA; 38 University College London, London, UK; 39 Division of Cancer Genomics, National Cancer Center Research Institute, National Cancer Center, Tokyo, Japan; 40 DLR Project Management Agency, Bonn, Germany; 41 Tokyo Women’s Medical University, Tokyo, Japan; 42 Center for Molecular Oncology, Memorial Sloan Kettering Cancer Center, New York, NY USA; 43 Los Alamos National Laboratory, Los Alamos, NM USA; 44 Department of Pathology, University Health Network, Toronto General Hospital, Toronto, ON Canada; 45 Nottingham University Hospitals NHS Trust, Nottingham, UK; 46 Epigenomics and Cancer Risk Factors, German Cancer Research Center (DKFZ), Heidelberg, Germany; 47 Computational Biology Program, Ontario Institute for Cancer Research, Toronto, ON Canada; 48 Department of Molecular Genetics, University of Toronto, Toronto, ON Canada; 49 Vector Institute, Toronto, ON Canada; 50 Hematopathology Section, Institute of Pathology, Christian-Albrechts-University, Kiel, Germany; 51 Department of Pathology and Laboratory Medicine, School of Medicine, University of North Carolina at Chapel Hill, Chapel Hill, NC USA; 52 Department of Cancer Genetics, Institute for Cancer Research, Oslo University Hospital, The Norwegian Radium Hospital, Oslo, Norway; 53 Pathology, Hospital Clinic, Institut d’Investigacions Biomèdiques August Pi i Sunyer (IDIBAPS), University of Barcelona, Barcelona, Spain; 54 Department of Veterinary Medicine, Transmissible Cancer Group, University of Cambridge, Cambridge, UK; 55 Alvin J. Siteman Cancer Center, Washington University School of Medicine, St. Louis, MO USA; 56 Wolfson Wohl Cancer Research Centre, Institute of Cancer Sciences, University of Glasgow, Glasgow, UK; 57 Lineberger Comprehensive Cancer Center, University of North Carolina at Chapel Hill, Chapel Hill, NC USA; 58 Broad Institute of MIT and Harvard, Cambridge, MA USA; 59 Dana-Farber/Boston Children’s Cancer and Blood Disorders Center, Boston, MA USA; 60 Department of Pediatrics, Harvard Medical School, Boston, MA USA; 61 Leeds Institute of Medical Research @ St. James’s, University of Leeds, St. James’s University Hospital, Leeds, UK; 62 Department of Pathology and Diagnostics, University and Hospital Trust of Verona, Verona, Italy; 63 Department of Surgery, Princess Alexandra Hospital, Brisbane, QLD Australia; 64 Surgical Oncology Group, Diamantina Institute, University of Queensland, Brisbane, QLD Australia; 65 Department of Population and Quantitative Health Sciences, Case Western Reserve University School of Medicine, Cleveland, OH USA; 66 Research Health Analytics and Informatics, University Hospitals Cleveland Medical Center, Cleveland, OH USA; 67 Gloucester Royal Hospital, Gloucester, UK; 68 European Molecular Biology Laboratory, European Bioinformatics Institute (EMBL-EBI), Cambridge, UK; 69 Diagnostic Development, Ontario Institute for Cancer Research, Toronto, ON Canada; 70 Barcelona Supercomputing Center (BSC), Barcelona, Spain; 71 Arnie Charbonneau Cancer Institute, University of Calgary, Calgary, AB Canada; 72 Departments of Surgery and Oncology, University of Calgary, Calgary, AB Canada; 73 Department of Pathology, Oslo University Hospital, The Norwegian Radium Hospital, Oslo, Norway; 74 PanCuRx Translational Research Initiative, Ontario Institute for Cancer Research, Toronto, ON Canada; 75 Department of Oncology, Sidney Kimmel Comprehensive Cancer Center at Johns Hopkins University School of Medicine, Baltimore, MD USA; 76 University Hospital Southampton NHS Foundation Trust, Southampton, UK; 77 Royal Stoke University Hospital, Stoke-on-Trent, UK; 78 Genome Sequence Informatics, Ontario Institute for Cancer Research, Toronto, ON Canada; 79 Human Longevity Inc, San Diego, CA USA; 80 Olivia Newton-John Cancer Research Institute, La Trobe University, Heidelberg, VIC Australia; 81 Computer Network Information Center, Chinese Academy of Sciences, Beijing, China; 82 Genome Canada, Ottawa, ON Canada; 83 CNAG-CRG, Centre for Genomic Regulation (CRG), Barcelona Institute of Science and Technology (BIST), Barcelona, Spain; 84 Universitat Pompeu Fabra (UPF), Barcelona, Spain; 85 Buck Institute for Research on Aging, Novato, CA USA; 86 Duke University Medical Center, Durham, NC USA; 87 Department of Human Genetics, Hannover Medical School, Hannover, Germany; 88 Center for Bioinformatics and Functional Genomics, Cedars-Sinai Medical Center, Los Angeles, CA USA; 89 Department of Biomedical Sciences, Cedars-Sinai Medical Center, Los Angeles, CA USA; 90 The Hebrew University Faculty of Medicine, Jerusalem, Israel; 91 Barts Cancer Institute, Barts and the London School of Medicine and Dentistry, Queen Mary University of London, London, UK; 92 Department of Computer Science, Bioinformatics Group, University of Leipzig, Leipzig, Germany; 93 Interdisciplinary Center for Bioinformatics, University of Leipzig, Leipzig, Germany; 94 Transcriptome Bioinformatics, LIFE Research Center for Civilization Diseases, University of Leipzig, Leipzig, Germany; 95 Department of Medical Oncology, Dana-Farber Cancer Institute, Boston, MA USA; 96 Department of Cancer Biology, Dana-Farber Cancer Institute, Boston, MA USA; 97 Harvard Medical School, Boston, MA USA; 98 USC Norris Comprehensive Cancer Center, University of Southern California, Los Angeles, CA USA; 99 Department of Diagnostics and Public Health, University and Hospital Trust of Verona, Verona, Italy; 100 Department of Mathematics, Aarhus University, Aarhus, Denmark; 101 Department of Molecular Medicine (MOMA), Aarhus University Hospital, Aarhus N, Denmark; 102 Instituto Carlos Slim de la Salud, Mexico City, Mexico; 103 Department of Medical Biophysics, University of Toronto, Toronto, ON Canada; 104 Cancer Division, Garvan Institute of Medical Research, Kinghorn Cancer Centre, University of New South Wales (UNSW Sydney), Sydney, NSW Australia; 105 South Western Sydney Clinical School, Faculty of Medicine, University of New South Wales (UNSW Sydney), Liverpool, NSW Australia; 106 West of Scotland Pancreatic Unit, Glasgow Royal Infirmary, Glasgow, UK; 107 Center for Digital Health, Berlin Institute of Health and Charitè - Universitätsmedizin Berlin, Berlin, Germany; 108 Heidelberg Center for Personalized Oncology (DKFZ-HIPO), German Cancer Research Center (DKFZ), Heidelberg, Germany; 109 The Preston Robert Tisch Brain Tumor Center, Duke University Medical Center, Durham, NC USA; 110 Massachusetts General Hospital, Boston, MA USA; 111 National Institute of Biomedical Genomics, Kalyani, West Bengal India; 112 Institute of Clinical Medicine and Institute of Oral Biology, University of Oslo, Oslo, Norway; 113 University of North Carolina at Chapel Hill, Chapel Hill, NC USA; 114 ARC-Net Centre for Applied Research on Cancer, University and Hospital Trust of Verona, Verona, Italy; 115 The Institute of Cancer Research, London, UK; 116 Centre for Computational Biology, Duke-NUS Medical School, Singapore, Singapore; 117 Programme in Cancer and Stem Cell Biology, Duke-NUS Medical School, Singapore, Singapore; 118 Division of Oncology and Pathology, Department of Clinical Sciences Lund, Lund University, Lund, Sweden; 119 Department of Pediatric Oncology, Hematology and Clinical Immunology, Heinrich-Heine-University, Düsseldorf, Germany; 120 Laboratory for Medical Science Mathematics, RIKEN Center for Integrative Medical Sciences, Yokohama, Japan; 121 RIKEN Center for Integrative Medical Sciences, Yokohama, Japan; 122 Department of Internal Medicine/Hematology, Friedrich-Ebert-Hospital, Neumünster, Germany; 123 Departments of Dermatology and Pathology, Yale University, New Haven, CT USA; 124 Centre for Genomic Regulation (CRG), The Barcelona Institute of Science and Technology, Barcelona, Spain; 125 Radcliffe Department of Medicine, University of Oxford, Oxford, UK; 126 Canadian Center for Computational Genomics, McGill University, Montreal, QC Canada; 127 Department of Human Genetics, McGill University, Montreal, QC Canada; 128 Department of Human Genetics, University of California Los Angeles, Los Angeles, CA USA; 129 Department of Pharmacology, University of Toronto, Toronto, ON Canada; 130 Faculty of Medicine and Health Technology, Tampere University and Tays Cancer Center, Tampere University Hospital, Tampere, Finland; 131 Haematology, Leeds Teaching Hospitals NHS Trust, Leeds, UK; 132 Translational Research and Innovation, Centre Léon Bérard, Lyon, France; 133 Fox Chase Cancer Center, Philadelphia, PA USA; 134 International Agency for Research on Cancer, World Health Organization, Lyon, France; 135 Earlham Institute, Norwich, UK; 136 Norwich Medical School, University of East Anglia, Norwich, UK; 137 Department of Molecular Biology, Faculty of Science, Radboud Institute for Molecular Life Sciences, Radboud University, Nijmegen, HB The Netherlands; 138 CRUK Manchester Institute and Centre, Manchester, UK; 139 Department of Radiation Oncology, University of Toronto, Toronto, ON Canada; 140 Division of Cancer Sciences, Manchester Cancer Research Centre, University of Manchester, Manchester, UK; 141 Radiation Medicine Program, Princess Margaret Cancer Centre, Toronto, ON Canada; 142 Department of Pathology, Brigham and Women’s Hospital, Harvard Medical School, Boston, MA USA; 143 Department of Surgery, Division of Thoracic Surgery, The Johns Hopkins University School of Medicine, Baltimore, MD USA; 144 Division of Molecular Pathology, The Netherlands Cancer Institute, Oncode Institute, Amsterdam, CX The Netherlands; 145 Department of Biomolecular Engineering, University of California Santa Cruz, Santa Cruz, CA USA; 146 UC Santa Cruz Genomics Institute, University of California Santa Cruz, Santa Cruz, CA USA; 147 Division of Applied Bioinformatics, German Cancer Research Center (DKFZ), Heidelberg, Germany; 148 German Cancer Consortium (DKTK), German Cancer Research Center (DKFZ), Heidelberg, Germany; 149 National Center for Tumor Diseases (NCT) Heidelberg, Heidelberg, Germany; 150 Center for Biological Sequence Analysis, Department of Bio and Health Informatics, Technical University of Denmark, Lyngby, Denmark; 151 Novo Nordisk Foundation Center for Protein Research, University of Copenhagen, Copenhagen, Denmark; 152 Institute for Molecular Bioscience, University of Queensland, St. Lucia, Brisbane, QLD Australia; 153 Biomedical Engineering, Oregon Health and Science University, Portland, OR USA; 154 Division of Theoretical Bioinformatics, German Cancer Research Center (DKFZ), Heidelberg, Germany; 155 Institute of Pharmacy and Molecular Biotechnology and BioQuant, Heidelberg University, Heidelberg, Germany; 156 Federal Ministry of Education and Research, Berlin, Germany; 157 Melanoma Institute Australia, University of Sydney, Sydney, NSW Australia; 158 Pediatric Hematology and Oncology, University Hospital Muenster, Muenster, Germany; 159 Department of Pathology, Johns Hopkins University School of Medicine, Baltimore, MD USA; 160 McKusick-Nathans Institute of Genetic Medicine, Sidney Kimmel Comprehensive Cancer Center at Johns Hopkins University School of Medicine, Baltimore, MD USA; 161 Foundation Medicine, Inc, Cambridge, MA USA; 162 Department of Biomedical Data Science, Stanford University School of Medicine, Stanford, CA USA; 163 Department of Genetics, Stanford University School of Medicine, Stanford, CA USA; 164 Bakar Computational Health Sciences Institute and Department of Pediatrics, University of California, San Francisco, CA USA; 165 Institute of Clinical Medicine, Faculty of Medicine, University of Oslo, Oslo, Norway; 166 National Cancer Institute, National Institutes of Health, Bethesda, MD USA; 167 Royal Marsden NHS Foundation Trust, London and Sutton, UK; 168 Genome Biology Unit, European Molecular Biology Laboratory (EMBL), Heidelberg, Germany; 169 Department of Oncology, University of Cambridge, Cambridge, UK; 170 Li Ka Shing Centre, Cancer Research UK Cambridge Institute, University of Cambridge, Cambridge, UK; 171 Institut Gustave Roussy, Villejuif, France; 172 Cambridge University Hospitals NHS Foundation Trust, Cambridge, UK; 173 Department of Haematology, University of Cambridge, Cambridge, UK; 174 Anatomia Patológica, Hospital Clinic, Institut d’Investigacions Biomèdiques August Pi i Sunyer (IDIBAPS), University of Barcelona, Barcelona, Spain; 175 Spanish Ministry of Science and Innovation, Madrid, Spain; 176 University of Michigan Comprehensive Cancer Center, Ann Arbor, MI USA; 177 Department for BioMedical Research, University of Bern, Bern, Switzerland; 178 Department of Medical Oncology, Inselspital, University Hospital and University of Bern, Bern, Switzerland; 179 Graduate School for Cellular and Biomedical Sciences, University of Bern, Bern, Switzerland; 180 University of Pavia, Pavia, Italy; 181 University of Alabama at Birmingham, Birmingham, AL USA; 182 UHN Program in BioSpecimen Sciences, Toronto General Hospital, Toronto, ON Canada; 183 Department of Urology, Icahn School of Medicine at Mount Sinai, New York, NY USA; 184 Centre for Law and Genetics, University of Tasmania, Sandy Bay Campus, Hobart, TAS Australia; 185 Faculty of Biosciences, Heidelberg University, Heidelberg, Germany; 186 Department of Biochemistry, Microbiology and Immunology, Faculty of Medicine, University of Ottawa, Ottawa, ON Canada; 187 Division of Anatomic Pathology, Mayo Clinic, Rochester, MN USA; 188 Division of Cancer Epidemiology and Genetics, National Cancer Institute, National Institutes of Health, Bethesda, MD USA; 189 Illawarra Shoalhaven Local Health District L3 Illawarra Cancer Care Centre, Wollongong Hospital, Wollongong, NSW Australia; 190 BioForA, French National Institute for Agriculture, Food, and Environment (INRAE), ONF, Orléans, France; 191 Department of Biostatistics, Bloomberg School of Public Health, Johns Hopkins University, Baltimore, MD USA; 192 University of California San Diego, San Diego, CA USA; 193 Division of Experimental Pathology, Mayo Clinic, Rochester, MN USA; 194 Centre for Cancer Research, The Westmead Institute for Medical Research, University of Sydney, Sydney, NSW Australia; 195 Department of Gynaecological Oncology, Westmead Hospital, Sydney, NSW Australia; 196 PDXen Biosystems Inc, Seoul, South Korea; 197 Korea Advanced Institute of Science and Technology, Daejeon, South Korea; 198 Electronics and Telecommunications Research Institute, Daejeon, South Korea; 199 Institut National du Cancer (INCA), Boulogne-Billancourt, France; 200 Department of Genetics, Informatics Institute, University of Alabama at Birmingham, Birmingham, AL USA; 201 Division of Medical Oncology, National Cancer Centre, Singapore, Singapore; 202 Medical Oncology, University and Hospital Trust of Verona, Verona, Italy; 203 Department of Pediatrics, University Hospital Schleswig-Holstein, Kiel, Germany; 204 Hepatobiliary/Pancreatic Surgical Oncology Program, University Health Network, Toronto, ON Canada; 205 School of Biological Sciences, University of Auckland, Auckland, New Zealand; 206 Department of Surgery, University of Melbourne, Parkville, VIC Australia; 207 The Murdoch Children’s Research Institute, Royal Children’s Hospital, Parkville, VIC Australia; 208 Walter and Eliza Hall Institute, Parkville, VIC Australia; 209 Vancouver Prostate Centre, Vancouver, Canada; 210 Lunenfeld-Tanenbaum Research Institute, Mount Sinai Hospital, Toronto, ON Canada; 211 University of East Anglia, Norwich, UK; 212 Norfolk and Norwich University Hospital NHS Trust, Norwich, UK; 213 Victorian Institute of Forensic Medicine, Southbank, VIC Australia; 214 Department of Biomedical Informatics, Harvard Medical School, Boston, MA USA; 215 Department of Chemistry, Centre for Molecular Science Informatics, University of Cambridge, Cambridge, UK; 216 Ludwig Center at Harvard Medical School, Boston, MA USA; 217 Human Genome Sequencing Center, Baylor College of Medicine, Houston, TX USA; 218 Peter MacCallum Cancer Centre, University of Melbourne, Melbourne, VIC Australia; 219 Physics Division, Optimization and Systems Biology Lab, Massachusetts General Hospital, Boston, MA USA; 220 Department of Medicine, Baylor College of Medicine, Houston, TX USA; 221 University of Cologne, Cologne, Germany; 222 International Genomics Consortium, Phoenix, AZ USA; 223 Genomics Research Program, Ontario Institute for Cancer Research, Toronto, ON Canada; 224 Barking Havering and Redbridge University Hospitals NHS Trust, Romford, UK; 225 Children’s Hospital at Westmead, University of Sydney, Sydney, NSW Australia; 226 Department of Medicine, Section of Endocrinology, University and Hospital Trust of Verona, Verona, Italy; 227 Computational Biology Center, Memorial Sloan Kettering Cancer Center, New York, NY USA; 228 Department of Biology, ETH Zurich, Zürich, Switzerland; 229 Department of Computer Science, ETH Zurich, Zurich, Switzerland; 230 SIB Swiss Institute of Bioinformatics, Lausanne, Switzerland; 231 Weill Cornell Medical College, New York, NY USA; 232 Academic Department of Medical Genetics, University of Cambridge, Addenbrooke’s Hospital, Cambridge, UK; 233 MRC Cancer Unit, University of Cambridge, Cambridge, UK; 234 Departments of Pediatrics and Genetics, University of North Carolina at Chapel Hill, Chapel Hill, NC USA; 235 Seven Bridges Genomics, Charlestown, MA USA; 236 Annai Systems, Inc, Carlsbad, CA USA; 237 Department of Pathology, General Hospital of Treviso, Department of Medicine, University of Padua, Treviso, Italy; 238 Department of Computational Biology, University of Lausanne, Lausanne, Switzerland; 239 Department of Genetic Medicine and Development, University of Geneva Medical School, Geneva, CH Switzerland; 240 Swiss Institute of Bioinformatics, University of Geneva, Geneva, CH Switzerland; 241 The Francis Crick Institute, London, UK; 242 University of Leuven, Leuven, Belgium; 243 Institute of Medical Genetics and Applied Genomics, University of Tübingen, Tübingen, Germany; 244 Computational and Systems Biology, Genome Institute of Singapore, Singapore, Singapore; 245 School of Computing, National University of Singapore, Singapore, Singapore; 246 Big Data Institute, Li Ka Shing Centre, University of Oxford, Oxford, UK; 247 Biomedical Data Science Laboratory, Francis Crick Institute, London, UK; 248 Bioinformatics Group, Department of Computer Science, University College London, London, UK; 249 The Edward S. Rogers Sr. Department of Electrical and Computer Engineering, University of Toronto, Toronto, ON Canada; 250 Breast Cancer Translational Research Laboratory JC Heuson, Institut Jules Bordet, Brussels, Belgium; 251 Department of Oncology, Laboratory for Translational Breast Cancer Research, KU Leuven, Leuven, Belgium; 252 Institute for Research in Biomedicine (IRB Barcelona), The Barcelona Institute of Science and Technology, Barcelona, Spain; 253 Research Program on Biomedical Informatics, Universitat Pompeu Fabra, Barcelona, Spain; 254 Division of Medical Oncology, Princess Margaret Cancer Centre, Toronto, ON Canada; 255 Department of Physiology and Biophysics, Weill Cornell Medicine, New York, NY USA; 256 Institute for Computational Biomedicine, Weill Cornell Medicine, New York, NY USA; 257 Department of Pathology, UPMC Shadyside, Pittsburgh, PA USA; 258 Independent Consultant, Wellesley, USA; 259 Department of Cell and Molecular Biology, Science for Life Laboratory, Uppsala University, Uppsala, Sweden; 260 Department of Medicine and Department of Genetics, Washington University School of Medicine, St. Louis, St. Louis, MO USA; 261 Hefei University of Technology, Anhui, China; 262 Translational Cancer Research Unit, GZA Hospitals St.-Augustinus, Center for Oncological Research, Faculty of Medicine and Health Sciences, University of Antwerp, Antwerp, Belgium; 263 Simon Fraser University, Burnaby, BC Canada; 264 University of Pennsylvania, Philadelphia, PA USA; 265 Faculty of Science and Technology, University of Vic—Central University of Catalonia (UVic-UCC), Vic, Spain; 266 The Wellcome Trust, London, UK; 267 The Hospital for Sick Children, Toronto, ON Canada; 268 Department of Pathology, Queen Elizabeth University Hospital, Glasgow, UK; 269 Department of Genetics and Computational Biology, QIMR Berghofer Medical Research Institute, Brisbane, QLD Australia; 270 Department of Oncology, Centre for Cancer Genetic Epidemiology, University of Cambridge, Cambridge, UK; 271 Department of Public Health and Primary Care, Centre for Cancer Genetic Epidemiology, University of Cambridge, Cambridge, UK; 272 Prostate Cancer Canada, Toronto, ON Canada; 273 University of Cambridge, Cambridge, UK; 274 Department of Laboratory Medicine, Translational Cancer Research, Lund University Cancer Center at Medicon Village, Lund University, Lund, Sweden; 275 Heidelberg University, Heidelberg, Germany; 276 New BIH Digital Health Center, Berlin Institute of Health (BIH) and Charité - Universitätsmedizin Berlin, Berlin, Germany; 277 CIBER Epidemiología y Salud Pública (CIBERESP), Madrid, Spain; 278 Research Group on Statistics, Econometrics and Health (GRECS), UdG, Barcelona, Spain; 279 Quantitative Genomics Laboratories (qGenomics), Barcelona, Spain; 280 Icelandic Cancer Registry, Icelandic Cancer Society, Reykjavik, Iceland; 281 State Key Laboratory of Cancer Biology, and Xijing Hospital of Digestive Diseases, Fourth Military Medical University, Shaanxi, China; 282 Department of Medicine (DIMED), Surgical Pathology Unit, University of Padua, Padua, Italy; 283 Rigshospitalet, Copenhagen, Denmark; 284 Center for Cancer Genomics, National Cancer Institute, National Institutes of Health, Bethesda, MD USA; 285 Department of Biochemistry and Molecular Medicine, University of Montreal, Montreal, QC Canada; 286 Australian Institute of Tropical Health and Medicine, James Cook University, Douglas, QLD Australia; 287 Department of Neuro-Oncology, Istituto Neurologico Besta, Milano, Italy; 288 Bioplatforms Australia, North Ryde, NSW Australia; 289 Department of Pathology (Research), University College London Cancer Institute, London, UK; 290 Department of Surgical Oncology, Princess Margaret Cancer Centre, Toronto, ON Canada; 291 Department of Medical Oncology, Josephine Nefkens Institute and Cancer Genomics Centre, Erasmus Medical Center, Rotterdam, CN The Netherlands; 292 The University of Queensland Thoracic Research Centre, The Prince Charles Hospital, Brisbane, QLD Australia; 293 CIBIO/InBIO - Research Center in Biodiversity and Genetic Resources, Universidade do Porto, Vairão, Portugal; 294 HCA Laboratories, London, UK; 295 University of Liverpool, Liverpool, UK; 296 The Azrieli Faculty of Medicine, Bar-Ilan University, Safed, Israel; 297 Department of Neurosurgery, University of Florida, Gainesville, FL USA; 298 Department of Pathology, Graduate School of Medicine, University of Tokyo, Tokyo, Japan; 299 University of Milano Bicocca, Monza, Italy; 300 BGI-Shenzhen, Shenzhen, China; 301 Department of Pathology, Oslo University Hospital Ulleval, Oslo, Norway; 302 Center for Biomedical Informatics, Harvard Medical School, Boston, MA USA; 303 Department Biochemistry and Molecular Biomedicine, University of Barcelona, Barcelona, Spain; 304 Office of Cancer Genomics, National Cancer Institute, National Institutes of Health, Bethesda, MD USA; 305 Cancer Epigenomics, German Cancer Research Center (DKFZ), Heidelberg, Germany; 306 Department of Cancer Biology, The University of Texas MD Anderson Cancer Center, Houston, TX USA; 307 Department of Surgical Oncology, The University of Texas MD Anderson Cancer Center, Houston, TX USA; 308 Department of Computer Science, Yale University, New Haven, CT USA; 309 Department of Molecular Biophysics and Biochemistry, Yale University, New Haven, CT USA; 310 Program in Computational Biology and Bioinformatics, Yale University, New Haven, CT USA; 311 Center for Cancer Research, Massachusetts General Hospital, Boston, MA USA; 312 Department of Pathology, Massachusetts General Hospital, Boston, MA USA; 313 Department of Pathology, Memorial Sloan Kettering Cancer Center, New York, NY USA; 314 Division of Gastroenterology and Hepatology, Mayo Clinic, Rochester, MN USA; 315 University of Sydney, Sydney, NSW Australia; 316 University of Oxford, Oxford, UK; 317 Department of Surgery, Academic Urology Group, University of Cambridge, Cambridge, UK; 318 Department of Medicine II, University of Würzburg, Wuerzburg, Germany; 319 Sylvester Comprehensive Cancer Center, University of Miami, Miami, FL USA; 320 Institut Hospital del Mar d’Investigacions Mèdiques (IMIM), Barcelona, Spain; 321 Genome Integrity and Structural Biology Laboratory, National Institute of Environmental Health Sciences (NIEHS), Durham, NC USA; 322 St. Thomas’s Hospital, London, UK; 323 Osaka International Cancer Center, Osaka, Japan; 324 Department of Pathology, Skåne University Hospital, Lund University, Lund, Sweden; 325 Department of Medical Oncology, Beatson West of Scotland Cancer Centre, Glasgow, UK; 326 National Human Genome Research Institute, National Institutes of Health, Bethesda, MD USA; 327 Centre for Cancer Research, Victorian Comprehensive Cancer Centre, University of Melbourne, Melbourne, VIC Australia; 328 Department of Medicine, Section of Hematology/Oncology, University of Chicago, Chicago, IL USA; 329 German Center for Infection Research (DZIF), Partner Site Hamburg-Borstel-Lübeck-Riems, Hamburg, Germany; 330 Bioinformatics Research Centre (BiRC), Aarhus University, Aarhus, Denmark; 331 Department of Biotechnology, Ministry of Science and Technology, Government of India, New Delhi, Delhi India; 332 National Cancer Centre Singapore, Singapore, Singapore; 333 Brandeis University, Waltham, MA USA; 334 Department of Urologic Sciences, University of British Columbia, Vancouver, BC Canada; 335 Department of Internal Medicine, Stanford University, Stanford, CA USA; 336 The University of Texas Health Science Center at Houston, Houston, TX USA; 337 Imperial College NHS Trust, Imperial College, London, INY UK; 338 Senckenberg Institute of Pathology, University of Frankfurt Medical School, Frankfurt, Germany; 339 Department of Medicine, Division of Biomedical Informatics, UC San Diego School of Medicine, San Diego, CA USA; 340 Center for Precision Health, School of Biomedical Informatics, The University of Texas Health Science Center, Houston, TX USA; 341 Oxford Nanopore Technologies, New York, NY USA; 342 Institute of Medical Science, University of Tokyo, Tokyo, Japan; 343 Howard Hughes Medical Institute, University of California Santa Cruz, Santa Cruz, CA USA; 344 Wakayama Medical University, Wakayama, Japan; 345 Department of Internal Medicine, Division of Medical Oncology, Lineberger Comprehensive Cancer Center, University of North Carolina at Chapel Hill, Chapel Hill, NC USA; 346 University of Tennessee Health Science Center for Cancer Research, Memphis, TN USA; 347 Department of Histopathology, Salford Royal NHS Foundation Trust, Salford, UK; 348 Faculty of Biology, Medicine and Health, University of Manchester, Manchester, UK; 349 BIOPIC, ICG and College of Life Sciences, Peking University, Beijing, China; 350 Peking-Tsinghua Center for Life Sciences, Peking University, Beijing, China; 351 Children’s Hospital of Philadelphia, Philadelphia, PA USA; 352 Department of Bioinformatics and Computational Biology and Department of Systems Biology, The University of Texas MD Anderson Cancer Center, Houston, TX USA; 353 Karolinska Institute, Stockholm, Sweden; 354 The Donnelly Centre, University of Toronto, Toronto, ON Canada; 355 Department of Medical Genetics, College of Medicine, Hallym University, Chuncheon, South Korea; 356 Department of Experimental and Health Sciences, Institute of Evolutionary Biology (UPF-CSIC), Universitat Pompeu Fabra, Barcelona, Spain; 357 Health Data Science Unit, University Clinics, Heidelberg, Germany; 358 Massachusetts General Hospital Center for Cancer Research, Charlestown, MA USA; 359 Hokkaido University, Sapporo, Japan; 360 Department of Pathology and Clinical Laboratory, National Cancer Center Hospital, Tokyo, Japan; 361 Department of Genetics, University of North Carolina at Chapel Hill, Chapel Hill, NC USA; 362 Computational Biology, Leibniz Institute on Aging - Fritz Lipmann Institute (FLI), Jena, Germany; 363 University of Melbourne Centre for Cancer Research, Melbourne, VIC Australia; 364 University of Nebraska Medical Center, Omaha, NE USA; 365 Syntekabio Inc, Daejeon, South Korea; 366 Department of Pathology, Academic Medical Center, Amsterdam, AZ The Netherlands; 367 China National GeneBank-Shenzhen, Shenzhen, China; 368 Division of Molecular Genetics, German Cancer Research Center (DKFZ), Heidelberg, Germany; 369 Division of Life Science and Applied Genomics Center, Hong Kong University of Science and Technology, Clear Water Bay, Hong Kong, China; 370 Icahn School of Medicine at Mount Sinai, New York, NY USA; 371 Geneplus-Shenzhen, Shenzhen, China; 372 School of Computer Science and Technology, Xi’an Jiaotong University, Xi’an, China; 373 AbbVie, North Chicago, IL USA; 374 Institute of Pathology, Charité – University Medicine Berlin, Berlin, Germany; 375 Centre for Translational and Applied Genomics, British Columbia Cancer Agency, Vancouver, BC Canada; 376 Edinburgh Royal Infirmary, Edinburgh, UK; 377 Berlin Institute for Medical Systems Biology, Max Delbrück Center for Molecular Medicine, Berlin, Germany; 378 Department of Pediatric Immunology, Hematology and Oncology, University Hospital, Heidelberg, Germany; 379 German Cancer Research Center (DKFZ), Heidelberg, Germany; 380 Heidelberg Institute for Stem Cell Technology and Experimental Medicine (HI-STEM), Heidelberg, Germany; 381 Institute for Computational Biomedicine, Weill Cornell Medical College, New York, NY USA; 382 New York Genome Center, New York, NY USA; 383 Department of Urology, James Buchanan Brady Urological Institute, Johns Hopkins University School of Medicine, Baltimore, MD USA; 384 Department of Preventive Medicine, Graduate School of Medicine, The University of Tokyo, Tokyo, Japan; 385 Department of Molecular and Cellular Biology, Baylor College of Medicine, Houston, TX USA; 386 Department of Pathology and Immunology, Baylor College of Medicine, Houston, TX USA; 387 Michael E. DeBakey Veterans Affairs Medical Center, Houston, TX USA; 388 Technical University of Denmark, Lyngby, Denmark; 389 Department of Pathology, College of Medicine, Hanyang University, Seoul, South Korea; 390 Academic Unit of Surgery, School of Medicine, College of Medical, Veterinary and Life Sciences, University of Glasgow, Glasgow Royal Infirmary, Glasgow, UK; 391 Department of Pathology, Asan Medical Center, College of Medicine, Ulsan University, Songpa-gu, Seoul South Korea; 392 Science Writer, Garrett Park, MD USA; 393 International Cancer Genome Consortium (ICGC)/ICGC Accelerating Research in Genomic Oncology (ARGO) Secretariat, Ontario Institute for Cancer Research, Toronto, ON Canada; 394 University of Ljubljana, Ljubljana, Slovenia; 395 Department of Public Health Sciences, University of Chicago, Chicago, IL USA; 396 Research Institute, NorthShore University HealthSystem, Evanston, IL USA; 397 Department for Biomedical Research, University of Bern, Bern, Switzerland; 398 Centre of Genomics and Policy, McGill University and Génome Québec Innovation Centre, Montreal, QC Canada; 399 Carolina Center for Genome Sciences, University of North Carolina at Chapel Hill, Chapel Hill, NC USA; 400 Hopp Children’s Cancer Center (KiTZ), Heidelberg, Germany; 401 Pediatric Glioma Research Group, German Cancer Research Center (DKFZ), Heidelberg, Germany; 402 Cancer Research UK, London, UK; 403 Indivumed GmbH, Hamburg, Germany; 404 Genome Integration Data Center, Syntekabio, Inc, Daejeon, South Korea; 405 University Hospital Zurich, Zurich, Switzerland; 406 Clinical Bioinformatics, Swiss Institute of Bioinformatics, Geneva, Switzerland; 407 Institute for Pathology and Molecular Pathology, University Hospital Zurich, Zurich, Switzerland; 408 Institute of Molecular Life Sciences, University of Zurich, Zurich, Switzerland; 409 MRC Human Genetics Unit, MRC IGMM, University of Edinburgh, Edinburgh, UK; 410 Women’s Cancer Program at the Samuel Oschin Comprehensive Cancer Institute, Cedars-Sinai Medical Center, Los Angeles, CA USA; 411 Department of Biology, Bioinformatics Group, Division of Molecular Biology, Faculty of Science, University of Zagreb, Zagreb, Croatia; 412 Department for Internal Medicine II, University Hospital Schleswig-Holstein, Kiel, Germany; 413 Genetics and Molecular Pathology, SA Pathology, Adelaide, SA Australia; 414 Department of Gastric Surgery, National Cancer Center Hospital, Tokyo, Japan; 415 Department of Bioinformatics, Division of Cancer Genomics, National Cancer Center Research Institute, Tokyo, Japan; 416 A.A. Kharkevich Institute of Information Transmission Problems, Moscow, Russia; 417 Oncology and Immunology, Dmitry Rogachev National Research Center of Pediatric Hematology, Moscow, Russia; 418 Skolkovo Institute of Science and Technology, Moscow, Russia; 419 Department of Surgery, The George Washington University, School of Medicine and Health Science, Washington, DC USA; 420 Endocrine Oncology Branch, Center for Cancer Research, National Cancer Institute, National Institutes of Health, Bethesda, MD USA; 421 Melanoma Institute Australia, Macquarie University, Sydney, NSW Australia; 422 MIT Computer Science and Artificial Intelligence Laboratory, Massachusetts Institute of Technology, Cambridge, MA USA; 423 Tissue Pathology and Diagnostic Oncology, Royal Prince Alfred Hospital, Sydney, NSW Australia; 424 Cholangiocarcinoma Screening and Care Program and Liver Fluke and Cholangiocarcinoma Research Centre, Faculty of Medicine, Khon Kaen University, Khon Kaen, Thailand; 425 Controlled Department and Institution, New York, NY USA; 426 Englander Institute for Precision Medicine, Weill Cornell Medicine, New York, NY USA; 427 National Cancer Center, Gyeonggi, South Korea; 428 Department of Biochemistry, College of Medicine, Ewha Womans University, Seoul, South Korea; 429 Health Sciences Department of Biomedical Informatics, University of California San Diego, La Jolla, CA USA; 430 Research Core Center, National Cancer Centre Korea, Goyang-si, South Korea; 431 Department of Health Sciences and Technology, Sungkyunkwan University School of Medicine, Seoul, South Korea; 432 Samsung Genome Institute, Seoul, South Korea; 433 Breast Oncology Program, Dana-Farber/Brigham and Women’s Cancer Center, Boston, MA USA; 434 Department of Surgery, Memorial Sloan Kettering Cancer Center, New York, NY USA; 435 Division of Breast Surgery, Brigham and Women’s Hospital, Boston, MA USA; 436 Integrative Bioinformatics Support Group, National Institute of Environmental Health Sciences (NIEHS), Durham, NC USA; 437 Department of Clinical Science, University of Bergen, Bergen, Norway; 438 Center For Medical Innovation, Seoul National University Hospital, Seoul, South Korea; 439 Department of Internal Medicine, Seoul National University Hospital, Seoul, South Korea; 440 Institute of Computer Science, Polish Academy of Sciences, Warsawa, Poland; 441 Functional and Structural Genomics, German Cancer Research Center (DKFZ), Heidelberg, Germany; 442 Laboratory of Translational Genomics, Division of Cancer Epidemiology and Genetics, National Cancer Institute, , National Institutes of Health, Bethesda, MD USA; 443 Institute for Medical Informatics Statistics and Epidemiology, University of Leipzig, Leipzig, Germany; 444 Morgan Welch Inflammatory Breast Cancer Research Program and Clinic, The University of Texas MD Anderson Cancer Center, Houston, TX USA; 445 Department of Hematology and Oncology, Georg-Augusts-University of Göttingen, Göttingen, Germany; 446 Institute of Cell Biology (Cancer Research), University of Duisburg-Essen, Essen, Germany; 447 King’s College London and Guy’s and St. Thomas’ NHS Foundation Trust, London, UK; 448 Center for Epigenetics, Van Andel Research Institute, Grand Rapids, MI USA; 449 The University of Queensland Centre for Clinical Research, Royal Brisbane and Women’s Hospital, Herston, QLD Australia; 450 Department of Pediatric Oncology and Hematology, University of Cologne, Cologne, Germany; 451 University of Düsseldorf, Düsseldorf, Germany; 452 Department of Pathology, Institut Jules Bordet, Brussels, Belgium; 453 Institute of Biomedicine, Sahlgrenska Academy at University of Gothenburg, Gothenburg, Sweden; 454 Children’s Medical Research Institute, Sydney, NSW Australia; 455 ILSbio, LLC Biobank, Chestertown, MD USA; 456 Division of Genetics and Genomics, Boston Children’s Hospital, Harvard Medical School, Boston, MA USA; 457 Institute for Bioengineering and Biopharmaceutical Research (IBBR), Hanyang University, Seoul, South Korea; 458 Department of Statistics, University of California Santa Cruz, Santa Cruz, CA USA; 459 National Genotyping Center, Institute of Biomedical Sciences, Academia Sinica, Taipei, Taiwan; 460 Department of Vertebrate Genomics/Otto Warburg Laboratory Gene Regulation and Systems Biology of Cancer, Max Planck Institute for Molecular Genetics, Berlin, Germany; 461 McGill University and Genome Quebec Innovation Centre, Montreal, QC Canada; 462 biobyte solutions GmbH, Heidelberg, Germany; 463 Gynecologic Oncology, NYU Laura and Isaac Perlmutter Cancer Center, New York University, New York, NY USA; 464 Division of Oncology, Stem Cell Biology Section, Washington University School of Medicine, St. Louis, MO USA; 465 Department of Systems Biology, The University of Texas MD Anderson Cancer Center, Houston, TX USA; 466 Harvard University, Cambridge, MA USA; 467 Urologic Oncology Branch, Center for Cancer Research, National Cancer Institute, National Institutes of Health, Bethesda, MD USA; 468 University of Oslo, Oslo, Norway; 469 University of Toronto, Toronto, ON Canada; 470 Peking University, Beijing, China; 471 School of Life Sciences, Peking University, Beijing, China; 472 Leidos Biomedical Research, Inc, McLean, VA USA; 473 Hematology, Hospital Clinic, Institut d’Investigacions Biomèdiques August Pi i Sunyer (IDIBAPS), University of Barcelona, Barcelona, Spain; 474 Second Military Medical University, Shanghai, China; 475 Chinese Cancer Genome Consortium, Shenzhen, China; 476 Department of Medical Oncology, Beijing Hospital, Beijing, China; 477 Laboratory of Molecular Oncology, Key Laboratory of Carcinogenesis and Translational Research (Ministry of Education), Peking University Cancer Hospital and Institute, Beijing, China; 478 School of Medicine/School of Mathematics and Statistics, University of St. Andrews, St, Andrews, Fife UK; 479 Institute for Systems Biology, Seattle, WA USA; 480 Department of Biochemistry and Molecular Biology, Faculty of Medicine, University Institute of Oncology-IUOPA, Oviedo, Spain; 481 Institut Bergonié, Bordeaux, France; 482 Cancer Unit, MRC University of Cambridge, Cambridge, UK; 483 Department of Pathology and Laboratory Medicine, Center for Personalized Medicine, Children’s Hospital Los Angeles, Los Angeles, CA USA; 484 John Curtin School of Medical Research, Canberra, ACT Australia; 485 MVZ Department of Oncology, PraxisClinic am Johannisplatz, Leipzig, Germany; 486 Department of Information Technology, Ghent University, Ghent, Belgium; 487 Department of Plant Biotechnology and Bioinformatics, Ghent University, Ghent, Belgium; 488 Institute for Genomic Medicine, Nationwide Children’s Hospital, Columbus, OH USA; 489 Computational Biology Program, School of Medicine, Oregon Health and Science University, Portland, OR USA; 490 Department of Surgery, Duke University, Durham, NC USA; 491 Institució Catalana de Recerca i Estudis Avançats (ICREA), Barcelona, Spain; 492 Institut Català de Paleontologia Miquel Crusafont, Universitat Autònoma de Barcelona, Barcelona, Spain; 493 University of Glasgow, Glasgow, UK; 494 Institut d’Investigacions Biomèdiques August Pi i Sunyer (IDIBAPS), Barcelona, Spain; 495 Division of Oncology, Washington University School of Medicine, St. Louis, MO USA; 496 Department of Surgery and Cancer, Imperial College, London, INY UK; 497 Applications Department, Oxford Nanopore Technologies, Oxford, UK; 498 Department of Obstetrics, Gynecology and Reproductive Services, University of California San Francisco, San Francisco, CA USA; 499 Department of Biochemistry and Molecular Medicine, University California at Davis, Sacramento, CA USA; 500 STTARR Innovation Facility, Princess Margaret Cancer Centre, Toronto, ON Canada; 501 Discipline of Surgery, Western Sydney University, Penrith, NSW Australia; 502 Yale School of Medicine, Yale University, New Haven, CT USA; 503 Department of Genetics, Lineberger Comprehensive Cancer Center, University of North Carolina at Chapel Hill, Chapel Hill, NC USA; 504 Departments of Neurology and Neurosurgery, Henry Ford Hospital, Detroit, MI USA; 505 Precision Oncology, OHSU Knight Cancer Institute, Oregon Health and Science University, Portland, OR USA; 506 Institute of Pathology, University Medical Center Hamburg-Eppendorf, Hamburg, Germany; 507 Department of Health Sciences, Faculty of Medical Sciences, Kyushu University, Fukuoka, Japan; 508 Heidelberg Academy of Sciences and Humanities, Heidelberg, Germany; 509 Department of Clinical Pathology, University of Melbourne, Melbourne, VIC, Australia; 510 Department of Pathology, Roswell Park Cancer Institute, Buffalo, NY USA; 511 Department of Computer Science, University of Helsinki, Helsinki, Finland; 512 Institute of Biotechnology, University of Helsinki, Helsinki, Finland; 513 Organismal and Evolutionary Biology Research Programme, University of Helsinki, Helsinki, Finland; 514 Department of Obstetrics and Gynecology, Division of Gynecologic Oncology, Washington University School of Medicine, St. Louis, MO USA; 515 Penrose St. Francis Health Services, Colorado Springs, CO USA; 516 Institute of Pathology, Ulm University and University Hospital of Ulm, Ulm, Germany; 517 National Cancer Center, Tokyo, Japan; 518 Genome Institute of Singapore, Singapore, Singapore; 519 32Program in Computational Biology and Bioinformatics, Yale University, New Haven, CT USA; 520 German Cancer Aid, Bonn, Germany; 521 Programme in Cancer and Stem Cell Biology, Centre for Computational Biology, Duke-NUS Medical School, Singapore, Singapore; 522 The Chinese University of Hong Kong, Shatin, NT, Hong Kong China; 523 Fourth Military Medical University, Shaanxi, China; 524 The University of Cambridge School of Clinical Medicine, Cambridge, UK; 525 St. Jude Children’s Research Hospital, Memphis, TN USA; 526 University Health Network, Princess Margaret Cancer Centre, Toronto, ON Canada; 527 Center for Biomolecular Science and Engineering, University of California Santa Cruz, Santa Cruz, CA USA; 528 Department of Medicine, University of Chicago, Chicago, IL USA; 529 Department of Neurology, Mayo Clinic, Rochester, MN USA; 530 Cambridge Oesophagogastric Centre, Cambridge University Hospitals NHS Foundation Trust, Cambridge, UK; 531 Department of Computer Science, Carleton College, Northfield, MN USA; 532 Institute of Cancer Sciences, College of Medical Veterinary and Life Sciences, University of Glasgow, Glasgow, UK; 533 Department of Epidemiology, University of Alabama at Birmingham, Birmingham, AL USA; 534 HudsonAlpha Institute for Biotechnology, Huntsville, AL USA; 535 O’Neal Comprehensive Cancer Center, University of Alabama at Birmingham, Birmingham, AL USA; 536 Department of Pathology, Keio University School of Medicine, Tokyo, Japan; 537 Department of Hepatobiliary and Pancreatic Oncology, National Cancer Center Hospital, Tokyo, Japan; 538 Sage Bionetworks, Seattle, WA USA; 539 Lymphoma Genomic Translational Research Laboratory, National Cancer Centre, Singapore, Singapore; 540 Department of Clinical Pathology, Robert-Bosch-Hospital, Stuttgart, Germany; 541 Department of Cell and Systems Biology, University of Toronto, Toronto, ON Canada; 542 Department of Biosciences and Nutrition, Karolinska Institutet, Stockholm, Sweden; 543 Center for Liver Cancer, Research Institute and Hospital, National Cancer Center, Gyeonggi, South Korea; 544 Division of Hematology-Oncology, Samsung Medical Center, Sungkyunkwan University School of Medicine, Seoul, South Korea; 545 Samsung Advanced Institute for Health Sciences and Technology, Sungkyunkwan University School of Medicine, Seoul, South Korea; 546 Cheonan Industry-Academic Collaboration Foundation, Sangmyung University, Cheonan, South Korea; 547 NYU Langone Medical Center, New York, NY USA; 548 Department of Hematology and Medical Oncology, Cleveland Clinic, Cleveland, OH USA; 549 Department of Radiation Oncology, University of California San Francisco, San Francisco, CA USA; 550 Department of Health Sciences Research, Mayo Clinic, Rochester, MN USA; 551 Helen F. Graham Cancer Center at Christiana Care Health Systems, Newark, DE USA; 552 Heidelberg University Hospital, Heidelberg, Germany; 553 CSRA Incorporated, Fairfax, VA USA; 554 Research Department of Pathology, University College London Cancer Institute, London, UK; 555 Department of Research Oncology, Guy’s Hospital, King’s Health Partners AHSC, King’s College London School of Medicine, London, UK; 556 Faculty of Medicine and Health Sciences, Macquarie University, Sydney, NSW Australia; 557 University Hospital of Minjoz, INSERM UMR 1098, Besançon, France; 558 Spanish National Cancer Research Centre, Madrid, Spain; 559 Center of Digestive Diseases and Liver Transplantation, Fundeni Clinical Institute, Bucharest, Romania; 560 Cureline, Inc, South San Francisco, CA USA; 561 St. Luke’s Cancer Centre, Royal Surrey County Hospital NHS Foundation Trust, Guildford, UK; 562 Cambridge Breast Unit, Addenbrooke’s Hospital, Cambridge University Hospital NHS Foundation Trust and NIHR Cambridge Biomedical Research Centre, Cambridge, UK; 563 East of Scotland Breast Service, Ninewells Hospital, Aberdeen, UK; 564 Department of Genetics, Microbiology and Statistics, University of Barcelona, IRSJD, IBUB, Barcelona, Spain; 565 Department of Obstetrics and Gynecology, Medical College of Wisconsin, Milwaukee, WI USA; 566 Hematology and Medical Oncology, Winship Cancer Institute of Emory University, Atlanta, GA USA; 567 Department of Computer Science, Princeton University, Princeton, NJ USA; 568 Vanderbilt Ingram Cancer Center, Vanderbilt University, Nashville, TN USA; 569 Ohio State University College of Medicine and Arthur G. James Comprehensive Cancer Center, Columbus, OH USA; 570 Department of Surgery, Yokohama City University Graduate School of Medicine, Kanagawa, Japan; 571 Division of Chromatin Networks, German Cancer Research Center (DKFZ) and BioQuant, Heidelberg, Germany; 572 Research Computing Center, University of North Carolina at Chapel Hill, Chapel Hill, NC USA; 573 School of Molecular Biosciences and Center for Reproductive Biology, Washington State University, Pullman, WA USA; 574 Finsen Laboratory and Biotech Research and Innovation Centre (BRIC), University of Copenhagen, Copenhagen, Denmark; 575 Department of Laboratory Medicine and Pathobiology, University of Toronto, Toronto, ON Canada; 576 Department of Pathology, Human Oncology and Pathogenesis Program, Memorial Sloan Kettering Cancer Center, New York, NY USA; 577 University Hospital Giessen, Pediatric Hematology and Oncology, Giessen, Germany; 578 Oncologie Sénologie, ICM Institut Régional du Cancer, Montpellier, France; 579 Institute of Clinical Molecular Biology, Christian-Albrechts-University, Kiel, Germany; 580 Institute of Pathology, University of Wuerzburg, Wuerzburg, Germany; 581 Department of Urology, North Bristol NHS Trust, Bristol, UK; 582 SingHealth, Duke-NUS Institute of Precision Medicine, National Heart Centre Singapore, Singapore, Singapore; 583 Department of Computer Science, University of Toronto, Toronto, ON Canada; 584 Bern Center for Precision Medicine, University Hospital of Bern, University of Bern, Bern, Switzerland; 585 Englander Institute for Precision Medicine, Weill Cornell Medicine and New York Presbyterian Hospital, New York, NY USA; 586 Meyer Cancer Center, Weill Cornell Medicine, New York, NY USA; 587 Pathology and Laboratory, Weill Cornell Medical College, New York, NY USA; 588 Vall d’Hebron Institute of Oncology: VHIO, Barcelona, Spain; 589 General and Hepatobiliary-Biliary Surgery, Pancreas Institute, University and Hospital Trust of Verona, Verona, Italy; 590 National Centre for Biological Sciences, Tata Institute of Fundamental Research, Bangalore, India; 591 Indiana University, Bloomington, IN USA; 592 Department of Pathology, GZA-ZNA Hospitals, Antwerp, Belgium; 593 Analytical Biological Services, Inc, Wilmington, DE USA; 594 Sydney Medical School, University of Sydney, Sydney, NSW Australia; 595 cBio Center, Dana-Farber Cancer Institute, Harvard Medical School, Boston, MA USA; 596 Department of Cell Biology, Harvard Medical School, Boston, MA USA; 597 Advanced Centre for Treatment Research and Education in Cancer, Tata Memorial Centre, Navi Mumbai, Maharashtra India; 598 School of Environmental and Life Sciences, Faculty of Science, The University of Newcastle, Ourimbah, NSW Australia; 599 Department of Dermatology, University Hospital of Essen, Essen, Germany; 600 Bioinformatics and Omics Data Analytics, German Cancer Research Center (DKFZ), Heidelberg, Germany; 601 Department of Urology, Charité Universitätsmedizin Berlin, Berlin, Germany; 602 Martini-Clinic, Prostate Cancer Center, University Medical Center Hamburg-Eppendorf, Hamburg, Germany; 603 Department of General Internal Medicine, University of Kiel, Kiel, Germany; 604 German Cancer Consortium (DKTK), Partner site Berlin, Berlin, Germany; 605 Cancer Research Institute, Beth Israel Deaconess Medical Center, Boston, MA USA; 606 University of Pittsburgh, Pittsburgh, PA USA; 607 Department of Ophthalmology and Ocular Genomics Institute, Massachusetts Eye and Ear, Harvard Medical School, Boston, MA USA; 608 Center for Psychiatric Genetics, NorthShore University HealthSystem, Evanston, IL USA; 609 Van Andel Research Institute, Grand Rapids, MI USA; 610 Laboratory of Molecular Medicine, Human Genome Center, Institute of Medical Science, University of Tokyo, Tokyo, Japan; 611 Japan Agency for Medical Research and Development, Tokyo, Japan; 612 Korea University, Seoul, South Korea; 613 Murtha Cancer Center, Walter Reed National Military Medical Center, Bethesda, MD USA; 614 Human Genetics, University of Kiel, Kiel, Germany; 615 Department of Oncologic Pathology, Dana-Farber Cancer Institute, Harvard Medical School, Boston, MA USA; 616 Oregon Health and Science University, Portland, OR USA; 617 Center for RNA Interference and Noncoding RNA, The University of Texas MD Anderson Cancer Center, Houston, TX USA; 618 Department of Experimental Therapeutics, The University of Texas MD Anderson Cancer Center, Houston, TX USA; 619 Department of Gynecologic Oncology and Reproductive Medicine, The University of Texas MD Anderson Cancer Center, Houston, TX USA; 620 University Hospitals Coventry and Warwickshire NHS Trust, Coventry, UK; 621 Department of Radiation Oncology, Radboud University Nijmegen Medical Centre, Nijmegen, GA The Netherlands; 622 Institute for Genomics and Systems Biology, University of Chicago, Chicago, IL USA; 623 Clinic for Hematology and Oncology, St.-Antonius-Hospital, Eschweiler, Germany; 624 Computational and Systems Biology Program, Memorial Sloan Kettering Cancer Center, New York, NY USA; 625 University of Iceland, Reykjavik, Iceland; 626 Division of Computational Genomics and Systems Genetics, German Cancer Research Center (DKFZ), Heidelberg, Germany; 627 Dundee Cancer Centre, Ninewells Hospital, Dundee, UK; 628 Department for Internal Medicine III, University of Ulm and University Hospital of Ulm, Ulm, Germany; 629 Institut Curie, INSERM Unit 830, Paris, France; 630 Department of Gastroenterology and Hepatology, Yokohama City University Graduate School of Medicine, Kanagawa, Japan; 631 Department of Laboratory Medicine, Radboud University Nijmegen Medical Centre, Nijmegen, GA The Netherlands; 632 Division of Cancer Genome Research, German Cancer Research Center (DKFZ), Heidelberg, Germany; 633 Department of General Surgery, Singapore General Hospital, Singapore, Singapore; 634 Cancer Science Institute of Singapore, National University of Singapore, Singapore, Singapore; 635 Department of Medical and Clinical Genetics, Genome-Scale Biology Research Program, University of Helsinki, Helsinki, Finland; 636 East Anglian Medical Genetics Service, Cambridge University Hospitals NHS Foundation Trust, Cambridge, UK; 637 Irving Institute for Cancer Dynamics, Columbia University, New York, NY USA; 638 Institute of Molecular and Cell Biology, Singapore, Singapore; 639 Laboratory of Cancer Epigenome, Division of Medical Science, National Cancer Centre Singapore, Singapore, Singapore; 640 Universite Lyon, INCa-Synergie, Centre Léon Bérard, Lyon, France; 641 Department of Urology, Mayo Clinic, Rochester, MN USA; 642 Royal National Orthopaedic Hospital - Stanmore, Stanmore, Middlesex UK; 643 Department of Biochemistry, Genetics and Immunology, University of Vigo, Vigo, Spain; 644 Giovanni Paolo II / I.R.C.C.S. Cancer Institute, Bari, BA Italy; 645 Neuroblastoma Genomics, German Cancer Research Center (DKFZ), Heidelberg, Germany; 646 Fondazione Policlinico Universitario Gemelli IRCCS, Rome, Italy, Rome, Italy; 647 University of Verona, Verona, Italy; 648 Centre National de Génotypage, CEA - Institute de Génomique, Evry, France; 649 CAPHRI Research School, Maastricht University, Maastricht, ER The Netherlands; 650 Department of Biopathology, Centre Léon Bérard, Lyon, France; 651 Université Claude Bernard Lyon 1, Villeurbanne, France; 652 Core Research for Evolutional Science and Technology (CREST), JST, Tokyo, Japan; 653 Department of Biological Sciences, Laboratory for Medical Science Mathematics, Graduate School of Science, University of Tokyo, Yokohama, Japan; 654 Department of Medical Science Mathematics, Medical Research Institute, Tokyo Medical and Dental University (TMDU), Tokyo, Japan; 655 Cancer Ageing and Somatic Mutation Programme, Wellcome Sanger Institute, Hinxton, UK; 656 University Hospitals Birmingham NHS Foundation Trust, Birmingham, UK; 657 Centre for Cancer Research and Cell Biology, Queen’s University, Belfast, UK; 658 Breast Medical Oncology, The University of Texas MD Anderson Cancer Center, Houston, TX USA; 659 Department of Surgery, Johns Hopkins University School of Medicine, Baltimore, MD USA; 660 Department of Oncology-Pathology, Science for Life Laboratory, Karolinska Institute, Stockholm, Sweden; 661 School of Cancer Sciences, Faculty of Medicine, University of Southampton, Southampton, UK; 662 Department of Gene Technology, Tallinn University of Technology, Tallinn, Estonia; 663 Genetics and Genome Biology Program, SickKids Research Institute, The Hospital for Sick Children, Toronto, ON Canada; 664 Departments of Neurosurgery and Hematology and Medical Oncology, Winship Cancer Institute and School of Medicine, Emory University, Atlanta, GA USA; 665 Department of Clinical and Molecular Medicine, Faculty of Medicine and Health Sciences, Norwegian University of Science and Technology, Trondheim, Norway; 666 Argmix Consulting, North Vancouver, BC Canada; 667 Department of Information Technology, Ghent University, Interuniversitair Micro-Electronica Centrum (IMEC), Ghent, Belgium; 668 Nuffield Department of Surgical Sciences, John Radcliffe Hospital, University of Oxford, Oxford, UK; 669 Institute of Mathematics and Computer Science, University of Latvia, Riga, LV Latvia; 670 Discipline of Pathology, Sydney Medical School, University of Sydney, Sydney, NSW Australia; 671 Department of Applied Mathematics and Theoretical Physics, Centre for Mathematical Sciences, University of Cambridge, Cambridge, UK; 672 Department of Epidemiology and Biostatistics, Memorial Sloan Kettering Cancer Center, New York, NY USA; 673 Department of Statistics, Columbia University, New York, NY USA; 674 Department of Immunology, Genetics and Pathology, Science for Life Laboratory, Uppsala University, Uppsala, Sweden; 675 School of Electronic and Information Engineering, Xi’an Jiaotong University, Xi’an, China; 676 Department of Histopathology, Cambridge University Hospitals NHS Foundation Trust, Cambridge, UK; 677 Oxford NIHR Biomedical Research Centre, University of Oxford, Oxford, UK; 678 Georgia Regents University Cancer Center, Augusta, GA USA; 679 Wythenshawe Hospital, Manchester, UK; 680 Department of Genetics, Washington University School of Medicine, St.Louis, MO USA; 681 Department of Biological Oceanography, Leibniz Institute of Baltic Sea Research, Rostock, Germany; 682 Wellcome Centre for Human Genetics, University of Oxford, Oxford, UK; 683 Department of Molecular and Human Genetics, Baylor College of Medicine, Houston, TX USA; 684 Thoracic Oncology Laboratory, Mayo Clinic, Rochester, MN USA; 685 Institute for Genomic Medicine, Nationwide Children’s Hospital, Columbus, OH USA; 686 Department of Obstetrics and Gynecology, Division of Gynecologic Oncology, Mayo Clinic, Rochester, MN USA; 687 International Institute for Molecular Oncology, Poznań, Poland; 688 Poznan University of Medical Sciences, Poznań, Poland; 689 Genomics and Proteomics Core Facility High Throughput Sequencing Unit, German Cancer Research Center (DKFZ), Heidelberg, Germany; 690 NCCS-VARI Translational Research Laboratory, National Cancer Centre Singapore, Singapore, Singapore; 691 Edison Family Center for Genome Sciences and Systems Biology, Washington University, St. Louis, MO USA; 692 MRC-University of Glasgow Centre for Virus Research, Glasgow, UK; 693 Department of Medical Informatics and Clinical Epidemiology, Division of Bioinformatics and Computational Biology, OHSU Knight Cancer Institute, Oregon Health and Science University, Portland, OR USA; 694 School of Electronic Information and Communications, Huazhong University of Science and Technology, Wuhan, China; 695 Department of Applied Mathematics and Statistics, Johns Hopkins University, Baltimore, MD USA; 696 Department of Cancer Genome Informatics, Graduate School of Medicine, Osaka University, Osaka, Japan; 697 Institute of Computer Science, Heidelberg University, Heidelberg, Germany; 698 School of Mathematics and Statistics, University of Sydney, Sydney, NSW Australia; 699 Ben May Department for Cancer Research, University of Chicago, Chicago, IL USA; 700 Department of Human Genetics, University of Chicago, Chicago, IL USA; 701 Tri-Institutional PhD Program in Computational Biology and Medicine, Weill Cornell Medicine, New York, NY USA; 702 The First Affiliated Hospital, Xi’an Jiaotong University, Xi’an, China; 703 Department of Medicine and Therapeutics, The Chinese University of Hong Kong, Shatin, NT, Hong Kong China; 704 Department of Biostatistics, The University of Texas MD Anderson Cancer Center, Houston, TX USA; 705 Duke-NUS Medical School, Singapore, Singapore; 706 Department of Surgery, Ruijin Hospital, Shanghai Jiaotong University School of Medicine, Shanghai, China; 707 School of Computing Science, University of Glasgow, Glasgow, UK; 708 Division of Orthopaedic Surgery, Oslo University Hospital, Oslo, Norway; 709 Eastern Clinical School, Monash University, Melbourne, VIC Australia; 710 Epworth HealthCare, Richmond, VIC Australia; 711 Department of Biostatistics and Computational Biology, Dana-Farber Cancer Institute and Harvard Medical School, Boston, MA USA; 712 Department of Biomedical Informatics, College of Medicine, The Ohio State University, Columbus, OH USA; 713 The Ohio State University Comprehensive Cancer Center (OSUCCC – James), Columbus, OH USA; 714 The University of Texas School of Biomedical Informatics (SBMI) at Houston, Houston, TX USA; 715 Department of Biostatistics, University of North Carolina at Chapel Hill, Chapel Hill, NC USA; 716 Department of Biochemistry and Molecular Genetics, Feinberg School of Medicine, Northwestern University, Chicago, IL USA; 717 Faculty of Medicine and Health, University of Sydney, Sydney, NSW Australia; 718 Department of Pathology, Erasmus Medical Center Rotterdam, Rotterdam, GD The Netherlands; 719 Division of Molecular Carcinogenesis, The Netherlands Cancer Institute, Amsterdam, CX The Netherlands; 720 Institute of Molecular Life Sciences and Swiss Institute of Bioinformatics, University of Zurich, Zurich, Switzerland

**Keywords:** Cancer genomics, Cancer genomics

Correction to: *Nature* 10.1038/s41586-020-1969-6 Published online 5 February 2020

In the published version of this paper, the list of members of the Pan-Cancer Analysis of Whole Genomes (PCAWG) Consortium and their affiliations contained minor errors in the affiliations. The original Article has been corrected to include the corrected list.

