# Supplementary material for: Pan-cancer analysis of whole genomes
Source: Nature. 2020 Feb 5;578(7793):82–93. doi: 10.1038/s41586-020-1969-6 (PMC7025898; doi:10.1038/s41586-020-1969-6)
Supplement: Supplementary file 1 — This file contains Supplementary Figures 1-19, Supplementary Methods and Supplementary Notes 1-6 [file 41586_2020_1969_MOESM1_ESM.pdf]

nature

https://doi.org/10.1038/s41586-020-1969-6

## Supplementary information

# Pan-cancer analysis of whole genomes

# In the format provided by the authors and unedited

# Pan-cancer analysis of whole genomes

The ICGC/TCGA Pan-Cancer Analysis of Whole Genomes Consortium §

§ Authors and affiliations listed at end of main paper

## Table of contents

**Supplementary Figures 1-19** 5

**Supplementary Methods** 24

1. Pilot-63 Analysis 24
   - 1.1 Validation Process 24
   - 1.2 Processing of Validation Data 25
   - 1.3 Variant Classification using VAFs 25
   - 1.4 Calculating Per-Caller Accuracy 25
   - 1.5 SNV/Indel Callers Used in Pilot-63 Exercise 26
     - 1.5.1 ADISCAN_Beta 26
     - 1.5.2 LOHcomplete 27
     - 1.5.3 OICR_bl 27
     - 1.5.4 OICR_SGA 27
     - 1.5.5 WUSTL 28
     - 1.5.6 CRG-clindel 28
     - 1.5.7 Novobreak-indel 29
2. Whole Genome Sequencing Somatic Variant Calling 29
   - 2.1 Whole Genome Alignment 29
   - 2.2 Variant Calling Pipelines Used During Production 30
     - 2.2.1 DKFZ Pipeline 30
     - 2.2.2 EMBL Pipeline 30
     - 2.2.3 Sanger Pipeline 31
     - 2.2.4 Broad Pipeline 32
     - 2.2.5 MuSE Pipeline 33
     - 2.2.6 SMuFIN Pipeline 33
   - 2.3 Consensus Somatic SNV/Indel Annotation 33
   - 2.4 Merging of WGS somatic variant calls 34
     - 2.4.1 Somatic SNV and indel Merging 34

2.4.2 Somatic SV Merging 34
2.4.3 Somatic Copy Number Alteration Merging 34
2.5 Variant Call Set Quality Control and Flagging 35
2.5.1 Tumour in Normal Estimation 35
2.5.2 Germline site somatic mutation filter 35
2.5.3 Oxidative Artefact Filtration 36
2.5.4 Strand Bias Filtration 36
2.5.5 Review and curation of consensus variant calls 36
2.5.5.1 Sample Exclusion Criteria 37
2.5.5.2 Variant Exclusion Criteria 37
2.6 miniBAM generation 38
3. Germline Variant Identification from WGS 38
3.1 Data Overview and Call-set Generation 38
3.1.1 Broad germline SNP and indel call-set generation 39
3.1.2 Freebayes germline SNP and InDel call-set 40
3.1.3 Short variant calling with the Real Time Genomics (RTG) software 40
3.1.4 Delly germline deletions 40
3.1.5 Mobile element insertion call set 41
3.1.6 Orthogonal validation of germline MEI with single-molecule sequencing 42
3.1.7 Germline L1 source element analysis 43
3.1.8 Short germline variant consensus call-set 44
3.2 Germline short variant validation 44
3.2.1 Short variant call validation experiments 44
3.3 Whole-genome low coverage structural variant validation using long-reads 46
3.4 Haplotype-block phasing of germline variants using 1000GP as a haplotype reference panel 47
3.5 Inference of continental-scale ancestry and genome-wide local ancestry deconvolution 48
3.6 Identification of protein-truncating variants (PTVs) 48
3.7 Rare variant germline-somatic variant association analysis 49
3.8 Common variant germline-somatic variant association analyses 49
3.9 Knowledge-based analysis of mutational processes 50
4. RNA-Seq Analysis 51
4.1 RNA-seq alignment and quality control 51
4.2 Quantification of gene and transcript-level expression 51
4.3 Identification of alternative splicing events 51

4.4 Fusion transcript identification 52
5. Clustering of Tumour Genomes Based on Telomere Maintenance-Related Features 52
6. Clustered mutational processes in PCAWG 53
6.1 Inference of chromothripsis 53
6.1.1 Chromothripsis drivers and patterns 54
6.1.2 Timing of amplified chromothripsis using SNVs 54
6.1.3 Quantifying the time spent during amplification after chromothripsis 55
6.2 Inference of chromoplexy 55
6.3 Inference of kataegis 56
6.3.1 Analysis of kataegis drivers and patterns 57
6.4 Subclonal architecture reconstruction 58
6.5 Clonality assessment of punctuated events 58
6.6. Data Availability 59
7. Tumours without detected driver mutations 59
8. Panorama of driver mutations in human cancer 60
8.1 The onCohortDrive method 60
8.2 The Compendium of Mutational Driver GEs 61
8.3 Features 62
8.3.1. Functional Impact (FI) 62
8.3.2. Clustering of mutations 63
8.3.3 Ranking mutations based on FI and clusters 64
8.3.4. Mutational unlikeliness 64
8.3.5. Element specific scores 65
8.3.6. Other evaluated features 65
8.4. The onCohortDrive workflow 65
8.4.1. Overview 65
8.4.2. Analysing mutations in driver GEs 65
8.4.3. Identification of known tumorigenic mutations 66
8.4.4. Rank-based approach 66
8.4.5. Rule-based approach 67
8.4.6. Post-processing 68
8.5. Benchmarking onCohortDrive 68
8.6. Processing exceptional GEs or mutations 69
8.6.1. Mutation in intronic splice sites 70
8.6.2. Indels and MNVs 70

8.6. Unbiased calculation of the contribution of noncoding driver mutations 70
8.7. Identification of driver SCNAs and SGRs in tumours of the PCAWG cohort 71
8.7.1. Creating the Compendium of driver SCNA elements 71
8.7.2. Identification of driver CNA events in PCAWG tumours 72
8.7.3. Additional driver SCNA events in PCAWG tumours 72
8.7.4. Identification of driver somatic genomic rearrangements in PCAWG tumours 72
8.8. Identification of likely tumorigenic germline variants 73
8.9. Identification of biallelic driver events 73
9. Literature Cited 74

## Supplementary Notes 84

Overview 84
1. Pilot-63 benchmarking and validation exercise 84
Stratified Mutation Sampling 85
Deep Sequencing 86
Accuracy on Validation Samples 87
2. Production calling and variant consensus development 87
Consensus SNV and Indel Models 88
3. Performance on Previously Validated Samples 89
Medulloblastoma 89
Known Cell lines 89
4. Production Somatic Variant Calling on the PCAWG Compute Cloud 90
Distributed Processing 91
Data Distribution to Downstream Analytic Groups 92
5. PCAWG data portals 93
The five data portals 93
Data sources for the PCAWG portals 97
6. Literature Cited 98

**Supplementary Figure 1. Distribution of coverage in (A) tumour and (B) normal samples in PCAWG.**

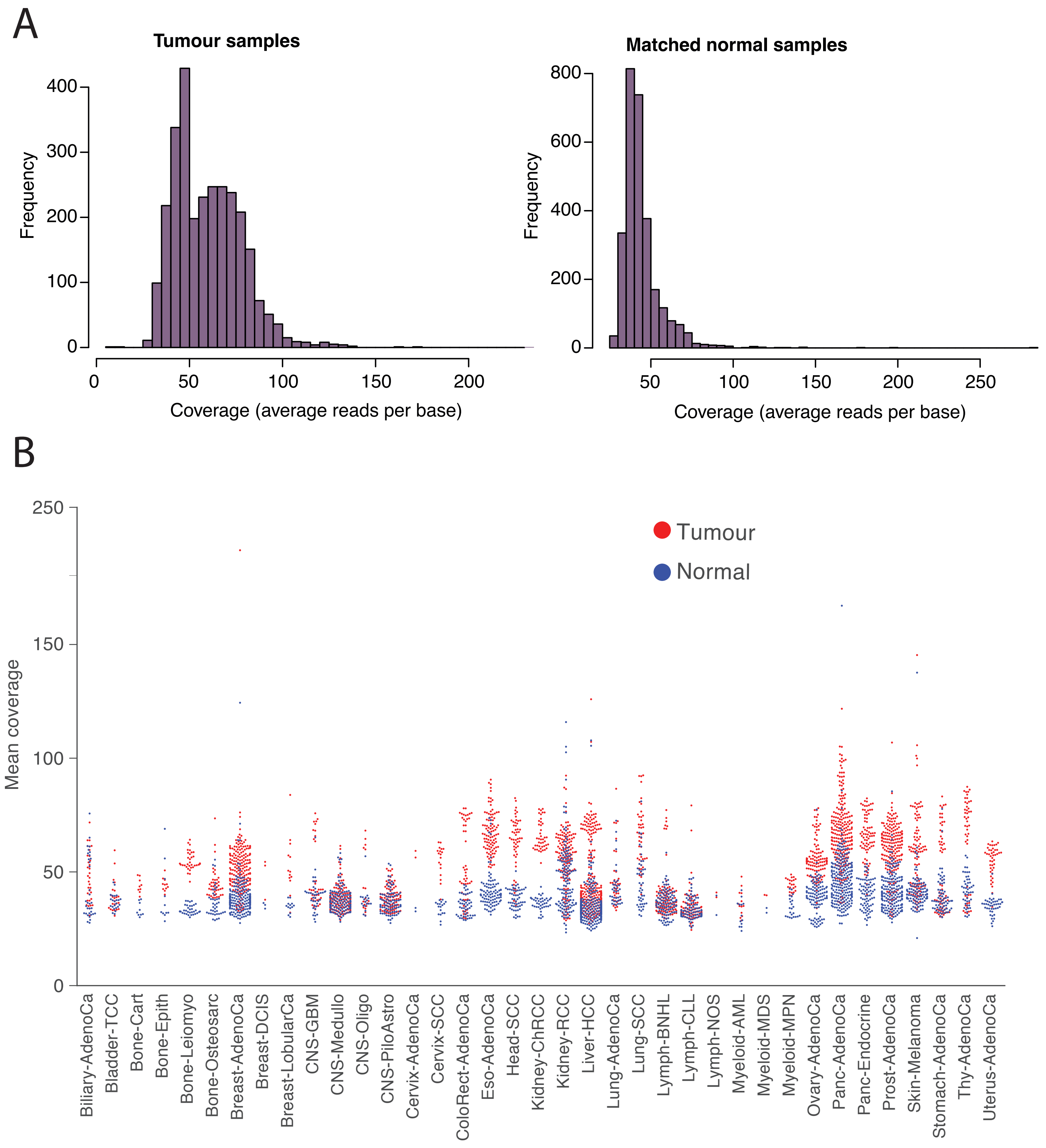

**Supplementary Figure 2. Flow-chart of variant calling in PCAWG.** Individual algorithms from the Sanger, DKFZ and Broad Institute pipelines, together with SMuFIN and MuSE, fed variants into a series of post-processing filters to remove false positives. These were then integrated using decision trees designed to maximise precision and sensitivity to generate a final set of called somatic mutations for each sample. SNV, single nucleotide variant; SV, structural variant; OxoG, filter for 8-oxoguanine-induced sequencing errors; Broad PoN Filter, filter for variants identified in a panel of normal samples constructed by the Broad Institute.

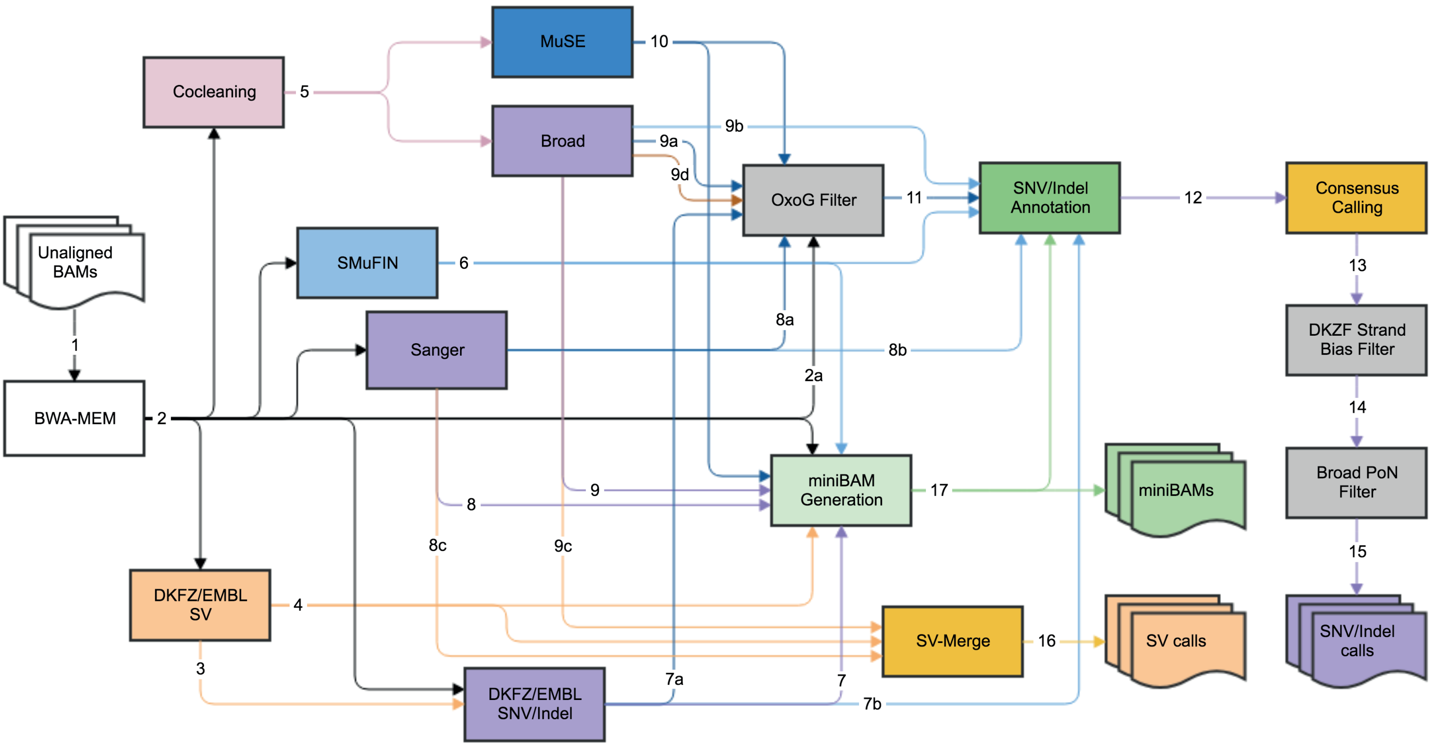


1: unaligned files
2: aligned BAMs (tumour/normal pair)
2a: aligned tumour BAM file
3: bedpe file
4: DKFZ/EMBL SV calls
5: Cocleaned BAMs
6: SMuFIN Indel calls
7: DKFZ/EMBL SNV/Indel calls
7a: DKFZ/EMBL SNV calls
7b: DKFZ/EMBL Indel calls
8: Sanger SNV/Indel/SV calls
8a: Sanger SNV calls
8b: Sanger Indel calls
8c: Sanger SV calls
9: Broad SNV/Indel/SV calls
9a: Broad SNV calls
9b: Broad Indel calls
9c: Broad SV calls
9d: OxoQ score
10: MuSE SNV calls
11: OxoG filtered SNV calls
12: Annotated SNV/Indel calls
13: Consensus SNV/Indel calls
14: Strand bias filtered calls
15: Panel of normals filtered calls (final SNV/Indel calls)
16: Consensus SV calls (final SV calls)
17: miniBAM files

**Supplementary Figure 3. Pairwise comparison of rates of different classes of somatic mutation.** Points are coloured by tumour type, as depicted in the legend. Both x and y axes are on a log scale. SNVs, single nucleotide variants (substitutions); Indels, insertions or deletions <100 base pairs in size; SVs, structural variants; Retrotranspositions, counts of somatic retrotransposon insertions, transductions and somatic pseudogene insertions.

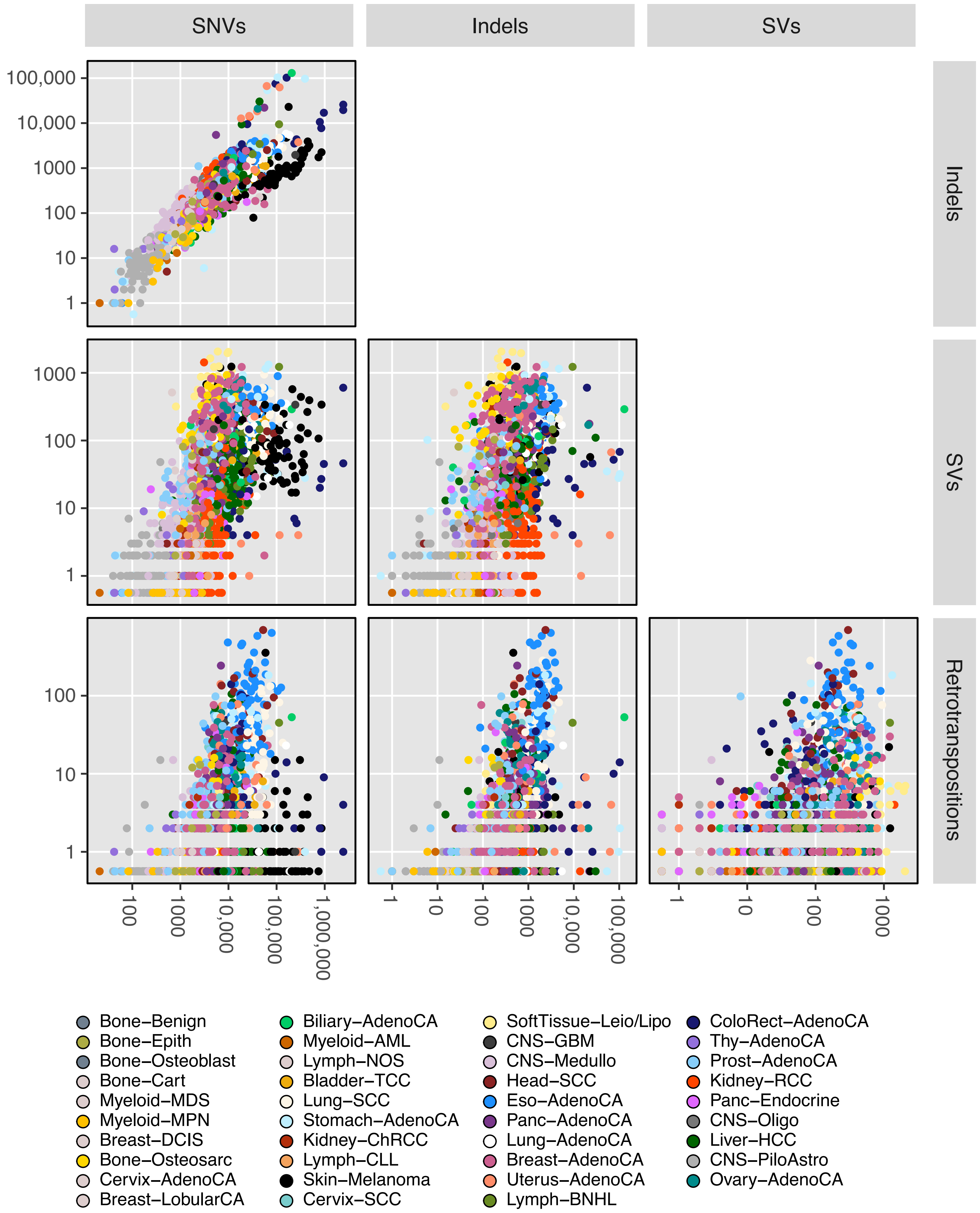

**Supplementary Figure 4. Correlation of somatic mutation load with age.** Each point corresponds to a patient, and points are coloured by tumour type, as depicted in the legend for Extended Figure 3. Clockwise from top left, panels correspond to burden of SNVs, Indels, SVs, Retrotranspositions, MNVs, and mtDNA mutations.

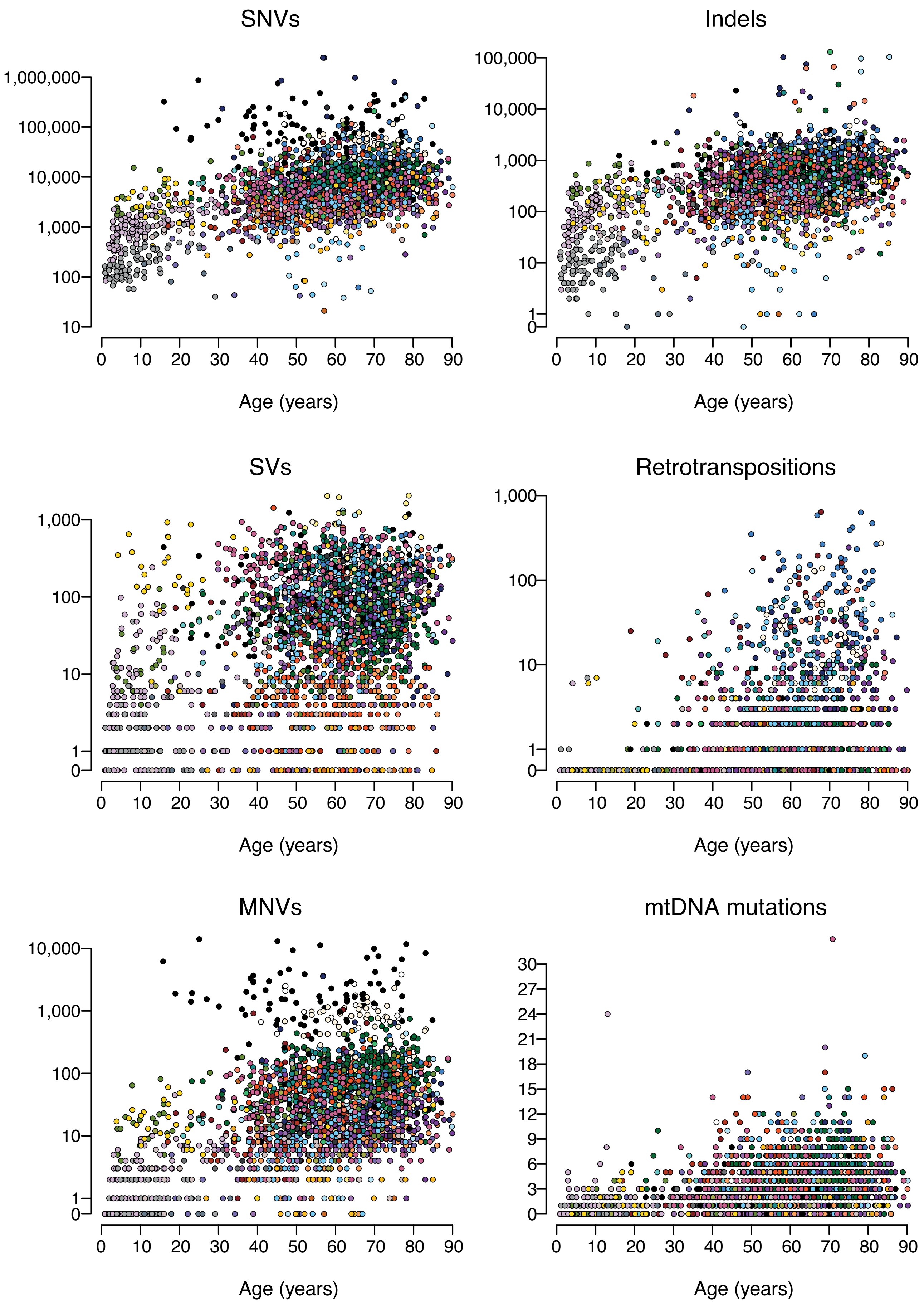

**Supplementary Figure 5. Volcano plot of mixed effects models of APOBEC kataegis.** Fitted coefficients for PCAWG drivers and cytidine deaminase expression levels (regular and blue circled dots, respectively) are plotted against the modulus of their Z-values, estimated using two-sided mixed effects models, based on n=1,222 patients with RNA-sequencing data. Coefficients with a Benjamini-Hochberg corrected q ≤ 0.05 are highlighted in red. Forward selection with these variables led to a final model containing APOBEC3B expression level, the number of rearrangements and patient age at diagnosis. Addition of BRCA2 or ATM status did not further improve the model.

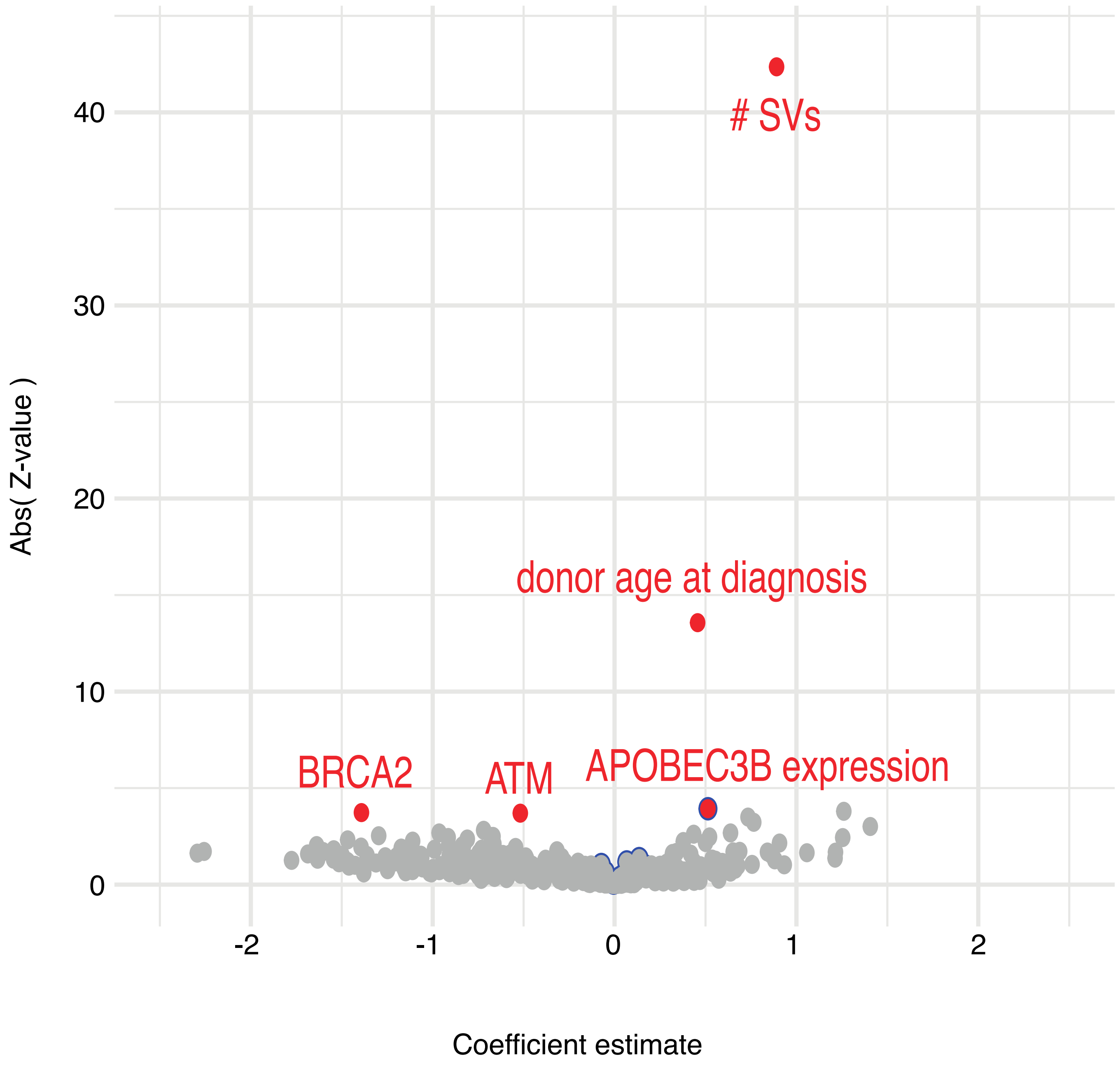

**Supplementary Figure 6. Association of kataegis with structural variants.** (A) Density estimates for the distance distribution from kataegis foci to the nearest breakpoint, stratified by signature. The dashed line indicates the 1kb cut-off used for SV-association. (B) Stacked bar plot showing the proportion of kataegis clusters associated with different classes of structural variant, split by kataegis signature. The overall distribution of classes of structural variant across PCAWG as a whole is shown at the top. (C) Density estimates for the size distribution (on a log scale) of deletions associated with different kataegis signatures.

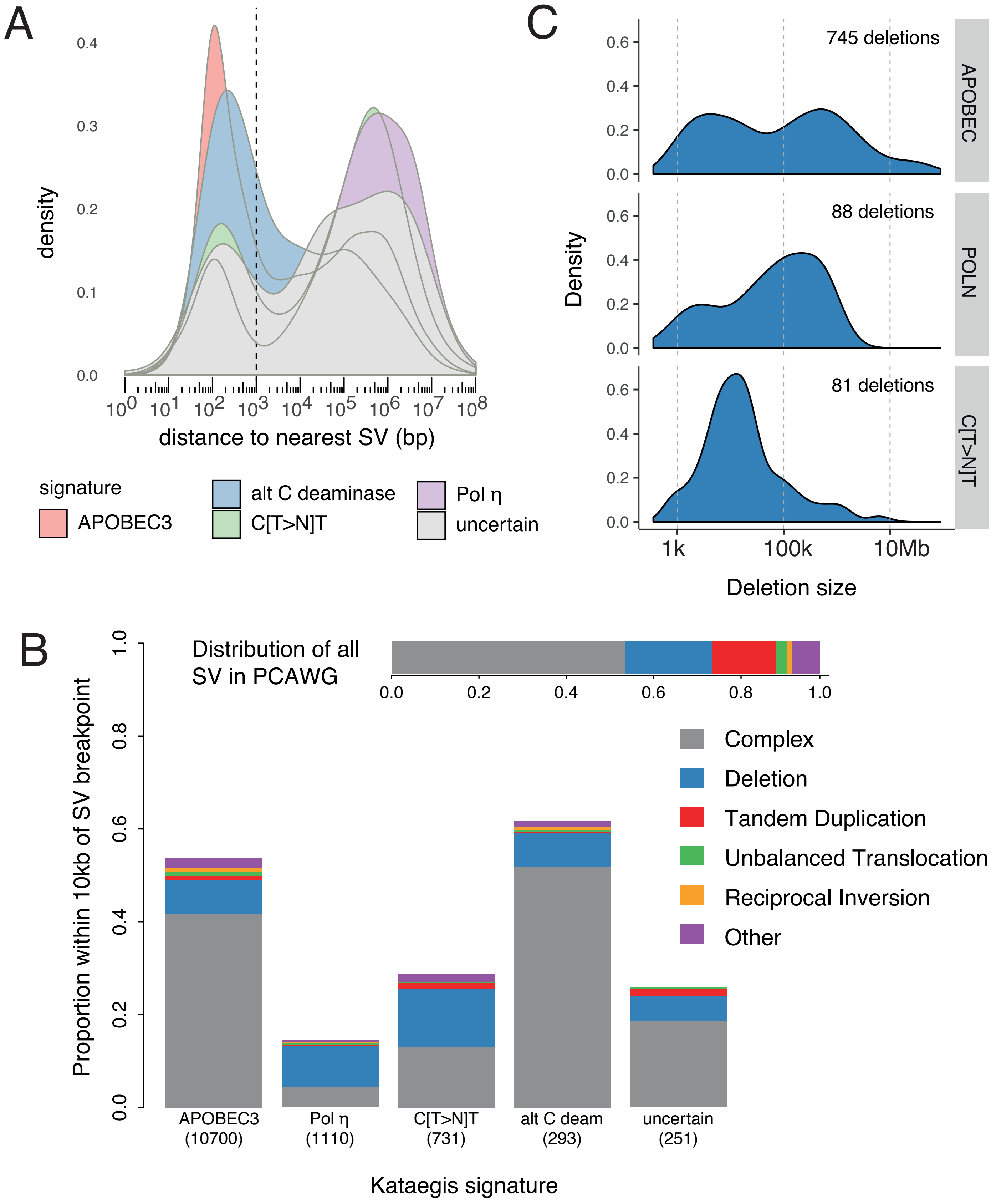

**Supplementary Figure 7. Chromothripsis events involving the TERT gene in chromophobe renal cell cancers.** The black points in the upper panel represent copy number estimates from individual genomic bins, with structural variants shown as coloured arcs (translocation in black, deletion in purple, duplication in brown, tail-to-tail inversion in cyan, head-to-head inversion in green), mostly demarcating copy number changes. The mate chromosomes are displayed above translocations. The lower panel shows the variant allele fraction (VAF) of somatic mutations distributed along the relevant chromosomal region.

## Chromophobe kidney cancers: Chromosome 5

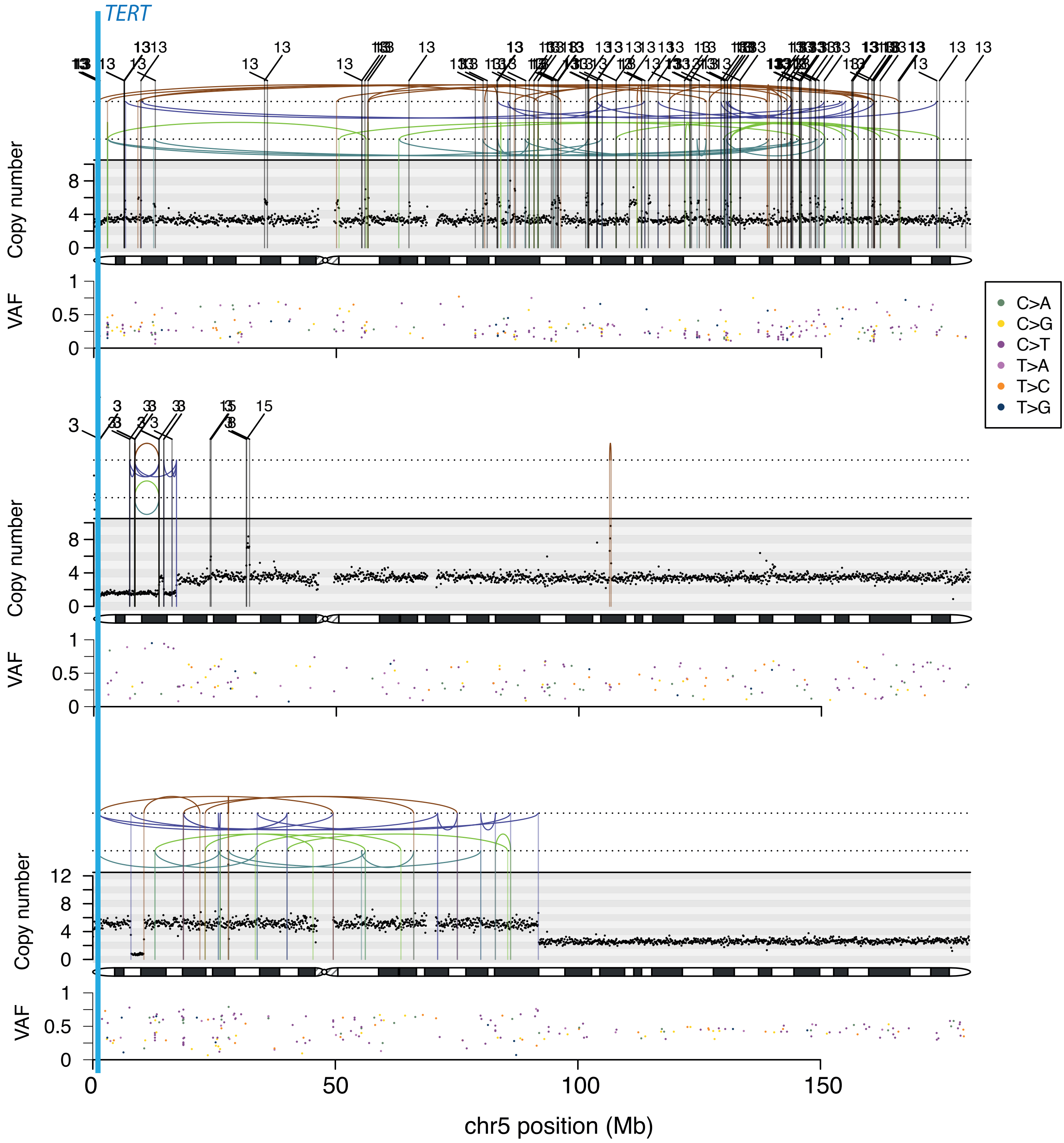

**Supplementary Figure 8. SV-related APOBEC during post-chromothripsis amplification across cancer types.** Copy number plot of chromothriptic regions categorised as “liposarc-like” in 10 samples from different cancer types. Segments indicate the copy number of the major allele. Points represent SNV multiplicities, i.e. the estimated number of copies carrying them, coloured by base change and shaped by strand. Small vertical arrows link SNVs to their corresponding copy number segment. Kataegis foci are shown within black boxes, and show typical strand-specificity (all triangles or all circles). Additional SV-related APOBEC SNVs are marked with a red box and were identified as C>T or C>G at intermediate multiplicities.

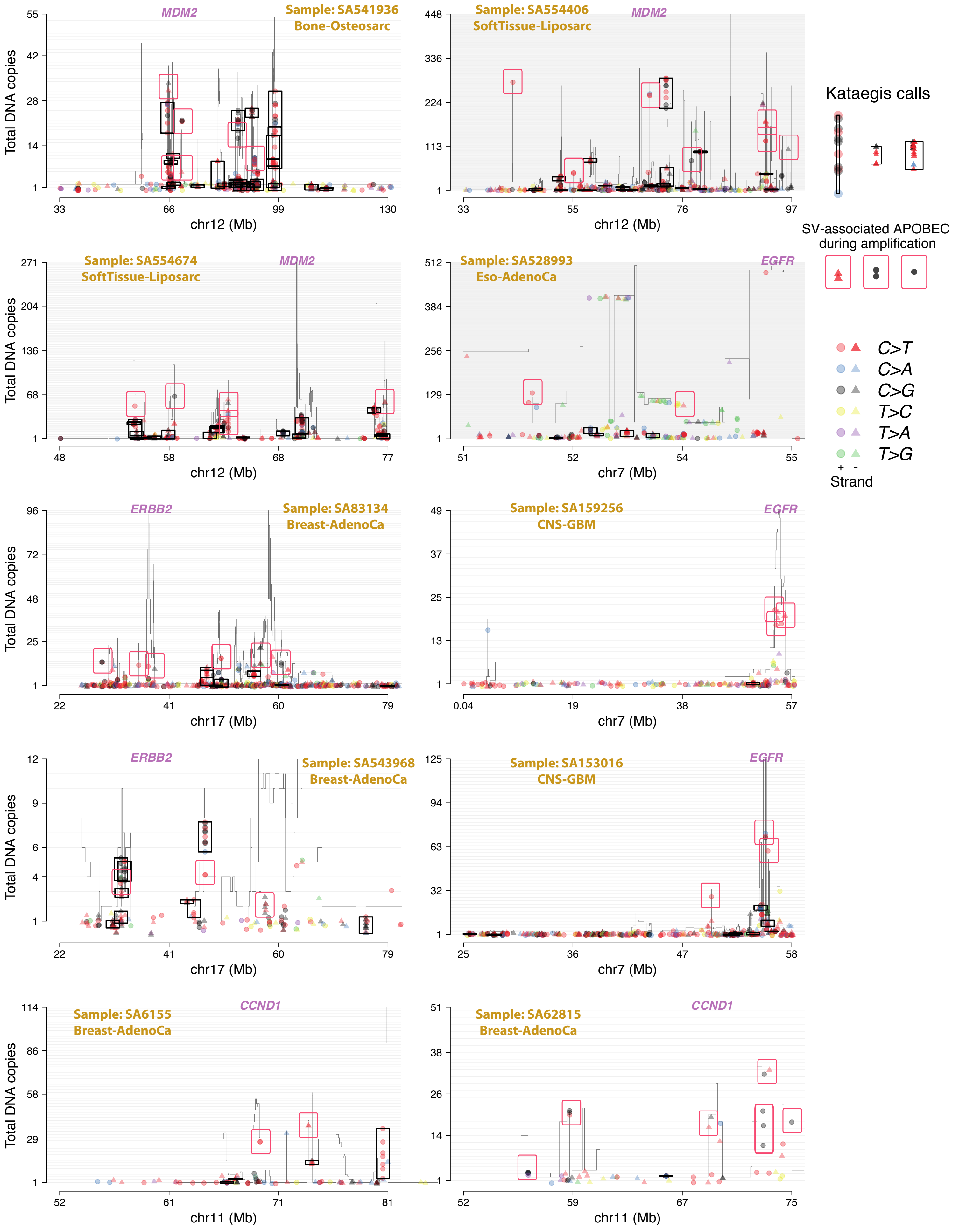

**Supplementary Figure 9. Properties of telomeres across different tumour clusters.** (A) Distribution of telomere sequence and properties across samples in the four clusters, with both tumour (blue points) and matched normal (red points) shown. Data are based on n=2518 tumour samples and their matched normal samples. (B) Enrichment (positive T statistics) or depletion (negative T statistics) of different variant sequence motifs in the four clusters of telomere properties. Data are based on n=2518 tumour samples. (C) Variance of frequency of different sequence motifs across the four clusters. Data are based on n=2518 tumour samples.

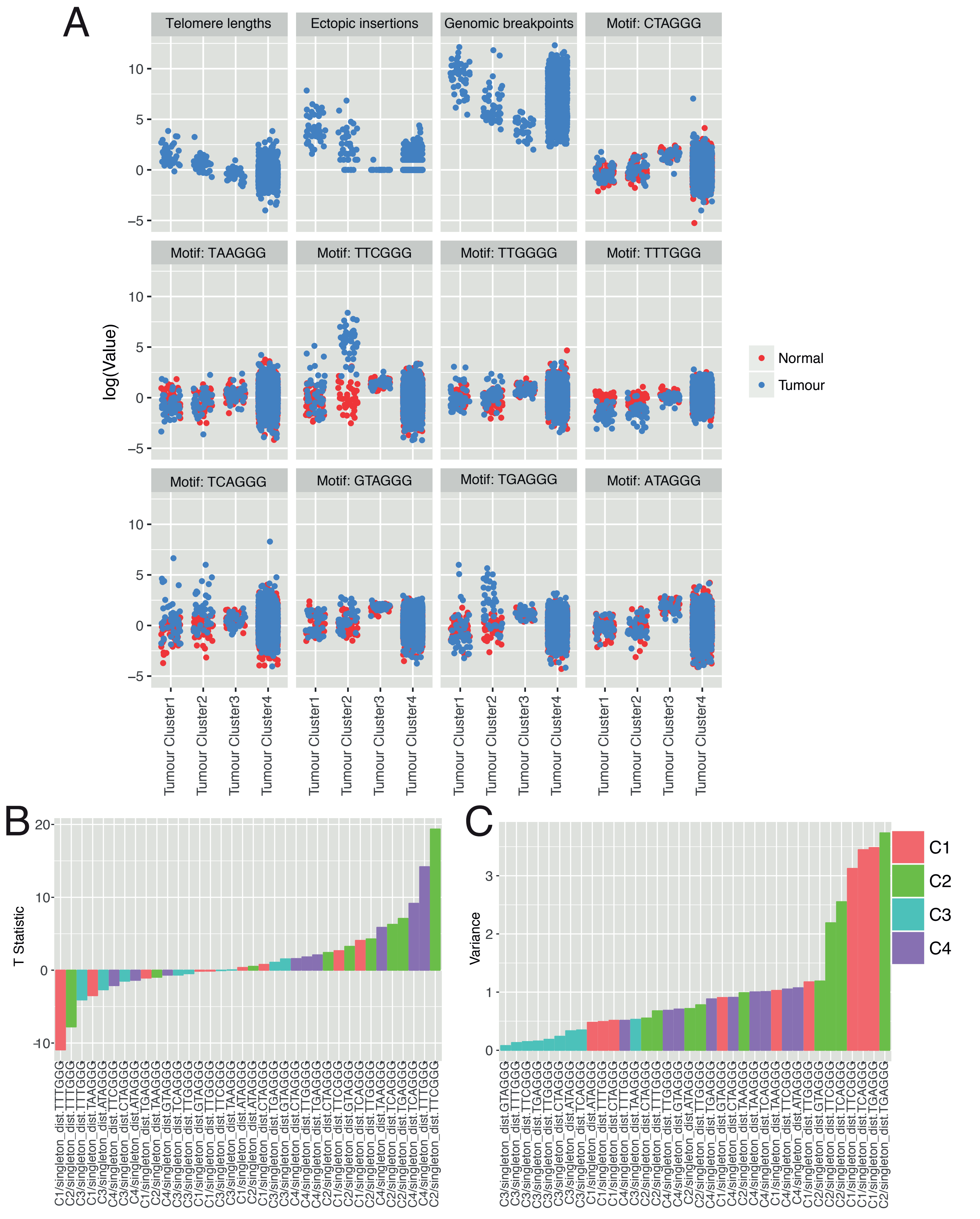

**Supplementary Figure 10. Properties of telomeres across different normal clusters.** Enrichment (positive T statistics) or depletion (negative T statistics) of different variant sequence motifs in the four normal clusters of telomere properties. Data are based on n=2518 normal samples.

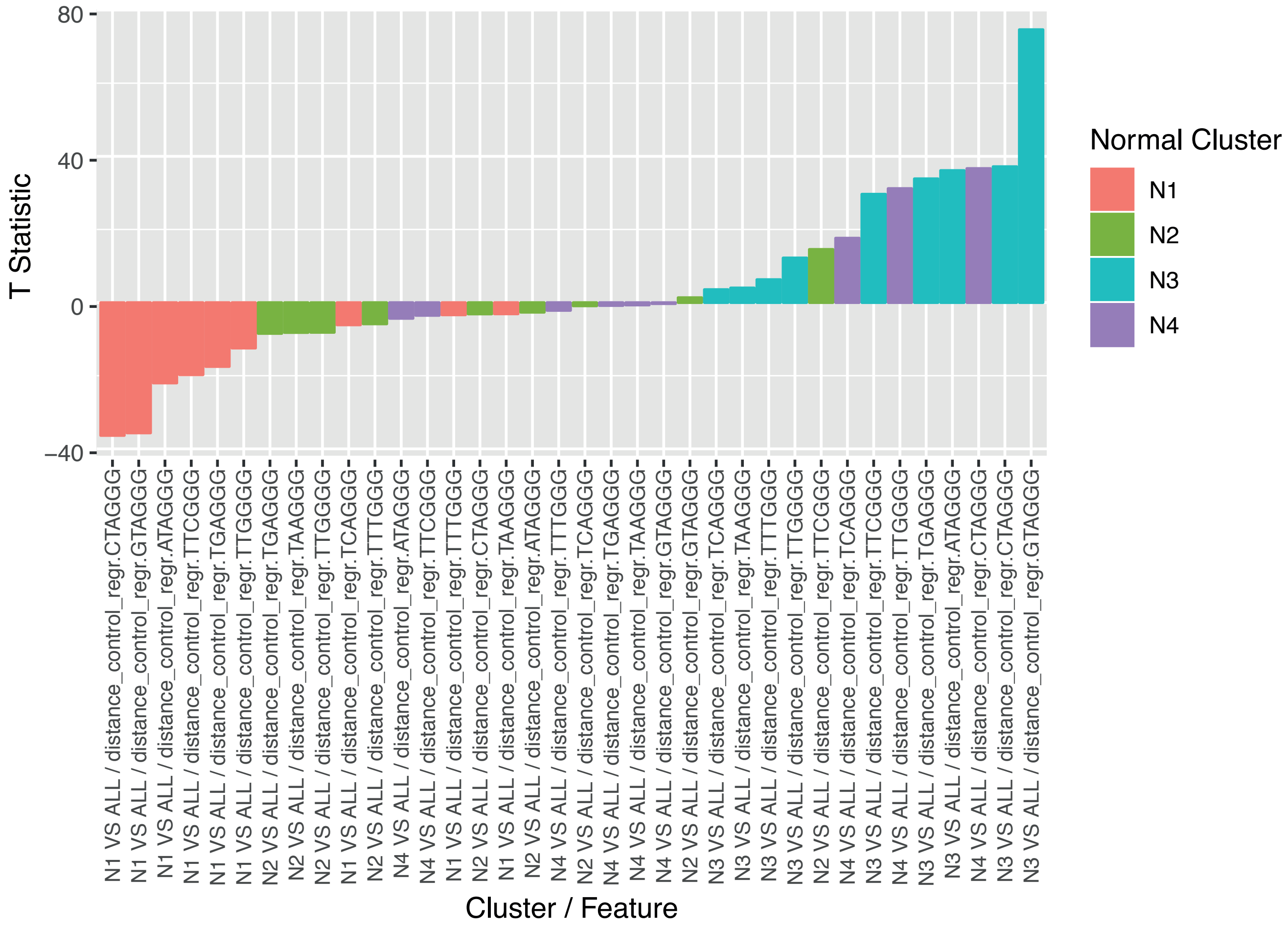

**Supplementary Figure 11. Distribution of Illumina Hi-Seq short read lengths across PCAWG tumour sample cohorts.** The distribution of DNA-seq read lengths across PCAWG tumour type cohorts are displayed as stacked bar plots. Reads from the 63 donors selected for the pilot projects are broken out as a separate category labeled "Pilot-63".

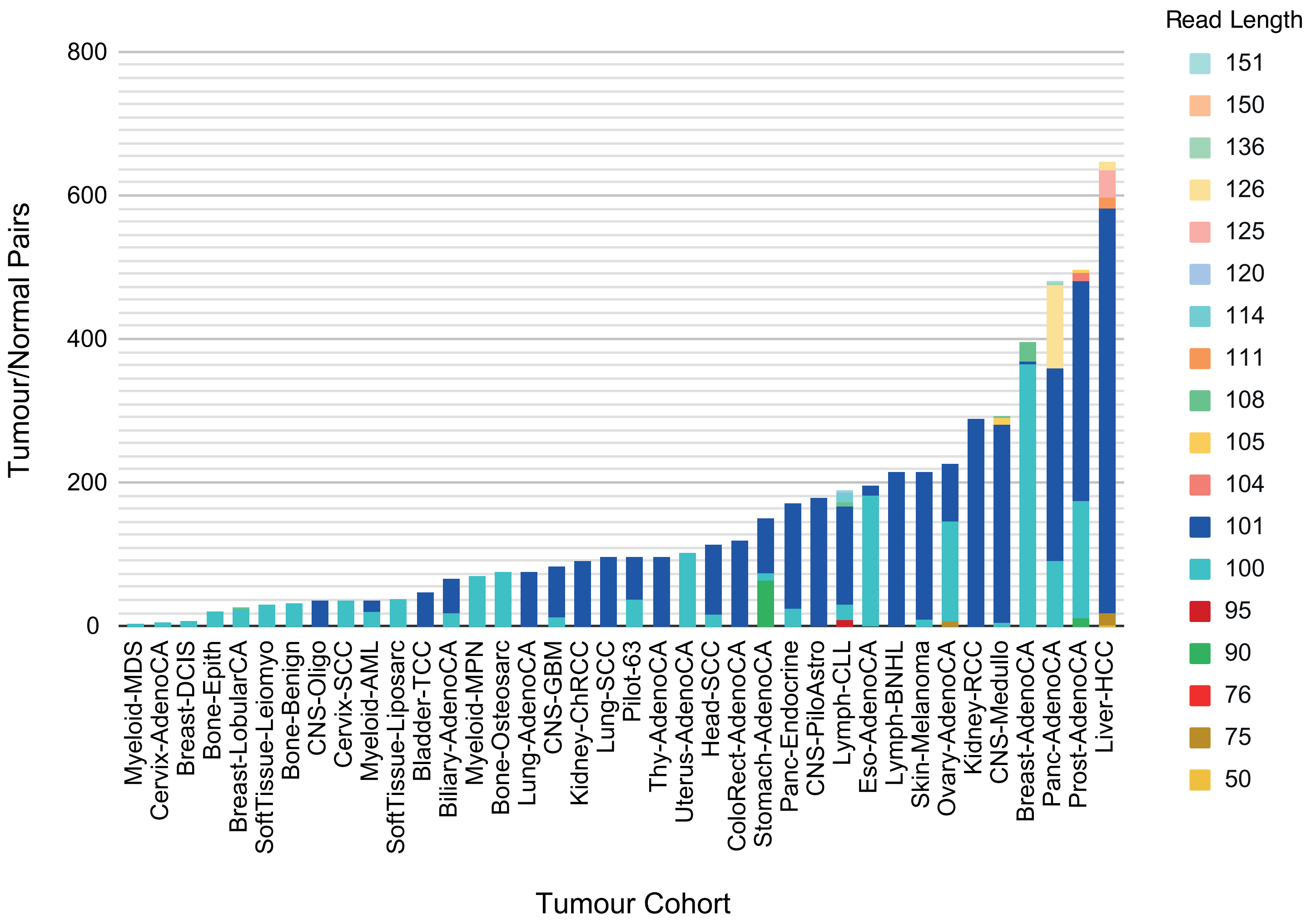

**Supplementary Figure 12. Performance of germline variant calling pipelines and the PCAWG consensus germline callset based on targeted resequencing.** Estimates for all panels are based on n=50 samples used for validation. (A) Precision, recall and F-score measures for SNP and Indel calls. (B) Genotype concordances considering homozygous reference sites. (C) Precision, recall and F-score measures for SNPs split by minor allele frequency (AF). (D) Precision, recall and F-score measures for indels split by minor allele frequency (AF), with common defined as variants with allele frequency > 20 %, mid defined as variants with 5% < allele frequency ≥ 20%, and rare defined as variants with allele frequency ≤ 5%.

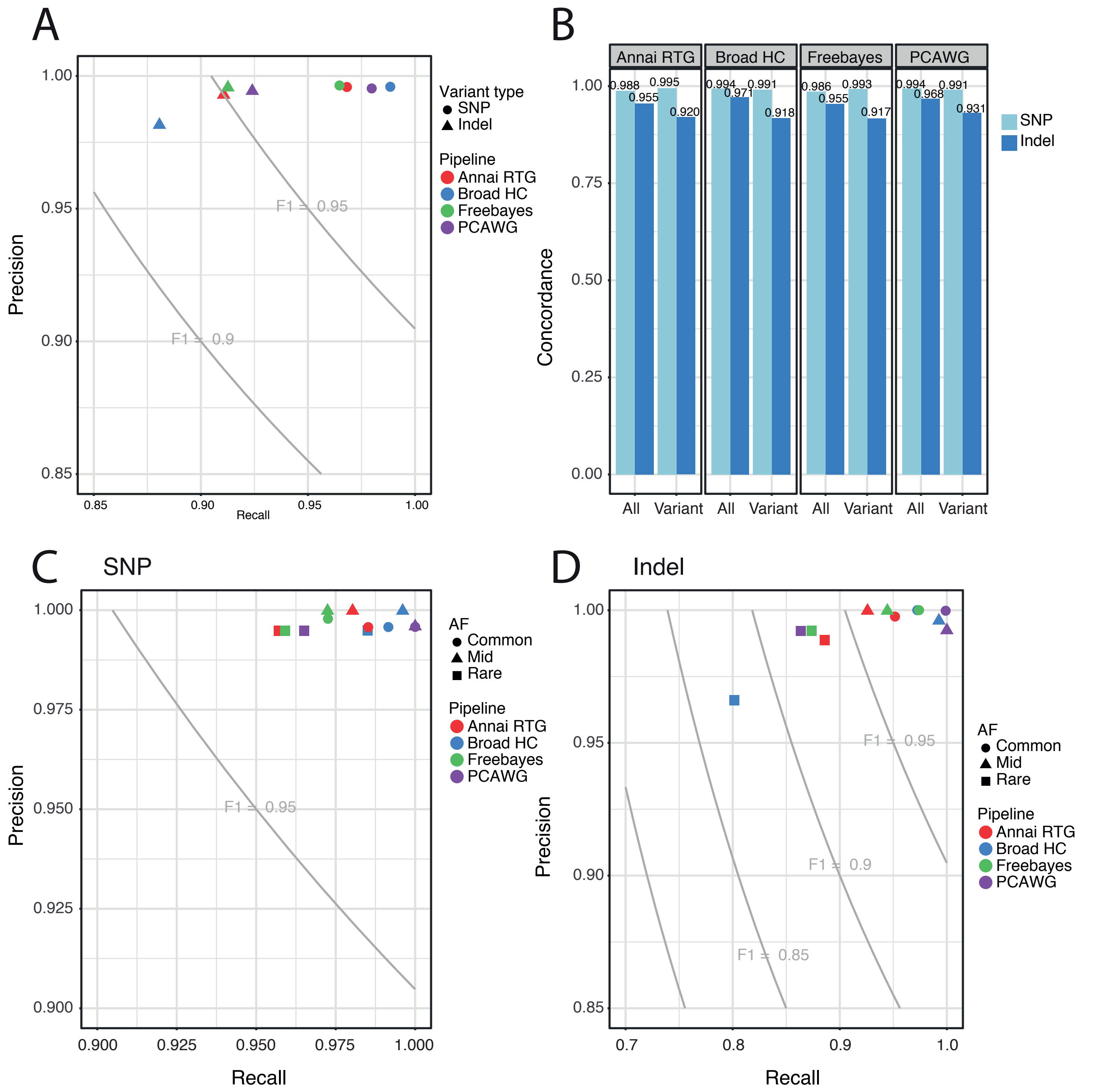

**Supplementary Figure 13. Example of a replication-based complex SV using long-read sequencing.** A tandem duplication (3,510 bp) on chromosome 2 with an inverted template insertion (356 bp) derived from chromosome 4 in-between (bottom panel). Alignment of the consensus sequence of locally assembled long reads to chromosome 2 and chromosome 4 of the human reference genome (top panel). Breakpoints are circled and marked as 1 (beginning of the tandem duplication), 2 (end of duplication) and 3 (templated insertion). For each breakpoint, the middle panel shows a snapshot of the Illumina short read data at the SV breakpoint. Paired-ends coloured in red and blue are supporting the translocations from chr2:chr4 and the coverage tracks show the expected increase and decrease of coverage from inside to outside of the duplicated segment.

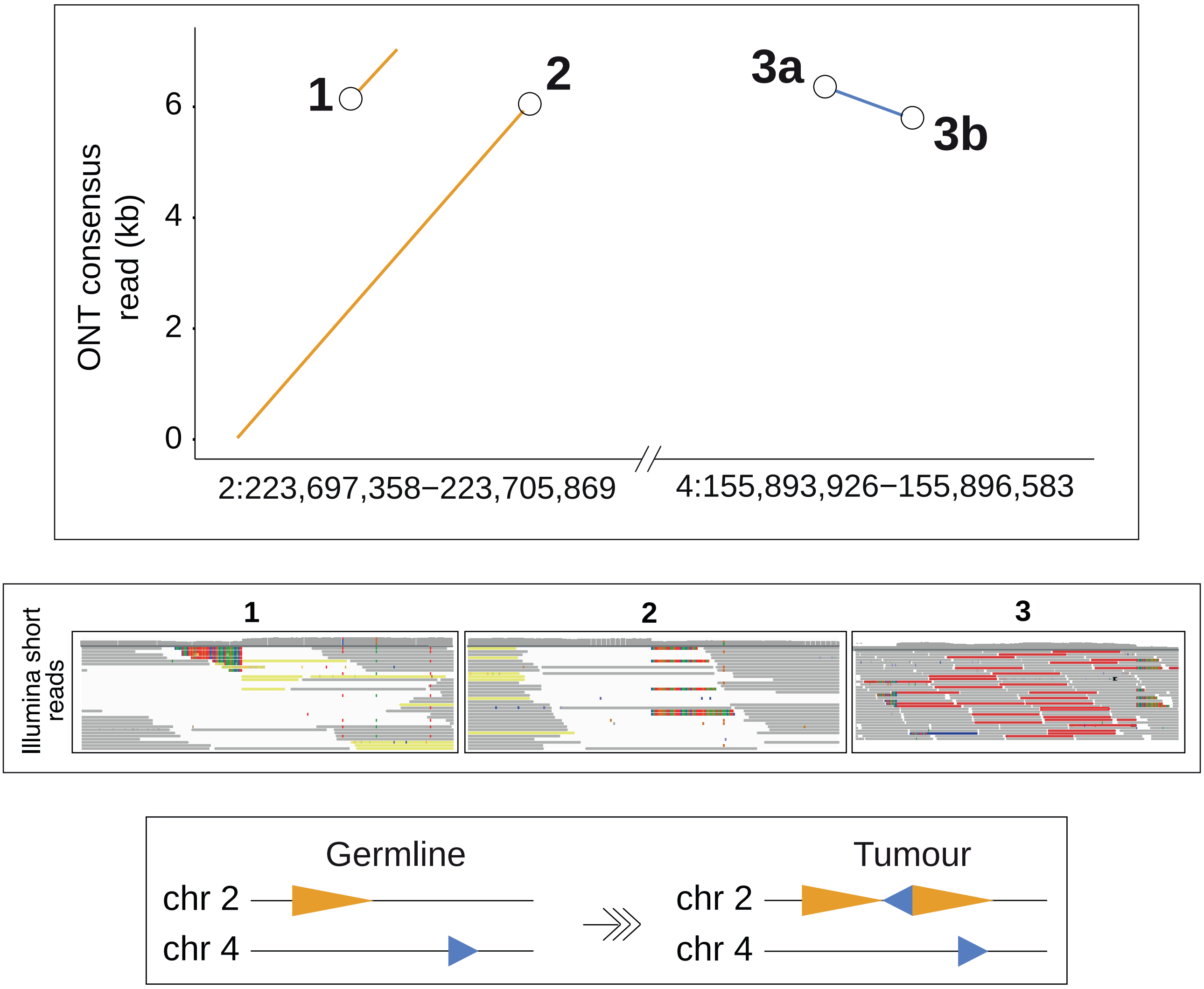

**Supplementary Figure 14. Summary of somatic call counts from the pilot-63 cases.** (A) Number of unique proposed variants called by sample, sorted in increasing order to show the range, for (left) SNVs and (right) indels. (B) The total distribution of somatic variants called by caller across all samples that were called on by all callers, colored by the total number of callers that made that call (the "concordance"). (C) The mean concordance of the calls for each sample, plotted vs the number of variants called by the core callers in the sample, with smaller points representing those samples that not all pilot callers made successful calls on. (Left) SNVs, (right) indels. The sample size is n=50 samples used for validation. The blue line represents the fitted line to the data, with the grey shaded area representing the 95% confidence intervals for the fitted line.

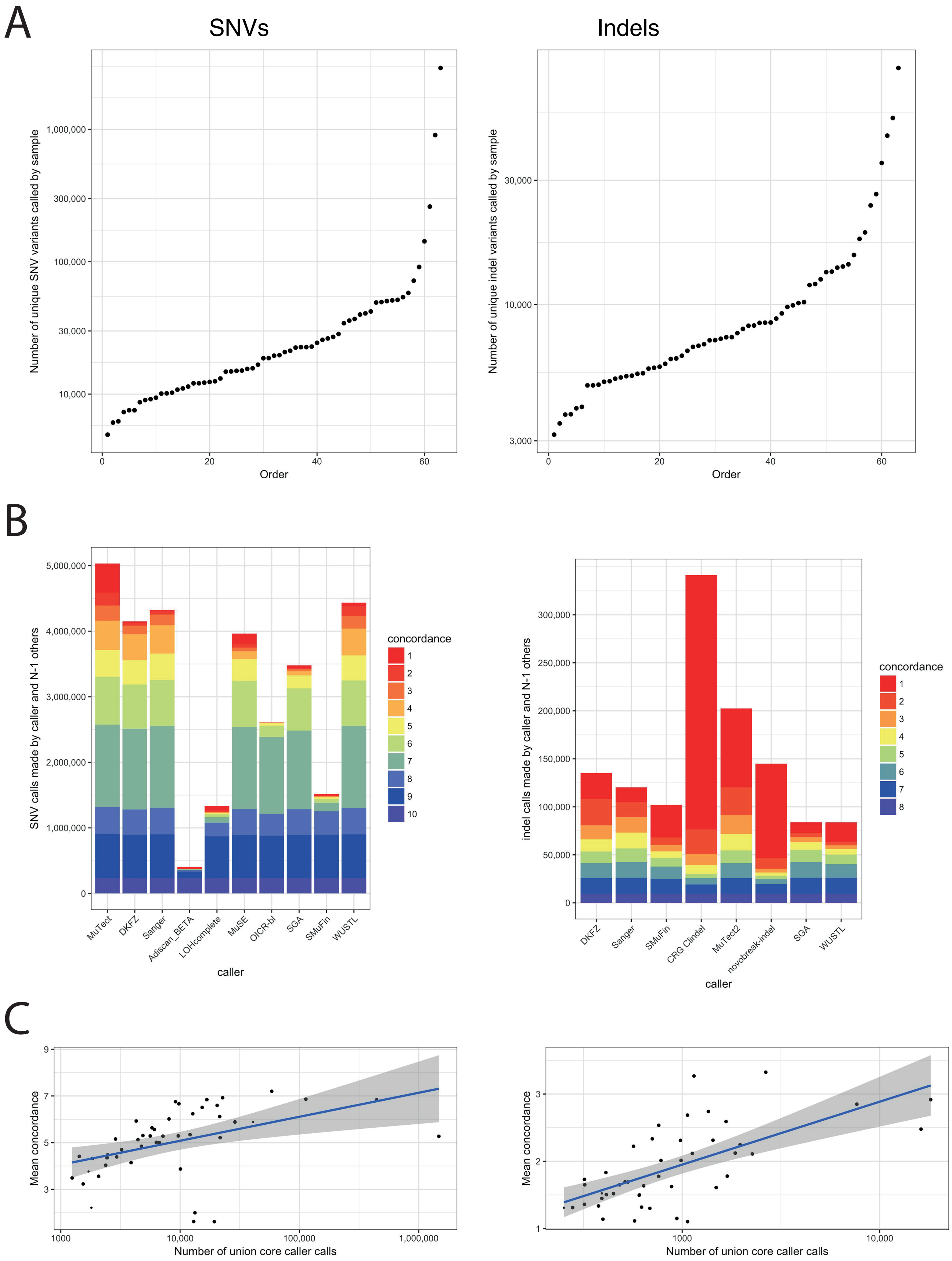

**Supplementary Figure 15. The total distribution of somatic indels called by caller across all samples** that were called on by all callers, colored by the total number of callers that made that call (the “concordance”), as with Supplementary Figure 13, but stratified by indel length. Note that even very short (1 or 2 bp) insertions or deletions show low degrees of concordance.

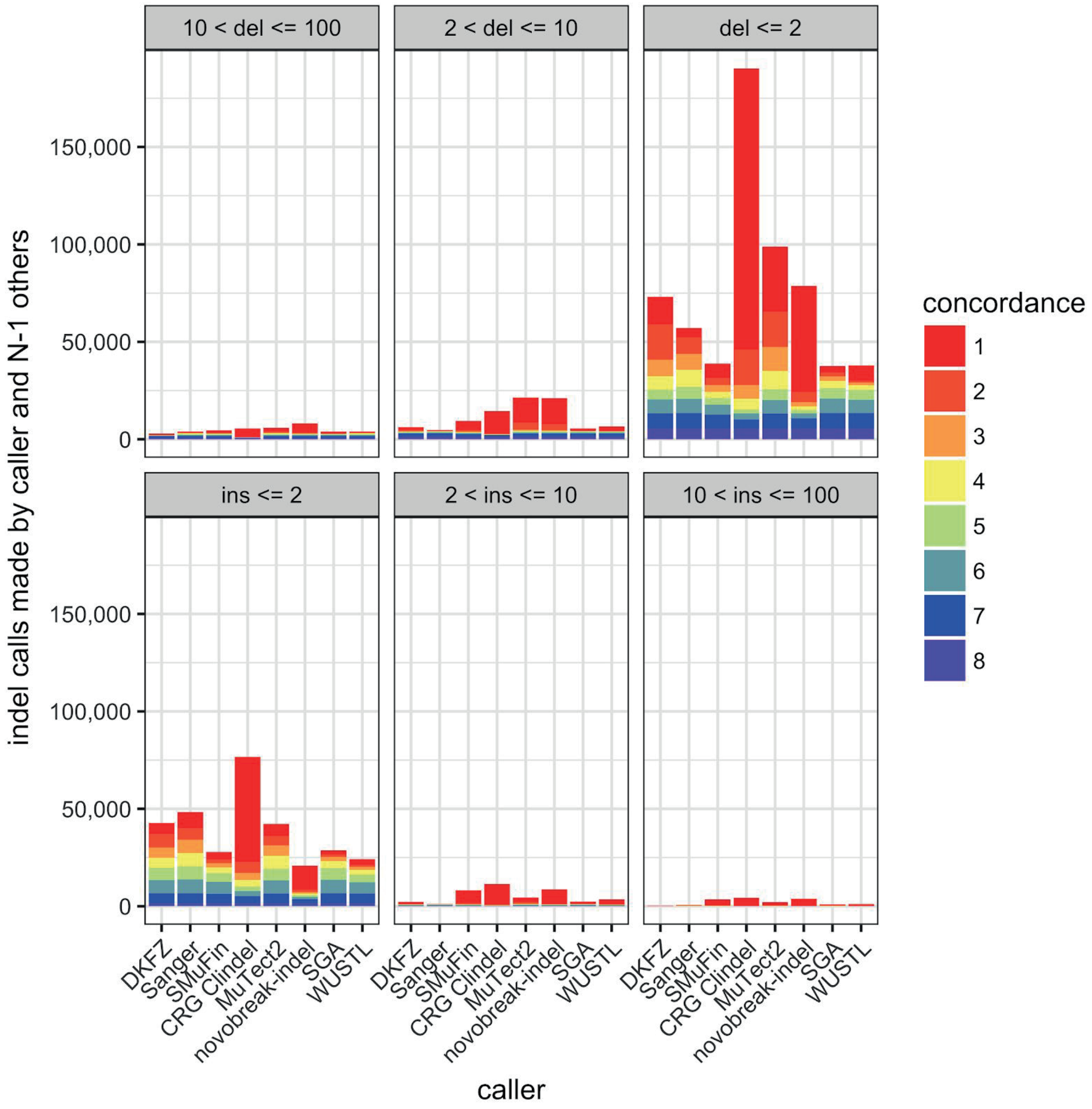

**Supplementary Figure 16. Accuracies of the individual callers on the validation samples.** (A) Box-and-whiskers plots showing the range of sensitivities, precisions, and F1 accuracies of the callers on individual samples (n=50), for SNVs (left) and Indels (right). The box denotes the interquartile range, with the median marked as a white point. The whiskers extend as far as the range or 1.5x the interquartile range, whichever is less. (B) Heatmap showing the same accuracies by caller and by sample, so that cross-caller correlations of accuracies can be seen. (C) A precision-recall plot of the overall accuracies across all validation samples for the callers, with contours shown of constant F1 accuracy. Note that the overall numbers are weighted more heavily for high-mutation-count samples, which particularly for SNVs tend to be easier to call and thus have higher accuracies. (D) As might be expected from the concordance data, accuracies were generally higher on more highly-mutated samples. The sample size is n=50 samples used for validation. The blue line represents the fitted line to the data, with the grey shaded area representing the 95% confidence intervals for the fitted line.

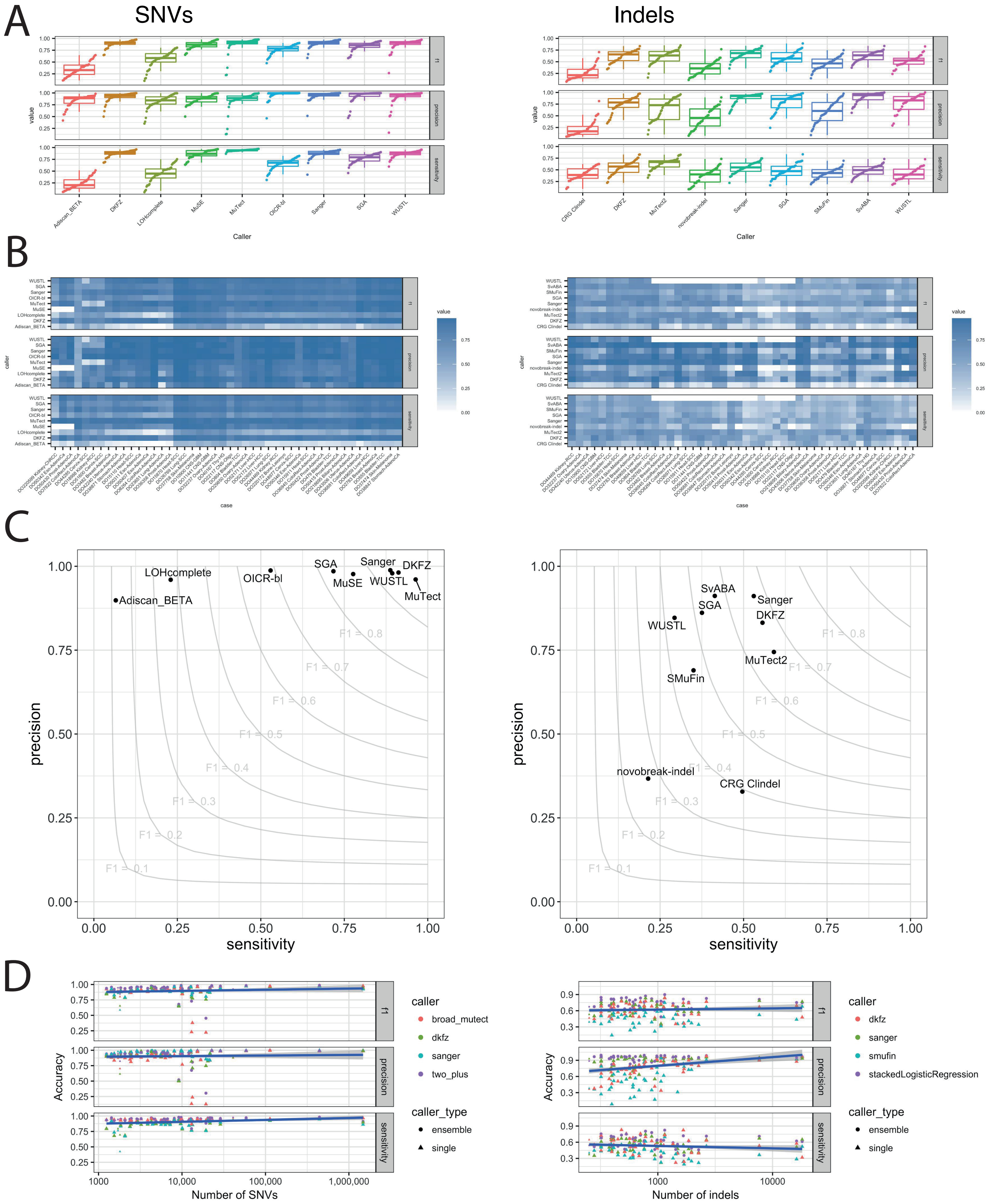

**Supplementary Figure 17. Smoothed average concordance of calls by inferred VAF, shown for SNVs and Indels.** The green and red lines represent smoothed average concordances, with the grey shaded area representing 95% confidence intervals for the fitted lines. Data are based on mutations assessed for validation in n=50 samples. Concordance is very low at low implied VAF because of small numbers of reads in support of the variant and varying filtering and noise estimation methods; concordance is also low at high VAF, due to the difficulty of distinguishing these from much more numerous germline variants.

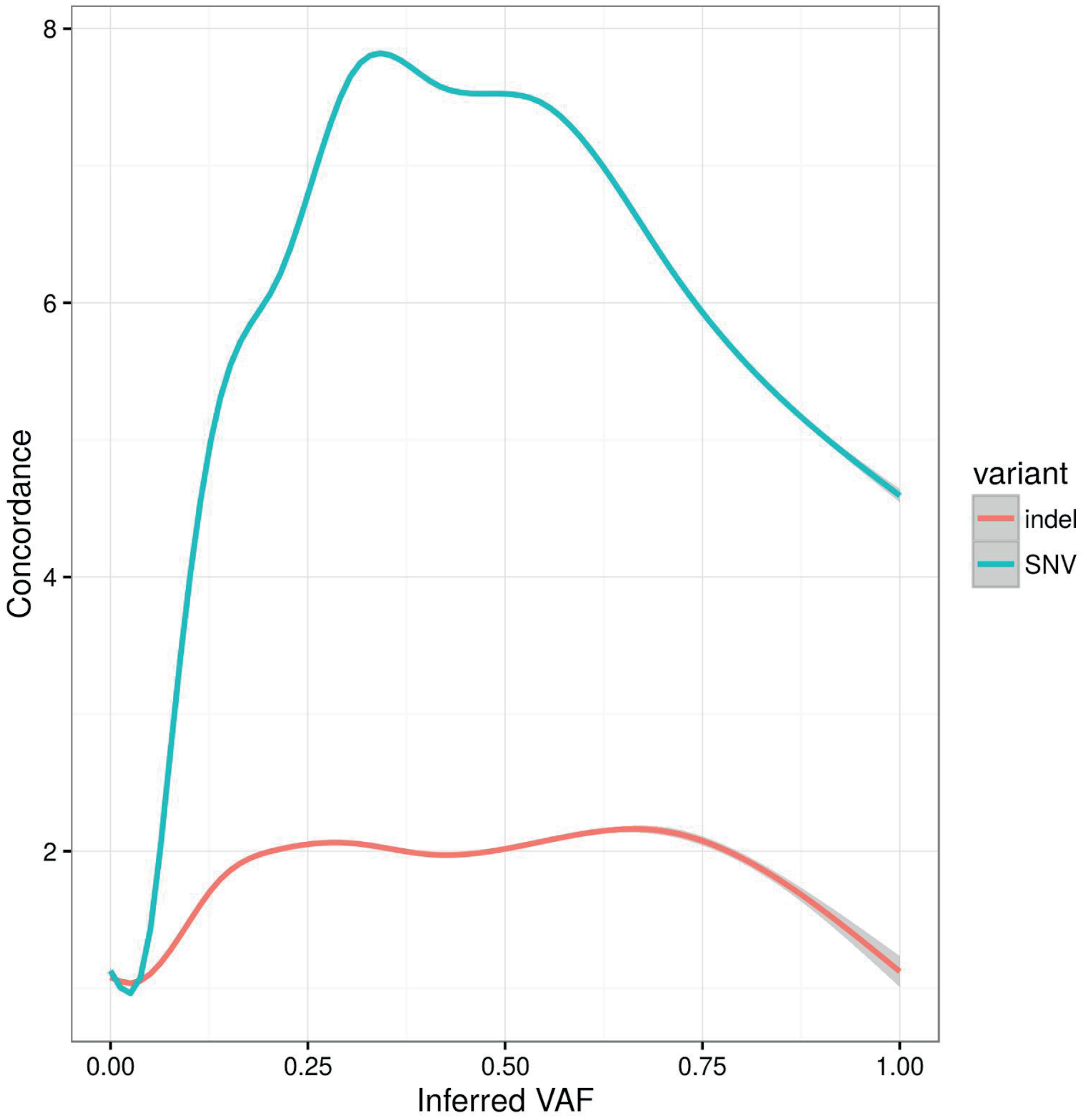

**Supplementary Figure 18. Raw deep-sequencing validation results.** (A) Depths achieved for deep-sequencing validation cases, for normal (red) and tumour (green), and indels (bottom) and SNVs (top). (B) Validation Tumour vs Normal VAFs for all calls with sufficient depth to make a call, coloured by the call made for all good samples, for SNVs (left) and indels (right). (C) Unused validation data from donor DO36352 for which normal sample appears to have been sequenced twice; this suggests an estimate for the false-positive PASS rate for the validation of under 1% (8/821 SNVs, 7/2083 indels). (D) Validated-true rate and fraction of calls vs concordance.

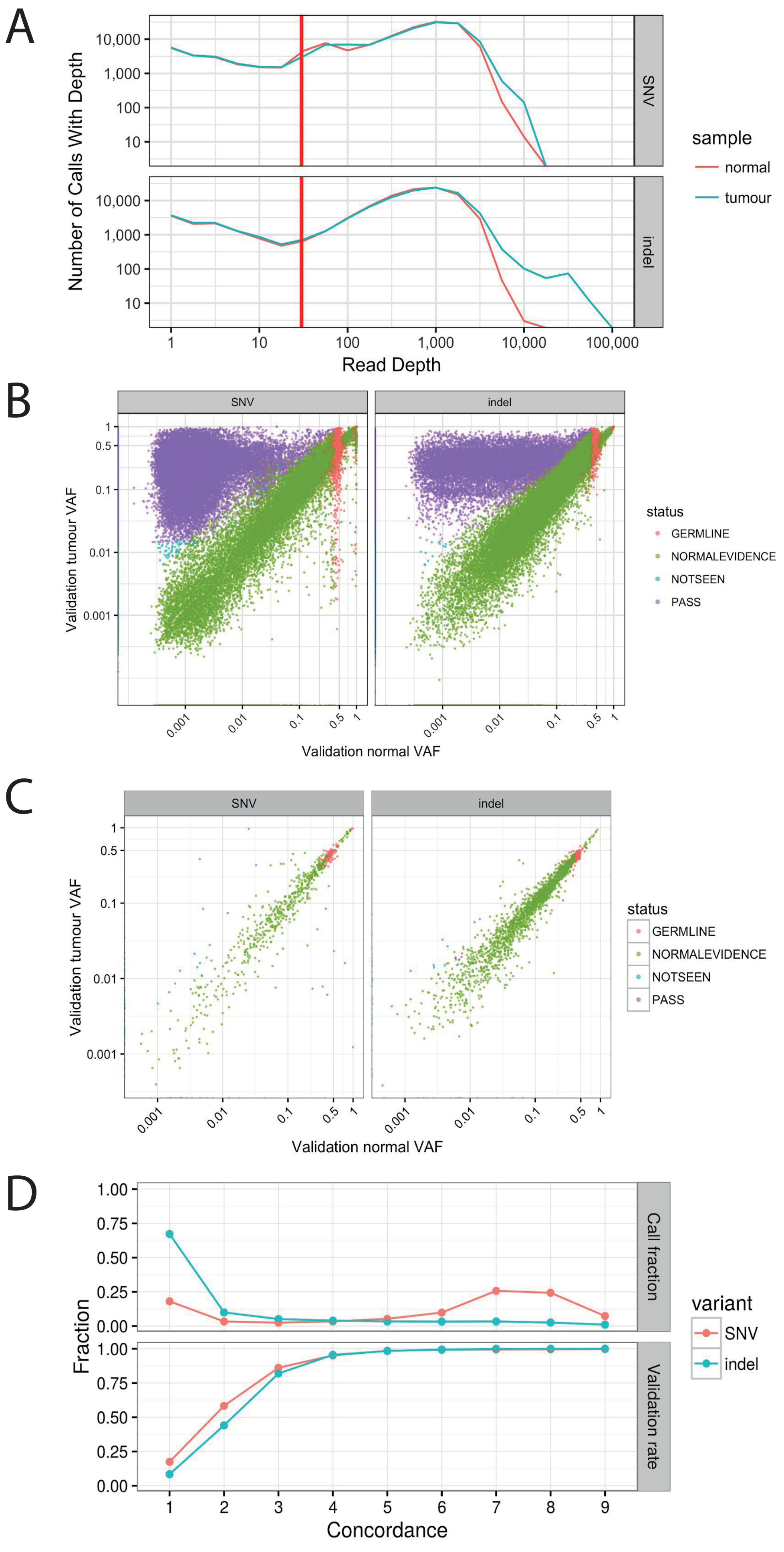

**Supplementary Figure 19. Accuracy of core callers and derived consensus models.** (A) plots of the accuracies of core callers and derived consensus models for SNVs (left) and indels (right). 4-fold cross-validation with random splits was used for the models requiring training; models were trained on 3 /4 of the samples, and applied to the remaining 1/ 4, until all samples were plotted. The box denotes the interquartile range, with the median marked as a white point. The whiskers extend as far as the range or 1.5x the interquartile range, whichever is less. The sample size is n=50 tumours used for validation. (B) Overall accuracies, broken down by VAF bins. The ensemble methods significantly improve accuracies at low allele fraction.

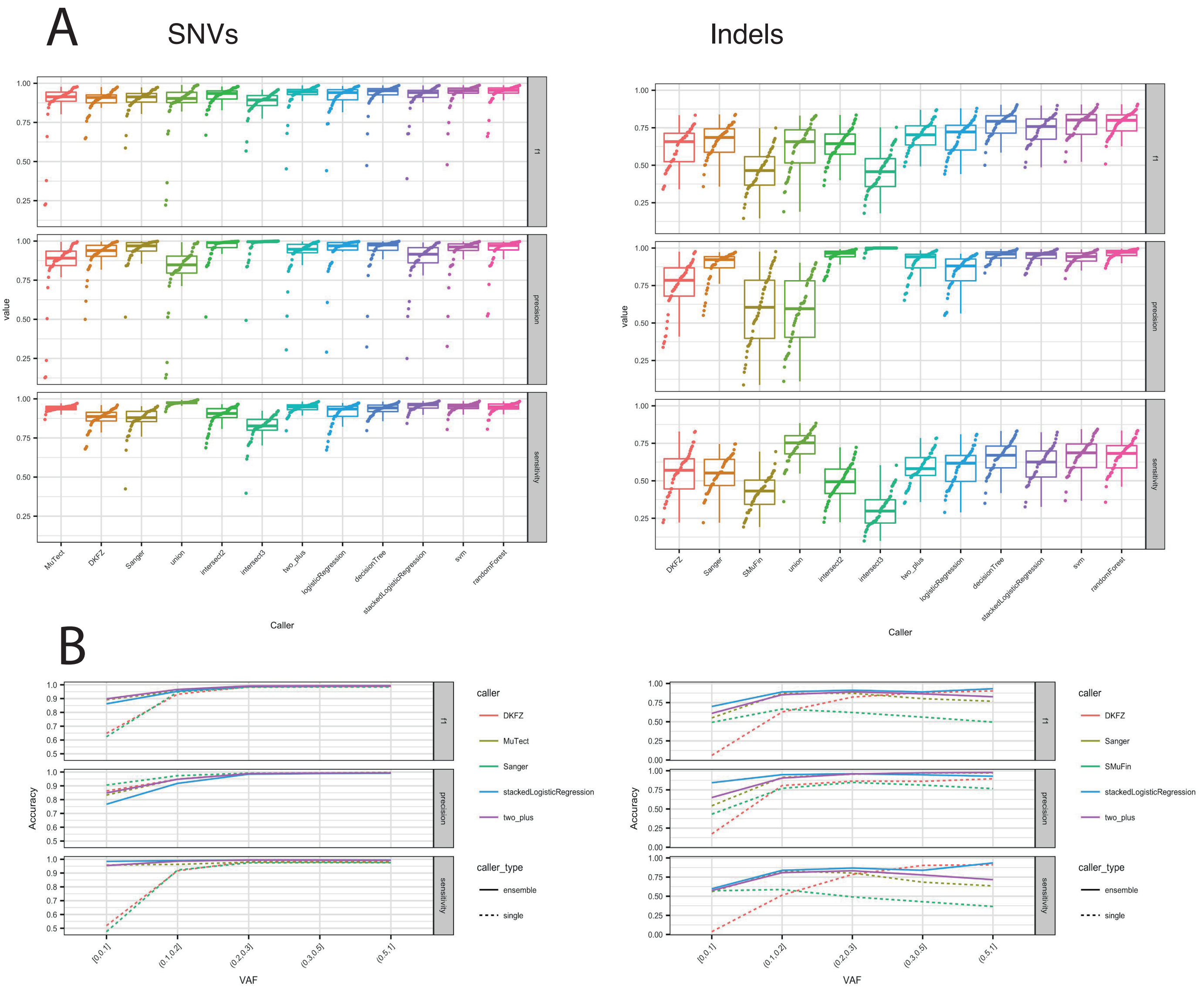

# Supplementary Methods

This file describes supplementary methods used in the ICGC/TCGA Pan-Cancer Analysis of Whole Genomes Cosortium paper. Individual data sets were accessioned at Synapse (https://www.synapse.org/), and are denoted throughout this document using with synXXXXX numbers; accessioned data sets are also mirrored at https://dcc.icgc.org as detailed in **Supplementary Table 4**.

## 1. Pilot-63 Analysis

These are methods applied to the Pilot-63 benchmark and validation exercise described in **Supplementary Notes 1**.

### 1.1 Validation Process

VCF files for the Pilot-63 calls were centrally collected and preprocessed for consistency by left-aligning indels. We merged the VCFs by case and annotated each call with its concordance as defined in the main text and the callers that made the call. For the 50 samples selected for validation we sampled calls stratified on caller and concordance using the following procedure. We aimed to select up to 10,000 sites per sample, split between 4000 SNVs, 3000 indels and 3000 structural variants. In practice many samples had fewer indels or SVs than the validation budget; in these cases we distributed the excess validation budget to additional indels or SNVs.

The sampling procedure follows that used by the DREAM[1] project. A "call budget" is established, with a desired number of calls equal across callers and prescribed across concordance bins; in our case we aimed for 30% of the call budget to be for private (concordance = 1) calls, and the rest evenly distributed among remaining concordance bins. This call budget can then be represented by a two-dimensional grid (**Supplementary Table 8**) as below.

Across callers, concordance bin by concordance bin, calls within each bin are uniformly randomly selected. When all callers are done, if some bins are now empty, the "extra" call budget is redistributed among concordance columns proportionately to the original distribution, and the process continues. Iterations over the full 2d grid are done until the budgeted number of calls have been selected. The procedure used is very stable; while rerunning multiple times selects different calls, the number of calls per bin changes only very modestly.

We split the 50 samples into 4 subsets for validation sequencing. We designed each capture array to minimize complexity for downstream data deposit based on national and international sequencing regulations. Thus, arrays 1, 3 and 4 were comprised solely of TCGA samples and array 2 had international ICGC samples along with 3 TCGA samples. Arrays 2, 3, and 4 were sequenced at Washington University of St. Louis and Array 1 was sequenced at the Baylor College of Medicine. (**Supplementary Table 9**)

## 1.2 Processing of Validation Data

The results of target capture and sequencing were processed largely following Nimblegen-recommended best practice[2], with the exception that duplicates were not removed due to the depths, nor were ends trimmed a second time. Reads were remapped to the PCAWGPanCancer reference using the standard PCAWG aligner and parameters, and then realigned around nearby germline indels using GATK[3] IndelRealigner and calls from HaplotypeCaller and the DKFZ pipeline.

Variant allele fractions (VAFs) for the tumour and normal validation sample were determined using bam-readcounts (https://github.com/genome/bam-readcount) for SNVs, and SGA somatic-variant-filters for indels. SGA somatic-variant-filters annotates realigns each read covering a candidate variant to determine whether it better supports the reference or the alternate allele.

## 1.3 Variant Classification using VAFs

From the read counts data above, variants were classified as follows:

- LOWDEPTH if the number of reads in the normal or tumour fell below our threshold of 30,
- NOTSEEN unless the number of evidential reads in the tumour was inconsistent with 1% noise with (p value < 0.02)
- STRANDBIAS if one of the strands was responsible for at least 90% of the evidence reads
- NORMALEVIDENCE if a Fisher exact test fails to rule out the normal VAF being consistent with the variant VAF within a factor of 2 (p < 0.01)
- GERMLINE if the number of evidential reads in the normal was consistent (binomial test) with a VAF of 0.95-1.0 or 0.45-0.5
- PASS otherwise

## 1.4 Calculating Per-Caller Accuracy

Using stratified sampling described above one can generate accuracy estimators that have greatly reduced variance for any given caller or difference between two callers than would be possible by (for instance) uniformly selecting from the entire call-set. However, those estimators require reweighting the results to reflect the populations of the bins the calls came from. Intuitively, if there were only two populations of calls, A with 100 calls and B with 1000, and one selected 10 calls from each to validate, the (say) overall true positive rate would not be simply the number of true positives divided by twenty, but would have to reflect the fact that each call selected from B "represents" 1000/10 = 100 calls from the population, and so have to be weighted more strongly than those from A which each represent 10. So, for any given caller, the (eg) true positive rate must be calculated by bins and reweighted:

$$\begin{aligned}\overline{\mathrm{TPR}} &= \frac{1}{N} \sum_{c \in \mathrm{concordances}} N_c \frac{\mathrm{TP}_c}{n_c} \\ &= \frac{1}{N} \sum_c N_c \mathrm{TPR}_c\end{aligned}$$

Where $N$ is the total number of calls, $N_c$ is the number per concordance bin, and $n_c$ is the number selected. False positive rates, *etc*, are calculated the same way, and from those rates accuracies are calculated.

For model selection, 20% of samples were held back, and training on the remaining 80% was performed by 5-fold cross-validation by case, with the model trained on 4/5 of the cases and accuracies calculated using the method above on the remaining 1/5. Once models were selected (2+/4 for SNVs, and stacked logistic regression for Indels), accuracies for the entire set of validation cases were calculated on the whole set for SNVs, and trained and evaluated using the same cross-validation procedure for indels. As with evaluation, models were not trained on calls in repeat-masked regions of the genome due to lack of validation data. The indel model was then trained on the entire 46-case validation set for application to the all PCAWG cases.

## 1.5 SNV/Indel Callers Used in Pilot-63 Exercise

This section describes non-production callers that were only used in the pilot and validation phase. Production callers are described in Section 2 of this document.

### *1.5.1 ADISCAN_Beta*

ADISCAN_Beta[4] uses three implicit suppositions: a genome in a tissue was homogenous; the proportions of an allele for each tester were respectively 0.5 and 1.0 at a heterozygous and homozygous position; and deviations of the allele frequency from 0.5 and 1.0 were derived from the errors during sequencing or alignment steps. The distance of pair allelic fractions (PAFs) at a genome position between two comparing testers was calculated as a tangential function, as follows:

$$a_i = \frac{A+1}{B+1} \text{ for tester 1, } x_i = \text{bin}[a_i] \qquad (1)$$

$$b_i = \frac{A\prime+1}{B\prime+1} \text{ for tester 2, } y_i = \text{bin}[b_i]$$

In equation (1), A, A’ and B, B’ were respectively the depth of reads for the minor and the major allele in the position i. The ratios were binned from 1 to 21. The ratio for the first group ranges from 0 to 0.0075 and the ratios for the subsequent bins increase 0.05 each time except the last steep where 0.075 was added. All other groups were evenly divided to get a 0.05 interval. $X_i$ and $y_i$ are the bins corresponding to $a_i$ and $b_i$,

$$t_i = \frac{1}{tan\ x_i/y_i} \frac{1}{tan[(22-y_i)/(22-x_i)]} \text{where } y_i > x_i\text{, or}$$

$$t_i = \frac{1}{tan\ y_i/x_i} \frac{1}{tan[(22-x_i)/(22-y_i)]} \text{otherwise} \qquad (2)$$

ADISCAN_Beta score = $log(40\ w\ t_i)\ - log(min(A, B, A', B'))C_1 -\ C_2$ (3))

In equation (2), 22 was a constant number that was generated by adding 1 to the largest bean number 21, and ti was the output of the tangential function of the ratio of allelic differences between two comparing testers.

In equation (3), five weights (w = 1.1 or 1.2; and 0.7, 0.8, or 0.9,) were specified. The first two weights (1.1 and 1.2) reward the cases with few or no sequencing errors in calling a homozygote, while the other three weights (0.7, 0.8 and 0.9) differentially penalize the cases with different extents of sequencing errors. When the ratio of reads for smaller allele was larger than 27.5%, the position was regarded the position as a potential heterozygote. The weights were biased toward cases in which the directions of pair allelic fraction (PAF) were opposite between two testers and their distance from the perfect heterozygotic status, 50 to 50. Positions with sequence depth below 0 in either tester were disregarded and not further considered for variant calling. Two constants were used to adjust ADISCAN_Beta scores within the range of 50.

### *1.5.2 LOHcomplete*

The LOHcomplete SNV pipeline utilizes GATK to call the genotypes of both control and tumour, which were jointly analyzed using custom Perl[5] or R. Two types of SNVs were identified, i.e. gain-of-heterozygosities (GOHs) where the genotypes of control and cancer are homozygous and heterozygous respectively and loss-of-heterozygosities (LOHs) where the genotypes of control and cancer are heterozygous and homozygous respectively.

### *1.5.3 OICR_bl*

Ensemble SNV calls from MuTect[6] (v1.14), RADIA[7] (v1.1.0) , Strelka[8] (v1.0.12), and SomaticSniper[9] (v1.0.2) were used (at least called by 3 out of the 4 callers or called by SomaticSniper and one other caller). See[1] for the detailed parameters of each caller. SNVs were further filtered by the following databases: dbSNP142[10] (modified to remove somatic and clinical variants, with variants with the following flags excluded: SAO = 2/3, PM, CDA, TPA, MUT and OM)[10], NHLBI exome sequencing study (Exome Variant Server, NHLBI GO Exome Sequencing Project, Seattle, WA; accessed March, 2013), 1000 Genomes Project (v3), Complete Genomics 69 whole genomes, duplicate gene database (v68)[11], ENCODE DAC and Duke Mappability Consensus Excludable databases[12] (comprising poorly mapping reads, repeat regions, and mitochondrial and ribosomal DNA), invalidated somatic SNVs from 68 human colorectal cancer exomes (unpublished data) using the AccuSNP platform (Roche NimbleGen), and the Fuentes database of likely false positive variants[13]. SNVs were whitelisted (and retained, independently of the presence in other filters) if they were contained within the Catalogue of Somatic Mutations in Cancer (COSMIC) database[14] (v71).

### *1.5.4 OICR_SGA*

SGA's graph-diff feature (from both fabc28ac, Sept 17 2014 and f1c64dd1, Jan 21 2015) and FreeBayes[15] (git commit 4233a239, Oct 7, 2014) were used to propose somatic variants, with graph-diff examining differences in the assembly graph structure between the paired samples. FreeBayes calls were filtered first for compatibility with the assembly graphs using

sga graph-concordance, and then with sga somatic-variant-filters to ensure a minimum read depth supporting the variant of 4, and a minimum allele fraction of 10%. The two call-sets were then merged with low-quality (lower than 20) variants filtered out. A final filtering step removed variants found in dbSNP build 142 but not COSMICv71.

#### *1.5.5 WUSTL*

Somatic SNVs detection was performed by running 3 callers on normal-tumour matched pairs: VarScan[16] (version 2.2.6 with default parameters except where "--min-coverage 3 --min-var-freq 0.08 --p-value 0.10 --somatic-p-value 0.05 --strand-filter 0"), SomaticSniper[9] (version 1.0.4 with default parameters except where "-q 1 -Q 20"), and Strelka[8] (version 1.0.14 with its bwa_default parameters, plus the extra parameter "--ignore-conflicting-read-names"). The raw calls were screened to remove common germline SNPs from a panel of whole-genome normals present at ≥ 0.1% MAF in dbSNP-138 and were filtered to meet the following criteria:

(1) All putative SNV calls were required to satisfy average mapping quality difference between var- and ref-supporting reads ≥ 30; maximum difference of average supporting read length between var and ref reads ≥ 25; maximum mismatch quality sum of ref-supporting reads ≤ 60; 5-bp maximum length of flanking homopolymer; minimum average relative distance from either end of reads ≥ 0.1; VAF < 2% with < 2 variant reads in the normal; minimum average relative distance to the effective 3´-end of read for var-supporting reads, ≥ 0.2;

(2) Calls generated by VarScan and Strelka were required to have minimum strandedness of 0.5%, maximum difference in average mismatch quality sum between the var and ref supporting reads ≤ 70, and VAF ≥ 2% with ≥ 2 var-supporting reads in the tumour;

(3) Calls generated by SomaticSniper were required to have depth of coverage between 20 and 75 in both normal and tumour, strandedness ≥ 5%; minimum average relative distance from the 3´-end of the read to be ≥ 0.2; maximum difference in average mismatch quality sum between the var and ref supporting reads ≤ 60, and VAF ≥10% with ≥ 6 var-supporting reads in the tumour.

Somatic indels detection was performed using Pindel[17,18] and Strelka (as above) on normal-tumour matched pairs. Pindel runs (version 0.2.5a3 with default parameters except where "-B 100 -A 20 -M 2 -e 0.03 -u 0.05 -b") were supplied with breakpoint events identified in either sample by BreakDancer (version 1.4.5 with default parameters). Candidate Pindel somatic calls were required to have VAF ≥ 10% with ≥ 1 read present in each direction and homopolymer length based on the reference genome not exceeding 6. The filtered Pindel calls, with complex indels having been removed, were merged with the passed call set from Strelka without additional processing.

#### *1.5.6 CRG-clindel*

ClinDel v0.1, a module of the SHORE platform[19], was used as described previously[20] to identify short indels up to 50bp independently in tumour (TD) and normal (ND) samples. Subsequently, variants identified in TD were flagged as somatic if no indel was observed in ND, and ND had at least 8x coverage at the respective position. ClinDel parameters were optimized for maximal sensitivity (‘discovery mode’), requiring for calls in TD a minimum coverage of 3x, minimum alternative allele count of 3, and minimum minor allele fraction

(MAF) of 0.05. This setting allows for identification of indels with low cancer cell fraction, at the expense of a potentially high false positive rate. In order to increase the sensitivity of identifying indels in ND (i.e. to minimize false somatic calls), we increased the prior probability for positions in which an indel was found in TD or with reported indels in dbSNP or 1000GP (optional parameter of ClinDel). Furthermore, a simple-sequence-repeat (SSR) filter step has been performed, but flagged indels have still been reported.

#### *1.5.7 Novobreak-indel*

novoBreak-indel[21] used the same settings for the indel sub-challenge of synthetic challenge 4 of the ICGC-TCGA DREAM Mutation Calling Challenge (https://www.synapse.org/#!Synapse:syn312572/wiki/). novoBreak (v1.03) was run under the parameters '-k31 -m2'. All the assembled contigs and unassembled short read pairs containing the novo-kmers were mapped to the reference using BWA-MEM [22]. The alignment results were sorted and the coordinates of indels were adjusted using SortSam of Picard (v1.107) (http://broadinstitute.github.io/picard/) and LeftAlignIndels of GATK [3,23,24] (v2.8-1), respectively. The "cigar" strings of the alignment results were parsed to generate an indel list (in VCFv4.1 format). Indels were further filtered using Database of Single Nucleotide Polymorphisms dbSNP (Build ID: 138, Available from: http://www.ncbi.nlm.nih.gov/SNP/) and low complexity regions identified with the mdust program (http://compbio.dfci.harvard.edu/tgi/). Finally, only indels with allele fraction greater than 1% were selected.

# 2. Whole Genome Sequencing Somatic Variant Calling

The following sections describe the somatic variant calling methods used during the production phase of the project.

## 2.1 Whole Genome Alignment

Beginning in early 2014, we compiled an inventory of matched tumour/normal whole cancer genomes in the ICGC Data Coordinating Centre and polled ICGC projects for whole genomes that they anticipated completing in the near future. Our PCAWG inclusion criteria for donors were: (i) a matched tumour and normal specimen pair; (ii) a minimal set of clinical information including patient age, sex and histopathological diagnosis; and (iii) characterisation of tumour and normal whole genomes using Illumina HiSeq platform short paired-end sequencing reads (**Supplementary Table 1**). The great majority (93%) of samples were sequenced with either 100 or 101 bp reads. Longer or shorter read lengths were applied to the remainder of the samples. Notably, within the Panc-AdenoCA cohort, 115 of 480 samples used 126 bp reads (24% of this cohort, 2% of total), and within the Stomach-AdenoCA 64 of 150 samples used 90 bp reads (60% of cohort, 1.4% of total) **(Supplementary Table 10; Supplementary Figure 11**). Most of the tumour samples came from treatment-naïve, primary cancers, but a small number of donors with multiple samples of primary, metastatic and/or recurrent tumour (**Supplementary Table 1)**.

All reads, from per-lane FASTQs or unaligned BAMs, were aligned with bwa-mem 0.7.8-r455 with all alignment scores output and using the default alignment algorithm options against human reference hs37d5 (available at https://dcc.icgc.org/releases/PCAWG/reference_data/pcawg-bwa-mem).

## 2.2 Variant Calling Pipelines Used During Production

This section describes the pipelines applied to somatic variant calling. Following application of these pipelines, variants were merged (**Section 2.4, Supplementary Notes Section 2**), and the resulting consensus variant set subjected to a set of filters and other QC steps described in **Section 2.5** and illustrated in the process flow diagram of **Supplementary Figure 2.**

### *2.2.1 DKFZ Pipeline*

*Single nucleotide variants.* Calls were generated by samtools[25] and bcftools[26] (version 0.1.19), and potential variants called in the tumour were followed by a lookup of the corresponding positions in the control. To enable calling of variants with low allele frequency we disabled the Bayesian model (by setting -p 2). Thus, all positions containing at least one high quality non-reference base are reported as candidate variant. The resulting raw calls were categorized into putative somatic variants and others (artefacts, germline) based on the presence of variant reads in the matched normal sample. The frequency of all putative somatic variants was then refined by checking for potential redundant information due to overlapping reads and precise base counts for each strand were determined. All variants were annotated with dbSNP141, 1000 Genomes (phase 1), Gencode Mappability track, UCSC[27] High Seq Depth track, UCSC Simple-Tandemrepeats, UCSC Repeat-Masker, DUKE-Excluded, DAC-Blacklist, UCSC Selfchain. The confidence for each variant was then determined by a heuristic punishment scheme taking the aforementioned tracks into account. In addition, variants with strong read biases according to the strand bias filter were removed. High confidence variants were reported.

*Small insertions and deletions.* Platypus[28] version 0.7.4 was used. All variants indicating an Indel were categorized into putative somatic and other based on the genotype likelihoods (matched genotype 0/0 for somatic indels). High confidence somatic variants were required to either have the Platypus filter flag PASS or pass custom filters allowing for low variant frequency using a scoring scheme. Candidates with the badReads flag, alleleBias, or strandBias were discarded if the variant allele frequency was <10%. Additionally, combinations of Platypus non-PASS filter flags, bad quality values, low genotype quality, very low variant counts in the tumour, and presence of variant reads in the control were not tolerated.

### *2.2.2 EMBL Pipeline*

*Structural variants.* We used DELLY[29] v0.6.6 to call simple and complex structural variants (SVs). A high-stringency SV set of somatic calls were derived by requiring at least four supporting read pairs, with the additional requirement for split read support for SVs smaller than 500 bp. Somatic SVs were filtered for absence in the paired (normal) control tissue. Additionally, we removed SVs detected either in ≥1% of a set of 1,105 germline samples from healthy individuals from the 1000 Genomes Project phase I or in the panel of normal samples

constructed from the DELLY's consensus germline SVs called in PCAWG normal tissues samples (see **Section 3**). Multi-tumour sample analyses were analyzed jointly together with the paired normal sample to improve SV discovery, and subsequently split into individual tumour samples.

*Copy number alterations.* We used ACEseq[30] v1.0.189 to call somatic copy number alterations and estimate tumour cell content and ploidy. Allele frequencies in tumour and matched normal were obtained for all SNPs recorded in dbSNP[10] (build 135), and positions with BAF values between 0.1 and 0.9 in the normal were assumed to be heterozygous in the germline. To improve sensitivity for the detection of allelic imbalances, heterozygous and homozygous SNPs were phased with IMPUTE[31] (version 2). In addition, the coverage for 10-kilobase (kb) windows was recorded for tumour and matched control and corrected for GC content- and replication timing-dependent coverage bias. The genome was segmented using the R package PSCBS[32] at change points in the coverage ratio and BAF signal. SV breakpoints identified by DELLY were incorporated as predefined segment borders. Segments were clustered according to coverage ratios and BAF values using *c*-means clustering. The R package mclust was used to determine the optimal number of clusters based on the Bayesian information criterion. Small segments (<9 kb) were attached to the more similar neighbor. Finally, tumour cell content and ploidy of a sample were estimated by fitting different tumour cell content and ploidy combinations to the data. Segments with balanced BAF values were fitted to even-numbered copy number states, whereas unbalanced segments could also be fitted to uneven copy numbers. Finally, estimated tumour cell content and ploidy values were used to compute the total and allele-specific copy number for each segment. Full details of the ACEseq processing steps can be found in the ACEseq documentation[33]).

### *2.2.3 Sanger Pipeline*

*Single nucleotide variants.* Sanger SNV calls were generated using CaVEMan[34] (v1.5.1). CaVEMan takes copy number segments, purity and ploidy information from the CNV caller ascatNgs19 to improve calling in regions of aberrant copy number. CaVEMan did not analyse known problematic regions based on the UCSC High Seq Depth track or variants on the non-primary chromosomes. The filtering phase, cgpCavemanPostProcessing (v1.0.2), uses germline calls from cgpPindel[18], a panel of aberrant sites generated from 59 blood normals and a set of engineered filters to reduce the data to a high confidence set of somatic calls. The filters are described in detail on the cgpCaVEManPostProcessing wiki page[35] and include read-depth, phasing, positional and directional bias, repeats (various classes).

*Small insertions and deletions.* We used cgpPindel v1.5.7 to call indels. This uses a slightly modified version of pindel v2.0 with custom read selection that works better with the standard PCAWG mapping pipeline, and additional post calling filtering, including the normal panel of aberrant sites used with the Sanger SNV calls and UCSC High Seq Depth regions. The read selection component has been updated to handle the increased incidence of split read mappings caused by indel events in BWA-mem data (while still being compatible with BWA-backtrack). As with the Sanger SNV calls a panel of aberrant sites generated from 59 blood normals is used to filter out common artefacts along with a set of engineered filters. Full details of the variant filters can be found on the cgpPindel wiki page[18] and include likely germline, depth dependant mutant fraction and repetitive slip filters.

*Structural variants.* We used BRASS[36] v4.012 to call simple and complex genomic rearrangements along with grass[37] to identify the likely consequence of the event. As in CaVEMan and cgpPindel the raw input data is filtered to remove regions corresponding to the UCSC High Seq Depth Regions, along with the exclusion of non-primary chromosomes, prior to identifying groupings of incorrectly paired reads. The generated groups that did not present in the matched normal sample were further filtered against groupings identified in the 59 blood normals described earlier. Surviving groupings were sanitised for fold-back artefacts, mismapping and microbial and viral contamination.

*Copy number alterations.* We used ascatNgs[38] v1.5.2 to call somatic copy number alterations. Due to the unguided execution of this algorithm it is expected that a subset of results would be suboptimal or fail to resolve. Where ascat was unable to find an appropriate solution a global 5/2 Tumour/Normal copy number was generated with normal contamination value of 0.3 to be passed into CaVEMan. Under normal conditions inspection and refitting is recommended, however, as Battenberg results were being manually reviewed for both general and sub-clonal copy number this was deemed sufficient to be used for input for SNV calling by CaVEMan.

### *2.2.4 Broad Pipeline*

*Single nucleotide variants.* Raw candidate SNV calls were produced by MuTect[6]. MuTect explores read evidence for a variant in tumour and matched normal samples and utilises a Bayesian classifier to detect somatic mutations with very low allele fractions, requiring only a few supporting reads. A log likelihood ratio statistic for each variant is calculated based on consideration of several confounding aspects: levels of foreign DNA contamination (estimated by ContEst[39]) and normal population samples-based statistics. Subsequent filtering within MuTect is based on bias metrics, such as mapping quality of reads, strand bias, read position bias and clustering of variants. These raw candidate calls are then filtered in the pipeline by the following Broad filters: Realignment Filter, Panel of Normals filter, and Orientation Bias (OxoG[40]) filter. These filters were later applied to the PCAWG consensus somatic mutation calls dataset separately.

*Small insertions and deletions (pilot-63 phase).* MuTect2 performs local assembly of all reads surrounding potential somatic variants (both SNVs and indels, but only indels were selected for the PCAWG pilot) to generate candidate haplotypes and realigns reads to these haplotypes with a pair hidden Markov model to obtain a likelihood of each read versus each haplotype. It inputs these likelihoods into a probabilistic model for the likelihood of variants implied by the assembled haplotypes. To eliminate artefacts due to library preparation, sequencing, and mapping error, MuTect2 filters variants based on mapping quality of reads, strand bias, gap and read end proximity, read position bias and clustering of variants. Calls are then filtered in the pipeline by the following Broad filters: Realignment Filter, and the Panel of Normals filter.

*Small insertions and deletions (production phase).* SvABA[41] calls variants by performing genome-wide local assemblies of gapped, clipped, unmapped and discordant read pairs. Contigs are assembled with a modified version of the SGA assembler. The assembled contigs are aligned to the reference to identify contigs with gapped alignments (indicating short insertions and deletions) or with multi-part alignments (indicating structural variations). Reads are also aligned to the contigs to identify the read support for either the tumour or

normal allele. The variants are then genotyped and classified as either germline or somatic. SvABA is freely available at https://github.com/walaj/svaba.

### *2.2.5 MuSE Pipeline*

*Single nucleotide variants.* MuSE[42] version 1.0rc calls are made in two steps, which requires (1) the indexed reference genome FASTA file, (2) the binary sequence alignment/map formatted (BAM) sequence data from the pair of tumour and normal DNA samples, and (3) the dbSNP variant call format (VCF) file that should be bgzip compressed, tabix indexed and based on the same reference genome as (1). The first step, 'MuSE call', takes as input (1) and (2). The BAM files require aligning all the sequence reads against the reference genome using the Burrows-Wheeler alignment tool (BWA[43]), with either the backtrack or the maximal exact matches (MEM[22]) algorithm. In addition, the BAM files need to be processed by following the Genome Analysis Toolkit (GATK) Best Practices that include marking duplicates, realigning the paired tumour-normal BAMs jointly and recalibrating base quality scores. To speed up 'MuSE call', the WGS data may be splitted into small blocks (<50Mb) by using the provided option either '-r' or '-l', and concatenating all the output files by the Linux command CAT. The second step, 'MuSE sump', takes as input the output file from 'MuSE call' and (3). There are two options for building the sample-specific error model. One is applicable to WES data (option '-E'), and the other to WGS data (option '-G').

### *2.2.6 SMuFIN Pipeline*

*Small insertions and deletions.* SMuFIN[44] was used for indel calling. For this analysis, SMuFin was run taking BAM files from whole genome sequences as input, corresponding to the tumour and normal samples of the same individual. The complete PanCancer set, which includes the pilot validation subset, was executed on an HPC environment using OpenMPI over an average of 32 nodes (2xIntel SandyBridge, 8-core/2.6GHz), taking six hours of execution time per genome pair in the Marenostrum 3 Supercomputer. Input and output files were stored locally, and then integrated into the PCAWG data sets. The version of SMuFin used here, modified 2014-10-26, has no algorithmic tuning parameters, and the method is as described in Moncunil *et al*[44].

## 2.3 Consensus Somatic SNV/Indel Annotation

Prior to merging, all called SNVs and indels were annotated according to their predicted functional impact on coding regions and other functional elements. Each variant was labeled using the union of annotations.

Within the Sanger pipeline, we ran VAGrENT[45] v2.1.2 which used Ensembl release 74 as a base for the annotation. Variants were compared to all overlapping transcripts and described using HGVS syntax and Sequence Ontology terms, two annotations were then recorded per variant. The first was a default against the most representative transcript, typically the longest transcript with a CCDS identifier. The second was a worst case, an annotation described with the most disruptive Sequence Ontology term.

Within the DKFZ and EMBL pipelines, we ran the ANNOVAR[46] package downloaded on 12 November 2014 to perform gene-based annotation using the GENCODE database (v19). It was run using default parameters.

## 2.4 Merging of WGS somatic variant calls

Following completion of the somatic mutation calling and annotation pipelines described above, all candidate somatic variants were subjected to the series of merging steps to generate an accurate consensus call set. All scripts are available as Dockstore packages, as described in **Supplementary Table 3.**

### *2.4.1 Somatic SNV and indel Merging*

The SNV and indel call sets produced by each pipeline were merged together to create a single consensus call set using the SNV-MERGE[47] script. This script implements the consensus model strategies described in **Supplementary Notes 2.** For SNVs, the script uses a simple "2+/4" approach where calls seen by at least two callers were selected as consensus calls. For indels, SNV-MERGE implements a previously-published stacked logistic regression method[48].

### *2.4.2 Somatic SV Merging*

To achieve the merged set of somatic SVs, somatic SV calls from SvABA (Broad pipeline), DELLY (DKFZ pipeline), BRASS (Sanger pipeline) and dRanger (Broad pipeline) were combined into a union set as described here. For each tumour/normal pair, high confidence SVs from each caller were pairwise joined based on SV class and position (identical paired-end read orientation), allowing 200 bp of slop at the breakpoints. Calls were merged using a graph structure, by inserting an edge in the graph for each joined pair. High confidence merged calls were derived by requiring at least two out of the four callers to support an SV. For each merged SV, consensus breakpoint positions were chosen based on proximity to the consensus breakpoint. The call-set was filtered to remove artefacts related to transposable element insertions (using curated transposable elements master copy hotspots, available in the PCAWG SV merge Docker container, and adding a slop of 15 Kbp), and fold-back inversion artefacts (using a list of fold-back inversion artefacts provided by the Sanger pipeline, adding a slop of 200bp). Additionally, pseudogenes and cDNA both contain DNA with exon-exon junctions, and these can manifest as deletions between exons of the affected gene. To remove such pseudogene and cDNA carry-over artefacts, we identified and removed exon-exon spanning SVs involving genes containing at least two exon-exon bridging deletions.

### *2.4.3 Somatic Copy Number Alteration Merging*

Consensus copy number profiles were constructed from the output of six copy-number aberration (CNA) callers, as detailed in[49]. We first segmented each cancer's genome into regions of constant copy number, separated by breakpoints denoting copy-number shifts. These breakpoints were based on PCAWG's consensus structural variants (SVs)[49], which were complemented with high-confidence breakpoints reported by multiple CNA callers obtained by summarising the intersection of genomic regions where these callers agreed a breakpoint must exist.

We then used the six CNA callers to determine the allele-specific copy-number for each consensus segment, requiring the callers to use a separately established consensus purity and ploidy value, and subsequently applied a multi-tiered approach to combine the callers' results into consensus profiles. Finally, segments were assigned a level of confidence based on the

degree of consensus on the major and minor allele copy-number states. On average, a strict majority of callers agreed on 93% of each cancer's genome.

## 2.5 Variant Call Set Quality Control and Flagging

Following merging, a series of algorithms were applied to somatic variants to detect and flag suspect samples and individual variants. These flagged sets were then manually reviewed to develop inclusion/exclusion rules described in the next section. Scripts that implement these steps are available as Dockstore images at the locations described in **Supplementary Table 3.**

### *2.5.1 Tumour in Normal Estimation*

Our ability to distinguish somatic variants from germline variants is adversely affected by contamination of tumour cells in the normal sample. We estimate the level of tumour-in-normal contamination (TiN), using deTiN[50] based on two key signals. Presence of tumour cells in the normal sample can be observed as "shadows" of somatic mutations in the normal sample. This is quantified by fitting the allele counts in the normal sample as a function of the allele counts and local copy number in the tumour. The slope of the fit is one estimate of TiN. The other key signal is the allele shift of germline heterozygous SNPs in the normal away from 50% at sites with tumour copy number variation. The second TiN estimate is the fitted allele fraction shift. Each signal provides a TiN likelihood curve (0>=TiN>=1, where TiN is defined relative to the tumour sample) ) and the two likelihood curves are combined into a single TiN likelihood curve and the final TiN estimate for each sample.

### *2.5.2 Germline site somatic mutation filter*

Somatic mutation calls can suffer from "bleed-through" of germline variants, which can be due to insufficient total coverage or lack of sufficient presence of the alternate allele in the matched normal sample. Both of these cases can lead to insufficient power to identify a germline SNP in the matched normal, while a somatic mutation may be called in the often more deeply sequenced tumour sample.

The MuTect mutation caller accounts for this problem by requiring higher read coverage in the normal sample to call a somatic mutation at a known site recorded in the dbSNP database supplied at runtime, compared to a somatic mutation at a non-dbSNP site[6]. A post-filtering step similar to this strategy was applied to the entire consensus mutation call set.

Somatic mutations from the May 2016 consensus SNV MAFs (filtered by the "at least two out of four callers" criterion) were subjected to overlap with >14M common (>1%) variants obtained from the 1000 genomes project. Of ~54M somatic SNV variants, 0.64% were classified as "possible germline risk" based on this overlap with common 1000 genomes variants.

Of these "possible germline risk" SNVs, 91.5% were flagged and removed by the Broad's panel of normals (PoN).

"Possible germline risk" somatic mutations that passed the PoN-filter were subjected to the following filtering strategy:

SNVs with more than one alternate read in the matched normal (n_alt_count >1) were flagged and filtered on any chromosome (78 mutations failed)

SNVs on autosomes were flagged and filtered if their total coverage in the normal sample was less than 19 reads (n_alt_count + n_ref_count <19) (563 mutations failed)

Due to expected low coverage, the coverage criterion from 2. was not applied on the Y chromosome

Due to expected low coverage, the coverage criterion from 2. was not applied to the X chromosome in *male* patients

Coverage filtering as in 2. was applied to chromosome X for *female* patients (945 mutations failed)

Mutations for 5 patients without reported donor sex were genotyped from sequencing data and filtered with criteria 4 or 5 as appropriate (11 mutations failed).

Read counts for the matched normal samples (n_alt_count, n_ref_count) were obtained from MuTect's force-called call_stats files produced with the OxoG docker on the union call set. As those calls were only available for SNVs, indels were not included in this germline site filter analysis.

### *2.5.3 Oxidative Artefact Filtration*

We applied OxoG[40] to identify and remove consensus variant calls that were likely false positives caused by oxidative DNA damage.

### *2.5.4 Strand Bias Filtration*

The strand bias filter flags SNVs that are likely false positives resulting from different artefact-causing processes. Sample-wide PCR template strand bias and sequencing strand bias (i.e. bias between forward and reverse sequencing reads) is identified for each of the 96 SNV categories defined by the type of base exchange and the context of the flanking bases. SNVs are considered as biased if they are from a biased category and have no sufficient support from the opposite strand. The software and a detailed description of the method is available at[51]. In the consensus SNV calls, flagged SNVs were filtered if in one sample more than 3% of the SNVs and more than 30 SNVs were flagged.

### *2.5.5 Review and curation of consensus variant calls*

Following annotation, merging and flagging, the SNV, indel, SV and SCNA consensus call sets were subjected to intensive examination by multiple groups in order to identify anomalies and artefacts, including uneven coverage of the genome, strand and orientation bias, contamination with reads from non-human species, contamination of the library with DNA from an unrelated donor, and high rates of common germline polymorphisms among the somatic variant calls. In keeping with our mission to provide a high-quality and uniformly annotated data set, we developed a series of filters to annotate and/or remove these artefacts. Tumour variant call sets that were deemed too problematic to use for downstream analysis were placed on an "exclusion list" (353 specimens, 176 donors). In addition, we established a "grey list" (150 specimens, 75 donors), of call sets that had failed some tests but not others and could be used, with caution, for certain types of downstream analysis.

#### 2.5.5.1 Sample Exclusion Criteria

After generating consensus calls, cases were excluded from further analysis if they had incomplete or inconsistent metadata, or if the data failed too many basic quality control measures. Some remaining samples were excluded for the following reasons:

High levels of cross-contamination with other subjects as inferred by MLE calculations based on SNV allele fractions using the tool ContEst[39].

Evidence of contamination of tumour in the normal sample (Tumour-In-Normal or TiN contamination; see *Tumour in Normal* below) by the presence of normal reads that support somatic mutations found in the tumour, or by a shift in the ratio of allele frequencies at heterozygous SNPs in regions of somatic copy number variation. Samples with TiN scores higher than 15% were excluded.

A number of samples initially seemed to have extraordinarily high rates of structural variation, in particular deletions in either or both of the tumour or normal. Upon closer inspection, these deletions were introns, and the samples were inferred to have been contaminated with RNA. These samples were removed.

#### 2.5.5.2 Variant Exclusion Criteria

Somatic variants called in the remaining 2,778 whole cancer genomes totalled 45.7 million passing and 5.4 million failing SNVs, and 2.5 million passing and 3.8 million failing indels. In addition to these variants, others were further filtered for particular artefacts seen by downstream analysis tools:

SNVs were run through the Broad's realignment filter[52], where variants supported primarily by BWA aligned reads that were unambiguously mapped to a different location by BLAT were omitted; this removed another 1.8 million SNVs, many of which were extremely high concordance;

Variants were then filtered out by a panel of normals based on 2,450 PCAWG samples developed and maintained by Broad. This step removed an additional 1.3 million SNVs and 683,000 indels;

Samples showing noticeable oxidative damage in reads by OxoQ metric[40] were subject to Orientation Bias filter (OxoG) to remove false positive calls generated by oxidative process during library construction;

A sequencing and PCR bias filter of SNV calls based on abnormal or suspicious distribution of forward/reverse read counts in the 96 possible mutations including triplet context removed 112 thousand SNVs;

Calls overlapping common variants (>1%) in the 1000 Genomes data set were removed, eliminating 1344 SNVs (description under Broad pipeline)

Y-chromosome variants in donors known to be female (368 SNVs and 134 indels) were removed; and

SNVs that overlapped a germline call in the same patient were removed

SNVs that overlapped with germline indels made in the normal were removed, which eliminated a final 328 SNVs.

In addition, calls that were less clearly due to artefacts but were worth flagging were annotated:

High contributions from “artefact” signatures R1, R2, and N3[53].

SNVs near indels: There is an enrichment of apparently false somatic snv calls near both somatic and germline indels because of challenges in aligning indel containing reads. Somatic snvs were therefore flagged if near a somatic or germline indel (position -10 to +25) called in the same sample.

### 2.6 miniBAM generation

The last step prior to distribution of somatic variants to downstream analytic groups was to prepare miniBAM files for each tumour/normal pair. The miniBAM file format is a reduced representation of the aligned BAM file in which reads that provide the evidence for the presence of a somatic or germline variant are retained and the remainder of the reads are discarded. This generates a dramatic reduction in file size, while retaining the ability to visually review called variants. We generated one “miniBAM” file per specimen pair using the VariantBam algorithm[54]. The genomic windows we chose for miniBAM generation were +/- 10 base pairs (bp) for SNVs, +/- 200 bp for indels, and +/- 500 bp for SV breakpoints. This reduced the size of each aligned BAM file to approximately 0.5% of its original, while retaining the reads needed to visually inspect and confirm each variant call. miniBAMs were generated using the coordinates of all raw variant calls generated from the core and supplementary callers prior to the filtering and merging process, allowing both consensus and suspect variants to be reviewed.

## 3. Germline Variant Identification from WGS

### 3.1 Data Overview and Call-set Generation

The PCAWG germline working group constructed a WGS-based germline variant call-set from non-cancerous samples of 2,642 patients affected by 39 different cancer types using the approach presented below. The non-cancerous samples in the PCAWG resource are mostly blood samples (>75%), though some fewer cases are based on tissue adjacent to the primary tumour or other sites of normal tissue such as bone marrow, lymph node or skin. The average germline sequencing coverage was 39x, which is presumed to be adequate for germline variant calling (Bentley et al., 2008). Several algorithms were used for SNP, indel and SV calling following genotyping, call-set integration and haplotype-phasing. Somatic variant calls (including somatic SNVs, indels, and structural variants) and normalized gene expression measurements (available for a subset of donors; *N*=1,172) generated by transcriptome sequencing were obtained from the PCAWG technical and transcriptome working groups. In cases were more multiple samples of primary, metastatic and/or recurrent tumour were

available from a donor, germline-somatic analyses were pursued using the primary tumour sample.

### *3.1.1 Broad germline SNP and indel call-set generation*

The Genome Analysis Tool Kit (GATK) HaplotypeCaller version 3.3.0 was used to analyse 2,818 samples on the Broad Institute's Firehose computing framework, by employing the current GATK best practices recommendations[24]. SNPs and indels identified in individual samples were jointly genotyped across all 2,818 samples using the GATK CombineGVCFs and GenotypeGVCFs modules on a Cray Urika-GX System equipped with 25 compute nodes (each with 32 cores, 256GB RAM) and an 800GB SSD with 2TB of hard disk and 120TB of additional Lustre distributed file system storage.

Variant filtering was performed using the GATK Variant Quality Score Recalibration (VQSR) workflow to identify calling artefacts. The VQSR approach trains a Gaussian mixture model based on supplied training data composed of known, validated ("true") variants as well as known artefacts. The model is trained over a number of variant quality metrics to determine the multi-dimensional quality metric profile of a true germline variant. The model is then applied to all variants in the call set, which are scored with the log-odds ratio of the probability of being a true variant (VQSLOD). Various sensitivity tranches (concentric dashed ellipses) are calculated based on VQSLOD scores such that a pre-specified percentage of known, "true" variants fall within each tranche. Novel variants that fall within multiple sensitivity thresholds are annotated with the most conservative, or restrictive, sensitivity tranche. Tranches that include higher percentages of known variants (outermost ellipses) have more permissive VQSLOD threshold values and are more likely to contain false positive variant calls than tranches with more conservative VQSLOD threshold values (inner ellipses). The sensitivity thresholds applied to the Broad call-set to classify "PASS" quality variants were 99.6 for SNPs and 95.0 for Indels (though the tranche annotations allow researchers to calibrate a custom sensitivity level to suit different analysis needs). The "gold standard" training sets used for the VQSR workflow were the Mills set of variants for indels[55] and the HapMap3[56] and Illumina Omni 2.5M SNP array set of variants for SNPs. Genotype calls were filtered for quality by removing any calls with a genotype quality score (the phred-scaled probability of an incorrect alternate allele call) less than 20.

Sample-level quality control metrics examined included the per-sample call rate (the percentage of all possible variant sites with a called genotype in a given sample), mean sequencing read depth, mean genotype quality score, ratio of transitions to transversions called, and the ratio of heterozygous to homozygous alternate genotypes called. Samples featuring at least one outlying metric (*i.e.*, with values greater or less than 4 standard deviations from the mean value calculated over all samples of the same ethnicity) were flagged as potential outliers. Sample ethnicities were imputed based on the first 8 principal components obtained over ~115,000 linkage disequilibrium-pruned SNPs across the genome with $r^2$>.1 and a minor allele frequency (MAF) > 0.01.

Duplicate samples and cryptically related samples in the cohort were identified by running KING[57] on the same set of ~115,000 pruned SNPs used in the principal components analysis. The 2,818 samples analysed using GATK included 176 that were later black-listed by the PCAWG project (their removal resulted in the set of 2,642 samples that were ultimately used by the PCAWG germline group).

### *3.1.2 Freebayes germline SNP and InDel call-set*

We employed freebayes (doi:arXiv:1207.3907) (v0.9.21-26-gbfd9832) in single-sample calling mode for SNP and indel discovery (--min-repeat-entropy 1, --report-genotype-likelihood-max). Raw calls were filtered for quality (QUAL>20, QUAL/AO>2), strand bias artefacts (SAF>1, SAR>1), and read position artefacts (RPR>1, RPL>1), and normalized for consistent representation with vt normalize[58] (v0.5). Freebayes short sequence variant calling was pursued on the EMBL-EBI Embassy Cloud (www.embassycloud.org), using the following setup: 1500 cores, 50 TB SSD storage, 4 TB of RAM. Variant calling with freebayes on the cloud was orchestrated using an early release version of the Butler cloud workflow orchestration framework[59] (https://github.com/llevar/butler, revision fa28b5c).

### *3.1.3 Short variant calling with the Real Time Genomics (RTG) software*

We called germline variants from the project BAMs using the single-sample variant caller from Real Time Genomics (RTG) version rtg-core 3.6.2 (Real Time Genomics Ltd, Hamilton, New Zealand; https://github.com/RealTimeGenomics). RTG snp uses a Bayesian network model for variant calling, and a haplotype-aware Bayesian method for making "complex" calls[60] (indels, MNPs, and combinations thereof). First, base-qualities from reads in the BAM files were recalibrated with RTG calibrate to generate calibration files needed by RTG snp. To maximise parallelization during calling, the BAMs were split by chromosome and the final call-set for each sample was obtained by merging the final VCFs from all jobs. Variant calls were scored using the RTG Adaptive Variant Rescoring method[60] (AVR), which uses a random forest algorithm model to score variants with respect to their probability to being correct. The model was trained with WGS calls from the CEPH and YRB trios of the 1000 Genomes Project. Variant calls where filtered with an empirically defined AVR cutoff of 0.1 to obtain a variant set with good quality metrics. We used two compute environments to run the calling pipeline. We analysed the first 1,300 samples in the Annai BioCompute Farm (Annai Systems Inc., Carlsbad Ca, USA), which is a OpenStack based virtualized cluster environment hosted at the UC San Diego Super Computer Center. The remaining samples were analyzed using a DNAnexus platform (DNAnexus Inc., Mountian View, CA, USA), which uses as backend the Amazon AWS cloud infrastructure, using an DNAnexus applet that implements the identical variant calling pipeline[61].

### *3.1.4 Delly germline deletions*

Delly[29] v.0.6.3 was applied to each pair of tumour and matched control to jointly call germline and somatic deletions >500bp. Candidate germline deletions were merged across samples into a unified deletion site list using a strict reciprocal overlap of 90% and a breakpoint offset <=100bp. The deletion sites were subsequently genotyped in parallel using Delly v0.7.3 at the EMBL-EBI Embassy Cloud across all control genomes and then merged using BCFtools. Uncertain genotypes with genotype quality below 20 were set to missing. Deletions with an overall genotype missing rate >15% were removed as well as deletions that lacked at least one carrier sample with a variant allele support >=20% to account for potential tumour-in-normal contaminations at lower level. Among clusters of overlapping deletions that likely arose due to breakpoint inaccuracies in single-sample SV calling, we selected the best deletion in terms of overall genotype quality. To ensure high specificity we further filtered the remaining SVs using a machine learning approach that used as a training set genotyped deletions in the 1000 Genomes Project phase 3 low coverage data. We evaluated the array

concordance of these deletions using Intensity Rank Sum testing (IRS) and differentiated likely true SVs (p-value < 0.5) and likely false SVs (p-value >= 0.5). The most predictive feature in the random forest model was the read-depth ratio of SV carriers compared to SV non-carriers, which is well in line with the nature of the Delly algorithm, which solely relies on paired-ends and split-reads but annotates read-depth to enable a subsequent read-depth based filtering of germline deletions. Machine learning parameters were picked to derive a final deletion site list of an estimated FDR of 5% using the PCAWG validation SNP6 array dataset. Overall, Delly ascertained 29,492 deletions (27,577 copy-number variable regions) in the set of 2,642 PCAWG control samples.

### *3.1.5 Mobile element insertion call set*

Pre-aligned BAM files from 2,834 PCAWG tumour and matched normal pairs were processed with TraFiC-mem v1.1.0 (https://gitlab.com/mobilegenomes/TraFiC) to jointly call non-reference germline and somatic MEIs, including Alu, L1, SVA and ERV-K insertions. Briefly, the algorithm relies on the identification of discordant read-pairs from Illumina paired-end sequencing data, followed by clipped-read analysis to characterize the insertion breakpoints at base pair resolution. A complete description of TraFiC-mem method is provided in the on-line methods section of the somatic retrotransposition manuscript[62]. In order to guarantee a high specificity, germline MEIs were required to have both their 5' and 3' insertion breakpoints characterized and be supported by at least 4 clipped-reads to be considered for further analyses. Then, candidate germline MEIs identified along all non-cancerous samples were clustered using a breakpoint offset of ≤50bp and the MEI supported by the highest number of reads (discordant plus clipped reads) in each cluster was selected as representative to generate a non-redundant MEI dataset. This dataset comprises 27,546 candidate germline MEI, including 22,207 Alu, 4,366 L1, 945 SVA and 28 ERV-K insertions.

The unified MEI site list was re-genotyped in all the normal genomes using TraFiC-genotyper v1.1.0 (https://gitlab.com/mobilegenomes/TraFiC-genotyper). To genotype each MEI, the algorithm inspected the read alignments around the predicted insertion breakpoints searching for reads supporting the reference (i.e. MEI absence) and alternative (i.e. MEI presence) alleles. Properly aligned reads spanning the breakpoint with a minimum overhang of 20 bp support the reference allele (REF-reads), while clipped-reads with clipping positions at a maximum distance of 3 bp to the predicted breakpoint support the alternative allele (ALT-reads). Reads marked as duplicates and reads clipped both at their beginning and ending extremes are removed, as they usually constitute mapping artefacts. Then, MEI allelic fraction (AF) is computed as the ratio of ALT-reads to TOTAL-reads (i.e. ALT-reads plus REF-reads). A heterozygous genotype call is made for MEI with AF between 0.1-0.9, a homozygous call for AF higher or equal than 0.9 and the genotype is set to 'missing' if the AF is lower than 0.1. Genotypes supported by less than 4 ALT-reads or REF-reads are also set to 'missing'. Finally, a single multi-sample VCF v4.2 file, containing germline MEIs genotypes for the complete set of normal samples, is produced as output.

In order to prevent sample-specific genotyping errors due to the accumulation of artefactual split-read alignments around the insertion breakpoints, heterozygous and homozygous alternative genotypes were set to 'missing' if they were supported by at least 5-fold more split-reads than the median among all the analyzed samples. Finally, MEIs with an allele count of 0 or an overall genotype missingness rate >5% were filtered out.

The released dataset is composed by 27,254 germline MEI events. Consistently with 1000 Genomes catalogue of germline variation[63], Alu and L1, with 97% (26,302/27,254) of the events, are the most abundant retrotransposon lineages in the PCAWG germline resource. On the other hand, SVA and ERV-K polymorphisms, with 927 and 25 instances, represent minor categories. Most MEIs (84%; 22,979/27,254) represent rare alleles (MAF < 1%), while 10% are common variants (MAF > 5%). To determine which MEIs were not previously reported, we compared our call set to 1KGP phase 3 and GoNL[63,64] v6.1 SV releases. Those MEIs whose insertion breakpoint was at a maximum distance 100 bp of an already known element from the same family were catalogued as known; or novel, otherwise. A large proportion of loci (58%; 15,681/27,254) constitute novel genetic variation not previously reported. Novel germline MEIs are particularly enriched in rare polymorphism although a substantial fraction of them (11%, 2,631/24,198) correspond to common variation. On a population level, we found that each donor bears ~1,300 polymorphic MEIs on average (**Extended Figure 12F**). This finding substantially differs from recent estimations of ~1,200 MEIs per donor[63] based on WGS at a coverage of ~8x, as compared to the ~39x WGS coverage of PCAWG matched normal samples. As previously reported for SNPs[65] and deletions[63], we observed that individuals of African ancestry exhibit higher MEI loads than individuals from other populations.

Finally, for germline MEIs already reported by 1000 Genomes Project, we evaluated the consistency between PCAWG and 1000 Genomes Project inferred allele frequencies overall and across each ethnicity. Correlations were over 0.7 for each retrotransposon subfamily – even for Admixed Americans and South Asians, two ethnics groups composed by only 29 and 39 samples, respectively (**Extended Figure 12G,H**).

### *3.1.6 Orthogonal validation of germline MEI with single-molecule sequencing*

In order to evaluate our germline MEI resource, a liver hepatocarcinoma specimen (SP112196) and the corresponding matched normal (SP112195) were selected from a PCAWG donor (DO50807) to be sequenced by single-molecule sequencing using Oxford Nanopore Technologies (ONT). Whole-genome libraries were constructed with the ONT 1D ligation library prep kit (SQK-LSK109), which includes steps to repair (NEBNext FFPE DNA Repair, NEB), end-repair and dA-tailing the DNA (NEBNext End Repair/dA-tailing module, NEB). We obtained 2 and 4 libraries for SP112196 and SP112195, respectively. Genomic libraries were loaded on MinION R9.4 flowcells (FLO-MIN106 rev D), and sequencing runs were controlled using the software MinKNOW v18.07.18 and v18.12.5. We used the basecallers Albacore v2.3.3 and Guppy v2.1.3 to identify DNA sequences directly from raw data and generate FASTQ files. Files with quality score values below 7 were excluded at this point. Minion adapter sequences were trimmed using Porechop v0.2.3 (https://github.com/rrwick/Porechop) and the internal guppy trimming. Then, for each sequencing run we used minimap2 v2.10-r761 to map sequencing reads onto the hs37d5 human reference genome, and the SAM files were converted to BAM format, sorted and indexed with Samtools v1.7. BAM files derived from the same sample were merged, sorted and indexed. After this process, sequencing coverage were 10x (SP112196) and 8x (SP112195), and the average read size of mapped reads were 5.6 Kb (SP112196) and 14 Kb (SP112195). We performed validation of 1,243 germline MEIs genotyped as heterozygous or alternative homozygous in the liver hepatocarcinoma donor (DO50807) sequenced using ONT. In order to maximize the coverage, we pooled the long-reads derived from the tumour

and matched normal sample under the assumption that most germline variation will be captured in both datasets. The resulting BAM file represents 18x coverage on average. Then, we applied the same approach we used for the assessment of somatic MEIs[62]. Briefly, we sought for two types of reads supporting each germline MEI in the BAM file: (i) reads completely spanning the insertion and that are identified as standard insertions on the reference; and (ii) reads spanning only one of the inserted element extremes, so they get clipped during the alignment in the reference. Germline MEIs supported by at least two reads were considered true positive (TP) events or false positives (FP), otherwise. Overall, we observed 6% (78/1,243) false positive events while when we stratified MEI by frequency, the FDR was 7%, 3% and 0% for common, low frequency and rare variants, respectively (**Extended Figure 12I**). False discovery rate was estimated as follows: FDR = FP / (TP + FP). We further evaluated the consistency between the predicted MEI lengths based on Illumina and Nanopore. Inferred lengths strongly correlate between both sequencing technologies for Alu (Spearman's $\rho = 0.49$, $P = 7.66e^{-61}$) and L1 elements (Spearman's $\rho = 0.94$, $P = 4.94e^{-50}$), while SVA lengths are frequently underestimated in Illumina calls (**Extended Figure 12J**). This underestimation of SVA lengths in Illumina data can be explained due to the variability of SVA sequences at their GC-rich tandem repeats (VNTR) central region[66], which cannot be resolved through short-read data analysis. In order to validate additional germline MEIs, we reused the dataset generated for evaluating TraFiC-mem somatic calls[62]. This dataset is composed by one cancer cell-line (NCI-H2087) and its matched normal cell-line (NCI-BL2087) sequenced both with Illumina and ONT. We re-genotyped our germline MEI call-set in the matched normal Illumina sample and each heterozygous and alternative homozygous MEI was subjected to long-read validation as described for the hepatocarcinoma donor. Consistently with this data, we observed a FDR of 7% (79/1,119) and a strong correlation for Alu and L1 inferred MEI lengths. Overall, through the analysis of two long-read datasets (i.e. PCAWG hepatocarcinoma donor and cell-line) we attempted validation for 1,789 distinct variants from our germline MEI variation resource with an FDR of 6% (103/1,789).

### *3.1.7 Germline L1 source element analysis*

BAM files from tumour and matched normal pairs were processed with TraFiC-mem v1.1.0 (https://gitlab.com/mobilegenomes/TraFiC) to identify somatic L1-mediated transductions, among other types of retrotransposition events, as described by the PCAWG Structural Variation Working Group[62]. L1-mediated transductions are small tracks of L1-adjacent unique DNA sequences mobilised through L1 retrotransposition that can be used as barcodes to trace somatic L1 insertions to individual source L1 loci[67]. L1-transduction calls were matched with our germline L1 resource to compile a dataset of candidate germline L1s mediating transductions in multiple tumour samples or more than one transduction in a single sample. Candidate L1s were subjected to manual curation and breakpoint annotation through visual inspection of BAM files with IGV the Integrative Genomics Viewer[68] (IGV). This analysis revealed 114 germline L1 loci with detectable somatic transduction activity, including 70 that represent insertions with respect to the human reference genome. L1 source elements were then genotyped in all the matched normal genomes using the same genotyping approach and filters described for germline MEIs, prior to their integration into the phased PCAWG germline variant release. 22 out of 44 elements in the reference genome appeared to be fixed within the PCAWG cohort. To further support this observation, we searched for discordant read-pair clusters pointing to the deletion of the elements amongst all the normal genomes; no deletion clusters were identified, what is consistent with the fixation of these loci in PCAWG donors.

To identify hot-L1 source elements two metrics were computed for each L1 source locus. First, the percentage of samples, with at least one retrotransposition event, where the element is active. Second, the activity rate, measured as the average number of transductions mediated by the element in those samples where it is active. Then, source elements were clustered according to these two parameters using scikit-learn[69] v0.18.1 DBSCAN implementation (eps=0.8, min_samples=5). This analysis revealed a well-defined cluster composed by L1s without hot activity and two clearly differentiated groups of outliers, corresponding to Plinian and Strombolian hot loci, respectively. Outlier elements were catalogued as Plinian if they exhibited activity rates over 5 and were active in less than 2% of the samples and Strombolian, otherwise.

#### *3.1.8 Short germline variant consensus call-set*

Germline SNPs and indels were discovered using Freebayes, the Genome Analysis Tool Kit (GATK) HaplotypeCaller and the Real Time Genomics (RTG) variant caller, as described above. The FreeBayes and RTG call sets were 'by sample' whereas the GATK call set was genotyped across all 2,642 samples for a unified VSQR filtered site list. We did not perform additional genotyping to obtain genotype likelihoods of Freebayes and RTG-based variant calls across all 2,642 samples. Because of our reference-panel based statistical phasing strategy, we first kept all GATK sites that are present in the 1000 Genomes Project phase 3 haplotype reference panel. All remaining bi-allelic and multi-allelic SNPs and indels out of the FreeBayes, RTG and GATK call sets were uniformly normalized to facilitate a variant site intersection. We decomposed all multi-allelic variants into bi-allelic variants, left-aligned all variants using vt[58] and then kept all GATK sites that are shared with at least one other caller (FreeBayes or RTG). For multi-allelic GATK sites, all decomposed bi-allelic variants needed to be confirmed by one additional caller. The consensus call set was then further subsetted to samples deemed of sufficient quality for inclusion (#n=2,642) and we set all genotypes with genotype quality <20 to missing. We subsequently dropped all sites with an updated allele count of zero or with less than 75% of the samples genotyped.

## 3.2 Germline short variant validation

Deep validation sequencing was performed on 50 of the original PCAWG pilot 63 tumour/normal pairs representing 24 cancer types. Originally 5,000 sites were selected by the PCAWG Germline Working group based on consensus variant calling. NimbleGen capture reagents were capable of capturing 3,112 (65%) of these sites following exclusion of repetitive sequence and poor nucleotide context (i.e. high GC-content). These sites were sequenced in every tumour/normal pair of the cohort. 38 of the 50 samples were sequenced at Washington University in St. Louis, while the remaining 12 were sequenced in at the Wellcome Trust Sanger Institute following international protocols and regulations. A median sequencing depth of 512 reads in the control sample, 610 reads in the tumour sample, was identified across all variants tested. 0.5% of the targeted germline variants did not have sufficient coverage (fewer than 20 reads per site) and were hence excluded.

### *3.2.1 Short variant call validation experiments*

We assessed the performance of the three pipelines for inference of germline variants from WGS data used in this study (GATK HaplotypeCaller[70], RTG (https://www.realtimegenomics.com), Freebayes[15] and the PCAWG consensus-phased

germline call-set using resequencing data for randomly picked candidate germline short variant sites. We identified the genotypes of the tested sites based on read counts in the ultra-deep sequencing data using a custom script. Several filters were applied to remove false positives (see below under “methodological details”). We then employed calls based on ultra-deep resequencing as a gold standard to benchmark germline short variant call-set quality. We separately evaluated SNP and indel calls using precision, recall, false discovery rate (FDR), F-score and genotype concordance as measures.

All benchmarked pipelines showed excellent performance in SNP calling (**Supplementary Figure 12**), with precision estimates >99%, and recall estimates ranging from 96.5%-98.8% (F-scores ranged from 0.980 to 0.992). All pipelines also showed high genotype concordance prior to phasing (with the maximum of 0.994 observed for the HaplotypeCaller) when considering homozygous reference, heterozygous and homozygous alternative calls in the analysis. When taking into account only alternative sites (ignoring homozygous reference), concordance was similarly high with a maximum of 0.995 achieved by RTG (**Supplementary Figure 12)**. In the consensus-phased call-set, the precision was 0.995 and the recall 0.980. Performance measures for rare (AF <= 5%), mid-common (5% < MAF >= 20%) and common variants (AF > 20%) revealed similar precision estimates for rare variants in all call-sets (**Supplementary Figure 12)**. Precision of indel calls of Freebayes and RTG was similarly high as for SNPs (ranging from 0.996 and 0.993), at the cost of lower recall (~91-92%) (similar to earlier studies[71]). HaplotypeCaller achieved a lower recall and precision (88.1 and 98.2 %, respectively), mostly due to a reduced performance for rare indels (**Supplementary Figure 12)** The consensus-phased calls reached values of 0.924 and 0.995 for recall and precision respectively. Indel genotype concordance ranged from 0.955 to 0.971 when taking into account homozygous reference sites, but was slightly reduced to 0.917-0.931 when only considering variant sites (**Supplementary Figure 12**) with the consensus-phased call-set showing the best concordance. Additionally, the allele frequency of variants in the tested population was correlated with the recall of the calls (**Supplementary Figure 12**).

*Methodological details***.** Ultra-deep sequencing reads were aligned using BWA-MEM 0.7.8-r455. We identified the genotypes of tested sites based on read count data, using a custom script. Several filters were applied to remove false positives, including a minimum coverage filter (depth [DP] > 15) and the exclusion of sites exhibiting strand bias (> 90 % in a specific strand) and sites with more than 2 alternative alleles in the same sample. Next, we used these calls as gold standard to benchmark the three variant analysis tools, as well as the PCAWG germline consensus sites list, achieved in ~39x WGS data from the PCAWG network. We separately evaluated SNP and indel (left aligned with *bcftools*) calls using precision, recall (sensitivity), false discovery rate (FDR), F-score and genotype concordances as measures. Only those variants that passed the specific filters of each caller were considered.

Note that for calculating precision, calls were considered as true positives when the pipeline found the correct alternative allele, irrespective of the called genotype (heterozygous or homozygous), while genotype concordance took into account the predicted genotype. In the concordance analysis, two types of concordance were obtained: one considering all genotypes and one ignoring homozygous reference genotypes. In the GATK HC results, “./.” genotypes were considered as homozygous reference (0/0).

- TP ~ True positive
- FP ~ False positive
- FN ~ False negative
- TN ~ True negative
- Precision = TP/(TP+FP)
- Recall (sensitivity) = TP/(TP+FN)
- FDR (False discovery rate) = 1 – precision
- F-score (F1) = 2TP/(2TP+FP+FN)

As another means of quality control, we performed Principal Component Analysis (PCA) jointly for PCAWG samples and 1000GP phase 3 samples using ancestry informative marker (AIM) SNPs. Reassuringly, PCAWG donors fell inside the diversity of their 1000GP counterparts. We also estimated the proportion of archaic ancestry in PCAWG samples using the ADMIXTOOLS software combining PCAWG samples with samples from the Simons Genome Diversity Project (SGDP)[72] following the methods recently described by Fu and colleagues[73]. We compared each PCAWG sample against 9 SGDP African samples, 3 Dinka samples, the Archaic genomes of one Neanderthal and one Denisovan individuals and the Chimpanzee reference genome (PanTro2). We also used 8 European and 7 East Asian samples from SGDP as controls. Using this dataset, we calculated the d-statistic for each PCAWG sample. We removed from the dataset all samples with over 50% inferred African ancestry and all samples with a d-statistic's Z-score lower than 3. We analyzed archaic admixture for the remaining 2670 samples using the f4-ratio statistic. The results by population show estimates within the diversity found by previous studies of 1.2-1.5% admixture for European samples and 1.4-1.8% for Asian samples.

## 3.3 Whole-genome low coverage structural variant validation using long-reads

Genomic DNA of a prostate cancer patient carrying a BRCA1 germline mutation was sequenced using the Oxford Nanopore Technologies (ONT) GridION device. The fast5 sequencing files were basecalled using Guppy and then aligned using minimap2[74] v2.11. Resulting BAM alignment files were sorted and indexed using SAMtools[25]. Alfred[75] was used for quality control and to estimate the mean sequencing coverage 1.6x (median coverage = 1) which was very low due to limited gDNA availability and low input DNA quality. The mean sequencing error rate was 11.9% at a median read length of 1596bp. 77% of the genome was covered >=1x. Because of the high error rate of long read sequencing we assumed that we will require at least a ~200bp prefix and suffix alignments across the SV junction to confidently validate an Illumina SV breakpoint, which lowered the effective coverage for SV junction detection to 1.3x.

Using the Lander and Waterman model [76] and an effective sequencing coverage of 1.3x (haploid coverage 0.65x) the percentage of SV breakpoints on a given haplotype that are covered by at least two reads is expected to be 14%, and 48% will be covered by at least one

read. Because of the low sequencing coverage, we devised an algorithm that collects all primary, secondary, and supplementary alignments for a given long read, sorts the internal breakpoints by sequence coordinate and then cross-matches these long read junctions to the Illumina SV call-set using a strict 80% reciprocal overlap criterion for intra-chromosomal SVs and a maximum breakpoint offset <1000bp for inter-chromosomal SVs. Using this approach, we could validate overall 36.8% of all Illumina SV breakpoints in the prostate cancer patient, with 34.1% for deletion-type SVs, 39.2% for duplication-type SVs, 32.5% for inversion-type SVs and 30.7% for inter-chromosomal SVs. These results suggest high accuracy (specificity) of the PCAWG somatic SV call-set for the selected prostate cancer patient, corroborated by long reads. We could not evaluate the sensitivity of the Illumina call-set because of insufficient long read coverage.

We next attempted to locally assemble long reads that jointly span the same SV breakpoint to elucidate SV breakpoint characteristics and to identify template insertions between adjacent SV breakpoints. For all SV breakpoints with more than one supporting long read we used a multiple-sequence alignment-based algorithm[75] to compute a consensus sequence of greater length and with slightly improved sequencing error rate (10.4%). These consensus sequences were then aligned back to the respective reference segments using Maze (https://gear.embl.de/maze) and a custom MUMmer pipeline[77]. Two examples of a long-read consensus alignment are shown in **Supplementary Figure 9** and **Supplementary Figure 13**.

## 3.4 Haplotype-block phasing of germline variants using 1000GP as a haplotype reference panel

Germline variant phasing of PCAWG normal samples was initiated with Eagle2[78] to phase bi-allelic SNPs and indels using the 1000 Genomes Project phase 3 dataset[63] as a haplotype reference panel. This haplotype scaffold included all bi-allelic variants shared between PCAWG and 1000GP. All remaining bi-allelic variants not present in 1000GP were subsequently phased using ShapeIt2[79] and added onto the input Eagle2 haplotype scaffold. The ShapeIt2 phasing was conducted in 2Mbp windows using 200Kbp buffer regions on either side of the window boundary; different chunks were concatenated into a combined Eagle2 and ShapeIt2 bi-allelic haplotype scaffold using BCFtools[80]. ShapeIt2 was run using default parameters except for "-S 800 –W 0.2 and --buffer 200000". Multi-allelic short variants with at least 2 alternative alleles were phased onto this combined scaffold using MVNcall[81] with the following non-default options: "--var-multi --k 100 --iteration 50". Conversion into MVNcall haplotype format was conducted using custom shell and python scripts, and BCFtools and HTSlib was used to merge and concatenate MVNcall output files. Germline deletions and mobile elements were phased depending on the availability of genotype likelihoods, which are required by MVNcall. Because of that, bi-allelic deletions were phased using MVNcall, while mobile element insertion calls (which did not have genotype likelihoods) were phased using ShapeIt2.

## 3.5 Inference of continental-scale ancestry and genome-wide local ancestry deconvolution

We estimated the proportion of genome-wide continental-scale ancestry of donors, by implemented a supervised version of the ADMIXTURE algorithm[82]. First, 4235 ancestry informative SNP markers (AIM) were chosen for maximum discriminative power between 5 continental super populations (European, East Asian, Africa, Native American, South Asian) as described in[83]. Minor allele frequencies were calculated for the 5 continental populations at these AIMs using 1000 Genomes Phase 3 data[71] from unrelated subjects as previously described[83]. For each sample, missing data at any AIM location was filled in with homozygous reference calls. We optimized the log-likelihood for the ADMIXTURE model assuming K=5 and the allele frequencies to be known from above. Individual ancestry likelihoods for each of the five continental super populations were estimated per donor using the LBFGS optimization algorithm, implemented in the R software (R Core Team 2014). Scripts, ancillary files, and ancestry proportion estimations are available at the Sage Bionetworks Synapse platform (Seattle, WA, USA) under syn4877977. We validated the method by analyzing the samples from the 1000 Genomes, assigning each individual to a superpopulation based on the highest estimated superpopulation likelihood (or the two highest in case of admixed populations), and comparing the results with the of the labels of the 1000 Genomes project samples. We also performed spot checking by comparing our results against the clusters obtaining by performing PCA with Plink[84], using a subset of 700k SNPs across the genome chosen with r-squared < 0.2 in 1 MB chunks and with a minor allele frequency of >= 1%. PCA plots where visualized using the Genesis software (http://www.bioinf.wits.ac.za/software/genesis/). Results are available at the Sage Bionetworks Synapse platform (Seattle, WA, USA) under syn4874212. Genome-wide local ancestry was estimated using RFMix[85] assuming five ancestral backgrounds: African, European, Native American, East Asian, and South Asian, as described in reference [86]. Genome-wide local ancestry estimations are available at the Sage Bionetworks Synapse platform (Seattle, WA, USA) under syn18412166.

Using inferred ancestry estimates, we assessed ancestry components to the somatic mutational burden. This analysis was possible for two tumour types: Liver-HCC (43 EUR *vs* 264 ASN) and Stomach-AdenoCA (24 EUR *vs* 41 ASN). We assessed these samples for differences in mutational burden by ancestry and identified that the somatic C>A ($\beta_{EUR}$=+2.1%, $P$=3.7e-3) and T>C ($\beta_{EUR}$=-4.7%, $P$=2.5e-4) mutation rate in liver cancer was significantly associated with ancestry (FDR<10%) after accounting for demographic (*ie*, sex and age at diagnosis) and technical factors (*ie*, tumour purity, tumour & normal sequencing coverage, and tumour & normal sequencing coverage skewness). Either genetic (East Asian *vs* European) or environmental (Japan *vs* USA *vs* France) factors may contribute to this differential mutational burden given that the Liver cancer specimens that contributed to this analysis were collected in different countries (5x France, 48x USA, 254x Japan).

## 3.6 Identification of protein-truncating variants (PTVs)

High impact (*i.e.* pathogenic) germline variants were defined as frameshift, nonsense, and canonical splice site variants. Putative pathogenic germline PTVs were removed if the estimated minor allele frequency (MAF) in at least one continental population (EUR, AFR, ASN, SAN, AMR) was above 0.5% based on information from 53,105 sequenced individuals

that were assigned to known populations and without a cancer diagnosis from ExAC[87], the 1000 Genomes Project[71], and the NHLBI GO Exome Sequencing Project[88]. We filtered out germline PTVs that were common in PCAWG (MAF>5%). Finally, all candidate germline PTVs were excluded from the analysis if annotated as benign in ClinVar (accessed 02/06/16). Germline deletion SVs called by Delly that overlapped at least one candidate exon and were absent in subjects from the 1000 Genomes Project phase 3 SV set were defined as high-confidence pathogenic (*i.e.* inferred to lead to PTV).

## 3.7 Rare variant germline-somatic variant association analysis

The association between germline PTVs in genes and somatic mutational phenotypes was modeled using linear regression (*lm* function, R package). The model accounted for sex, age at diagnosis [quantiles], population structure (five principal components), tumour histology, project, and technical confounders (tumour purity [quantiles]; tumour and normal sequencing coverage [quantiles]; tumour and normal sequencing coverage bias [quantiles]). We limited our tests to genes with at least four germline PTV carriers and performed our analysis in individuals with European ancestry to reduce population-specific effects. We modeled somatic mutation rates by normalising mutation counts against the total number of somatic mutations (*eg*, total number of SNVs, total number of SVs). Tumours with a low mutational burden (<10 SNVs or SVs) were excluded from the analysis. We followed-up genes that passed the exome-wide significance threshold of *P*<0.05/20,000 (=2.5e-6) for further analysis. Somatic CpG mutation rates were estimated using the proportion of signature 1 counts (PCAWG beta2 release) and the proportion of Np[C>T]pG mutation load counts (**Supplementary Methods 3.9**). We validated germline associations with mutational signatures that were derived from 8,134 TCGA WES samples and we tested the hypothesis that variation in gene expression is correlated with mutational signatures. We obtained for the latter gene expression quantifications based on FPKM-UQ values for 1,172 PCAWG donors. We restricted hereby our analysis to primary tumours and solid, non-haematological cancer types with at least ten donors. Cancer-type specific association analysis between rank-normal transformed gene expression levels and somatic mutation rates was based on 951 tumour samples and 20 cancer types. Linear regression models included (if available) sex, age at diagnosis, and ICGC project as putative confounders.

## 3.8 Common variant germline-somatic variant association analyses

We performed genome-wide association analysis for two endogenous mutational processes. We performed the analysis with common SNPs/indels (MAF>5%) that passed quality control (genotyping rate >95%, HWE>1e-5) and restricted the analysis to individuals with European and East Asian ancestry, respectively. We accounted for demographic (sex, age at diagnosis), histological, and technical confounders (tumour purity, tumour/normal coverage, tumour/normal coverage bias, project). Residual mutational phenotypes were rank-normal transformed and the analysis was controlled for population structure using principal components (N=3). The genome-wide association analysis was performed with PLINK[84] v1.9, Manhattan plots were prepared with *qqman* v0.1.4 in *R*, and locus zoom-in plots were prepared with *LocusZoom* (http://locuszoom.org/). Somatic CpG mutation rates were estimated using the proportion of Signature 1 counts (PCAWG beta2 release) and the proportion of Np[C>T]pG mutation load counts (Supplementary Methods 3.9). APOBEC3B-like mutagenesis was estimated using the APOBEC3B enrichment score (Supplementary

Methods 3.9). We considered tumours with a statistically significant contribution of APOBEC mutagenesis for the analysis and followed-up loci that passed the genome-wide significance threshold (*P*<5e-8). Skin melanoma samples were excluded from the APOBEC3B-like mutagenesis GWAS. Non-coding loci were further assessed for *cis*-regulatory activity in donor-matched primary tumour samples based on pan-cancer *cis*-eQTL analysis using rank-normal transformed expression phenotypes and a linear regression model that accounted for sex, age at diagnosis [quantiles], histology, ICGC project, ten principles components.

## 3.9 Knowledge-based analysis of mutational processes

Enrichment and mutation load of a suspected specific mutational process were calculated based on prior mechanistic knowledge about mutation motifs associated with certain mutagenic factors and pathways[89–91]. The enrichment with a tri- or tetra-nucleotide motif *pXq→pZq*, wherein the mutated residue is capitalized, was calculated in each sample as

Enrichment(pXq→pZq) = (Mutations(pXq→pZq) x Context(x)) / (Mutations(X→Z) x Context(pxq))

where *X* is the mutated nucleotide, *Z* is the nucleotide after base substitution, *p* is the -1 nucleotide (or -1 and -2 nucleotides), and *q* is the +1 nucleotide (within the context of the given mutation type/ trinucleotide). For each motif, we also included the reverse complement sequence that would represent the mutagenic process occurring on the opposite DNA strand. The context was derived from the 41 nucleotides surrounding the mutated residue. This approach focuses on the genomic regions wherein the mutation occurred, without excluding any specific genomic areas. It also would not be affected by preference of mutagenesis to certain genomic areas over the others. This methodology usually gives results similar to NMF-based signature analysis in which the whole genome was considered for the context in the calculations. To statistically evaluate whether a certain mutation type is enriched in a sample as compared to mutations generated by random mutagenesis, a one-sided Fisher's exact test was performed to compare the following two ratios:

Ratio 1: Mutations(pXq→pZq) / (Mutations(X→Z) - Mutations(pXq→pZq))

Ratio 2: Context(pxq) / (Context(x) - Context(pxq))

To account for multiple testing, *P*-values obtained were corrected using the Benjamini-Hochberg method. For samples with enrichment > 1 and q-values ≤ 0.05, the minimum estimated mutation loads were calculated as:

MutLoad (pXq→pZq) = (Mutations(pXq→pZq) x (Enrichment(pXq→pZq)-1)) / Enrichment(pXq→pZq)

If the enrichment < 1 or the q-value was ≥ 0.05, the minimum estimated mutation load was assigned a value of "0". Np[C>T]pG mutational signature was studied based on "5' [a|t|g|c]C[g] 3'" motif analysis and APOBEC3B-like mutational signature[90] was studied based on "5' [a|g]tT[a] 3'" and "5' [a|g]tG[a] 3'" motif analysis.

# 4. RNA-Seq Analysis

## 4.1 RNA-seq alignment and quality control

A total of 2,217 RNA-seq libraries were aligned with both STAR[92] (version 2.4.0i, 2-pass), performed at MSKCC and ETH Zürich, and TopHat2[93] (version 2.0.12), performed at the European Bioinformatics Institute. We compared gene quantification using both alignment strategies giving consistent results. Different downstream analysis pipelines required either STAR or TopHat2; therefore, we provided alignments using both methods. The human genome reference used was GRCh37.p13 and GENCODE v19 was used as the transcriptome reference. Code and parameters used for the STAR alignment can be found at https://github.com/akahles/icgc_rnaseq_align and for the TopHat2 alignment at https://hub.docker.com/r/nunofonseca/irap_pcawg/. QC filtering was based on metrics from the raw FASTQ files, metrics from aligned reads, and correlation of gene expression when using either STAR or TopHat2. Technical replicates (722 libraries) were merged giving a final number of 1,359 fully processed RNA-seq sample aliquots from 1,188 donors. The list of sample aliquots and additional metadata can be found at https://dcc.icgc.org/releases/PCAWG/transcriptome/metadata/rnaseq.extended.metadata.aliquot_id.V4.tsv. More details of PCAWG RNA-Seq data processing can be found in PCAWG Transcriptome Core Group[94].

## 4.2 Quantification of gene and transcript-level expression

Gene expression quantification was performed using HT-Seq[95] (version 0.6.1p1) separately on STAR-aligned reads and TopHat2-aligned reads from the same sample and then a consensus expression quantification was performed by taking the average. Gene counts were normalized by adjusting the counts to fragments per kilobase of million mapped (FPKM) as well as fragments per kilobase of million mapped with upper quartile normalization (FPKM-UQ) where the total read counts in the FPKM definition has been replaced by the upper quartile of the read count distribution multiplied by the total number of protein-coding genes. Transcript-level expression quantification was performed using Kallisto[96] (version 0.42.1).

## 4.3 Identification of alternative splicing events

Alternative splicing events were identified and quantified using SplAdder[97] with default parameters and confidence level 3 based on read alignments using STAR. Splicing graphs were created for both tumour and normal samples (when available) individually and then merged to create a combined graph of all events identified in the PCAWG dataset. SplAdder was used to extract alternative splicing events of the following types: alternative 3' splice site, alternative 5' splice site, cassette exon, intron retention, mutually exclusive exons,

coordinated exon skip. A percent spliced in (PSI) value was calculated for each event and was used for further analysis.

### 4.4 Fusion transcript identification

Two different gene fusion detection pipelines were used for identifying fusions: FusionMap (version 2015-03-31) pipeline[98] and FusionCatcher (version 0.99.6a)/STAR-Fusion (version 0.8.0) pipeline[99]. FusionMap was run on unaligned reads from TopHat2. To reduce false positive fusions, fusion calls were excluded based on the number of supporting junction reads, sequence homology, and occurrence in normal samples. A high-confident consensus fusion call set was generated from fusions that were detected by both tools in at least one sample; and/or be detected by one of the methods and had a matched SV in at least one sample.

## 5. Clustering of Tumour Genomes Based on Telomere Maintenance-Related Features

Using the TelomereHunter tool, Sieverling[100] analyzed the 2,518 PCAWG whole genome samples and produced 12 telomere related features for each sample. TelomereHunter takes WGS BAM files and extracts reads with telomeric repeats. The telomeric repeats are filtered according to alignment coordinates and further categorized. The total telomere length is then calculated with a consideration of GC bias. The 12 features used in Sieverling to determine telomere maintenance defects included counts of nine different telomere variant repeats (TVRs) within the telomere, the number of telomere-like insertions within the genome, the number of genomic breakpoints, and the telomere length as a ratio between tumour and normal **(Supplementary Table 11)**. For this specific analysis the TVR count was determined by calculating the difference from a regression line through the normals, as opposed to a line through the TERT modified samples as was performed in Sieverling[100].

The T distributed stochastic neighbor embedding (T-SNE) algorithm was applied to the 12 telomere related features using the R package Rtsne. The Perplexity and alpha Rtsne parameters were set to 15 and 0.5 respectively. First the T-SNE algorithm was applied to both normal and tumour showing a distinct separation between normal and tumour (see **Extended Figure 13A**). Density-based spatial clustering of applications with noise (DBSCAN) was then applied to the first two dimensions of the t-SNE output and revealed 4 tumour clusters and 4 normal clusters (**Figure 7A, Extended Figure 13A**). Student t-tests revealed the most significant features among clusters (**Supplementary Figures 10-11**). A Fisher's Exact test (using the fisher.test package in R) was performed to determine if any gene has significantly more mutations among the samples in one cluster compared to those outside the cluster (or vice versa). Only the genes present in the curated TMM list from the TelNet Database were considered[101] (**Supplementary Table 12**).

# 6. Clustered mutational processes in PCAWG

These methods were used to infer the presence of the mutational processes of chomothripsis, kataegis and chromoplexy, and to characterize their clustering patterns.

## 6.1 Inference of chromothripsis

The landscape of chromothripsis regions across PCAWG samples is described by Cortés-Ciriano[102]. Here, an independent set of filters was developed focused on obtaining and timing punctuated chromothripsis events. To maximize confidence, the overlap between the two sets of calls was taken as the final set.

Chromothripsis events were inferred by integrating copy number profile (LogR), B allele frequency (BAF), and DNA rearrangement (SV) data. Across each chromosome arm, a piecewise constant fit was performed on the segment lengths to identify regions with high SV breakpoint density (average segment length < 3Mb = 150Mb / 50). Flagged regions on the same chromosome arm were merged. To discriminate punctuated events from sequential ones, the distribution of segment lengths within each region was compared to an exponential distribution with the same breakage rate, which is the distribution expected for random sequential breaks. The rate is computed by maximum-likelihood fitting of the observed distribution against an exponential distribution. A region is retained if the Kolomogorov-Smirnov test $p < 0.01$.

Whereas prototypical chromothripsis events exhibit copy number oscillations involving two copy number states[103], chromothriptic regions can present with more states, for example owing to prior or subsequent DNA rearrangements affecting the genomic region in question. As the number of breakpoints increases, the average segment size decreases, which may reduce the accuracy of somatic copy number calling. To allow for small biological variation and copy number errors, we scaled the expected minimal fraction that at most three allele-specific states must cover as $f_{expected} = min\,(1, -0.006N + 1.1)$, with $N$ the number of segments in the region. The linear relationship between $f_{expected}$ and $N$ is represented by the line passing through points A ($N = 50, f_{expected} = 0.8$) and B ($N = 100, f_{expected} = 0.5$). Regions were considered when at least $f_{expected}$ x 100 % of the region was covered by at most 3 allele-specific copy number states. As previously proposed by Kinsella et al, $N$ should represent a large number to reject the sequential-event hypothesis[104]. Here we set $N_{arm} = 30$ as the minimum number of breakpoints per chromosome arm. Next, we performed a multinomial test on the numbers of rearrangements showing a tandem duplication, head-to-head inversion, tail-to-tail inversion and deletion pattern. The null model states equal probability of 0.25 for each class, based on the reasoning that chromothripsis events should generate a near random orientation of fragment joins[105]. Occasionally, chromothripsis involves distal genomic regions or even different chromosomes, the result of which are derivative chromosomes harbouring sequences from more than one chromosome[103]. We reasoned that utilization of this information may facilitate 'rescuing' regions that exhibit too few breakpoints overall for reliable inference of chromothripsis. To this end, chromothripsis events displaying > 20 SVs linking to a region on a different chromosome were considered as 'connected' to that distal region. If not, they were still considered connected if > 20% of SVs

(at least 6) of the chromothriptic region and > 50% of the SVs (at least 6) in the distal region connected the two regions. Finally, we filtered out 33% of regions as they did not overlap ≥ 50% with region called by Cortés-Ciriano[102], leading to a high-quality set of punctuated chromothripsis calls.

### *6.1.1 Chromothripsis drivers and patterns*

In each cancer type, we tested the association of chromothripsis with whole genome duplication using an exact binomial test where the probability of success in each trial was the fraction of diploid samples that had chromothripsis. For each single base substitution and double base substitution signature and in each cancer type, we also tested the association of the exposure in each sample with chromothripsis using Mann-Whitney U tests. We also tested the association with the age of the patients through the same rank-based tests. Then, in each cancer type and for each driver event present in at least 5 samples, we tested for the association of chromothripsis with driver status using the Fisher-Boschloo test from the R package *Exact* v1.7.

Finally, we tested the significance of association between chromothripsis and driver events in the same region in two ways. First, we assessed how many drivers in the cohort are expected to be hit by the same number of chromothripsis events of similar complexity but happening at random along the genome. Therefore, we randomised all chromothripsis events along the genome. In each randomisation exercise, the probability to be assigned to a chromosome was proportional to the size of the chromosome. A random offset was added to the original start and end positions and taken uniformly from $U(-(S_{event} - 1), C_{size} - E_{event}))$, where $S_{event}$, $E_{event}$ are the start and end positions of the event and $C_{size}$ is the size of the new assigned chromosome. We repeated this 1,000 times and for each randomisation of the whole set, we counted the number of drivers amplified or homozygously deleted, leading to a distribution of expected counts of drivers with randomised chromothripsis positions.

Second, we tested for two types of enrichment of chromothripsis at driver gene loci: 1) within each cancer type, we tested for enrichment of one driver event against all other driver events using an exact Binomial test where the probability of success in each trial is the average fraction of other drivers co-occurring with chromothripsis in that cancer type; 2) for each driver event and cancer type, we also tested for enrichment of chromothripsis with that driver event in that cancer type against the average fraction of that driver event co-occurring with chromothripsis in all other cancer types, again using an exact Binomial test. P-values were adjusted for multiple testing by controlling the false discovery rate according to Benjamini and Hochberg.

### *6.1.2 Timing of amplified chromothripsis using SNVs*

We further timed the amplified chromothripsis events in molecular time by counting the number of SNVs present on all copies of the major allele, i.e. present before the amplification, and comparing it to the background mutation rate across the genome. We defined a segment *s* as amplified if the number of copies of the major allele $N_{maj,s} > 5$. For each segment, we first counted the number of SNVs present on all copies of the major allele $C_{amp}$ using the inferred multiplicities of the mutations $mult_i$. We counted the mutation *i* if $mult_i > N_{maj,i} \times 90\%$. We then derived an average total mutation rate per Mb along the genome by looking at 1+1

copy number segments >5Mb ($\mu_{tot} = \frac{\#SNVs}{2*segment\ length}$). We then calculated the relative timing of the amplified material as $t_{amp} = \frac{\frac{C_{amp}}{l_{amp}}}{\mu_{tot}}$, where $l_{amp}$ is the length of the amplified segment. We obtained 95% confidence intervals by bootstrapping the SNVs 1,000 times, during which, if $C_{amp} = 0$, we added a pseudocount to $C_{amp}$: $t_{amp} = \frac{1}{\mu_{tot}}$.

### *6.1.3 Quantifying the time spent during amplification after chromothripsis*

In amplified material, we observed that almost no mutations were seen at intermediate multiplicities ($N_{minor}$<multiplicities<$N_{major}$) even in hypermutators. This led us to speculate that it could be an indication that amplification proceeded quickly in molecular time. To test this intuition, we simulated the amplification as a stepwise increase in number of copies of each segment. Each time step would represent a fraction of total molecular time, i.e. would carry a fraction of the total mutation load. This fraction can be modulated so that early steps are slower and later steps are faster and vice versa. For this exercise, we took time steps of equal length. We amplified each segment independently so that the last amplification step is synchronous for all segments. The cumulative molecular time spent during amplification is modelled as a fraction of the total molecular time. Assuming constant mutation rate over time, each time step can be modelled by modulating the mutation burden accordingly. We derive the mutation rate per Mb along the genome $\mu_{total}$ from 1+1 and 1+0 regions of the genome as above. We did not count subclonal SNVs, i.e. SNVs with P(clonal)<0.5. When modelling amplifications, for each segment of length $l$ in Mb and at each time step of length $l_t$, a number of SNVs was drawn from a Poisson distribution with rate corresponding to the expected number of mutations given the genome wide mutation rate per Mb $\mu_{total}$: $Pois(\mu_{total} \times l \times N_{current} \times l_t)$, where $N_{current}$ is the number of copies present when the SNV appeared. We followed the multiplicities of each simulated mutations, calculated as $\frac{N_{major}}{N_{current}}$.

Starting from the observed copy number profile of an amplified chromothriptic region in a sample, we simulated 1,000 amplifications for each $f_{time\ spent} \in \{1,0.1,0.01,0.001\}$ = total fraction of molecular time spent during amplifications. We then counted the number of SNVs at intermediate multiplicities defined as SNVs with multiplicities satisfying ($max\ (N_{minor} + 1,5) < mult_i < N_{major} \times 75\%$) and modelled the distribution of counts as a negative binomial using maximum likelihood fitting. This allowed us to derive normalised likelihoods for each $f_{time\ spent}$ to generate the observed counts of SNVs with ($max\ (N_{minor} + 1,5) < mult_i < N_{major} \times 75\%$) after removing kataegis foci.

## 6.2 Inference of chromoplexy

For the purposes of identifying chromoplexy events we utilized the merged set of somatic SVs, along with a classification scheme developed by the PCAWG SV working group[49]. This scheme involves the clustering of SV breakpoints by proximity, and subsequent evaluation of breakpoint clusters, termed ‘footprints’, according to orientation, copy number context and connectivity of adjacent rearrangements. Chromoplexy footprints consist of two breakpoints with a +/- (low/high) orientation and a dip in copy number between the breakpoints. Such footprints were joined together into an event by their interconnecting SVs. The events were classified as a chromoplexy chain or cycle if all constituent footprints were of this type.

Balanced translocations constitute the simplest chromoplexy cycles. SV signature analysis demonstrated that balanced translocations, but not unbalanced translocations or chromoplexy chains of length 2 (i.e. a single footprint with SVs not connecting to another), co-occurred with longer chromoplexy events in the same signature. For the purpose of our analysis we included all chromoplexy chains and cycles, except those chains of length 2.

## 6.3 Inference of kataegis

Regions exhibiting localized hypermutation were identified by performing a piecewise constant fit of inter-mutation distances. Multinucleotide variants were considered as single mutations at the position of the first SNV. Sets of at least $K$ adjacent consensus SNVs were flagged as candidate kataegis events if the segmented inter-mutation distance dropped below a threshold $d$. The thresholds $(K, d)$ depend on the total mutational burden $mb$ of the sample as follows. The inter-mutation distance $(X)$ in a sample is robustly modeled assuming an exponential distribution with rate $\lambda = \frac{ln2}{median(X)}$. The probability of observing an inter-mutation distance $\leq d$ can then be computed as $P(X \leq d) = 1 - e^{-\lambda d}$. Setting this as the probability of success $p$ in a Bernoulli trial and assuming $N \gg K$, we can estimate the probability of a streak of $K-1$ successes (i.e. K adjacent SNVs) in $N$ $(\sim mb)$ trials as $S(N, K-1) = N(1-p)p^{K-1}$. We limit $S(N, K-1) \leq 0.01$, consider $p \ll 1$ and work back to derive the threshold $d \leq \frac{-ln(1-\sqrt[K-1]{\frac{0.01}{mb}})}{\lambda}$ for $K\ in\ (4, 5, 6)$. We set a ceiling $d_{max} = 1$kb and take the pair $(K, d)$ which first maximizes $d$ and then minimizes K.

Kataegis candidate foci were subsequently annotated by the mutational signature(s) contributing to each focus, using PCAWG mutation signature classifications developed by the mutation signature working group using non-negative matrix factorization[53]. Cosine similarities and multinomial likelihoods were computed between the mutational spectrum of each focus, adjusted for local trinucleotide content, and the mutational signatures that are operating in the sample (including previously unreported signatures such as the C[T>N]T kataegis that we identified during initial analyses, and the merged signature 2/13). Cosine similarity biases towards 'peaky' signatures, while the likelihood is sensitive to contributions from background SNVs (in which case it biases towards 'flat' signatures). Balance was achieved by summing the ranks of their scores and assigning the focus to the signatures with the lowest rank sum.

Two additional metrics were devised to verify the simultaneous, as opposed to sequential, nature of the events, with kataegis candidate foci having to meet at least one of two criteria: (1) Typical APOBEC-type kataegis events occur on a ssDNA template[106] and initial analyses suggested that this holds true for at least some other mutational processes contributing to kataegis. To assess 'strandedness', we consider the sequence of mutated reference strand bases in the focus and compute the probability of a streak of a single base that is at least as long as the longest one observed, given the local background nucleotide frequency. For example, in a focus of $N = 6$ SNVs, with $K = 5$ consecutive mutations at reference C nucleotides and a background frequency $p = 0.22$ of C, this probability is given by the following recursion $S(N, K) = p^K + \sum_{j=1,K} p^{j-1}(1-p)S(N-j, K)$ to be equal to $9.17 \times 10^{-4}$. *P*-values were corrected for multiple testing by controlling the false discovery rate according to Benjamini and Hochberg, and foci were considered to exhibit strand bias when $q \leq 0.1$.

(2) Point mutations mapping to a single kataegis events should phase to a single haplotype. To confirm that SNVs reside on the same chromosomal homolog, we used the mutation-to-mutation phasing information extracted from the aligned bam files by the PCAWG evolution and heterogeneity working group[107]. Briefly, we obtained phasing information for all SNV pairs that are within 700bp. Low quality reads and base calls were filtered out by setting minimal mapping and base qualities thresholds of ≥ 20. Reads that were not properly paired, were flagged as duplicates or had a failed vendor quality control flag were also removed from consideration. Kataegis generates SNVs on the same physical chromosome, as a result, reads should report either the mutant or the WT alleles of the two variants (*Mut-Mut* or *WT-WT* reads) but not just one of each (*Mut-WT* or *WT-Mut*). Candidate foci were considered punctuated if ≥ 75% of SNVs were in-phase with one another.

Finally, in line with metric (2), any focus in which ≥ 10% of the SNVs show evidence of sequential mutagenesis (i.e. both *Mut-Mut* and *Mut-WT* or *WT-Mut* reads) or anti-phasing (i.e. only *Mut-WT* and *WT-Mut* reads) is filtered out.

### *6.3.1 Analysis of kataegis drivers and patterns*

The R package blme was used to fit Bayesian generalized linear mixed effect models of the number of APOBEC kataegis foci per sample. We performed Poisson regression iteratively including the PCAWG drivers and cytidine deaminase expression levels as fixed effects, while controlling for tumour type (random intercept per tumour type and slope for drivers/cytidine deaminase expression) as well as the number of structural variants and donor age at diagnosis (fixed effects, both log-transformed, centered and scaled). Patient sex (as inferred from the sequencing data) was found not to carry significant predictive value and was further excluded from the modeling. A standard normal prior was placed over the modelled coefficients (fixed effects) while a default Wishart prior was used for the covariance of the random effects. Coefficient *p*-values were adjusted for multiple testing according to Benjamini and Hochberg. Forward selection using drivers and cytidine deaminase gene expression with $q \leq 0.05$ and evaluation of BIC, AIC and likelihood ratio tests of the full model with the variable in question against the model without it resulted in an optimal model incorporating *APOBEC3B* expression levels, the number of structural variants, patient age at diagnosis (random effects) and tumour type (fixed effect). In R/lme4 formula syntax this final model is described as follows:

$$\#APOBEC\ foci \sim APOBEC3B\ expression + age\ at\ diagnosis + \#SVs + (1|tumour\ type)$$

Accounting for overdispersion by repeating the exercise above with a negative binomial model (R package glmmTMB) yielded identical conclusions.

Samples in the top ~5% of kataegis burden (> 30 foci) were classified into 4 groups: (non-APOBEC) < 50% of foci with an APOBEC signature; (SV-associated) ≥ 50% APOBEC foci and ≥ 45% of foci within 1 kb of a breakpoint; (rearrangement-independent) ≥ 50% APOBEC foci and ≤ 20% of foci within 1 kb of a breakpoint; (mix) ≥ 50% APOBEC foci and 20-45% of foci within 1 kb of a breakpoint. Replication Fork Directionality (RFD) measurements from sequencing of Okazaki fragments (OK-Seq) in HeLa cells was obtained from Petryk[108]. Replication timing measurements derived from percentage-normalized and wavelet-smoothed Repli-Seq signals were obtained from ENCODE/University of Washington[109].

## 6.4 Subclonal architecture reconstruction

To determine the probability of a clustered event being (sub)clonal, we used the clonal-subclonal assignment probabilities for SNVs and SVs produced by the PCAWG evolution and heterogeneity working group[110]. Briefly, for each PCAWG sample, 11 distinct subclonal reconstruction methods were run on the consensus SNVs in the highest confidence consensus copy number segments covering at least 75% of the genome. Their outputs (i.e. # mutation clusters, # mutations/cluster, tumour cell fraction of each cluster and mutation assignments to clusters) were combined by taking a weighted median of the locations (cellular prevalences) and the proportion of SNVs assigned to the location, to construct a robust consensus subclonal architecture description. This consensus architecture, together with the full consensus copy number and all consensus SNVs, indels and SVs for which allele frequencies were available, were fed into the MutationTimer algorithm[11]. MutationTimer describes mutation clusters using a beta-binomial model and derives assignment probabilities for each mutation belonging to each cluster while taking into account cluster size. The result is the complete consensus subclonal architecture with probabilistic assignments of mutations to (sub)clones. MutationTimer also splits up the probability of a variant $j$ being clonal ($1 - p_{sub,j}$) into the probability that it is present on a single or on multiple chromosomal copies ($p_{single,j}$ and $p_{gain,j}$, such that $p_{single,j} + p_{gain,j} + p_{sub,j} = 1$). In regions with chromosomal gains, this allows timing of clonal variants as clonal early, clonal late or clonal NA (i.e. indistinguishable), depending on whether they occurred before or after the gain. Mutations which are present on multiple copies in a region with copy number gains are classified as clonal early. If they are present on a single copy of the gained chromosome and there is loss of heterozygosity in the region, they are considered clonal late, as they must have happened after the gain on that same chromosome. All other clonal variants are classified as clonal NA.

## 6.5 Clonality assessment of punctuated events

For every punctuated event, we computed the probability of it being clonal as the normalized likelihood using the clonal assignment probabilities of the constituent SNVs or SVs. For instance, for an event $i$ involving $N = 1 \dots j$ variants, each with an associated probability $1 - p_{sub,j}$ of being clonal in the tumour sample, the likelihood of being clonal was determined as $\prod_{j=1}^{N} \quad (1 - p_{sub,j})$ and of being subclonal as $\prod_{j=1}^{N} \quad p_{sub,j}$. The likelihoods were normalized to yield probabilities for (sub)clonality of the event ($p_{cl,i}$ and $p_{sub,i}$). We also computed the probability of every event being clonal early, clonal late or clonal NA ($p_{early,i}$, $p_{late,i}$ and $p_{NA,i}$, respectively) using the probabilities that the variants involved are clonal and present on a single chromosomal copy, on multiple copies, or they are subclonal, as derived by MutationTimer. Normalised likelihoods were computed using the variants in the event stratified by consensus gain/LOH status (consensus copy number), weighted by the fraction of variants in each class, and summed according to the rules for distinguishing clonal early/late/NA (as described above) to obtain the final probabilities.

The odds of observing clonal versus subclonal events of different types (kataegis, chromoplexy, chromothripsis or simple events such as SNVs, indels or SVs) were computed for every cancer type by bootstrapping the ratio $\frac{\frac{\sum p_{cl,i} + 0.5}{\sum p_{cl,sim,i} + 0.5}}{\frac{\sum p_{sub,i} + 0.5}{\sum p_{sub,sim,i} + 0.5}}$ where 0.5 represents a

pseudocount (i.e. a single event with $p_{cl,i} = p_{sub,i} = 0.5$) and $p_{cl,sim,i}$ and $p_{sub,sim,i}$ are the clonal and subclonal assignment probabilities of a simulated event matched to observed event $i$. For every punctuated event observed, we simulated 10,000 comparable events by sampling the same number of SNVs or SVs from the background of non-punctuated variants with identical gain/LOH status in that sample. Clonal and subclonal assignment probabilities ($p_{cl,sim,i}$ and $p_{sub,sim,i}$) as well as probabilities of being clonal early, late or NA ($p_{early,sim,i}$, $p_{late,sim,i}$ and $p_{na,sim,i}$) were computed for the simulated events as described above for the observed events. To obtain the median odds ratio and 95% percentiles, 10,000 bootstrap replicates of the observed events dataset were generated while a different matched set of events was used for each iteration. During the bootstrap, events were weighted according to $\frac{1}{(\# \, events \, in \, sample)}$ to give equal weight to samples with different numbers of punctuated events. The odds of observing clonal early versus clonal late events were computed similarly by bootstrapping the ratio $\frac{\frac{\sum p_{early,i} + 0.5}{\sum p_{early,sim,i} + 0.5}}{\frac{\sum p_{late,i} + 0.5}{\sum p_{late,sim,i} + 0.5}}$.

### 6.6. Data Availability

All kataegis, chromoplexy and chromothripsis calls generated in this section are available from Synapse (syn12978907).

# 7. Tumours without detected driver mutations

Average detection sensitivity for each samples was calculated from mean coverage and consensus purity and ploidy as described in Rheinbay *et al*[111]. To rescue mutations lost to tumour in normal contamination, we applied the deTiN as described previously[112] (https://github.com/broadinstitute/deTiN). Tumour-in-normal (TiN) contamination was estimated from unfiltered Broad pipeline SNV (called by MuTect) and Sanger pipeline indel calls and allelic copy number data. Briefly, deTiN estimates TiN by measuring the proportion of DNA supporting somatic aberrations in each sample and then recovered indels and SSNVs previously discarded as possible germline events based on this estimate. Bone tumours were removed from this analysis due to a high number of artefact calls[53]. Variants recovered by deTiN are limited to those that otherwise would have been rejected by MuTect due to low-allele fraction presence in the normal.

Driver discovery was performed on 169 tumours without drivers after the described rescue of events present in the matched normal, TERT hotspot mutations, and power considerations. Coding and non-coding significance analyses for tumour cohorts was carried out using MutSig as described in Rheinbay *et al*[111]. GISTIC version 2.0.23 was run on PCAWG consensus copy number[107] for all samples and individual cohorts with at least five tumours with parameters -conf 0.95 -savegene to identify significant copy number gains and losses[113]. Medulloblastoma group information was obtained from Northcott et al, 2017[114].

# 8. Panorama of driver mutations in human cancer

## 8.1 The onCohortDrive method

The discovery of mutational cancer driver genomic elements (GEs), both coding and non-coding, has received much attention in recent years, with several methods specifically developed to address it. The approach followed by all these methods consists in identifying the GEs with signals of positive selection from the deviation of their mutational pattern across a cohort of tumours from the random expectation[115–122]. However, the identification of the individual driver mutations in each patient remains an open problem because of two main reasons. First, not all observed mutations in driver GEs are tumorigenic[123,124]. Second, currently probed cohorts of tumours are underpowered for the identification of all driver GEs using these methods[125]. (Note that, for simplicity, here we use the term mutation to refer to a point mutation, as defined in the paper.)

To address the problem of accurately identifying driver mutations in the PCAWG cohort, we developed onCohortDrive, which is predicated on two principles derived from the aforementioned hurdles. On the one hand, it assumes that not all mutations in driver elements are drivers. On the basis of this predicate, it also assumes that an accurate mutational null model – taking into account mutational processes and genomic covariates of the mutation rate – may be computed for each GE across a cohort of tumours. The comparison of the number of mutations detected in a GE with their expected number derived from this null model then yields an excess of observed mutations above expected, which can be used as an estimate of the number of driver mutations in the GE across the analysed tumours. On the other hand, the method considers that some driver GEs are below the statistical power of the cohort of tumours and the methods employed for their discovery. It therefore starts from a Compendium of Mutational Driver GEs (see section 8.2) composed not only of those discovered in the cohort under analysis (i.e., discovery GEs), but also of other GEs with prior knowledge of involvement in tumourigenesis (i.e., prior knowledge GEs).

On discovery GEs with computable mutational excess, onCohortDrive ranks mutations in each GE according to a number of features associated to their likelihood of being tumorigenic, and then nominates as likely drivers those at the top of the ranking, up to a number equal to the estimated excess as 'drivers by rank'. We tested several ranking features that could be applied to mutations in all types of elements, and selected two: the CADD[126] functional impact score and whether mutations occur in clusters. In combination, the combination of these two features improved the relative rank of known oncogenic mutations[127] with respect to random. We also observed that known driver mutations exhibit significantly lower probability (computed using ncdDetect[120]) to occur than other mutations. This is not surprising: while passenger point mutations are expected to occur with the probabilities dictated by the predominant mutational process in the tissue, the drivers are also favoured by positive selection. We therefore used the probability of occurrence of each mutation as another feature to rank the mutations in driver elements. Finally, we also used several features specific to different types of GEs, such as the mode of action of the driver, the creation or disruption of transcription factors or microRNAs binding sites (TFBS, microRNABS) in coding

genes, promoters, and 3'UTRs, respectively. For a few (n=6) discovery GEs, we are unable to compute a mutational excess: point mutations in these are classified as drivers or passengers following a rule-based approach, derived from establishing thresholds of the same features employed in the ranking, which we call 'drivers by rule' (see **Section 8.4.5**).

To compute the number of driver point mutations of a discovery GE across a cohort of tumours, in this implementation of onCohortDrive we use the excess of mutations above the background rate estimated by NBR[128,129], a method that identifies driver genes based on the recurrence of mutations across tumours. Briefly, NBR computes the expected number of mutations in a GE accounting for trinucleotide mutational signatures, sequence composition of the GE and the local density of mutations around it. The trinucleotide mutational signatures are computed from all the mutations observed across all GEs. A first estimate of the background mutation rate of the GE is done using only this trinucleotide composition and is subsequently refined using known covariates of the mutation rate, such as the local density of somatic mutations (normalized by sequence composition), the regional replication timing and the expression level.

Once the excess for each GE is computed, its mutations across tumours are ranked based on several features that appraise their likelihood of being tumorigenic. These features were selected on the basis of prior knowledge and careful evaluation of their performance on the task of ranking known driver mutations in the cohort (see **Section 8.3**). Calculating the excess of mutations from the cohort allows the approach to be rank-based rather than categorical. It may be applied to GEs bearing signals of positive selection and a mutational excess in the cohort under analysis. Within the context of PCAWG, these GEs are detected applying an array of statistical methods to their observed mutational pattern.

For prior knowledge GEs, and discovery GEs where NBR fails to compute a mutational excess, onCohortDrive implements a rule-based method to identify putative driver mutations. Both methods, the rank-based and the rule-based are coupled within the workflow of onCohortDrive, so that GEs undergo either one or the other on the basis of whether or not they have been found to carry a significant mutational excess. The algorithm is designed with the purpose of being equally functional on both coding and non-coding GEs (see below).

The implementation of onCohortDrive is the result of a collaborative effort with the PCAWG Drivers and Functional Interpretation Group. Namely, we obtained from several labs within the working group i) the Compendium of Mutational Driver GEs; ii) the mutational excess computed using NBR (see above); and iii) several features of the mutations, some of which we ultimately employed in their ranking (see below).

We have benchmarked the performance of onCohortDrive (see **Section 8.4**) on its ability to correctly classify a set of known driver mutations and benign mutations observed in driver GEs in the PCAWG cohort.

## 8.2 The Compendium of Mutational Driver GEs

The compendium of mutational driver GEs is composed, first by all driver GEs with signals of positive selection in their pattern of point mutations across the cohort (discovery compendium), with the exception of five coding genes and one promoter region of a coding gene identified in the lymphoma cohorts with a high proportion of mutations introduced by

AID (>40%). It also contains manually collated and curated previously known cancer GEs – driving tumourigenesis through point mutations, CNAs and/or balanced genomic rearrangements of somatic origin– across tumour types. This list of drivers comprises: a) GEs with validated tumorigenic effect, obtained from the Cancer Gene Census (CGC[130] and literature reports (with special attention paid to recently reported instances of driver non-coding genomic elements[127]), and b) genes whose mutational patterns show signals of positive selection across previously analyzed cohorts of tumour exomes or genomes[113,131,132]. Each GE in the Compendium is annotated with the list of cancer types where it has been linked to tumourigenesis and the type of evidence and source on which this link relies. Furthermore, the mode of action of GEs in tumourigenesis (either loss-of-function, activating, or unknown) is also annotated. The Compendium of mutational driver GEs is available at syn11679360.

We also compiled a list of known driver point mutations integrated by a previous collection of known tumorigenic coding point mutations[127] and a few manually collected known driver non-coding point mutations from the literature[133–137]. Three known and one novel (in PCAWG) POLE proof-reading affecting mutations[138,139] were also included. Furthermore, we included an inframe indel, which causes a three amino acid insertion (p.R506_insVLR) in the BRAF protein, based on a previous study[140]. We used this list of known driver point mutations as part of the onCohortDrive workflow and as part of its benchmarking (see below).

## 8.3 Features

Both ranking the mutations in a GE so that likely driver mutations appear at the top, and using rules to discretely classify the mutations observed in a GE rely on a series of features that can be measured for all mutations across GEs. These features need to show different distribution within driver and passenger mutations in driver GEs. To decide which features could inform both the ranking and rules-classification processes we relied on two types of metrics. On the one hand, we compared the distribution of values of a feature for groups of known oncogenic mutations and somatic variants of cancer genes experimentally verified to be benign, or polymorphisms. On the other, we checked the ability of the features to produce a ranking of mutations observed across PCAWG tumours in which known driver mutations observed in the cohort appeared systematically at the top. We measured this through the relative rank of known drivers produced by a feature and its comparison with a random ranking of the mutations.

Below, we describe the features that after careful selection, were incorporated to rank and rule-classify mutations in onCohortDrive, and we describe the rationale followed to select each of them. Note that although the features used and their implementation are similar to those frequently employed to identify signals of positive selection on GEs across cohorts, within onCohortDrive we only use them to rank mutations that appear in GEs that have already been identified as drivers. Moreover, different features contribute differently to the ranking of mutations (see **Section 8.3.4**).

### *8.3.1. Functional Impact (FI)*

A mutation conferring a selective advantage must increase the cell fitness, and this is achieved by acquiring, enhancing or disrupting cellular functions. We therefore hypothesized that

cancer driver mutations tend to possess a high functional impact on the proteins they affect[130,141,142].

One of the most widely used metrics for quantifying the deleteriousness of specific mutations is the CADD score[126], which integrates multiple annotations such as conservation across evolutionarily related sequences, functional genomics data and protein-level scores. The CADD score of known driver in cancer genes (from the Cancer Gene Census[130], CGC) is greater than that of other mutations observed in PCAWG, all possible mutations and known polymorphisms in cancer genes. We therefore decided to use the CADD score of mutations to rank all mutations in one GE across tumours.

### *8.3.2. Clustering of mutations*

Driver mutations are known to cluster in certain regions of some cancer proteins[143]. These clusters could mark sensitive regions in the primary structure of tumour suppressor genes and regions relevant for the activation of oncogenes. Methods to identify driver GEs have exploited the clustering of mutations as a signal of positive selection. Nevertheless, the difficulty in correctly modelling the background mutational processes taking into account all influences at the local level[144] makes it hard to fine-tune these methods to avoid the identification of many false positive GEs. In the context of onCohortDrive, we use the clustering of mutations solely as a feature to rank the mutations detected in GEs with signals of positive selection following their likelihood of being drivers. In other words, in GEs that have been identified as drivers, we deem the mutations in clusters more likely to be driver mutations than non-clustered mutations.

For each GE (G), coding or non-coding, we aim to assess to what extent each mutational cluster formed by the mutations observed in G provides an extremal likelihood assuming a background probability distribution that we infer from the mutations observed in G.

We define a mutational cluster to be any connected subsequence $C \subset G$ such that: i) C encloses more than two mutations; ii) both the first (5') and last (3') positions of C correspond to mutations; iii) any two consecutive pairs of mutations in C are located within 30bp distance from each other; iv) C is maximal with respect to the above mentioned properties, i.e., it is not properly contained in another sequence satisfying conditions i), ii), and iii). In order to specify the clusters in G, we only require the mutations of the cohort alongside their respective positions. This definition is meant to be an abstraction of a mutational hotspot.

Next, we want to compute the likelihood to observe m mutations in C taking into account an estimated mutation rate at each base position. Thus, we require a discrete probability distribution that assigns a likelihood to any sample of mutated positions of C. Moreover, the sought distribution must allow to consider distinct mutation rates per base position. In view of these requirements, the probability model of choice is a Poisson Binomial distribution. Taking into account that multiple mutations can occur in the same base position in the cohort, we define $\tilde{C}$ as the DNA sequence consisting of as many copies of C as samples are in the cohort. The Poisson Binomial distribution will draw mutations from $\tilde{C}$.

The probability that a mutation is observed in a base position $x$ of $\tilde{C}$ will be considered to depend on the reference tri-nucleotide at $x$, i.e., the base at position $x$ alongside its 5'/3' flanking bases. In order to estimate this probability, we first consider two different mutation

rates per base: i) the overall mutation rate per base position in G, $r = m/N$, i.e., where $m$ is the number of mutations observed in G, and N is the number of base positions in G multiplied by the number of samples in the cohort; ii) the tri-nucleotide specific mutation rate $r_\tau = m_\tau/N_\tau$, where $m_\tau$ is the number of mutations observed at tri-nucleotide $\tau$, and $N_\tau$ is the abundance of $\tau$ in $\tilde{C}$. Then, for each base position of $\tilde{C}$ with tri-nucleotide $\tau$ we set the mutation rate:

$$p_\tau = \frac{N}{N + N_\tau}.r + \frac{N_\tau}{N + N_\tau}.r_\tau = \frac{m + m_\tau}{N + N_\tau}.$$

We implemented this model using the Python package poibin[145]. Equipped with an appropriate implementation of our Poisson Binomial probability model, for each cluster C we compute a P-value, meaning the exact probability under the Poisson Binomial model to draw as many or more mutations from $C \subset G$ than the number observed in C. Furthermore, mutational clusters with P-value smaller than 0.05 were deemed significant. As explained above, clustered mutations (i.e., within a significant cluster) in a driver GE are regarded as more likely drivers than non-clustered mutations. The algorithm then uses this information to re-organise the ranking of mutations (**Section 8.3.4**).

### *8.3.3 Ranking mutations based on FI and clusters*

Next, we checked whether ranking mutations in driver GEs based on these two features (FI and clusters) is an effective way to identify drivers. To this end, we compared the relative rank of known driver mutations resulting from ranking the mutations in all GEs randomly, using the CADD score alone and combining the CADD score and the clustering. (For the CADD + clusters rank, we first ranked known driver mutations in GEs based on their CADD scores, and then the mutations found in a significant cluster were moved to the top of the ranking, preserving their originally established order.) This analysis demonstrated that ranking the mutations using CADD score ranks known driver mutations higher than random ranking, and that the ranking based on both features produced better relative rank than CADD alone. We therefore decided to use the combined rank of FI + clusters in onCohortDrive.

### *8.3.4. Mutational unlikeliness*

We also hypothesised that driver and passenger mutations have different probability distributions. While passenger point mutations are expected to occur with the probabilities dictated by the predominant mutational process in the tissue, the drivers are also favoured by positive selection, and can therefore appear with the same or lower probability. Using the sample- and position-specific probabilities of mutations (ncdDetect[120]), we computed the average probability of occurrence of known driver mutations observed in each PCAWG tumour. Then, we compared the average probability of occurrence of driver mutations obtained for each tumour with the average probability of occurrence of 10,000 groups of mutations of the same size that we sampled randomly from the same tumour. We thus computed an empirical p-value estimating the bias of occurrence of known driver mutations towards lower or higher probabilities than other mutations. We repeated the analysis using only known coding mutations to guarantee that the observed bias was not caused by different probabilities of occurrence of coding and non-coding mutations, both in individual cohorts

and pan-cancer. We found that known driver mutations exhibit significantly lower probabilities of occurrence than other mutations in most cancer types, indicating that the mutational unlikeliness can be used to further refine the sifting between drivers and passenger mutations in driver GEs.

#### *8.3.5. Element specific scores*

The features described until now, namely FI, clusters and mutation unlikeliness, have the advantage that they can be computed for all variants in the genome. Some other features are relevant only for particular types of elements (**Supplementary Table 13**). For instance, the creation or disruption of transcription factor binding site (TFBS) is very informative for variants in promoters, 5'UTRs and enhancers. We selected different element-specific scores to aid the ranking of mutations following their likelihood of being drivers. All these scores were binary and stated whether a mutation in one of the following elements fulfilled the corresponding feature.

#### *8.3.6. Other evaluated features*

We evaluated other features for the method, which were eventually discarded for their use in onCohortDrive (**Supplementary Table 14**).

## 8.4. The onCohortDrive workflow

This section describes the workflow used to apply onCohortDrive to the PCAWG cohort.

#### *8.4.1. Overview*

The overall workflow implements a decision-making tree on all mutations identified in the cohort (47,022,343) that integrates the identification of known tumorigenic mutations, the ranking approach and the rule-decision approach. First, only mutations affecting GEs in the Compendium of Mutational Driver Elements enter this decision-making process; all other mutations are automatically labelled passengers. The algorithm then makes a decision on whether the mutations observed in a GE will undergo the ranking-based or the rule-based process. Mutations in GEs bearing significant signals of positive selection in particular cohorts, metacohorts, or the pan-cancer cohort (discovery GEs) and an excess of mutations above their background rate undergo the ranking process. Mutations in GEs without computable excess undergo the rule-based process.

#### *8.4.2. Analysing mutations in driver GEs*

The Compendium of Mutational Driver GEs is integrated by a wealth of data characterising each GE. Discovery driver GEs are annotated with the excess of mutations above the background rate, which is employed to decide the number of expected driver mutations of each element. Prior knowledge GEs are annotated with their mode of action (i.e. Loss of Function, Activating or ambiguous), either known or predicted[146]; the tumour type where they have been shown to promote tumourigenesis; and the original source reporting it, or the method that identified it (https://www.cancergenomeinterpreter.org/genes[127]).

Of note, lymphoma mutations in GEs with a high proportion (>40%) of mutations introduced by the AID enzyme during the somatic hypermutation process in the germinal center are deemed passengers. These mutations were identified using the PCAWG AID signature (syn11804065).

### *8.4.3. Identification of known tumorigenic mutations*

Known tumorigenic mutations (k) in GEs in the Compendium are first nominated as known-drivers, and subsequently excluded from the rank-based and rule-based approaches to identify drivers. This process is not followed in the unbiased exercise to estimate the contribution of non-coding driver mutations (see **Section 8.6**) nor in the benchmark exercise (see section 8.5).

### *8.4.4. Rank-based approach*

OnCohortDrive then deals with GEs bearing an excess of mutations above the background rate in cohorts of individual tumour types. Mutations in these GEs are processed in two steps.

Step 1:

Let $N$ be the total number of mutations in a GE in the cohort, and $n$ the number of mutations it carries in excess above the background rate across the tumours in a cohort (i.e. the expected number of driver mutations in the GE). Then, the number of mutations available to the rank-based approach is $N$-$k$, and the excess after identifying known tumorigenic mutations is $n$-$k$. First, the $N$-$k$ mutations in the element available to the rank-based approach are ranked based on their CADD scores. Then, mutations fulfilling element-specific binary features are moved to the top of the ranking, keeping the previously established order within them. Next, the mutations found in a significant cluster are moved to the top of the ranking, again preserving their originally established order. (Of note, clusters with high proportion (>50%) of lymphoma mutations introduced by the AID enzyme, identified using the PCAWG AID signature available at syn11804065, are deemed not significant and their mutations ranked accordingly.) Finally, mutations not fulfilling any binary feature-specific score and not in clusters are re-ranked based on the product of the rank of their unlikeliness and their functional impact. Finally, the $n$-$k$ mutations at the top of the resulting ranking are nominated as drivers.

Step 2:

The calculation of the excess depends on the size of the cohort. Therefore, the algorithm also exploits the excess found at a pan-cancer level. Let $r$ (residual excess) be the number of mutations in excess in the pan-cancer cohort after the mutations in excess in all tumour type cohorts have been processed as explained in step 1. These $r$ mutations are then processed, as explained, across all tumours in the pan-cancer cohort, excluding those in cohorts already processed in step 1.

Discovery GEs identified only when pooling several cohorts together (i.e., metacohorts) are processed within their respective metacohort as described in step 1. In other words, their mutations across all the samples in the metacohort are ranked as explained in Step 1, and

drivers are selected from the top of this ranking using the excess computed within the metacohort. No residual excess is computed in this case.

Finally, after all excess is exhausted, mutations in cohorts where the GE has not been identified as a driver are evaluated via the rule-based approach (see below). The rationale in this case is analogous to that of the use of the rules: driver mutations may still appear in cohorts where the GE is not identified as a driver due to a lack of statistical power for its detection.

#### *8.4.5. Rule-based approach*

Mutations in GEs with no excess above the background rate are processed with a rule-based approach. We reasoned that only a few mutations in prior knowledge GEs in the Compendium, which by definition are under the limit of detection of the cohort, are expected to be drivers. Furthermore, it is reasonable to assume that the likelihood of mutations in these GEs to be drivers should correlate with their unlikeliness or indirectly correlate with their probability to occur in each particular tumour (computed via ncdDetect; see description above). We designed rules to nominate driver mutations in these GEs taking into account the same features as in the rank-and-cut approach, namely i) their CADD functional impact score, ii) whether they occur in mutational clusters, iii) their unlikeliness, and iv) element-specific features.

To do this, we first separated the mutations affecting coding genes into six groups depending on their probability to occur in the tumours where they are detected. The groups were made such that the area under the curve of the probability density function was equal for the six groups. Mutations in groups with increasing probability then entered the rule-based process to be considered as possible drivers if they occurred in genes identified as drivers with increasing level of confidence (**Supplementary Table 15**). For instance, all mutations affecting genes with the highest confidence to contain driver mutations in the same tumour type (Level of confidence 1 in **Supplementary Table 15**) entered the rule-based process. However, only the most unlikely mutations in the tumour sample (Mutation probability group 1 in **Supplementary Table 15**) in genes with lower confidence in the tumour type entered the rule-based process (Level of confidence 6 in **Supplementary Table 15**).

Coding mutations that entered the rule-based approach are then nominated as drivers if any of the following is true:

a) The mutation causes a missense change in a significant mutational cluster;

b) The mutation results in the truncation of a transcript of a loss-of-function driver or *NOTCH1 or NOTCH2* (whose truncating mutations cause activation of the protein and its mobilization to the nucleus);

c) The mutation has a CADD score above the 95th percentile of the CADD scores of all coding genes.

Mutations affecting driver non-coding GEs were separated into three groups of increasing probability of occurrence, following the same rationale explained above for coding mutations.

Then, we processed the mutations in groups of increasing probability using rules of increasing stringency. We considered drivers the:

a) mutations in the group of lowest probability, with CADD score above the 95th percentile of CADD scores in GEs of the type,

b) mutations in the group of intermediate probability, fulfilling a binary feature score and with CADD score above the 90th percentile of CADD scores in GEs of the type, and

c) mutations in the group with highest probability that fulfil a binary feature score and with CADD score above the 95th percentile of CADD scores in GEs of the type.

The thresholds of CADD score were decided on the basis of the comparison between the distributions of CADD scores of known driver point mutations and other sets of point mutations.

The above explanation for the separation of mutations into groups of increasing probability of occurrence applies for autosomal SNVs. SNVs in sex chromosomes and indels are processed analogously, with the difference that the mutation probability groups are established on the basis of the count of mutations (either total SNVs or indels) in the sample, as no ncdDetect probability is available for these mutations. Finally, MNVs are separated according to the total count of SNVs. Then, these mutations are processed via the rule-based approach following special rules (**Section 8.5.2**).

Finally, after known, drivers-by-rank and drivers-by-rule mutations are identified, any mutation that is identical to a previously nominated driver mutation, or any coding mutations causing the same amino acid change, in a different tumour type is also nominated as a driver.

#### *8.4.6. Post-processing*

According to evidence provided by the PCAWG drivers discovery group, we classified as passengers mutation of RFTN1 that onCohortDrive deemed drivers, if their probability of being generated by the AID signature was greater than 0.5, and we promoted to driver-by-rule 11 point mutations of TP53 affecting either the 5'UTR or a non-canonical splice site in the first exon.

## 8.5. Benchmarking onCohortDrive

Next, we assessed the capability of onCohortDrive to correctly identify known cancer mutations in driver genes (as drivers) and known benign mutations in cancer genes (as passengers). To that end, we first identified all known cancer mutations (1032) and known benign mutations (12) in cancer genes in tumours in the PCAWG cohort from a total of 22854 SNVs in cancer genes of the Full Compendium. We next executed the analysis skipping the step of identification of known mutations. OnCohortDrive identified 3719 SNVs as drivers (16%). Of the 1032 known driver mutations it correctly classified 95% (982) as drivers, and all 12 benign mutations as passengers. Note that these 1032 known tumorigenic mutations actually correspond to 468 unique SNVs, some of which appear recurrently in several patients of the cohort. We count them separately for the benchmark, because they all compete in the ranking for the purpose of identifying drivers. In summary, while only 16% of SNVs in cancer

genes are classified as drivers by onCohortDrive, the vast majority of real driver mutations (95%) are correctly classified as drivers.

In addition to benchmark the full onCohortDrive, we specifically benchmarked the capacity of the rule-based approach to correctly classify drivers and passengers. It is important to bear in mind that the rules within onCohortDrive are designed to identify few driver mutations that may appear in elements below the statistical power of detection of the cohort, and are therefore made very stringent. The rules operate on GEs that are expected to be depleted of driver mutations, and therefore their most highly desired characteristic is specificity (i.e., the capability to correctly classify benign mutations as passengers). To validate the rule-based approach we classified all SNVs as above solely by the rules. As expected, a lower proportion of SNVs are classified as drivers by rules, 2412 (11%), still maintaining a high proportion of the known driver mutations correctly classified, 805 (78%) and all 12 benign variants classified correctly as passengers.

Since benign SNVs in driver GEs were very scarce in the PCAWG cohort, we carried out two more benchmarks to assess the specificity of the rules. We applied the rules to all benign SNVs in ClinVar[147] (using the version of the rules adapted to group 1 of mutations; see section 8.5). We found that 97.1% of the 314 SNVs were correctly nominated as passengers. Finally, we did the same experiment for a list of common coding polymorphisms obtained from ExAC[87] by filtering out those observed in TCGA tumours, or with allele frequencies below 1% or above 50%. OnCohortDrive correctly identified 98.4% of the resulting 1394 SNVs as passengers.

We carried out a specific sanity check of the PCAWG catalog of driver point mutations produced by onCohortDrive. Namely, we compared the distribution of the number of driver coding point mutations identified across the samples of each cohort within PCAWG with the number of driver mutations estimated to be in excess over the expected (that is, the number of all potential driver mutations) across the same samples. We estimated the distribution of the number of non-silent coding mutations in the genes in our Compendium of Driver Elements across the samples of each cohort using the recently published dNdScv approach. We reasoned that the number of driver point mutations identified by onCohortDrive in each tumour of the cohort (resulting from adding known, driver-by-rank and driver-by-rule mutations identified in each gene) should be very similar to that estimated globally using the dNdScv approach. The comparison of both distributions indeed shows a remarkable agreement between the mean number of driver point mutations estimated in both ways. Note that the mean number of driver point mutations identified by onCohortDrive in the samples of each cohort is in the vast majority of cases within the confidence intervals of the mean number of non-silent coding point mutations in excess computed by the dNdScv.

In summary, this benchmark demonstrated that the decision-making process implemented within onCohortDrive via the ranking and rules-decision approaches possesses high accuracy in the classification of mutations in driver GEs as drivers and passengers.

## 8.6. Processing exceptional GEs or mutations

Some exceptional GEs and types of mutations require some particularities in their analysis by onCohortDrive. Such particularities are described in this section.

#### *8.6.1. Mutation in intronic splice sites*

OnCohortDrive nominates as driver-by-rule mutations all intronic SNVs and indels affecting a splice acceptor region or a splice donor region in a loss-of-function coding gene. Only mutations affecting the canonical acceptor or donor sites and predicted by the LOFTEE to be high-confidence loss-of-function are processed. The known mode of action of cancer genes is respected for this analysis; for GEs with unknown mode of action, we applied the 20/20 rule proposed by Vogelstein[148].

#### *8.6.2. Indels and MNVs*

Indels and MNVs are processed through ranking or rules, like SNVs. The excess of indels was also calculated via NBR. Similar to SNVs, indels falling in GEs with excess undergo a process of ranking, otherwise they follow a rule-based approach.

The CADD score of observed indels was computed as the maximum CADD score of all substitutions comprised by the sequence either inserted of deleted. The consequence type of indels was obtained using the Ensembl Variant Effect Predictor.

Indels in coding GEs are ranked based on their CADD score. If the gene is a loss-of-function driver and the indel causes a frameshift, it is promoted to the top of the ranking, respecting the order introduced by the CADD score among them if more than one is observed. On the other hand, if the gene is an activating driver, mutations causing a frame-shift mutation are moved to the bottom of the rank, unless the gene is NOTCH1 or NOTCH2 (see above). Indels in non-coding GEs are ranked based solely on their CADD scores.

For indels processed through rules, the same probability groups and level of evidence as in SNVs are used (section 8.3.5). Specifically, indels in coding genes of the appropriate groups of evidence are nominated as driver-by-rule if:

a) it causes a frameshift in a loss-of-function gene, or

b) it causes an in-frame mutation with CADD greater than 25.

Indels in non-coding GEs are deemed driver-by-rule if:

a) they appear in the group with the lowest probability of occurrence and have a CADD score above 25,

b) they appear in the group with intermediate probability of occurrence and have a CADD score above 30,

c) they appear in the group with the highest probability of occurrence and have a CADD score above 35

## 8.6. Unbiased calculation of the contribution of noncoding driver mutations

To carry out an unbiased (i.e., without the over-representation of coding mutations introduced by the prior knowledge) calculation of the contribution of non-coding mutations to tumourigenesis, we applied onCohortDrive only to discovery GEs as described above with

one major change. The rank-and-cut approach was implemented on the mutations affecting these elements without first nominating known driver mutations among them. In other words, instead of carrying out the ranking on *N-k* mutations (as described in the Step 1 of section 8.4 above), we did it on all *N* mutations observed in the GE across the cohort. The rule-based approach in this case is not applied to any mutations once the excess is exhausted.

## 8.7. Identification of driver SCNAs and SGRs in tumours of the PCAWG cohort

This section describes the designation of driver SCNAs and SGRs in the PCAWG cohort.

### *8.7.1. Creating the Compendium of driver SCNA elements*

GISTIC[113] peaks found to be significant from the analysis of cohorts of 23 tumour types and 5 metacohorts combining tumour types (**Supplementary Table 16**) analysed by TCGA were first retrieved. The strategy to define the region of the relevant peaks is based on associating overlapping peaks across different cancers into "metapeaks", and if possible associating each metapeak with a driver gene that provides a consistent location at which to assess the DNA copy number. The definition of the footprints of peaks (their 95% confidence intervals window) within a cohort was carried out using an arm-level peel-off method, and the peaks were then extended to other diseases using the 'classic' peel-off approach. In addition, the classic peel-off method was applied to peaks detected across a pan-cancer analysis comprising some 11,000 TCGA tumours[113]. Peaks with more than 25 genes were subsequently discarded.

Seed peak footprints were sorted, with the pan-cancer peaks ranking first, and the cancer specific peaks sorted following the size of their footprint (from smallest to biggest). Following this order, seed peaks were then tested one at a time for overlap with all other seed peak footprints, and overlapping peaks were greedily aggregated into meta-peaks using this ordering. Metapeaks were thus constructed, following the rule that each could not contain more than one peak from the same cancer type. On detail, groups of overlapping metapeaks were optimised so that peaks were assigned to the closest metapeak, and classic peel-off peaks were added to the metapeaks if their disease was not already represented in the metapeak by a seed peak.

The metapeaks resulting from this merging process then underwent a manual curation step. On detail, metapeaks containing more than one somatic SCNA driver gene were split into separate metapeaks for each driver to make sure that the copy number at the locus of each driver was assessed independently. The names of metapeaks were homogenised, with member peaks losing their original identity, and making sure that all seed peaks were assigned to at least one metapeak. This manual curation process respected that merged peaks still had only one peak per cancer type or combined type. Seed peaks were preferred over extension peaks in the merging process.

Finally, we filtered these curated metapeaks using the shift in expression observed for the drivers contained in each metapeak. In the TCGA cohort of tumours (**Supplementary Table 16**) where a metapeak had been found significant, we separated the samples into those showing a copy number change coherent with the identified metapeak and those with normal copy number. Then, we compare the expression of the driver gene within the metapeak in both sets of samples. The metapeak was subsequently annotated as a driver only if a

significant shift in expression coherent with the type of SCNA, amplification or deletion, was observed. In the case of metapeaks containing no known driver gene, we compared the expression of all the genes within the metapeak in both sets of samples, and kept the metapeak in the Compendium only if at least one gene exhibited a significant expression shift.

We added to the Compendium a known highly recurrent deletion spanning the cytoband 14 of 13q chromosomal arm in CLL[137], because no CLL cohort had been analysed by TCGA. To define the boundaries of this peak, we aligned the deleted segments overlapping the area in the tumours of the aforementioned cohort and located the region covered in more than 75% of the tumours.

### *8.7.2. Identification of driver CNA events in PCAWG tumours*

First, for each tumour $t$ in the PCAWG, the relative copy number of segment $s$ ($rCN_{s,t}$) was computed as:

$$rCN_{s,t} = \frac{2CN_{s.t}}{mCN_t} - 2$$

where $CN_{s,t}$is the absolute copy number of $s$ in $t$, and $mCN_t$ is the median copy number of tumour $t$. Finally, we assessed the copy number of the locus of each metapeak in tumours of the same malignancies where the metapeak was discovered. The copy number of a tumour at the locus of a peak was computed as the minimum copy number value across the peak locus. A relative copy number of the peak greater than 0 is considered an amplification at amplification peaks, whereas a value greater than 1 is deemed a high-level amplification. On the other hand, a relative copy number below 0 is considered a loss at deletion peaks, and values below -1 are nominated as high-level deletions. This assessment is carried out on segments of the genome that are shorter that one chromosomal arm, so that only focal events are taken into account.

### *8.7.3. Additional driver SCNA events in PCAWG tumours*

We manually identified additional driver SCNA events in tumours of cohorts contributed by published ICGC studies, and not included in the analysis summarized in Extended Data Table 19, under the rationale that these cancer types, with no GISTIC analysis available were biased against in the Compendium. Specifically, in the CNS-Medullo cohort, we manually included all amplifications of MYC, MYCN, TERT, CCND2, CDK6, and GFI1B, and the deletions of PTCH1, TSC1, and PTEN identified in the original study[114]. These SCNAs were found in 19 PCAWG medulloblastomas. Deletions of CDKN2A and PRDM1 identified in one Lymph-BNHL sample were also added, together with a deletion of BRCA2 in a Panc-Endocrine sample and ATM in a Prost-AdenoCa based on the PCAWG CNA data, but were missed in the above analysis.

### *8.7.4. Identification of driver somatic genomic rearrangements in PCAWG tumours*

The driver SGRs we considered included genic fusions involving an oncogene, truncation of tumour suppressors, and cis-activating rearrangements (e.g., promoter-rearrangement and enhancer hijacking). These events were obtained from literature reports, curated databases (Cancer Gene Census[130]), and a set of high-confidence novel genomic rearrangements that were identified in the PCAWG cohorts (provided by PCAWG Structural Variants analysis working group). Using this information, each tumour within PCAWG was probed for the

presence of driver rearrangements. In the case of gene fusions, the gene coordinates plus a 50-kb flanking window on either side of each member of the pair of fused genes were scanned for the presence of rearrangements. Furthermore, the rearrangements were annotated when they produced a sense in-frame fusion.

This resulted in 331 fusion events in 319 samples. For 214 of these events (in 204 samples) we could not find the expression evidence (i.e., expression of fusion transcripts) due to the lack of expression data (RNA-seq) available for those samples. However, for the 117 events (in 115 samples) with expression data available, we identified 41 events (in 40 samples) that have fusion transcript match, based on the results provided by PCAWG transcriptomics group (syn10003873). For the remaining 76 fusion events (in 76 samples), the lack of fusion transcript match may be explained by promoter/enhancer hijacking events that resulted in an over-expression of the target oncogene. The majority of the fusion events that fall under this category are related to the fusion with IGH/IGL locus. On the other hand, we have included fusion events in seven samples based on the evidence from fusion transcripts, but were not detected based on the aforementioned SV analysis. In addition, we have included four fusion events in nine samples of CNS-PiloAstro samples, based on a previous study[140].

In the case of tumour suppressor genes, the breakpoints affecting exons were considered as drivers. In addition, we analyzed rearrangements affecting the cis-regulatory elements of the coding genes in the compendium. This included rearrangements in the promoter regions (promoter-SV) and those causing a juxtaposition of enhancers close to a gene (enhancer-hijacking). In the latter case, we focused on genes that CESAM[149] analysis has shown to become over-expressed through the enhancer-hijacking process, which takes into account the breakpoints, SCNAs, target gene (mRNA) expression, and chromatin interaction data from topologically associating domains (TADs). For those genes, we performed CESAM analysis to identify PCAWG samples with genes that showed over-expression.

## 8.8. Identification of likely tumorigenic germline variants

We identified all truncating (stop gain, frameshift, splice site) germline point mutations and rare germline SV deletions affecting genes within a cancer susceptibility list[150]. We also identified all truncating germline point mutations and SV deletions affecting genes involved in DNA repair[151], given that a second inactivating event, either somatic (truncating or missense) or germline (truncating) was observed in the other allele.

## 8.9. Identification of biallelic driver events

To identify tumour suppressor two-hit events[152], we defined biallelic inactivation as a gene locus $G^{A/B}$, where alleles A and B are genetically altered, leading to a genetic $G^{mut/mut}$ state. The biallelic inactivation assessment includes three genetic inactivation event types consisting of somatic or germline deletions (“Loss”), somatic or germline SVs (“Break”) and somatic or germline SNVs (“Mutation”). Given a heterozygous GA/B locus, we required a loss of the A allele of the gene, leading to a hemizygous $G^{-/B}$ state, and genetic inactivation of the remaining B allele, specifically requiring the second event to overlap the loss on the A allele, leading to biallelic inactivation. We considered four classes of biallelic inactivations: i) Loss/Mutation, nonsynonymous driver mutations of the B allele; ii) Loss/Loss, two deletion events that overlap an exon and the copy-number derived allele count is 0 both for A and B

allele; iii) Loss/Break, SVs where one or both breakpoints are situated in an exon of the B allele; and iv) Mutation/Mutation, a nonsynonymous germline SNV and a nonsynonymous driver somatic SNV of the same gene. We infer the germline mutation to occur on the A allele and the somatic mutation on the B allele, with the assumption that two independent driver mutation events are highly unlikely to occur on the same allele. All biallelic inactivation events involving at least one Loss event which had not been detected in the process of identification of driver SCNAs –either because the SCNA GE was under the statistical power of detection in the corresponding cohort, failed the expression filter, or because the Loss event involved an arm-level deletion (see section 8.7)– were included in the panorama as driver SCNA loss events.

# 9. Literature Cited


1. Ewing, A. D. *et al.* Combining tumour genome simulation with crowdsourcing to benchmark somatic single-nucleotide-variant detection. *Nat. Methods* **12**, 623–630 (2015).

2. GmbH, R. D. *HOWTO Evaluate SeqCap EZ Target Enrichment Data*. (2014).

3. McKenna, A. *et al.* The Genome Analysis Toolkit: a MapReduce framework for analyzing next-generation DNA sequencing data. *Genome Res.* **20**, 1297–1303 (2010).

4. Cho, Y. *et al.* Development of the variant calling algorithm, ADIScan, and its use to estimate discordant sequences between monozygotic twins. *Nucleic Acids Res.* **46**, e92 (2018).

5. Kumar, Y. *et al.* Massive interstitial copy-neutral loss-of-heterozygosity as evidence for cancer being a disease of the DNA-damage response. *BMC Med. Genomics* **8**, 42 (2015).

6. Cibulskis, K. *et al.* Sensitive detection of somatic point mutations in impure and heterogeneous cancer samples. *Nat. Biotechnol.* **31**, 213–219 (2013).

7. Radenbaugh, A. J. *et al.* RADIA: RNA and DNA integrated analysis for somatic mutation detection. *PLoS One* **9**, e111516 (2014).

8. Saunders, C. T. *et al.* Strelka: accurate somatic small-variant calling from sequenced tumour-normal sample pairs. *Bioinformatics* **28**, 1811–1817 (2012).

9. Larson, D. E. *et al.* SomaticSniper: identification of somatic point mutations in whole genome sequencing data. *Bioinformatics* **28**, 311–317 (2012).

10. Sherry, S. T. *et al.* dbSNP: the NCBI database of genetic variation. *Nucleic Acids Res.* **29**, 308–311 (2001).

11. Ouedraogo, M. *et al.* The duplicated genes database: identification and functional annotation of co-localised duplicated genes across genomes. *PLoS One* **7**, e50653 (2012).

12. Gerstein, M. B. *et al.* Architecture of the human regulatory network derived from ENCODE data. *Nature* **489**, 91–100 (2012).

13. Fuentes Fajardo, K. V. *et al.* Detecting false-positive signals in exome sequencing. *Hum. Mutat.* **33**, 609–613 (2012).

14. Forbes, S. A. *et al.* COSMIC: exploring the world's knowledge of somatic mutations in human cancer. *Nucleic Acids Res.* **43**, D805–11 (2015).

15. Garrison, E. & Marth, G. Haplotype-based variant detection from short-read sequencing. *arXiv [q-bio.GN]* (2012).

16. Koboldt, D. C. *et al.* VarScan 2: somatic mutation and copy number alteration discovery in cancer by exome sequencing. *Genome Res.* **22**, 568–576 (2012).

17. Ye, K., Schulz, M. H., Long, Q., Apweiler, R. & Ning, Z. Pindel: a pattern growth approach to detect break points of large deletions and medium sized insertions from paired-end short reads. *Bioinformatics* **25**, 2865–2871 (2009).

18. Raine, K. M. *et al.* cgpPindel: Identifying Somatically Acquired Insertion and Deletion Events from Paired End Sequencing. *Curr. Protoc. Bioinformatics* **52**, 15.7.1–12 (2015).

19. Ossowski, S. *et al.* Sequencing of natural strains of Arabidopsis thaliana with short reads. *Genome Res.* **18**, 2024–2033 (2008).

20. Cao, J. *et al.* Whole-genome sequencing of multiple Arabidopsis thaliana populations. *Nat. Genet.* **43**, 956–963 (2011).

21. Chong, Z. *et al.* novoBreak: local assembly for breakpoint detection in cancer genomes. *Nat. Methods* **14**, 65–67 (2017).

22. Li, H. Aligning sequence reads, clone sequences and assembly contigs with BWA-MEM. *arXiv [q-bio.GN]* (2013).

23. DePristo, M. A. *et al.* A framework for variation discovery and genotyping using next-generation DNA sequencing data. *Nat. Genet.* **43**, 491–498 (2011).

24. Van der Auwera, G. A. *et al.* From FastQ data to high confidence variant calls: the Genome Analysis Toolkit best practices pipeline. *Curr. Protoc. Bioinformatics* **43**, 11.10.1–33 (2013).

25. Li, H. *et al.* The Sequence Alignment/Map format and SAMtools. *Bioinformatics* **25**, 2078–2079 (2009).

26. Danecek, P., Schiffels, S. & Durbin, R. Multiallelic calling model in bcftools (-m). (2016).

27. Karolchik, D. *et al.* The UCSC Genome Browser Database. *Nucleic Acids Res.* **31**, 51–54 (2003).

28. Rimmer, A. *et al.* Integrating mapping-, assembly- and haplotype-based approaches for calling variants in clinical sequencing applications. *Nature Genetics* **46**, 912–918 (2014).

29. Rausch, T. *et al.* DELLY: structural variant discovery by integrated paired-end and split-read analysis. *Bioinformatics* **28**, i333–i339 (2012).

30. Kleinheinz, K. *et al.* ACEseq - allele specific copy number estimation from whole genome sequencing. *bioRxiv* 210807 (2017). doi:10.1101/210807

31. Howie, B. N., Donnelly, P. & Marchini, J. A flexible and accurate genotype imputation method for the next generation of genome-wide association studies. *PLoS Genet.* **5**, e1000529 (2009).

32. Olshen, A. B., Venkatraman, E. S., Lucito, R. & Wigler, M. Circular binary segmentation for the analysis of array-based DNA copy number data. *Biostatistics* **5**, 557–572 (2004).

33. ACEseq - Allele-specific copy numbers from sequencing — ACEseqDocs 1.2.8 documentation. Available at: http://aceseq.readthedocs.io/en/latest/index.html. (Accessed: 8th July 2019)

34. Jones, D. *et al.* cgpCaVEManWrapper: Simple Execution of CaVEMan in Order to Detect Somatic Single Nucleotide Variants in NGS Data. *Curr. Protoc. Bioinformatics* **56**, 15.10.1–15.10.18 (2016).

35. CaVEMan. Unpublished software. Source code and documentation available at GitHub. *GitHub* Available at: https://github.com/cancerit/CaVEMan/.

36. BRASS. Unpublished software. Source code and documentation available at GitHub. *GitHub* Available at: https://github.com/cancerit/BRASS.

37. GRASS. Unpublished software. Source code and documentation available at GitHub. *GitHub* Available at: https://github.com/cancerit/grass.

38. Raine, K. M. *et al.* ascatNgs: Identifying Somatically Acquired Copy-Number Alterations from Whole-Genome Sequencing Data. *Curr. Protoc. Bioinformatics* **56**, 15.9.1–15.9.17 (2016).

39. Cibulskis, K. *et al.* ContEst: estimating cross-contamination of human samples in next-generation sequencing data. *Bioinformatics* **27**, 2601–2602 (2011).

40. Costello, M. *et al.* Discovery and characterization of artifactual mutations in deep coverage targeted capture sequencing data due to oxidative DNA damage during sample preparation. *Nucleic Acids Research* **41**, e67–e67 (2013).

41. Wala, J. A. *et al.* SvABA: genome-wide detection of structural variants and indels by local assembly. *Genome Research* **28**, 581–591 (2018).

42. Fan, Y. *et al.* MuSE: accounting for tumour heterogeneity using a sample-specific error model improves sensitivity and specificity in mutation calling from sequencing data. *Genome Biol.* **17**, 178 (2016).

43. Li, H. & Durbin, R. Fast and accurate long-read alignment with Burrows–Wheeler transform. *Bioinformatics* **26**, 589–595 (2010).

44. Moncunill, V. *et al.* Comprehensive characterization of complex structural variations in cancer by directly comparing genome sequence reads. *Nat. Biotechnol.* **32**, 1106–1112 (2014).

45. Menzies, A. *et al.* VAGrENT: Variation Annotation Generator. *Curr. Protoc. Bioinformatics* **52**, 15.8.1–11 (2015).

46. Wang, K., Li, M. & Hakonarson, H. ANNOVAR: functional annotation of genetic variants from high-throughput sequencing data. *Nucleic Acids Res.* **38**, e164 (2010).

47. Dockstore. Available at: https://dockstore.org/containers/registry.hub.docker.com/weischenfeldt/pcawg_sv_merge:1.0.2?tab=info. (Accessed: 11th May 2019)

48. Kim, S. Y., Jacob, L. & Speed, T. P. Combining calls from multiple somatic mutation-callers. *BMC Bioinformatics* **15**, 154 (2014).

49. Li, Y. *et al.* Patterns of somatic structural variation in human cancer genomes. *Nature* (2019).

50. Taylor-Weiner, A. *et al.* DeTiN: overcoming tumour-in-normal contamination. *Nat. Methods* **15**, 531–534 (2018).

51. *DKFZBiasFilter*. (Github).

52. Tian, S., Yan, H., Kalmbach, M. & Slager, S. L. Impact of post-alignment processing in variant discovery from whole exome data. *BMC Bioinformatics* **17**, 403 (2016).

53. Alexandrov, L. B. *et al.* The Repertoire of Mutational Signatures in Human Cancer. *Nature* (2019).

54. Wala, J., Zhang, C.-Z., Meyerson, M. & Beroukhim, R. VariantBam: filtering and profiling of next-generational sequencing data using region-specific rules. *Bioinformatics* **32**, 2029–2031 (2016).

55. Mills, R. E. *et al.* An initial map of insertion and deletion (INDEL) variation in the human genome. *Genome Res.* **16**, 1182–1190 (2006).

56. Thorisson, G. A., Smith, A. V., Krishnan, L. & Stein, L. D. The International HapMap Project Web site. *Genome Res.* **15**, 1592–1593 (2005).

57. Manichaikul, A. *et al.* Robust relationship inference in genome-wide association studies. *Bioinformatics* **26**, 2867–2873 (2010).

58. Tan, A., Abecasis, G. R. & Kang, H. M. Unified representation of genetic variants. *Bioinformatics* **31**, 2202–2204 (2015).

59. Yakneen, S., Waszak, S. M., Gertz, M. & Korbel, J. O. Enabling rapid cloud-based analysis of thousands of human genomes via Butler. *Nat. Biotechnol.* (2019).

60. Cleary, J. G. *et al.* Joint variant and de novo mutation identification on pedigrees from high-throughput sequencing data. *J. Comput. Biol.* **21**, 405–419 (2014).

61. Shringarpure, S. S., Carroll, A., De La Vega, F. M. & Bustamante, C. D. Inexpensive and Highly Reproducible Cloud-Based Variant Calling of 2,535 Human Genomes. *PLoS One* **10**, e0129277 (2015).

62. Rodriguez-Martin, B. *et al.* Pan-cancer analysis of whole genomes identifies driver rearrangements promoted by LINE-1 retrotransposition. *Nat. Genet.* (2019).

63. Sudmant, P. H. *et al.* An integrated map of structural variation in 2,504 human genomes. *Nature* **526**, 75–81 (2015).

64. Hehir-Kwa, J. Y. *et al.* A high-quality human reference panel reveals the complexity and distribution of genomic structural variants. *Nat. Commun.* **7**, 12989 (2016).

65. Simons, Y. B., Turchin, M. C., Pritchard, J. K. & Sella, G. The deleterious mutation load is insensitive to recent population history. *Nat. Genet.* **46**, 220–224 (2014).

66. Hancks, D. C. & Kazazian, H. H., Jr. SVA retrotransposons: Evolution and genetic instability. *Semin. Cancer Biol.* **20**, 234–245 (2010).

67. Tubio, J. M. C. *et al.* Mobile DNA in cancer. Extensive transduction of nonrepetitive DNA mediated by L1 retrotransposition in cancer genomes. *Science* **345**, 1251343 (2014).

68. Thorvaldsdóttir, H., Robinson, J. T. & Mesirov, J. P. Integrative Genomics Viewer (IGV): high-performance genomics data visualization and exploration. *Brief. Bioinform.* **14**, 178–192 (2013).

69. Nelli, F. Machine Learning with scikit-learn. *Python Data Analytics* 237–264 (2015). doi:10.1007/978-1-4842-0958-5_8

70. From FastQ Data to High-Confidence Variant Calls: The Genome Analysis Toolkit Best Practices Pipeline. in *Current Protocols in Bioinformatics* (eds. Bateman, A., Pearson, W. R., Stein, L. D., Stormo, G. D. & Yates, J. R., III) **467**, 11.10.1–11.10.33 (John Wiley & Sons, Inc., 2002).

71. 1000 Genomes Project Consortium *et al.* A global reference for human genetic variation. *Nature* **526**, 68–74 (2015).

72. Mallick, S. *et al.* The Simons Genome Diversity Project: 300 genomes from 142 diverse populations. *Nature* **538**, 201–206 (2016).

73. Fu, Q. *et al.* The genetic history of Ice Age Europe. *Nature* **534**, 200–205 (2016).

74. Li, H. Minimap2: pairwise alignment for nucleotide sequences. *Bioinformatics* **34**, 3094–3100 (2018).

75. Rausch, T., Fritz, M. H.-Y., Korbel, J. O. & Benes, V. Alfred: Interactive multi-sample BAM alignment statistics, feature counting and feature annotation for long- and short-read sequencing. *Bioinformatics* (2018). doi:10.1093/bioinformatics/bty1007

76. Lander, E. S. & Waterman, M. S. Genomic mapping by fingerprinting random clones: a mathematical analysis. *Genomics* **2**, 231–239 (1988).

77. Kurtz, S. *et al.* Versatile and open software for comparing large genomes. *Genome Biol.* **5**, R12 (2004).

78. Loh, P.-R. *et al.* Reference-based phasing using the Haplotype Reference Consortium panel. *Nat. Genet.* **48**, 1443–1448 (2016).

79. Delaneau, O., Marchini, J. & Zagury, J.-F. A linear complexity phasing method for thousands of genomes. *Nat. Methods* **9**, 179–181 (2011).

80. Danecek, P. *et al.* The variant call format and VCFtools. *Bioinformatics* **27**, 2156–2158 (2011).

81. Menelaou, A. & Marchini, J. Genotype calling and phasing using next-generation sequencing reads and a haplotype scaffold. *Bioinformatics* **29**, 84–91 (2013).

82. Alexander, D. H., Novembre, J. & Lange, K. Fast model-based estimation of ancestry in unrelated individuals. *Genome Res.* **19**, 1655–1664 (2009).

83. Wojcik, G. L. *et al.* Imputation-Aware Tag SNP Selection To Improve Power for Large-Scale, Multi-ethnic Association Studies. *G3* **8**, 3255–3267 (2018).

84. Purcell, S. *et al.* PLINK: a tool set for whole-genome association and population-based linkage analyses. *Am. J. Hum. Genet.* **81**, 559–575 (2007).

85. Maples, B. K., Gravel, S., Kenny, E. E. & Bustamante, C. D. RFMix: a discriminative modeling approach for rapid and robust local-ancestry inference. *Am. J. Hum. Genet.* **93**, 278–288 (2013).

86. Martin, A. R. *et al.* Human Demographic History Impacts Genetic Risk Prediction across Diverse Populations. *Am. J. Hum. Genet.* **100**, 635–649 (2017).

87. Lek, M. *et al.* Analysis of protein-coding genetic variation in 60,706 humans. *Nature* **536**, 285–291 (2016).

88. Tennessen, J. A. *et al.* Evolution and functional impact of rare coding variation from deep sequencing of human exomes. *Science* **337**, 64–69 (2012).

89. Roberts, S. A. & Gordenin, D. A. Hypermutation in human cancer genomes: footprints and mechanisms. *Nat. Rev. Cancer* **14**, 786–800 (2014).

90. Chan, K. *et al.* An APOBEC3A hypermutation signature is distinguishable from the signature of background mutagenesis by APOBEC3B in human cancers. *Nat. Genet.* **47**, 1067–1072 (2015).

91. Saini, N. *et al.* The Impact of Environmental and Endogenous Damage on Somatic Mutation Load in Human Skin Fibroblasts. *PLoS Genet.* **12**, e1006385 (2016).

92. Dobin, A. *et al.* STAR: ultrafast universal RNA-seq aligner. *Bioinformatics* **29**, 15–21 (2013).

93. Kim, D. *et al.* TopHat2: accurate alignment of transcriptomes in the presence of insertions, deletions and gene fusions. *Genome Biol.* **14**, R36 (2013).

94. PCAWG Transcriptome Core Group *et al.* Genomic basis for RNA alterations revealed by whole-genome analyses of 27 cancer types. *Nature* (2019).

95. Anders, S., Pyl, P. T. & Huber, W. HTSeq--a Python framework to work with high-throughput sequencing data. *Bioinformatics* **31**, 166–169 (2015).

96. Bray, N. L., Pimentel, H., Melsted, P. & Pachter, L. Near-optimal probabilistic RNA-seq quantification. *Nat. Biotechnol.* **34**, 525–527 (2016).

97. Kahles, A., Ong, C. S., Zhong, Y. & Rätsch, G. SplAdder: identification, quantification and testing of alternative splicing events from RNA-Seq data. *Bioinformatics* **32**, 1840–1847 (2016).

98. Ge, H. *et al.* FusionMap: detecting fusion genes from next-generation sequencing data at base-pair resolution. *Bioinformatics* **27**, 1922–1928 (2011).

99. Nicorici, D. *et al.* FusionCatcher – a tool for finding somatic fusion genes in paired-end RNA-sequencing data. *bioRxiv* 011650 (2014). doi:10.1101/011650

100. Sieverling, L. *et al.* Genomic footprints of activated telomere maintenance mechanisms in cancer. *Genomics* (2017).

101. Braun, D. M., Chung, I., Kepper, N., Deeg, K. I. & Rippe, K. TelNet - a database for human and yeast genes involved in telomere maintenance. *BMC Genet.* **19**, 32 (2018).

102. Cortes-Ciriano, I. *et al.* Comprehensive analysis of chromothripsis in 2,658 human cancers using whole-genome sequencing. *Genomics* (2018).

103. Stephens, P. J. *et al.* Massive Genomic Rearrangement Acquired in a Single Catastrophic Event during Cancer Development. *Cell* **144**, 27–40 (2011).

104. Kinsella, M., Patel, A. & Bafna, V. The elusive evidence for chromothripsis. *Nucleic Acids Res.* **42**, 8231–8242 (2014).

105. Korbel, J. O. & Campbell, P. J. Criteria for inference of chromothripsis in cancer genomes. *Cell* **152**, 1226–1236 (2013).

106. Chan, K. & Gordenin, D. A. Clusters of Multiple Mutations: Incidence and Molecular Mechanisms. *Annu. Rev. Genet.* **49**, 243–267 (2015).

107. Gerstung, M. *et al.* The evolutionary history of 2,658 cancers. *Nature* (2019).

108. Petryk, N. *et al.* Replication landscape of the human genome. *Nat. Commun.* **7**, 10208 (2016).

109. Hansen, R. S. *et al.* Sequencing newly replicated DNA reveals widespread plasticity in human replication timing. *Proc. Natl. Acad. Sci. U. S. A.* **107**, 139–144 (2010).

110. Dentro, S. C. *et al.* Portraits of genetic intra-tumour heterogeneity and subclonal selection across cancer types. doi:10.1101/312041

111. Rheinbay, E. *et al.* On the discovery of somatic driver events in >2,500 whole cancer genomes. *Nature* (2019).

112. Taylor-Weiner, A. *et al.* DeTiN: overcoming tumour-in-normal contamination. *Nat. Methods* **15**, 531–534 (2018).

113. Mermel, C. H. *et al.* GISTIC2.0 facilitates sensitive and confident localization of the targets of focal somatic copy-number alteration in human cancers. *Genome Biol.* **12**, R41 (2011).

114. Northcott, P. A. *et al.* The whole-genome landscape of medulloblastoma subtypes. *Nature* **547**, 311–317 (2017).

115. Lawrence, M. S. *et al.* Mutational heterogeneity in cancer and the search for new cancer-associated genes. *Nature* **499**, 214–218 (2013).

116. Dees, N. D. *et al.* MuSiC: identifying mutational significance in cancer genomes. *Genome Res.* **22**, 1589–1598 (2012).

117. Gonzalez-Perez, A. & Lopez-Bigas, N. Abstract LB-401: Functional impact bias reveals cancer drivers. *Molecular and Cellular Biology* (2012). doi:10.1158/1538-7445.am2012-lb-401

118. Mularoni, L., Sabarinathan, R., Deu-Pons, J., Gonzalez-Perez, A. & López-Bigas, N. OncodriveFML: a general framework to identify coding and non-coding regions with cancer driver mutations. *Genome Biol.* **17**, 128 (2016).

119. Weinhold, N., Jacobsen, A., Schultz, N., Sander, C. & Lee, W. Genome-wide analysis of noncoding regulatory mutations in cancer. *Nat. Genet.* **46**, 1160–1165 (2014).

120. Juul, M. *et al.* Non-coding cancer driver candidates identified with a sample- and position-specific model of the somatic mutation rate. *Elife* **6**, (2017).

121. Reimand, J. & Bader, G. D. Systematic analysis of somatic mutations in phosphorylation signaling predicts novel cancer drivers. *Mol. Syst. Biol.* **9**, 637 (2013).

122. Lanzos, A. *et al.* Discovery of Cancer Driver Long Noncoding RNAs across 1112 Tumour Genomes: New Candidates and Distinguishing Features. doi:10.1101/065805

123. Dogruluk, T. *et al.* Identification of Variant-Specific Functions of PIK3CA by Rapid Phenotyping of Rare Mutations. *Cancer Res.* **75**, 5341–5354 (2015).

124. Kim, E. *et al.* Systematic Functional Interrogation of Rare Cancer Variants Identifies Oncogenic Alleles. *Cancer Discov.* **6**, 714–726 (2016).

125. Lawrence, M. S. *et al.* Discovery and saturation analysis of cancer genes across 21 tumour types. *Nature* **505**, 495–501 (2014).

126. Kircher, M. *et al.* A general framework for estimating the relative pathogenicity of human genetic variants. *Nat. Genet.* **46**, 310–315 (2014).

127. Tamborero, D. *et al.* Cancer Genome Interpreter annotates the biological and clinical relevance of tumour alterations. *Genome Med.* **10**, 25 (2018).

128. Martincorena, I. *et al.* Universal Patterns of Selection in Cancer and Somatic Tissues. *Cell* **171**, 1029–1041.e21 (2017).

129. Nik-Zainal, S. *et al.* Landscape of somatic mutations in 560 breast cancer whole-genome sequences. *Nature* **534**, 47–54 (2016).

130. Futreal, P. A. *et al.* A census of human cancer genes. *Nat. Rev. Cancer* **4**, 177–183 (2004).

131. Gonzalez-Perez, A. *et al.* IntOGen-mutations identifies cancer drivers across tumour types. *Nat. Methods* **10**, 1081–1082 (2013).

132. Rubio-Perez, C. *et al.* In silico prescription of anticancer drugs to cohorts of 28 tumour types reveals targeting opportunities. *Cancer Cell* **27**, 382–396 (2015).

133. Huang, F. W. *et al.* Highly recurrent TERT promoter mutations in human melanoma. *Science* **339**, 957–959 (2013).

134. Killela, P. J. *et al.* TERT promoter mutations occur frequently in gliomas and a subset of tumours derived from cells with low rates of self-renewal. *Proc. Natl. Acad. Sci. U. S. A.* **110**, 6021–6026 (2013).

135. Horn, S. *et al.* TERT promoter mutations in familial and sporadic melanoma. *Science* **339**, 959–961 (2013).

136. Rheinbay, E. *et al.* Recurrent and functional regulatory mutations in breast cancer. *Nature* **547**, 55–60 (2017).

137. Puente, X. S. *et al.* Non-coding recurrent mutations in chronic lymphocytic leukaemia. *Nature* **526**, 519–524 (2015).

138. Church, D. N. *et al.* DNA polymerase ε and δ exonuclease domain mutations in endometrial cancer. *Hum. Mol. Genet.* **22**, 2820–2828 (2013).

139. Cancer Genome Atlas Research Network *et al.* Integrated genomic characterization of endometrial carcinoma. *Nature* **497**, 67–73 (2013).

140. Jones, D. T. W. *et al.* Recurrent somatic alterations of FGFR1 and NTRK2 in pilocytic astrocytoma. *Nat. Genet.* **45**, 927–932 (2013).

141. Reva, B., Antipin, Y. & Sander, C. Predicting the functional impact of protein mutations: application to cancer genomics. *Nucleic Acids Res.* **39**, e118 (2011).

142. González-Pérez, A. & López-Bigas, N. Improving the assessment of the outcome of nonsynonymous SNVs with a consensus deleteriousness score, Condel. *Am. J. Hum. Genet.* **88**, 440–449 (2011).

143. Tamborero, D., Gonzalez-Perez, A. & Lopez-Bigas, N. OncodriveCLUST: exploiting the positional clustering of somatic mutations to identify cancer genes. *Bioinformatics* **29**, 2238–2244 (2013).

144. Sabarinathan, R., Mularoni, L., Deu-Pons, J., Gonzalez-Perez, A. & López-Bigas, N. Nucleotide excision repair is impaired by binding of transcription factors to DNA. *Nature* **532**, 264–267 (2016).

145. Hong, Y. On computing the distribution function for the Poisson binomial distribution. *Comput. Stat. Data Anal.* **59**, 41–51 (2013).

146. Schroeder, M. P., Rubio-Perez, C., Tamborero, D., Gonzalez-Perez, A. & Lopez-Bigas, N. OncodriveROLE classifies cancer driver genes in loss of function and activating mode of action. *Bioinformatics* **30**, i549–55 (2014).

147. Landrum, M. J. *et al.* ClinVar: public archive of relationships among sequence variation and human phenotype. *Nucleic Acids Res.* **42**, D980–5 (2014).

148. Vogelstein, B. *et al.* Cancer genome landscapes. *Science* **339**, 1546–1558 (2013).

149. Weischenfeldt, J. *et al.* Pan-cancer analysis of somatic copy-number alterations implicates IRS4 and IGF2 in enhancer hijacking. *Nat. Genet.* **49**, 65–74 (2017).

150. Rahman, N. Realizing the promise of cancer predisposition genes. *Nature* **505**, 302–308 (2014).

151. Pearl, L. H., Schierz, A. C., Ward, S. E., Al-Lazikani, B. & Pearl, F. M. G. Therapeutic opportunities within the DNA damage response. *Nat. Rev. Cancer* **15**, 166–180 (2015).

152. Knudson, A. G., Jr. Mutation and cancer: statistical study of retinoblastoma. *Proc. Natl. Acad. Sci. U. S. A.* **68**, 820–823 (1971).

# Supplementary Notes

## Overview

Previous experience suggests that ensembles of somatic variant callers can robustly provide results that are both more sensitive and more precise than the component callers[1–3]. We describe here the somatic variant calling ensemble pipeline for SNVs and indels, run primarily on the PCAWG cloud infrastructure. An initial validation phase, involving a pilot of 63 samples and 13 pipelines run by individuals within the PCAWG-1 working group, generated candidate somatic variant calls, which were subsequently selected for validation by deep sequencing. Those validation results were then analyzed and consensus strategies were developed, incorporating information from the production pipelines, annotations from the WGS data, genomic context, and external databases.

Production call sets from four SNV callers (MuTect, MuSE, and the Sanger and DKFZ pipelines) and three indel callers (Sanger and DKFZ pipelines, and SvABA) were then generated through the PCAWG cloud infrastructure. The process included uniform alignment, invocation of callers, post-hoc filtering of calls exhibiting OxoG oxidative artifacts[4], and addition of simple read statistics annotations. An additional caller, SMuFin[5], was also run using the same alignments. The deep sequencing validation data were used to assess accuracies and choose consensus calling strategies. The consensus caller was then run on the production output of all tumours. Additional filtering was employed to remove artifacts that were either present in the samples, not seen in great numbers in the validation cases, or that the validation strategy was unable to recognize. Samples that were determined to be contaminated, had undergone presumed sample-swaps, or had clinical data inconsistent with the sequenced data were excluded from further consideration, producing a final result of high-confidence somatic SNV and indel calls across 2,778 cancer whole genomes from 2,658 donors.

## 1. Pilot-63 benchmarking and validation exercise

We selected 63 paired tumour-normal samples that were uniformly aligned to cover a range of cancer types (23) and sequencing projects (26). The samples were selected to represent a diversity of tumour types and to have available biological material for experimental validation. The aligned tumour and control reads for the selected samples were distributed to the 13 participating subgroups of the PCAWG SNV Calling Working Group, each of which ran their own somatic variant pipeline. In order to maximize the list of candidate somatic mutations, the pipelines encompassed a wide range of methods, including established and emerging callers using both alignment and assembly-based strategies. All calls were then collected centrally. The callers are summarized in **Supplementary Table 17** and are described in more detail, along with critical run-time parameters, under **Supplementary Methods S1.** In addition to their SNV and Indel calling functions, the "Broad," "Sanger" and "EMBL/DKFZ" pipelines incorporate methods for calling SVs and SCNAs (see **Supplementary Methods S2** and **Supplementary Table 3**).

These 63 samples demonstrated a wide range of mutation rates, with the total number of unique somatic mutation calls from all callers varying ~1,000-fold for SNVs and ~30-fold for indels (**Supplementary Figure 14A**).

The number of callers that identify a given variant in a given tumour is called the "concordance" of that call. Considering only those 46 samples that were successfully processed by all callers ("common samples"), the number of calls by concordance and caller is shown in **Supplementary Figure 14B**. There is clearly much stronger agreement between SNV calls than Indel calls; 59% of all SNV calls are present in six of the ten callers, whereas only 6.5% of all indel calls are made by a majority of callers, with 74% of all calls being private to one caller; this much lower concordance holds even for very short indels (**Supplementary Figure 15**). Callers make different tradeoffs between sensitivity and precision, which is valuable for an ensemble, as they provide a range of levels of evidence in support of any given call. Agreement among callers tended to be higher (robust linear fit: $p = 0.046$ in SNVs, 0.0012 in indels) in more highly-mutated tumours, as shown in **Supplementary Figure 16.**

We observed that the level of agreement between callers also varies with the variant allele fraction (VAF; **Supplementary Figure 17**) with low and high VAF mutations tending to have lower concordance. Low VAF mutations are naturally more difficult to call since the evidence for the mutation is nearer to the limit of sequencing noise; conversely high VAF mutations should occur rarely as they require a corresponding copy number change so are difficult to distinguish from the far more numerous germline polymorphisms.

## Stratified Mutation Sampling

Once the sets of proposed calls were collected, we sampled approximately 250,000 of the 6.4 million unique somatic short variants for validation by targeted deep sequencing. Sampling a population is a deeply-studied field, with methodological detail even filling textbooks[6]. Here, we wanted precise estimates less for the individual callers and more for the differences or overlaps between the callers on each sample, in order to find ways of using the caller outputs to obtain an optimal combination of the call sets. The concordance of a call directly indicates the number of times that callers differed in making the call. Thus we followed the DREAM somatic mutation challenge[7] strategy of stratifying the sampling by case and concordance, and assigning as equal as possible the number of calls per case across each stratum; this allows for the construction of low variance, bias-free estimators of the differences in accuracies between callers using suitable re-weighted averaging of the validation results. In our case we aimed for 30% of the call budget to be for private (concordance = 1) calls, and the rest evenly distributed among remaining concordance bins.

Fifty cases (spanning the same 23 cancer types but 25 sequencing projects) that still had original source DNA for targeted resequencing were selected for deep-sequencing validation. For each matched sample pair, approximately 3000 SNVs and 2190 indels were selected for validation (40% and 30% of the somatic variant validation budget, respectively; another 30% went to structural variants, described in another publication); proportionally more calls went to the more complex variants as there was much less concordance for them.

## Deep Sequencing

Deep sequencing for validation was performed with Nimblegen Liquid-Phase Capture and Illumina HiSeq sequencing. Samples were split over four arrays, with probes on the array designed for variants selected as described above; Genomic DNA fragments captured with Array 1 were sequenced at the Baylor College of Medicine, and the genomic DNA fragments captured with Arrays 2-4 were sequenced at the Washington University of St. Louis. The first array designed (Array 2) experienced a large number (26.4%) of probe design failures, due to variants being in a low complexity or repetitive region of the genome. The remaining arrays were designed by selecting only variants outside of genomic regions annotated by the RepBase database of RepeatMasker[8], thereby reducing the rate of probe failures significantly (down to 12.3%, 9.5%, 9.9% for arrays 1, 3, and 4 respectively), though at the cost of foregoing information in those regions, approximately 41% of the genome. Results were not sensitive to including or excluding samples on array 2; the by-sample validation rates on array 2 were consistent with those of the other arrays (two-sided Kolmogorov-Smirnov, p = 0.7904).

The results of targeted capture and sequencing were processed following Nimblegen recommended best practices[9], with minor differences described below**.** Median sequencing depth at all candidate mutation sites was 610 in the validation tumour sample, 512 in the normal (**Supplementary Figure 18**). We developed a method to classify each mutation based on comparing the evidence for the variant in the validation-tumour and validation-normal samples (**Supplementary Figure 18B**). Given that the call had already been identified in the WGS data, the validation data were treated as supporting the call if in the tumour sample the alternate-supporting read count was inconsistent with noise, and the read-counts in the normal were more consistent with noise than the counts in the tumour (less a factor of two for possible LOH events). A population of calls with normal and tumour VAFs broadly consistent (denoted "NORMALEVIDENCE") are seen; this suggests that the WGS call was a consistent mapping or sequencing artifact and so the call is rejected. Results are largely insensitive to the location of the cutoff between PASS and NORMALEVIDENCE calls in normal VAF. A very small number of calls are clearly homozygous or heterozygous germline variants. The population of accepted calls ("PASS") are seen in the upper-left of each panel. In the case of indels, these calls "bridge" all the way across to the NORMALEVIDENCE calls, due to the calls often occurring in homopolymers or other simple repeats.

Two of the samples in the validation set were omitted due to their cases having later been excluded due to contamination (estimated tumour-in-normal (TiN) contamination >10%); one of these sample pairs corresponding to donor DO36352 (TiN of 16%) seems to have further underwent a sample swap during validation, with the normal sample being sequenced twice. This unintended technical replicate provides a test of the false-positive rate of our variant classification procedure, as none of the variants in the mislabeled "tumour" sample should occur at significantly different rates than in the normal sample. **Supplementary Figure 18C** shows the classification for this sample. As expected, almost all calls cluster along the line of equality for normal and variant VAF, and the validation false positive rate is under 1% for both variant types (8/821 for SNVs, and 7/2083 for indels).

**Supplementary Figure 18D** shows the validation rate versus concordance for both SNVs and Indels, and the fraction of calls at each concordance level. In both sets, high-concordance and low-concordance calls are very likely to validate as true positives and false positives,

respectively. However, with SNVs, most calls were at moderately high concordance, while indel calls were predominantly low-concordance.

### Accuracy on Validation Samples

Performance statistics (sensitivity, precision, and F1 accuracy) can be calculated for all participating callers on the validation samples by appropriately weighting by the sampling frequencies. We omit performance characterization within RepeatMasker-masked regions because of lack of validation data and because callers made calls within these regions at varying rates. Statistics are shown in **Supplementary Figure 16** with box plots illustrating the distribution of accuracies over samples in **Supplementary Figure 16A**, with a heat map clustered by sample and caller in **Supplementary Figure 16B**, and a sensitivity-recall plot showing overall accuracies in **Supplementary Figure 16C**, with SNV accuracies on the left and indel accuracies on the right.

Accuracies cluster for both sets of calls, with outliers typically being those methods focussed on particular subtypes of calls, *e.g.* LOHcomplete, which specializes in loss-of-heterozygosity events. All SNV callers displayed high precision, but varied in sensitivity, particularly for low-VAF variants. General purpose callers typically performed roughly equally well across samples. Indel call accuracies were however much more varied, both between callers and for a given caller between different samples.

As might be expected from the lower agreement on the low mutation-count tumours, accuracies for individual callers and combinations thereof were generally lower for these quiet tumours.

## 2. Production calling and variant consensus development

Following the Pilot-63 assessment and validation exercise, five pipelines were selected for application to the entire PCAWG data set using the PCAWG cloud infrastructure (see below): the SNV+indel pipelines DKFZ, Sanger, and Broad, and the indel-only pipelines SvABA and SMuFIN. During the production phase of the project, some pipelines were modified to improve scalability or robustness, or to address other issues identified during the validation project (see **Supplementary Methods S2)**. In most cases, the changes were quite small. Specifically, the DKFZ pipeline was modified to improve the selection of bases in overlapping regions of read pairs that are used for SNV calling. Additionally, the upper coverage limit allowed in the control on valid somatic SNV positions was changed from 150X to a dynamic value estimated per sample. The initial MuTect run mistakenly used an inflated estimate of foreign DNA contamination, which was later corrected (**Supplementary Methods S2.2.6)**. The modifications to the production versions of these two pipelines resulted in changes to fewer than 1% of calls in the pilot samples. The Sanger and SMuFIN pipelines were unchanged between the validation process and the production runs.

However, in two cases, the changes were such that the calls changed significantly, and validation results cannot be extrapolated with confidence to the production calls:

For the Broad indel pipeline, the pilot/validation phase indel calls were made with MuTect2, while production calls were made with SvABA (see **Supplementary Methods S2.2.4)**.

There were also changes to the MuSE pipeline. MuSE v0.9 was used to generate calls for the validation set, and only the most confident calls were considered. In MuSE v1.0, run as part of the production pipeline, a sample-specific error model was used in assessing calls, and additional filters both before and after calling were included, and all calls of all tiers of confidence were included (see **Supplementary Methods S2.2.5**).

### *Consensus SNV and Indel Models*

We sought to produce a single call set per sample per variant type that would be used by the downstream PCAWG working groups. Using the validation data and calls from the PCAWG production callers, a number of consensus ensemble models were trained and examined. The desired properties of the ensemble models included transparency, accuracy, and robustness of accuracy. Specifically, they should not strongly underperform on some samples. Each caller already fields its own complex modelling of somatic variants, so we concentrated only on high-level features to adjudicate among them. The 6 features used in training the consensus ensemble models are the calls themselves, read counts (using bam-readcount for SNVs or SGA for indels) supporting and not supporting the variant, VAF, 3-mer context on each end, repeat context, and presence or not in several regions or variant databases (*e.g.*, dbSNP, 1000 Genomes, COSMIC).

Also available to us in the consensus process were calls from an indel caller that had not participated in the validation pilot, SvABA[10], and a significantly updated version of the MuSE SNV caller, with recommendations for less stringent thresholding/filtering than what was used in the validation pilot. These callers introduced many variants not seen during the pilot phase of the project. Lack of validation data for these unique calls renders them too uncertain for full incorporation. Consequently, we opted for a conservative strategy where the MuSE SNVs and SvABA indels are used to support existing calls (as an additional input feature) from the validated callers, but calls unique to only these pipelines are excluded from the consensus set.

Simple models were preferred for both transparency of the results to the downstream analysis groups, and to avoid overfitting on the small number of cancer types in the validation samples compared to the full PCAWG population. The results of the consensus model approaches are shown in **Supplementary Figure 19.** For somatic SNVs, very simple combinations of the callers performed as well on average, and at least as well in the worst case, as more complex models, so a simple, interpretable "2+/4" approach was chosen, where calls seen by at least two callers were selected as consensus calls.

This simple approach was not feasible for indel calls because of the much lower degree of agreement, and other approaches, such as simple decision trees, SVM, random forest, and stacked logistic regression, were investigated.

All model training was performed in R. The stacked logistic regression model used the CRAN glmnet package; the random forest models used randomForest packages, respectively; and the kernlab package was used for support-vector machine (SVM) model learning. The baseline models, logistic regression and simple decision tree, used glmnet and party, respectively. Package defaults for binary classification were used for the hyperparameters.

As described above, the models were trained on features including the callers' calls, depths (normal and tumour), VAF (normal and tumour) repeat context, and presence of variant or region in various databases. In the non-stacked models, all of these features were treated symmetrically; in stacked logistic regression, following[1], weights for each caller were calculated as a function of the co-variates. This provides for a more interpretable model, as the reasons for preferring a given caller for a particular call were explicit. All of the non-baseline gave extremely high precision and good sensitivity; with no clear winner between the four, we selected the more interpretable stacked logistic regression method which is well motivated in the somatic mutation calling literature[1].

Once chosen - and in the case of indels, the model trained over all validation data sets - the models were applied to the variant call sets for all PCAWG samples. The final merged call sets were annotated with variant consequence for protein-coding genes and location for non-coding (UTR, RNA, intronic, intergenic), whether the variant occurs at a known dbSNP site and 1000 genomes data and reference context around the event. The annotated call sets were disseminated to the working groups for downstream analysis.

# 3. Performance on Previously Validated Samples

To assess the performance of the final consensus pipeline we ran the production version of each calling pipeline on three well-characterized samples external to the project.

## Medulloblastoma

The pipelines were run on a medulloblastoma sample sequenced as a benchmark for the ICGC[11]. This sample has a curated "gold set" of calls, including many having low-VAF, based on a very high-depth (300x) data set aggregated from several sequencing centres. This tumour has a low mutation rate, with 1,263 SNVs and 347 indels, both of which are on the extreme low side of our validation samples. As such, we expected accuracies to be low, both for the individual callers and for the ensemble methods.

Results are shown in **Supplementary Table 18.** For SNVs, the accuracies are indeed on the low side of the observed range of values for the validation samples, but the consensus call set outperforms the input call sets on sensitivity, precision, and F1 scores. For indels, overall accuracies for the consensus call set are closer to typical values seen on the validation samples, but the input DKFZ caller performs better on sensitivity, at the expense of significantly worse precision.

## Known Cell lines

Two well-characterized human cancer cell lines, HCC1143 and HCC1954, were also analyzed by the production pipelines for this project; a "gold set" of curated SNV calls in use internally at the Broad Institute was used to measure the accuracy of the core and consensus callers. In both cases, the consensus SNV calls were competitive with the best of the core callers (**Supplementary Table 19).**

# 4. Production Somatic Variant Calling on the PCAWG Compute Cloud

The final process flow chart for WGS somatic variant calling is shown in **Supplementary Figure 2.** It consists of 15 major processing steps whose outputs were filtered and combined to produce the consensus lists of somatic SNVs, indels, SCNAs and SVs used for downstream analysis.

Unaligned BAM files representing tumour and normal genomes were aligned with BWA-MEM to produce aligned BAMs. Paired aligned BAM files representing tumour and normal genomes for each PCAWG donor were then passed to six variant calling pipelines: (1) the EMBL pipeline for SV/CNA calls; (2) the DKFZ pipeline for SNV/indel calls; (3) the Sanger pipeline for SNV, indel, SV and SCNA calls; (4) the Broad pipeline for SNV, indel, SV and SCNA calls; (5) SMuFIN for indels; and (6) MuSE for SNV calls only. SNVs and indels were annotated for functional impact by Oncotator[1], and the sets of variants called by each pipeline were then merged into consensus sets using two pipelines: the SV-Merge package for SVs and SCNAs, and the SNV-MERGE package for SNVs and indels.

Alignments for the Broad and MuSE pipelines were preprocessed with local indel realignment and BQSR. Local realignment around indels in both the tumour and matched normal ensures alignment consistency in samples of the same individual. This serves to prevent spurious somatic indels and SNV calls near such sites. BQSR adjusts the base quality scores in the sequencing data according to the empirical error distribution[12].

Following consensus SNV and indel calling, three filters were run to identify and remove suspect SNVs: (1) a strand bias filter to remove SNVs whose evidence is heavily weighted to one strand or another, and (2) a panel of normals (PoN) filter to identify and remove uncommon germline polymorphisms using a deep alignment of 2,450 PCAWG normal genomes, and (3) OxoG[4] algorithm to flag and remove those variants likely miscalled due to oxidative damage artefact.

After generation of all unmerged variants, we generated a series of “miniBAM” files using a novel format for representing the evidence that underlies genomic variant calls. A miniBAM contains the read pairs that span a called variant within a specified window, greatly reducing the size of the aligned BAM file while preserving the neighbourhood around each called variant. The parameters we chose for miniBAM generation resulted in a 200-fold reduction in the size of the aligned BAMs, totalling about 4 TB for all PCAWG specimens and making it easier to download and store for the purpose of inspecting variants and their underlying read evidence.

Each of the steps shown in **Supplementary Figure 2** has been packaged as an executable image using the Dockstore[13] software containerization system. This system allows for complex workflows to be conveniently packaged into standalone images and executed across any compute environment that supports the Docker (www.docker.com) containerization system. **Supplementary Table 3** provides shortened links to the Dockstore package that contains each workflow step. See **Supplementary Methods S2** for details on each of the algorithms and software packages used.

## Distributed Processing

The size of the data set (in excess of 800 TB) and the high computational demands of the alignment and somatic variant calling workflow described presented logistic challenges. Our solution was to distribute the project's storage and computation across a series of 15 data centres which were either donated by PCAWG participants or leased from commercial cloud service providers (**Supplementary Table 20**). There were three broad phases of the project: (1) Marshalling and upload of the data into data analysis centres (3 months); (2) Alignment and variant calling (18 months); and (3) Quality filtering, merging, synchronization and distribution of the variant calls to downstream research groups (2 months).

*Phase 1: Data Marshalling and Upload.* A significant challenge for the project was that at its inception, a large portion of the raw read sequencing data had yet to be submitted to a read archive and thus had no standard retrieval mechanism. In addition, the metadata standards for describing the raw data varied considerably from project to project. For this reason, we asked the participating projects to prepare and upload the 774 TB of raw whole genome sequencing (WGS) data and 27 TB raw RNA-seq data into a series of geographically distributed data repositories, each running a uniform system for registering the data set, accepting and validating the raw read data and standardized metadata.

We utilized seven geographically distributed data repositories located at: (1) Barcelona Supercomputing Centre (BSC), (2) European Bioinformatics Institute (EMBL-EBI) in the UK, (3) German Cancer Research Center (DKFZ) in Germany; (4) the University of Tokyo in Japan; (5) Electronics and Telecommunications Research Institute (ETRI) in South Korea; (6) the Cancer Genome Hub (CGHub) and (7) the Bionimbus Protected Data Cloud (PDC) in the USA.

To accept and validate sequence set uploads, each data repository ran a commercial software system, GNOS (Annai Systems). We chose GNOS because of the heavy testing it had previously received as the engine powering TCGA CGHub, and its support for validation of metadata according to the Sequence Read Archive (SRA) standard and file submission, strong user authentication and encryption, as well as its highly optimized data transfer protocol [CITE]. Each of the seven data centers initially allocated several hundred terabytes of storage to accept raw sequencing data from submitters within the region. The data centers also provided co-located compute resources to perform alignment and variant calling on the uploaded data.

Genomic data uploaded to the GNOS repositories was accompanied with detailed and accurate metadata to describe the cancer type, sample type, sequencing type and other attributes for managing and searching the files. We required that identifiers for project, donor, sample follow a standardized convention such that validation and auditing tools could be implemented. Most of the naming conventions in PCAWG were adopted from the well-established ICGC data dictionary (http://docs.icgc.org/dictionary/about/).

Since most member projects at the time of upload already had sequencing reads aligned and annotated using their own metadata standards, a non-trivial effort was required to prepare the sequencing data for submission to GNOS. Each member project had to (1) prepare lane-level unaligned reads in BAM format, (2) reheader the BAM files with metadata following the PCAWG conventions, (3) generate metadata XML files, and (4) upload the BAM files along with the metadata XML files to GNOS. To facilitate this process, we developed the *PCAP-core*

tool (https://github.com/ICGC-TCGA-PanCancer/PCAP-core) to extract the metadata from the BAM headers, validate the metadata, transform the metadata into the XML files conforming to the SRA specifications, and submitting the BAM files along with the metadata XML files to GNOS.

*Phase 2: Sequence Alignment and Variant Calling.* We began the process of sequence alignment about two months after the uploading process had begun. When possible, both the alignment and variant calling pipelines were executed in the same regional compute centers to which the data sets were uploaded. As the project progressed, we utilized additional compute resources from AWS, Azure, iDASH, the Ontario Institute for Cancer Research (OICR), the Sanger Institute, and Seven Bridges. These centers computed on data sets located in the same region to optimize data transfer. Over the course of the project, some centers outpaced others and we rebalanced data sets as needed to use resources as efficiently as possible.

*Phase 3: Variant merging, filtering, and synchronization.* Following the completion of the variant calling workflows, variants were passed to additional filtering and annotation pipelines as described earlier, and the evidence for each filtered and unfiltered variant was captured in a series of miniBAM files. Variants from multiple call sets were then merged as described as above and in **Supplementary Methods S2.4.**

We then used GNOS to synchronize the aligned reads and variant call sets among a small number of download sites for use by PCAWG downstream analysis working groups (**Supplementary Table 21**). We also provided login credentials to members of PCAWG working groups for compute cloud-based access to the aligned read data across several of the regional data analysis centers, which avoided the overhead of downloading the data.

## Data Distribution to Downstream Analytic Groups

While GNOS was used for the core pipelines, Synapse (https://www.synapse.org/) was used to provide an interface to the files generated by the working groups and other intermediate results created throughout the project. Unlike GNOS which is focused on archival storage, Synapse allowed for collective editing in the form of a wiki, provenance tracking and versioning of results through a web interface as well as programmatic APIs. While Synapse provided an interface that allowed analyses to be shared rapidly across the consortia, the controlled access data was stored on a secure SFTP server provided by the National Cancer Institute (NCI). As the working groups completed their analysis, the metadata was retained in Synapse while the final version of the results was transferred to the ICGC Data Portal (https://dcc.icgc.org) for archival.

In addition to GNOS-based repositories, the PCAWG dataset has been mirrored to multiple locations: the European Genome-phenome Archive (EGA, https://www.ebi.ac.uk/ega/studies/EGAS00001001692), AWS Simple Storage Service (S3, https://dcc.icgc.org/icgc-in-the-cloud/aws), and the Cancer Genome Collaboratory (http://cancercollaboratory.org). To help researchers locate the PCAWG data, the ICGC Data Portal (https://dcc.icgc.org) provides a faceted search interface to query about donor, cancer type, data type or data repositories. Users can browse the collection of released PCAWG data and generate a manifest that facilitates downloading of the selected files.

The data repositories hosted at AWS S3 and the Collaboratory are powered by an open source object-based ICGC Storage System (https://github.com/icgc-dcc/dcc-storage) that enables fast, secure and multi-part downloads of files. Since AWS and the Collaboratory also have compute power co-located with the PCAWG data, they serve as effective cloud resources for researchers wishing to conduct further analyses on the PCAWG data without having to provision local compute resources and to download terabytes of data to their local compute environment.

# 5. PCAWG data portals

The PCAWG Landing Page at http://docs.icgc.org/pcawg provides links to several data resources for interactive online browsing, analysis and download of PCAWG data and results. We developed five data portals to provide routes into the PCAWG data: ICGC Data Portal; UCSC Xena; Chromothripsis Explorer; Expression Atlas; and PCAWG-Scout. These five resources are built upon the primary genomic and transcriptomic data types generated by the PCAWG project, including simple somatic mutations (single- and multiple- nucleotide variants (SNVs, MNVs)); small insertions and deletions (INDELs); large somatic structural variants (SVs); copy number variants; gene fusions; RNA-seq gene- and miRNA-expression; DNA methylation; and phenotypic annotations.

Two types of files were generated by the PCAWG analysis: primary BAM and VCF files, and downstream analysis results. The ICGC Data Portal provides a uniform search interface for both file types (https://dcc.icgc.org). Each of the four other resources, UCSC Xena, Chromothripsis Explorer, Expression Atlas, and PCAWG-Scout, separately ingested the same primary result files and individually refined them for online visualization, exploration and download.

## The five data portals

**1. ICGC Data Portal -** https://dcc.icgc.org

The ICGC Data Portal[14] serves as the main entry point for accessing PCAWG datasets with a single uniform web interface and a high performance data download client. This uniform interface gives users easy access to the myriad of PCAWG sequencing data and variant calls that reside in many repositories and compute clouds worldwide. The intuitive search interface is enabled through permanent, unique ICGC identifiers for each file and a set of harmonised metadata (such as data types and formats, experimental assays and computation methods). Streaming technology gives users high-level visualisations in real time of BAM and VCF files stored remotely on Amazon Web Services and the Cancer Genome Collaboratory. PCAWG consensus simple somatic mutations (excluding non-coding mutations from US projects due to policy constraints) are integrated with clinical data elements and rich functional annotations including affected proteins, pathways, gene ontology terms, and other factors. The Advanced Search and Analysis tools allow users to explore functional associations with phenotypic data such as molecular subtype and patient survival.

All raw and derived data from the PCAWG project are available for general research use. In accordance with the data access policies of the ICGC and TCGA projects, most molecular, clinical and specimen data are in an open tier which does not require access approval. To access potentially identifying information, such as germline alleles and underlying read data, researchers will need to apply to the TCGA Data Access Committee (DAC) via dbGaP (https://dbgap.ncbi.nlm.nih.gov/aa/wga.cgi?page=login) for access to the TCGA portion of the data set, and to the ICGC Data Access Compliance office (DACO; http://icgc.org/daco) for the remainder. Currently, TCGA defines non-coding somatic SNVs and indels identified by WGS as controlled tier elements, while ICGC places them in the open tier. To obtain the full catalogue of somatic variants, researchers will need to obtain TCGA authorization.

*Direct download of PCAWG data*

Aligned PCAWG read data in BAM format is also available at the European Genome Phenome Archive (EGA; https://www.ebi.ac.uk/ega/search/site/pcawg under accession EGAS00001001692). In addition, all open tier PCAWG genomics data as well as reference data sets used for analysis, can be downloaded from the ICGC Data Portal at http://docs.icgc.org/pcawg/data/. Researchers who have obtained ICGC DACO authorisation, and have logged into the Data Portal using their credentials, will also have access to genotype and haplotype calls for the ICGC-originated subset of PCAWG donors.

Controlled tier genomic data, including SNVs and indels that originated from TCGA projects (in VCF format), and aligned reads (in BAM format) can be downloaded using the icgc-storage-client software package, which implements accelerated and secure file transfer. Instructions for installing and using this software can be found at http://docs.icgc.org/pcawg/data/

*Compute cloud-based access to PCAWG data*

Because of the large size of the PCAWG data files, which are in excess of 800TB for the aligned read files and 750GB for the combined genotype and somatic variant call sets, we encourage researchers to make use of several compute cloud systems that provide fast and convenient access to PCAWG raw and analysed data:

● The Cancer Genome Collaboratory (https://www.cancercollaboratory.org/) is a Canadian compute cloud facility that provides academic and non-academic researchers with the ability to configure and launch virtual machines in a secure private workspace. The open tier data set and the ICGC-originated controlled tiers are resident within the Collaboratory where they can be transiently copied into the researcher's workspace for inspection and analysis. Controlled-tier TCGA data can be transferred into a user's workspace using a fast network link that connects the Collaboratory to its sister facility, the Protected Data Cloud (see below). This facility charges for compute and storage use at heavily subsidised rates.

● The Bionimbus Protected Data Cloud (https://bionimbus-pdc.opensciencedatacloud.org/) is an American compute cloud facility that provides secure compute and storage facilities to academic and non-academic researchers. This cloud holds the TCGA-originated controlled tier data set. ICGC-originated controlled tier data can be

copied into transient or permanent storage in the researcher's workspace over a fast link that interconnects the Protected Data Cloud and the Collaboratory. Use of compute and storage facilities is currently free for researchers who have dbGaP authorization for access to TCGA data.

- Amazon Web Services (https://aws.amazon.com/) is a commercial cloud compute facility. PCAWG variant calls are mirrored on the Simple Storage Service (S3), along with roughly 85% of the ICGC-originated portion of the controlled tier, including whole genome alignments. S3-resident PCAWG data can be transferred into a user's storage area for inspection and analysis using S3 GET and other standard tools, while both resident and non-resident data can be copied into Amazon Web Services using icgc-storage-client. Amazon Web Services charge market rates for compute and data storage.

**2. UCSC Xena - https://pcawg.xenahubs.net**

UCSC Xena visualises all PCAWG primary results, including copy number, gene expression, gene fusion, promoter usage, simple somatic mutations, large somatic structural variation, mutational signatures and phenotypic data. This open-access data is available through a public Xena hub, while consensus simple somatic mutations (including both coding and non-coding) can be loaded into a user's local computer private Xena hub. The UCSC Xena Browser accesses data from multiple data hubs simultaneously, allowing users to visualise PCAWG data alongside their own private data while maintaining data security. Xena integrates simple mutations, structural variants, gene expression data and more, for the same or multiple genes across large numbers of samples. Kaplan-Meier plots, histograms, boxplots, scatterplots and transcript-specific views offer additional visualisation options and statistical analyses.

The following open-access PCAWG results are hosted on UCSC Xena:

- Consensus coding SNVs, MNVs, and small indels
- Consensus whole-genome SNVs, MNVs, and small indels from non-US donors
- Consensus whole genome structural variants
- Consensus whole genome copy number
- RNA-seq gene expression, consensus fusion calls, alternative promoter usage and JuncBASE alternative splicing events
- miRNA expression
- Coding drivers
- Mutation Signatures
- Tumour purity and ploidy

- Tumour subtype, histology information, donor clinical data
- Specimen quality control designation (white, grey and excluded lists)

**3. Expression Atlas -** https://www.ebi.ac.uk/gxa/experiments?experimentSet=Pan-Cancer

The Expression Atlas contains RNAseq and expression microarray data for querying gene expression across tissues, cell types, developmental stages and/or experimental conditions. Queries can be either in a baseline context or in a differential context. PCAWG RNAseq gene expression data are manually curated to a high standard by Expression Atlas curators and are presented in a heatmap with summarised baseline expression. Two different views of the data are provided: summarised expression levels for each tumour type and gene expression at the level of individual samples.

**4. PCAWG-Scout -** http://pcawgscout.bsc.es/

PCAWG-Scout provides a framework for 'omics workflow and website templating to make on-demand, in-depth analyses over the PCAWG data openly available to the whole research community. Views of protected data are available that still safeguard sensitive data. Through the PCAWG-Scout web interface, users can access an array of reports and visualisations that leverage on-demand bioinformatic computing infrastructure to produce results in real-time, allowing users to discover trends as well as form and test hypotheses. The web interface and underlying infrastructure are open-source, based on the Ruby bioinformatics toolkit (Rbbt), and can be installed locally, with new reports added or altered easily through the modular templating system. This also allows the entire analysis suite to be applied to datasets outside those from PCAWG.

Gene expression data are specifically processed for each visualisation or analysis in PCAWG-Scout. For differential expression, the values are log2 transformed, with 'no expression' replaced by the smallest number found in the matrix. For differential expression and for expression boxplots on a single gene, only tumour samples are considered and all possible samples for every donor in the group are shown together. When using a colour gradient to represent expression of a gene in a donor, all tumour samples for that donor are averaged and the expression is compared with the rest of values for the other tumour samples in the cohort; the rank of that value in the list for all samples in the cohort is used to define the gradient.

**5. Chromothripsis Explorer -** http://compbio.med.harvard.edu/chromothripsis/

The Chromothripsis Explorer portal enables exploration of patterns of chromothripsis in the PCAWG dataset. Chromothripsis Explorer enables the user to explore and visualise the tumours comprising the PCAWG cohort, including properties such as purity and ploidy. A key feature of the portal is to provide interactive circos plots for all tumours, with the plots including tracks for the somatic SNV, indel and structural variation events, as well as the total

and minor copy number profiles for chromosomes 1-22 and X. These plots provide an easy and intuitive interface for visualisation of complex mutational profiles such as chromothripsis and kataegis, deletions of chromosome arms, loss of heterozygosity and so on.

In addition to explore the PCAWG data case-by-case through circos plots, Chromothripsis Explorer also provides a tunable search-and-summarise module for generating summary plots of chromothripsis events by, for example, tumour type, number of breakpoints, number of chromosomes affected and so on.

## Data sources for the PCAWG portals

Each of the PCAWG portals is based on importing, combining and displaying primary PCAWG datasets, each of which is referenced by a corresponding synapse ID - these are listed in the table below. Synapse folder ID is the identifier for the synapse landing page for each type of primary results. The landing page typically includes a summary written by the analysis working group to briefly describe the bioinformatics methods used and a list of results generated. Because there are often multiple versions of the same results files (such as fpkm vs fpkm-uq gene expression estimations, or simple mutations from all specimens or aggregated by donors), synapse identifiers in the remaining columns point to the actual data file ingested by each online resource. The data snapshot was taken as of Feb 10, 2017. RNAseq data are visualised by Expression Atlas, UCSC Xena and PCAWG-Scout. Each resource started with the same primary results generated by the analysis working group, and subsequently further processed, curated and refined to meet each resource's quality-control and visualisation requirements. The secondarily processed data are displayed on the web.

| Data | UCSC Xena | Expression Atlas | PCAWG-Scout | Chromothripsis Explorer |
|---|---|---|---|---|
| **Consensus SNVs and indels** | syn7364923 | | syn7364923 | syn7357330 |
| **Consensus SVs** | syn7596712 | | syn7596712 | syn7596712 |
| **Consensus copy number** | syn8042988 | | syn8042988 | syn8042988 |
| **Gene expression** | syn5553991 | syn5553983<br>syn5553985 | syn5553991 | |
| **GTEx gene expression using PCAWG RNA-seq SOP** | | syn8105922 | | |
| **RNAseq gene fusion** | syn7221157 | | | |
| **RNAseq alternative promoter usage** | syn10332949 | | | |
| **small RNA-Seq (miRNA) analyses** | syn5878064<br>syn5878067 | | | |
| **Patient-centric driver catalogue** | syn11639581 | | syn11639581 | |
| **APOBEC mutagenesis analysis** | syn7437313 | | | |
| **Tumour subtype and histology information** | syn10389164 | syn10389164 | syn10389164 | syn10389164 |
| **Donor clinical data** | syn10389158 | | syn10389158 | syn10389158 |
| **Consensus purity and ploidy** | | | | syn8272483 |

## 6. Literature Cited


1. Kim, S. Y., Jacob, L. & Speed, T. P. Combining calls from multiple somatic mutation-callers. *BMC Bioinformatics* **15**, 154 (2014).

2. Ewing, A. D. *et al.* Combining tumour genome simulation with crowdsourcing to benchmark somatic single-nucleotide-variant detection. *Nature Methods* **12**, 623–630 (2015).

3. Validating multiple cancer variant callers and prioritization in tumour-only samples. *Blue Collar Bioinformatics* Available at: http://bcb.io/2015/03/05/cancerval/. (Accessed: 8th March 2019)

4. Costello, M. *et al.* Discovery and characterization of artifactual mutations in deep coverage targeted capture sequencing data due to oxidative DNA damage during sample preparation. *Nucleic Acids Res.* **41**, e67 (2013).

5. Moncunill, V. *et al.* Comprehensive characterization of complex structural variations in cancer by directly comparing genome sequence reads. *Nat. Biotechnol.* **32**, 1106–1112 (2014).

6. Lohr, S. *Sampling: Design and Analysis*. (Cengage Learning, 2009).

7. Ewing, A. D. *et al.* Combining tumour genome simulation with crowdsourcing to benchmark somatic single-nucleotide-variant detection. *Nat. Methods* **12**, 623–630 (2015).

8. A. F. A. Smit, R. Hubley, P. Green. RepeatMasker Open-4.0. *RepeatMasker* (2015). Available at: http://www.repeatmasker.org. (Accessed: November 2015)

9. GmbH, R. D. *HOWTO Evaluate SeqCap EZ Target Enrichment Data*. (2014).

10. Wala, J. A. *et al.* SvABA: genome-wide detection of structural variants and indels by local assembly. *Genome Research* **28**, 581–591 (2018).

11. Alioto, T. S., Buchhalter, I., Derdak, S. & Hutter, B. A comprehensive assessment of somatic mutation detection in cancer using whole-genome sequencing. *Nature Communications* (2015).

12. Tian, S., Yan, H., Kalmbach, M. & Slager, S. L. Impact of post-alignment processing in variant discovery from whole exome data. *BMC Bioinformatics* **17**, 403 (2016).

13. O'Connor, B. D. *et al.* The Dockstore: enabling modular, community-focused sharing of Docker-based genomics tools and workflows. *F1000Res.* **6**, 52 (2017).

14. Zhang, J. *et al*. The International Cancer Genome Consortium Data Portal. *Nat. Biotechnol*. **37**, 367–369 (2019).
