# Supplementary material for: Pan-cancer analysis of whole genomes
Source: Nature. 2020 Feb 5;578(7793):82–93. doi: 10.1038/s41586-020-1969-6 (PMC7025898; doi:10.1038/s41586-020-1969-6)
Supplement: Supplementary file 3 — This zipped file contains Supplementary Tables 1-21 and a Supplementary Table Guide [file 41586_2020_1969_MOESM3_ESM.zip › supplementary Tables/Supplementary Table 11.docx]

**Supplementary Table 11.** Telomere-related features used to cluster tumour genomes

| **Feature** | **Description** |
| --- | --- |
| Telomere Length Ratio Tumor/Normal | Log2 ratio of tumour telomere content and matched normal telomere content |
| Telomere Insertions | Log2(x+1) number of telomere insertions into non-telomeric regions of the genome |
| Genomic Breakpoints | Log2(x+1) number of genomic breakpoints in the non-telomeric regions of the genome |
| 9 Telomere Variant Repeats (TVRs):  ATAGGG  CTAGGG  TAAGGG  TTCGGG  TTGGGG  TTTGGG  TCAGGG  GTAGGG  TGAGGG | Log2(x+1) number of TVR patterns calculated as the distance from the expected pattern occurrence. The expected pattern occurrence is the number of TVR patterns in the normal samples corrected by telomere length by calculating a regression line dependent on telomere length ratio. |

# 
