# Supplementary material for: Pan-cancer analysis of whole genomes
Source: Nature. 2020 Feb 5;578(7793):82–93. doi: 10.1038/s41586-020-1969-6 (PMC7025898; doi:10.1038/s41586-020-1969-6)
Supplement: Supplementary file 3 — This zipped file contains Supplementary Tables 1-21 and a Supplementary Table Guide [file 41586_2020_1969_MOESM3_ESM.zip › supplementary Tables/Supplementary Table 13.docx]

**Supplementary Table 13. Element-specific features scored for use in OnCohortDrive**

| **Element** | **Score** | **Description** |
| --- | --- | --- |
| CDS | Truncating mutation in a tumour suppressor gene | High confidence truncating variants in tumor suppressor genes were obtained using Variant Ensembl Predictor^1^ (VEP) with LOFTEE plugin. |
| Promoters, 5’-UTR, enhancers | TFBS creation and disruption | The scores for TFBS creation (motif gain) and disruption (motif break) were computed by following the steps described in Fu et al. (FunSeq2)^2^. The score value indicates the difference between position weight matrix matching scores of the reference and mutant alleles. |
| 3’-UTR | Experimentally verified and *in silico* prediction of miRNA binding disruption | This includes the collection of miRNA binding sites that were experimentally identified using AGO-CLIP from 32 individual datasets, as well as the TargetScan predicted miRNA binding motifs on reference sequence (provided by Mark Hamilton; syn4485000). |
| miRNA | RNASnp | The impact of mutations on the secondary structure of miRNAs (hairpins) were computed using the RNAsnp^3^. The ‘Mode 1’ option of RNAsnp was used to obtain the Euclidean distance score, which indicates the differences in the base pair probabilities of wild-type and mutant structure. |

References:

1. McLaren, W. et al. The Ensembl Variant Effect Predictor. Genome Biol. 17, 122 (2016).
2. Fu, Y. et al. FunSeq2: a framework for prioritizing noncoding regulatory variants in cancer. Genome Biol. 15, 480 (2014).
3. Sabarinathan, R. et al. RNAsnp: efficient detection of local RNA secondary structure changes induced by SNPs. Hum. Mutat. 34, 546–56 (2013).
