# Supplementary material for: Pan-cancer analysis of whole genomes
Source: Nature. 2020 Feb 5;578(7793):82–93. doi: 10.1038/s41586-020-1969-6 (PMC7025898; doi:10.1038/s41586-020-1969-6)
Supplement: Supplementary file 3 — This zipped file contains Supplementary Tables 1-21 and a Supplementary Table Guide [file 41586_2020_1969_MOESM3_ESM.zip › supplementary Tables/Supplementary Table 14.docx]

**Supplementary Table 14. Other evaluated features used in onCohortDrive**

| **Element** | **Score/Description** | **Contributor** |
| --- | --- | --- |
| CDS | Mutations affecting protein posttranslational modification (PTM) sites (computed using ActiveDriver2^1^) | Juri Reimand |
| CDS | Genes with significant clustering of missense mutations in the 3D protein structure (predicted using CLUMPS^2^) | Atanas Kamburov |
| CDS | Identification of pathogenic variants in human kinases (predicted using KinMut^3^) | Miguel Vazquez and Jose MG Izarzugaza |
| CDS | Mutations affecting protein-protein interfaces (predicted using structure-PPi^4^) | Miguel Vazquez |
| CDS | Mutations affecting catalytic and ligand-binding residues in protein sequences (predicted using firestar) | Miguel Vazquez |

References:

1. Reimand, J. & Bader, G. D. Systematic analysis of somatic mutations in phosphorylation signaling predicts novel cancer drivers. Mol. Syst. Biol. 9, 637 (2013).
2. Kamburov, A. et al. Comprehensive assessment of cancer missense mutation clustering in protein structures. Proc Natl Acad Sci U S A. 112(40):E5486-95 (2015).
3. Izarzugaza, J. M., del Pozo, A., Vazquez, M. & Valencia, A. Prioritization of pathogenic mutations in the protein kinase superfamily. BMC Genomics 13, S3 (2012).
4. Vázquez, M., Valencia, A. & Pons, T. Structure-PPi: a module for the annotation of cancer-related single-nucleotide variants at protein–protein interfaces: Fig. 1. Bioinformatics 31, 2397–2399 (2015).
