# Supplementary material for: Pan-cancer analysis of whole genomes
Source: Nature. 2020 Feb 5;578(7793):82–93. doi: 10.1038/s41586-020-1969-6 (PMC7025898; doi:10.1038/s41586-020-1969-6)
Supplement: Supplementary file 3 — This zipped file contains Supplementary Tables 1-21 and a Supplementary Table Guide [file 41586_2020_1969_MOESM3_ESM.zip › supplementary Tables/Supplementary Table 15.docx]

**Supplementary Table 15. Groups of coding genes according to the confidence that they drive tumorigenesis in the cancer type under analysis and the mutations considered for driver nomination according to their probability.**

| **Confidence level of genes (decreasing)** | **Evidence of the genes driving tumorigenesis in the tumour type** | **Mutations in probability group** |
| --- | --- | --- |
| 1 | Experimentally validated gene in the same tumour type | 1-6 |
| 2 | Gene identified by IntOGen with at least two signals of positive selection in the same tumour type | 1-5 |
| 3 | Experimentally validated gene in other tumour type | 1-4 |
| 4 | Gene identified by IntOGen with at least two signals of positive selection in other tumour type | 1-3 |
| 5 | Gene identified by IntOGen with only one signal of positive selection in the same tumour type | 1-2 |
| 6 | Gene identified by IntOGen with only one signal of positive selection in other tumour type, or in a pan-cancer analysis | 1 |
