# Supplementary material for: Pan-cancer analysis of whole genomes
Source: Nature. 2020 Feb 5;578(7793):82–93. doi: 10.1038/s41586-020-1969-6 (PMC7025898; doi:10.1038/s41586-020-1969-6)
Supplement: Supplementary file 3 — This zipped file contains Supplementary Tables 1-21 and a Supplementary Table Guide [file 41586_2020_1969_MOESM3_ESM.zip › supplementary Tables/Supplementary Table 16.docx]

**Supplementary Table 16. TCGA cohorts employed to identify GISTIC significant peaks.**

| **Cancer type acronym** | **Samples** | **Cancer type** |
| --- | --- | --- |
| brca | 1080 | Breast invasive adenocarcinoma |
| ov | 579 | Ovarian serous cystadenocarcinoma |
| gbm | 577 | Glioblastoma multiforme |
| ucec | 539 | Uterine corpus endometrioid carcinoma |
| kirc | 528 | Kidney renal clear cell carcinoma |
| hnsc | 522 | Head and neck squamous cell carcinoma |
| luad | 516 | Lung adenocarcinoma |
| lgg | 513 | Brain lower grade glioma |
| lusc | 501 | Lung squamous cell carcinoma |
| thca | 501 | Thyroid carcinoma |
| prad | 492 | Prostate adenocarcinoma |
| coad | 450 | Colon adenocarcinoma |
| stad | 441 | Stomach adenocarcinoma |
| blca | 408 | Bladder urothelial carcinoma |
| lihc | 370 | Liver hepatocellular carcinoma |
| skcm | 366 | Cutaneous melanoma |
| cesc | 295 | Cervical squamous cell carcinoma |
| kirp | 288 | Kidney renal papillary cell carcinoma |
| sarc | 256 | Sarcoma |
| laml | 191 | Acute myeloid leukaemia |
| esca | 184 | Oesophageal carcinoma |
| paad | 184 | Pancreatic adenocarcinoma |
| read | 165 | Rectum adenocarcinoma |
| pan-cancer | 10844 | All cancers, including other TCGA types |
| all_neural | 1090 | Combined lgg and gbm |
| all_lung | 1017 | Combined lusc and luad |
| all_colorectal | 615 | Combined coad and read |
| kirckirp | 816 | Combined kirc and kirp |
