# Supplementary material for: Pan-cancer analysis of whole genomes
Source: Nature. 2020 Feb 5;578(7793):82–93. doi: 10.1038/s41586-020-1969-6 (PMC7025898; doi:10.1038/s41586-020-1969-6)
Supplement: Supplementary file 3 — This zipped file contains Supplementary Tables 1-21 and a Supplementary Table Guide [file 41586_2020_1969_MOESM3_ESM.zip › supplementary Tables/Supplementary Table 17.docx]

**Supplementary Table 17.** Summary of SNV and indel callers used in the pilot evaluation

| \| Single Nucleotide Variants \| Insertions/Deletions \| \| --- \| --- \| \| Broad - MuTect[^1^](https://paperpile.com/c/MbPscO/4M5Yy) \| Broad - MuTect2 (pilot only) \| \| DKFZ \| Broad - SvABA[^2^](https://paperpile.com/c/MbPscO/Nqhxs)(production only) \| \| Sanger \| DKFZ \| \| MuSE[^3^](https://paperpile.com/c/MbPscO/ygpKj) \| Sanger \| \| ADISCAN_Beta[^4^](https://paperpile.com/c/MbPscO/jSQe1) \| CRG Clindel \| \| LOHcomplete \| novobreak-indel[^5^](https://paperpile.com/c/MbPscO/4GWp0) \| \| SGA[^6^](https://paperpile.com/c/MbPscO/LfhwI) \| SGA[^6^](https://paperpile.com/c/MbPscO/LfhwI) \| \| SMuFin[^7^](https://paperpile.com/c/MbPscO/aCasD) \| SMuFin[^7^](https://paperpile.com/c/MbPscO/aCasD) \| \| WUSTL \| WUSTL \| \| OICR BL \|  \| |
| --- | --- | --- | --- | --- | --- | --- | --- | --- | --- | --- | --- | --- | --- | --- | --- | --- | --- | --- | --- | --- | --- | --- |

1. [Cibulskis, K. *et al.* Sensitive detection of somatic point mutations in impure and heterogeneous cancer samples. *Nat. Biotechnol.* **31**, 213–219 (2013).](http://paperpile.com/b/MbPscO/4M5Yy)

2. [Wala, J. *et al.* Genome-wide detection of structural variants and indels by local assembly. *bioRxiv* 105080 (2017). doi:](http://paperpile.com/b/MbPscO/Nqhxs)[10.1101/105080](http://dx.doi.org/10.1101/105080)

3. [Fan, Y. *et al.* MuSE: accounting for tumor heterogeneity using a sample-specific error model improves sensitivity and specificity in mutation calling from sequencing data. *Genome Biol.* **17**, 1–11 (2016).](http://paperpile.com/b/MbPscO/ygpKj)

4. [Cho, Y. et al. Prevalence of rare genetic variations and their Implications in NGS-data interpretation. *Sci. Rep.* **7**, 9810 (2017).](http://paperpile.com/b/MbPscO/jSQe1)

5. [Chong, Z. *et al.* novoBreak: local assembly for breakpoint detection in cancer genomes. *Nat. Methods* **14**, 65–67 (2017).](http://paperpile.com/b/MbPscO/4GWp0)

6. [Simpson, J. T. & Durbin, R. Efficient de novo assembly of large genomes using compressed data structures. *Genome Res.* **22**, 549–556 (2012).](http://paperpile.com/b/MbPscO/LfhwI)

7. [Moncunill, V. *et al.* Comprehensive characterization of complex structural variations in cancer by directly comparing genome sequence reads. *Nat. Biotechnol.* **32**, 1106–1112 (2014).](http://paperpile.com/b/MbPscO/aCasD)
