# Supplementary material for: Pan-cancer analysis of whole genomes
Source: Nature. 2020 Feb 5;578(7793):82–93. doi: 10.1038/s41586-020-1969-6 (PMC7025898; doi:10.1038/s41586-020-1969-6)
Supplement: Supplementary file 3 — This zipped file contains Supplementary Tables 1-21 and a Supplementary Table Guide [file 41586_2020_1969_MOESM3_ESM.zip › supplementary Tables/Supplementary Table 18.docx]

**Supplementary Table 18.** Accuracies of core and consensus callers on the very low-mutation count Medulloblastoma benchmark; (top) SNVs, (bottom) indels

| \|  \| **Consensus** \| **MuTect** \| **DKFZ** \| **Sanger** \| \| --- \| --- \| --- \| --- \| --- \| \| **TP** \| **1005** \| 961 \| 890 \| 920 \| \| **FN** \| **258** \| 302 \| 373 \| 343 \| \| **FP** \| **150** \| 208 \| 229 \| 222 \| \|  \|  \|  \|  \|  \| \| **Precision** \| **0.87** \| 0.82 \| 0.80 \| 0.81 \| \| **Sensitivity** \| **0.80** \| 0.76 \| 0.70 \| 0.73 \| \| **F1** \| **0.83** \| 0.79 \| 0.75 \| 0.77 \| |
| --- | --- | --- | --- | --- | --- | --- | --- | --- | --- | --- | --- | --- | --- | --- | --- | --- | --- | --- | --- | --- | --- | --- | --- | --- | --- | --- | --- | --- | --- | --- | --- | --- | --- | --- | --- | --- | --- | --- | --- | --- |
| \|  \| **Consensus** \| **DKFZ** \| **Sanger** \| **SMuFin** \| \| --- \| --- \| --- \| --- \| --- \| \| **TP** \| 223 \| **273** \| 199 \| 97 \| \| **FN** \| 124 \| **74** \| 148 \| 250 \| \| **FP** \| **63** \| 244 \| 72 \| 293 \| \|  \|  \|  \|  \|  \| \| **Precision** \| **0.78** \| 0.53 \| 0.73 \| 0.25 \| \| **Sensitivity** \| 0.64 \| **0.79** \| 0.57 \| 0.28 \| \| **F1** \| **0.70** \| 0.63 \| 0.64 \| 0.26 \| |
