# Supplementary material for: Pan-cancer analysis of whole genomes
Source: Nature. 2020 Feb 5;578(7793):82–93. doi: 10.1038/s41586-020-1969-6 (PMC7025898; doi:10.1038/s41586-020-1969-6)
Supplement: Supplementary file 3 — This zipped file contains Supplementary Tables 1-21 and a Supplementary Table Guide [file 41586_2020_1969_MOESM3_ESM.zip › supplementary Tables/Supplementary Table 19.docx]

**Supplementary Table 19**. Accuracies of core and consensus SNV callers on the cancer cell lines HCC1143 (top) and HCC1954 (bottom).

| \|  \| **Consensus** \| **MuTect** \| **DKFZ** \| **Sanger** \| \| --- \| --- \| --- \| --- \| --- \| \| **TP** \| **17868** \| 17651 \| 14627 \| 16656 \| \| **FN** \| **976** \| 1193 \| 4217 \| 2188 \| \| **FP** \| 1596 \| 1307 \| 1205 \| **860** \| \|  \|  \|  \|  \|  \| \| **Precision** \| 0.92 \| 0.93 \| 0.92 \| **0.95** \| \| **Sensitivity** \| **0.95** \| 0.94 \| 0.77 \| 0.88 \| \| **F1** \| 0.93 \| **0.93** \| 0.84 \| 0.92 \| |
| --- | --- | --- | --- | --- | --- | --- | --- | --- | --- | --- | --- | --- | --- | --- | --- | --- | --- | --- | --- | --- | --- | --- | --- | --- | --- | --- | --- | --- | --- | --- | --- | --- | --- | --- | --- | --- | --- | --- | --- | --- |
| \|  \| **Consensus** \| **MuTect** \| **DKFZ** \| **Sanger** \| \| --- \| --- \| --- \| --- \| --- \| \| **TP** \| **17167** \| 16951 \| 14091 \| 15785 \| \| **FN** \| **988** \| 1204 \| 4064 \| 2370 \| \| **FP** \| 6103 \| 6037 \| **4163** \| 4772 \| \|  \|  \|  \|  \|  \| \| **Precision** \| 0.74 \| 0.74 \| **0.77** \| 0.77 \| \| **Sensitivity** \| **0.95** \| 0.93 \| 0.78 \| 0.87 \| \| **F1** \| **0.83** \| 0.82 \| 0.77 \| 0.82 \| |

# 
