# Supplementary material for: Pan-cancer analysis of whole genomes
Source: Nature. 2020 Feb 5;578(7793):82–93. doi: 10.1038/s41586-020-1969-6 (PMC7025898; doi:10.1038/s41586-020-1969-6)
Supplement: Supplementary file 3 — This zipped file contains Supplementary Tables 1-21 and a Supplementary Table Guide [file 41586_2020_1969_MOESM3_ESM.zip › supplementary Tables/Supplementary Table 2.docx]

**Supplementary Table 2. PCAWG germline variant call-set.** False discovery rate (FDR) estimates are defined as defined as FP/(FP+TP), where FP is the number of false positives and TP the number of true positives. Estimates of the germline variant sites list are based on ultra-deep resequencing of randomly picked candidate germline variant sites in n=48 PCAWG donors using custom sequence capture^#^ for SNPs and indels; intensity rank sum (IRS)[^23^](https://paperpile.com/c/olkLab/x04E) testing on the basis of Genome-Wide Human SNP Array 6.0 datasets available for a subset (*N*=787) of PCAWG normal tissue samples* for SVs; and mobile element insertions, including Alu, L1, and SVA were validated through Oxford Nanopore sequencing data available for one PCAWG normal tissue sample and one cell line derived from normal blood**. 798 variant sites within HLA regions (exons of *HLA-A, -B, -C, -DPA1, -DPB1, -DQA1, -DQB1, -DRB1*), median size of 1bp (median kb per individual: 0.320) were separately genotyped. *NA*, not assessed.

| **Germline variant**  **class** | **Number of sites** | **Median size (bp)** | **Median kbp**  **per individual** | **Site**  **FDR (%)** |
| --- | --- | --- | --- | --- |
| Biallelic SNVs | 80,085,108 | 1 | 3,536 | 0.43^#^ |
| Biallelic indels | 5,888,176 | 2 | 789 | 0.46^#^ |
| Multiallelic short variants | 1,789,419 | 1 | 689 | 1.17^#^ |
| Biallelic deletions ≥50bp | 29,492 | 2,743 | 2,805 | 4.88* |
| *Alu* insertions | 22,000 | 281 | 369 | 6.09** |
| L1 insertions | 4,302 | 1,593 | 538 | 2.15** |
| SVA insertions | 927 | 1,309 | 107 | 0.00** |
| ERV insertions | 25 | *NA* | *NA* | NA |
| **Total** | **87,816,050** |  |  |  |
