# Supplementary material for: Pan-cancer analysis of whole genomes
Source: Nature. 2020 Feb 5;578(7793):82–93. doi: 10.1038/s41586-020-1969-6 (PMC7025898; doi:10.1038/s41586-020-1969-6)
Supplement: Supplementary file 3 — This zipped file contains Supplementary Tables 1-21 and a Supplementary Table Guide [file 41586_2020_1969_MOESM3_ESM.zip › supplementary Tables/Supplementary Table 20.docx]

**Supplementary Table 20.** Percentage samples/donors run at each site for each pipeline.

| **Resource** | **Type** | **BWA** | **Sanger** | **DKFZ/EMBL** | **Broad/MuSE** | **OxoG** |
| --- | --- | --- | --- | --- | --- | --- |
| **AWS Ireland** | Commercial Compute Cloud (Amazon) | 5.0 | 16.4 | 0.6 |  | 31.1 |
| **Azure** | Commercial Compute Cloud (Microsoft) | 0.4 | 0.6 | 2.6 | 8.6 |  |
| **Barcelona Supercomputer Centre** | High Performance Compute Cluster | 10.2 | 17.2 | 28.5 |  |  |
| **Cancer Genome Collaboratory** | Academic Compute Cloud |  |  |  |  | 68.9 |
| **DKFZ (HPC)** | High Performance Compute Cluster |  |  | 55.8 |  |  |
| **DKFZ (OpenStack)** | Academic Compute Cloud | 14.5 | 10.2 | 8.5 |  |  |
| **EMBL-EBI** | Academic Compute Cloud | 12.6 | 3.3 |  |  |  |
| **ETRI** | High Performance Compute Cluster | 2.1 | 5.8 |  |  |  |
| **UC San Diego iDASH** | Academic Compute Cloud |  | 4.8 |  |  |  |
| **OICR** | High Performance Compute Cluster | 1.8 | 5.6 | 1.0 |  |  |
| **PDC** | Academic Compute Cloud | 11.8 | 4.2 |  |  |  |
| **Sanger** | High Performance Compute Cluster |  | 7.0 | 3.0 |  |  |
| **Seven Bridges** | Commercial Compute Cloud |  |  |  | 23.1 |  |
| **UCSC** | High Performance Compute Cluster | 30.6 | 13.0 |  | 68.2 |  |
| **UTokyo** | High Performance Compute Cluster | 10.9 | 11.9 |  |  |  |
