# Supplementary material for: Pan-cancer analysis of whole genomes
Source: Nature. 2020 Feb 5;578(7793):82–93. doi: 10.1038/s41586-020-1969-6 (PMC7025898; doi:10.1038/s41586-020-1969-6)
Supplement: Supplementary file 3 — This zipped file contains Supplementary Tables 1-21 and a Supplementary Table Guide [file 41586_2020_1969_MOESM3_ESM.zip › supplementary Tables/Supplementary Table 21.docx]

**Supplementary Table 21.** *Data distribution*. Latest information can be found at <https://dcc.icgc.org/repositories>

|  | **ICGC Data** | | | **TCGA Data** | | |
| --- | --- | --- | --- | --- | --- | --- |
| **Data Repository** | **% WG Alignments**  **(534 TB)** | **% RNA-Seq Alignments (13 TB)** | **% Variant calls**  **(520 GB)** | **% WG Alignments**  **(240 TB)** | **% RNA-Seq Alignments (14 TB)** | **% Variant calls**  **(227 GB)** |
| EGA* | 100 | 100 |  |  |  |  |
| Collaboratory* | 100.0 | 100.0 | 100.0 |  |  |  |
| AWS* | 75.6 | 76.9 | 73.8 |  |  |  |
| Bionimbus PDC* |  |  |  | 100.0 | 100.0 | 100.0 |
