# Supplementary material for: Pan-cancer analysis of whole genomes
Source: Nature. 2020 Feb 5;578(7793):82–93. doi: 10.1038/s41586-020-1969-6 (PMC7025898; doi:10.1038/s41586-020-1969-6)
Supplement: Supplementary file 3 — This zipped file contains Supplementary Tables 1-21 and a Supplementary Table Guide [file 41586_2020_1969_MOESM3_ESM.zip › supplementary Tables/Supplementary Table 3.docx]

**Supplementary Table 3.** Major algorithms and software versions used in somatic variant calling workflow

| **Workflow Step** | **Algorithm** | **Version** | **Dockstore Package^*^** |
| --- | --- | --- | --- |
| WGS Alignment | BWA-MEM | v0.7.8-r455 | https://goo.gl/oqp4Xd |
| EMBL/DKFZ SV caller | DELLY | v0.6.6 | https://goo.gl/Y46MCo |
| EMBL/DKFZ SCNA caller | ACEseq | v1.0.189 | https://goo.gl/4zoV42 |
| EMBL/DKFZ SNV caller | DKFZ somatic SNV workflow | 1.0.132-1 |  |
| EMBL/DKFZ indel caller | Platypus | v0.7.4 |  |
| Sanger SCNA caller | ascatNgs | v1.5.2 | https://goo.gl/9DSrbA |
| Sanger SV caller | BRASS | v4.012 |  |
|  | grass | v1.1.6 |  |
| Sanger SNV caller | CaVEMan | v1.50 |  |
| Sanger indel caller | Pindel | v1.5.7 |  |
| Broad SCNA caller | ABSOLUTE/JaBbA | v1.5/? | https://goo.gl/YkdtDt |
| Broad SV caller | SvABA/dRanger/BreakPointer | 2015-05-20/2016-03-13/2015-12-22 |  |
| Broad SNV caller | MuTect | v1.1.4 |  |
| Broad indel caller | SvABA | 2015-05-20 |  |
| MuSE SNV caller | MuSE | v1.0rc | https://goo.gl/5SR4bF |
| SMuFIN indel caller | SMuFIN | 2014-10-26 | https://goo.gl/EuUP5k |
| Oxidative artefact filter | OxoG | 2016-4- 28 | https://goo.gl/cUKP9K |
| SNV/Indel annotation | VAGrENT | v2.1.2 | https://goo.gl/9DSrbA |
|  | ANNOVAR | v2014Nov12 | https://goo.gl/4zoV42 |
| miniBAM generation | VariantBAM | v2017Dec12 | https://goo.gl/S8h8e5 |
| SNV/Indel merging and consensus generation | SNV-MERGE | v2017May26 | https://goo.gl/TETSB8 |
| SV merging and consensus generation | SV-MERGE | v2017Dec12 | https://goo.gl/A9CEup |
| Strand bias filter | DKFZ Strand Bias Filter | v2016Dec15 | https://goo.gl/8jXrvZ |

* Dockstore packages may execute several workflow steps. Tutorial-style instructions for running each Dockstore package can be found by following the corresponding link.
