# Supplementary material for: Pan-cancer analysis of whole genomes
Source: Nature. 2020 Feb 5;578(7793):82–93. doi: 10.1038/s41586-020-1969-6 (PMC7025898; doi:10.1038/s41586-020-1969-6)
Supplement: Supplementary file 3 — This zipped file contains Supplementary Tables 1-21 and a Supplementary Table Guide [file 41586_2020_1969_MOESM3_ESM.zip › supplementary Tables/Supplementary Table 4.docx]

**Supplementary Table 4.** *Data resources from PCAWG available to the scientific community.* Files are listed with a synapse ID (accession number), which refers to data organised on the Synapse platform (https://www.synapse.org/). In addition, every file has been mirrored at the International Cancer Genome Consortium Data Co-ordination Center (ICGC DCC), with the URL and filename reported in the table. The final column indicates whether the file is open access or is controlled tier, requiring authorisation to access it (instructions for access provided in the Data Availability statement and at https://docs.icgc.org/pcawg/data/). WGS, whole genome sequencing; SNV, single nucleotide variant; MNV, multinucleotide variant; SV, structural variant; CAN, copy number alteration; MAF, Mutation Annotation Format (described at https://docs.gdc.cancer.gov/Data/File_Formats/MAF_Format/); VCF, Variant Call Format (described at <https://www.internationalgenome.org/wiki/Analysis/vcf4.0/>); BED, Browser Extensible Data format (<https://genome.ucsc.edu/FAQ/FAQformat.html#format1>).

| Label | | | Synapse ID | DCC URL | DCC Filename | | Access |
| --- | --- | --- | --- | --- | --- | --- | --- |
| Aligned BAM Files | | |  | <https://dcc.icgc.org/pcawg> | n/a | | Control |
| Description: Sequencing reads aligned to the human reference genome for all tumour and normal samples in PCAWG. Reference genome is build hs37d5 (as used in the 1000 Genomes Project). Files are available in BAM format, and alignments were generated using the BWA algorithm. | | | | | | | |
| Final sample sheet, WGS + RNA-seq only | | |  | <http://dcc.icgc.org/releases/PCAWG/donors_and_biospecimens/> | pcawg_sample_sheet.tsv | | Open |
| Description: List of unique identifiers for sequencing files specifying whether the file contains whole genome sequencing (WGS) or RNA-sequencing data. Each row in the table records the PCAWG donor, tumour specimen and sample it derived from, noting that some patients had multiple specimens analysed. | | | | | | | |
| PCAWG sample sheet, WGS + RNA-seq + miRNA-seq | | |  | <http://dcc.icgc.org/releases/PCAWG/donors_and_biospecimens/> | pcawg-wgs-rnaseq-mirna.xlsx | | Open |
| Description: List of unique identifiers for sequencing files specifying whether the file contains WGS data, RNA-sequencing or microRNA sequencing data. Each row in the table records the PCAWG donor, tumour specimen and sample it derived from, noting that some patients had multiple specimens analysed. | | | | | | | |
| List of donors excluded for quality control issues | | |  | <http://dcc.icgc.org/releases/PCAWG/donors_and_biospecimens/> | pcawg-exclusion-list.xlsx | | Open |
| Description: List of donors who were excluded from downstream analyses because of poor quality data. Typical reasons for exclusion included contamination of the DNA with DNA or RNA from other individuals, tumour cells in the normal sample, mapping errors or high rates of 8-oxoG base call errors. | | | | | | | |
| Grey list – donors acceptable for some but not all analyses | | |  | <http://dcc.icgc.org/releases/PCAWG/donors_and_biospecimens/> | pcawg-grey-list.xlsx | | Open |
| Description: List of donors who were not excluded from downstream analyses, but had minor quality assurance concerns. Typical reasons for grey-listing included low-level contamination with DNA or RNA from other individuals, sequencing errors in the normal sample or other minor base call error profiles. | | | | | | | |
| Specimen histology and tumour subtypes | | | syn10389164 | <http://dcc.icgc.org/releases/PCAWG/clinical_and_histology/> | pcawg_specimen_histology_August2016_v9.xlsx | | Open |
| Description: The tumour subtypes were hand-curated and harmonised to ICD-0-3 organ system and histological descriptions using a semi-automated process, and grouped into tiers using a tumour subtype grouping system. This grouping system was reviewed and approved by a group of pathology experts. | | | | | | | |
| Donor clinical information | | | syn10389158 | <http://dcc.icgc.org/releases/PCAWG/clinical_and_histology/> | pcawg_donor_clinical_August2016_v9.xlsx | | Open |
| Description: Clinical data from PCAWG patients. This dataset includes information on donor demographics (age and sex); treatment, vital status and survival time; smoking history and alcohol history. Note that some of the data for some of the clinical features and risk factors are missing. | | | | | | | |
| Consensus ICGC somatic SNV, MNV, indel (MAF) | | | syn7364923 | <http://dcc.icgc.org/releases/PCAWG/consensus_snv_indel/> | final_consensus_passonly.snv_mnv_indel.icgc.maf.gz | | Open |
| Description: Somatically acquired SNVs and indels across PCAWG tumour samples contributed by projects run through ICGC. Variant calls were generated by three pipelines run independently on each sample, with subsequent merging into a consensus set of high-quality calls. The file uses MAF format. | | | | | | | |
| Consensus TCGA somatic SNV, MNV, Indel (MAF) | | | syn7364923 | <http://dcc.icgc.org/releases/PCAWG/consensus_snv_indel/> | final_consensus_passonly.snv_mnv_indel.tcga.controlled.maf.gz | | Control |
| Description: Somatically acquired SNVs and indels across PCAWG tumour samples contributed by projects run through TCGA. Variant calls were generated by three pipelines run independently on each sample, with subsequent merging into a consensus set of high-quality calls. The file uses MAF format. | | | | | | | |
| Consensus ICGC somatic SNV, indel (VCF) | | | syn7357330 | <http://dcc.icgc.org/releases/PCAWG/consensus_snv_indel/> | final_consensus_snv_indel_icgc.controlled.tgz | | Control |
| Description: Somatically acquired SNVs and indels across PCAWG tumour samples contributed by projects run through ICGC. Variant calls were generated by three pipelines run independently on each sample, with subsequent merging into a consensus set of high-quality calls. The file uses VCF format. | | | | | | | |
| Consensus TCGA somatic SNV, indel (VCF) | | | syn7357330 | <http://dcc.icgc.org/releases/PCAWG/consensus_snv_indel/> | final_consensus_snv_indel_tcga.controlled.tgz | | Control |
| Description: Somatically acquired SNVs and indels across PCAWG tumour samples contributed by projects run through TCGA. Variant calls were generated by three pipelines run independently on each sample, with subsequent merging into a consensus set of high-quality calls. The file uses VCF format. | | | | | | | |
| Consensus ICGC SVs (BED format) | | | syn7596712 | <http://dcc.icgc.org/releases/PCAWG/consensus_sv/> | final_consensus_sv_bedpe_passonly.icgc.tgz | | Open |
| Description: Somatically acquired structural variants across PCAWG tumour samples contributed by projects run through ICGC. Variant calls were generated by three pipelines run independently on each sample, with subsequent merging into a consensus set of high-quality calls. The file uses BED format. | | | | | | | |
| Consensus TCGA SVs (BED format) | | | syn7596712 | <http://dcc.icgc.org/releases/PCAWG/consensus_sv/> | final_consensus_sv_bedpe_passonly.tcga.tgz | | Open |
| Description: Somatically acquired structural variants across PCAWG tumour samples contributed by projects run through TCGA. Variant calls were generated by three pipelines run independently on each sample, with subsequent merging into a consensus set of high-quality calls. The file uses BED format. | | | | | | | |
| Consensus ICGC SVs (VCF format) | | | syn7596712 | <http://dcc.icgc.org/releases/PCAWG/consensus_sv/> | final_consensus_sv_vcfs_passonly.icgc.tgz | | Open |
| Description: Somatically acquired structural variants across PCAWG tumour samples contributed by projects run through ICGC. Variant calls were generated by three pipelines run independently on each sample, with subsequent merging into a consensus set of high-quality calls. The file uses VCF format. | | | | | | | |
| Consensus TCGA SVs (VCF format) | | | syn7596712 | <http://dcc.icgc.org/releases/PCAWG/consensus_sv/> | final_consensus_sv_vcfs_passonly.tcga.tgz | | Open |
| Description: Somatically acquired structural variants across PCAWG tumour samples contributed by projects run through TCGA. Variant calls were generated by three pipelines run independently on each sample, with subsequent merging into a consensus set of high-quality calls. The file uses VCF format. | | | | | | | |
| Consensus CNA (ICGC) | | | syn8042988 | <http://dcc.icgc.org/releases/PCAWG/consensus_cnv/> | consensus.20170119.somatic.cna.icgc.tar.gz | | Open |
| Description: Somatically acquired copy number alterations across PCAWG tumour samples contributed by projects run through ICGC. Variant calls were generated by three pipelines run independently on each sample, with subsequent merging into a consensus set of high-quality calls. | | | | | | | |
| Consensus CNA (TCGA) | | | syn8042988 | <http://dcc.icgc.org/releases/PCAWG/consensus_cnv/> | consensus.20170119.somatic.cna.tcga.tar.gz | | Open |
| Description: Somatically acquired copy number alterations across PCAWG tumour samples contributed by projects run under through TCGA. Variant calls were generated by three pipelines run independently on each sample, with subsequent merging into a consensus set of high-quality calls. | | | | | | | |
| Purity and ploidy calls | | | syn8272483 | <http://dcc.icgc.org/releases/PCAWG/consensus_cnv/> | consensus.20170217.purity.ploidy.txt.gz | | Open |
| Description: Inferred purity and ploidy calls for each patient’s tumour across PCAWG samples. Purity values represent the estimated fraction of cells in the sample derived from the tumour clone; ploidy values represent estimated average copy number in tumour cells. Format is a tab-delimited text file. | | | | | | | |
| Germline variants and ancestry proportion estimations | | | syn4877977 | <https://dcc.icgc.org/releases/PCAWG/germline_variations/> | <Multiple files> | | Open |
| Description: Germline variants of all classes, including SNVs, indels, complex short variants, structural variants and mobile element insertions. Germline variants have been phased into haplotype blocks (VCF format). Ancestry estimates also available. Data are separated into TCGA and ICGC callsets. | | | | | | | |
| Clustered mutation processes | | | syn12978907 | <https://dcc.icgc.org/releases/PCAWG/clustered_mutational_processes/> | <Multiple files> | | Control |
| Description: This folder contains files listing all the events arising from clustered mutational processes across PCAWG tumours. This includes kataegis events, chromoplexy and chromothripsis. | | | | | | | |
| Mutational signatures | syn11804065 | <https://dcc.icgc.org/releases/PCAWG/mutational_signatures/Signatures/SP_Signatures/SigProfiler_reference_signatures/SigProfiler_reference_whole-genome_signatures> | | | | sigProfiler_SBS_signatures_2019_05_22.csv | Open |
| Description: For each PCAWG sample, the fractional contribution of each signature from the consensus set of mutational signatures is reported. The same set of folders carries information on the signature weights across mutation types and local sequence context. The format is a comma-separated flat text file. | | | | | | | |
| Gene fusions | | | syn10003873 | <http://dcc.icgc.org/releases/PCAWG/transcriptome/fusion/> | gene.fusions.V1.tsv.gz | | Open |
| Description: A list of all gene fusions detected in RNA-sequencing data from across PCAWG. Events are recorded on a per-patient basis and whether the event is supported by a matching structural variant in the genome. The format is a tab-delimited flat text file. | | | | | | | |
| Joint TopHat+STAR RNA-seq FPKM expression values | | | syn5553985 | <http://dcc.icgc.org/releases/PCAWG/transcriptome/gene_expression/> | tophat_star_fpkm.v2_aliquot_gl.tsv.gz | | Open |
| Description: Expression levels of genes across PCAWG donors with RNA-sequencing data. Levels were estimated using the FPKM metric, based on alignments from the TopHat and STAR algorithms. The format is a tab-delimited flat text file. | | | | | | | |
| Joint TopHat+STAR RNA-seq FPKM UQ expression values | | | syn5553991 | <http://dcc.icgc.org/releases/PCAWG/transcriptome/gene_expression/> | tophat_star_fpkm_uq.v2_aliquot_gl.tsv.gz | | Open |
| Description: Expression levels of genes across PCAWG donors with RNA-sequencing data. Levels were estimated using the FPKM metric, based on alignments from the TopHat and STAR algorithms and normalised with the Upper Quartile (UQ) method. The format is a tab-delimited flat text file. | | | | | | | |
| GTEx gene expression derived using the PCAWG RNA-seq SOP | | | syn8105922 | <http://dcc.icgc.org/releases/PCAWG/transcriptome/gene_expression/> | GTEx.tophat2.gene.fpkm.tsv.gz | | Open |
| Description: Expression levels of genes across normal tissues generated by the GTEX consortium, and analysed using the same pipeline as applied to PCAWG cancer samples. This enables direct comparison of results from normal and cancer tissues. The format is a tab-delimited flat text file. | | | | | | | |
| RNA-seq fusion transcripts, recurrence analysis | | | syn7221157 | [http://dcc.icgc.org/releases/PCAWG/transcriptome/recurrence analyses/](http://dcc.icgc.org/releases/PCAWG/transcriptome/recurrence%20analyses/) | pcawg3_fusions_PKU_EBI.gene_centric.tsv | | Open |
| Description: Recurrence analysis of transcriptional abnormalities, including gene fusions. This file includes a sample-by-sample matrix for genes recurrently contributing to gene fusions. The format is a tab-delimited flat text file. | | | | | | | |
| Patient-centric driver catalogue (ICGC) | | | syn11639581 | <https://dcc.icgc.org/releases/PCAWG/driver_mutations> | TableS3_panorama_driver_mutations_ICGC_samples.controlled.tsv.gz | | Open |
| Description: The set of inferred driver mutations in each patient’s tumour across PCAWG samples contributed by researchers in ICGC. All classes of somatic mutation are incorporated, including SNVs, indels, somatic mutations and copy number alterations. Both somatic and pathogenic germline variants are reported. | | | | | | | |
| Patient-centric driver catalogue (TCGA) | | | syn11639581 | <https://dcc.icgc.org/releases/PCAWG/driver_mutations> | TableS3_panorama_driver_mutations_TCGA_samples.controlled.tsv.gz | | Control |
| Description: The set of inferred driver mutations in each patient’s tumour across PCAWG samples contributed by researchers in TCGA. All classes of somatic mutation are incorporated, including SNVs, indels, somatic mutations and copy number alterations. Both somatic and pathogenic germline variants are reported. | | | | | | | |
| APOBEC mutagenesis analysis (ICGC) | | | syn7437313 | <https://dcc.icgc.org/releases/PCAWG/APOBEC_mutagenesis/> | MAF_Aug31_2016_sorted_anz5.icgc.controlled.txt.gz | | Control |
| Description: The set of variants attributed to APOBEC mutagenesis in each patient’s tumour across PCAWG samples contributed by researchers in ICGC. In the same folder, files annotating likelihood of mutations arising from either APOBEC3A or APOBEC3B are available. The format is a tab-delimited flat file. | | | | | | | |
| APOBEC mutagenesis analysis (TCGA) | | | syn7437313 | <https://dcc.icgc.org/releases/PCAWG/APOBEC_mutagenesis/> | MAF_Aug31_2016_sorted_anz5.tcga.controlled.txt.gz | | Control |
| Description: The set of variants attributed to APOBEC mutagenesis in each patient’s tumour across PCAWG samples contributed by researchers in TCGA. In the same folder, files annotating likelihood of mutations arising from either APOBEC3A or APOBEC3B are available. The format is a tab-delimited flat file. | | | | | | | |
