# Supplementary material for: Pan-cancer analysis of whole genomes
Source: Nature. 2020 Feb 5;578(7793):82–93. doi: 10.1038/s41586-020-1969-6 (PMC7025898; doi:10.1038/s41586-020-1969-6)
Supplement: Supplementary file 3 — This zipped file contains Supplementary Tables 1-21 and a Supplementary Table Guide [file 41586_2020_1969_MOESM3_ESM.zip › supplementary Tables/Supplementary Table 8.docx]

**Supplementary Table 8.** Call counts resulting from running the selection procedure with a uniform requirement across concordance bins 10 times, selecting 3000 SNV calls from case DO50388.

| **concordance/**  **caller** | **1** | **2** | **3** | **4** | **5** | **6** | **7** | **total** | **Out of** |
| --- | --- | --- | --- | --- | --- | --- | --- | --- | --- |
| **smufin** | 61±0 | 159±8 | 178±7 | 212±8 | 213±8 | 422±1 | 427±0 | 1675±7 | 25796 |
| **oicr_sga** | 61±0 | 74±3 | 123±4 | 255±6 | 376±5 | 416±2 | 427±0 | 1733±6 | 28081 |
| **oicr_bl** | 61±0 | 79±3 | 85±2 | 156±11 | 328±5 | 386±4 | 427±0 | 1525±15 | 25980 |
| **wustl** | 61±0 | 208±6 | 302±5 | 393±3 | 410±2 | 422±1 | 427±0 | 2225±9 | 31401 |
| **dkfz** | 61±0 | 149±7 | 247±6 | 305±8 | 372±5 | 411±2 | 427±0 | 1974±18 | 30313 |
| **broad_mutect** | 61±0 | 117±7 | 274±5 | 309±8 | 358±6 | 398±3 | 427±0 | 1946±13 | 29619 |
| **adiscan** | 61±0 | 58±0 | 68±1 | 75±3 | 75±2 | 103±4 | 427±0 | 869±4 | 9329 |
| **average** | 61 | 121 | 183 | 244 | 305 | 366 | 427 |  |  |
