# Supplementary material for: Pan-cancer analysis of whole genomes
Source: Nature. 2020 Feb 5;578(7793):82–93. doi: 10.1038/s41586-020-1969-6 (PMC7025898; doi:10.1038/s41586-020-1969-6)
Supplement: Supplementary file 3 — This zipped file contains Supplementary Tables 1-21 and a Supplementary Table Guide [file 41586_2020_1969_MOESM3_ESM.zip › supplementary Tables/SUPPLEMENTARY TABLE GUIDE.pdf]

SUPPLEMENTARY TABLE GUIDE

Supplementary Table 1. Sample, demographic and basic mutation data for the 2,583 white-listed donors in the PCAWG data-set.

Supplementary Table 2. PCAWG germline variant call-set.

Supplementary Table 3. Major algorithms and software versions used in somatic variant calling workflow.

Supplementary Table 4. Datasets available from PCAWG analyses.

Supplementary Table 5. Likely driver mutations attributed to kataegis and the signatures responsible.

Supplementary Table 6. Patterns and distribution of chromothripsis across 108 melanoma samples in PCAWG.

Supplementary Table 7. Germline source L1 elements capable of active somatic retrotransposition.

Supplementary Table 8. Call counts resulting from running the selection procedure with a uniform requirement.

Supplementary Table 9. Cases used in the pilot, and whether or not they were also used for validation and training.

Supplementary Table 10. Distribution of read lengths.

Supplementary Table 11. Telomere-related features used to cluster tumour genomes.

Supplementary Table 12. Replicative Immortality TMM Gene List.

Supplementary Table 13. Element-specific features scored for use in OnCohortDrive.

Supplementary Table 14. Other evaluated features used in onCohortDrive.

Supplementary Table 15. Groups of coding genes according to the confidence that they drive tumorigenesis in the cancer type under analysis.

Supplementary Table 16. TCGA cohorts employed to identify GISTIC significant peaks.

Supplementary Table 17. Summary of SNV and indel callers used in the pilot evaluation.

Supplementary Table 18. Accuracies of core and consensus callers on the very low-mutation count Medulloblastoma benchmark.

Supplementary Table 19. Accuracies of core and consensus SNV callers on the cancer cell lines HCC1143 and HCC1954.

Supplementary Table 20. Percentage samples/donors run at each site for each pipeline.

Supplementary Table 21. Data distribution.
