# Supplementary material for: Pan-cancer analysis of whole genomes
Source: Nature. 2020 Feb 5;578(7793):82–93. doi: 10.1038/s41586-020-1969-6 (PMC7025898; doi:10.1038/s41586-020-1969-6)
Supplement: Supplementary file 4 — Supplementary information [file 41586_2020_1969_MOESM4_ESM.pdf]

Supplementary Information

The ICGC/TCGA Pan-Cancer Analysis of Whole Genomes Consortium

Writing Committee

Writing Committee Leads Peter J. Campbell[1,2,745]*, Gad Getz[3,4,5,6,745]*, Jan O. Korbel[7,8,745]*, Joshua M. Stuart[9,745]*, Jennifer L. Jennings[10,11] & Lincoln D. Stein[12,13,745]*

Sample collection Marc D. Perry[14,15], Hardeep K. Nahal-Bose[15] & B. F. Francis Ouellette[16,17]

Histopathology harmonization Constance H. Li[12,18], Esther Rheinbay[3,6,19], G. Petur Nielsen[19], Dennis C. Sgroi[19], Chin-Lee Wu[19], William C. Faquin[19], Vikram Deshpande[19], Paul C. Boutros[12,18,20,21], Alexander J. Lazar[22], Katherine A. Hoadley[23,24], Lincoln D. Stein[12,13] & David N. Louis[19]

Uniform processing, somatic and germline variant calling L. Jonathan Dursi[12,25], Christina K. Yung[15], Matthew H. Bailey[26,27], Gordon Saksena[3], Keiran M. Raine[1], Ivo Buchhalter[28,29,30], Kortine Kleinheinz[28,30], Matthias Schlesner[28,31], Junjun Zhang[15], Wenyi Wang[32], David A. Wheeler[33,34], Li Ding[26,27,35] & Jared T. Simpson[12,36]

Methods section 'Core alignment and variant calling by cloud computing' Christina K. Yung[15], Brian D. O'Connor[15,37], Sergei Yakneen[8], Junjun Zhang[15], Kyle Ellrott[38], Kortine Kleinheinz[28,30], Naoki Miyoshi[39], Keiran M. Raine[1], Adam P. Butler[1], Romina Royo[40], Gordon Saksena[3], Matthias Schlesner[28,31], Solomon I. Shorser[12] & Miguel Vazquez[40,41]

Methods section 'Integration, phasing and validation of germline variant call sets' Tobias Rausch[8], Grace Tiao[3], Sebastian M. Waszak[8], Bernardo Rodriguez-Martin[42,43,44], Suyash Shringarpure[45], Dai-Ying Wu[46], Sergei Yakneen[8], German M. Demidov[47,48,49], Olivier Delaneau[50,51,52], Shuto Hayashi[39], Seiya Imoto[39], Nina Habermann[8], Ayellet V. Segre[3,53], Erik Garrison[1], Andy Cafferkey[7], Eva G. Alvarez[42,43,44], José María Heredia-Genestar[54], Francesc Muyas[47,48,49], Oliver Drechsel[47,49], Alicia L. Bruzos[42,43,44], Javier Temes[42,43], Jorge Zamora[1,42,43,44], L. Jonathan Dursi[12,25], Adrian Baez-Ortega[55], Hyung-Lae Kim[56], Matthew H. Bailey[26,27], R. Jay Mashl[27,57], Kai Ye[58,59], Ivo Buchhalter[28,29,30], Anthony DiBiase[60], Kuan-lin Huang[27,61], Ivica Letunic[62], Michael D. McLellan[26,27,35], Steven J. Newhouse[7], Matthias Schlesner[28,31], Tal Shmaya[46], Sushant Kumar[63,64], David C. Wedge[1,65,66], Mark H. Wright[45], Venkata D. Yellapantula[67,68], Mark Gerstein[63,64,69], Ekta Khurana[70,71,72,73], Tomas Marques-Bonet[74,75,76,77], Arcadi Navarro[74,75,76], Carlos D. Bustamante[45,78], Jared T. Simpson[12,36], Li Ding[26,27,35], Reiner Siebert[79,80], Hidewaki Nakagawa[81], Douglas F. Easton[82,83], Stephan Ossowski[47,48,49], Jose M. C. Tubio[42,43,44], Gad Getz[3,4,5,6], Francisco M. De La Vega[45,46,78], Xavier Estivill[47,84] & Jan O. Korbel[7,8]

Methods section 'Validation, benchmarking and merging of somatic variant calls' L. Jonathan Dursi[12,25], David A. Wheeler[33,34], Christina K. Yung[15], Li Ding[26,27,35] & Jared T. Simpson[12,36]

Data and code availability Junjun Zhang[15], Christina K. Yung[15], Sergei Yakneen[8], Denis Yuen[12], George L. Mihaiescu[15], Larsson Omberg[85] & Vincent Ferretti[15,86]

Pan-cancer burden of somatic mutations Junjun Zhang[15] & Peter J. Campbell[1,2]

Panorama of driver mutations in human cancer Radhakrishnan Sabarinathan[87,88,89], Oriol Pich[87,89] & Abel Gonzalez-Perez[87,89]

PCAWG tumours with no apparent driver mutations Esther Rheinbay[3,6,19], Amaro Taylor-Weiner[90], Radhakrishnan Sabarinathan[87,88,89], Peter J. Campbell[1,2] & Gad Getz[3,4,5,6]

Patterns and oncogenicity of kataegis and chromoplexy Matthew W. Fittall[91], Jonas Demeulemeester[91,92], Maxime Tarabichi[1,91], Nicola D. Roberts[1], Peter J. Campbell[1,2], Jan O. Korbel[7,8] & Peter Van Loo[91,92]

Patterns and oncogenicity of chromothripsis Maxime Tarabichi[1,91], Jonas Demeulemeester[91,92], Matthew W. Fittall[91], Isidro Cortés-Ciriano[93,94,95], Lara Urban[7,8], Peter Park[94,95], Peter J. Campbell[1,2], Jan O. Korbel[7,8] & Peter Van Loo[91,92]

Timing-clustered mutational processes during tumour evolution Jonas Demeulemeester[91,92], Maxime Tarabichi[1,91], Matthew W. Fittall[91], Jan O. Korbel[7,8], Peter J. Campbell[1,2] & Peter Van Loo[91,92]

Germline genetic determinants of the somatic mutation landscape Sebastian M. Waszak[8], Bin Zhu[96], Bernardo Rodriguez-Martin[42,43,44], Esa Pitkänen[8], Tobias Rausch[8], Yilong Li[1], Natalie Saini[97], Leszek J. Klimczak[98], Joachim Weischenfeldt[8,99,100], Nikos Sidiropoulos[100], Ludmil B. Alexandrov[1,101], Francesc Muyas[47,48,49], Raquel Rabionet[47,49,102], Georgia Escaramis[47,103,104], Adrian Baez-Ortega[55], Mattia Bosio[40,47,49], Aliaksei Z. Holik[47], Hana Susak[47,49], Eva G. Alvarez[42,43,44], Alicia L. Bruzos[42,43,44], Javier Temes[42,43], Aparna Prasad[49], Nina Habermann[8], Serap Erkek[8], Lara Urban[7,8], Claudia Calabrese[7,8], Benjamin Raeder[8], Eoghan Harrington[105], Simon Mayes[106], Daniel Turner[106], Sissel Juul[105], Steven A. Roberts[107], Lei Song[96], Roelof Koster[108], Lisa Mirabello[96], Xing Hua[96], Tomas J. Tanskanen[109], Marta Tojo[44], David C. Wedge[1,65,66], Jorge Zamora[1,42,43,44], Jieming Chen[64,110], Lauri A. Aaltonen[111], Gunnar Rätsch[112,113,114,115,116,117], Roland F. Schwarz[7,118,119,120], Atul J. Butte[121], Alvis Brazma[7], Peter J. Campbell[1,2], Stephen J. Chanock[96], Nilanjan Chatterjee[122,123], Oliver Stegle[7,8,124], Olivier Harismendy[125], G. Steven Bova[126], Dmitry A. Gordenin[97], Jose M. C. Tubio[42,43,44], Douglas F. Easton[82,83], Xavier Estivill[47,84] & Jan O. Korbel[7,8]

Replicative immortality David Haan[9], Lina Sieverling[127,128], Lars Feuerbach[127], Lincoln D. Stein[12,13] & Joshua M. Stuart[9]

Ethical considerations of genomic cloud computing Don Chalmers[129], Yann Joly[130], Bartha Knoppers[130], Fruzsina Molnár-Gábor[131], Jan O. Korbel[7,8], Mark Phillips[130], Adrian Thorogood[130] & David Townend[130]

Online resources for data access, visualization and analysis Mary Goldman[132], Junjun Zhang[15], Nuno A. Fonseca[7,133], Qian Xiang[15], Brian Craft[132], Elena Piñeiro-Yáñez[134], Alfonso Muñoz[7], Robert Petryszak[7], Anja Füllgrabe[7], Fatima Al-Shahrour[134], Maria Keays[7], David Haussler[132,135], John Weinstein[136,137], Wolfgang Huber[8], Alfonso Valencia[40,76], Irene Papatheodorou[7], Jingchun Zhu[132], Brian D. O'Connor[15,37], Lincoln D. Stein[12,13], Alvis Brazma[7], Vincent Ferretti[15,86] & Miguel Vazquez[40,41]

Pilot-63 Validation Process L. Jonathan Dursi[12,25], Christina K. Yung[15], Matthew H. Bailey[26,27], Gordon Saksena[3], Keiran M. Raine[1], Ivo Buchhalter[28,29,30], Kortine Kleinheinz[28,30], Matthias Schlesner[28,31], Yu Fan[32], David Torrents[40,76], Matthias Bieg[138,139], Paul C. Boutros[12,18,20,21], Ken Chen[140], Zechen Chong[141], Kristian Cibulskis[3], Oliver Drechsel[47,49], Roland Eils[28,30,142,143], Robert S. Fulton[26,27,35], Josep L. Gelpi[40,144], Mark Gerstein[63,64,69], Santiago Gonzalez[7,8], Gad Getz[3,4,5,6], Ivo G. Gut[49,74], Faraz Hach[145,146], Michael Heinold[28,30], Taobo Hu[147], Vincent Huang[12], Barbara Hutter[139,148,149], Hyung-Lae Kim[56], Natalie Jäger[28], Jongsun Jung[150], Sushant Kumar[63,64], Yogesh Kumar[147], Christopher Lalansingh[12], Ignaty Leshchiner[3], Ivica Letunic[62], Dimitri Livitz[3], Eric Z. Ma[147], Yosef E. Maruvka[3,19,151], R. Jay Mashl[27,57], Michael D. McLellan[26,27,35], Ana Milovanovic[40], Morten Muhlig Nielsen[152], Brian D. O'Connor[15,37], Stephan Ossowski[47,48,49], Nagarajan Paramasivam[28,139], Jakob Skou Pedersen[152,153], Marc D. Perry[14,15], Montserrat Puiggròs[40], Romina Royo[40], Esther Rheinbay[3,6,19], S. Cenk Sahinalp[146,154,155], Iman Sarrafi[146,155], Chip Stewart[3], Miranda D. Stobbe[49,74], Grace Tiao[3], Jeremiah A. Wala[3,6,156], Jiayin Wang[27,58,157], Wenyi Wang[32], Sebastian M. Waszak[8], Joachim Weischenfeldt[8,99,100], Michael Wendl[27,158,159], Johannes Werner[28,160], Zhenggang Wu[147], Hong Xue[147], Sergei Yakneen[8], Takafumi N. Yamaguchi[12], Kai Ye[58,59], Venkata Yellapantula[67,68], Junjun Zhang[15], David A. Wheeler[33,34], Li Ding[26,27,35] & Jared T. Simpson[12,36]

Pilot-63 Processing of Validation Data Christina K. Yung[15], Brian D. O'Connor[15,37], Sergei Yakneen[8], Junjun Zhang[15], Kyle Ellrott[38], Kortine Kleinheinz[28,30], Naoki Miyoshi[39], Keiran M. Raine[1], Romina Royo[40], Gordon Saksena[3], Matthias Schlesner[28,31], Solomon I. Shorser[12], Miguel Vazquez[40,41], Joachim Weischenfeldt[8,99,100], Denis Yuen[12], Adam P. Butler[1], Brandi N. Davis-Dusenbery[161], Roland Eils[28,30,142,143], Vincent Ferretti[15,86], Robert L. Grossman[162], Olivier Harismendy[125], Youngwook Kim[163,164], Hidewaki Nakagawa[81], Steven J. Newhouse[7], David Torrents[40,76] & Lincoln D. Stein[12,13]

Whole-genome Sequencing Somatic Variant calling Junjun Zhang[15], Christina K. Yung[15] & Solomon I. Shorser[12]

Whole Genome alignment Keiran M. Raine[1], Junjun Zhang[15] & Brian D. O'Connor[15,37]

DKFZ Pipeline Kortine Kleinheinz[28,30], Tobias Rausch[8], Jan O. Korbel[7,8], Ivo Buchhalter[28,29,30], Michael C. Heinold[28,30], Barbara Hutter[139,148,149], Natalie Jäger[28], Nagarajan Paramasivam[28,139] & Matthias Schlesner[28,31]

EMBL Pipeline Joachim Weischenfeldt[8,99,100] & Tobias Rausch[8]

Sanger Pipeline Keiran M. Raine[1], Jonathan Hinton[1], David R. Jones[1], Andrew Menzies[1] & Lucy Stebbings[1]

Broad Pipeline Gordon Saksena[3], Dimitri Livitz[3], Esther Rheinbay[3,6,19], Julian M. Hess[3,151], Ignaty Leshchiner[3], Chip Stewart[3], Grace Tiao[3], Jeremiah A. Wala[3,6,156], Amaro Taylor-Weiner[90], Mara Rosenberg[3,19], Andrew J. Dunford[3], Manaswi Gupta[3], Marcin Imielinski[165,166], Matthew Meyerson[3,6,156], Rameen Beroukhim[3,6,167] & Gad Getz[3,4,5,6]

MuSE Pipeline Yu Fan[32] & Wenyi Wang[32]

Consensus Somatic SNV/indel Annotation Andrew Menzies[1], Matthias Schlesner[28,31], Jüri Reimand[12,18], Priyanka Dhingra[71,73] & Ekta Khurana[70,71,72,73]

Somatic SNV and Indel Merging L. Jonathan Dursi[12,25], Christina K. Yung[15], Matthew H. Bailey[26,27], Gordon Saksena[3], Keiran M. Raine[1], Ivo Buchhalter[28,29,30], Kortine Kleinheinz[28,30], Matthias Schlesner[28,31], Yu Fan[32], David Torrents[40,76], Matthias Bieg[138,139], Paul C. Boutros[12,18,20,21], Ken Chen[140], Zechen Chong[141], Kristian Cibulskis[3], Oliver Drechsel[47,49], Roland Eils[28,30,142,143], Robert S. Fulton[26,27,35], Josep L. Gelpi[40,144], Mark Gerstein[63,64,69], Santiago Gonzalez[7,8], Gad Getz[3,4,5,6], Ivo G. Gut[49,74], Faraz Hach[145,146], Michael Heinold[28,30], Taobo Hu[147], Vincent Huang[12], Barbara Hutter[139,148,149], Hyung-Lae Kim[56], Natalie Jäger[28], Jongsun Jung[150], Sushant Kumar[63,64], Yogesh Kumar[147], Christopher Lalansingh[12], Ignaty Leshchiner[3], Ivica Letunic[62], Dimitri Livitz[3], Eric Z. Ma[147], Yosef E. Maruvka[3,19,151], R. Jay Mashl[27,57], Michael D. McLellan[26,27,35],

Ana Milovanovic[40], Morten Muhlig Nielsen[152], Brian D. O'Connor[15,37], Stephan Ossowski[47,48,49], Nagarajan Paramasivam[28,139], Jakob Skou Pedersen[152,153], Marc D. Perry[14,15], Montserrat Puiggròs[40], Romina Royo[40], Esther Rheinbay[3,6,19], S. Cenk Sahinalp[146,154,155], Iman Sarrafi[146,155], Chip Stewart[3], Miranda D. Stobbe[49,74], Grace Tiao[3], Jeremiah A. Wala[3,6,156], Jiayin Wang[27,58,157], Wenyi Wang[32], Sebastian M. Waszak[8], Joachim Weischenfeldt[8,99,100], Michael Wendl[27,158,159], Johannes Werner[28,160], Zhenggang Wu[147], Hong Xue[147], Sergei Yakneen[8], Takafumi N. Yamaguchi[12], Kai Ye[58,59], Venkata Yellapantula[67,68], Junjun Zhang[15], David A. Wheeler[33,34], Li Ding[26,27,35] & Jared T. Simpson[12,36]

Somatic SV Merging Joachim Weischenfeldt[8,99,100], Francesco Favero[168] & Yilong Li[1]

Somatic CNA Merging Stefan Dentro[1,65,91], Jeff Wintersinger[169,170,171] & Ignaty Leshchiner[3]

Oxidative Artefact Filtration Dimitri Livitz[3], Ignaty Leshchiner[3], Chip Stewart[3], Esther Rheinbay[3,6,19], Gordon Saksena[3] & Gad Getz[3,4,5,6]

Strand Bias Filtration Matthias Bieg[138,139], Ivo Buchhalter[28,29,30], Johannes Werner[28,160] & Matthias Schlesner[28,31]

miniBAM Generation Jeremiah A. Wala[3,6,156], Gordon Saksena[3], Rameen Beroukhim[3,6,167] & Gad Getz[3,4,5,6]

Germline Variant Identification from WGS Tobias Rausch[8], Grace Tiao[3], Sebastian M. Waszak[8], Bernardo Rodriguez-Martin[42,43,44], Suyash Shringarpure[45], Dai-Ying Wu[46], Sergei Yakneen[8], German M. Demidov[47,48,49], Olivier Delaneau[50,51,52], Shuto Hayashi[39], Seiya Imoto[39], Nina Habermann[8], Ayellet V. Segre[3,53], Erik Garrison[1], Andy Cafferkey[7], Eva G. Alvarez[42,43,44], Alicia L. Bruzos[42,43,44], Jorge Zamora[1,42,43,44], José María Heredia-Genestar[54], Francesc Muyas[47,48,49], Oliver Drechsel[47,49], L. Jonathan Dursi[12,25], Adrian Baez-Ortega[55], Hyung-Lae Kim[56], Matthew H. Bailey[26,27], R. Jay Mashl[27,57], Kai Ye[58,59], Ivo Buchhalter[28,29,30], Vasilisa Rudneva[8], Ji Wan Park[172], Eun Pyo Hong[172], Seong Gu Heo[172], Anthony DiBiase[60], Kuan-lin Huang[27,61], Ivica Letunic[62], Michael D. McLellan[26,27,35], Steven J. Newhouse[7], Matthias Schlesner[28,31], Tal Shmaya[46], Sushant Kumar[63,64], David C. Wedge[1,65,66], Mark H. Wright[45], Venkata D. Yellapantula[67,68], Mark Gerstein[63,64,69], Ekta Khurana[70,71,72,73], Tomas Marques-Bonet[74,75,76,77], Arcadi Navarro[74,75,76], Carlos D. Bustamante[45,78], Jared T. Simpson[12,36], Li Ding[26,27,35], Reiner Siebert[79,80], Hidewaki Nakagawa[81], Douglas F. Easton[82,83], Stephan Ossowski[47,48,49], Jose M. C. Tubio[42,43,44], Gad Getz[3,4,5,6], Francisco M. De La Vega[45,46,78] & Xavier Estivill[47,84] & Jan O. Korbel[7,8]

RNA-seq Analysis Nuno A. Fonseca[7,133], André Kahles[112,113,114,115,116], Kjong-Van Lehmann[112,114,115,173,174], Lara Urban[7,8], Cameron M. Soulette[37], Yuichi Shiraishi[39], Fenglin Liu[175,176], Yao He[175], Deniz Demircioğlu[177,178], Natalie R. Davidson[112,114,115,117,173], Claudia Calabrese[7,8], Junjun Zhang[15], Marc D. Perry[14,15], Qian Xiang[15], Liliana Greger[7], Siliang Li[179,180], Dongbing Liu[179,180], Stefan G. Stark[115,173,181,182], Fan Zhang[175], Samirkumar B. Amin[183,184,185], Peter Bailey[186], Aurélien Chateigner[15], Isidro Cortés-Ciriano[93,94,95], Brian Craft[132], Serap Erkek[8], Milana Frenkel-Morgenstern[187], Mary Goldman[132], Katherine A. Hoadley[23,24], Yong Hou[179,180], Matthew R. Huska[118], Ekta Khurana[70,71,72,73], Helena Kilpinen[188], Jan O. Korbel[7,8], Fabien C. Lamaze[12], Chang Li[179,180], Xiaobo Li[179,180], Xinyue Li[179], Xingmin Liu[179,180], Maximillian G. Marin[37], Julia Markowski[118], Tannistha Nandi[189], Morten Muhlig Nielsen[152], Akinyemi I. Ojesina[190,191,192], Qiang Pan-Hammarström[179,193], Peter J. Park[94,95], Chandra Sekhar Pedamallu[3,6,167], Jakob Skou Pedersen[152,153], Reiner Siebert[79,80], Hong Su[179,180], Patrick Tan[189,194,195,196], Bin Tean Teh[194,195,196,197,198], Jian Wang[179], Sebastian M. Waszak[8], Heng Xiong[179,180], Sergei Yakneen[8], Chen Ye[179,180], Christina Yung[15], Xiuqing Zhang[179], Liangtao Zheng[175], Jingchun Zhu[132], Shida Zhu[179,180], Philip Awadalla[12,13], Chad J. Creighton[199], Matthew Meyerson[3,6,156], B. F. Francis Ouellette[16,17], Kui Wu[179,180], Huanming Yang[179], Jonathan Göke[177,200], Roland F. Schwarz[7,118,119,120], Oliver Stegle[7,8,124], Zemin Zhang[175,201], Alvis Brazma[7], Gunnar Rätsch[112,113,114,115,116,117] & Angela N. Brooks[3,37,156]

Clustering of Tumour Genomes Based on Telomere Maintenance-Related Features David Haan[9], Lincoln D. Stein[12,13] & Joshua M. Stuart[9]

Clustered Mutational Processes in PCAWG Jonas Demeulemeester[91,92], Maxime Tarabichi[1,91], Matthew W Fittall[91], Peter J. Campbell[1,2], Jan O. Korbel[7,8] & Peter Van Loo[91,92]

Tumours Without Detected Driver Mutations Esther Rheinbay[3,6,19], Amaro Taylor-Weiner[90], Radhakrishnan Sabarinathan[87,88,89], Peter J. Campbell[1,2] & Gad Getz[3,4,5,6]

Panorama of Driver Mutations in Human Cancer Radhakrishnan Sabarinathan[87,88,89], Oriol Pich[87,89], Iñigo Martincorena[1], Carlota Rubio-Perez[87,89,202], Malene Juul[152], Jeremiah A. Wala[3,6,156], Steven Schumacher[3,203], Ofer Shapira[3,156], Nikos Sidiropoulos[100], Sebastian M. Waszak[8], David Tamborero[87,89], Loris Mularoni[87,89], Esther Rheinbay[3,6,19], Henrik Hornshøj[152], Jordi Deu-Pons[89,204], Ferran Muiños[87,89], Johanna Bertl[152,205], Qianyun Guo[153], Chad J. Creighton[199], Joachim Weischenfeldt[8,99,100], Jan O. Korbel[7,8], Gad Getz[3,4,5,6], Peter J. Campbell[1,2], Jakob Skou Pedersen[152,153], Rameen Beroukhim[3,6,167] & Abel Gonzalez-Perez[87,89,206]

Pilot-63 Benchmarking and Validation Exercise, Production Calling and Variant Consensus Development, and Performance on Previously Validated Samples L. Jonathan Dursi[12,25], Christina K. Yung[15], Matthew H. Bailey[26,27], Gordon Saksena[3], Keiran M. Raine[1], Ivo Buchhalter[28,29,30], Kortine Kleinheinz[28,30], Matthias Schlesner[28,31], Yu Fan[32], David Torrents[40,76], Matthias Bieg[138,139], Paul C. Boutros[12,18,20,21], Ken Chen[140], Zechen Chong[141], Kristian Cibulskis[3], Oliver Drechsel[47,49], Roland Eils[28,30,142,143], Robert S. Fulton[26,27,35], Josep L. Gelpi[40,144], Mark Gerstein[63,64,69], Santiago Gonzalez[7,8], Gad Getz[3,4,5,6], Ivo G. Gut[49,74], Faraz Hach[145,146], Michael Heinold[28,30], Taobo Hu[147], Vincent Huang[12], Barbara Hutter[139,148,149], Hyung-Lae Kim[56], Natalie Jäger[28], Jongsun Jung[150], Sushant Kumar[63,64], Yogesh Kumar[147], Christopher Lalansingh[12], Ignaty Leshchiner[3], Ivica Letunic[62], Dimitri Livitz[3], Eric Z. Ma[147], Yosef E. Maruvka[3,19,151], R. Jay Mashl[27,57], Michael D. McLellan[26,27,35], Ana Milovanovic[40], Morten Muhlig Nielsen[152], Brian D. O'Connor[15,37], Stephan Ossowski[47,48,49], Nagarajan Paramasivam[28,139], Jakob Skou Pedersen[152,153], Marc D. Perry[14,15], Montserrat Puiggròs[40], Romina Royo[40], Esther Rheinbay[3,6,19], S. Cenk Sahinalp[146,154,155], Iman Sarrafi[146,155], Chip Stewart[3], Miranda D. Stobbe[49,74], Grace Tiao[3], Jeremiah A. Wala[3,6,156], Jiayin Wang[27,58,157], Wenyi Wang[32], Sebastian M. Waszak[8], Joachim Weischenfeldt[8,99,100], Michael Wendl[27,158,159], Johannes Werner[28,160], Zhenggang Wu[147], Hong Xue[147], Sergei Yakneen[8], Takafumi N. Yamaguchi[12], Kai Ye[58,59], Venkata Yellapantula[67,68], Junjun Zhang[15], David A. Wheeler[33,34], Li Ding[26,27,35] & Jared T. Simpson[12,36]

Production Somatic Variant Calling on the PCAWG Compute Cloud Christina K. Yung[15], Brian D. O'Connor[15,37], Sergei Yakneen[8], Junjun Zhang[15], Kyle Ellrott[38], Kortine Kleinheinz[28,30], Naoki Miyoshi[39], Keiran M. Raine[1], Romina Royo[40], Gordon Saksena[3], Matthias Schlesner[28,31], Solomon I. Shorser[12], Miguel Vazquez[40,41], Joachim Weischenfeldt[8,99,100], Denis Yuen[12], Adam P. Butler[1], Brandi N. Davis-Dusenbery[161], Roland Eils[28,30,142,143], Vincent Ferretti[15,86], Robert L. Grossman[162], Olivier Harismendy[125], Youngwook Kim[163,164], Hidewaki Nakagawa[81], Steven J. Newhouse[7], David Torrents[40,76] & Lincoln D. Stein[12,13]

PCAWG data portals Mary Goldman[132], Junjun Zhang[15], Nuno A. Fonseca[7,133], Isidro Cortés-Ciriano[93,94,95], Qian Xiang[207], Brian Craft[132], Elena Piñeiro-Yáñez[134], Brian D. O'Connor[15,37], Wojciech Bazant[7], Elisabet Barrera[7], Alfonso Muñoz[7], Robert Petryszak[7], Anja Füllgrabe[7], Fatima Al-Shahrour[134], Maria Keays[7], David Haussler[132,135], John Weinstein[136,137], Wolfgang Huber[8], Alfonso Valencia[40,76], Irene Papatheodorou[7], Jingchun Zhu[132], Vincent Ferretti[15,86] & Miguel Vazquez[40,41]

ICGC/TCGA Pan-Cancer Analysis of Whole Genomes Working Groups

PCAWG Steering committee Peter J. Campbell[1,2], Gad Getz[3,4,5,6], Jan O. Korbel[7,8], Lincoln D. Stein[12,13] & Joshua M. Stuart[9]

PCAWG Head of Project Management Jennifer L. Jennings[10,11]

PCAWG Executive committee Sultan T. Al-Sedairy[208], Axel Aretz[209], Cindy Bell[210], Miguel Betancourt[211], Christiane Buchholz[212], Fabien Calvo[213], Christine Chomienne[214], Michael Dunn[215], Stuart Edmonds[216], Eric Green[217], Shailja Gupta[218], Carolyn M. Hutter[217], Karine Jegalian[219], Jennifer L. Jennings[10,11], Nic Jones[220], Hyung-Lae Kim[56], Youyong Lu[221,222,223], Hitoshi Nakagama[224], Gerd Nettekoven[225], Laura Planko[225], David Scott[220], Tatsuhiro Shibata[226,227], Kiyo Shimizu[228], Lincoln D. Stein[12,13], Michael R. Stratton[1], Takashi Yugawa[228], Giampaolo Tortora[229,230], K. VijayRaghavan[218], Huanming Yang[179] & Jean C. Zenklusen[231]

PCAWG Ethics and Legal Working Group Yann Joly[130], Fruzsina Molnár-Gábor[131], Mark Phillips[130], Adrian Thorogood[130], David Townend[232], Don Chalmers[129] & Bartha M. Knoppers[130]

PCAWG Technical Working Group Brice Aminou[15], Javier Bartolome[40], Keith A. Boroevich[81,233], Rich Boyce[7], Alvis Brazma[7], Angela N. Brooks[3,37,156], Alex Buchanan[38], Ivo Buchhalter[28,29,30], Adam P. Butler[1], Niall J. Byrne[15], Andy Cafferkey[7], Peter J. Campbell[1,2], Zhaohong Chen[234], Sunghoon Cho[235], Wan Choi[236], Peter Clapham[1], Brandi N. Davis-Dusenbery[161], Francisco M. De La Vega[45,46,78], Jonas Demeulemeester[91,92], Michelle T. Dow[234], Lewis Jonathan Dursi[12,25], Juergen Eils[142,143], Roland Eils[28,30,142,143], Kyle Ellrott[38], Claudiu Farcas[234], Nodirjon Fayzullaev[15], Vincent Ferretti[15,86], Paul Flicek[7], Nuno A. Fonseca[7,133], Josep L. Gelpi[40,144], Gad Getz[3,4,5,6], Robert L. Grossman[162], Olivier Harismendy[125], Allison P. Heath[237], Michael C. Heinold[28,30], Julian M. Hess[3,151], Oliver Hofmann[238], Jongwhi H. Hong[239], Thomas J. Hudson[240,241], Barbara Hutter[139,148,149], Carolyn M. Hutter[217], Daniel Hübschmann[30,120,142,242,243], Seiya Imoto[39], Sinisa Ivkovic[244], Seung-Hyup Jeon[236], Wei Jiao[12], Jongsun Jung[150], Rolf Kabbe[28], Andre Kahles[112,113,114,115,174], Jules N. A. Kerssemakers[28], Hyung-Lae Kim[56], Hyunghwan Kim[236], Jihoon Kim[245], Youngwook Kim[163,164], Kortine Kleinheinz[28,30], Jan O. Korbel[7,8], Michael Koscher[246], Antonios Koures[234], Milena Kovacevic[244], Chris Lawerenz[143], Ignaty Leshchiner[3], Jia Liu[247], Dimitri Livitz[3], George L. Mihaiescu[15], Sanja Mijalkovic[244], Ana Mijalkovic Mijalkovic-Lazic[244], Satoru Miyano[39], Naoki Miyoshi[39], Hardeep K. Nahal-Bose[15], Hidewaki Nakagawa[81], Mia Nastic[244], Steven J. Newhouse[7], Jonathan Nicholson[1], David Ocana[7], Kazuhiro Ohi[39], Lucila Ohno-Machado[234], Larsson Omberg[85], B. F. Francis Ouellette[16,17], Nagarajan Paramasivam[28,139], Marc D. Perry[14,15], Todd D. Pihl[248], Manuel Prinz[28], Montserrat Puiggròs[40], Petar Radovic[244], Keiran M. Raine[1], Esther Rheinbay[3,6,19], Mara Rosenberg[3,19], Romina Royo[40], Gunnar Rätsch[112,113,114,115,116,117], Gordon Saksena[3], Matthias Schlesner[28,31], Solomon I. Shorser[12], Charles Short[7], Heidi J. Sofia[217], Jonathan Spring[162], Adam J. Struck[38], Grace Tiao[3], Nebojsa Tijanic[244], David Torrents[40,76], Peter Van Loo[91,92], Miguel Vazquez[40,41], David Vicente[40], Jeremiah A. Wala[3,6,156], Zhining Wang[231], Sebastian M. Waszak[8], Joachim Weischenfeldt[8,99,100], Johannes Werner[28,160], Ashley Williams[234], Youngchoon Woo[236], Adam J. Wright[12], Qian Xiang[207], Liming Yang[231], Denis Yuen[12], Brian D. O'Connor[15,37], Lincoln D. Stein[12,13], Sergei Yakneen[8], Christina K. Yung[15] & Junjun Zhang[15]

PCAWG Reference Annotations Working Group Angela N. Brooks[3,37,156], Ivo Buchhalter[28,29,30], Peter J. Campbell[1,2], Priyanka Dhingra[71,73], Lars Feuerbach[127], Mark Gerstein[63,64,69], Gad Getz[3,4,5,6], Mark P. Hamilton[249], Henrik Hornshøj[152], Todd A. Johnson[233], Andre Kahles[112,113,114,115,174], Abdullah Kahraman[250,251,252], Manolis Kellis[3,253], Jan O. Korbel[7,8], Morten Muhlig Nielsen[152], Jakob Skou Pedersen[152,153], Paz Polak[3,4,6], Jüri Reimand[12,18], Esther Rheinbay[3,6,19], Nicola D. Roberts[1], Gunnar Rätsch[112,113,114,115,116,117], Richard Sallari[3], Nasa Sinnott-Armstrong[3,45], Alfonso Valencia[40,76], Miguel Vazquez[40,41], Sebastian M. Waszak[8], Joachim Weischenfeldt[8,99,100], Christian von Mering[252,254] & Ekta Khurana[70,71,72,73]

PCAWG Quality-Control Working Group Sergi Beltran[49,74], Ivo Buchhalter[28,29,30], Peter J. Campbell[1,2], Roland Eils[28,30,142,143], Daniela S. Gerhard[255], Gad Getz[3,4,5,6], Marta Gut[49,74], Barbara Hutter[139,148,149], Daniel Hübschmann[30,120,142,242,243], Kortine Kleinheinz[28,30], Jan O. Korbel[7,8], Dimitri Livitz[3], Marc D. Perry[14,15], Keiran M. Raine[1], Esther Rheinbay[3,6,19], Mara Rosenberg[3,19], Gordon Saksena[3], Matthias Schlesner[28,31], Miranda D. Stobbe[49,74], Jean-Rémi Trotta[74], Johannes Werner[28,160], Justin P. Whalley[74] & Ivo G. Gut[49,74]

PCAWG SNV Calling Working Group Matthew H. Bailey[26,27], Beifang Niu[256], Matthias Bieg[138,139], Paul C. Boutros[12,18,20,21], Ivo Buchhalter[28,29,30], Adam P. Butler[1], Ken Chen[140], Zechen Chong[141], Oliver Drechsel[47,49], Lewis Jonathan Dursi[12,25], Roland Eils[28,30,142,143], Kyle Ellrott[38], Shadrielle M. G. Espiritu[12], Yu Fan[32], Robert S. Fulton[26,27,35], Shengjie Gao[179], Josep L. Gelpi[40,144], Mark Gerstein[63,64,69], Gad Getz[3,4,5,6], Santiago Gonzalez[7,8], Ivo G. Gut[49,74], Faraz Hach[145,146], Michael C. Heinold[28,30], Julian M. Hess[3,151], Jonathan Hinton[1], Taobo Hu[147], Vincent Huang[12], Yi Huang[157,257], Barbara Hutter[139,148,149], David R. Jones[1], Jongsun Jung[150], Natalie Jäger[28], Hyung-Lae Kim[56], Kortine Kleinheinz[28,30], Sushant Kumar[63,64], Yogesh Kumar[147], Christopher M. Lalansingh[12], Ignaty Leshchiner[3], Ivica Letunic[62], Dimitri Livitz[3], Eric Z. Ma[147], Yosef E. Maruvka[3,19,151], R. Jay Mashl[27,57], Michael D. McLellan[26,27,35], Andrew Menzies[1], Ana Milovanovic[40], Morten Muhlig Nielsen[152], Stephan Ossowski[47,48,49], Nagarajan Paramasivam[28,139], Jakob Skou Pedersen[152,153], Marc D. Perry[14,15], Montserrat Puiggròs[40], Keiran M. Raine[1], Esther Rheinbay[3,6,19], Romina Royo[40], S. Cenk Sahinalp[146,154,155], Gordon Saksena[3], Iman Sarrafi[146,155], Matthias Schlesner[28,31], Lucy Stebbings[1], Chip Stewart[3], Miranda D. Stobbe[49,74], Jon W. Teague[1], Grace Tiao[3], David Torrents[40,76], Jeremiah A. Wala[3,6,156], Jiayin Wang[27,58,157], Wenyi Wang[32], Sebastian M. Waszak[8], Joachim Weischenfeldt[8,99,100], Michael C. Wendl[27,158,159], Johannes Werner[28,160], David A. Wheeler[33,34], Zhenggang Wu[147], Hong Xue[147], Sergei Yakneen[8], Takafumi N. Yamaguchi[12], Kai Ye[58,59], Venkata D. Yellapantula[67,68], Christina K. Yung[15], Junjun Zhang[15], Li Ding[26,27,35] & Jared T. Simpson[12,36]

PCAWG Drivers and Functional Interpretation Working Group Federico Abascal[1], Samirkumar B. Amin[183,184,185], Gary D. Bader[13], Pratiti Bandopadhayay[3,258,259], Jonathan Barenboim[12], Rameen Beroukhim[3,6,167], Johanna Bertl[152,205], Keith A. Boroevich[81,233], Søren Brunak[260,261], Peter J. Campbell[1,2], Joana Carlevaro-Fita[262,263,264], Dimple Chakravarty[265,266], Calvin Wing Yiu Chan[28,128], Ken Chen[140], Jung Kyoon Choi[267], Jordi Deu-Pons[89,204], Priyanka Dhingra[71,73], Klev Diamanti[268], Lars Feuerbach[127], J. Lynn Fink[40,269], Nuno A. Fonseca[7,133], Joan Frigola[204], Carlo Gambacorti-Passerini[270], Dale W. Garsed[271], Qianyun Guo[153], Ivo G. Gut[49,74], David Haan[9], Mark P. Hamilton[249], Nicholas J. Haradhvala[3,19], Arif O. Harmanci[64,272], Mohamed Helmy[170], Carl Herrmann[28,30,273], Julian M. Hess[3,151], Asger Hobolth[153,205], Ermin Hodzic[155], Chen Hong[127,128], Henrik Hornshøj[152], Keren Isaev[12,18], Jose M. G. Izarzugaza[260], Rory Johnson[263,274], Todd A. Johnson[233], Malene Juul[152], Randi Istrup Juul[152], Andre Kahles[112,113,114,115,174], Abdullah Kahraman[250,251,252], Manolis Kellis[3,253], Ekta Khurana[70,71,72,73], Jaegil Kim[3], Jong K. Kim[275], Youngwook Kim[163,164], Jan Komorowski[268,276], Jan O. Korbel[7,8], Sushant Kumar[63,64], Andrés Lanzós[263,264,274], Erik Larsson[112], Donghoon Lee[64], Kjong-Van Lehmann[112,114,115,173,174], Shantao Li[64], Xiaotong Li[64], Ziao Lin[3,277], Eric Minwei Liu[71,73,278], Lucas Lochovsky[63,64,185], Shaoke Lou[63,64], Tobias Madsen[152], Kathleen Marchal[279,280], Iñigo Martincorena[1], Alexander Martinez-Fundichely[71,72,73], Yosef E. Maruvka[3,19,151], Patrick D. McGillivray[63], William Meyerson[64,281], Ferran Muiños[87,89], Loris Mularoni[87,89], Hidewaki Nakagawa[81], Morten Muhlig Nielsen[152], Marta Paczkowska[12], Keunchil Park[282,283], Kiejung Park[284], Tirso Pons[285], Sergio Pulido-Tamayo[279,280], Jüri Reimand[12,18], Iker Reyes-Salazar[87], Matthew A. Reyna[286], Esther Rheinbay[3,6,19], Mark A. Rubin[274,287,288,289,290], Carlota Rubio-Perez[87,89,202], S. Cenk Sahinalp[146,154,155], Gordon Saksena[3], Leonidas Salichos[63,64], Chris Sander[112,156,291,292], Steven E. Schumacher[3,203], Mark Shackleton[271], Ofer Shapira[3,156], Ciyue Shen[292,293], Raunak Shrestha[146], Shimin Shuai[12,13], Nikos Sidiropoulos[100], Lina Sieverling[127,128], Nasa Sinnott-Armstrong[3,45], Lincoln D. Stein[12,13], David Tamborero[87,89], Grace Tiao[3], Tatsuhiko Tsunoda[233,294,295,296], Husen M. Umer[268,297], Liis Uusküla-Reimand[298,299], Alfonso Valencia[40,76], Miguel Vazquez[40,41], Lieven P. C. Verbeke[280,300], Claes Wadelius[301], Lina Wadi[12], Jiayin Wang[27,58,157], Jonathan Warrell[63,64], Sebastian M. Waszak[8], Joachim Weischenfeldt[8,99,100], Guanming Wu[302], Jun Yu[303], Jing Zhang[64], Xuanping Zhang[157,304], Yan Zhang[64,305,306], Zhongming Zhao[307], Lihua Zou[308], Christian von Mering[252,254], Mark Gerstein[63,64,69], Gad Getz[3,4,5,6], Michael S. Lawrence[3,19,233], Jakob Skou Pedersen[152,153], Benjamin J. Raphael[286], Joshua M. Stuart[9] & David A. Wheeler[33,34]

PCAWG Transcriptome Working Group Samirkumar B. Amin[183,184,185], Philip Awadalla[12,13], Peter J. Bailey[186], Claudia Calabrese[7,8], Aurélien Chateigner[15], Isidro Cortés-Ciriano[93,94,95], Brian Craft[132], David Craft[3,309], Chad J. Creighton[199], Natalie R. Davidson[112,114,115,117,173], Deniz Demircioğlu[177,178], Serap Erkek[8], Nuno A. Fonseca[7,133], Milana Frenkel-Morgenstern[187], Mary J. Goldman[132], Liliana Greger[7], Jonathan Göke[177,200], Yao He[175], Katherine A. Hoadley[23,24], Yong Hou[179,180], Matthew R. Huska[118], Andre Kahles[112,113,114,115,174], Ekta Khurana[70,71,72,73], Helena Kilpinen[188], Jan O. Korbel[7,8], Fabien C. Lamaze[12], Kjong-Van Lehmann[112,114,115,173,174], Chang Li[179,180], Siliang Li[179,180], Xiaobo Li[179,180], Xinyue Li[179], Dongbing Liu[179,180], Fenglin Liu[175,176], Xingmin Liu[179,180], Maximillian G. Marin[37], Julia Markowski[118], Matthew Meyerson[3,6,156], Tannistha Nandi[189], Morten Muhlig Nielsen[152], Akinyemi I. Ojesina[190,191,192], B. F. Francis Ouellette[16,17], Qiang Pan-Hammarström[179,193], Peter J. Park[94,95], Chandra Sekhar Pedamallu[3,6,167], Jakob Skou Pedersen[152,153], Marc D. Perry[14,15], Roland F. Schwarz[7,118,119,120], Yuichi Shiraishi[39], Reiner Siebert[79,80], Cameron M. Soulette[37], Stefan G. Stark[115,173,181,182], Oliver Stegle[7,8,124], Hong Su[179,180], Patrick Tan[189,194,195,196], Bin Tean Teh[194,195,196,197,198], Lara Urban[7,8], Jian Wang[179], Sebastian M. Waszak[8], Kui Wu[179,180], Qian Xiang[207], Heng Xiong[179,180], Sergei Yakneen[8], Huanming Yang[179], Chen Ye[179,180], Christina K. Yung[15], Fan Zhang[175], Junjun Zhang[15], Xiuqing Zhang[179], Zemin Zhang[175,201], Liangtao Zheng[175], Jingchun Zhu[132], Shida Zhu[179,180], Alvis Brazma[7], Angela N. Brooks[3,37,156] & Gunnar Rätsch[112,113,114,115,116,117]

PCAWG Epigenome Working Group Hiroyuki Aburatani[310], Hans Binder[311,312], Huy Q. Dinh[313], Lars Feuerbach[127], Shengjie Gao[179], Ivo G. Gut[49,74], Simon C. Heath[49,74], Steve Hoffmann[311,312,314,315], Charles David Imbusch[127], Ekta Khurana[70,71,72,73], Helene Kretzmer[312,315], Peter W. Laird[316], Jose I. Martin-Subero[76,317], Genta Nagae[310,318], Paz Polak[3,4,6], Hui Shen[319], Reiner Siebert[79,80], Nasa Sinnott-Armstrong[3,45], Miranda D. Stobbe[49,74], Qi Wang[246], Dieter Weichenhan[320], Sergei Yakneen[8], Wanding Zhou[319], Benjamin P. Berman[313,321,322], Benedikt Brors[127,149,323] & Christoph Plass[320]

PCAWG Structural Variation Working Group Kadir C. Akdemir[140], Eva G. Alvarez[42,43,44], Adrian Baez-Ortega[55], Paul C. Boutros[12,18,20,21], David D. L. Bowtell[271], Benedikt Brors[127,149,323], Kathleen H. Burns[324,325], John Busanovich[3,326], Kin Chan[327], Ken Chen[140], Isidro Cortés-Ciriano[93,94,95], Ana Dueso-Barroso[40], Andrew J. Dunford[3], Paul A. Edwards[328,329], Xavier Estivill[47,84], Dariush Etemadmoghadam[271], Lars Feuerbach[127], J. Lynn Fink[40,269], Milana Frenkel-Morgenstern[187], Dale W. Garsed[271], Mark Gerstein[63,64,69], Dmitry A. Gordenin[97], David Haan[9], James E. Haber[330], Julian M. Hess[3,151], Barbara Hutter[139,148,149], Marcin Imielinski[165,166], David T. W. Jones[331,332], Young Seok Ju[1,267], Marat D. Kazanov[333,334,335], Leszek J. Klimczak[98], Youngil Koh[336,337], Jan O. Korbel[7,8], Kiran Kumar[3], Eunjung Alice Lee[338], Jake June-Koo Lee[94,95], Yilong Li[1], Andy G. Lynch[328,329,339], Geoff Macintyre[328], Florian Markowetz[328,329], Iñigo Martincorena[1], Alexander Martinez-Fundichely[71,72,73], Matthew Meyerson[3,6,156], Satoru Miyano[39], Hidewaki Nakagawa[81], Fabio C. P. Navarro[63], Stephan Ossowski[47,48,49], Peter J. Park[94,95], John V. Pearson[340,341], Montserrat Puiggròs[40], Karsten Rippe[120], Nicola D. Roberts[1], Steven A. Roberts[107], Bernardo Rodriguez-Martin[42,43,44], Steven E. Schumacher[3,203], Ralph Scully[342], Mark Shackleton[271], Nikos Sidiropoulos[100], Lina Sieverling[127,128], Chip Stewart[3], David Torrents[40,76], Jose M. C. Tubio[42,43,44], Izar Villasante[40], Nicola Waddell[340,341], Jeremiah A. Wala[3,6,156], Joachim Weischenfeldt[8,99,100], Lixing Yang[343], Xiaotong Yao[165,344], Sung-Soo Yoon[337], Jorge Zamora[1,42,43,44], Cheng-Zhong Zhang[3,6,156], Rameen Beroukhim[3,6,167] & Peter J. Campbell[1,2]

PCAWG Mutational Signatures Working Group Ludmil B. Alexandrov[1,101], Erik N. Bergstrom[345], Arnoud Boot[195,346], Paul C. Boutros[12,18,20,21], Kin Chan[327], Kyle Covington[34], Akihiro Fujimoto[81], Gad Getz[3,4,5,6], Dmitry A. Gordenin[97], Nicholas J. Haradhvala[3,19], Mi Ni Huang[195,346], S. M. Ashiqul Islam[101], Marat D. Kazanov[333,334,335], Jaegil Kim[3], Leszek J. Klimczak[98], Michael S. Lawrence[3,19,233], Iñigo Martincorena[1], John R. McPherson[195,346], Sandro Morganella[1], Ville Mustonen[347,348,349], Hidewaki Nakagawa[81], Alvin Wei Tian Ng[350], Paz Polak[3,4,6], Stephenie D. Prokopec[12], Steven A. Roberts[107], Radhakrishnan Sabarinathan[87,88,89], Natalie Saini[97], Tatsuhiro Shibata[226,227], Yuichi Shiraishi[39], Ignacio Vázquez-García[1,67,351,352], Yang Wu[195,346], Fouad Yousif[12], Willie Yu[353], Steven G. Rozen[195,196,346], Michael R. Stratton[1] & Bin Tean Teh[194,195,196,197,198]

PCAWG Germline Cancer Genome Working Group Ludmil B. Alexandrov[1,101], Eva G. Alvarez[42,43,44], Adrian Baez-Ortega[55], Matthew H. Bailey[26,27], Mattia Bosio[40,47,49], G. Steven Bova[126], Alvis Brazma[7], Alicia L. Bruzos[42,43,44], Ivo Buchhalter[28,29,30], Carlos D. Bustamante[45,78], Atul J. Butte[121], Andy Cafferkey[7], Claudia Calabrese[7,8], Peter J. Campbell[1,2], Stephen J. Chanock[96], Nilanjan Chatterjee[122,123], Jieming Chen[64,110], Francisco M. De La Vega[45,46,78], Olivier Delaneau[50,51,52], German M. Demidov[47,48,49], Anthony DiBiase[60], Li Ding[26,27,35], Oliver Drechsel[47,49], Lewis Jonathan Dursi[12,25], Douglas F. Easton[82,83], Serap Erkek[8], Georgia Escaramis[47,103,104], Erik Garrison[1], Mark Gerstein[63,64,69], Gad Getz[3,4,5,6], Dmitry A. Gordenin[97], Nina Habermann[8], Olivier Harismendy[125], Eoghan Harrington[105], Shuto Hayashi[39], Seong Gu Heo[172], José María Heredia-Genestar[54], Aliaksei Z. Holik[47], Eun Pyo Hong[172], Xing Hua[96], Kuan-lin Huang[27,61], Seiya Imoto[39], Sissel Juul[105], Ekta Khurana[70,71,72,73], Hyung-Lae Kim[56], Youngwook Kim[163,164], Leszek J. Klimczak[98], Roelof Koster[108], Sushant Kumar[63,64], Ivica Letunic[62], Yilong Li[1], Tomas Marques-Bonet[74,75,76,77], R. Jay Mashl[27,57], Simon Mayes[106], Michael D. McLellan[26,27,35], Lisa Mirabello[96], Francesc Muyas[47,48,49], Hidewaki Nakagawa[81], Arcadi Navarro[74,75,76], Steven J. Newhouse[7], Stephan Ossowski[47,48,49], Ji Wan Park[172], Esa Pitkänen[8], Aparna Prasad[49], Raquel Rabionet[47,49,102], Benjamin Raeder[8], Tobias Rausch[8], Steven A. Roberts[107], Bernardo Rodriguez-Martin[42,43,44], Vasilisa A. Rudneva[8], Gunnar Rätsch[112,113,114,115,116,117], Natalie Saini[97], Matthias Schlesner[28,31], Roland F. Schwarz[7,118,119,120], Ayellet V. Segre[3,53], Tal Shmaya[46], Suyash S. Shringarpure[45], Nikos Sidiropoulos[100], Reiner Siebert[79,80], Jared T. Simpson[12,36], Lei Song[96], Oliver Stegle[7,8,124], Hana Susak[47,49], Tomas J. Tanskanen[109], Grace Tiao[3], Marta Tojo[44], Jose M. C. Tubio[42,43,44], Daniel J. Turner[106], Lara Urban[7,8], Sebastian M. Waszak[8], David C. Wedge[1,65,66], Joachim Weischenfeldt[8,99,100], David A. Wheeler[33,34], Mark H. Wright[45], Dai-Ying Wu[46], Tian Xia[354], Sergei Yakneen[8], Kai Ye[58,59], Venkata D. Yellapantula[67,68], Jorge Zamora[1,42,43,44], Bin Zhu[96], Xavier Estivill[47,84] & Jan O. Korbel[7,8]

PCAWG Pathology and Clinical Correlates Working Group Fatima Al-Shahrour[134], Gurnit Atwal[12,13,171], Peter J. Bailey[186], Paul C. Boutros[12,18,20,21], Peter J. Campbell[1,2], David K. Chang[186,355], Susanna L. Cooke[186], Vikram Deshpande[19], Bishoy M. Faltas[117], William C. Faquin[19], Gad Getz[3,4,5,6], Syed Haider[12], Wei Jiao[12], Vera B. Kaiser[356], Rosa Karlić[357], Mamoru Kato[358], Kirsten Kübler[3,6,19], Alexander J. Lazar[22], Constance H. Li[12,18], David N. Louis[19], Adam Margolin[38], Sancha Martin[1,359], Hardeep K. Nahal-Bose[15], G. Petur Nielsen[19], Serena Nik-Zainal[1,360,361,362],

Larsson Omberg[85], Christine P'ng[12], Marc D. Perry[14,15], Paz Polak[3,4,6], Esther Rheinbay[3,6,19], Mark A. Rubin[274,287,288,289,290], Colin A. Semple[356], Dennis C. Sgroi[19], Tatsuhiro Shibata[226,227], Reiner Siebert[79,80], Jaclyn Smith[38], Miranda D. Stobbe[49,74], Ren X. Sun[12], Kevin Thai[15], Derek W. Wright[363,364], Chin-Lee Wu[19], Ke Yuan[328,359,365], Junjun Zhang[15], Andrew V. Biankin[186,355,366,367], Levi Garraway[156], Sean M. Grimmond[368], Katherine A. Hoadley[23,24] & Lincoln D. Stein[12,13]

PCAWG Evolution & Heterogeneity Working Group David J. Adams[1], Pavana Anur[369], Rameen Beroukhim[3,6,167], Paul C. Boutros[12,18,20,21], David D. L. Bowtell[271], Peter J. Campbell[1,2], Shaolong Cao[32], Elizabeth L. Christie[271], Marek Cmero[370,371,372], Yupeng Cun[373], Kevin J. Dawson[1], Jonas Demeulemeester[91,92], Stefan C. Dentro[1,65,91], Amit G. Deshwar[374], Nilgun Donmez[146,155], Ruben M. Drews[328], Roland Eils[28,30,142,143], Yu Fan[32], Matthew W. Fittall[91], Dale W. Garsed[271], Moritz Gerstung[7,8], Gad Getz[3,4,5,6], Santiago Gonzalez[7,8], Gavin Ha[3], Kerstin Haase[91], Marcin Imielinski[165,166], Lara Jerman[8,375], Yuan Ji[376,377], Clemency Jolly[91], Kortine Kleinheinz[28,30], Juhee Lee[378], Henry Lee-Six[1], Ignaty Leshchiner[3], Dimitri Livitz[3], Geoff Macintyre[328], Salem Malikic[146,155], Florian Markowetz[328,329], Iñigo Martincorena[1], Thomas J. Mitchell[1,329,379], Quaid D. Morris[171,380], Ville Mustonen[347,348,349], Layla Oesper[381], Martin Peifer[373], Myron Peto[382], Benjamin J. Raphael[286], Daniel Rosebrock[3], Yulia Rubanova[36,171], S. Cenk Sahinalp[146,154,155], Adriana Salcedo[12], Matthias Schlesner[28,31], Steven E. Schumacher[3,203], Subhajit Sengupta[383], Ruian Shi[380], Seung Jun Shin[182], Oliver Spiro[3], Lincoln D. Stein[12,13], Maxime Tarabichi[1,91], Shankar Vembu[380,384], Ignacio Vázquez-García[1,67,351,352], Wenyi Wang[32], David A. Wheeler[33,34], Jeffrey A. Wintersinger[169,170,171], Tsun-Po Yang[373], Xiaotong Yao[165,344], Kaixian Yu[385], Ke Yuan[328,359,365], Hongtu Zhu[386,387], Paul T. Spellman[388], David C. Wedge[1,65,66] & Peter Van Loo[91,92]

PCAWG Portals and Visualization Working Group Fatima Al-Shahrour[134], Elisabet Barrera[7], Wojciech Bazant[7], Alvis Brazma[7], Isidro Cortés-Ciriano[93,94,95], Brian Craft[132], David Craft[3,309], Vincent Ferretti[15,86], Nuno A. Fonseca[7,133], Anja Füllgrabe[7], Mary J. Goldman[132], Wolfgang Huber[8], Maria Keays[7], Alfonso Muñoz[7], Brian D. O'Connor[15,37], Irene Papatheodorou[7], Robert Petryszak[7], Elena Piñeiro-Yáñez[134], Alfonso Valencia[40,76], John N. Weinstein[136,137], Qian Xiang[207], Junjun Zhang[15], David Haussler[132,135], Miguel Vazquez[40,41] & Jingchun Zhu[132]

PCAWG Mitochondrial Genome and Immunogenomics Working Group Peter J. Campbell[1,2], Yiwen Chen[32], Chad J. Creighton[199], Li Ding[26,27,35], Akihiro Fujimoto[81], Masashi Fujita[81], Gad Getz[3,4,5,6], Leng Han[304], Takanori Hasegawa[39], Shuto Hayashi[39], Seiya Imoto[39], Young Seok Ju[1,267], Hyung-Lae Kim[56], Youngwook Kim[163,164], Youngil Koh[336,337], Mitsuhiro Komura[39], Jun Li[32], Iñigo Martincorena[1], Satoru Miyano[39], Shinichi Mizuno[389], Keunchil Park[282,283], Eigo Shimizu[39], Yumeng Wang[32,390], John N. Weinstein[136,137], Yanxun Xu[391], Rui Yamaguchi[39], Fan Yang[380], Yang Yang[304], Christopher J. Yoon[267], Sung-Soo Yoon[337], Yuan Yuan[32], Fan Zhang[175], Zemin Zhang[175,201], Han Liang[32] & Hidewaki Nakagawa[81]

PCAWG Pathogens Working Group Malik Alawi[392,393], Ivan Borozan[12], Daniel S. Brewer[394,395], Colin S. Cooper[395,396,397], Nikita Desai[15], Roland Eils[28,30,142,143], Vincent Ferretti[15,86], Adam Grundhoff[392,398], Murat Iskar[399], Kortine Kleinheinz[28,30], Hidewaki Nakagawa[81], Akinyemi I. Ojesina[190,191,192], Chandra Sekhar Pedamallu[3,6,167], Matthias Schlesner[28,31], Xiaoping Su[400], Marc Zapatka[399] & Peter Lichter[148,399]

Providers of tumour-sequencing data

Tumour-specific providers (ovarian cancer) in Australia Kathryn Alsop[271], Timothy J. C. Bruxner[269], Angelika N. Christ[269], Elizabeth L. Christie[271], Stephen M. Cordner[401], Prue A. Cowin[402], Ronny Drapkin[403], Dariush Etemadmoghadam[271], Sian Fereday[402], Dale W. Garsed[271], Joshy George[185], Sean M. Grimmond[368], Anne Hamilton[402], Oliver Holmes[340,341], Jillian A. Hung[404,405], Karin S. Kassahn[269,406], Stephen H. Kazakoff[340,341], Catherine J. Kennedy[407,408], Conrad R. Leonard[340,341], Linda Mileshkin[271], David K. Miller[269,355,409], Gisela Mir Arnau[402], Chris Mitchell[402], Felicity Newell[340,341], Katia Nones[340,341], Ann-Marie Patch[340,341], John V. Pearson[340,341], Michael C. Quinn[340,341], Mark Shackleton[271], Darrin F. Taylor[269], Heather Thorne[402], Nadia Traficante[402], Ravikiran Vedururu[402], Nick M. Waddell[341], Nicola Waddell[340,341], Paul M. Waring[410], Scott Wood[340,341], Qinying Xu[340,341], Anna deFazio[411,412,413] & David D. L. Bowtell[271]

Tumour-specific providers (pancreatic cancer) in Australia Matthew J. Anderson[269], Davide Antonello[414], Andrew P. Barbour[415,416], Claudio Bassi[414], Samantha Bersani[417], Timothy J. C. Bruxner[269], Ivana Cataldo[417,418], David K. Chang[186,355], Lorraine A. Chantrill[355,419], Yoke-Eng Chiew[411], Angela Chou[355,420], Angelika N. Christ[269], Sara Cingarlini[229], Nicole Cloonan[421], Vincenzo Corbo[418,422], Maria Vittoria Davi[423], Fraser R. Duthie[186,424], J. Lynn Fink[40,269], Anthony J. Gill[355,420], Janet S. Graham[186,425], Ivon Harliwong[269], Oliver Holmes[340,341], Nigel B. Jamieson[186,367,426], Amber L. Johns[355,409], Karin S. Kassahn[269,406], Stephen H. Kazakoff[340,341], James G. Kench[355,420,427], Luca Landoni[414], Rita T. Lawlor[418], Conrad R. Leonard[340,341], Andrea Mafficini[418], Neil D. Merrett[414,428], David K. Miller[269,355,409], Marco Miotto[414], Elizabeth A. Musgrove[186], Adnan M. Nagrial[355], Felicity Newell[340,341], Katia Nones[340,341], Karin A. Oien[410,429], Marina Pajic[355], Ann-Marie Patch[340,341], John V. Pearson[340,341], Mark Pinese[430], Michael C. Quinn[340,341], Alan J. Robertson[269], Ilse Rooman[355], Borislav C. Rusev[418], Jaswinder S. Samra[414,420], Maria Scardoni[417], Christopher J. Scarlett[355,431], Aldo Scarpa[418], Elisabetta Sereni[414], Katarzyna O. Sikora[418], Michele Simbolo[422], Morgan L. Taschuk[15], Christopher W. Toon[355], Giampaolo Tortora[229,230], Caterina Vicentini[418], Nick M. Waddell[341], Nicola Waddell[340,341], Scott Wood[340,341], Jianmin Wu[355], Qinying Xu[340,341], Nikolajs Zeps[432,433], Andrew V. Biankin[186,355,366,367] & Sean M. Grimmond[368]

Tumour-specific providers (skin cancer) in Australia Lauri A. Aaltonen[111], Andreas Behren[434], Hazel Burke[435], Jonathan Cebon[434], Rebecca A. Dagg[436], Ricardo De Paoli-Iseppi[437], Ken Dutton-Regester[340], Matthew A. Field[438], Anna Fitzgerald[439], Sean M. Grimmond[368], Peter Hersey[435], Oliver Holmes[340,341], Valerie Jakrot[435], Peter A. Johansson[340], Hojabr Kakavand[437], Stephen H. Kazakoff[340,341], Richard F. Kefford[440], Loretta M. S. Lau[441], Conrad R. Leonard[340,341], Georgina V. Long[442], Felicity Newell[340,341], Katia Nones[340,341], Ann-Marie Patch[340,341], John V. Pearson[340,341], Hilda A. Pickett[441], Antonia L. Pritchard[340], Gulietta M. Pupo[443], Robyn P. M. Saw[442], Sarah-Jane Schramm[444], Mark Shackleton[271], Catherine A. Shang[439], Ping Shang[442], Andrew J. Spillane[442], Jonathan R. Stretch[442], Varsha Tembe[411,444], John F. Thompson[442], Ricardo E. Vilain[445], Nick M. Waddell[341], Nicola Waddell[340,341], James S. Wilmott[442], Scott Wood[340,341], Qinying Xu[340,341], Jean Y. Yang[446], Nicholas K. Hayward[340,435], Graham J. Mann[411,447] & Richard A. Scolyer[412,442,445,448]

Tumour-specific providers (pancreatic cancer) in Canada John Bartlett[449,450], Prashant Bavi[451], Ivan Borozan[12], Dianne E. Chadwick[452], Michelle Chan-Seng-Yue[451], Sean Cleary[451,453], Ashton A. Connor[453,454], Karolina Czajka[241], Robert E. Denroche[451], Neesha C. Dhani[455], Jenna Eagles[241], Vincent Ferretti[15,86], Steven Gallinger[451,453,454], Robert C. Grant[451,454], David Hedley[455], Michael A. Hollingsworth[456], Gun Ho Jang[451], Jeremy Johns[241], Sangeetha Kalimuthu[451], Sheng-Ben Liang[457], Ilinca Lungu[451,458], Xuemei Luo[12], Faridah Mbabaali[241], Treasa A. McPherson[454], Jessica K. Miller[241], Malcolm J. Moore[455], Faiyaz Notta[451,459], Danielle Pasternack[241], Gloria M. Petersen[460], Michael H. A. Roehrl[18,451,461,462,463], Michelle Sam[241], Iris Selander[454], Stefano Serra[410], Sagedeh Shahabi[457], Morgan L. Taschuk[15], Sarah P. Thayer[456], Lee E. Timms[241], Gavin W. Wilson[12,451], Julie M. Wilson[451], Bradly G. Wouters[464], Thomas J. Hudson[240,241], John D. McPherson[241,451,465] & Lincoln D. Stein[12,13]

Tumour-specific providers (prostate cancer) in Canada Timothy A. Beck[15,466], Vinayak Bhandari[12], Colin C. Collins[146], Shadrielle M. G. Espiritu[12], Neil E. Fleshner[467], Natalie S. Fox[12], Michael Fraser[12], Syed Haider[12], Lawrence E. Heisler[468], Vincent Huang[12], Emilie Lalonde[12], Julie Livingstone[12], John D. McPherson[241,451,465], Alice Meng[469], Veronica Y. Sabelnykova[12], Adriana Salcedo[12], Yu-Jia Shiah[12], Theodorus Van der Kwast[470], Takafumi N. Yamaguchi[12], Paul C. Boutros[12,18,20,21] & Robert G. Bristow[18,471,472,473,474]

Tumour-specific providers (gastric cancer) in China Shuai Ding[475], Daiming Fan[476], Yong Hou[179,180], Yi Huang[157,257], Lin Li[179], Siliang Li[179,180], Dongbing Liu[179,180], Xingmin Liu[179,180], Yongzhan Nie[476,477], Hong Su[179,180], Jian Wang[179], Kui Wu[179,180], Xiao Xiao[157], Rui Xing[222,478], Shanlin Yang[475], Yingyan Yu[479], Xiuqing Zhang[179], Yong Zhou[179], Shida Zhu[179,180], Youyong Lu[221,222,223] & Huanming Yang[179]

Tumour-specific providers (renal cancer) in the EU and France Rosamonde E. Banks[480], Guillaume Bourque[481,482], Alvis Brazma[7], Paul Brennan[483], Louis Letourneau[484], Yasser Riazalhosseini[482], Ghislaine Scelo[483], Naveen Vasudev[480,485], Juris Viksna[486], Mark Lathrop[482] & Jörg Tost[487]

Tumour-specific providers (breast cancer) in the EU & United Kingdom Sung-Min Ahn[488], Ludmil B. Alexandrov[1,101], Samuel Aparicio[489], Laurent Arnould[490], M. R. Aure[491], Shriram G. Bhosle[1], Ewan Birney[7], Ake Borg[492], Sandrine Boyault[493], Arie B. Brinkman[494], Jane E. Brock[495], Annegien Broeks[496], Adam P. Butler[1], Anne-Lise Børresen-Dale[491], Carlos Caldas[497,498], Peter J. Campbell[1,2], Suet-Feung Chin[497,498], Helen Davies[1,360,361], Christine Desmedt[499,500], Luc Dirix[501], Serge Dronov[1], Anna Ehinger[502], Jorunn E. Eyfjord[503], Aquila Fatima[203], John A. Foekens[504], P. Andrew Futreal[505], Øystein Garred[506,507], Moritz Gerstung[7,8], Dilip D. Giri[508], Dominik Glodzik[1], Dorthe Grabau[509], Holmfridur Hilmarsdottir[503], Gerrit K. Hooijer[510], Jocelyne Jacquemier[511], Se Jin Jang[512], Jon G. Jonasson[503], Jos Jonkers[513], Hyung-Yong Kim[511], Tari A. King[514,515], Stian Knappskog[1,516], Gu Kong[511], Savitri Krishnamurthy[517], Sunil R. Lakhani[518], Anita Langerød[491], Denis Larsimont[519], Hee Jin Lee[512], Jeong-Yeon Lee[520], Ming Ta Michael Lee[505], Yilong Li[1], Ole Christian Lingjærde[521], Gaetan MacGrogan[522], John W. M. Martens[504], Sancha Martin[1,359], Iñigo Martincorena[1], Andrew Menzies[1], Sandro Morganella[1], Ville Mustonen[347,348,349], Serena Nik-Zainal[1,360,361,362], Sarah O'Meara[1], Iris Pauporté[214], Sarah Pinder[523], Xavier Pivot[524], Elena Provenzano[525], Colin A. Purdie[526], Keiran M. Raine[1], Manasa Ramakrishna[1], Kamna Ramakrishnan[1], Jorge Reis-Filho[508], Andrea L. Richardson[203], Markus Ringnér[492], Javier Bartolomé Rodriguez[40], F. Germán Rodríguez-González[261], Gilles Romieu[527], Roberto Salgado[410], Torill Sauer[521], Rebecca Shepherd[1], Anieta M. Sieuwerts[504], Peter T. Simpson[518], Marcel Smid[504], Christos Sotiriou[234], Paul N. Span[528], Lucy Stebbings[1], Ólafur Andri Stefánsson[529], Alasdair Stenhouse[530], Henk G. Stunnenberg[180,531], Fred Sweep[532], Benita Kiat Tee Tan[533], Jon W. Teague[1], Gilles Thomas[534], Alastair M. Thompson[530], Stefania Tommasi[535], Isabelle Treilleux[536,537], Andrew Tutt[203], Naoto T. Ueno[387], Steven Van Laere[501], Peter Van Loo[91,92], Gert G. Van den Eynden[501], Peter Vermeulen[501], Alain Viari[418], Anne Vincent-Salomon[531], David C. Wedge[1,65,66], Bernice H. Wong[538], Lucy Yates[1], Xueqing Zou[1], Carolien H. M. van Deurzen[539], Marc J. van de Vijver[410], Laura van't Veer[540] & Michael R. Stratton[1]

Tumour-specific providers (malignant lymphoma) in Germany Ole Ammerpohl[541,542,543], Sietse Aukema[542,543,544], Anke K. Bergmann[545], Stephan H. Bernhart[311,312,315], Hans Binder[311,312], Arndt Borkhardt[546], Christoph Borst[547], Benedikt Brors[127,149,323], Birgit Burkhardt[548], Alexander Claviez[549], Roland Eils[28,30,142,143], Maria Elisabeth Goebler[550], Andrea Haake[541], Siegfried Haas[547], Martin Hansmann[551], Jessica I. Hoell[546], Steve Hoffmann[311,312,314,315], Michael Hummel[552], Daniel Hübschmann[30,120,142,242,243], Dennis Karsch[553], Wolfram Klapper[544], Kortine Kleinheinz[28,30], Michael Kneba[553], Jan O. Korbel[7,8], Helene Kretzmer[312,315], Markus Kreuz[554], Dieter Kube[555], Ralf Küppers[556], Chris Lawerenz[143], Dido Lenze[552], Peter Lichter[148,399], Markus Loeffler[554], Cristina López[80,541], Luisa Mantovani-Löffler[557], Peter Möller[558], German Ott[559], Bernhard Radlwimmer[399], Julia Richter[541,544], Marius Rohde[560], Philip C. Rosenstiel[561], Andreas Rosenwald[562],

Markus B. Schilhabel[561], Matthias Schlesner[28,31], Stefan Schreiber[563], Peter F. Stadler[311,312,315], Peter Staib[564], Stephan Stilgenbauer[565], Stephanie Sungalee[8], Monika Szczepanowski[544], Umut H. Toprak[30,566], Lorenz H. P. Trümper[555], Rabea Wagener[80,541], Thorsten Zenz[149] & Reiner Siebert[79,80]

Tumour-specific providers (paediatric brain cancer) in Germany Ivo Buchhalter[28,29,30], Juergen Eils[142,143], Roland Eils[28,30,142,143], Volker Hovestadt[399], Barbara Hutter[139,148,149], David T. W. Jones[331,332], Natalie Jäger[28], Christof von Kalle[120], Marcel Kool[246,331], Jan O. Korbel[7,8], Andrey Korshunov[246], Pablo Landgraf[567,568], Chris Lawerenz[143], Hans Lehrach[569], Paul A. Northcott[570], Stefan M. Pfister[246,331,571], Bernhard Radlwimmer[399], Guido Reifenberger[568], Matthias Schlesner[28,31], Hans-Jörg Warnatz[569], Joachim Weischenfeldt[8,99,100], Stephan Wolf[572], Marie-Laure Yaspo[569], Marc Zapatka[399] & Peter Lichter[148,399]

Tumour-specific providers (prostate cancer) in Germany Yassen Assenov[573], Benedikt Brors[127,149,323], Juergen Eils[142,143], Roland Eils[28,30,142,143], Lars Feuerbach[127], Clarissa Gerhauser[320], Jan O. Korbel[7,8], Chris Lawerenz[143], Hans Lehrach[569], Sarah Minner[574], Christoph Plass[320], Thorsten Schlomm[99,575], Nikos Sidiropoulos[100], Ronald Simon[576], Hans-Jörg Warnatz[569], Dieter Weichenhan[320], Joachim Weischenfeldt[8,99,100], Marie-Laure Yaspo[569], Guido Sauter[576] & Holger Sültmann[149,577]

Tumour-specific providers (oral cancer) in India Nidhan K. Biswas[578], Luca Landoni[414], Arindam Maitra[578], Partha P. Majumder[578] & Rajiv Sarin[579]

Tumour-specific providers (pancreatic cancer) in Italy Davide Antonello[414], Stefano Barbi[422], Claudio Bassi[414], Samantha Bersani[417], Giada Bonizzato[418], Cinzia Cantù[418], Ivana Cataldo[417,418], Sara Cingarlini[229], Vincenzo Corbo[418,422], Maria Vittoria Davi[423], Angelo P. Dei Tos[580], Matteo Fassan[581], Sonia Grimaldi[418], Luca Landoni[414], Rita T. Lawlor[418], Claudio Luchini[417], Andrea Mafficini[418], Giuseppe Malleo[414], Giovanni Marchegiani[414], Michele Milella[229], Marco Miotto[414], Salvatore Paiella[414], Antonio Pea[414], Paolo Pederzoli[414], Borislav C. Rusev[418], Andrea Ruzzenente[414], Roberto Salvia[414], Maria Scardoni[417], Elisabetta Sereni[414], Michele Simbolo[422], Nicola Sperandio[418], Giampaolo Tortora[229,230], Caterina Vicentini[418] & Aldo Scarpa[418]

Tumour-specific providers (biliary tract cancer) in Japan Yasuhito Arai[226], Natsuko Hama[226], Nobuyoshi Hiraoka[582], Fumie Hosoda[226], Mamoru Kato[358], Hiromi Nakamura[226], Hidenori Ojima[583], Takuji Okusaka[584], Yasushi Totoki[226], Tomoko Urushidate[227] & Tatsuhiro Shibata[226,227]

Tumour-specific providers (gastric cancer) in Japan Yasuhito Arai[226], Masashi Fukayama[585], Natsuko Hama[226], Fumie Hosoda[226], Shumpei Ishikawa[586], Hitoshi Katai[587], Mamoru Kato[358], Hiroto Katoh[586], Daisuke Komura[586], Genta Nagae[310,318], Hiromi Nakamura[226], Hirofumi Rokutan[358], Mihoko Saito-Adachi[358], Akihiro Suzuki[310,588], Hirokazu Taniguchi[589], Kenji Tatsuno[310], Yasushi Totoki[226], Tetsuo Ushiku[585], Shinichi Yachida[226,590], Shogo Yamamoto[310], Hiroyuki Aburatani[310] & Tatsuhiro Shibata[226,227]

Tumour-specific providers (liver cancer) in Japan Hiroyuki Aburatani[310], Hiroshi Aikata[591], Koji Arihiro[591], Shun-ichi Ariizumi[592], Keith A. Boroevich[81,233], Kazuaki Chayama[591], Akihiro Fujimoto[81], Masashi Fujita[81], Mayuko Furuta[81], Kunihito Gotoh[593], Natsuko Hama[226], Takanori Hasegawa[39], Shinya Hayami[594], Shuto Hayashi[39], Satoshi Hirano[595], Seiya Imoto[39], Mamoru Kato[358], Yoshiiku Kawakami[591], Kazuhiro Maejima[81], Satoru Miyano[39], Genta Nagae[310,318], Hiromi Nakamura[226], Toru Nakamura[595], Kaoru Nakano[81], Hideki Ohdan[591], Aya Sasaki-Oku[81], Yuichi Shiraishi[39], Hiroko Tanaka[39], Yasushi Totoki[226], Tatsuhiko Tsunoda[233,294,295,296], Masaki Ueno[594], Rui Yamaguchi[39], Masakazu Yamamoto[592], Hiroki Yamaue[594], Hidewaki Nakagawa[81] & Tatsuhiro Shibata[226,227]

Tumour-specific providers (biliary tract cancer) in Singapore Su Pin Choo[596], Ioana Cutcutache[195,346], Narong Khuntikeo[414,597], John R. McPherson[195,346], Choon Kiat Ong[598], Chawalit Pairojkul[410], Irinel Popescu[599], Steven G. Rozen[195,196,346], Patrick Tan[189,194,195,196] & Bin Tean Teh[194,195,196,197,198]

Tumour-specific providers (blood cancer) in South Korea Keun Soo Ahn[600], Hyung-Lae Kim[56], Youngil Koh[336,337] & Sung-Soo Yoon[337]

Tumour-specific providers (chronic lymphocytic leukaemia) in Spain Marta Aymerich[601], Josep L. Gelpi[40,144], Ivo G. Gut[49,74], Marta Gut[49,74], Armando Lopez-Guillermo[602], Carlos López-Otín[603], Xose S. Puente[603], Romina Royo[40], David Torrents[40,76] & Elias Campo[604,605]

Tumour-specific providers (bone cancer) in the United Kingdom Fernanda Amary[606], Daniel Baumhoer[607], Sam Behjati[1], Bodil Bjerkehagen[607,608], P. A. Futreal[505], Ola Myklebost[516], Nischalan Pillay[609], Patrick Tarpey[610], Roberto Tirabosco[611], Olga Zaikova[612], Peter J. Campbell[1,2] & Adrienne M. Flanagan[613]

Tumour-specific providers (chronic myeloid disorders) in the United Kingdom Jacqueline Boultwood[614], David T. Bowen[1], Adam P. Butler[1], Mario Cazzola[615], Carlo Gambacorti-Passerini[270], Anthony R. Green[329], Eva Hellstrom-Lindberg[616], Luca Malcovati[615], Sancha Martin[1,359], Jyoti Nangalia[617], Elli Papaemmanuil[1], Paresh Vyas[340,618] & Peter J. Campbell[1,2]

Tumour-specific providers (oesophageal cancer) in the United Kingdom Yeng Ang[619], Hugh Barr[620], Duncan Beardsmore[621], Matthew Eldridge[328], James Gossage[622], Nicola Grehan[361], George B. Hanna[623], Stephen J. Hayes[624,625], Ted R. Hupp[626], David Khoo[627], Jesper Lagergren[616,628], Laurence B. Lovat[188], Shona MacRae[136], Maria O'Donovan[361], J. Robert O'Neill[629], Simon L. Parsons[630], Shaun R. Preston[631], Sonia Puig[632], Tom Roques[633], Grant Sanders[24], Sharmila Sothi[634], Simon Tavaré[328], Olga Tucker[635], Richard Turkington[636], Timothy J. Underwood[637], Ian Welch[638] & Rebecca C. Fitzgerald[361]

Tumour-specific providers (prostate cancer) in the United Kingdom Daniel M. Berney[639], Johann S. De Bono[396], G. Steven Bova[126], Daniel S. Brewer[394,395], Adam P. Butler[1], Declan Cahill[640], Niedzica Camacho[396], Nening M. Dennis[640], Tim Dudderidge[640,641], Sandra E. Edwards[396], Cyril Fisher[640], Christopher S. Foster[642,643], Mohammed Ghori[1], Pelvender Gill[618], Vincent J. Gnanapragasam[379,644], Gunes Gundem[278], Freddie C. Hamdy[645], Steve Hawkins[328], Steven Hazell[640], William Howat[379], William B. Isaacs[646], Katalin Karaszi[618], Jonathan D. Kay[188], Vincent Khoo[640], Zsofia Kote-Jarai[396], Barbara Kremeyer[1], Pardeep Kumar[640], Adam Lambert[618], Daniel A. Leongamornlert[1,396], Naomi Livni[640], Yong-Jie Lu[639,647], Hayley J. Luxton[188], Andy G. Lynch[328,329,339], Luke Marsden[618], Charlie E. Massie[328], Lucy Matthews[396], Erik Mayer[640,648], Ultan McDermott[1], Sue Merson[396], Thomas J. Mitchell[1,329,379], David E. Neal[328,379], Anthony Ng[649], David Nicol[640], Christopher Ogden[640], Edward W. Rowe[640], Nimish C. Shah[379], Jon W. Teague[1], Sarah Thomas[640], Alan Thompson[640], Peter Van Loo[91,92], Clare Verrill[618,650], Tapio Visakorpi[126], Anne Y. Warren[379,651], David C. Wedge[1,65,66], Hayley C. Whitaker[188], Jorge Zamora[1,42,43,44], Hongwei Zhang[647], Nicholas van As[640], Colin S. Cooper[395,396,397] & Rosalind A. Eeles[396,640]

Tumour-specific providers (TCGA) in the United States Adam Abeshouse[278], Nishant Agrawal[162], Rehan Akbani[361,652], Hikmat Al-Ahmadie[278], Monique Albert[450], Kenneth Aldape[400,653], Adrian Ally[654], Yeng Ang[619], Elizabeth L. Appelbaum[27,188], Joshua Armenia[655], Sylvia Asa[630,656], J. Todd Auman[657], Matthew H. Bailey[26,27], Miruna Balasundaram[654], Saianand Balu[24], Jill Barnholtz-Sloan[658,659], Hugh Barr[620], John Bartlett[449,450], Oliver F. Bathe[660,661], Stephen B. Baylin[123,641], Duncan Beardsmore[621], Christopher Benz[662], Andrew Berchuck[663], Benjamin P. Berman[313,321,322], Rameen Beroukhim[3,6,167], Mario Berrios[664], Darell Bigner[665], Michael Birrer[19], Tom Bodenheimer[24], Lori Boice[632], Moiz S. Bootwalla[664], Marcus Bosenberg[666], Reanne Bowlby[654], Jeffrey Boyd[667], Russell R. Broaddus[400], Malcolm Brock[668], Denise Brooks[654], Susan Bullman[3,167], Samantha J. Caesar-Johnson[231], Thomas E. Carey[669], Rebecca Carlsen[654], Robert Cerfolio[670], Vishal S. Chandan[671], Hsiao-Wei Chen[619,655], Andrew D. Cherniack[3,156,167], Jeremy Chien[672], Juok Cho[3], Eric Chuah[654], Carrie Cibulskis[3], Kristian Cibulskis[3], Leslie Cope[673], Matthew G. Cordes[27,633], Kyle Covington[34], Erin Curley[674], Bogdan Czerniak[400,627], Ludmila Danilova[673], Ian J. Davis[675], Timothy Defreitas[3], John A. Demchok[231], Noreen Dhalla[654], Rajiv Dhir[676], Li Ding[26,27,35], HarshaVardhan Doddapaneni[34], Adel El-Naggar[400,627], Ina Felau[231], Martin L. Ferguson[677], Gaetano Finocchiaro[678], Kwun M. Fong[679], Scott Frazer[3], William Friedman[680], Catrina C. Fronick[27,633], Lucinda A. Fulton[27], Robert S. Fulton[26,27,35], Stacey B. Gabriel[3], Jianjiong Gao[655], Nils Gehlenborg[3,681], Jeffrey E. Gershenwald[682,683], Gad Getz[3,4,5,6], Ronald Ghossein[508], Nasra H. Giama[684], Richard A. Gibbs[34], Carmen Gomez[685], James Gossage[622], Ramaswamy Govindan[26], Nicola Grehan[361], George B. Hanna[623], D. Neil Hayes[24,686,687], Stephen J. Hayes[624,625], Apurva M. Hegde[136,137], David I. Heiman[3], Zachary Heins[278], Austin J. Hepperla[24], Katherine A. Hoadley[23,24], Andrea Holbrook[664], Robert A. Holt[654], Alan P. Hoyle[24], Ralph H. Hruban[673], Jianhong Hu[34], Mei Huang[632], David Huntsman[688], Ted R. Hupp[626], Jason Huse[278], Christine A. Iacobuzio-Donahue[508], Michael Ittmann[689,690], Joy C. Jayaseelan[34], Stuart R. Jefferys[24], Corbin D. Jones[691], Steven J. M. Jones[692], Hartmut Juhl[693], Koo Jeong Kang[694], Beth Karlan[695], Katayoon Kasaian[692], Electron Kebebew[696,697], David Khoo[627], Hark Kyun Kim[698], Jaegil Kim[3], Tari A. King[514,515], Viktoriya Korchina[34], Ritika Kundra[619,655], Jesper Lagergren[616,628], Phillip H. Lai[664], Peter W. Laird[316], Eric Lander[3], Michael S. Lawrence[3,19,233], Alexander J. Lazar[22], Xuan Le[699], Darlene Lee[654], Douglas A. Levine[278,700], Lora Lewis[34], Tim Ley[701], Haiyan Irene Li[654], Pei Lin[3], W. M. Linehan[702], Eric Minwei Liu[71,73,278], Fei Fei Liu[380], Laurence B. Lovat[188], Yiling Lu[137], Lisa Lype[703], Yussanne Ma[654], Shona MacRae[136], Dennis T. Maglinte[664,704], Elaine R. Mardis[27,667,705], Jeffrey Marks[414,706], Marco A. Marra[654], Thomas J. Matthew[37], Michael Mayo[654], Karen McCune[707], Michael D. McLellan[26,27,35], Samuel R. Meier[3], Shaowu Meng[24], Matthew Meyerson[3,6,156], Piotr A. Mieczkowski[23], Tom Mikkelsen[708], Christopher A. Miller[27], Gordon B. Mills[709], Richard A. Moore[654], Carl Morrison[410,710], Lisle E. Mose[24], Catherine D. Moser[684], Andrew J. Mungall[654], Karen Mungall[654], David Mutch[711], Donna M. Muzny[712], Jerome Myers[713], Yulia Newton[37], Michael S. Noble[3], Peter O'Donnell[714], Brian Patrick O'Neill[715], Angelica Ochoa[278], Akinyemi I. Ojesina[190,191,192], Joong-Won Park[716], Joel S. Parker[717], Simon L. Parsons[630], Harvey Pass[718], Alessandro Pastore[112], Chandra Sekhar Pedamallu[3,6,167], Nathan A. Pennell[719], Charles M. Perou[720], Gloria M. Petersen[460], Nicholas Petrelli[721], Olga Potapova[722], Shaun R. Preston[631], Sonia Puig[632], Janet S. Rader[723], Suresh Ramalingam[724], W. Kimryn Rathmell[725], Victor Reuter[508], Sheila M. Reynolds[703], Matthew Ringel[726], Jeffrey Roach[727], Lewis R. Roberts[684], A. Gordon Robertson[654], Tom Roques[633], Mark A. Rubin[274,287,288,289,290], Sara Sadeghi[654], Gordon Saksena[3], Charles Saller[728], Francisco Sanchez-Vega[619,655], Chris Sander[112,156,291,292], Grant Sanders[24], Dirk Schadendorf[148,729], Jacqueline E. Schein[654], Heather K. Schmidt[27], Nikolaus Schultz[655], Steven E. Schumacher[3,203], Richard A. Scolyer[412,442,445,448], Raja Seethala[730], Yasin Senbabaoglu[112], Troy Shelton[674], Yan Shi[24], Juliann Shih[3,167], Ilya Shmulevich[703], Craig Shriver[731], Sabina Signoretti[167,263,732], Janae V. Simons[24], Samuel Singer[414,733], Payal Sipahimalani[654], Tara J. Skelly[23], Karen Smith-McCune[707], Nicholas D. Socci[112], Heidi J. Sofia[217], Matthew G. Soloway[717], Anil K. Sood[734], Sharmila Sothi[634], Angela Tam[654], Donghui Tan[23], Roy Tarnuzzer[231], Nina Thiessen[654], R. Houston Thompson[735], Leigh B. Thorne[632], Ming Tsao[630,656], Olga Tucker[635], Richard Turkington[636], Christopher Umbricht[324,621,736], Timothy J. Underwood[637], David J. Van Den Berg[664], Erwin G. Van Meir[737], Umadevi Veluvolu[23], Douglas Voet[3], Jiayin Wang[27,58,157], Linghua Wang[34], Zhining Wang[231], Paul Weinberger[738], John N. Weinstein[136,137], Daniel J. Weisenberger[664], Ian Welch[638], David A. Wheeler[33,34], Dennis Wigle[739], Matthew D. Wilkerson[23], Richard K. Wilson[27,740], Boris Winterhoff[741], Maciej Wiznerowicz[742,743], Tina Wong[27,654], Winghing Wong[744], Liu Xi[34], Liming Yang[231], Christina Yau[662], Venkata D. Yellapantula[67,68], Hailei Zhang[3], Hongxin Zhang[655], Jiashan Zhang[231], Carolyn M. Hutter[217] & Jean C. Zenklusen[231]

[1]Wellcome Sanger Institute, Hinxton, UK. [2]Department of Haematology, University of Cambridge, Cambridge, UK. [3]Broad Institute of MIT and Harvard, Cambridge, MA, USA. [4]Center for Cancer Research, Massachusetts General Hospital, Boston, MA, USA. [5]Department of Pathology, Massachusetts General Hospital, Boston, MA, USA. [6]Harvard Medical School, Boston, MA, USA. [7]European Molecular Biology Laboratory (EMBL), European Bioinformatics Institute (EMBL-EBI), Hinxton, UK. [8]European Molecular Biology Laboratory (EMBL), Genome Biology Unit, Heidelberg, Germany. [9]Biomolecular Engineering Department, University of California Santa Cruz, Santa Cruz, CA, USA. [10]Adaptive Oncology Initiative, Ontario Institute for Cancer Research, Toronto, Ontario, Canada. [11]International Cancer Genome Consortium (ICGC)/ICGC Accelerating Research in Genomic Oncology (ICGC-ARGO) Secretariat, Toronto, Ontario, Canada. [12]Computational Biology Program, Ontario Institute for Cancer Research, Toronto, Ontario, Canada. [13]Department of Molecular Genetics, University of Toronto, Toronto, Ontario, Canada. [14]Department of Radiation Oncology, University of California San Francisco, San Francisco, CA, USA. [15]Genome Informatics Program, Ontario Institute for Cancer Research, Toronto, Ontario, Canada. [16]Department of Cell and Systems Biology, University of Toronto, Toronto, Ontario, Canada. [17]Genome Informatics, Ontario Institute for Cancer Research, Toronto, Ontario, Canada. [18]Department of Medical Biophysics, University of Toronto, Toronto, Ontario, Canada. [19]Massachusetts General Hospital, Boston, MA, USA. [20]Department of Pharmacology, University of Toronto, Toronto, Ontario, Canada. [21]University of California Los Angeles, Los Angeles, CA, USA. [22]Department of Pathology, Department of Genomic Medicine and Department of Translational Molecular Pathology, The University of Texas MD Anderson Cancer Center, Houston, TX, USA. [23]Department of Genetics, University of North Carolina at Chapel Hill, Chapel Hill, NC, USA. [24]Lineberger Comprehensive Cancer Center, University of North Carolina at Chapel Hill, Chapel Hill, NC, USA. [25]The Hospital for Sick Children, Toronto, Ontario, Canada. [26]Alvin J. Siteman Cancer Center, Washington University School of Medicine, St Louis, MO, USA. [27]The McDonnell Genome Institute, Washington University, St Louis, MO, USA. [28]Division of Theoretical Bioinformatics, German Cancer Research Center (DKFZ), Heidelberg, Germany. [29]Heidelberg Center for Personalized Oncology (DKFZ-HIPO), German Cancer Research Center, Heidelberg, Germany. [30]Institute of Pharmacy and Molecular Biotechnology, and BioQuant, Heidelberg University, Heidelberg, Germany. [31]Bioinformatics and Omics Data Analytics, German Cancer Research Center (DKFZ), Heidelberg, Germany. [32]Department of Bioinformatics and Computational Biology, The University of Texas MD Anderson Cancer Center, Houston, TX, USA. [33]Department of Molecular and Human Genetics, Baylor College of Medicine, Houston, TX, USA. [34]Human Genome Sequencing Center, Baylor College of Medicine, Houston, TX, USA. [35]Department of Genetics and Department of Medicine, Washington University in St Louis, St Louis, MO, USA. [36]Department of Computer Science, University of Toronto, Toronto, Ontario, Canada. [37]University of California Santa Cruz, Santa Cruz, CA, USA. [38]Computational Biology Program, Oregon Health & Science University, Portland, OR, USA. [39]The Institute of Medical Science, The University of Tokyo, Tokyo, Japan. [40]Barcelona Supercomputing Center (BSC), Barcelona, Spain. [41]Department of Clinical and Molecular Medicine, Faculty of Medicine and Health Sciences, Norwegian University of Science and Technology, Trondheim, Norway. [42]Centre for Research in Molecular Medicine and Chronic Diseases (CiMUS), Universidade de Santiago de Compostela, Santiago de Compostela, Spain. [43]Department of Zoology, Genetics and Physical Anthropology, Centre for Research in Molecular Medicine and Chronic Diseases (CiMUS), Universidade de Santiago de Compostela, Santiago de Compostela, Spain. [44]The Biomedical Research Centre (CINBIO), Universidade de Vigo, Vigo, Spain. [45]Department of Genetics, Stanford University School of Medicine, Stanford, CA, USA. [46]Annai Systems, Carlsbad, CA, USA. [47]Centre for Genomic Regulation (CRG), The Barcelona Institute of Science and Technology (BIST), Barcelona, Spain. [48]Institute of Medical Genetics and Applied Genomics, University of Tübingen, Tübingen, Germany. [49]Universitat Pompeu Fabra (UPF), Barcelona, Spain. [50]Department of Computational Biology, University of Lausanne, Lausanne, Switzerland. [51]Department of Genetic Medicine and Development, University of Geneva Medical School, Geneva, Switzerland. [52]Swiss Institute of Bioinformatics, University of Geneva, Geneva, Switzerland. [53]Department of Ophthalmology, Ocular Genomics Institute, Massachusetts Eye and Ear, Harvard Medical School, Boston, MA, USA. [54]Department of Experimental and Health Sciences, Institute of Evolutionary Biology (UPF-CSIC), Universitat Pompeu Fabra (UPF), Barcelona, Spain. [55]Department of Veterinary Medicine, Transmissible Cancer Group, University of Cambridge, Cambridge, UK. [56]Department of Biochemistry, College of Medicine, Ewha Womans University, Seoul, South Korea. [57]Division of Oncology, Washington University School of Medicine, St Louis, MO, USA. [58]School of Electronic and Information Engineering, Xi'an Jiaotong University, Xi'an, China. [59]The First Affiliated Hospital, Xi'an Jiaotong University, Xi'an, China. [60]Independent Consultant, Wellesley, MA, USA. [61]Icahn School of Medicine at Mount Sinai, New York, NY, USA. [62]Biobyte Solutions, Heidelberg, Germany. [63]Department of Molecular Biophysics and Biochemistry, Yale University, New Haven, CT, USA. [64]Program in Computational Biology and Bioinformatics, Yale University, New Haven, CT, USA. [65]Big Data Institute, Li Ka Shing Centre, University of Oxford, Oxford, UK. [66]Oxford NIHR Biomedical Research Centre, University of Oxford, Oxford, UK. [67]Department of Epidemiology and Biostatistics, Memorial Sloan Kettering Cancer Center, New York, NY, USA. [68]The McDonnell Genome Institute at Washington University School of Medicine, and Department of Genetics and Department of Medicine, Siteman Cancer Center, Washington University in St Louis, St Louis, MO, USA. [69]Department of Computer Science, Yale University, New Haven, CT, USA. [70]Sandra and Edward Meyer Cancer Center, Weill Cornell Medicine, New York, NY, USA. [71]Department of Physiology and Biophysics, Weill Cornell Medicine, New York, NY, USA. [72]Englander Institute for Precision Medicine, Weill Cornell Medicine, New York, NY, USA. [73]Institute for Computational Biomedicine, Weill Cornell Medicine, New York, NY, USA. [74]CNAG-CRG, Centre for Genomic Regulation (CRG), Barcelona Institute of Science and Technology (BIST), Barcelona, Spain. [75]Department of Experimental and Health Sciences, Institute of Evolutionary Biology (UPF-CSIC), Universitat Pompeu Fabra (UPF), Barcelona, Spain. [76]Institució Catalana de Recerca i Estudis Avançats (ICREA), Barcelona, Spain. [77]Institut Català de Paleontologia Miquel Crusafont, Universitat Autònoma de Barcelona, Barcelona, Spain. [78]Department of Biomedical Data Science, Stanford University School of Medicine, Stanford, CA, USA. [79]Human Genetics, University of Kiel, Kiel, Germany. [80]Institute of Human Genetics, Ulm University and Ulm University Medical Center, Ulm, Germany. [81]RIKEN Center for Integrative Medical Sciences, Yokohama, Japan. [82]Department of Oncology, Centre for Cancer Genetic Epidemiology, University of Cambridge, Cambridge, UK. [83]Department of Public Health and Primary Care, Centre for Cancer Genetic Epidemiology, University of Cambridge, Cambridge, UK. [84]Quantitative Genomics Laboratories (qGenomics), Barcelona, Spain. [85]Sage Bionetworks, Seattle, WA, USA. [86]Department of Biochemistry and Molecular Medicine, University of Montreal, Montreal, Quebec, Canada. [87]Institute for Research in Biomedicine (IRB Barcelona), Barcelona, Spain. [88]National Centre for Biological Sciences, Tata Institute of Fundamental Research, Bangalore, India. [89]Research Program on Biomedical Informatics, Universitat Pompeu Fabra (UPF), Barcelona, Spain. [90]Broad Institute of Harvard and MIT, Cambridge, MA, USA. [91]The Francis Crick Institute, London, UK. [92]University of Leuven, Leuven, Belgium. [93]Centre for Molecular Science Informatics, Department of Chemistry, University of Cambridge, Cambridge, UK. [94]Department of Biomedical Informatics, Harvard Medical School, Boston, MA, USA. [95]Ludwig Center at Harvard Medical School, Boston, MA, USA. [96]Division of Cancer Epidemiology and Genetics, National Cancer Institute, National Institutes of Health, Bethesda, MD, USA. [97]Genome Integrity and Structural Biology Laboratory, National Institute of Environmental Health Sciences (NIEHS), Durham, NC, USA. [98]Integrative Bioinformatics Support Group, National Institute of Environmental Health Sciences (NIEHS), Durham, NC, USA. [99]Department of Urology, Charité Universitätsmedizin Berlin, Berlin, Germany. [100]Finsen Laboratory and Biotech Research & Innovation Centre (BRIC), University of Copenhagen, Copenhagen, Denmark. [101]Department of Bioengineering and Department of Cellular and Molecular Medicine, Moores Cancer Center, University of California San Diego, La Jolla, CA, USA. [102]Department of Genetics, Microbiology and Statistics, University of Barcelona, IRSJD, IBUB, Barcelona, Spain. [103]CIBER Epidemiología y Salud Pública (CIBERESP), Madrid, Spain. [104]Research Group on Statistics, Econometrics and Health (GRECS), UdG, Barcelona, Spain. [105]Oxford Nanopore Technologies, New York, NY, USA. [106]Applications Department, Oxford Nanopore Technologies, Oxford, UK. [107]School of Molecular Biosciences and Center for Reproductive Biology, Washington State University, Pullman, WA, USA. [108]Laboratory of Translational Genomics, Division of Cancer Epidemiology and Genetics, National Cancer Institute, National Institutes of Health, Bethesda, MD, USA. [109]Department of Medical and Clinical Genetics, Genome-Scale Biology Research Program, University of Helsinki, Helsinki, Finland. [110]Integrated Graduate Program in Physical and Engineering Biology, Yale University, New Haven, CT, USA. [111]Applied Tumor Genomics Research Program, Research Programs Unit, University of Helsinki, Helsinki, Finland. [112]Computational Biology Center, Memorial Sloan Kettering Cancer Center, New York, NY, USA. [113]Department of Biology, ETH Zurich, Zurich, Switzerland. [114]Department of Computer Science, ETH Zurich, Zurich, Switzerland. [115]SIB Swiss Institute of Bioinformatics, Lausanne, Switzerland. [116]University Hospital Zurich, Zurich, Switzerland. [117]Weill Cornell Medical College, New York, NY, USA. [118]Berlin Institute for Medical Systems Biology, Max Delbrück Center for Molecular Medicine, Berlin, Germany. [119]German Cancer Consortium (DKTK), Partner site Berlin, Berlin, Germany. [120]German Cancer Research Center (DKFZ), Heidelberg, Germany. [121]Bakar Computational Health Sciences Institute and Department of Pediatrics, University of California, San Francisco, CA, USA. [122]Department of Biostatistics, Bloomberg School of Public Health, Johns Hopkins University, Baltimore, MD, USA. [123]Department of Oncology, The Johns Hopkins School of Medicine, The Sidney Kimmel Comprehensive Cancer Center at Johns Hopkins University, Baltimore, MD, USA. [124]Division of Computational Genomics and Systems Genetics, German Cancer Research Center (DKFZ), Heidelberg, Germany. [125]Department of Medicine and Moores Cancer Center, Division of Biomedical Informatics, UC San Diego School of Medicine, San Diego, CA, USA. [126]Faculty of Medicine and Health Technology, Tampere University and Tays Cancer Center, Tampere University Hospital, Tampere, Finland. [127]Division of Applied Bioinformatics, German Cancer Research Center (DKFZ), Heidelberg, Germany. [128]Faculty of Biosciences, Heidelberg University, Heidelberg, Germany. [129]Centre for Law and Genetics, University of Tasmania, Hobart, Tasmania, Australia. [130]Centre of Genomics and Policy, McGill University and Génome Québec Innovation Centre, Montreal, Quebec, Canada. [131]Heidelberg Academy of Sciences and Humanities, Heidelberg, Germany. [132]UC Santa Cruz Genomics Institute, University of California Santa Cruz, Santa Cruz, CA, USA. [133]CIBIO/InBIO, Research Center in Biodiversity and Genetic Resources, Universidade do Porto, Vairão, Portugal. [134]Bioinformatics Unit, Spanish National Cancer Research Centre (CNIO), Madrid, Spain. [135]Howard Hughes Medical Institute, University of California Santa Cruz, Santa Cruz, CA, USA. [136]Cancer Unit, MRC University of Cambridge, Cambridge, UK. [137]Department of Bioinformatics and Computational Biology and Department of Systems Biology, The University of Texas MD Anderson Cancer Center, Houston, TX, USA. [138]Center for Digital Health, Berlin Institute of Health (BIH) and Charitè–Universitätsmedizin Berlin, Berlin, Germany. [139]Heidelberg Center for Personalized Oncology (DKFZ-HIPO), German Cancer Research Center (DKFZ), Heidelberg, Germany. [140]Department of Bioinformatics and Computational Biology, University of Texas MD Anderson Cancer Center, Houston, TX, USA. [141]Department of Genetics and Informatics Institute, University of Alabama at Birmingham,

Birmingham, AL, USA. [142]Heidelberg University, Heidelberg, Germany. [143]New BIH Digital Health Center, Berlin Institute of Health (BIH) and Charité–Universitätsmedizin Berlin, Berlin, Germany. [144]Department of Biochemistry and Molecular Biomedicine, University of Barcelona, Barcelona, Spain. [145]Department of Urologic Sciences, University of British Columbia, Vancouver, British Columbia, Canada. [146]Vancouver Prostate Centre, Vancouver, British Columbia, Canada. [147]Division of Life Science and Applied Genomics Center, Hong Kong University of Science and Technology, Hong Kong, China. [148]German Cancer Consortium (DKTK), Heidelberg, Germany. [149]National Center for Tumor Diseases (NCT) Heidelberg, Heidelberg, Germany. [150]Genome Integration Data Center, Syntekabio, Daejon, South Korea. [151]Massachusetts General Hospital Center for Cancer Research, Charlestown, MA, USA. [152]Department of Molecular Medicine (MOMA), Aarhus University Hospital, Aarhus, Denmark. [153]Bioinformatics Research Centre (BiRC), Aarhus University, Aarhus, Denmark. [154]Indiana University, Bloomington, IN, USA. [155]Simon Fraser University, Burnaby, British Columbia, Canada. [156]Dana-Farber Cancer Institute, Boston, MA, USA. [157]School of Computer Science and Technology, Xi'an Jiaotong University, Xi'an, China. [158]Department of Genetics, Washington University School of Medicine, St Louis, MO, USA. [159]Department of Mathematics, Washington University in St Louis, St Louis, MO, USA. [160]Department of Biological Oceanography, Leibniz Institute of Baltic Sea Research, Rostock, Germany. [161]Seven Bridges Genomics, Charlestown, MA, USA. [162]University of Chicago, Chicago, IL, USA. [163]Department of Health Sciences and Technology, Sungkyunkwan University School of Medicine, Seoul, South Korea. [164]Samsung Genome Institute, Seoul, South Korea. [165]New York Genome Center, New York, NY, USA. [166]Weill Cornell Medicine, New York, NY, USA. [167]Department of Medical Oncology, Dana-Farber Cancer Institute, Boston, MA, USA. [168]Rigshospitalet, Copenhagen, Denmark. [169]Department of Computer Science, University of Toronto, Toronto, Ontario, Canada. [170]The Donnelly Centre, University of Toronto, Toronto, Ontario, Canada. [171]Vector Institute, Toronto, Ontario, Canada. [172]Department of Medical Genetics, College of Medicine, Hallym University, Chuncheon, South Korea. [173]Department of Biology, ETH Zurich, Zurich, Switzerland. [174]University Hospital Zurich, Zurich, Switzerland. [175]Peking University, Beijing, China. [176]School of Life Sciences, Peking University, Beijing, China. [177]Computational and Systems Biology, Genome Institute of Singapore, Singapore, Singapore. [178]School of Computing, National University of Singapore, Singapore, Singapore. [179]BGI-Shenzhen, Shenzhen, China. [180]China National GeneBank-Shenzhen, Shenzhen, China. [181]Computational & Systems Biology Program, Memorial Sloan Kettering Cancer Center, New York, NY, USA. [182]Korea University, Seoul, South Korea. [183]Department of Genomic Medicine, The University of Texas MD Anderson Cancer Center, Houston, TX, USA. [184]Quantitative & Computational Biosciences Graduate Program, Baylor College of Medicine, Houston, TX, USA. [185]The Jackson Laboratory for Genomic Medicine, Farmington, CT, USA. [186]Wolfson Wohl Cancer Research Centre, Institute of Cancer Sciences, University of Glasgow, Bearsden, UK. [187]The Azrieli Faculty of Medicine, Bar-Ilan University, Safed, Israel. [188]University College London, London, UK. [189]Genome Institute of Singapore, Singapore, Singapore. [190]Department of Epidemiology, University of Alabama at Birmingham, Birmingham, AL, USA. [191]HudsonAlpha Institute for Biotechnology, Huntsville, AL, USA. [192]O'Neal Comprehensive Cancer Center, University of Alabama at Birmingham, Birmingham, AL, USA. [193]Department of Biosciences and Nutrition, Karolinska Institutet, Stockholm, Sweden. [194]Cancer Science Institute of Singapore, National University of Singapore, Singapore, Singapore. [195]Programme in Cancer & Stem Cell Biology, Duke-NUS Medical School, Singapore, Singapore. [196]SingHealth, Duke-NUS Institute of Precision Medicine, National Heart Centre Singapore, Singapore, Singapore. [197]Institute of Molecular and Cell Biology, Singapore, Singapore. [198]Laboratory of Cancer Epigenome, Division of Medical Science, National Cancer Centre Singapore, Singapore, Singapore. [199]Department of Medicine, Baylor College of Medicine, Houston, TX, USA. [200]National Cancer Centre Singapore, Singapore, Singapore. [201]BIOPIC, ICG and College of Life Sciences, Peking University, Beijing, China. [202]Vall d'Hebron Institute of Oncology (VHIO), Barcelona, Spain. [203]Department of Cancer Biology, Dana-Farber Cancer Institute, Boston, MA, USA. [204]Institute for Research in Biomedicine (IRB Barcelona), The Barcelona Institute of Science and Technology (BIST), Barcelona, Spain. [205]Department of Mathematics, Aarhus University, Aarhus, Denmark. [206]Institut Hospital del Mar d'Investigacions Mèdiques (IMIM), Barcelona, Spain. [207]Ontario Institute for Cancer Research, Toronto, Ontario, Canada. [208]King Faisal Specialist Hospital and Research Centre, Riyadh, Saudi Arabia. [209]DLR Project Management Agency, Bonn, Germany. [210]Genome Canada, Ottawa, Ontario, Canada. [211]Instituto Carlos Slim de la Salud, Mexico City, Mexico. [212]Federal Ministry of Education and Research, Berlin, Germany. [213]Institut Gustave Roussy, Villejuif, France. [214]Institut National du Cancer (INCA), Boulogne-Billancourt, France. [215]The Wellcome Trust, London, UK. [216]Prostate Cancer Canada, Toronto, Ontario, Canada. [217]National Human Genome Research Institute, National Institutes of Health, Bethesda, MD, USA. [218]Department of Biotechnology, Ministry of Science & Technology, Government of India, New Delhi, Delhi, India. [219]Science Writer, Garrett Park, MD, USA. [220]Cancer Research UK, London, UK. [221]Chinese Cancer Genome Consortium, Shenzhen, China. [222]Laboratory of Molecular Oncology, Key Laboratory of Carcinogenesis and Translational Research (Ministry of Education), Peking University Cancer Hospital & Institute, Beijing, China. [223]Key Laboratory of Carcinogenesis and Translational Research (Ministry of Education), Peking University Cancer Hospital & Institute, Beijing, China. [224]National Cancer Center, Tokyo, Japan. [225]German Cancer Aid, Bonn, Germany. [226]Division of Cancer Genomics, National Cancer Center Research Institute, National Cancer Center, Tokyo, Japan. [227]Laboratory of Molecular Medicine, Human Genome Center, The Institute of Medical Science, The University of Tokyo, Minato-ku, Tokyo, Japan. [228]Japan Agency for Medical Research and Development, Chiyoda-ku, Tokyo, Japan. [229]Medical Oncology, University and Hospital Trust of Verona, Verona, Italy. [230]University of Verona, Verona, Italy. [231]National Cancer Institute, National Institutes of Health, Bethesda, MD, USA. [232]CAPHRI Research School, Maastricht University, Maastricht, The Netherlands. [233]Laboratory for Medical Science Mathematics, RIKEN Center for Integrative Medical Sciences, Yokohama, Japan. [234]University of California San Diego, San Diego, CA, USA. [235]PDXen Biosystems, Seoul, South Korea. [236]Electronics and Telecommunications Research Institute, Daejeon, South Korea. [237]Children's Hospital of Philadelphia, Philadelphia, PA, USA. [238]University of Melbourne Centre for Cancer Research, Melbourne, Victoria, Australia. [239]Syntekabio, Daejon, South Korea. [240]AbbVie, North Chicago, IL, USA. [241]Genomics Research Program, Ontario Institute for Cancer Research, Toronto, Ontario, Canada. [242]Department of Pediatric Immunology, Hematology and Oncology, University Hospital, Heidelberg, Germany. [243]Heidelberg Institute for Stem Cell Technology and Experimental Medicine (HI-STEM), Heidelberg, Germany. [244]Seven Bridges, Charlestown, MA, USA. [245]Health Sciences Department of Biomedical Informatics, University of California San Diego, La Jolla, CA, USA. [246]Functional and Structural Genomics, German Cancer Research Center (DKFZ), Heidelberg, Germany. [247]Leidos Biomedical Research, McLean, VA, USA. [248]CSRA Incorporated, Fairfax, VA, USA. [249]Department of Internal Medicine, Stanford University, Stanford, CA, USA. [250]Clinical Bioinformatics, Swiss Institute of Bioinformatics, Geneva, Switzerland. [251]Institute for Pathology and Molecular Pathology, University Hospital Zurich, Zurich, Switzerland. [252]Institute of Molecular Life Sciences, University of Zurich, Zurich, Switzerland. [253]MIT Computer Science and Artificial Intelligence Laboratory, Massachusetts Institute of Technology, Cambridge, MA, USA. [254]Institute of Molecular Life Sciences and Swiss Institute of Bioinformatics, University of Zurich, Zurich, Switzerland. [255]Office of Cancer Genomics, National Cancer Institute, National Institutes of Health, Bethesda, MD, USA. [256]Computer Network Information Center, Chinese Academy of Sciences, Beijing, China. [257]Geneplus-Shenzhen, Shenzhen, China. [258]Dana-Farber/Boston Children's Cancer and Blood Disorders Center, Boston, MA, USA. [259]Department of Pediatrics, Harvard Medical School, Boston, MA, USA. [260]Technical University of Denmark, Lyngby, Denmark. [261]University of Copenhagen, Copenhagen, Denmark. [262]Department for BioMedical Research, University of Bern, Bern, Switzerland. [263]Department of Medical Oncology, Inselspital, University Hospital and University of Bern, Bern, Switzerland. [264]Graduate School for Cellular and Biomedical Sciences, University of Bern, Bern, Switzerland. [265]Department of Genitourinary Medical Oncology - Research, Division of Cancer Medicine, The University of Texas MD Anderson Cancer Center, Houston, TX, USA. [266]Department of Urology, Icahn School of Medicine at Mount Sinai, New York, NY, USA. [267]Korea Advanced Institute of Science and Technology, Daejeon, South Korea. [268]Science for Life Laboratory, Department of Cell and Molecular Biology, Uppsala University, Uppsala, Sweden. [269]Queensland Centre for Medical Genomics, Institute for Molecular Bioscience, The University of Queensland, Brisbane, Queensland, Australia. [270]University of Milano Bicocca, Monza, Italy. [271]Sir Peter MacCallum Department of Oncology, Peter MacCallum Cancer Centre, University of Melbourne, Melbourne, Victoria, Australia. [272]Center for Precision Health, School of Biomedical Informatics, The University of Texas Health Science Center, Houston, TX, USA. [273]Health Data Science Unit, University Clinics, Heidelberg, Germany. [274]Department for Biomedical Research, University of Bern, Bern, Switzerland. [275]Research Core Center, National Cancer Centre Korea, Goyang-si, South Korea. [276]Institute of Computer Science, Polish Academy of Sciences, Warsawa, Poland. [277]Harvard University, Cambridge, MA, USA. [278]Memorial Sloan Kettering Cancer Center, New York, NY, USA. [279]Department of Information Technology, Ghent University, Ghent, Belgium. [280]Department of Plant Biotechnology and Bioinformatics, Ghent University, Ghent, Belgium. [281]Yale School of Medicine, Yale University, New Haven, CT, USA. [282]Division of Hematology-Oncology, Samsung Medical Center, Sungkyunkwan University School of Medicine, Seoul, South Korea. [283]Samsung Advanced Institute for Health Sciences and Technology, Sungkyunkwan University School of Medicine, Seoul, South Korea. [284]Cheonan Industry-Academic Collaboration Foundation, Sangmyung University, Cheonan, South Korea. [285]Spanish National Cancer Research Centre, Madrid, Spain. [286]Department of Computer Science, Princeton University, Princeton, NJ, USA. [287]Bern Center for Precision Medicine, University Hospital of Bern, University of Bern, Bern, Switzerland. [288]Englander Institute for Precision Medicine, Weill Cornell Medicine and New York Presbyterian Hospital, New York, NY, USA. [289]Meyer Cancer Center, Weill Cornell Medicine, New York, NY, USA. [290]Pathology and Laboratory, Weill Cornell Medical College, New York, NY, USA. [291]cBio Center, Dana-Farber Cancer Institute, Harvard Medical School, Boston, MA, USA. [292]Department of Cell Biology, Harvard Medical School, Boston, MA, USA. [293]cBio Center, Dana-Farber Cancer Institute, Boston, MA, USA. [294]CREST, Japan Science and Technology Agency, Tokyo, Japan. [295]Department of Medical Science Mathematics, Medical Research Institute, Tokyo Medical and Dental University, Bunkyo-ku, Tokyo, Japan. [296]Laboratory for Medical Science Mathematics, Department of Biological Sciences, Graduate School of Science, The University of Tokyo, Bunkyo-ku, Tokyo, Japan. [297]Science for Life Laboratory, Department of Oncology-Pathology, Karolinska Institutet, Stockholm, Sweden. [298]Department of Gene Technology, Tallinn University of Technology, Tallinn, Estonia. [299]Genetics & Genome Biology Program, SickKids Research Institute, The Hospital for Sick Children, Toronto, Ontario, Canada. [300]Department of Information Technology, Ghent University, Interuniversitair Micro-Electronica Centrum (IMEC), Ghent, Belgium. [301]Science for Life Laboratory, Department of Immunology, Genetics and Pathology, Uppsala University, Uppsala, Sweden. [302]Oregon Health & Sciences University, Portland, OR, USA. [303]Department of Medicine and Therapeutics, The Chinese University of Hong Kong, Shatin, Hong Kong, China. [304]The University of Texas Health Science Center at Houston, Houston, TX, USA. [305]Department of Biomedical Informatics, College of Medicine, The Ohio State University, Columbus, OH, USA. [306]The Ohio State University Comprehensive Cancer Center (OSUCCC – James), Columbus, OH, USA. [307]The University of Texas School of Biomedical Informatics (SBMI) at Houston, Houston, TX, USA. [308]Department of

Biochemistry and Molecular Genetics, Feinberg School of Medicine, Northwestern University, Chicago, IL, USA. [309]Physics Division, Optimization and Systems Biology Lab, Massachusetts General Hospital, Boston, MA, USA. [310]Genome Science Division, Research Center for Advanced Science and Technology, The University of Tokyo, Tokyo, Japan. [311]Bioinformatics Group, Department of Computer Science, University of Leipzig, Leipzig, Germany. [312]Interdisciplinary Center for Bioinformatics, University of Leipzig, Leipzig, Germany. [313]Center for Bioinformatics and Functional Genomics, Cedars-Sinai Medical Center, Los Angeles, CA, USA. [314]Computational Biology, Leibniz Institute on Aging - Fritz Lipmann Institute (FLI), Jena, Germany. [315]Transcriptome Bioinformatics, LIFE Research Center for Civilization Diseases, University of Leipzig, Leipzig, Germany. [316]Center for Epigenetics, Van Andel Research Institute, Grand Rapids, MI, USA. [317]Institut d'Investigacions Biomèdiques August Pi i Sunyer (IDIBAPS), Barcelona, Spain. [318]Research Center for Advanced Science and Technology, The University of Tokyo, Minato-ku, Tokyo, Japan. [319]Van Andel Research Institute, Grand Rapids, MI, USA. [320]Cancer Epigenomics, German Cancer Research Center (DKFZ), Heidelberg, Germany. [321]Department of Biomedical Sciences, Cedars-Sinai Medical Center, Los Angeles, CA, USA. [322]The Hebrew University Faculty of Medicine, Jerusalem, Israel. [323]German Cancer Consortium (DKTK), German Cancer Research Center (DKFZ), Heidelberg, Germany. [324]Department of Pathology, Johns Hopkins University School of Medicine, Baltimore, MD, USA. [325]McKusick-Nathans Institute of Genetic Medicine, Sidney Kimmel Comprehensive Cancer Center, Johns Hopkins University School of Medicine, Baltimore, MD, USA. [326]Foundation Medicine, Cambridge, MA, USA. [327]Department of Biochemistry, Microbiology and Immunology, Faculty of Medicine, University of Ottawa, Ottawa, Ontario, Canada. [328]Cancer Research UK Cambridge Institute, University of Cambridge, Cambridge, UK. [329]University of Cambridge, Cambridge, UK. [330]Brandeis University, Waltham, MA, USA. [331]Hopp Children's Cancer Center (KiTZ), Heidelberg, Germany. [332]Pediatric Glioma Research Group, German Cancer Research Center (DKFZ), Heidelberg, Germany. [333]A. A. Kharkevich Institute of Information Transmission Problems, Moscow, Russia. [334]Oncology and Immunology, Dmitry Rogachev National Research Center of Pediatric Hematology, Moscow, Russia. [335]Skolkovo Institute of Science and Technology, Moscow, Russia. [336]Center for Medical Innovation, Seoul National University Hospital, Seoul, South Korea. [337]Department of Internal Medicine, Seoul National University Hospital, Seoul, South Korea. [338]Division of Genetics and Genomics, Boston Children's Hospital, Harvard Medical School, Boston, MA, USA. [339]School of Medicine/School of Mathematics and Statistics, University of St Andrews, St Andrews, UK. [340]Department of Genetics and Computational Biology, QIMR Berghofer Medical Research Institute, Brisbane, Queensland, Australia. [341]Institute for Molecular Bioscience, University of Queensland, Brisbane, Queensland, Australia. [342]Cancer Research Institute, Beth Israel Deaconess Medical Center, Boston, MA, USA. [343]Ben May Department for Cancer Research, Department of Human Genetics, The University of Chicago, Chicago, IL, USA. [344]Tri-Institutional PhD Program in Computational Biology and Medicine, Weill Cornell Medicine, New York, NY, USA. [345]Department of Bioengineering, and Department of Cellular and Molecular Medicine, Moores Cancer Center, University of California, San Diego, La Jolla, CA, USA. [346]Centre for Computational Biology, Duke-NUS Medical School, Singapore, Singapore. [347]Department of Computer Science, University of Helsinki, Helsinki, Finland. [348]Institute of Biotechnology, University of Helsinki, Helsinki, Finland. [349]Organismal and Evolutionary Biology Research Programme, University of Helsinki, Helsinki, Finland. [350]Programme in Cancer & Stem Cell Biology, Centre for Computational Biology, Duke-NUS Medical School, Singapore, Singapore. [351]Department of Applied Mathematics and Theoretical Physics, Centre for Mathematical Sciences, University of Cambridge, Cambridge, UK. [352]Department of Statistics, Columbia University, New York, NY, USA. [353]Duke-NUS Medical School, Singapore, Singapore. [354]School of Electronic Information and Communications, Huazhong University of Science and Technology, Wuhan, China. [355]The Kinghorn Cancer Centre, Cancer Division, Garvan Institute of Medical Research, University of New South Wales, Sydney, New South Wales, Australia. [356]MRC Human Genetics Unit, MRC IGMM, University of Edinburgh, Edinburgh, UK. [357]Bioinformatics Group, Division of Molecular Biology, Department of Biology, Faculty of Science, University of Zagreb, Zagreb, Croatia. [358]Department of Bioinformatics, Division of Cancer Genomics, National Cancer Center Research Institute, National Cancer Center, Tokyo, Japan. [359]University of Glasgow, Glasgow, UK. [360]Academic Department of Medical Genetics, University of Cambridge, Addenbrooke's Hospital, Cambridge, UK. [361]MRC Cancer Unit, University of Cambridge, Cambridge, UK. [362]The University of Cambridge School of Clinical Medicine, Cambridge, UK. [363]MRC-University of Glasgow Centre for Virus Research, Glasgow, UK. [364]Wolfson Wohl Cancer Research Centre, Institute of Cancer Sciences, University of Glasgow, Bearsden, UK. [365]School of Computing Science, University of Glasgow, Glasgow, UK. [366]South Western Sydney Clinical School, Faculty of Medicine, University of New South Wales, Liverpool, New South Wales, Australia. [367]West of Scotland Pancreatic Unit, Glasgow Royal Infirmary, Glasgow, UK. [368]University of Melbourne Centre for Cancer Research, Melbourne, Victoria, Australia. [369]Molecular and Medical Genetics, Oregon Health & Science University, Portland, OR, USA. [370]Department of Surgery, University of Melbourne, Parkville, Victoria, Australia. [371]The Murdoch Children's Research Institute, Royal Children's Hospital, Parkville, Victoria, Australia. [372]Walter + Eliza Hall Institute, Parkville, Victoria, Australia. [373]University of Cologne, Cologne, Germany. [374]The Edward S. Rogers Sr Department of Electrical and Computer Engineering, University of Toronto, Toronto, Ontario, Canada. [375]University of Ljubljana, Ljubljana, Slovenia. [376]Department of Public Health Sciences, The University of Chicago, Chicago, IL, USA. [377]Research Institute, NorthShore University HealthSystem, Evanston, IL, USA. [378]Department of Statistics, University of California Santa Cruz, Santa Cruz, CA, USA. [379]Cambridge University Hospitals NHS Foundation Trust, Cambridge, UK. [380]University of Toronto, Toronto, Ontario, Canada. [381]Department of Computer Science, Carleton College, Northfield, MN, USA. [382]Molecular and Medical Genetics, Oregon Health & Science University, Portland, OR, USA. [383]Center for Psychiatric Genetics, NorthShore University HealthSystem, Evanston, IL, USA. [384]Argmix Consulting, North Vancouver, British Columbia, Canada. [385]Department of Biostatistics, The University of Texas MD Anderson Cancer Center, Houston, TX, USA. [386]Department of Biostatistics, University of North Carolina at Chapel Hill, Chapel Hill, NC, USA. [387]The University of Texas MD Anderson Cancer Center, Houston, TX, USA. [388]Molecular and Medical Genetics, Knight Cancer Institute, Oregon Health & Science University, Portland, OR, USA. [389]Department of Health Sciences, Faculty of Medical Sciences, Kyushu University, Fukuoka, Japan. [390]Baylor College of Medicine, Houston, TX, USA. [391]Department of Applied Mathematics and Statistics, Johns Hopkins University, Baltimore, MD, USA. [392]Heinrich Pette Institute, Leibniz Institute for Experimental Virology, Hamburg, Germany. [393]University Medical Center Hamburg-Eppendorf, Bioinformatics Core, Hamburg, Germany. [394]Earlham Institute, Norwich, UK. [395]Norwich Medical School, University of East Anglia, Norwich, UK. [396]The Institute of Cancer Research, London, UK. [397]University of East Anglia, Norwich, UK. [398]German Center for Infection Research (DZIF), Partner Site Hamburg-Borstel-Lübeck-Riems, Hamburg, Germany. [399]Division of Molecular Genetics, German Cancer Research Center (DKFZ), Heidelberg, Germany. [400]Department of Pathology, The University of Texas MD Anderson Cancer Center, Houston, TX, USA. [401]Victorian Institute of Forensic Medicine, Southbank, Victoria, Australia. [402]Peter MacCallum Cancer Centre, University of Melbourne, Melbourne, Victoria, Australia. [403]University of Pennsylvania, Philadelphia, PA, USA. [404]Centre for Cancer Research, The Westmead Institute for Medical Research, Sydney, New South Wales, Australia. [405]Department of Gynaecological Oncology, Westmead Hospital, Sydney, New South Wales, Australia. [406]Genetics and Molecular Pathology, SA Pathology, Adelaide, South Australia, Australia. [407]Centre for Cancer Research, The Westmead Institute for Medical Research, The University of Sydney, Sydney, New South Wales, Australia. [408]Department of Gynaecological Oncology, Westmead Hospital, Sydney, New South Wales, Australia. [409]Garvan Institute of Medical Research, Darlinghurst, New South Wales, Australia. [410]Department of Clinical Pathology, University of Melbourne, Melbourne, Victoria, Australia. [411]Centre for Cancer Research, The Westmead Institute for Medical Research, The University of Sydney Sydney, Sydney, New South Wales, Australia. [412]Department of Gynaecological Oncology, Westmead Hospital, Sydney, New South Wales, Australia. [413]Westmead Clinical School, The Westmead Institute for Medical Research, New South Wales, Australia. [414]Department of Surgery, Pancreas Institute, University and Hospital Trust of Verona, Verona, Italy. [415]Department of Surgery, Princess Alexandra Hospital, Brisbane, Queensland, Australia. [416]Surgical Oncology Group, Diamantina Institute, The University of Queensland, Brisbane, Queensland, Australia. [417]Department of Diagnostics and Public Health, University and Hospital Trust of Verona, Verona, Italy. [418]ARC-Net Centre for Applied Research on Cancer, University and Hospital Trust of Verona, Verona, Italy. [419]Illawarra Shoalhaven Local Health District L3 Illawarra Cancer Care Centre, Wollongong Hospital, Wollongong, New South Wales, Australia. [420]Department of Pathology, University of Sydney, Sydney, New South Wales, Australia. [421]School of Biological Sciences, The University of Auckland, Auckland, New Zealand. [422]Department of Pathology and Diagnostics, University and Hospital Trust of Verona, Verona, Italy. [423]Department of Medicine, Section of Endocrinology, University and Hospital Trust of Verona, Verona, Italy. [424]Department of Pathology, Queen Elizabeth University Hospital, Glasgow, UK. [425]Department of Medical Oncology, Beatson West of Scotland Cancer Centre, Glasgow, UK. [426]Academic Unit of Surgery, School of Medicine, College of Medical, Veterinary and Life Sciences, University of Glasgow, Glasgow Royal Infirmary, Glasgow, UK. [427]Tissue Pathology and Diagnostic Oncology, Royal Prince Alfred Hospital, Camperdown, New South Wales, Australia. [428]Discipline of Surgery, Western Sydney University, Penrith, New South Wales, Australia. [429]Institute of Cancer Sciences, College of Medical Veterinary and Life Sciences, University of Glasgow, Glasgow, UK. [430]The Kinghorn Cancer Centre, Cancer Division, Garvan Institute of Medical Research, University of New South Wales, Sydney, New South Wales, Australia. [431]School of Environmental and Life Sciences, Faculty of Science, The University of Newcastle, Ourimbah, New South Wales, Australia. [432]Eastern Clinical School, Monash University, Melbourne, Victoria, Australia. [433]Epworth HealthCare, Richmond, Victoria, Australia. [434]Olivia Newton-John Cancer Research Institute, La Trobe University, Heidelberg, Victoria, Australia. [435]Melanoma Institute Australia, The University of Sydney, Wollstonecraft, New South Wales, Australia. [436]Children's Hospital at Westmead, The University of Sydney, Sydney, New South Wales, Australia. [437]Melanoma Institute Australia, The University of Sydney, Sydney, New South Wales, Australia. [438]Australian Institute of Tropical Health and Medicine, James Cook University, Douglas, Queensland, Australia. [439]Bioplatforms Australia, North Ryde, New South Wales, Australia. [440]Melanoma Institute Australia, Macquarie University, Wollstonecraft, New South Wales, Australia. [441]Children's Medical Research Institute, Sydney, New South Wales, Australia. [442]Melanoma Institute Australia, The University of Sydney, Wollstonecraft, New South Wales, Australia. [443]Centre for Cancer Research, The Westmead Millennium Institute for Medical Research, University of Sydney, Westmead Hospital, Sydney, New South Wales, Australia. [444]Translational Cancer Research Centre, The University of Sydney at the Westmead Institute, Sydney, New South Wales, Australia. [445]Discipline of Pathology, Sydney Medical School, The University of Sydney, Sydney, New South Wales, Australia. [446]School of Mathematics and Statistics, The University of Sydney, Sydney, New South Wales, Australia. [447]Melanoma Institute Australia, The University of Sydney, Wollstonecraft, New South Wales, Australia. [448]Royal Prince Alfred Hospital, Sydney, New South Wales, Australia. [449]Diagnostic Development, Ontario Institute for Cancer Research, Toronto, Ontario, Canada. [450]Ontario Tumour Bank, Ontario Institute for Cancer Research, Toronto, Ontario, Canada. [451]PanCuRx Translational Research Initiative,

Ontario Institute for Cancer Research, Toronto, Ontario, Canada. [452]BioSpecimen Sciences Program, University Health Network, Toronto, Ontario, Canada. [453]Hepatobiliary/Pancreatic Surgical Oncology Program, University Health Network, Toronto, Ontario, Canada. [454]Lunenfeld-Tanenbaum Research Institute, Mount Sinai Hospital, Toronto, Ontario, Canada. [455]Division of Medical Oncology, Princess Margaret Cancer Centre, Toronto, Ontario, Canada. [456]University of Nebraska Medical Center, Omaha, NE, USA. [457]BioSpecimen Sciences Program, University Health Network, Toronto, Ontario, Canada. [458]Transformative Pathology, Ontario Institute for Cancer Research, Toronto, Ontario, Canada. [459]University Health Network, Princess Margaret Cancer Centre, Toronto, Ontario, Canada. [460]Department of Health Sciences Research, Mayo Clinic, Rochester, MN, USA. [461]BioSpecimen Sciences, Laboratory Medicine (Toronto), Medical Biophysics, PanCuRX, Toronto, Ontario, Canada. [462]Department of Laboratory Medicine and Pathobiology, University of Toronto, Toronto, Ontario, Canada. [463]Department of Pathology, Human Oncology and Pathogenesis Program, Memorial Sloan Kettering Cancer Center, New York, NY, USA. [464]Department of Medical Biophysics, University of Toronto, Toronto, Ontario, Canada. [465]Department of Biochemistry and Molecular Medicine, University California at Davis, Sacramento, CA, USA. [466]Human Longevity, San Diego, CA, USA. [467]Department of Surgical Oncology, Princess Margaret Cancer Centre, Toronto, Ontario, Canada. [468]Genome Informatics Program, Ontario Institute for Cancer Research, Toronto, Ontario, Canada. [469]STTARR Innovation Facility, Princess Margaret Cancer Centre, Toronto, Ontario, Canada. [470]Department of Pathology, Toronto General Hospital, Toronto, Ontario, Canada. [471]CRUK Manchester Institute and Centre, Manchester, UK. [472]Department of Radiation Oncology, University of Toronto, Toronto, Ontario, Canada. [473]Manchester Cancer Research Centre, Cancer Division, FBMH, University of Manchester, Manchester, UK. [474]Radiation Medicine Program, Princess Margaret Cancer Centre, Toronto, Ontario, Canada. [475]Hefei University of Technology, Anhui, China. [476]State Key Laboratory of Cancer Biology and Xijing Hospital of Digestive Diseases, Fourth Military Medical University, Shaanxi, China. [477]Fourth Military Medical University, Shaanxi, China. [478]Laboratory of Molecular Oncology, Key Laboratory of Carcinogenesis and Translational Research (Ministry of Education), Peking University Cancer Hospital & Institute, Beijing, China. [479]Department of Surgery, Ruijin Hospital, Shanghai Jiaotong University School of Medicine, Shanghai, China. [480]Leeds Institute of Medical Research, University of Leeds, St James's University Hospital, Leeds, UK. [481]Canadian Center for Computational Genomics, McGill University, Montreal, Quebec, Canada. [482]Department of Human Genetics, McGill University, Montreal, Quebec, Canada. [483]International Agency for Research on Cancer, Lyon, France. [484]McGill University and Genome Quebec Innovation Centre, Montreal, Quebec, Canada. [485]St James Institute of Oncology, University of Leeds, St James's University Hospital, Leeds, UK. [486]Institute of Mathematics and Computer Science, University of Latvia, Riga, Latvia. [487]Centre National de Génotypage, CEA - Institute de Génomique, Evry, France. [488]Department of Oncology, Gil Medical Center, Gachon University, Incheon, South Korea. [489]Department of Molecular Oncology, BC Cancer Agency, Vancouver, British Columbia, Canada. [490]Los Alamos National Laboratory, Los Alamos, NM, USA. [491]Department of Genetics, Institute for Cancer Research, Oslo University Hospital, The Norwegian Radium Hospital, Oslo, Norway. [492]Lund University, Lund, Sweden. [493]Translational Research Lab, Centre Léon Bérard, Lyon, France. [494]Department of Molecular Biology, Faculty of Science, Radboud Institute for Molecular Life Sciences, Radboud University, Nijmegen, The Netherlands. [495]Department of Pathology, Brigham and Women's Hospital, Harvard Medical School, Boston, MA, USA. [496]Department of Molecular Pathology, The Netherlands Cancer Institute, Amsterdam, The Netherlands. [497]Li Ka Shing Centre, Cancer Research UK Cambridge Institute, University of Cambridge, Cambridge, UK. [498]Department of Oncology, University of Cambridge, Cambridge, UK. [499]Breast Cancer Translational Research Laboratory J. C. Heuson, Institut Jules Bordet, Brussels, Belgium. [500]Laboratory for Translational Breast Cancer Research, Department of Oncology, KU Leuven, Leuven, Belgium. [501]Translational Cancer Research Unit, GZA Hospitals St-Augustinus, Center for Oncological Research, Faculty of Medicine and Health Sciences, University of Antwerp, Antwerp, Belgium. [502]Department of Gynecology & Obstetrics and Department of Clinical Sciences, Skåne University Hospital, Lund University, Lund, Sweden. [503]Icelandic Cancer Registry, Icelandic Cancer Society, Reykjavik, Iceland. [504]Department of Medical Oncology, Josephine Nefkens Institute and Cancer Genomics Centre, Erasmus Medical Center, Rotterdam, The Netherlands. [505]National Genotyping Center, Institute of Biomedical Sciences, Academia Sinica, Taipei, Taiwan. [506]Department of Pathology, Oslo University Hospital Ulleval, Oslo, Norway. [507]Faculty of Medicine and Institute of Clinical Medicine, University of Oslo, Oslo, Norway. [508]Department of Pathology, Memorial Sloan Kettering Cancer Center, New York, NY, USA. [509]Department of Pathology, Skåne University Hospital, Lund University, Lund, Sweden. [510]Department of Pathology, Academic Medical Center, Amsterdam, The Netherlands. [511]Department of Pathology, College of Medicine, Hanyang University, Seoul, South Korea. [512]Department of Pathology, Asan Medical Center, College of Medicine, Ulsan University, Songpa-gu, Seoul, South Korea. [513]The Netherlands Cancer Institute, Amsterdam, The Netherlands. [514]Department of Surgery, Dana-Farber Cancer Institute, Brigham and Women's Hospital, Boston, MA, USA. [515]Department of Surgery, Memorial Sloan Kettering Cancer Center, New York, NY, USA. [516]Department of Clinical Science, University of Bergen, Bergen, Norway. [517]Morgan Welch Inflammatory Breast Cancer Research Program and Clinic, The University of Texas MD Anderson Cancer Center, Houston, TX, USA. [518]The University of Queensland Centre for Clinical Research, The Royal Brisbane & Women's Hospital, Herston, Queensland, Australia. [519]Department of Pathology, Institut Jules Bordet, Brussels, Belgium. [520]Institute for Bioengineering and Biopharmaceutical Research (IBBR), Hanyang University, Seoul, South Korea. [521]University of Oslo, Oslo, Norway. [522]Institut Bergonié, Bordeaux, France. [523]Department of Research Oncology, Guy's Hospital, King's Health Partners AHSC, King's College London School of Medicine, London, UK. [524]University Hospital of Minjoz, INSERM UMR 1098, Besançon, France. [525]Cambridge Breast Unit, Addenbrooke's Hospital, Cambridge University Hospital NHS Foundation Trust and NIHR Cambridge Biomedical Research Centre, Cambridge, UK. [526]East of Scotland Breast Service, Ninewells Hospital, Aberdeen, UK. [527]Oncologie Sénologie, ICM Institut Régional du Cancer, Montpellier, France. [528]Department of Radiation Oncology, Radboud University Nijmegen Medical Centre, Nijmegen, The Netherlands. [529]University of Iceland, Reykjavik, Iceland. [530]Dundee Cancer Centre, Ninewells Hospital, Dundee, UK. [531]Institut Curie, INSERM Unit 830, Paris, France. [532]Department of Laboratory Medicine, Radboud University Nijmegen Medical Centre, Nijmegen, The Netherlands. [533]Department of General Surgery, Singapore General Hospital, Singapore, Singapore. [534]INCa-Synergie, Centre Léon Bérard, Universite Lyon, Lyon, France. [535]Giovanni Paolo II/I.R.C.C.S. Cancer Institute, Bari, Italy. [536]Department of Biopathology, Centre Léon Bérard, Lyon, France. [537]Université Claude Bernard Lyon 1, Villeurbanne, France. [538]NCCS-VARI Translational Research Laboratory, National Cancer Centre Singapore, Singapore, Singapore. [539]Department of Pathology, Erasmus Medical Center Rotterdam, Rotterdam, The Netherlands. [540]Division of Molecular Carcinogenesis, The Netherlands Cancer Institute, Amsterdam, The Netherlands. [541]Institute of Human Genetics, Christian-Albrechts-University, Kiel, Germany. [542]Institute of Human Genetics, University of Ulm, Ulm, Germany. [543]University Hospital of Ulm, Ulm, Germany. [544]Hematopathology Section, Institute of Pathology, Christian-Albrechts-University, Kiel, Germany. [545]Department of Human Genetics, Hannover Medical School, Hannover, Germany. [546]Department of Pediatric Oncology, Hematology and Clinical Immunology, Heinrich-Heine-University, Düsseldorf, Germany. [547]Department of Internal Medicine/Hematology, Friedrich-Ebert-Hospital, Neumünster, Germany. [548]Pediatric Hematology and Oncology, University Hospital Muenster, Muenster, Germany. [549]Department of Pediatrics, University Hospital Schleswig-Holstein, Kiel, Germany. [550]Department of Medicine II, University of Würzburg, Würzburg, Germany. [551]Senckenberg Institute of Pathology, University of Frankfurt Medical School, Frankfurt, Germany. [552]Institute of Pathology, Charité–University Medicine Berlin, Berlin, Germany. [553]Department for Internal Medicine II, University Hospital Schleswig-Holstein, Kiel, Germany. [554]Institute for Medical Informatics Statistics and Epidemiology, University of Leipzig, Leipzig, Germany. [555]Department of Hematology and Oncology, Georg-Augusts-University of Göttingen, Göttingen, Germany. [556]Institute of Cell Biology (Cancer Research), University of Duisburg-Essen, Essen, Germany. [557]MVZ Department of Oncology, PraxisClinic am Johannisplatz, Leipzig, Germany. [558]Institute of Pathology, Ulm University and University Hospital of Ulm, Ulm, Germany. [559]Department of Pathology, Robert-Bosch-Hospital, Stuttgart, Germany. [560]Pediatric Hematology and Oncology, University Hospital Giessen, Giessen, Germany. [561]Institute of Clinical Molecular Biology, Christian-Albrechts-University, Kiel, Germany. [562]Institute of Pathology, University of Wuerzburg, Wuerzburg, Germany. [563]Department of General Internal Medicine, University Kiel, Kiel, Germany. [564]Clinic for Hematology and Oncology, St-Antonius-Hospital, Eschweiler, Germany. [565]Department for Internal Medicine III, University of Ulm and University Hospital of Ulm, Ulm, Germany. [566]Neuroblastoma Genomics, German Cancer Research Center (DKFZ), Heidelberg, Germany. [567]Department of Pediatric Oncology and Hematology, University of Cologne, Cologne, Germany. [568]University of Düsseldorf, Düsseldorf, Germany. [569]Department of Vertebrate Genomics/Otto Warburg Laboratory Gene Regulation and Systems Biology of Cancer, Max Planck Institute for Molecular Genetics, Berlin, Germany. [570]St Jude Children's Research Hospital, Memphis, TN, USA. [571]Heidelberg University Hospital, Heidelberg, Germany. [572]Genomics and Proteomics Core Facility High Throughput Sequencing Unit, German Cancer Research Center (DKFZ), Heidelberg, Germany. [573]Epigenomics and Cancer Risk Factors, German Cancer Research Center (DKFZ), Heidelberg, Germany. [574]University Medical Center Hamburg-Eppendorf, Hamburg, Germany. [575]Martini-Clinic, Prostate Cancer Center, University Medical Center Hamburg-Eppendorf, Hamburg, Germany. [576]Institute of Pathology, University Medical Center Hamburg-Eppendorf, Hamburg, Germany. [577]Division of Cancer Genome Research, German Cancer Research Center (DKFZ), Heidelberg, Germany. [578]National Institute of Biomedical Genomics, Kalyani, India. [579]Advanced Centre for Treatment Research & Education in Cancer, Tata Memorial Centre, Navi Mumbai, India. [580]Department of Pathology, General Hospital of Treviso, Department of Medicine, University of Padua, Treviso, Italy. [581]Department of Medicine (DIMED), Surgical Pathology Unit, University of Padua, Padua, Italy. [582]Department of Hepatobiliary and Pancreatic Oncology, Hepatobiliary and Pancreatic Surgery Division, Division of Pathology and Clinical Laboratories, National Cancer Center Hospital, Chuo-ku, Tokyo, Japan. [583]Department of Pathology, Keio University School of Medicine, Tokyo, Japan. [584]Department of Hepatobiliary and Pancreatic Oncology, National Cancer Center Hospital, Tokyo, Japan. [585]Department of Pathology, Graduate School of Medicine, The University of Tokyo, Bunkyo-ku, Tokyo, Japan. [586]Preventive Medicine, Graduate School of Medicine, The University of Tokyo, Tokyo, Japan. [587]Gastric Surgery Division, Division of Pathology and Clinical Laboratories, National Cancer Center Hospital, Tokyo, Japan. [588]Department of Gastroenterology and Hepatology, Yokohama City University Graduate School of Medicine, Kanagawa, Japan. [589]Laboratory of Molecular Medicine, Human Genome Center, The Institute of Medical Science, University of Tokyo, Tokyo, Japan. [590]Department of Cancer Genome Informatics, Graduate School of Medicine, Osaka University, Osaka, Japan. [591]Hiroshima University, Hiroshima, Japan. [592]Tokyo Women's Medical University, Tokyo, Japan. [593]Osaka International Cancer Center, Osaka, Japan. [594]Wakayama Medical University, Wakayama, Japan. [595]Hokkaido University, Sapporo, Japan. [596]Division of Medical Oncology, National Cancer Centre, Singapore, Singapore. [597]Cholangiocarcinoma Screening and Care Program and Liver Fluke and Cholangiocarcinoma Research Centre, Faculty of Medicine, Khon Kaen University, Khon Kaen, Thailand. [598]Lymphoma Genomic Translational Research Laboratory, National Cancer

Centre, Singapore, Singapore. [599]Center of Digestive Diseases and Liver Transplantation, Fundeni Clinical Institute, Bucharest, Romania. [600]Division of Hepatobiliary and Pancreatic Surgery, Department of Surgery, School of Medicine, Keimyung University Dongsan Medical Center, Daegu, South Korea. [601]Pathology, Hospital Clinic, Institut d'Investigacions Biomèdiques August Pi i Sunyer (IDIBAPS), University of Barcelona, Barcelona, Spain. [602]Hematology, Hospital Clinic, Institut d'Investigacions Biomèdiques August Pi i Sunyer (IDIBAPS), University of Barcelona, Barcelona, Spain. [603]Department of Biochemistry and Molecular Biology, Faculty of Medicine, University Institute of Oncology-IUOPA, Oviedo, Spain. [604]Anatomia Patológica, Hospital Clinic, Institut d'Investigacions Biomèdiques August Pi i Sunyer (IDIBAPS), University of Barcelona, Barcelona, Spain. [605]Spanish Ministry of Science and Innovation, Madrid, Spain. [606]Royal National Orthopaedic Hospital (Bolsover), London, UK. [607]Department of Pathology, Oslo University Hospital, The Norwegian Radium Hospital, Oslo, Norway. [608]Institute of Clinical Medicine and Institute of Oral Biology, University of Oslo, Oslo, Norway. [609]Research Department of Pathology, University College London Cancer Institute, London, UK. [610]East Anglian Medical Genetics Service, Cambridge University Hospitals NHS Foundation Trust, Cambridge, UK. [611]Royal National Orthopaedic Hospital (Stanmore), London, UK. [612]Division of Orthopaedic Surgery, Oslo University Hospital, Oslo, Norway. [613]Department of Pathology (Research), University College London Cancer Institute, London, UK. [614]Radcliffe Department of Medicine, University of Oxford, Oxford, UK. [615]University of Pavia, Pavia, Italy. [616]Karolinska Institute, Stockholm, Sweden. [617]Wellcome Sanger Institute, Hinxton, UK. [618]University of Oxford, Oxford, UK. [619]Salford Royal NHS Foundation Trust, Salford, UK. [620]Gloucester Royal Hospital, Gloucester, UK. [621]Royal Stoke University Hospital, Stoke-on-Trent, UK. [622]St Thomas's Hospital, London, UK. [623]Imperial College NHS Trust, Imperial College London, London, UK. [624]Department of Histopathology, Salford Royal NHS Foundation Trust, Salford, UK. [625]Faculty of Biology, Medicine and Health, The University of Manchester, Manchester, UK. [626]Edinburgh Royal Infirmary, Edinburgh, UK. [627]Barking Havering and Redbridge University Hospitals NHS Trust, Romford, UK. [628]King's College London and Guy's and St Thomas' NHS Foundation Trust, London, UK. [629]Cambridge Oesophagogastric Centre, Cambridge University Hospitals NHS Foundation Trust, Cambridge, UK. [630]Nottingham University Hospitals NHS Trust, Nottingham, UK. [631]St Luke's Cancer Centre, Royal Surrey County Hospital NHS Foundation Trust, Guildford, UK. [632]University of North Carolina at Chapel Hill, Chapel Hill, NC, USA. [633]Norfolk and Norwich University Hospital NHS Trust, Norwich, UK. [634]University Hospitals Coventry and Warwickshire NHS Trust, Coventry, UK. [635]University Hospitals Birmingham NHS Foundation Trust, Birmingham, UK. [636]Centre for Cancer Research and Cell Biology, Queen's University, Belfast, UK. [637]School of Cancer Sciences, Faculty of Medicine, University of Southampton, Southampton, UK. [638]Wythenshawe Hospital, Manchester, UK. [639]Barts Cancer Institute, Barts and the London School of Medicine and Dentistry, Queen Mary University of London, London, UK. [640]Royal Marsden NHS Foundation Trust, London and Sutton, London, UK. [641]University Hospital Southampton NHS Foundation Trust, Southampton, UK. [642]HCA Laboratories, London, UK. [643]University of Liverpool, Liverpool, UK. [644]Academic Urology Group, Department of Surgery, University of Cambridge, Cambridge, UK. [645]University of Oxford, Oxford, Oxford, UK. [646]Department of Urology, James Buchanan Brady Urological Institute, Johns Hopkins University School of Medicine, Baltimore, MD, USA. [647]Second Military Medical University, Shanghai, China. [648]Department of Surgery and Cancer, Imperial College London, London, UK. [649]The Chinese University of Hong Kong, Shatin, Hong Kong, China. [650]Nuffield Department of Surgical Sciences, John Radcliffe Hospital, University of Oxford, Headington, Oxford, UK. [651]Department of Histopathology, Cambridge University Hospitals NHS Foundation Trust, Cambridge, UK. [652]Department of Bioinformatics and Computational Biology and Department of Systems Biology, The University of Texas MD Anderson Cancer Center, Houston, TX, USA. [653]Laboratory of Pathology, Center for Cancer Research, National Cancer Institute, Bethesda, MD, USA. [654]Canada's Michael Smith Genome Sciences Center, BC Cancer Agency, Vancouver, British Columbia, Canada. [655]Center for Molecular Oncology, Memorial Sloan Kettering Cancer Center, New York, NY, USA. [656]University Health Network, Toronto, Ontario, Canada. [657]Department of Pathology and Laboratory Medicine, School of Medicine, University of North Carolina at Chapel Hill, Chapel Hill, NC, USA. [658]Department of Population and Quantitative Health Sciences, Case Western Reserve University School of Medicine, Cleveland, OH, USA. [659]Research Health Analytics and Informatics, University Hospitals Cleveland Medical Center, Cleveland, OH, USA. [660]Arnie Charbonneau Cancer Institute, University of Calgary, Calgary, Alberta, Canada. [661]Department of Surgery and Department of Oncology, University of Calgary, Calgary, Alberta, Canada. [662]Buck Institute for Research on Aging, Novato, CA, USA. [663]Duke University Medical Center, Durham, NC, USA. [664]USC Norris Comprehensive Cancer Center, University of Southern California, Los Angeles, CA, USA. [665]The Preston Robert Tisch Brain Tumor Center, Duke University Medical Center, Durham, NC, USA. [666]Department of Dermatology and Department of Pathology, Yale University, New Haven, CT, USA. [667]Fox Chase Cancer Center, Philadelphia, PA, USA. [668]Department of Surgery, Division of Thoracic Surgery, The Johns Hopkins University School of Medicine, Baltimore, MD, USA. [669]University of Michigan Comprehensive Cancer Center, Ann Arbor, MI, USA. [670]University of Alabama at Birmingham, Birmingham, AL, USA. [671]Division of Anatomic Pathology, Mayo Clinic, Rochester, MN, USA. [672]Division of Experimental Pathology, Mayo Clinic, Rochester, MN, USA. [673]Department of Oncology, The Johns Hopkins School of Medicine, The Sidney Kimmel Comprehensive Cancer Center at Johns Hopkins University, Baltimore, MD, USA. [674]International Genomics Consortium, Phoenix, AZ, USA. [675]Department of Pediatrics and Department of Genetics, University of North Carolina at Chapel Hill, Chapel Hill, NC, USA. [676]Department of Pathology, UPMC Shadyside, Pittsburgh, PA, USA. [677]Center for Cancer Genomics, National Cancer Institute, National Institutes of Health, Bethesda, MD, USA. [678]Department of Neuro-Oncology, Istituto Neurologico Besta, Milan, Italy. [679]University of Queensland Thoracic Research Centre, The Prince Charles Hospital, Brisbane, Queensland, Australia. [680]Department of Neurosurgery, University of Florida, Gainesville, FL, USA. [681]Center for Biomedical Informatics, Harvard Medical School, Boston, MA, USA. [682]Department of Cancer Biology, The University of Texas MD Anderson Cancer Center, Houston, TX, USA. [683]Department of Surgical Oncology, The University of Texas MD Anderson Cancer Center, Houston, TX, USA. [684]Division of Gastroenterology and Hepatology, Mayo Clinic, Rochester, MN, USA. [685]Sylvester Comprehensive Cancer Center, University of Miami, Miami, FL, USA. [686]Department of Internal Medicine, Division of Medical Oncology, Lineberger Comprehensive Cancer Center, University of North Carolina at Chapel Hill, Chapel Hill, NC, USA. [687]University of Tennessee Health Science Center for Cancer Research, Memphis, TN, USA. [688]Centre for Translational and Applied Genomics, British Columbia Cancer Agency, Vancouver, British Columbia, Canada. [689]Department of Pathology & Immunology, Baylor College of Medicine, Houston, TX, USA. [690]Michael E. DeBakey Veterans Affairs Medical Center, Houston, TX, USA. [691]Carolina Center for Genome Sciences, University of North Carolina at Chapel Hill, Chapel Hill, NC, USA. [692]Canada's Michael Smith Genome Sciences Centre, BC Cancer Agency, Vancouver, British Columbia, Canada. [693]Indivumed, Hamburg, Germany. [694]Division of Hepatobiliary and Pancreatic Surgery, Department of Surgery, School of Medicine, Keimyung University Dong-san Medical Center, Daegu, South Korea. [695]Women's Cancer Program at the Samuel Oschin Comprehensive Cancer Institute, Cedars-Sinai Medical Center, Los Angeles, CA, USA. [696]Department of Surgery, School of Medicine and Health Science, The George Washington University, Washington, DC, USA. [697]Endocrine Oncology Branch, Center for Cancer Research, National Cancer Institute, National Institutes of Health, Bethesda, MD, USA. [698]National Cancer Center, Gyeonggi, South Korea. [699]ILSbio, LLC Biobank, Chestertown, MD, USA. [700]Gynecologic Oncology, NYU Laura and Isaac Perlmutter Cancer Center, New York University, New York, NY, USA. [701]Division of Oncology, Stem Cell Biology Section, Washington University School of Medicine, St Louis, MO, USA. [702]Urologic Oncology Branch, Center for Cancer Research, National Cancer Institute, National Institutes of Health, Bethesda, MD, USA. [703]Institute for Systems Biology, Seattle, WA, USA. [704]Center for Personalized Medicine, Department of Pathology and Laboratory Medicine, Children's Hospital Los Angeles, Los Angeles, CA, USA. [705]Institute for Genomic Medicine, Nationwide Children's Hospital, Columbus, OH, USA. [706]Department of Surgery, Duke University, Durham, NC, USA. [707]Department of Obstetrics, Gynecology and Reproductive Services, University of California San Francisco, San Francisco, CA, USA. [708]Department of Neurology and Department of Neurosurgery, Henry Ford Hospital, Detroit, MI, USA. [709]Knight Cancer Institute, Oregon Health & Science University, Portland, OR, USA. [710]Department of Pathology, Roswell Park Cancer Institute, Buffalo, NY, USA. [711]Department of Obstetrics and Gynecology, Division of Gynecologic Oncology, Washington University School of Medicine, St Louis, MO, USA. [712]Department of Palliative, Rehabilitation and Integrative Medicine, The University of Texas MD Anderson Cancer Center, Houston, TX, USA. [713]Penrose St Francis Health Services, Colorado Springs, CO, USA. [714]The University of Chicago, Chicago, IL, USA. [715]Department of Neurology, Mayo Clinic, Rochester, MN, USA. [716]Center for Liver Cancer, Research Institute and Hospital, National Cancer Center, Gyeonggi, South Korea. [717]Department of Genetics and Lineberger Comprehensive Cancer Center, University of North Carolina at Chapel Hill, Chapel Hill, NC, USA. [718]NYU Langone Medical Center, New York, NY, USA. [719]Department of Hematology and Medical Oncology, Cleveland Clinic, Cleveland, OH, USA. [720]Department of Genetics, Department of Pathology and Laboratory Medicine, School of Medicine, University of North Carolina at Chapel Hill, Chapel Hill, NC, USA. [721]Helen F. Graham Cancer Center at Christiana Care Health Systems, Newark, DE, USA. [722]Cureline, South San Francisco, CA, USA. [723]Department of Obstetrics and Gynecology, Medical College of Wisconsin, Milwaukee, WI, USA. [724]Hematology and Medical Oncology, Winship Cancer Institute of Emory University, Atlanta, GA, USA. [725]Vanderbilt Ingram Cancer Center, Vanderbilt University, Nashville, TN, USA. [726]Ohio State University College of Medicine and Arthur G. James Comprehensive Cancer Center, Columbus, OH, USA. [727]Research Computing Center, University of North Carolina at Chapel Hill, Chapel Hill, NC, USA. [728]Analytical Biological Services, Wilmington, DE, USA. [729]Department of Dermatology, University Hospital Essen, Westdeutsches Tumorzentrum and German Cancer Consortium, Essen, Germany. [730]University of Pittsburgh, Pittsburgh, PA, USA. [731]Murtha Cancer Center, Walter Reed National Military Medical Center, Bethesda, MD, USA. [732]Brigham and Women's Hospital, Harvard Medical School, Boston, MA, USA. [733]Department of Surgery, Memorial Sloan Kettering Cancer Center, New York, NY, USA. [734]Department of Gynecologic Oncology and Reproductive Medicine, and Center for RNA Interference and Non-Coding RNA, The University of Texas MD Anderson Cancer Center, Houston, TX, USA. [735]Department of Urology, Mayo Clinic, Rochester, MN, USA. [736]Department of Surgery, Johns Hopkins University School of Medicine, Baltimore, MD, USA. [737]Department of Neurosurgery, Department of Hematology and Department of Medical Oncology, Winship Cancer Institute and School of Medicine, Emory University, Atlanta, GA, USA. [738]Georgia Regents University Cancer Center, Augusta, GA, USA. [739]Thoracic Oncology Laboratory, Mayo Clinic, Rochester, MN, USA. [740]Institute for Genomic Medicine, Nationwide Children's Hospital, Columbus, OH, USA. [741]Department of Obstetrics & Gynecology, Division of Gynecologic Oncology, Mayo Clinic, Rochester, MN, USA. [742]International Institute for Molecular Oncology, Poznań, Poland. [743]Poznan University of Medical Sciences, Poznań, Poland. [744]Edison Family Center for Genome Sciences and Systems Biology, Washington University, St Louis, MO, USA. [745]These authors jointly supervised this work: Peter J. Campbell, Gad Getz, Jan O. Korbel, Joshua M. Stuart, Lincoln D. Stein. *e-mail: pc8@sanger.ac.uk; gadgetz@broadinstitute.org; korbel@embl.de; jstuart@ucsc.edu; lincoln.stein@gmail.com
